# Transcriptomic response of the mycoparasitic fungus *Trichoderma atroviride *to the presence of a fungal prey

**DOI:** 10.1186/1471-2164-10-567

**Published:** 2009-11-30

**Authors:** Verena Seidl, Lifu Song, Erika Lindquist, Sabine Gruber, Alexeji Koptchinskiy, Susanne Zeilinger, Monika Schmoll, Pedro Martínez, Jibin Sun, Igor Grigoriev, Alfredo Herrera-Estrella, Scott E Baker, Christian P Kubicek

**Affiliations:** 1 Research Area Gene Technology and Applied Biochemistry, Institute of Chemical Engineering, Vienna University of Technology, Getreidemarkt 9/166, A-1060 Vienna, Austria; 2 Tianjin Institute of Industrial Biotechnology, Chinese Academy of Sciences, West Seven Road 18, Airport Industrial Park, Tianjin 300308, PR China; 3 Joint Genome Institute, US Department of Energy, Walnut Creek, CA USA; 4 Laboratorio Nacional de Genómica para la Biodiversidada and Departamento de Ingeniería Genética, Cinvestav Campus Guanajuato, Km 9.6 Libramiento Norte Carretera Irapuato-León, A.P. 629, Irapuato 36500, Guanajuato, Mexico; 5 Pacific Northwest National Laboratory, P.O. Box 999, Richland, WA 99352, USA

## Abstract

**Background:**

Combating the action of plant pathogenic microorganisms by mycoparasitic fungi has been announced as an attractive biological alternative to the use of chemical fungicides since two decades. The fungal genus *Trichoderma *includes a high number of taxa which are able to recognize, combat and finally besiege and kill their prey. Only fragments of the biochemical processes related to this ability have been uncovered so far, however.

**Results:**

We analyzed genome-wide gene expression changes during the begin of physical contact between *Trichoderma atroviride *and two plant pathogens *Botrytis cinerea *and *Rhizoctonia solani*, and compared with gene expression patterns of mycelial and conidiating cultures, respectively. About 3000 ESTs, representing about 900 genes, were obtained from each of these three growth conditions. 66 genes, represented by 442 ESTs, were specifically and significantly overexpressed during onset of mycoparasitism, and the expression of a subset thereof was verified by expression analysis. The upregulated genes comprised 18 KOG groups, but were most abundant from the groups representing posttranslational processing, and amino acid metabolism, and included components of the stress response, reaction to nitrogen shortage, signal transduction and lipid catabolism. Metabolic network analysis confirmed the upregulation of the genes for amino acid biosynthesis and of those involved in the catabolism of lipids and aminosugars.

**Conclusion:**

The analysis of the genes overexpressed during the onset of mycoparasitism in *T. atroviride *has revealed that the fungus reacts to this condition with several previously undetected physiological reactions. These data enable a new and more comprehensive interpretation of the physiology of mycoparasitism, and will aid in the selection of traits for improvement of biocontrol strains by recombinant techniques.

## Background

Evolution has driven fungi to develop manifold different lifestyles which enable them to successfully thrive in their habitat. These include saprotrophic growth, parasitism on plants and animals, symbiosis with plants and algae and many more [1]. Thereby, a small group of genera and taxa within the order *Hypocreales *have adopted the ability parasite other fungi [2]. The ability to utilize other fungi as a source of nutrients and thus e.g. combat the action of plant pathogenic fungi by antagonistic or mycoparasitic fungi is an attractive biological alternative to the use of chemical fungicides [3]. Members of the fungal genus *Hypocrea/Trichoderma *(*Ascomycota, Hypocreales, Hypocreaceae*) contain many prominent examples of such biocontrol agents, because they not only antagonize plant-pathogenic fungi [4,5], but are often also rhizosphere competent and can even enhance plant growth and elicit plant defence responses [5,6].

Development of improved biocontrol agents for agricultural applications requires an understanding of the biological principle of their action. Consequently, some molecular aspects of biocontrol by *Trichoderma *spp. - such as the regulation and role of cell wall hydrolytic enzymes and antagonistic secondary metabolites - have been studied [5]. More global analyses (e.g. by the use of substractive hybridiation techniques, proteomics or expressed sequence tag (EST) approaches) have also been performed with different *Trichoderma *species, but have suffered from the fact that no full genome sequences of the used species were available for an in depth interpretation of the results. These studies mostly used a mix of different growth conditions to generate snapshots of the genetic arsenal of these fungi, rather than to study individual events during the mycoparasitic attack [7-11].

The analysis of EST transcripts represents an efficient means of characterizing the transcriptome of an organism, particularly when an annotated genome database is already available as in the present case. *Trichoderma atroviride *(teleomorph *Hypocrea atroviridis*) is one of the mycoparasitic *Trichoderma *spp., which has most often been used to investigate this process, but has not yet been used for a genomic analysis of mycoparasitism. Its 36.1 Mbp genome was recently completely sequenced, and contains 11.100 genes [12], which offered the opportunity to perform a systematic and comprehensive study of the transcriptional response to the presence of a host fungus. In addition, 40.000 ESTs were prepared from mycelia cultivated under mycoparasitic and non-mycoparasitic (mycelial growth, sporulation) conditions in order to aid in the annotation of its genome sequence.

The availability of these ESTs and an annotated genome thus raised the possibility to obtain a genome-wide insight into the transcriptomic response of *T. atroviride *under biocontrol-relevant conditions. We therefore used this EST collection to identify genes whose is expression in *T. atroviride *is modulated in relation to the presence of a fungal host, and thus arrive at a global picture of the molecular physiology of the fungus at the early stages of this intriguing process.

## Results

### Characterization of the EST database

We constructed EST libraries of *T. atroviride *from four different cultivation conditions: (a) confrontation with two plant pathogenic hosts (*Botrytis cinerea, Rhizoctonia solani*) on agar plates in the dark, using an mRNA extraction time point when their hyphae were only 1-2 mm apart; (b) a respective control consisting of only *T. atroviride *growing in the dark and not sporulating; (c) cultures illuminated by blue light to induce conidiation [13]; and (d) mycelia growing on plates in the dark and subjected to stress provoked by mechanical injury, a method known to lead to sporulation independent of light [13]. The rationale of this approach was that genes which would be uniquely expressed under (a) could so be distinguished from those required for filamentous growth and sporulation by *T. atroviride*.

After editing the mean length of the single read (see Materials and Methods), sequences ranged from 0.57 - 0.62 kb. A summary of the properties of the EST database used is given in Additional File 1. The representativeness of the libraries was confirmed by the high degree of transcript diversity (diversity index ranging from 60 to 75%). No ESTs representing rRNA or prokaryotic contaminations were found.

### Functional analysis of the EST libraries

Since an annotated genome sequence was available for this study, we developed the following approach for analysis of the ESTs: 27658 ESTs from all four libraries (cf. Additional File 1) were incorporated into a custom-made BLAST database, and the database then queried (BLASTX) with a total of 6889 genes from the *T. atroviride *genome database for which at least a putative function could be predicted and which were members of one of the 21 KOG groups (5 - 779 protein sequences/KOG group; Additional File 2). The respective protein models were extracted as FASTA files from the *T. atroviride *genome portal [12]. 9478 ESTs, showed positive matches. A comparison of the ESTs isolated under each of the four conditions illustrated that they were derived to approximately equal portions of ESTs from plate confrontations (2882), mycelial growth (2913), and light induced sporulation (3003). The number of hits from ESTs from plates subjected to mechanical injury were significantly lower (680), possibly due to the stress evoked by this growth conditions, which consequently had a negative impact on RNA quality, and they were thus excluded from most of the comparisons.

The 9478 ESTs represented 2734 genes, thus indicating that 39.7% of the queried genes were indeed detected in our EST collection. We examined to which of the 21 KOG classes the detected genes belonged and found that most KOG classes contained a similar proportion of expressed genes (29 ± 7%). Only three classes (J, translation; U, protein secretion; and Z, cytoskeleton) exhibited a strongly increased percentage of expressed genes (59, 53 and 51%, respectively). EST-density (the number of individual ESTs per average single identified gene) was highest in class J, too (9.1) but also significantly elevated in class C (energy metabolism; 4.2), indicating that these two classes may contain the most abundantly expressed genes (Additional File 2).

We also compared the distribution of the ESTs from the three different cultivation conditions in the 21 KOG groups. Since the EST collections for mycoparasitism, mycelial growth and light-induced sporulation comprise roughly similar numbers of ESTs, KOG groups that contain genes with similar expression patterns under all investigated growth conditions should be indicated by values close to 33.3% of ESTs for individual growth condition). However, values ranging from 17 - 50% were found (Table 1). When the ESTs from mycelial growth were taken as a reference growth condition, an increased number of ESTs for mycoparasitism was found in the KOG groups N (cell motility), O (posttranslational events), Z (cytoskeleton) and E (amino acid metabolism). The latter accounted for 46% of all ESTs assigned to this KOG group. Conversely, the light-induced, sporulating cultures exhibited enhanced EST numbers for the groups T (signalling), Z (cytoskeleton), K (transcription factors), D (cell cycle), C (energy metabolism) and L (DNA-repair) in comparison with mycelial growth.

**Table 1:** KOG-distribution of ESTs isolated from mycoparasitism (MP), mycelial growth (MG) and light induced sporulation (LC)

		MP	MG	LC	Total	Fraction
**Cellular processes and signalling**						
						
M	**M_cell wall membrane**	16	23	17	56	0,26
N	**N_cell motility**	6	3	3	12	0,5
O	**Posttranslational events**	464	355	328	1147	0,4
T	**Signalling**	170	194	215	579	0,29
Y	**nuclear structures**	19	35	28	82	0,23
U	**Secretion**	149	159	162	470	0,31
V	**V-defense**	34	42	62	138	0,24
W	**W-extracellular structures**	6	14	14	34	0,17
Z	**cytosceleton**	135	95	152	382	0,35
						
**Information storage and Processing**						
						
A	**RNA processing**	64	91	96	251	0,25
B	**chromatin dynamics**	76	72	77	225	0,33
J	**protein synthesis**	717	794	750	2261	0,32
K	**Transcription factors**	131	115	155	401	0,32
L	**repair**	41	32	50	123	0,33
						
**Metabolism**						
						
C	**energy metabolism**	255	252	273	780	0,33
D	**Cell cycle**	52	67	88	207	0,25
E	**Amino acid metabolism**	192	92	128	412	0,46
I	**Lipid metabolism**	106	130	113	349	0,3
F	**Nucleotide metabolism**	60	88	45	193	0,31
G	**Carbohydrate metabolism**	126	163	171	460	0,27
P	**Inorganic metabolism**	63	97	76	236	0,24
						
	Total	2882	2913	3003	8798	average 0,306

**Figure 1:**
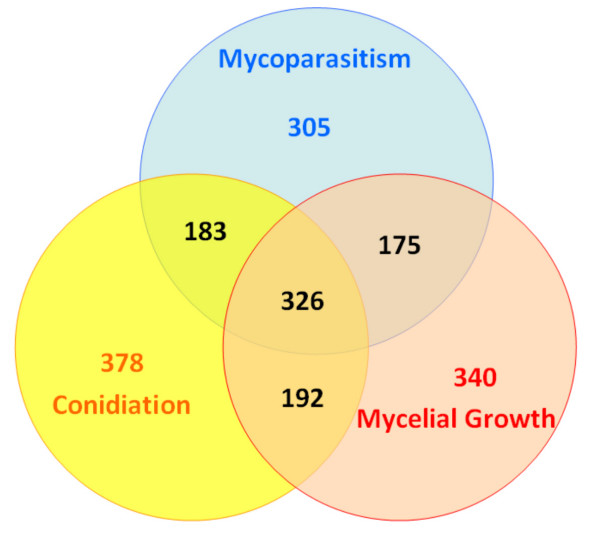
**VENN diagram of distribution and sharing of genes between onset of mycoparasitism, mycelial growth and light-induced conidiation**.

Of the total 2734 genes, for which ESTs were found, roughly 60% were found under only a single cultivation condition (Fig. 1). The remaining 1082 genes were retrieved from two or more conditions. Among these, 326 genes were found under all three conditions (mycoparasitism, mycelial growth and light-induced sporulation), and combinations of each two of these conditions were found for 175-192 genes, respectively. Combinations involving the genes expressed under mechanical injury also displayed roughly equal percentages but significantly lower numbers (35-41), which is however consistent with the other data when the lower number of total ESTs from this growth condition is taken into account. ESTs which were found under at least two conditions were particularly found in the KOG groups posttranslational modification, signal transduction, protein synthesis and energy metabolism (Table 2).

**Table 2:** Shared distribution of ESTs

	pairs						triplets				All	Total
MP	**x**	**x**	**x**				**x**	**x**		**x**	**x**	
MG	**x**			**x**	**x**		**x**	**x**	**x**		**x**	
LC		**x**		**x**		**x**	**x**		**x**	**x**	**x**	
IC			**x**		**x**	**x**		**x**	**x**	**x**	**x**	
												
**Cellular processes and signalling**												
cell walls and membranes	1			3	1	2	3					**10**
cell motility		1					1					**2**
posttranslational mod.	21	40		34	1		35	1	1	3	4	**140**
signal transduction	15	11	4	27	8	9	22	3	2	4	2	**107**
nuclear structure	3	4		3	3	2	1		2			**18**
secretion	20	18	5			1	22	3	1	1		**71**
defense		3	1	5			8		1	1		**19**
extracellular structures												**0**
cytosceleton	6	17			4	2	19				1	**49**
												
**Information storage and Processing**												
RNA processing	10	13		10	3	3	6			1		**46**
chromatin	4			5	2	1	7			1		**20**
protein synthesis	17	17	2	19			83	2	1	2	11	**154**
transcription factors	6	11	5	11	1	4	17			3	1	**59**
repair mechanisms	5	6	4			1						**16**
												
**Metabolism**												
energy metabolism	15	9	6	24	7	1	34	1	1	4	9	**111**
cell cycle	3	8	4	13	3	1	5	2	2	2		**43**
amino acid metabolism	14	16	3	8	3	2	15	1		1	2	**65**
lipid metabolism	9		1	6	1	1	10				3	**31**
nucleotide metabolism	8		1	5		1	8			1	1	**25**
carbohydrate metabolism	10	5	3	12	1	3	18	1	2	1	2	**58**
inorganic metabolism	8	4	2	7	1	1	12		2	1		**38**
												
	**99**	**76**	**31**	**110**	**19**	**16**	**209**	**7**	**8**	**16**	**29**	**620**

### Genes involved in sporulation are overexpressed under light-induced sporulation

As a prove of-principle that our dataset allows identification of genes specifically induced under a certain condition, we tested the expression of a set of 15 genes which are known to be involved in sporulation in *Neurospora crassa *[14], and for which orthologues were identified in *T. atroviride *(Kubicek et al.; ms in preparation). We found a total of 26 transcripts for 10 of them. These ESTs were distributed between mycoparasistism, mycelial growth, light-induced conidiation and mycelial injury, respectively, in a ratio of 4:4:14:4 (Additional File 3). When normalized to the total number of ESTs/growth condition, this shows that ESTs involved in sporulation (light-induced conidiation; mycelial injury) are ca. 3.5- and 4.3-fold overrepresented under the two conditions leading to sporulation, even though they were represented by different numbers of ESTs. This demonstrates that the number of ESTs in our sample correlates with their expression under the respective condition. The presence of an increased number of ESTs under one condition thus indicates an upregulation of the respective gene.

### Constitutively strongly expressed genes of *T. atroviride*

27 genes made up for 1034 of the 9478 ESTs found, and they were found in comparable numbers under the three major conditions (Additional File 4). The percentage of these strongly and constitutively expressed genes (10.8%) is slightly lower than reported for other *Trichoderma *EST datasets (15-17% for the 25 most abundantly expressed ESTs; [11,15]). Some of these genes encoded glycolytic enzymes, hydrophobins or histones that were also found in other studies. Some strongly expressed genes that we detected, however, were unexpected. Intriguingly, the most abundantly expressed gene was *cpc1*, which encodes the regulator of general amino acid control ("cross pathway control") in fungi [16]. Although its transcript abundance was much higher under conditions of mycoparasitism, its nevertheless significant abundance under the other conditions made us to refrain from calling it "mycoparasitism-specific" (see also below) but we note that it is strongly expressed under this condition.

### Genes overexpressed under mycoparasitic conditions

Our EST collection (2734 genes) contained only 28% of all genes present in the *T. atroviride *genome (11100 genes) and we therefore considered the presence of only single ESTs (albeit clearly proving the expression of the gene under this condition) to be insufficient for claiming specific induction in comparison to other conditions. To identify those genes from our EST collection that are significantly overexpressed under mycoparasitic conditions, we therefore looked for genes which were represented either by at least 3 ESTs under mycoparasitic conditions and by none under the others, or by 4 or more ESTs and thereby accounting for at least 65% of the total number of ESTs from the respective gene. Additional File 5 show s the results from this screening, grouped according to KOG terms: 442 ESTs, corresponding to 66 genes, were found to fulfil this criterion and thus be strongly overexpressed under mycoparasitic conditions. They were distributed over 18 KOG groups, but were most abundant in those comprising posttranslational processing (O) and amino acid metabolism (E): 159 of the total number of ESTs from the KOG group posttranslational processing (183) and 70 out of 159 ESTs assigned to amino acid metabolism belonged to the mycoparasitism-EST library. Conspicuously, several heat shock factors and tRNA synthases, belonging to the posttranslational processing group according to the KOG classification, were present in high numbers and may indicate a significant shift towards response to environmental stress (see Discussion). In addition, lipid metabolism also accounted for a significant portion of these EST (24 from 107). However, apart from these quantitative considerations, a number of genes (e.g. components of signal transduction, proteases) which comprised only a small portion of their KOG groups were also found and will be emphasized in the Discussion.

### RT-PCR confirms upregulation of a subset of genes whose ESTs are abundant during mycoparasitism

To test whether the EST abundance in fact reflects gene expression patterns under conditions of confrontation of *T. atroviride *with its prey, we performed reverse transcriptase PCR (RT-PCR) with 12 genes up-regulated during mycoparasitism and in addition with *cpc1*, which was according to our EST analysis abundant during mycoparasitism but also during mycelial growth. Two housekeeping genes served as a control (*gpd1, tef1*). To verify that an upregulation of the selected genes could be attributed to the presence of the host fungus irrespectively of the medium used, plate confrontation assays were set up on potato dextrose agar (PDA), whereas a minimal medium with 0.3% carbon source had been used for generation of the ESTs. Using *Rhizoctonia solani *as a host fungus, samples were taken before contact (5 mm distance of the mycelia), just at contact, and during overgrowth. It should be noted that for the ESTs mycelia were harvested at a growth stage that was between 'before contact' and 'contact' of this experiment. Expression patterns of the selected genes were monitored. Growth of *T. atroviride *alone on PDA plates, was used as control growth condition, corresponding to 'mycelial growth' in our EST collection.

**Figure 2:**
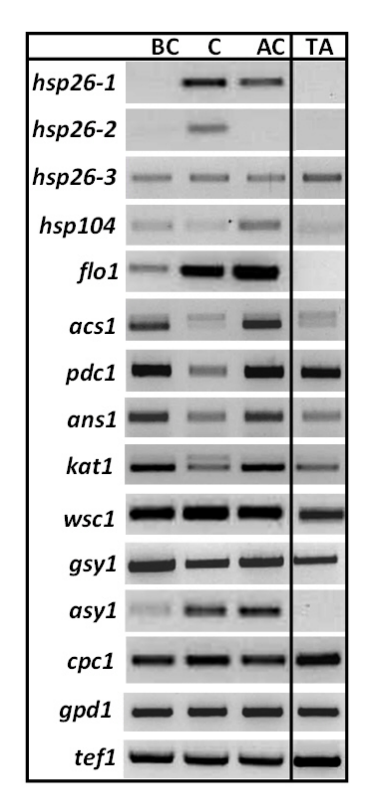
**Expression analysis of selected genes by RT-PCR**. Confrontations against *R. solani *on PDA plates covered by cellophane (before contact = BC, contact = C, after contact = AC); TA, *T. atroviride *growing alone on PDA plate. Gene abbreviations: *hsp26-1, hsp26-2, hsp26-3, hsp104 *heat shock proteins (146119, 146319, 160834 and 157453, respectively); *flo1 *unknown protein related to a *Candida *flocculent associated protein (135185); *acs1 *acyl-CoA synthetase (134354); *pdc1 *pyruvate decarboxylase (150078); *ans1 *anthranilate synthase component II (152602); *kat1 *kynurenine aminotransferase (159605); *wsc1*, WSC-domain containing extracellular protein (135366); *gsy1 *glutamyl-tRNA-synthase (146978); *asy1 *alanyl-tRNA synthase (153342); *cpc1*, cross pathway regulator CPC1 (132971); *gpd1*, glyceraldehyde-3-phosphate dehydrogenase (143663); *tef1*, elongation factor 1-alpha (146236).

In the gene expression analysis (Fig. 2), eight of the twelve putative mycoparasitismspecific genes showed a behaviour consistent with the EST data and were upregulated during mycoparasitism, whereas their transcripts were either absent or of much lower abundance in the control. The four other genes, however, also showed a high level of transcription in the control: *hsp26-3, wsc1, pdc *and *gsy1 *(although the latter was still more abundant at the onset of mycoparasitism), indicating that their upregulation is not mycoparasitism-specific. Transcripts of *cpc1 *were detected under all tested conditions. Therefore, our threshold for detection of mycoparasitism-specific genes appears to have mainly identified genes which are indeed actually upregulated during this process.

### Reconstruction and comparative analysis of a metabolic network

In order to obtain a global understanding of the metabolic changes of *T. atroviride *during the onset of mycoparasitism, we also used the genome sequence data to reconstruct a metabolic network and subsequently attempted to identify pathways that were differently expressed at the onset of mycoparasitism. Only enzymes with standardized EC numbers were thereby considered, which yielded 832 EC numbers in total. From these, a bipartite metabolic network containing 2013 reactions and 2198 metabolites was constructed. Subnetworks containing a portion of these reactions were then extracted from the whole network to address the aforementioned differentially expressed genes and KOG groups in a metabolic context.

The first intriguing finding - was that a significant number of amino acid biosynthetic pathways, especially those for common amino acids were significantly up-regulated (the both number of ESTs was more than two-fold higher than under other conditions). These pathways included (Additional File 6): urea cycle and metabolism of amino groups; glutamate metabolism; methionine metabolism; alanine and aspartate metabolism; valine, leucine and isoleucine biosynthesis; lysine biosynthesis; arginine and proline metabolism; cysteine metabolism. In addition, the biosynthesis of several aminoacyl-tRNAs was also enhanced. Amino acid catabolism (e.g. that of valine, leucine and isoleucine degradation), on the other hand, was obviously downregulated. These data are supportive of our data from EST evaluation and suggest that mycoparasitism is associated with a state of amino acids starvation. We also found an upregulation of pyridoxalphosphate biosynthesis which is consistent with an increased amino acid biosynthesis. Metabolism of sulphur containing compounds was also remarkably up-regulated, and it is important to note that its genes were not expressed under any of the other conditions. Analysis of the lipid metabolism showed that the degradation pathways of lipid metabolism were slightly up-regulated. In addition, biosynthesis of pantothenate was also increased, consistent with its role as a constituent of coenzyme A required for fatty acid catabolism. Another upregulation was found for genes involved in sphingolipid metabolism. Biosynthesis of unsaturated fatty acids pathway was absent. Also, aminosugar catabolism was significantly enhanced under mycoparasitic conditions, which would be in accordance of an attack of *T. atroviride *on the prey's cell wall.

## Discussion

We used an EST-based approach to investigate the global changes in the physiology of the mycoparasite *T. atroviride *when confronted with a potential fungal host. ESTs for approximately 900 genes were obtained from onset of mycoparasitism, and this number is roughly similar to that of ESTs found for the two other cultivation conditions (vegetative growth, light-induced conidiation), which were used for comparison. How complete is this analysis? In other fungi, genome wide expression analysis showed that, on the average, 55 - 60% of the genes present in the genome are transcribed under a given, investigated condition [17-19]. Of the 11.100 genes annotated in the genome of *T. atroviride*, we only used those for which a function could be predicted, which were 6889 genes. Therefore, if for 55 - 60% of these genes a transcript would be detected, this would have been 3789 - 4133 genes. The about 900 genes that were actually found under conditions of mycoparasitism, are thus only 13%. This number is much lower, and indicates that the number of ESTs collected represent only the fraction of more and most strongly expressed genes. Previous studies on the genome wide-expression of genes in other fungi arrived at a comparable figure of 400 - 450 transcripts (4-6% of all genes) that were at least 2-fold elevated under a specific condition (e.g. carbon catabolite repression in *S. cerevisiae *[20]; general amino acid control in *N. crassa*, [19]; or secretion stress response in *T. reesei*, [15]). Thus, our EST collection contains most likely only those genes which are highly (or at least medium-highly) expressed under the conditions used, and genes which are transcribed at a low level are probably absent.

Despite of these method-inherent limitations, the genes that we identified to be upregulated under conditions of confrontation with the prey reveal a clear trend in their KOG-distribution: 60% of all ESTs came from 3 KOG groups: posttranslational modification (O), amino acid metabolism (E) and lipid metabolism (I). In group O, 13 of the 17 genes encoded proteins of the heat shock/stress response such as HSP23, HSP70, HSP90 and HSP104. Moreover, even though these transcripts were among the most abundant ones detected in the mycoparasitic library, in several cases they were completely absent under the two other conditions. The function of these proteins in other organisms is intriguing: HSP90 is a molecular chaperone for many signal transducers and has been postulated to be involved in releasing previously silent genetic variation in response to environmental changes [21], such as drug resistance, and this resistance can be abrogated by HSP90 inhibitors [22]. HSP104 is an essential protein of the heat-shock response and belongs to the class 1 family of Clp/HSP100 AAA+ ATPases. Members of this family form large ring structures and have the ability to rescue proteins from an aggregated state, a process for which the assistance of the cognate HSP70 chaperone system is also needed [23]. The massive upregulation of all of these heat-shock proteins indicates that *T. atroviride *faces severe stress shortly before contact with its prey.

What type of stress could this be? One could envisage that *T. atroviride *has to respond to antifungal components formed by the host. We know that the preys used in this study are capable to form secondary metabolites [24-26], and although no information is so far available about competition and defense reactions of the hosts, it is possible that the above stress response reflects *T. atroviride's *attempts to combat against them. Based on an inspection of the metabolic network and an analysis of other strongly overexpressed transcripts, our data suggest an alternative interpretation: the upregulation of several genes of amino acid biosynthesis and of aminoacyl tRNA synthases would be consistent with the operation of the "cross pathway control" of amino acid biosynthesis. This control mechanism is induced in lower eukaryotes by starvation for any one of a number of metabolically sensible amino acids, and simultaneously leads to induced transcription followed by derepression of the enzymes in several of these amino acid's biosynthetic pathways [16]. The ultimate element and central transcription factor of the cross-pathway control is CPC1/GCN4, a member of the c-Jun-like transcriptional activator family [27]. It is intriguing that ESTs identified as the *T. atroviride cpc1 *orthologue were in total the most abundant transcripts in this study. Expression of *cpc1/cpcA *in *N. crassa *and *A. nidulans *is also subject to a transcriptional autoregulatory mechanism that causes a several-fold increase in its mRNA levels when cells are starved for amino acids [19,28]. The high, constitutive expression of *cpc1 *in this study has previously not been observed in any fungus, and suggests its involvement in other cellular regulatory processes than nitrogen shortage. As an example, in *S. cerevisiae *GCN4 is also involved in responses to purine starvation, glucose limitation, growth on ethanol, or high salinity (for review see [27]). *N. crassa *CPC1, on the other hand, also responds to the oxygen radicals and metal ions [19]. We should like to note that - supporting the operation of the cross pathway control during mycoparasitism - we also found ESTs for the *T. atroviride cpc3 *protein kinase orthologue (Triat1:130153) under this condition (data not shown).

An additional characteristic feature of the mycoparasitic EST-collection was its large number of genes for amino acyl-tRNA synthases. As explained above, cross pathway control is triggered by an accumulation of amino acid-free tRNAs. The strong upregulation of some of these genes may therefore suggest the operation of an autoregulatory response by which the cell attempts to compensate its deficiency of amino acyl-tRNAs. Elevated levels of some tRNA synthase genes have also been observed in *N. crassa *upon induction of cross pathway control by a 3-aminotriazol, although the effect may be indirect since most of their promoters do not contain the consensus site for CPC1 binding [19].

One could argue that the upregulation of amino acid biosynthesis and aminoacyl tRNA synthases may not be due to a nitrogen limitation signal, but simply reflect increased protein synthesis, which would be necessary for the increased synthesis of enzymes which are secreted during the onset of mycoparasitism and participate in resisting the attack on the prey [29]. We consider this explanation less likely for the following reasons: genes encoding such enzymes (chitinase, β-glucanases and proteases) were underrepresented in our EST library, and we detected the upregulation of only a few genes encoding major extracellular proteins or of the secretory pathway. The finding of increased aminosugar catabolism indicates that chitinases are probably formed, but that a low level of transcription is sufficient for the fungus at the stage of onset of mycoparasitism. A comparison with transcriptome data from other fungi under protein overexpressing and secreting conditions [15,30] implies that, if massive enzyme secretion would take place, we should have then seen the overexpression of some of the genes for components of the secretory pathway as well.

Therefore, we interpret our data such that *T. atroviride *is facing stress from nitrogen limitation when it is confronted with its prey. A similar interpretation was offered to explain that *A. fumigatus *Δ*cpcA *strains exhibited reduced virulence and viability *in vivo *in their mammalian host [31]. However, the upregulation of amino acid biosynthesis during the onset of mycoparasitism is unlikely to be due to a real limitation of nitrogen in the medium e.g. caused by the consumption of the nitrogen from the medium by the approaching antagonist. The genes *ans1 *and *kat1 *showed also a clear upregulation during mycoparasitism on PDA plates, as determined by RT-PCR. On this rich carbon source any starvation effects are unlikely to occur and upregulated genes are thus probably directly related to mycoparasitism and the presence of a prey. Our hypothesis is that the receptors, which sense the nitrogen status of the medium, are modulated by components derived from the host fungus and thereby pretend nitrogen limitation. In *S. cerevisiae*, the TOR kinase cascade regulates the cellular response to the nutrient status of the cell [32]. It is active under conditions of nitrogen sufficiency and is inactivated by nitrogen starvation conditions. In *G. fujikuroi*, Teichert et al. [33] found evidence for a regulation of TOR1 by glutamine synthase and an as yet-unknown nitrogen sensor. The STM1 seven transmembrane helix receptor of *Schizosaccharomyces pombe *[34] is such a sensor for amino acid starvation, and delivers its signal via a G-protein complex involving GNA2. *T. atroviride *has two paralogues of STM1 (STM1, Triat1:151018 and STM2, Triat1:133836). We found 3 ESTs of *stm1 *- but not of *stm2 *- exclusively under mycoparasitic conditions. A possible involvement of the STM1/GNA2 cascade in nitrogen signalling in *T. atroviride *would further be supported by the fact that knock out mutants of the *T. atroviride *orthologue of yeast *GNA2 *- *gna3/tga3 *- have been reported to be unable to parasitize *R. solani *or *B. cinerea *in plate confrontation assays [35]. The signal delivering nitrogen starvation to STM1 in *S. pombe *is not known yet, but Chung et al. [34] speculated that some nitrogenous metabolites may bind to it in a ligand-receptorspecific manner, thereby changing its structure and activating it. In nematophagous fungi, trapping of the prey has been shown to be induced by oligopeptides from the nematodes [36]. It is possible that a similar mechanism may also occur at the early stage of mycoparasitism by *T. atroviride*.

A further hint towards the operation of stress during the mycoparasitic attack comes from the finding that of some genes encoding proteins involved in signal transduction were also overexpressed. Although these genes are not members of the major upregulated KOG groups, they deserve comments as the signalling processes involved in recognition of the host by *Trichoderma *have recently been the subject of intense research [37-45]. In our analysis, particularly two genes were conspicuous: TMK3, encoding an orthologue of the HOG1 protein kinase required for appropriate reaction to cellular, especially osmotic stress, and whose inactivation in *T. harzianum *has already been reported to partially impair antagonism against its prey [37]. The other gene encodes a low-molecular weight protein tyrosine phosphate phosphatase (LMYP). Although its function is unknown, we should like to note that TMK3 is regulated by tyrosine phosphorylation and dephosphorylation [46]. The PTP2 protein, which dephosphorylates yeast TMK3 has not been studied in filamentous fungi yet. The *T. atroviride *genome contains a protein (Triat1:80848) with low similarity (e-34) to PTP2, but of which no ESTs were found in this study.

The mechanism of mycoparasitism has frequently been discussed in terms of involvement of extracellular enzymes capable of hydrolyzing the host's cell wall [29]. Interestingly, we found in our analysis only few of them to be upregulated during the onset of mycoparasitism. Two of them encoded proteases: an aspartyl protease (Triat1:129404), whose orthologue has already been isolated from *T. asperellum *and reported to be potentially involved in mycoparasitism [47]; and a subtilisin-like serine protease (Triat1:147394), which is an orthologue of *Metarhizium anisopliae *PR1C which is involved in insect cuticle degradation [48]. This suggests that proteases also play an important role in the first stages of mycoparasitism, and their expression would be consistent with a situation of nitrogen starvation. In support of this, Olmedo-Monfil et al. [49] reported that the induction of the *prb1 *gene, which encodes yet another protease of *T. atroviride *and whose overexpression enhances the mycoparasitic ability [50], depends on nitrogen limitation. The expression of proteolytic enzymes during the onset of mycoparasitism correlates with our above explained hypothesis of formation of peptides as a signal for nitrogen-deficiency.

During the final stages of mycoparasitism, *Trichoderma *also penetrates the host hyphae to utilize its cellular contents as nutrients [51]. Whether this process requires the formation of infection structures, like those known for plant pathogenic fungi, has been frequently postulated but not convincingly been shown or studied in detail yet. In any case, the penetration of host hyphae will most likely require mechanical pressure as well [51]. In the rice blast fungus *Magnaporthe grisea*, glycerol generated from storage lipids serves to build up the turgor needed for this pressure [52]. It is therefore intriguing that genes involved in lipid catabolism, notably fatty acid acyl CoA dehydrogenases, were strongly upregulated during mycoparasitism. The role of lipid degradation as a prerequisite for mycoparasitism has not yet been recognized and therefore needs further investigation. The simultaneous upregulation of the aquaporin Triat1:39327, a water channel, and *tmk3*, involved in signal transduction cascade of osmosensing, underlines a potential importance of osmoregulation at the onset of mycoparasitism.

## Conclusion

Genome-wide expression profiling of *T. atroviride *at the onset of mycoparasitism has revealed that the fungus undergoes major changes in gene expression which revealed previously unrecognized areas of importance to this process such as stress response, response to nitrogen shortage including cross pathway control, lipid metabolism and signalling. Our study therefore opens new opportunities (such as manipulation of the pathways mentioned above) for further research on the mechanism of mycoparasitism by reversed genetics and for development or selection of biocontrol agents. We also expect that future studies with whole-genomic oligonucleotide arrays, and the investigation of later steps of mycoparasitism in more detail will lead to a better understanding of this process.

## Methods

### Cultivation conditions

*T. atroviride *IMI206040 was grown on Vogel's minimal medium [53] with 0.3% (w/v) glucose as carbon source. For confrontation assays agar plates were overlaid with cellophane and inoculated with an agar plaques of the plant pathogen (*Rhizoctonia solani*, *Botrytis cinerea*), and *T. atroviride *in approximately 5 cm distance from the pathogen. Strains were grown in complete darkness at 28°C and *T. atroviride *was harvested under red safety light at pre-contact (1-2 mm distance of the mycelia) and immediately frozen in liquid nitrogen. For light induced conidiation, *T. atroviride *cultures were grown in the dark for 48 h at 27°C on PDA plates and used as pre-inoculum. Mycelial plugs (0.5 cm diam.) were taken from the colony growth front and placed on the centre of plates containing Vogel's medium covered with a cellophane membrane. Cultures were allowed to grow for further 36 h under these conditions, and then photoinduced as described in [54] by exposure to white light for 5 min (fluence rate 27 mmol m^-2 ^s^-1^). For injury-induced conidiation, fungal colonies were grown in total darkness on PDA at 27°C for 72 h, cut in stripes with a scalpel and incubated for an additional 24 h in the dark at 27°C. For mere mycelial growth, *T. atroviride *was grown on PDA plates in complete darkness at 28°C for 48 hrs.

For RT-PCR experiments strains were grown on PDA, covered with cellophane, in constant light at 25°C and harvested when the mycelia were ca. 5 mm apart (before contact), at contact of the mycelia and after *T. atroviride *had overgrown the host fungus by ca. 5 mm (after contact). As control *T. atroviride *was grown alone on plates and, in analogy to the growth conditions for the EST library, the peripheral hyphal zone was harvested.

### Nucleic acid isolation and manipulation

Mycelia were ground to a fine powder under liquid nitrogen and total RNA was isolated using the RNeasy kit (Qiagen). cDNA was synthesized using poly A+ RNA and oligo dT primers with 5'-Biotin-GGCGGCCGCACAACTTTGTACAAGAAAGTTGGGT-(T)19-3'. cDNAs were ligated to attB1 adapter (5'-TCGTCGGGGACAACTTTGTACAAAAAAGTTGG-3'), size fractionated using sephacryl S-500 HR columns, and recombined to pDONR222-Lib vector.

Mycelium for the control condition of the RT-PCR was directly extracted from agar. In this case the guanidinium thiocyanate buffer was substituted by a buffer containing 0.6 M NaCl, 10 mM EDTA, 4% SDS, 100 mM Tris, pH8) and after phenol extraction a precipitation step with 8 M LiCl was introduced. The resulting pellet was dissolved in 0.3 ml RNase-free water and subsequently precipitated again with isopropanol. All other steps were carried out identically to the guanidinium thiocyanate method [55].

### RT-PCR

For cDNA synthesis, RNA obtained from various cultivations was treated with DNase I (Fermentas, Burlington, Canada) and purified with the RNeasy MinElute Cleanup Kit (Qiagen, Valencia, CA, USA). 5 μg RNA/reaction were reverse transcribed using the the SuperScript™ III Reverse Transcriptase (Invitrogen, Carlsbad, CA, USA) and a mixture of random hexamer primer and oligo(dT)_18 _primer. For transcript analysis cDNA equivalent to 50 ng RNA/reaction, 25 cycles and the primers and annealing temperatures listed in Additional File 7 were used. PCR reactions were carried out in a total volume of 50 μl using the GoTaq system (Promega, Madison, WI, USA) according to the manufacturer's instructions. 40 μl of each PCR reaction were separated on a 1.5% agarose gel containing 0.5 μg/ml ethidium bromide. As negative controls, to ensure the absence of genomic DNA, templates for PCR were prepared by as described above, but no reverse transcriptase was added during the cDNA synthesis step.

### cDNA library sequencing

Bacterial colonies containing each *T. atroviride *cDNA library were plated onto agarose plates (254 mm plates from Teknova, Hollister, CA) at a density of approximately 1000 colonies per plate. Plates were grown at 37 C for 18 hours then individual colonies were picked and each used to inoculate a well containing LB media with appropriate antibiotic in a 384 well plate (Nunc, Rochester, NY). Clones were grown in 384 well plates at 37 C for 18 hours. Contained plasmid DNA for sequencing was produced by rolling circle amplification ([56] Templiphi, GE Healthcare, Piscataway, NJ). Inserts were sequenced from both ends using primers complimentary to the flanking vector sequence and Big Dye terminator chemistry then run on ABI 3730 instruments (ABI, Foster City, CA). The sequencing primers used were the following: for the mycelial injury library (vector: pDONR222; Fw: 5'-GTAAAACGACGGCCAGT, Rv: 5'-AGGAAACAGCTATGACCAT), for the light exposed library and the mycoparasitism library (vector: pEXP-AD502; Fw: 5' CTATTCGATGATGAAGATACC Rv: 5' AGAAGTCCAAAGCTCCACC), and for the dark exposed library (vector: pSPORT1; 5'-GTTTTCCCAGTCACGACGTTGTA, Rv: 5'-AGGAAACAGCTATGACCAT).

### EST sequence processing and assembly

ESTs were processed through the JGI EST pipeline (ESTs were generated in pairs, a 5' and 3' end read from each cDNA clone). To trim vector and adaptor sequences, common sequence patterns at the ends of ESTs (Expressed Sequence Tags) were identified and removed using an internally developed software tool. Insertless clones were identified if either of the following criteria were met: >200 bases of vector sequence at the 5' end or less than 100 bases of non-vector sequence remained. ESTs were then trimmed for quality using a sliding window trimmer (window = 11 bases). Once the average quality score in the window was below the threshold (Q15) the EST was split and the longest remaining sequence segment was retained as the trimmed EST. EST sequences with less than 100 bases of high quality sequence were removed. ESTs were evaluated for the presence of polyA or polyT tails (which if present were removed) and the EST re-evaluated for length, removing ESTs with less than 100 bases remaining. ESTs consisting of more than 50% low complexity sequence were also removed from the final set of "good ESTs". In the case of re-sequencing the same EST, the longest high quality EST was retained. Sister ESTs (end pair reads) were categorized as follows: if an EST lacked an insert or was a contaminant then by default the second sister was categorized as the same. However, each sister EST was treated separately for complexity and quality scores. Finally, EST sequences were compared to the GenBank/EMBL/DDBJ nucleotide database in order to identify contaminants; non-desirable ESTs such as those matching non-cellular and rRNA sequences were removed.

For clustering, ESTs were evaluated with MALIGN, a k-mer based alignment tool (Chapman, Unpublished), which clusters ESTs based on sequence overlap (k-mer = 16, seed length requirement = 32, alignment ID ≥ 98%). Clusters of ESTs were further merged based on sister ESTs using double linkage. Double linkage requires that 2 or more matching sister ESTs exist in both clusters to be merged. EST clusters were then each assembled using CAP3 [57] to form consensus sequences. Clusters may have more than one consensus sequence for various reasons to include; the clone has a long insert, clones are splice variants or consensus sequences are erroneously not assembled. Cluster singlets are clusters of one EST, whereas CAP3 singlets are single ESTs which had joined a cluster but during cluster assembly were isolated into a separate singlet consensus sequence. ESTs from each separate cDNA library were clustered and assembled separately and subsequently the entire set of ESTs for all cDNA libraries were clustered and assembled together.

A list of the ESTs and the corresponding accession numbers is given in Additional File 8.

### Functional analysis of the EST libraries

Since an annotated genome sequence of *T. atroviride *IMI206040 was available for this study [12], the following approach was taken for analysis of the 27658 ESTs: they were first used to construct a custom-BLAST database. This database was then queried (BLASTX) with the protein sequences extracted as FASTA files from individual KOG groups available at the *T. atroviride *genome portal [12]. To look for genes with special functions (e.g. CAZYs, receptors, proteins involved in sporulation etc.), these were manually retrieved from the database, combined to FASTA files and used for the query as above. All proteins contained in the same KOG group were manually verified. In the case of positive matches, the full length amino acid sequences of the respective proteins were further cross-checked by NCBI-BLAST or HMMER [58]. Significant hits were defined as those with 100% similarity between the amino acid sequences. Sequences exhibiting only a few aa mismatches (< 5%), which were speculated to represent sequencing errors, were manually verified by subjecting their ntsequences to TBLASTN against the *T. atroviride *genome sequence, and considered positive if no other protein showed the same similarity. The same strategy was used for ESTs, whose deduced aa-sequence was < 40 aa (and thus eventually representing a domain shared by several proteins), and identity was assumed when no other protein contained the same sequence. A complete list of identified genes is available in Additional File 9.

Proteins were considered to be orthologues by best bidirectional hits in BLASTP with a cutoff value of 1e-80. In cases of lower cutoff value, phylogenetic trees were constructed by NJ (MEGA 3.1; [59]), and an orthologue confirmed if the best bidirectional hit occurred in the same, strongly supported (bootstrap support > 75%) terminal clade.

### Metabolic network construction

We used the EC numbers and the updated KEGG reaction database [60] to build a bipartite metabolic network, which was constructed based on the connection matrix of reactions [61]. Compared to reaction graph or metabolite graph, wherein either reactions or metabolites (called "node") are shown in an interconnected way, the bipartite network is more understandable because, similar to the biochemistry textbook, both the reactions and metabolites are visualized at mean time. The transcriptional data can be easily mapped to the bipartite network by using the visualization attributes of the reaction nodes, representing the expression levels of corresponding genes. Some currency metabolites [60], such as ATP, NADH etc., easily involve in hundreds of biological reactions. In order to reduce the complexity of the network to make the real biological changes more visible, we snipped some connections of reactions mediated by the currency metabolites. The software Cytoscape [62,63] was used as a layout tool for the metabolic network. We mapped the ESTs to the whole network and its sub networks, by using the opacity of the background of reaction nodes to represent the level of expression which was normalized by the number of ESTs of the corresponding gene expressed in one condition divided by the total number of ESTs of this gene in all compared conditions. A minimal opacity of 50 is set to distinguish them with those not expressed. A series of sub networks, enabling a clearer view on the expression differences between mycoparasitism and mycelial growth, was extracted from the whole network according to the KEGG pathway category of the reactions.

## Authors' contributions

VS and SG performed the RT-PCR analyses and performed evaluation of the data, LFS and YS did the metabolic network analysis, EL sequenced and analysed the ESTs, AK produced the custom-made BLAST for this study, SZ analysed part of the data, MS, PM and AHE produced the respective cDNAs, IG and SEB supervised the *T. atroviride *genome sequencing effort, and CPK designed the study, analysed the data and - together with VS - wrote the paper. All authors approved the final version of the paper.

## Supplementary Material

Additional file 1 **Properties of the EST collection used for this work**. the table gives numbers of ESTs, clones, average reading length and variability of the EST collection. Click here for file

Additional file 2 **Gene fraction detected in the ESTs**. this table attributes ESTs to KOG categories, and provides information of the gene number and fraction that is expressed for each KOG category. Click here for file

Additional file 3 **Expression of sporulation specific genes in *T. atroviride***. this table lists EST numbers for selected genes known to be involved in conidiogenesis under conditions of mycoparasitism (MP), mycelial growth (MG), light induced conidiation (LI), and mechanical injury (IC). Click here for file

Additional file 4 **Most abundantly expressed genes**. this table lists EST numbers for the most abundantly expressed genes detected in this study. Abbreviation of conditions is as explained in Additional File S3. Click here for file

Additional file 5 **Genes significantly overexpressed under mycoparaistic conditions**. this table lists EST numbers for the most abundantly expressed genes detected under mycoparasitic conditions. Abbreviation of conditions is as explained in Additional File S3. Click here for file

Additional file 6 **Metabolic subnetworks of *T. atroviride *under mycoparasitic (A, C and E) and mycelial growth (B, D and F) conditions**. AB, CD, EF are pairs of contrasts to show the metabolic features under two conditions. AB show the subnetworks of common amino acid metabolism; CD show the subnetworks of sulfur amino acid related metabolic pathway; EF show the subnetworks of lipid metabolism and aminosugar catabolism. The rectangles and circles represent the enzymatic reactions and metabolites, respectively. The larger circles indicate the main metabolites, while the smaller circles show the currency metabolites such as ATP, NADH and etc. The links with arrows in one or two ends represent the irreversible or reversible reactions, respectively. The opacity of the rectangles indicates the strength of the expression of corresponding genes, which is normalized by the number of ESTs of a gene in one condition divided by the total number ESTs of this gene in both conditions. The darker the color, the stronger the expression. If no EST is present, there is no color and the background of the rectangle is transparent. Click here for file

Additional file 7 **Primers used for RT-PCR**. this table lists the primers used in this study, the corresponding protein ID of the respective gene and the annealing temperature. Click here for file

Additional file 8 **Genebank accession numbers of ESTs created and used in the work**. this table lists the ESTs, gene bank number, and number in the *T. atroviride *genome database. CCAH, mycoparasitic conditions; CBYT, mycelial growth; CBYP, light induced sporulation; CBWT, mechanical injury. Click here for file

Additional file 9 **Total list of genes identified in this study**. this table lists number of all ESTs under the four conditions (abbreviated as in Additional File 3) and their identification obtained during this study. Click here for file
