# Supplementary material for: Transcriptomic response of the mycoparasitic fungus Trichoderma atroviride to the presence of a fungal prey
Source: BMC Genomics. 2009 Nov 30;10:567. doi: 10.1186/1471-2164-10-567 (PMC2794292; doi:10.1186/1471-2164-10-567)
Supplement: Additional file 1 — Properties of the EST collection used for this work. the table gives numbers of ESTs, clones, average reading length and variability of the EST collection. [file 1471-2164-10-567-S1.PDF]

Additional File S1. Properties of the EST collection used for this work

Condition	**Mycoparasitism**	**Mycelial Growth**	**Light-induced Conidiation**	**Injury-provoked Conidiation**
Abbreviation	**MP**	**MG**	**LC**	**IC**
ESTs	7093	7658	7555	5352
Clones	4113	4175	4201	3043
Average read length (nt's)	574	624	627	584
Diversity [%] *	69	60	66	75

* Diversity was defined as the expression variance within a group of samples.
