# Supplementary material for: Transcriptomic response of the mycoparasitic fungus Trichoderma atroviride to the presence of a fungal prey
Source: BMC Genomics. 2009 Nov 30;10:567. doi: 10.1186/1471-2164-10-567 (PMC2794292; doi:10.1186/1471-2164-10-567)
Supplement: Additional file 2 — Gene fraction detected in the ESTs. this table attributes ESTs to KOG categories, and provides information of the gene number and fraction that is expressed for each KOG category. [file 1471-2164-10-567-S2.PDF]

Additional File S2. Gene fraction detected in the ESTs

	KOG Group	Total gene number	genes expressed	percentage	EST density*
**Cellular Processes and Signalling**					
M	**M_cell wall membrane**	155	23	0,14	2,6
N	**N_cell motility**	5	4	0,8	3
O	**Posttranslational events**	779	351	0,45	3,4
T	**Signalling**	650	272	0,39	2,47
Y	**nuclear structures**	147	57	0,39	1,91
U	**Secretion**	399	211	**0,53**	2,42
V	**V-defense**	162	43	0,26	3,35
W	**W-extracellular structures**	47	11	0,23	3,09
Z	**cytosceleton**	224	115	**0,51**	3,51
**Information storage and Processing**					
A	**RNA processing**	418	144	0,34	1,97
B	**chromatin dynamics**	192	73	0,38	3,41
J	**protein synthesis**	425	254	**0,59**	**9,12**
K	**Transcription factors**	487	181	0,37	2,52
L	**repair**	249	103	0,44	1,49
**Metabolism**					
C	**energy metabolism**	530	198	0,37	**4,23**
D	**Cell cycle**	245	104	0,42	2,23
E	**Amino acid metabolism**	413	152	0,36	2,99
I	**Lipid metabolism**	435	146	0,33	2,6
F	**Nucleotide metabolism**	133	57	0,42	3,59
G	**Carbohydrate metabolism**	549	146	0,27	3,36
P	**Inorganic metabolism**	245	89	0,36	3,02
