# Supplementary material for: Transcriptomic response of the mycoparasitic fungus Trichoderma atroviride to the presence of a fungal prey
Source: BMC Genomics. 2009 Nov 30;10:567. doi: 10.1186/1471-2164-10-567 (PMC2794292; doi:10.1186/1471-2164-10-567)
Supplement: Additional file 3 — Expression of sporulation specific genes in T. atroviride. this table lists EST numbers for selected genes known to be involved in conidiogenesis under conditions of mycoparasitism (MP), mycelial growth (MG), light induced conidiation (LI), and mechanical injury (IC). [file 1471-2164-10-567-S3.PDF]

Additional File S3. Expression of sporulation specific genes in *T. atroviride*

			MP	MG	LC	IC
Triat1:48009]	ADY2	Acetate transporter required for normal sporulation	1	1	6	
Triat1:49156]	DTR1	dityrosine transporter	1			1
Triat1:155842]	SMK1	Middle sporulation-specific mitogen-activated protein kinase	1	2		
Triat1:139084]	SPO14	Phospholipase D				1
Triat1:157019]	SPO20	Meiosis-specific subunit of the t-SNARE complex				1
Triat1:156465]	SPO75	Meiosis-specific protein, required for spore wall formation			1	
Triat1:157855]	SPR3	Sporulation-specific homolog of the yeast CDC3/10/11/12 family		1	1	
Triat1:152596]	GNA3	G-protein complex alpha subunit GpaA/FadA	1		4	
Triat1:151848]	STU1	cell pattern formation-associated protein			2	
Triat1:139169]	WET1	Developmental regulatory protein WetA				1
		Total:	4	4	**14**	4
