# Supplementary material for: Transcriptomic response of the mycoparasitic fungus Trichoderma atroviride to the presence of a fungal prey
Source: BMC Genomics. 2009 Nov 30;10:567. doi: 10.1186/1471-2164-10-567 (PMC2794292; doi:10.1186/1471-2164-10-567)
Supplement: Additional file 4 — Most abundantly expressed genes. this table lists EST numbers for the most abundantly expressed genes detected in this study. Abbreviation of conditions is as explained in Additional File S3. [file 1471-2164-10-567-S4.PDF]

Additional File S4. Most abundantly expressed genes

KOG ID	Protein ID	MP	MG	LC	IC	Total	KOG Description
KOG0837	132971	37	20	19		76	CPC1, regulator of general amino acid control
KOG0888	158745	19	36	14		69	Nucleoside diphosphate kinase
KOG0001	150957	30	20	13		63	Ubiquitin and ubiquitin-like proteins
KOG0749	135957	20	12	30		62	Mitochondrial ADP/ATP carrier proteins
- *	149654	18	18	23	1	60	hydrophobin HA_2b
KOG3855	143500	8	21	26	1	56	Monooxygenase involved in ubiquinone biosynthesis
KOG0003	47556	24	19	12		55	Ubiquitin/60s ribosomal protein L40 fusion
KOG3467	146680	20	19	12		51	Histone H4
KOG3301	78759	10	22	12	1	45	Ribosomal protein S4
KOG1242	85616	13	15	15	1	44	Protein containing adaptin N-terminal region
KOG0754	131412	18	7	17	1	43	Mitochondrial oxodicarboxylate carrier protein
KOG1728	94749	11	13	14	2	40	40S ribosomal protein S11
KOG0676	157685	3	17	18		38	Actin
KOG1950	129518		11	18	5	34	Glycosyl transferase, family 8 - glycogenin
KOG1745	158250	13	8	6		27	Histones H3 and H4
KOG0657	143663	9	8	9		26	Glyceraldehyde 3-phosphate dehydrogenase
KOG0009	146551	7	11	7		25	Ubiquitin-like/40S ribosomal S30 protein fusion
KOG0841	131742	10	9	5		24	Multifunctional chaperone (14-3-3 family)
KOG0841	159020	10	9	5		24	Multifunctional chaperone (14-3-3 family)
KOG3457	159353	10	9	5		24	Sec61 protein translocation complex, beta subunit
KOG2099	136507	2	11	10		23	Glycogen phosphorylase
KOG2670	157035	12	8	3		23	Enolase
KOG1744	150041	7	8	7		22	Histone H2B
KOG1756	150043	**3**	8	9		20	Histone 2A
KOG3344	49376	7	6	7		20	40s ribosomal protein s10
KOG0693	146335	6	10	4		20	Myo-inositol-1-phosphate synthase
KOG3957	146584	**1**	8	11		20	Predicted L-carnitine dehydratase/alpha-methylacyl-CoA racemase

* this protein does not have a KOG number
