# Supplementary material for: Transcriptomic response of the mycoparasitic fungus Trichoderma atroviride to the presence of a fungal prey
Source: BMC Genomics. 2009 Nov 30;10:567. doi: 10.1186/1471-2164-10-567 (PMC2794292; doi:10.1186/1471-2164-10-567)
Supplement: Additional file 5 — Genes significantly overexpressed under mycoparaistic conditions. this table lists EST numbers for the most abundantly expressed genes detected under mycoparasitic conditions. Abbreviation of conditions is as explained in Additional File S3. [file 1471-2164-10-567-S5.PDF]

Additional File S5. Genes significantly over-expressed under mycoparasitic conditions

	Protein ID	KOG ID	MP	MG	LC	IC	Total	Encoded Protein
Posttranslational	129404	KOG1339	6				6	Aspartyl protease
Processing	129493	KOG0019	23	4	3		30	Molecular chaperone (HSP90 family)
	132465	KOG0101	11	1	4		16	Molecular chaperones HSP70/HSC70, HSP70 superfamily
	137510	KOG3566	3				3	Glycosylphosphatidylinositol anchor attachment protein GAA1
	140907	KOG0742	6				6	AAA+-type ATPase
	142538	KOG0100	5		2		7	Molecular chaperones GRP78/BiP/KAR2, HSP70 superfamily
	146119	KOG0710	28				28	Molecular chaperone (small heat-shock protein Hsp26/Hsp42)
	146319	KOG0710	15				15	Molecular chaperone (small heat-shock protein Hsp26/Hsp42)
	147394	KOG4266	3				3	Serine protease
	149341	KOG0102	4		2		6	Molecular chaperones mortalin/PBP74/GRP75, HSP70 superfamily
	151287	KOG3158	7	3		1	11	HSP90 co-chaperone p23
	157172	KOG1051	3		1		4	Chaperone HSP104 and related ATP-dependent Clp proteases
	157453	KOG1051	12				12	Chaperone HSP104 and related ATP-dependent Clp proteases
	159436	KOG0548	9		1		10	Molecular co-chaperone STI1
	39107	KOG3158	7	2	1	1	11	HSP90 co-chaperone p23
	94401	KOG1651	3		1		4	Glutathione peroxidase
	95049	KOG0710	14		1		15	Molecular chaperone (small heat-shock protein Hsp26/Hsp42)
Transcription	136064	KOG1601	3		1	1	5	GATA-4/5/6 transcription factors
	153649	KOG0939	3				3	E3 ubiquitin-protein ligase/Putative upstream regulatory element binding protein, nsdD orthologue
Inorganic metabolism	135158	KOG3599	6	1	1		8	unknown, remotedly related to a flocculent-associated protein from Candida
	139416	KOG0209	3		1		4	P-type ATPase
Lipid metabolism	134354	KOG1176	6			1	7	Acyl-CoA synthetase

	142316	KOG3717	3	1		4	Carnitine O-acyltransferase CRAT
	135468	KOG1680	3			3	Enoyl-CoA hydratase
	160412	KOG1269	3			3	SAM-dependent methyltransferases
	91135	KOG3717	3	1		4	Carnitine O-acyltransferase CRAT
	94947	KOG1269	3			3	SAM-dependent methyltransferases
	146755	KOG2419	3			3	Phosphatidylserine decarboxylase
Amino acid metabolism	127449	KOG0454	3		1	4	3-isopropylmalate dehydratase
	129397	KOG4165	3		1	4	Gamma-glutamyl phosphate reductase
	140635	KOG0172	7	4		11	Lysine-ketoglutarate reductase/saccharopine dehydrogenase
	143079	KOG0053	3			3	Cystathionine beta-lyases/cystathionine gamma-synthase
	144243	KOG2448	4			4	Dihydroxy-acid dehydratase
	150078	KOG1184	16	1	1	18	Pyruvate decarboxylase
	151384	KOG2770	3			3	Aminomethyl transferase
	152602	KOG4201	7		1	8	Anthranilate synthase component II
	157321	KOG0975	4	1		5	Branched chain aminotransferase BCAT1, pyridoxal phosphate enzymes type IV superfamily
	159605	KOG0257	3	1		4	Kynurenine aminotransferase, glutamine transaminase K
	35395	KOG0786	3		1	4	3-isopropylmalate dehydrogenase
	42213	KOG2616	6	1	1	8	Pyridoxalphosphate-dependent enzyme/predicted threonine synthase
	90784	KOG1237	3			3	H+/oligopeptide symporter
	94783	KOG0257	5			5	Kynurenine aminotransferase, glutamine transaminase K
Nucleotide metabolism	127817	KOG3985	3	1	1		Methylthioadenosine phosphorylase MTAP
	133088	KOG2938	3				Predicted inosine-uridine preferring nucleoside hydrolase
Carbohydrate metabolism	39628	KOG2517	3			3	Ribulose kinase or related carbohydrate kinases
	50442	KOG2533	3	1	1	5	Permease of the major facilitator superfamily

Chromatin assembly	146642	KOG1507	7	2	2		11	Nucleosome assembly protein NAP-1
Signalling	146191	KOG0660	7		4	1	12	Mitogen-activated protein kinase
	146573	KOG3217	3			1	4	Protein tyrosine phosphatase
	152359	KOG1217	4				4	secreted unknown protein
Secretory pathway	146572	KOG3337	4				4	Protein similar to predicted member of the intramitochondrial sorting protein family
Nuclear transport	140190	KOG2992	3	1			4	Nucleolar GTPase/ATPase p130
Extracellular structures	135366	KOG1216	5	1	1		7	Secreted protein containing WSC domain
Cytosceleton	160239	KOG0677	3		1		4	Actin-related protein Arp2/3 complex, subunit Arp2
	82360	KOG0677	3		1		4	Actin-related protein Arp2/3 complex, subunit Arp2
RNA processing	36105	KOG 1908	4	1				Ribonuclease inhibitor type leucine-rich repeat proteins, unknown, intracellular
Energy metabolism	133076	KOG3855	6	1	1		8	Monooxygenase involved in coenzyme Q (ubiquinone) biosynthesis
	137869	KOG3025	9	2	2		13	Mitochondrial F1F0-ATP synthase, subunit c/ATP9/proteolipid
	143042	KOG2404	4	1			5	Fumarate reductase, flavoprotein subunit
	34158	KOG1563	3		1		4	Mitochondrial protein Surfeit 1/SURF1/SHY1, required for expression of cytochrome oxidase
	35034	KOG1563	3		1		4	Mitochondrial protein Surfeit 1/SURF1/SHY1, required for expression of cytochrome oxidase
Translation	146978	KOG1147	5	1			6	Glutamyl-tRNA synthetase
	153342	KOG0188	6	2			8	Alanyl-tRNA synthetase
	154464	KOG2314	3		1		4	Translation initiation factor 3, subunit b (eIF-3b)
	80258	KOG1779	3				3	40s ribosomal protein S27
	92814	KOG1195	3	1			4	Arginyl-tRNA synthetase
