# Supplementary figures and images for: Transcriptomic response of the mycoparasitic fungus Trichoderma atroviride to the presence of a fungal prey
Source: BMC Genomics. 2009 Nov 30;10:567. doi: 10.1186/1471-2164-10-567 (PMC2794292; doi:10.1186/1471-2164-10-567)

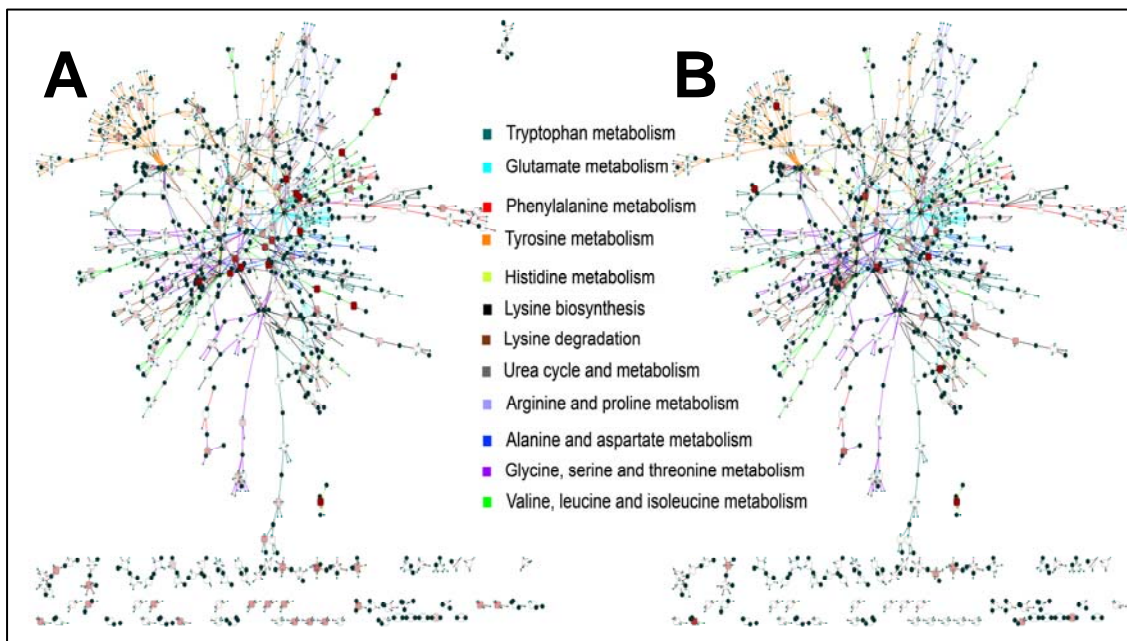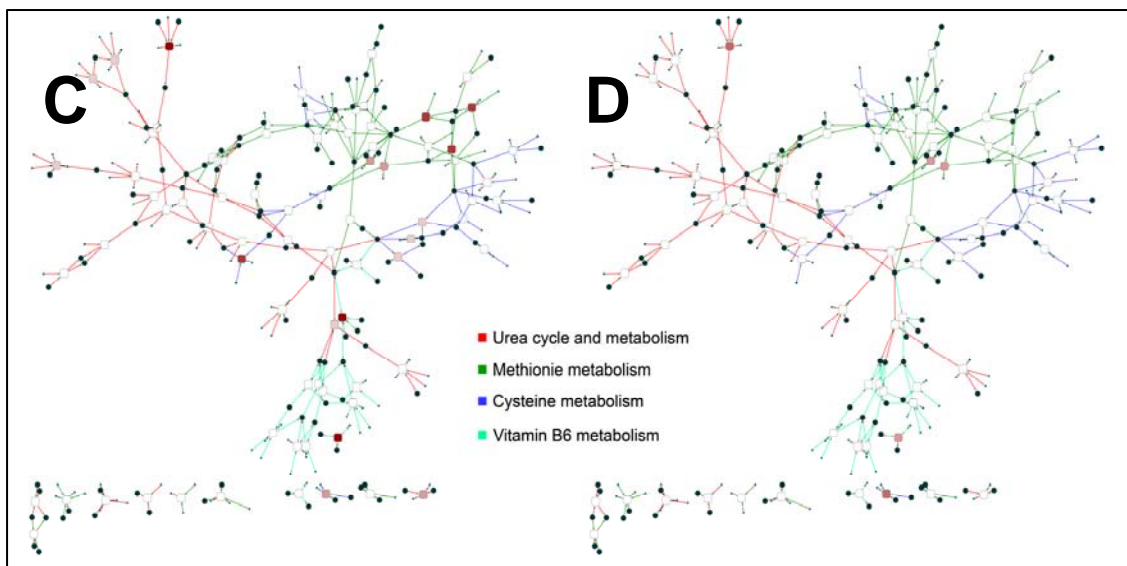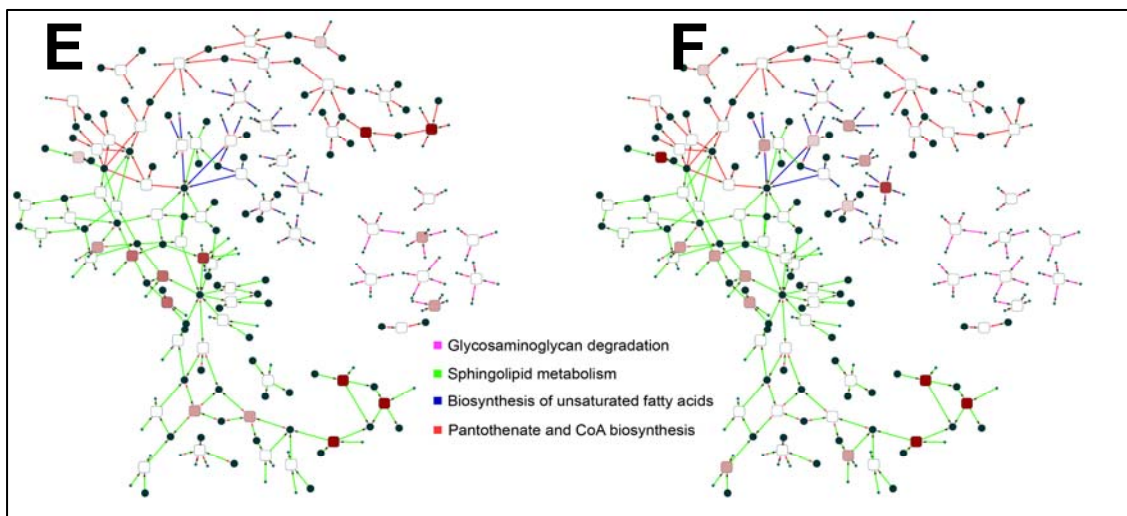

Supplement: Additional file 6 — Metabolic subnetworks of T. atroviride under mycoparasitic (A, C and E) and mycelial growth (B, D and F) conditions. AB, CD, EF are pairs of contrasts to show the metabolic features under two conditions. AB show the subnetworks of common amino acid metabolism; CD show the subnetworks of sulfur amino acid related metabolic pathway; EF show the subnetworks of lipid metabolism and aminosugar catabolism. The rectangles and circles represent the enzymatic reactions and metabolites, respectively. The larger circles indicate the main metabolites, while the smaller circles show the currency metabolites such as ATP, NADH and etc. The links with arrows in one or two ends represent the irreversible or reversible reactions, respectively. The opacity of the rectangles indicates the strength of the expression of corresponding genes, which is normalized by the number of ESTs of a gene in one condition divided by the total number ESTs of this gene in both conditions. The darker the color, the stronger the expression. If no EST is present, there is no color and the background of the rectangle is transparent. [file 1471-2164-10-567-S6.PDF]
