# Supplementary material for: Transcriptomic response of the mycoparasitic fungus Trichoderma atroviride to the presence of a fungal prey
Source: BMC Genomics. 2009 Nov 30;10:567. doi: 10.1186/1471-2164-10-567 (PMC2794292; doi:10.1186/1471-2164-10-567)
Supplement: Additional file 7 — Primers used for RT-PCR. this table lists the primers used in this study, the corresponding protein ID of the respective gene and the annealing temperature. [file 1471-2164-10-567-S7.PDF]

Additional Table S7. Primers used for RT-PCR.

Protein ID	Primer	Sequence 5'-3'	Annealing Temp. [°C]
143663	gpdh-RT-fw	CCAGAACATCATCCCCAGCAGC	63
	gdph-RT-rv	GATGGAAGAGTTGTTGTTGCCGAG	
146236	tef-fw	GGTACTGGTGAGTTCGAGGCTG	60
	tef-rv	GGGCTCAATGGCGTCAATG	
146119	hsp26-1-fw	CTCATCGCACTGTCGCACAC	60
	hsp26-1-rv	TGGCAGTAGTGGTTCCGTCTTC	
146319	hsp26-2-fw	TCTCTCACTCCTCTCTTCC	56
	hsp26-2-rv	TGAGGATCAGCGAACTCG	
157453	hsp104-fw	GAGCCCTGAAGAAGAGTC	56
	hsp104-rv	TCATGTCGATGGTGAATTTGG	
150078	cpc1-fw	CCGTGTCTCCTCAAGATC	56
	cpc1-rv	CTGATTCCAAAGGCGTCTG	
135158	flo1-fw	ACTGACAACTTCGGCTCCAC	60
	flo1-rv	AGTCATCAGTGCGACGAGTTC	
135336	wsc1-fw	AACAACATCCTCTCCGTTTG	56
	wsc1-rv	GCATTCGCTTCCAAACTCG	
153342	asy1-fw	GAGCTGTGGCAGAAGACTGG	60
	asy1-rv	TAGTTGGAGGCGGTATCCTG	
152602	ans1-fw	CACCCGCCTTGACTGCTTC	60
	ans1-rv	GCGTCAATGCCGTCCTCG	
134354	acs1-fw	CTGGAGTGAGACGGACGACG	61
	acs1-rv	CGACGGGTATTTCAAGACAGGA	
160834	hsp26-3-fw	CGACACGAGAAGGGAAGTTGAAG	60
	hsp26-3-rv	CCGACAAGATAATGGCACCG	
150078	pdc1-fw	GAGCCGTCACCAACAAAGAGG	62
	pdc1-rv	GCTCACCCGTCATTACACAGG	
146978	gsy1-fw	CGACTTTGCCTGCCCCG	60
	gsy1-rv	CGGCTGGGTCCTTGCTTG	
159605	kat1-fw	GAACAAAGTAGCCGCCCTCG	61
	kat1-rv	CACGGTTGGTTCTGCTGGC	
