# Supplementary material for: Transcriptomic response of the mycoparasitic fungus Trichoderma atroviride to the presence of a fungal prey
Source: BMC Genomics. 2009 Nov 30;10:567. doi: 10.1186/1471-2164-10-567 (PMC2794292; doi:10.1186/1471-2164-10-567)
Supplement: Additional file 8 — Genebank accession numbers of ESTs created and used in the work. this table lists the ESTs, gene bank number, and number in the T. atroviride genome database. CCAH, mycoparasitic conditions; CBYT, mycelial growth; CBYP, light induced sporulation; CBWT, mechanical injury. [file 1471-2164-10-567-S8.PDF]

Additional File S8.

Genebank accession numbers of ESTs created and used in the work.

dbEST_Id	User_Id	GenBank_Accn
=======	=======	============
61914398	CBWT1153.b1	GE270443
61914399	CBWT1153.g1	GE270444
61914400	CBWT1156.b1	GE270445
61914401	CBWT1158.b1	GE270446
61914402	CBWT1158.g1	GE270447
61914403	CBWT1159.b1	GE270448
61914404	CBWT1159.g1	GE270449
61914405	CBWT1160.b1	GE270450
61914406	CBWT1160.g1	GE270451
61914407	CBWT1161.b1	GE270452
61914408	CBWT1161.g1	GE270453
61914409	CBWT1162.b1	GE270454
61914410	CBWT1162.g1	GE270455
61914411	CBWT1166.b1	GE270456
61914412	CBWT1166.g1	GE270457
61914413	CBWT1167.b1	GE270458
61914414	CBWT1167.g1	GE270459
61914415	CBWT1170.b1	GE270460
61914416	CBWT1172.g1	GE270461
61914417	CBWT1177.b1	GE270462
61914418	CBWT1177.g1	GE270463
61914419	CBWT1178.b1	GE270464
61914420	CBWT1178.g1	GE270465
61914421	CBWT1183.b1	GE270466
61914422	CBWT1183.g1	GE270467
61914423	CBWT1185.b1	GE270468
61914424	CBWT1185.g1	GE270469
61914425	CBWT1186.b1	GE270470
61914426	CBWT1187.g1	GE270471
61914427	CBWT1195.b1	GE270472
61914428	CBWT1196.b1	GE270473
61914429	CBWT1196.g1	GE270474
61914430	CBWT1204.b1	GE270475
61914431	CBWT1205.b1	GE270476
61914432	CBWT1207.b1	GE270477
61914433	CBWT1208.b1	GE270478
61914434	CBWT1208.g1	GE270479
61914435	CBWT1210.b1	GE270480
61914436	CBWT1210.g1	GE270481
61914437	CBWT1211.g1	GE270482
61914438	CBWT1216.g1	GE270483
61914439	CBWT1218.b1	GE270484
61914440	CBWT1220.b1	GE270485
61914441	CBWT1221.b1	GE270486
61914442	CBWT1221.g1	GE270487
61914443	CBWT1222.g1	GE270488
61914444	CBWT1226.b1	GE270489
61914445	CBWT1226.g1	GE270490
61914446	CBWT1227.b1	GE270491
61914447	CBWT1227.g1	GE270492
61914448	CBWT1230.b1	GE270493
61914449	CBWT1230.g1	GE270494
61914450	CBWT1231.b1	GE270495

61914451	CBWT1231.g1	GE270496
61914452	CBWT1232.b1	GE270497
61914453	CBWT1232.g1	GE270498
61914454	CBWT1233.b1	GE270499
61914455	CBWT1233.g1	GE270500
61914456	CBWT1235.b1	GE270501
61914457	CBWT1235.g1	GE270502
61914458	CBWT1236.b1	GE270503
61914459	CBWT1237.b1	GE270504
61914460	CBWT1237.g1	GE270505
61914461	CBWT1238.b1	GE270506
61914462	CBWT1238.g1	GE270507
61914463	CBWT1239.b1	GE270508
61914464	CBWT1239.g1	GE270509
61914465	CBWT1242.b1	GE270510
61914466	CBWT1242.g1	GE270511
61914467	CBWT1244.b1	GE270512
61914468	CBWT1247.g1	GE270513
61914469	CBWT1249.b1	GE270514
61914470	CBWT1250.b1	GE270515
61914471	CBWT1250.g1	GE270516
61914472	CBWT1251.b1	GE270517
61914473	CBWT1251.g1	GE270518
61914474	CBWT1252.b1	GE270519
61914475	CBWT1252.g1	GE270520
61914476	CBWT1253.g1	GE270521
61914477	CBWT1255.b1	GE270522
61914478	CBWT1255.g1	GE270523
61914479	CBWT1256.b1	GE270524
61914480	CBWT1256.g1	GE270525
61914481	CBWT1257.b1	GE270526
61914482	CBWT1257.g1	GE270527
61914483	CBWT1258.b1	GE270528
61914484	CBWT1258.g1	GE270529
61914485	CBWT1259.b1	GE270530
61914486	CBWT1259.g1	GE270531
61914487	CBWT1261.b1	GE270532
61914488	CBWT1261.g1	GE270533
61914489	CBWT1262.b1	GE270534
61914490	CBWT1263.b1	GE270535
61914491	CBWT1263.g1	GE270536
61914492	CBWT1264.b1	GE270537
61914493	CBWT1264.g1	GE270538
61914494	CBWT1267.b1	GE270539
61914495	CBWT1267.g1	GE270540
61914496	CBWT1269.b1	GE270541
61914497	CBWT1269.g1	GE270542
61914498	CBWT1270.b1	GE270543
61914499	CBWT1270.g1	GE270544
61914500	CBWT1272.b1	GE270545
61914501	CBWT1272.g1	GE270546
61914502	CBWT1273.b1	GE270547
61914503	CBWT1273.g1	GE270548
61914504	CBWT1274.g1	GE270549
61914505	CBWT1275.b1	GE270550
61914506	CBWT1275.g1	GE270551
61914507	CBWT1276.g1	GE270552
61914508	CBWT1277.b1	GE270553
61914509	CBWT1277.g1	GE270554
61914510	CBWT1278.b1	GE270555
61914511	CBWT1278.g1	GE270556
61914512	CBWT1281.g1	GE270557
61914513	CBWT1288.b1	GE270558

61914514	CBWT1288.g1	GE270559
61914515	CBWT1289.b1	GE270560
61914516	CBWT1289.g1	GE270561
61914517	CBWT1291.b1	GE270562
61914518	CBWT1291.g1	GE270563
61914519	CBWT1292.b1	GE270564
61914520	CBWT1293.b1	GE270565
61914521	CBWT1293.g1	GE270566
61914522	CBWT1294.b1	GE270567
61914523	CBWT1294.g1	GE270568
61914524	CBWT1296.b1	GE270569
61914525	CBWT1296.g1	GE270570
61914526	CBWT1298.b1	GE270571
61914527	CBWT1298.g1	GE270572
61914528	CBWT1305.b1	GE270573
61914529	CBWT1305.g1	GE270574
61914530	CBWT1306.b1	GE270575
61914531	CBWT1306.g1	GE270576
61914532	CBWT1312.b1	GE270577
61914533	CBWT1312.g1	GE270578
61914534	CBWT1313.b1	GE270579
61914535	CBWT1313.g1	GE270580
61914536	CBWT1314.g1	GE270581
61914537	CBWT1315.b1	GE270582
61914538	CBWT1317.b1	GE270583
61914539	CBWT1319.b1	GE270584
61914540	CBWT1319.g1	GE270585
61914541	CBWT1321.b1	GE270586
61914542	CBWT1321.g1	GE270587
61914543	CBWT1323.b1	GE270588
61914544	CBWT1328.b1	GE270589
61914545	CBWT1328.g1	GE270590
61914546	CBWT1329.b1	GE270591
61914547	CBWT1329.g1	GE270592
61914548	CBWT1330.b1	GE270593
61914549	CBWT1330.g1	GE270594
61914550	CBWT1331.b1	GE270595
61914551	CBWT1331.g1	GE270596
61914552	CBWT1332.b1	GE270597
61914553	CBWT1332.g1	GE270598
61914554	CBWT1333.b1	GE270599
61914555	CBWT1335.b1	GE270600
61914556	CBWT1335.g1	GE270601
61914557	CBWT1336.b1	GE270602
61914558	CBWT1336.g1	GE270603
61914559	CBWT1338.b1	GE270604
61914560	CBWT1338.g1	GE270605
61914561	CBWT1339.b1	GE270606
61914562	CBWT1339.g1	GE270607
61914563	CBWT1340.b1	GE270608
61914564	CBWT1340.g1	GE270609
61914565	CBWT1341.b1	GE270610
61914566	CBWT1341.g1	GE270611
61914567	CBWT1342.b1	GE270612
61914568	CBWT1342.g1	GE270613
61914569	CBWT1343.b1	GE270614
61914570	CBWT1343.g1	GE270615
61914571	CBWT1344.b1	GE270616
61914572	CBWT1344.g1	GE270617
61914573	CBWT1345.b1	GE270618
61914574	CBWT1345.g1	GE270619
61914575	CBWT1346.b1	GE270620
61914576	CBWT1346.g1	GE270621

61914577	CBWT1347.b1	GE270622
61914578	CBWT1347.g1	GE270623
61914579	CBWT1348.b1	GE270624
61914580	CBWT1348.g1	GE270625
61914581	CBWT1349.b1	GE270626
61914582	CBWT1349.g1	GE270627
61914583	CBWT1350.b1	GE270628
61914584	CBWT1350.g1	GE270629
61914585	CBWT1351.b1	GE270630
61914586	CBWT1351.g1	GE270631
61914587	CBWT1357.b1	GE270632
61914588	CBWT1357.g1	GE270633
61914589	CBWT1358.b1	GE270634
61914590	CBWT1361.b1	GE270635
61914591	CBWT1361.g1	GE270636
61914592	CBWT1363.g1	GE270637
61914593	CBWT1364.b1	GE270638
61914594	CBWT1365.b1	GE270639
61914595	CBWT1365.g1	GE270640
61914596	CBWT1366.g1	GE270641
61914597	CBWT1368.b1	GE270642
61914598	CBWT1369.b1	GE270643
61914599	CBWT1369.g1	GE270644
61914600	CBWT1371.b1	GE270645
61914601	CBWT1371.g1	GE270646
61914602	CBWT1373.b1	GE270647
61914603	CBWT1373.g1	GE270648
61914604	CBWT1375.b1	GE270649
61914605	CBWT1375.g1	GE270650
61914606	CBWT1377.b1	GE270651
61914607	CBWT1377.g1	GE270652
61914608	CBWT1378.b1	GE270653
61914609	CBWT1378.g1	GE270654
61914610	CBWT1379.g1	GE270655
61914611	CBWT1384.b1	GE270656
61914612	CBWT1384.g1	GE270657
61914613	CBWT1385.b1	GE270658
61914614	CBWT1385.g1	GE270659
61914615	CBWT1390.g1	GE270660
61914616	CBWT1391.b1	GE270661
61914617	CBWT1391.g1	GE270662
61914618	CBWT1392.g1	GE270663
61914619	CBWT1393.b1	GE270664
61914620	CBWT1393.g1	GE270665
61914621	CBWT1394.b1	GE270666
61914622	CBWT1395.b1	GE270667
61914623	CBWT1396.b1	GE270668
61914624	CBWT1396.g1	GE270669
61914625	CBWT1398.g1	GE270670
61914626	CBWT1401.b1	GE270671
61914627	CBWT1401.g1	GE270672
61914628	CBWT1402.b1	GE270673
61914629	CBWT1405.b1	GE270674
61914630	CBWT1406.b1	GE270675
61914631	CBWT1406.g1	GE270676
61914632	CBWT1407.b1	GE270677
61914633	CBWT1408.b1	GE270678
61914634	CBWT1409.g1	GE270679
61914635	CBWT1413.b1	GE270680
61914636	CBWT1416.b1	GE270681
61914637	CBWT1416.g1	GE270682
61914638	CBWT1417.b1	GE270683
61914639	CBWT1417.g1	GE270684

61914640	CBWT1418.b1	GE270685
61914641	CBWT1418.g1	GE270686
61914642	CBWT1419.b1	GE270687
61914643	CBWT1419.g1	GE270688
61914644	CBWT1424.b1	GE270689
61914645	CBWT1425.b1	GE270690
61914646	CBWT1425.g1	GE270691
61914647	CBWT1426.b1	GE270692
61914648	CBWT1426.g1	GE270693
61914649	CBWT1427.b1	GE270694
61914650	CBWT1427.g1	GE270695
61914651	CBWT1428.b1	GE270696
61914652	CBWT1428.g1	GE270697
61914653	CBWT1429.b1	GE270698
61914654	CBWT1429.g1	GE270699
61914655	CBWT1430.b1	GE270700
61914656	CBWT1430.g1	GE270701
61914657	CBWT1432.b1	GE270702
61914658	CBWT1432.g1	GE270703
61914659	CBWT1435.b1	GE270704
61914660	CBWT1435.g1	GE270705
61914661	CBWT1436.b1	GE270706
61914662	CBWT1436.g1	GE270707
61914663	CBWT1438.b1	GE270708
61914664	CBWT1439.b1	GE270709
61914665	CBWT1439.g1	GE270710
61914666	CBWT1440.b1	GE270711
61914667	CBWT1440.g1	GE270712
61914668	CBWT1442.b1	GE270713
61914669	CBWT1442.g1	GE270714
61914670	CBWT1443.b1	GE270715
61914671	CBWT1443.g1	GE270716
61914672	CBWT1445.b1	GE270717
61914673	CBWT1445.g1	GE270718
61914674	CBWT1446.b1	GE270719
61914675	CBWT1446.g1	GE270720
61914676	CBWT1447.b1	GE270721
61914677	CBWT1447.g1	GE270722
61914678	CBWT1450.b1	GE270723
61914679	CBWT1452.b1	GE270724
61914680	CBWT1452.g1	GE270725
61914681	CBWT1453.b1	GE270726
61914682	CBWT1453.g1	GE270727
61914683	CBWT1455.b1	GE270728
61914684	CBWT1457.b1	GE270729
61914685	CBWT1457.g1	GE270730
61914686	CBWT1462.b1	GE270731
61914687	CBWT1462.g1	GE270732
61914688	CBWT1463.b1	GE270733
61914689	CBWT1463.g1	GE270734
61914690	CBWT1465.b1	GE270735
61914691	CBWT1465.g1	GE270736
61914692	CBWT1466.b1	GE270737
61914693	CBWT1466.g1	GE270738
61914694	CBWT1467.b1	GE270739
61914695	CBWT1467.g1	GE270740
61914696	CBWT1469.g1	GE270741
61914697	CBWT1472.b1	GE270742
61914698	CBWT1472.g1	GE270743
61914699	CBWT1473.b1	GE270744
61914700	CBWT1473.g1	GE270745
61914701	CBWT1474.b1	GE270746
61914702	CBWT1474.g1	GE270747

61914703	CBWT1478.b1	GE270748
61914704	CBWT1480.b1	GE270749
61914705	CBWT1480.g1	GE270750
61914706	CBWT1481.b1	GE270751
61914707	CBWT1481.g1	GE270752
61914708	CBWT1482.b1	GE270753
61914709	CBWT1482.g1	GE270754
61914710	CBWT1483.b1	GE270755
61914711	CBWT1483.g1	GE270756
61914712	CBWT1484.b1	GE270757
61914713	CBWT1484.g1	GE270758
61914714	CBWT1487.b1	GE270759
61914715	CBWT1487.g1	GE270760
61914716	CBWT1489.b1	GE270761
61914717	CBWT1489.g1	GE270762
61914718	CBWT1491.b1	GE270763
61914719	CBWT1493.g1	GE270764
61914720	CBWT1494.b1	GE270765
61914721	CBWT1495.b1	GE270766
61914722	CBWT1496.b1	GE270767
61914723	CBWT1496.g1	GE270768
61914724	CBWT1499.b1	GE270769
61914725	CBWT1499.g1	GE270770
61914726	CBWT1502.b1	GE270771
61914727	CBWT1502.g1	GE270772
61914728	CBWT1503.g1	GE270773
61914729	CBWT1504.b1	GE270774
61914730	CBWT1504.g1	GE270775
61914731	CBWT1510.g1	GE270776
61914732	CBWT1511.b1	GE270777
61914733	CBWT1511.g1	GE270778
61914734	CBWT1512.b1	GE270779
61914735	CBWT1512.g1	GE270780
61914736	CBWT1513.b1	GE270781
61914737	CBWT1513.g1	GE270782
61914738	CBWT1517.g1	GE270783
61914739	CBWT1518.b1	GE270784
61914740	CBWT1518.g1	GE270785
61914741	CBWT1520.g1	GE270786
61914742	CBWT1521.b1	GE270787
61914743	CBWT1521.g1	GE270788
61914744	CBWT1522.b1	GE270789
61914745	CBWT1522.g1	GE270790
61914746	CBWT1524.g1	GE270791
61914747	CBWT1526.b1	GE270792
61914748	CBWT1526.g1	GE270793
61914749	CBWT1527.b1	GE270794
61914750	CBWT1527.g1	GE270795
61914751	CBWT1528.b1	GE270796
61914752	CBWT1528.g1	GE270797
61914753	CBWT1530.b1	GE270798
61914754	CBWT1530.g1	GE270799
61914755	CBWT1531.b1	GE270800
61914756	CBWT1531.g1	GE270801
61914757	CBWT1532.b1	GE270802
61914758	CBWT1535.b1	GE270803
61914759	CBWT1535.g1	GE270804
61914760	CBWT1536.g1	GE270805
61914761	CBWT1540.b1	GE270806
61914762	CBWT1540.g1	GE270807
61914763	CBWT1541.b1	GE270808
61914764	CBWT1541.g1	GE270809
61914765	CBWT1542.b1	GE270810

61914766	CBWT1542.g1	GE270811
61914767	CBWT1543.b1	GE270812
61914768	CBWT1543.g1	GE270813
61914769	CBWT1544.g1	GE270814
61914770	CBWT1546.b1	GE270815
61914771	CBWT1546.g1	GE270816
61914772	CBWT1549.b1	GE270817
61914773	CBWT1549.g1	GE270818
61914774	CBWT1550.b1	GE270819
61914775	CBWT1550.g1	GE270820
61914776	CBWT1551.b1	GE270821
61914777	CBWT1551.g1	GE270822
61914778	CBWT1553.b1	GE270823
61914779	CBWT1553.g1	GE270824
61914780	CBWT1554.b1	GE270825
61914781	CBWT1554.g1	GE270826
61914782	CBWT1555.g1	GE270827
61914783	CBWT1556.b1	GE270828
61914784	CBWT1557.g1	GE270829
61914785	CBWT1558.b1	GE270830
61914786	CBWT1559.b1	GE270831
61914787	CBWT1559.g1	GE270832
61914788	CBWT1560.b1	GE270833
61914789	CBWT1560.g1	GE270834
61914790	CBWT1562.b1	GE270835
61914791	CBWT1562.g1	GE270836
61914792	CBWT1563.b1	GE270837
61914793	CBWT1563.g1	GE270838
61914794	CBWT1564.g1	GE270839
61914795	CBWT1570.b1	GE270840
61914796	CBWT1570.g1	GE270841
61914797	CBWT1571.b1	GE270842
61914798	CBWT1571.g1	GE270843
61914799	CBWT1574.b1	GE270844
61914800	CBWT1574.g1	GE270845
61914801	CBWT1575.b1	GE270846
61914802	CBWT1577.b1	GE270847
61914803	CBWT1577.g1	GE270848
61914804	CBWT1579.b1	GE270849
61914805	CBWT1579.g1	GE270850
61914806	CBWT1581.b1	GE270851
61914807	CBWT1581.g1	GE270852
61914808	CBWT1582.b1	GE270853
61914809	CBWT1582.g1	GE270854
61914810	CBWT1583.b1	GE270855
61914811	CBWT1583.g1	GE270856
61914812	CBWT1584.b1	GE270857
61914813	CBWT1584.g1	GE270858
61914814	CBWT1585.b1	GE270859
61914815	CBWT1585.g1	GE270860
61914816	CBWT1586.b1	GE270861
61914817	CBWT1586.g1	GE270862
61914818	CBWT1588.g1	GE270863
61914819	CBWT1589.b1	GE270864
61914820	CBWT1589.g1	GE270865
61914821	CBWT1590.b1	GE270866
61914822	CBWT1590.g1	GE270867
61914823	CBWT1591.b1	GE270868
61914824	CBWT1591.g1	GE270869
61914825	CBWT1592.b1	GE270870
61914826	CBWT1592.g1	GE270871
61914827	CBWT1593.b1	GE270872
61914828	CBWT1593.g1	GE270873

61914829	CBWT1594.g1	GE270874
61914830	CBWT1595.g1	GE270875
61914831	CBWT1597.b1	GE270876
61914832	CBWT1597.g1	GE270877
61914833	CBWT1599.b1	GE270878
61914834	CBWT1599.g1	GE270879
61914835	CBWT1600.b1	GE270880
61914836	CBWT1600.g1	GE270881
61914837	CBWT1601.g1	GE270882
61914838	CBWT1602.g1	GE270883
61914839	CBWT1603.g1	GE270884
61914840	CBWT1604.b1	GE270885
61914841	CBWT1604.g1	GE270886
61914842	CBWT1605.b1	GE270887
61914843	CBWT1605.g1	GE270888
61914844	CBWT1606.b1	GE270889
61914845	CBWT1606.g1	GE270890
61914846	CBWT1607.g1	GE270891
61914847	CBWT1608.b1	GE270892
61914848	CBWT1608.g1	GE270893
61914849	CBWT1609.b1	GE270894
61914850	CBWT1609.g1	GE270895
61914851	CBWT1612.g1	GE270896
61914852	CBWT1615.b1	GE270897
61914853	CBWT1615.g1	GE270898
61914854	CBWT1616.b1	GE270899
61914855	CBWT1617.b1	GE270900
61914856	CBWT1617.g1	GE270901
61914857	CBWT1618.b1	GE270902
61914858	CBWT1618.g1	GE270903
61914859	CBWT1621.b1	GE270904
61914860	CBWT1621.g1	GE270905
61914861	CBWT1622.g1	GE270906
61914862	CBWT1623.b1	GE270907
61914863	CBWT1623.g1	GE270908
61914864	CBWT1626.b1	GE270909
61914865	CBWT1629.b1	GE270910
61914866	CBWT1629.g1	GE270911
61914867	CBWT1630.b1	GE270912
61914868	CBWT1631.b1	GE270913
61914869	CBWT1631.g1	GE270914
61914870	CBWT1632.b1	GE270915
61914871	CBWT1633.b1	GE270916
61914872	CBWT1633.g1	GE270917
61914873	CBWT1634.g1	GE270918
61914874	CBWT1635.b1	GE270919
61914875	CBWT1635.g1	GE270920
61914876	CBWT1636.b1	GE270921
61914877	CBWT1636.g1	GE270922
61914878	CBWT1637.b1	GE270923
61914879	CBWT1637.g1	GE270924
61914880	CBWT1638.b1	GE270925
61914881	CBWT1638.g1	GE270926
61914882	CBWT1641.b1	GE270927
61914883	CBWT1643.b1	GE270928
61914884	CBWT1643.g1	GE270929
61914885	CBWT1644.b1	GE270930
61914886	CBWT1644.g1	GE270931
61914887	CBWT1645.b1	GE270932
61914888	CBWT1645.g1	GE270933
61914889	CBWT1646.b1	GE270934
61914890	CBWT1646.g1	GE270935
61914891	CBWT1647.b1	GE270936

61914892	CBWT1647.g1	GE270937
61914893	CBWT1648.b1	GE270938
61914894	CBWT1648.g1	GE270939
61914895	CBWT1649.b1	GE270940
61914896	CBWT1649.g1	GE270941
61914897	CBWT1650.b1	GE270942
61914898	CBWT1650.g1	GE270943
61914899	CBWT1652.g1	GE270944
61914900	CBWT1653.b1	GE270945
61914901	CBWT1653.g1	GE270946
61914902	CBWT1655.b1	GE270947
61914903	CBWT1655.g1	GE270948
61914904	CBWT1656.b1	GE270949
61914905	CBWT1657.b1	GE270950
61914906	CBWT1657.g1	GE270951
61914907	CBWT1658.b1	GE270952
61914908	CBWT1658.g1	GE270953
61914909	CBWT1659.b1	GE270954
61914910	CBWT1659.g1	GE270955
61914911	CBWT1660.g1	GE270956
61914912	CBWT1662.b1	GE270957
61914913	CBWT1662.g1	GE270958
61914914	CBWT1664.b1	GE270959
61914915	CBWT1664.g1	GE270960
61914916	CBWT1665.b1	GE270961
61914917	CBWT1670.b1	GE270962
61914918	CBWT1672.b1	GE270963
61914919	CBWT1672.g1	GE270964
61914920	CBWT1673.b1	GE270965
61914921	CBWT1673.g1	GE270966
61914922	CBWT1674.b1	GE270967
61914923	CBWT1674.g1	GE270968
61914924	CBWT1675.b1	GE270969
61914925	CBWT1675.g1	GE270970
61914926	CBWT1677.b1	GE270971
61914927	CBWT1677.g1	GE270972
61914928	CBWT1678.g1	GE270973
61914929	CBWT1679.b1	GE270974
61914930	CBWT1679.g1	GE270975
61914931	CBWT1680.b1	GE270976
61914932	CBWT1681.b1	GE270977
61914933	CBWT1681.g1	GE270978
61914934	CBWT1687.b1	GE270979
61914935	CBWT1687.g1	GE270980
61914936	CBWT1688.g1	GE270981
61914937	CBWT1689.b1	GE270982
61914938	CBWT1689.g1	GE270983
61914939	CBWT1690.b1	GE270984
61914940	CBWT1690.g1	GE270985
61914941	CBWT1693.b1	GE270986
61914942	CBWT1694.b1	GE270987
61914943	CBWT1694.g1	GE270988
61914944	CBWT1695.b1	GE270989
61914945	CBWT1695.g1	GE270990
61914946	CBWT1697.g1	GE270991
61914947	CBWT1698.b1	GE270992
61914948	CBWT1699.b1	GE270993
61914949	CBWT1699.g1	GE270994
61914950	CBWT1700.b1	GE270995
61914951	CBWT1700.g1	GE270996
61914952	CBWT1701.b1	GE270997
61914953	CBWT1701.g1	GE270998
61914954	CBWT1703.b1	GE270999

61914955	CBWT1703.g1	GE271000
61914956	CBWT1705.b1	GE271001
61914957	CBWT1705.g1	GE271002
61914958	CBWT1706.b1	GE271003
61914959	CBWT1706.g1	GE271004
61914960	CBWT1709.b1	GE271005
61914961	CBWT1709.g1	GE271006
61914962	CBWT1710.b1	GE271007
61914963	CBWT1710.g1	GE271008
61914964	CBWT1711.b1	GE271009
61914965	CBWT1711.g1	GE271010
61914966	CBWT1712.b1	GE271011
61914967	CBWT1712.g1	GE271012
61914968	CBWT1713.b1	GE271013
61914969	CBWT1713.g1	GE271014
61914970	CBWT1714.b1	GE271015
61914971	CBWT1715.b1	GE271016
61914972	CBWT1715.g1	GE271017
61914973	CBWT1716.b1	GE271018
61914974	CBWT1718.b1	GE271019
61914975	CBWT1718.g1	GE271020
61914976	CBWT1719.b1	GE271021
61914977	CBWT1719.g1	GE271022
61914978	CBWT1720.b1	GE271023
61914979	CBWT1720.g1	GE271024
61914980	CBWT1721.b1	GE271025
61914981	CBWT1722.b1	GE271026
61914982	CBWT1722.g1	GE271027
61914983	CBWT1723.b1	GE271028
61914984	CBWT1723.g1	GE271029
61914985	CBWT1724.b1	GE271030
61914986	CBWT1724.g1	GE271031
61914987	CBWT1725.b1	GE271032
61914988	CBWT1725.g1	GE271033
61914989	CBWT1727.b1	GE271034
61914990	CBWT1727.g1	GE271035
61914991	CBWT1728.b1	GE271036
61914992	CBWT1728.g1	GE271037
61914993	CBWT1729.b1	GE271038
61914994	CBWT1730.b1	GE271039
61914995	CBWT1730.g1	GE271040
61914996	CBWT1731.b1	GE271041
61914997	CBWT1731.g1	GE271042
61914998	CBWT1732.g1	GE271043
61914999	CBWT1733.b1	GE271044
61915000	CBWT1733.g1	GE271045
61915001	CBWT1734.b1	GE271046
61915002	CBWT1734.g1	GE271047
61915003	CBWT1735.b1	GE271048
61915004	CBWT1735.g1	GE271049
61915005	CBWT1739.b1	GE271050
61915006	CBWT1739.g1	GE271051
61915007	CBWT1740.b1	GE271052
61915008	CBWT1743.b1	GE271053
61915009	CBWT1743.g1	GE271054
61915010	CBWT1746.b1	GE271055
61915011	CBWT1746.g1	GE271056
61915012	CBWT1748.b1	GE271057
61915013	CBWT1749.b1	GE271058
61915014	CBWT1749.g1	GE271059
61915015	CBWT1750.b1	GE271060
61915016	CBWT1750.g1	GE271061
61915017	CBWT1751.b1	GE271062

61915018	CBWT1751.g1	GE271063
61915019	CBWT1752.b1	GE271064
61915020	CBWT1752.g1	GE271065
61915021	CBWT1753.b1	GE271066
61915022	CBWT1754.b1	GE271067
61915023	CBWT1754.g1	GE271068
61915024	CBWT1755.g1	GE271069
61915025	CBWT1756.b1	GE271070
61915026	CBWT1760.b1	GE271071
61915027	CBWT1760.g1	GE271072
61915028	CBWT1761.b1	GE271073
61915029	CBWT1761.g1	GE271074
61915030	CBWT1763.g1	GE271075
61915031	CBWT1764.b1	GE271076
61915032	CBWT1764.g1	GE271077
61915033	CBWT1766.b1	GE271078
61915034	CBWT1766.g1	GE271079
61915035	CBWT1767.b1	GE271080
61915036	CBWT1767.g1	GE271081
61915037	CBWT1768.b1	GE271082
61915038	CBWT1768.g1	GE271083
61915039	CBWT1770.b1	GE271084
61915040	CBWT1770.g1	GE271085
61915041	CBWT1772.g1	GE271086
61915042	CBWT1773.b1	GE271087
61915043	CBWT1773.g1	GE271088
61915044	CBWT1774.b1	GE271089
61915045	CBWT1774.g1	GE271090
61915046	CBWT1775.b1	GE271091
61915047	CBWT1775.g1	GE271092
61915048	CBWT1776.b1	GE271093
61915049	CBWT1776.g1	GE271094
61915050	CBWT1778.b1	GE271095
61915051	CBWT1778.g1	GE271096
61915052	CBWT1780.b1	GE271097
61915053	CBWT1782.b1	GE271098
61915054	CBWT1782.g1	GE271099
61915055	CBWT1784.b1	GE271100
61915056	CBWT1785.b1	GE271101
61915057	CBWT1785.g1	GE271102
61915058	CBWT1789.g1	GE271103
61915059	CBWT1790.b1	GE271104
61915060	CBWT1790.g1	GE271105
61915061	CBWT1791.g1	GE271106
61915062	CBWT1792.b1	GE271107
61915063	CBWT1792.g1	GE271108
61915064	CBWT1793.b1	GE271109
61915065	CBWT1793.g1	GE271110
61915066	CBWT1794.b1	GE271111
61915067	CBWT1794.g1	GE271112
61915068	CBWT1795.b1	GE271113
61915069	CBWT1795.g1	GE271114
61915070	CBWT1796.g1	GE271115
61915071	CBWT1797.b1	GE271116
61915072	CBWT1797.g1	GE271117
61915073	CBWT1798.b1	GE271118
61915074	CBWT1798.g1	GE271119
61915075	CBWT1800.b1	GE271120
61915076	CBWT1800.g1	GE271121
61915077	CBWT1801.b1	GE271122
61915078	CBWT1801.g1	GE271123
61915079	CBWT1802.b1	GE271124
61915080	CBWT1802.g1	GE271125

61915081	CBWT1803.b1	GE271126
61915082	CBWT1803.g1	GE271127
61915083	CBWT1804.b1	GE271128
61915084	CBWT1804.g1	GE271129
61915085	CBWT1805.b1	GE271130
61915086	CBWT1805.g1	GE271131
61915087	CBWT1807.b1	GE271132
61915088	CBWT1808.b1	GE271133
61915089	CBWT1810.b1	GE271134
61915090	CBWT1810.g1	GE271135
61915091	CBWT1812.b1	GE271136
61915092	CBWT1812.g1	GE271137
61915093	CBWT1813.b1	GE271138
61915094	CBWT1813.g1	GE271139
61915095	CBWT1814.b1	GE271140
61915096	CBWT1814.g1	GE271141
61915097	CBWT1815.b1	GE271142
61915098	CBWT1815.g1	GE271143
61915099	CBWT1819.b1	GE271144
61915100	CBWT1819.g1	GE271145
61915101	CBWT1821.b1	GE271146
61915102	CBWT1821.g1	GE271147
61915103	CBWT1823.b1	GE271148
61915104	CBWT1823.g1	GE271149
61915105	CBWT1825.b1	GE271150
61915106	CBWT1827.b1	GE271151
61915107	CBWT1827.g1	GE271152
61915108	CBWT1829.b1	GE271153
61915109	CBWT1829.g1	GE271154
61915110	CBWT1831.b1	GE271155
61915111	CBWT1831.g1	GE271156
61915112	CBWT1832.b1	GE271157
61915113	CBWT1832.g1	GE271158
61915114	CBWT1834.b1	GE271159
61915115	CBWT1834.g1	GE271160
61915116	CBWT1835.b1	GE271161
61915117	CBWT1835.g1	GE271162
61915118	CBWT1836.b1	GE271163
61915119	CBWT1836.g1	GE271164
61915120	CBWT1837.b1	GE271165
61915121	CBWT1838.b1	GE271166
61915122	CBWT1838.g1	GE271167
61915123	CBWT1839.b1	GE271168
61915124	CBWT1839.g1	GE271169
61915125	CBWT1840.b1	GE271170
61915126	CBWT1840.g1	GE271171
61915127	CBWT1842.b1	GE271172
61915128	CBWT1842.g1	GE271173
61915129	CBWT1843.b1	GE271174
61915130	CBWT1843.g1	GE271175
61915131	CBWT1844.g1	GE271176
61915132	CBWT1847.b1	GE271177
61915133	CBWT1847.g1	GE271178
61915134	CBWT1848.b1	GE271179
61915135	CBWT1848.g1	GE271180
61915136	CBWT1849.b1	GE271181
61915137	CBWT1849.g1	GE271182
61915138	CBWT1850.b1	GE271183
61915139	CBWT1850.g1	GE271184
61915140	CBWT1851.b1	GE271185
61915141	CBWT1851.g1	GE271186
61915142	CBWT1853.g1	GE271187
61915143	CBWT1856.b1	GE271188

61915144	CBWT1856.g1	GE271189
61915145	CBWT1857.b1	GE271190
61915146	CBWT1857.g1	GE271191
61915147	CBWT1858.b1	GE271192
61915148	CBWT1858.g1	GE271193
61915149	CBWT1860.b1	GE271194
61915150	CBWT1860.g1	GE271195
61915151	CBWT1863.b1	GE271196
61915152	CBWT1863.g1	GE271197
61915153	CBWT1864.b1	GE271198
61915154	CBWT1864.g1	GE271199
61915155	CBWT1865.b1	GE271200
61915156	CBWT1865.g1	GE271201
61915157	CBWT1866.g1	GE271202
61915158	CBWT1867.b1	GE271203
61915159	CBWT1867.g1	GE271204
61915160	CBWT1868.b1	GE271205
61915161	CBWT1868.g1	GE271206
61915162	CBWT1869.g1	GE271207
61915163	CBWT1871.b1	GE271208
61915164	CBWT1872.b1	GE271209
61915165	CBWT1872.g1	GE271210
61915166	CBWT1873.g1	GE271211
61915167	CBWT1874.g1	GE271212
61915168	CBWT1875.g1	GE271213
61915169	CBWT1878.g1	GE271214
61915170	CBWT1880.b1	GE271215
61915171	CBWT1880.g1	GE271216
61915172	CBWT1882.b1	GE271217
61915173	CBWT1882.g1	GE271218
61915174	CBWT1884.g1	GE271219
61915175	CBWT1885.b1	GE271220
61915176	CBWT1885.g1	GE271221
61915177	CBWT1887.b1	GE271222
61915178	CBWT1887.g1	GE271223
61915179	CBWT1889.b1	GE271224
61915180	CBWT1889.g1	GE271225
61915181	CBWT1890.b1	GE271226
61915182	CBWT1892.b1	GE271227
61915183	CBWT1892.g1	GE271228
61915184	CBWT1895.b1	GE271229
61915185	CBWT1895.g1	GE271230
61915186	CBWT1897.b1	GE271231
61915187	CBWT1897.g1	GE271232
61915188	CBWT1900.b1	GE271233
61915189	CBWT1900.g1	GE271234
61915190	CBWT1902.b1	GE271235
61915191	CBWT1902.g1	GE271236
61915192	CBWT1903.b1	GE271237
61915193	CBWT1907.b1	GE271238
61915194	CBWT1907.g1	GE271239
61915195	CBWT1908.b1	GE271240
61915196	CBWT1908.g1	GE271241
61915197	CBWT1910.b1	GE271242
61915198	CBWT1910.g1	GE271243
61915199	CBWT1912.b1	GE271244
61915200	CBWT1912.g1	GE271245
61915201	CBWT1913.b1	GE271246
61915202	CBWT1913.g1	GE271247
61915203	CBWT1914.b1	GE271248
61915204	CBWT1914.g1	GE271249
61915205	CBWT1915.b1	GE271250
61915206	CBWT1915.g1	GE271251

61915207	CBWT1916.b1	GE271252
61915208	CBWT1916.g1	GE271253
61915209	CBWT1917.b1	GE271254
61915210	CBWT1917.g1	GE271255
61915211	CBWT1919.g1	GE271256
61915212	CBWT1921.b1	GE271257
61915213	CBWT1921.g1	GE271258
61915214	CBWT1926.b1	GE271259
61915215	CBWT1926.g1	GE271260
61915216	CBWT1930.b1	GE271261
61915217	CBWT1930.g1	GE271262
61915218	CBWT1931.b1	GE271263
61915219	CBWT1931.g1	GE271264
61915220	CBWT1932.b1	GE271265
61915221	CBWT1932.g1	GE271266
61915222	CBWT1933.b1	GE271267
61915223	CBWT1933.g1	GE271268
61915224	CBWT1936.g1	GE271269
61915225	CBWT1937.b1	GE271270
61915226	CBWT1938.b1	GE271271
61915227	CBWT1938.g1	GE271272
61915228	CBWT1939.b1	GE271273
61915229	CBWT1940.b1	GE271274
61915230	CBWT1940.g1	GE271275
61915231	CBWT1941.b1	GE271276
61915232	CBWT1941.g1	GE271277
61915233	CBWT1942.b1	GE271278
61915234	CBWT1942.g1	GE271279
61915235	CBWT1943.b1	GE271280
61915236	CBWT1943.g1	GE271281
61915237	CBWT1944.b1	GE271282
61915238	CBWT1944.g1	GE271283
61915239	CBWT1946.g1	GE271284
61915240	CBWT1947.b1	GE271285
61915241	CBWT1949.g1	GE271286
61915242	CBWT1950.b1	GE271287
61915243	CBWT1952.b1	GE271288
61915244	CBWT1952.g1	GE271289
61915245	CBWT1953.b1	GE271290
61915246	CBWT1953.g1	GE271291
61915247	CBWT1955.b1	GE271292
61915248	CBWT1955.g1	GE271293
61915249	CBWT1958.b1	GE271294
61915250	CBWT1958.g1	GE271295
61915251	CBWT1960.b1	GE271296
61915252	CBWT1960.g1	GE271297
61915253	CBWT1961.b1	GE271298
61915254	CBWT1961.g1	GE271299
61915255	CBWT1964.b1	GE271300
61915256	CBWT1965.b1	GE271301
61915257	CBWT1965.g1	GE271302
61915258	CBWT1970.b1	GE271303
61915259	CBWT1970.g1	GE271304
61915260	CBWT1972.b1	GE271305
61915261	CBWT1972.g1	GE271306
61915262	CBWT1974.b1	GE271307
61915263	CBWT1974.g1	GE271308
61915264	CBWT1975.b1	GE271309
61915265	CBWT1975.g1	GE271310
61915266	CBWT1976.b1	GE271311
61915267	CBWT1976.g1	GE271312
61915268	CBWT1977.b1	GE271313
61915269	CBWT1977.g1	GE271314

61915270	CBWT1978.b1	GE271315
61915271	CBWT1979.b1	GE271316
61915272	CBWT1979.g1	GE271317
61915273	CBWT1980.b1	GE271318
61915274	CBWT1980.g1	GE271319
61915275	CBWT1982.b1	GE271320
61915276	CBWT1982.g1	GE271321
61915277	CBWT1983.b1	GE271322
61915278	CBWT1983.g1	GE271323
61915279	CBWT1984.b1	GE271324
61915280	CBWT1984.g1	GE271325
61915281	CBWT1985.b1	GE271326
61915282	CBWT1985.g1	GE271327
61915283	CBWT1987.b1	GE271328
61915284	CBWT1987.g1	GE271329
61915285	CBWT1990.b1	GE271330
61915286	CBWT1990.g1	GE271331
61915287	CBWT1991.b1	GE271332
61915288	CBWT1991.g1	GE271333
61915289	CBWT1992.b1	GE271334
61915290	CBWT1992.g1	GE271335
61915291	CBWT1993.b1	GE271336
61915292	CBWT1993.g1	GE271337
61915293	CBWT1994.b1	GE271338
61915294	CBWT1994.g1	GE271339
61915295	CBWT1995.b1	GE271340
61915296	CBWT1995.g1	GE271341
61915297	CBWT1996.b1	GE271342
61915298	CBWT1996.g1	GE271343
61915299	CBWT1997.b1	GE271344
61915300	CBWT1997.g1	GE271345
61915301	CBWT1998.b1	GE271346
61915302	CBWT1998.g1	GE271347
61915303	CBWT1999.b1	GE271348
61915304	CBWT1999.g1	GE271349
61915305	CBWT2000.b1	GE271350
61915306	CBWT2000.g1	GE271351
61915307	CBWT2002.b1	GE271352
61915308	CBWT2002.g1	GE271353
61915309	CBWT2003.b1	GE271354
61915310	CBWT2003.g1	GE271355
61915311	CBWT2006.b1	GE271356
61915312	CBWT2008.b1	GE271357
61915313	CBWT2008.g1	GE271358
61915314	CBWT2009.b1	GE271359
61915315	CBWT2009.g1	GE271360
61915316	CBWT2010.g1	GE271361
61915317	CBWT2011.b1	GE271362
61915318	CBWT2011.g1	GE271363
61915319	CBWT2012.b1	GE271364
61915320	CBWT2012.g1	GE271365
61915321	CBWT2013.b1	GE271366
61915322	CBWT2013.g1	GE271367
61915323	CBWT2015.b1	GE271368
61915324	CBWT2015.g1	GE271369
61915325	CBWT2016.b1	GE271370
61915326	CBWT2016.g1	GE271371
61915327	CBWT2017.b1	GE271372
61915328	CBWT2017.g1	GE271373
61915329	CBWT2018.b1	GE271374
61915330	CBWT2018.g1	GE271375
61915331	CBWT2019.b1	GE271376
61915332	CBWT2019.g1	GE271377

61915333	CBWT2020.b1	GE271378
61915334	CBWT2020.g1	GE271379
61915335	CBWT2021.b1	GE271380
61915336	CBWT2021.g1	GE271381
61915337	CBWT2023.b1	GE271382
61915338	CBWT2023.g1	GE271383
61915339	CBWT2024.g1	GE271384
61915340	CBWT2027.b1	GE271385
61915341	CBWT2027.g1	GE271386
61915342	CBWT2028.b1	GE271387
61915343	CBWT2028.g1	GE271388
61915344	CBWT2029.b1	GE271389
61915345	CBWT2029.g1	GE271390
61915346	CBWT2030.b1	GE271391
61915347	CBWT2030.g1	GE271392
61915348	CBWT2031.g1	GE271393
61915349	CBWT2032.b1	GE271394
61915350	CBWT2032.g1	GE271395
61915351	CBWT2034.b1	GE271396
61915352	CBWT2034.g1	GE271397
61915353	CBWT2035.b1	GE271398
61915354	CBWT2035.g1	GE271399
61915355	CBWT2036.b1	GE271400
61915356	CBWT2036.g1	GE271401
61915357	CBWT2037.b1	GE271402
61915358	CBWT2037.g1	GE271403
61915359	CBWT2041.b1	GE271404
61915360	CBWT2041.g1	GE271405
61915361	CBWT2045.b1	GE271406
61915362	CBWT2045.g1	GE271407
61915363	CBWT2048.b1	GE271408
61915364	CBWT2048.g1	GE271409
61915365	CBWT2049.b1	GE271410
61915366	CBWT2049.g1	GE271411
61915367	CBWT2050.b1	GE271412
61915368	CBWT2051.b1	GE271413
61915369	CBWT2051.g1	GE271414
61915370	CBWT2052.b1	GE271415
61915371	CBWT2052.g1	GE271416
61915372	CBWT2054.b1	GE271417
61915373	CBWT2054.g1	GE271418
61915374	CBWT2055.b1	GE271419
61915375	CBWT2055.g1	GE271420
61915376	CBWT2057.b1	GE271421
61915377	CBWT2057.g1	GE271422
61915378	CBWT2058.b1	GE271423
61915379	CBWT2058.g1	GE271424
61915380	CBWT2059.b1	GE271425
61915381	CBWT2059.g1	GE271426
61915382	CBWT2060.b1	GE271427
61915383	CBWT2060.g1	GE271428
61915384	CBWT2061.b1	GE271429
61915385	CBWT2063.b1	GE271430
61915386	CBWT2063.g1	GE271431
61915387	CBWT2064.b1	GE271432
61915388	CBWT2064.g1	GE271433
61915389	CBWT2065.b1	GE271434
61915390	CBWT2066.b1	GE271435
61915391	CBWT2066.g1	GE271436
61915392	CBWT2068.b1	GE271437
61915393	CBWT2068.g1	GE271438
61915394	CBWT2070.b1	GE271439
61915395	CBWT2070.g1	GE271440

61915396	CBWT2073.b1	GE271441
61915397	CBWT2073.g1	GE271442
61915398	CBWT2074.b1	GE271443
61915399	CBWT2074.g1	GE271444
61915400	CBWT2075.g1	GE271445
61915401	CBWT2077.b1	GE271446
61915402	CBWT2077.g1	GE271447
61915403	CBWT2078.b1	GE271448
61915404	CBWT2078.g1	GE271449
61915405	CBWT2079.b1	GE271450
61915406	CBWT2079.g1	GE271451
61915407	CBWT2082.b1	GE271452
61915408	CBWT2082.g1	GE271453
61915409	CBWT2083.b1	GE271454
61915410	CBWT2085.b1	GE271455
61915411	CBWT2085.g1	GE271456
61915412	CBWT2086.b1	GE271457
61915413	CBWT2086.g1	GE271458
61915414	CBWT2087.b1	GE271459
61915415	CBWT2087.g1	GE271460
61915416	CBWT2088.b1	GE271461
61915417	CBWT2088.g1	GE271462
61915418	CBWT2090.b1	GE271463
61915419	CBWT2090.g1	GE271464
61915420	CBWT2092.b1	GE271465
61915421	CBWT2092.g1	GE271466
61915422	CBWT2093.g1	GE271467
61915423	CBWT2095.b1	GE271468
61915424	CBWT2095.g1	GE271469
61915425	CBWT2096.b1	GE271470
61915426	CBWT2096.g1	GE271471
61915427	CBWT2097.b1	GE271472
61915428	CBWT2097.g1	GE271473
61915429	CBWT2098.b1	GE271474
61915430	CBWT2098.g1	GE271475
61915431	CBWT2099.b1	GE271476
61915432	CBWT2099.g1	GE271477
61915433	CBWT2100.b1	GE271478
61915434	CBWT2100.g1	GE271479
61915435	CBWT2101.b1	GE271480
61915436	CBWT2101.g1	GE271481
61915437	CBWT2102.b1	GE271482
61915438	CBWT2102.g1	GE271483
61915439	CBWT2103.b1	GE271484
61915440	CBWT2103.g1	GE271485
61915441	CBWT2104.b1	GE271486
61915442	CBWT2104.g1	GE271487
61915443	CBWT2106.b1	GE271488
61915444	CBWT2106.g1	GE271489
61915445	CBWT2108.b1	GE271490
61915446	CBWT2108.g1	GE271491
61915447	CBWT2109.b1	GE271492
61915448	CBWT2110.b1	GE271493
61915449	CBWT2110.g1	GE271494
61915450	CBWT2111.b1	GE271495
61915451	CBWT2111.g1	GE271496
61915452	CBWT2112.g1	GE271497
61915453	CBWT2113.b1	GE271498
61915454	CBWT2113.g1	GE271499
61915455	CBWT2114.b1	GE271500
61915456	CBWT2114.g1	GE271501
61915457	CBWT2115.b1	GE271502
61915458	CBWT2115.g1	GE271503

61915459	CBWT2116.b1	GE271504
61915460	CBWT2116.g1	GE271505
61915461	CBWT2117.b1	GE271506
61915462	CBWT2117.g1	GE271507
61915463	CBWT2118.b1	GE271508
61915464	CBWT2118.g1	GE271509
61915465	CBWT2119.b1	GE271510
61915466	CBWT2119.g1	GE271511
61915467	CBWT2121.g1	GE271512
61915468	CBWT2122.b1	GE271513
61915469	CBWT2122.g1	GE271514
61915470	CBWT2123.g1	GE271515
61915471	CBWT2125.b1	GE271516
61915472	CBWT2125.g1	GE271517
61915473	CBWT2128.g1	GE271518
61915474	CBWT2130.b1	GE271519
61915475	CBWT2130.g1	GE271520
61915476	CBWT2131.b1	GE271521
61915477	CBWT2133.b1	GE271522
61915478	CBWT2137.g1	GE271523
61915479	CBWT2138.g1	GE271524
61915480	CBWT2139.b1	GE271525
61915481	CBWT2139.g1	GE271526
61915482	CBWT2140.b1	GE271527
61915483	CBWT2140.g1	GE271528
61915484	CBWT2141.b1	GE271529
61915485	CBWT2141.g1	GE271530
61915486	CBWT2142.g1	GE271531
61915487	CBWT2145.b1	GE271532
61915488	CBWT2145.g1	GE271533
61915489	CBWT2146.b1	GE271534
61915490	CBWT2146.g1	GE271535
61915491	CBWT2150.b1	GE271536
61915492	CBWT2150.g1	GE271537
61915493	CBWT2152.g1	GE271538
61915494	CBWT2153.b1	GE271539
61915495	CBWT2153.g1	GE271540
61915496	CBWT2155.b1	GE271541
61915497	CBWT2155.g1	GE271542
61915498	CBWT2157.b1	GE271543
61915499	CBWT2157.g1	GE271544
61915500	CBWT2159.b1	GE271545
61915501	CBWT2162.b1	GE271546
61915502	CBWT2162.g1	GE271547
61915503	CBWT2163.b1	GE271548
61915504	CBWT2163.g1	GE271549
61915505	CBWT2164.b1	GE271550
61915506	CBWT2164.g1	GE271551
61915507	CBWT2166.b1	GE271552
61915508	CBWT2167.b1	GE271553
61915509	CBWT2167.g1	GE271554
61915510	CBWT2168.b1	GE271555
61915511	CBWT2168.g1	GE271556
61915512	CBWT2169.b1	GE271557
61915513	CBWT2169.g1	GE271558
61915514	CBWT2170.b1	GE271559
61915515	CBWT2170.g1	GE271560
61915516	CBWT2171.b1	GE271561
61915517	CBWT2171.g1	GE271562
61915518	CBWT2172.b1	GE271563
61915519	CBWT2172.g1	GE271564
61915520	CBWT2173.b1	GE271565
61915521	CBWT2173.g1	GE271566

61915522	CBWT2174.b1	GE271567
61915523	CBWT2174.g1	GE271568
61915524	CBWT2175.b1	GE271569
61915525	CBWT2175.g1	GE271570
61915526	CBWT2177.b1	GE271571
61915527	CBWT2177.g1	GE271572
61915528	CBWT2178.b1	GE271573
61915529	CBWT2178.g1	GE271574
61915530	CBWT2179.b1	GE271575
61915531	CBWT2179.g1	GE271576
61915532	CBWT2183.b1	GE271577
61915533	CBWT2183.g1	GE271578
61915534	CBWT2184.g1	GE271579
61915535	CBWT2186.b1	GE271580
61915536	CBWT2186.g1	GE271581
61915537	CBWT2187.b1	GE271582
61915538	CBWT2187.g1	GE271583
61915539	CBWT2188.b1	GE271584
61915540	CBWT2188.g1	GE271585
61915541	CBWT2189.b1	GE271586
61915542	CBWT2189.g1	GE271587
61915543	CBWT2192.b1	GE271588
61915544	CBWT2192.g1	GE271589
61915545	CBWT2193.b1	GE271590
61915546	CBWT2193.g1	GE271591
61915547	CBWT2194.b1	GE271592
61915548	CBWT2194.g1	GE271593
61915549	CBWT2195.b1	GE271594
61915550	CBWT2195.g1	GE271595
61915551	CBWT2196.b1	GE271596
61915552	CBWT2196.g1	GE271597
61915553	CBWT2197.b1	GE271598
61915554	CBWT2197.g1	GE271599
61915555	CBWT2198.b1	GE271600
61915556	CBWT2198.g1	GE271601
61915557	CBWT2199.b1	GE271602
61915558	CBWT2199.g1	GE271603
61915559	CBWT2200.b1	GE271604
61915560	CBWT2200.g1	GE271605
61915561	CBWT2201.b1	GE271606
61915562	CBWT2201.g1	GE271607
61915563	CBWT2204.b1	GE271608
61915564	CBWT2206.b1	GE271609
61915565	CBWT2207.b1	GE271610
61915566	CBWT2207.g1	GE271611
61915567	CBWT2208.b1	GE271612
61915568	CBWT2208.g1	GE271613
61915569	CBWT2210.b1	GE271614
61915570	CBWT2210.g1	GE271615
61915571	CBWT2211.b1	GE271616
61915572	CBWT2211.g1	GE271617
61915573	CBWT2213.b1	GE271618
61915574	CBWT2213.g1	GE271619
61915575	CBWT2215.b1	GE271620
61915576	CBWT2215.g1	GE271621
61915577	CBWT2216.b1	GE271622
61915578	CBWT2216.g1	GE271623
61915579	CBWT2218.b1	GE271624
61915580	CBWT2218.g1	GE271625
61915581	CBWT2219.b1	GE271626
61915582	CBWT2219.g1	GE271627
61915583	CBWT2220.b1	GE271628
61915584	CBWT2220.g1	GE271629

61915585	CBWT2221.b1	GE271630
61915586	CBWT2221.g1	GE271631
61915587	CBWT2222.b1	GE271632
61915588	CBWT2222.g1	GE271633
61915589	CBWT2224.b1	GE271634
61915590	CBWT2224.g1	GE271635
61915591	CBWT2225.b1	GE271636
61915592	CBWT2225.g1	GE271637
61915593	CBWT2226.b1	GE271638
61915594	CBWT2226.g1	GE271639
61915595	CBWT2227.b1	GE271640
61915596	CBWT2227.g1	GE271641
61915597	CBWT2229.b1	GE271642
61915598	CBWT2229.g1	GE271643
61915599	CBWT2231.g1	GE271644
61915600	CBWT2232.b1	GE271645
61915601	CBWT2232.g1	GE271646
61915602	CBWT2236.b1	GE271647
61915603	CBWT2236.g1	GE271648
61915604	CBWT2238.b1	GE271649
61915605	CBWT2238.g1	GE271650
61915606	CBWT2240.b1	GE271651
61915607	CBWT2240.g1	GE271652
61915608	CBWT2241.b1	GE271653
61915609	CBWT2241.g1	GE271654
61915610	CBWT2243.b1	GE271655
61915611	CBWT2243.g1	GE271656
61915612	CBWT2244.b1	GE271657
61915613	CBWT2244.g1	GE271658
61915614	CBWT2245.b1	GE271659
61915615	CBWT2245.g1	GE271660
61915616	CBWT2246.b1	GE271661
61915617	CBWT2246.g1	GE271662
61915618	CBWT2247.b1	GE271663
61915619	CBWT2247.g1	GE271664
61915620	CBWT2248.b1	GE271665
61915621	CBWT2248.g1	GE271666
61915622	CBWT2251.g1	GE271667
61915623	CBWT2252.b1	GE271668
61915624	CBWT2252.g1	GE271669
61915625	CBWT2253.b1	GE271670
61915626	CBWT2253.g1	GE271671
61915627	CBWT2254.b1	GE271672
61915628	CBWT2254.g1	GE271673
61915629	CBWT2255.b1	GE271674
61915630	CBWT2256.b1	GE271675
61915631	CBWT2256.g1	GE271676
61915632	CBWT2257.b1	GE271677
61915633	CBWT2257.g1	GE271678
61915634	CBWT2258.b1	GE271679
61915635	CBWT2258.g1	GE271680
61915636	CBWT2259.b1	GE271681
61915637	CBWT2261.b1	GE271682
61915638	CBWT2261.g1	GE271683
61915639	CBWT2262.b1	GE271684
61915640	CBWT2262.g1	GE271685
61915641	CBWT2263.b1	GE271686
61915642	CBWT2263.g1	GE271687
61915643	CBWT2266.b1	GE271688
61915644	CBWT2266.g1	GE271689
61915645	CBWT2269.b1	GE271690
61915646	CBWT2269.g1	GE271691
61915647	CBWT2271.b1	GE271692

61915648	CBWT2271.g1	GE271693
61915649	CBWT2272.b1	GE271694
61915650	CBWT2272.g1	GE271695
61915651	CBWT2273.g1	GE271696
61915652	CBWT2274.b1	GE271697
61915653	CBWT2275.b1	GE271698
61915654	CBWT2276.b1	GE271699
61915655	CBWT2276.g1	GE271700
61915656	CBWT2277.b1	GE271701
61915657	CBWT2277.g1	GE271702
61915658	CBWT2278.b1	GE271703
61915659	CBWT2278.g1	GE271704
61915660	CBWT2279.b1	GE271705
61915661	CBWT2279.g1	GE271706
61915662	CBWT2281.b1	GE271707
61915663	CBWT2281.g1	GE271708
61915664	CBWT2282.g1	GE271709
61915665	CBWT2283.g1	GE271710
61915666	CBWT2284.b1	GE271711
61915667	CBWT2284.g1	GE271712
61915668	CBWT2286.b1	GE271713
61915669	CBWT2286.g1	GE271714
61915670	CBWT2287.b1	GE271715
61915671	CBWT2287.g1	GE271716
61915672	CBWT2289.b1	GE271717
61915673	CBWT2289.g1	GE271718
61915674	CBWT2290.b1	GE271719
61915675	CBWT2290.g1	GE271720
61915676	CBWT2291.b1	GE271721
61915677	CBWT2291.g1	GE271722
61915678	CBWT2292.b1	GE271723
61915679	CBWT2292.g1	GE271724
61915680	CBWT2293.b1	GE271725
61915681	CBWT2293.g1	GE271726
61915682	CBWT2294.b1	GE271727
61915683	CBWT2294.g1	GE271728
61915684	CBWT2297.b1	GE271729
61915685	CBWT2298.b1	GE271730
61915686	CBWT2298.g1	GE271731
61915687	CBWT2299.b1	GE271732
61915688	CBWT2299.g1	GE271733
61915689	CBWT2303.b1	GE271734
61915690	CBWT2303.g1	GE271735
61915691	CBWT2304.b1	GE271736
61915692	CBWT2304.g1	GE271737
61915693	CBWT2305.b1	GE271738
61915694	CBWT2305.g1	GE271739
61915695	CBWT2307.b1	GE271740
61915696	CBWT2309.b1	GE271741
61915697	CBWT2309.g1	GE271742
61915698	CBWT2310.b1	GE271743
61915699	CBWT2310.g1	GE271744
61915700	CBWT2311.b1	GE271745
61915701	CBWT2311.g1	GE271746
61915702	CBWT2312.b1	GE271747
61915703	CBWT2312.g1	GE271748
61915704	CBWT2313.g1	GE271749
61915705	CBWT2315.b1	GE271750
61915706	CBWT2316.g1	GE271751
61915707	CBWT2317.b1	GE271752
61915708	CBWT2318.g1	GE271753
61915709	CBWT2321.b1	GE271754
61915710	CBWT2321.g1	GE271755

61915711	CBWT2322.b1	GE271756
61915712	CBWT2322.g1	GE271757
61915713	CBWT2323.b1	GE271758
61915714	CBWT2326.b1	GE271759
61915715	CBWT2326.g1	GE271760
61915716	CBWT2333.b1	GE271761
61915717	CBWT2333.g1	GE271762
61915718	CBWT2334.b1	GE271763
61915719	CBWT2334.g1	GE271764
61915720	CBWT2335.b1	GE271765
61915721	CBWT2335.g1	GE271766
61915722	CBWT2337.b1	GE271767
61915723	CBWT2337.g1	GE271768
61915724	CBWT2338.b1	GE271769
61915725	CBWT2338.g1	GE271770
61915726	CBWT2339.b1	GE271771
61915727	CBWT2339.g1	GE271772
61915728	CBWT2341.b1	GE271773
61915729	CBWT2341.g1	GE271774
61915730	CBWT2342.b1	GE271775
61915731	CBWT2342.g1	GE271776
61915732	CBWT2344.b1	GE271777
61915733	CBWT2344.g1	GE271778
61915734	CBWT2345.b1	GE271779
61915735	CBWT2345.g1	GE271780
61915736	CBWT2346.b1	GE271781
61915737	CBWT2346.g1	GE271782
61915738	CBWT2347.g1	GE271783
61915739	CBWT2350.b1	GE271784
61915740	CBWT2350.g1	GE271785
61915741	CBWT2351.b1	GE271786
61915742	CBWT2351.g1	GE271787
61915743	CBWT2352.g1	GE271788
61915744	CBWT2355.b1	GE271789
61915745	CBWT2356.b1	GE271790
61915746	CBWT2356.g1	GE271791
61915747	CBWT2357.b1	GE271792
61915748	CBWT2357.g1	GE271793
61915749	CBWT2359.b1	GE271794
61915750	CBWT2359.g1	GE271795
61915751	CBWT2360.b1	GE271796
61915752	CBWT2360.g1	GE271797
61915753	CBWT2362.b1	GE271798
61915754	CBWT2362.g1	GE271799
61915755	CBWT2364.b1	GE271800
61915756	CBWT2365.b1	GE271801
61915757	CBWT2365.g1	GE271802
61915758	CBWT2368.b1	GE271803
61915759	CBWT2368.g1	GE271804
61915760	CBWT2369.b1	GE271805
61915761	CBWT2369.g1	GE271806
61915762	CBWT2370.b1	GE271807
61915763	CBWT2370.g1	GE271808
61915764	CBWT2371.b1	GE271809
61915765	CBWT2371.g1	GE271810
61915766	CBWT2373.b1	GE271811
61915767	CBWT2373.g1	GE271812
61915768	CBWT2374.b1	GE271813
61915769	CBWT2375.g1	GE271814
61915770	CBWT2376.b1	GE271815
61915771	CBWT2376.g1	GE271816
61915772	CBWT2377.b1	GE271817
61915773	CBWT2377.g1	GE271818

61915774	CBWT2378.b1	GE271819
61915775	CBWT2378.g1	GE271820
61915776	CBWT2380.b1	GE271821
61915777	CBWT2380.g1	GE271822
61915778	CBWT2381.b1	GE271823
61915779	CBWT2381.g1	GE271824
61915780	CBWT2384.b1	GE271825
61915781	CBWT2384.g1	GE271826
61915782	CBWT2385.b1	GE271827
61915783	CBWT2387.b1	GE271828
61915784	CBWT2387.g1	GE271829
61915785	CBWT2388.b1	GE271830
61915786	CBWT2388.g1	GE271831
61915787	CBWT2389.b1	GE271832
61915788	CBWT2389.g1	GE271833
61915789	CBWT2390.b1	GE271834
61915790	CBWT2391.b1	GE271835
61915791	CBWT2393.b1	GE271836
61915792	CBWT2395.b1	GE271837
61915793	CBWT2395.g1	GE271838
61915794	CBWT2397.b1	GE271839
61915795	CBWT2397.g1	GE271840
61915796	CBWT2398.b1	GE271841
61915797	CBWT2400.b1	GE271842
61915798	CBWT2400.g1	GE271843
61915799	CBWT2402.b1	GE271844
61915800	CBWT2402.g1	GE271845
61915801	CBWT2403.b1	GE271846
61915802	CBWT2403.g1	GE271847
61915803	CBWT2404.g1	GE271848
61915804	CBWT2405.b1	GE271849
61915805	CBWT2405.g1	GE271850
61915806	CBWT2406.b1	GE271851
61915807	CBWT2408.b1	GE271852
61915808	CBWT2408.g1	GE271853
61915809	CBWT2409.b1	GE271854
61915810	CBWT2409.g1	GE271855
61915811	CBWT2410.b1	GE271856
61915812	CBWT2410.g1	GE271857
61915813	CBWT2412.b1	GE271858
61915814	CBWT2412.g1	GE271859
61915815	CBWT2414.b1	GE271860
61915816	CBWT2414.g1	GE271861
61915817	CBWT2415.b1	GE271862
61915818	CBWT2415.g1	GE271863
61915819	CBWT2416.b1	GE271864
61915820	CBWT2416.g1	GE271865
61915821	CBWT2417.b1	GE271866
61915822	CBWT2417.g1	GE271867
61915823	CBWT2419.b1	GE271868
61915824	CBWT2419.g1	GE271869
61915825	CBWT2421.b1	GE271870
61915826	CBWT2421.g1	GE271871
61915827	CBWT2422.b1	GE271872
61915828	CBWT2422.g1	GE271873
61915829	CBWT2423.b1	GE271874
61915830	CBWT2424.b1	GE271875
61915831	CBWT2425.b1	GE271876
61915832	CBWT2425.g1	GE271877
61915833	CBWT2427.b1	GE271878
61915834	CBWT2427.g1	GE271879
61915835	CBWT2428.g1	GE271880
61915836	CBWT2430.g1	GE271881

61915837	CBWT2432.b1	GE271882
61915838	CBWT2432.g1	GE271883
61915839	CBWT2433.g1	GE271884
61915840	CBWT2434.b1	GE271885
61915841	CBWT2434.g1	GE271886
61915842	CBWT2435.b1	GE271887
61915843	CBWT2435.g1	GE271888
61915844	CBWT2438.b1	GE271889
61915845	CBWT2438.g1	GE271890
61915846	CBWT2439.b1	GE271891
61915847	CBWT2439.g1	GE271892
61915848	CBWT2440.b1	GE271893
61915849	CBWT2440.g1	GE271894
61915850	CBWT2444.b1	GE271895
61915851	CBWT2445.b1	GE271896
61915852	CBWT2447.b1	GE271897
61915853	CBWT2447.g1	GE271898
61915854	CBWT2448.b1	GE271899
61915855	CBWT2448.g1	GE271900
61915856	CBWT2450.b1	GE271901
61915857	CBWT2452.b1	GE271902
61915858	CBWT2452.g1	GE271903
61915859	CBWT2453.b1	GE271904
61915860	CBWT2453.g1	GE271905
61915861	CBWT2454.b1	GE271906
61915862	CBWT2454.g1	GE271907
61915863	CBWT2455.b1	GE271908
61915864	CBWT2455.g1	GE271909
61915865	CBWT2456.b1	GE271910
61915866	CBWT2456.g1	GE271911
61915867	CBWT2457.b1	GE271912
61915868	CBWT2457.g1	GE271913
61915869	CBWT2458.b1	GE271914
61915870	CBWT2458.g1	GE271915
61915871	CBWT2459.b1	GE271916
61915872	CBWT2459.g1	GE271917
61915873	CBWT2460.b1	GE271918
61915874	CBWT2460.g1	GE271919
61915875	CBWT2461.b1	GE271920
61915876	CBWT2461.g1	GE271921
61915877	CBWT2462.g1	GE271922
61915878	CBWT2463.b1	GE271923
61915879	CBWT2463.g1	GE271924
61915880	CBWT2465.b1	GE271925
61915881	CBWT2465.g1	GE271926
61915882	CBWT2466.b1	GE271927
61915883	CBWT2466.g1	GE271928
61915884	CBWT2467.b1	GE271929
61915885	CBWT2467.g1	GE271930
61915886	CBWT2468.b1	GE271931
61915887	CBWT2469.b1	GE271932
61915888	CBWT2469.g1	GE271933
61915889	CBWT2471.b1	GE271934
61915890	CBWT2471.g1	GE271935
61915891	CBWT2472.b1	GE271936
61915892	CBWT2472.g1	GE271937
61915893	CBWT2473.b1	GE271938
61915894	CBWT2473.g1	GE271939
61915895	CBWT2474.b1	GE271940
61915896	CBWT2474.g1	GE271941
61915897	CBWT2476.b1	GE271942
61915898	CBWT2476.g1	GE271943
61915899	CBWT2477.b1	GE271944

61915900	CBWT2477.g1	GE271945
61915901	CBWT2478.b1	GE271946
61915902	CBWT2478.g1	GE271947
61915903	CBWT2479.b1	GE271948
61915904	CBWT2479.g1	GE271949
61915905	CBWT2481.b1	GE271950
61915906	CBWT2481.g1	GE271951
61915907	CBWT2483.b1	GE271952
61915908	CBWT2483.g1	GE271953
61915909	CBWT2484.b1	GE271954
61915910	CBWT2485.b1	GE271955
61915911	CBWT2485.g1	GE271956
61915912	CBWT2486.b1	GE271957
61915913	CBWT2486.g1	GE271958
61915914	CBWT2489.b1	GE271959
61915915	CBWT2493.b1	GE271960
61915916	CBWT2493.g1	GE271961
61915917	CBWT2494.b1	GE271962
61915918	CBWT2495.g1	GE271963
61915919	CBWT2496.b1	GE271964
61915920	CBWT2496.g1	GE271965
61915921	CBWT2497.b1	GE271966
61915922	CBWT2497.g1	GE271967
61915923	CBWT2498.b1	GE271968
61915924	CBWT2498.g1	GE271969
61915925	CBWT2499.b1	GE271970
61915926	CBWT2499.g1	GE271971
61915927	CBWT2500.g1	GE271972
61915928	CBWT2501.b1	GE271973
61915929	CBWT2501.g1	GE271974
61915930	CBWT2504.b1	GE271975
61915931	CBWT2504.g1	GE271976
61915932	CBWT2505.b1	GE271977
61915933	CBWT2505.g1	GE271978
61915934	CBWT2506.b1	GE271979
61915935	CBWT2506.g1	GE271980
61915936	CBWT2510.b1	GE271981
61915937	CBWT2510.g1	GE271982
61915938	CBWT2511.g1	GE271983
61915939	CBWT2512.b1	GE271984
61915940	CBWT2512.g1	GE271985
61915941	CBWT2514.b1	GE271986
61915942	CBWT2514.g1	GE271987
61915943	CBWT2515.b1	GE271988
61915944	CBWT2515.g1	GE271989
61915945	CBWT2516.b1	GE271990
61915946	CBWT2516.g1	GE271991
61915947	CBWT2518.b1	GE271992
61915948	CBWT2518.g1	GE271993
61915949	CBWT2519.b1	GE271994
61915950	CBWT2520.b1	GE271995
61915951	CBWT2524.b1	GE271996
61915952	CBWT2524.g1	GE271997
61915953	CBWT2526.b1	GE271998
61915954	CBWT2526.g1	GE271999
61915955	CBWT2528.b1	GE272000
61915956	CBWT2528.g1	GE272001
61915957	CBWT2529.b1	GE272002
61915958	CBWT2529.g1	GE272003
61915959	CBWT2530.b1	GE272004
61915960	CBWT2530.g1	GE272005
61915961	CBWT2533.b1	GE272006
61915962	CBWT2533.g1	GE272007

61915963	CBWT2536.b1	GE272008
61915964	CBWT2536.g1	GE272009
61915965	CBWT2537.b1	GE272010
61915966	CBWT2537.g1	GE272011
61915967	CBWT2538.b1	GE272012
61915968	CBWT2539.b1	GE272013
61915969	CBWT2539.g1	GE272014
61915970	CBWT2544.g1	GE272015
61915971	CBWT2548.b1	GE272016
61915972	CBWT2548.g1	GE272017
61915973	CBWT2549.b1	GE272018
61915974	CBWT2549.g1	GE272019
61915975	CBWT2551.g1	GE272020
61915976	CBWT2554.b1	GE272021
61915977	CBWT2554.g1	GE272022
61915978	CBWT2555.b1	GE272023
61915979	CBWT2555.g1	GE272024
61915980	CBWT2556.b1	GE272025
61915981	CBWT2557.b1	GE272026
61915982	CBWT2557.g1	GE272027
61915983	CBWT2559.b1	GE272028
61915984	CBWT2559.g1	GE272029
61915985	CBWT2560.b1	GE272030
61915986	CBWT2560.g1	GE272031
61915987	CBWT2561.g1	GE272032
61915988	CBWT2564.b1	GE272033
61915989	CBWT2564.g1	GE272034
61915990	CBWT2566.b1	GE272035
61915991	CBWT2566.g1	GE272036
61915992	CBWT2567.b1	GE272037
61915993	CBWT2567.g1	GE272038
61915994	CBWT2569.b1	GE272039
61915995	CBWT2570.g1	GE272040
61915996	CBWT2572.b1	GE272041
61915997	CBWT2572.g1	GE272042
61915998	CBWT2573.b1	GE272043
61915999	CBWT2573.g1	GE272044
61916000	CBWT2574.b1	GE272045
61916001	CBWT2574.g1	GE272046
61916002	CBWT2578.b1	GE272047
61916003	CBWT2580.b1	GE272048
61916004	CBWT2581.b1	GE272049
61916005	CBWT2581.g1	GE272050
61916006	CBWT2584.b1	GE272051
61916007	CBWT2584.g1	GE272052
61916008	CBWT2585.b1	GE272053
61916009	CBWT2585.g1	GE272054
61916010	CBWT2586.b1	GE272055
61916011	CBWT2586.g1	GE272056
61916012	CBWT2587.b1	GE272057
61916013	CBWT2587.g1	GE272058
61916014	CBWT2588.b1	GE272059
61916015	CBWT2588.g1	GE272060
61916016	CBWT2589.b1	GE272061
61916017	CBWT2589.g1	GE272062
61916018	CBWT2590.g1	GE272063
61916019	CBWT2591.b1	GE272064
61916020	CBWT2591.g1	GE272065
61916021	CBWT2592.b1	GE272066
61916022	CBWT2592.g1	GE272067
61916023	CBWT2593.b1	GE272068
61916024	CBWT2593.g1	GE272069
61916025	CBWT2594.b1	GE272070

61916026	CBWT2594.g1	GE272071
61916027	CBWT2595.b1	GE272072
61916028	CBWT2595.g1	GE272073
61916029	CBWT2596.b1	GE272074
61916030	CBWT2596.g1	GE272075
61916031	CBWT2597.b1	GE272076
61916032	CBWT2598.b1	GE272077
61916033	CBWT2599.b1	GE272078
61916034	CBWT2599.g1	GE272079
61916035	CBWT2600.b1	GE272080
61916036	CBWT2600.g1	GE272081
61916037	CBWT2601.b1	GE272082
61916038	CBWT2601.g1	GE272083
61916039	CBWT2602.b1	GE272084
61916040	CBWT2602.g1	GE272085
61916041	CBWT2603.b1	GE272086
61916042	CBWT2603.g1	GE272087
61916043	CBWT2604.b1	GE272088
61916044	CBWT2604.g1	GE272089
61916045	CBWT2605.g1	GE272090
61916046	CBWT2606.b1	GE272091
61916047	CBWT2606.g1	GE272092
61916048	CBWT2607.g1	GE272093
61916049	CBWT2608.b1	GE272094
61916050	CBWT2608.g1	GE272095
61916051	CBWT2609.b1	GE272096
61916052	CBWT2609.g1	GE272097
61916053	CBWT2610.b1	GE272098
61916054	CBWT2610.g1	GE272099
61916055	CBWT2611.b1	GE272100
61916056	CBWT2611.g1	GE272101
61916057	CBWT2612.b1	GE272102
61916058	CBWT2612.g1	GE272103
61916059	CBWT2615.b1	GE272104
61916060	CBWT2615.g1	GE272105
61916061	CBWT2617.b1	GE272106
61916062	CBWT2617.g1	GE272107
61916063	CBWT2621.b1	GE272108
61916064	CBWT2621.g1	GE272109
61916065	CBWT2623.b1	GE272110
61916066	CBWT2623.g1	GE272111
61916067	CBWT2625.b1	GE272112
61916068	CBWT2625.g1	GE272113
61916069	CBWT2626.b1	GE272114
61916070	CBWT2626.g1	GE272115
61916071	CBWT2627.b1	GE272116
61916072	CBWT2627.g1	GE272117
61916073	CBWT2628.b1	GE272118
61916074	CBWT2628.g1	GE272119
61916075	CBWT2630.b1	GE272120
61916076	CBWT2630.g1	GE272121
61916077	CBWT2631.b1	GE272122
61916078	CBWT2631.g1	GE272123
61916079	CBWT2634.b1	GE272124
61916080	CBWT2634.g1	GE272125
61916081	CBWT2635.b1	GE272126
61916082	CBWT2635.g1	GE272127
61916083	CBWT2636.g1	GE272128
61916084	CBWT2639.b1	GE272129
61916085	CBWT2639.g1	GE272130
61916086	CBWT2641.b1	GE272131
61916087	CBWT2641.g1	GE272132
61916088	CBWT2644.b1	GE272133

61916089	CBWT2644.g1	GE272134
61916090	CBWT2646.b1	GE272135
61916091	CBWT2646.g1	GE272136
61916092	CBWT2647.b1	GE272137
61916093	CBWT2647.g1	GE272138
61916094	CBWT2648.b1	GE272139
61916095	CBWT2648.g1	GE272140
61916096	CBWT2651.b1	GE272141
61916097	CBWT2651.g1	GE272142
61916098	CBWT2655.b1	GE272143
61916099	CBWT2655.g1	GE272144
61916100	CBWT2656.b1	GE272145
61916101	CBWT2657.b1	GE272146
61916102	CBWT2657.g1	GE272147
61916103	CBWT2658.b1	GE272148
61916104	CBWT2658.g1	GE272149
61916105	CBWT2660.b1	GE272150
61916106	CBWT2660.g1	GE272151
61916107	CBWT2661.b1	GE272152
61916108	CBWT2661.g1	GE272153
61916109	CBWT2662.g1	GE272154
61916110	CBWT2663.b1	GE272155
61916111	CBWT2663.g1	GE272156
61916112	CBWT2664.b1	GE272157
61916113	CBWT2664.g1	GE272158
61916114	CBWT2665.b1	GE272159
61916115	CBWT2665.g1	GE272160
61916116	CBWT2666.b1	GE272161
61916117	CBWT2666.g1	GE272162
61916118	CBWT2667.b1	GE272163
61916119	CBWT2667.g1	GE272164
61916120	CBWT2669.b1	GE272165
61916121	CBWT2669.g1	GE272166
61916122	CBWT2670.b1	GE272167
61916123	CBWT2670.g1	GE272168
61916124	CBWT2672.b1	GE272169
61916125	CBWT2672.g1	GE272170
61916126	CBWT2677.b1	GE272171
61916127	CBWT2677.g1	GE272172
61916128	CBWT2678.b1	GE272173
61916129	CBWT2679.b1	GE272174
61916130	CBWT2679.g1	GE272175
61916131	CBWT2680.b1	GE272176
61916132	CBWT2680.g1	GE272177
61916133	CBWT2681.b1	GE272178
61916134	CBWT2683.b1	GE272179
61916135	CBWT2683.g1	GE272180
61916136	CBWT2684.b1	GE272181
61916137	CBWT2684.g1	GE272182
61916138	CBWT2685.b1	GE272183
61916139	CBWT2685.g1	GE272184
61916140	CBWT2686.b1	GE272185
61916141	CBWT2686.g1	GE272186
61916142	CBWT2687.b1	GE272187
61916143	CBWT2688.b1	GE272188
61916144	CBWT2688.g1	GE272189
61916145	CBWT2692.b1	GE272190
61916146	CBWT2692.g1	GE272191
61916147	CBWT2693.b1	GE272192
61916148	CBWT2693.g1	GE272193
61916149	CBWT2694.b1	GE272194
61916150	CBWT2694.g1	GE272195
61916151	CBWT2695.b1	GE272196

61916152	CBWT2695.g1	GE272197
61916153	CBWT2696.b1	GE272198
61916154	CBWT2696.g1	GE272199
61916155	CBWT2699.b1	GE272200
61916156	CBWT2699.g1	GE272201
61916157	CBWT2700.b1	GE272202
61916158	CBWT2700.g1	GE272203
61916159	CBWT2702.b1	GE272204
61916160	CBWT2702.g1	GE272205
61916161	CBWT2704.b1	GE272206
61916162	CBWT2704.g1	GE272207
61916163	CBWT2705.g1	GE272208
61916164	CBWT2707.b1	GE272209
61916165	CBWT2707.g1	GE272210
61916166	CBWT2709.b1	GE272211
61916167	CBWT2709.g1	GE272212
61916168	CBWT2710.b1	GE272213
61916169	CBWT2710.g1	GE272214
61916170	CBWT2711.g1	GE272215
61916171	CBWT2712.b1	GE272216
61916172	CBWT2712.g1	GE272217
61916173	CBWT2713.b1	GE272218
61916174	CBWT2713.g1	GE272219
61916175	CBWT2714.b1	GE272220
61916176	CBWT2714.g1	GE272221
61916177	CBWT2715.b1	GE272222
61916178	CBWT2715.g1	GE272223
61916179	CBWT2719.b1	GE272224
61916180	CBWT2719.g1	GE272225
61916181	CBWT2720.b1	GE272226
61916182	CBWT2720.g1	GE272227
61916183	CBWT2721.b1	GE272228
61916184	CBWT2721.g1	GE272229
61916185	CBWT2722.b1	GE272230
61916186	CBWT2722.g1	GE272231
61916187	CBWT2724.b1	GE272232
61916188	CBWT2724.g1	GE272233
61916189	CBWT2726.b1	GE272234
61916190	CBWT2727.b1	GE272235
61916191	CBWT2727.g1	GE272236
61916192	CBWT2728.b1	GE272237
61916193	CBWT2728.g1	GE272238
61916194	CBWT2729.b1	GE272239
61916195	CBWT2729.g1	GE272240
61916196	CBWT2736.b1	GE272241
61916197	CBWT2736.g1	GE272242
61916198	CBWT2739.b1	GE272243
61916199	CBWT2739.g1	GE272244
61916200	CBWT2741.b1	GE272245
61916201	CBWT2741.g1	GE272246
61916202	CBWT2742.b1	GE272247
61916203	CBWT2742.g1	GE272248
61916204	CBWT2743.b1	GE272249
61916205	CBWT2743.g1	GE272250
61916206	CBWT2744.b1	GE272251
61916207	CBWT2744.g1	GE272252
61916208	CBWT2746.b1	GE272253
61916209	CBWT2746.g1	GE272254
61916210	CBWT2747.b1	GE272255
61916211	CBWT2747.g1	GE272256
61916212	CBWT2748.b1	GE272257
61916213	CBWT2748.g1	GE272258
61916214	CBWT2750.b1	GE272259

61916215	CBWT2750.g1	GE272260
61916216	CBWT2752.b1	GE272261
61916217	CBWT2752.g1	GE272262
61916218	CBWT2754.b1	GE272263
61916219	CBWT2754.g1	GE272264
61916220	CBWT2757.b1	GE272265
61916221	CBWT2757.g1	GE272266
61916222	CBWT2758.b1	GE272267
61916223	CBWT2758.g1	GE272268
61916224	CBWT2759.b1	GE272269
61916225	CBWT2759.g1	GE272270
61916226	CBWT2761.b1	GE272271
61916227	CBWT2763.g1	GE272272
61916228	CBWT2765.b1	GE272273
61916229	CBWT2765.g1	GE272274
61916230	CBWT2766.b1	GE272275
61916231	CBWT2766.g1	GE272276
61916232	CBWT2767.b1	GE272277
61916233	CBWT2767.g1	GE272278
61916234	CBWT2768.b1	GE272279
61916235	CBWT2768.g1	GE272280
61916236	CBWT2769.b1	GE272281
61916237	CBWT2769.g1	GE272282
61916238	CBWT2771.b1	GE272283
61916239	CBWT2771.g1	GE272284
61916240	CBWT2772.b1	GE272285
61916241	CBWT2772.g1	GE272286
61916242	CBWT2773.b1	GE272287
61916243	CBWT2773.g1	GE272288
61916244	CBWT2775.b1	GE272289
61916245	CBWT2775.g1	GE272290
61916246	CBWT2776.b1	GE272291
61916247	CBWT2776.g1	GE272292
61916248	CBWT2777.b1	GE272293
61916249	CBWT2777.g1	GE272294
61916250	CBWT2778.b1	GE272295
61916251	CBWT2778.g1	GE272296
61916252	CBWT2779.b1	GE272297
61916253	CBWT2779.g1	GE272298
61916254	CBWT2780.b1	GE272299
61916255	CBWT2780.g1	GE272300
61916256	CBWT2782.b1	GE272301
61916257	CBWT2782.g1	GE272302
61916258	CBWT2783.b1	GE272303
61916259	CBWT2783.g1	GE272304
61916260	CBWT2786.b1	GE272305
61916261	CBWT2786.g1	GE272306
61916262	CBWT2787.b1	GE272307
61916263	CBWT2787.g1	GE272308
61916264	CBWT2790.b1	GE272309
61916265	CBWT2790.g1	GE272310
61916266	CBWT2791.b1	GE272311
61916267	CBWT2791.g1	GE272312
61916268	CBWT2794.b1	GE272313
61916269	CBWT2794.g1	GE272314
61916270	CBWT2795.b1	GE272315
61916271	CBWT2795.g1	GE272316
61916272	CBWT2796.b1	GE272317
61916273	CBWT2796.g1	GE272318
61916274	CBWT2797.b1	GE272319
61916275	CBWT2797.g1	GE272320
61916276	CBWT2798.b1	GE272321
61916277	CBWT2798.g1	GE272322

61916278	CBWT2799.b1	GE272323
61916279	CBWT2799.g1	GE272324
61916280	CBWT2800.b1	GE272325
61916281	CBWT2800.g1	GE272326
61916282	CBWT2802.b1	GE272327
61916283	CBWT2802.g1	GE272328
61916284	CBWT2803.b1	GE272329
61916285	CBWT2803.g1	GE272330
61916286	CBWT2804.b1	GE272331
61916287	CBWT2804.g1	GE272332
61916288	CBWT2806.b1	GE272333
61916289	CBWT2806.g1	GE272334
61916290	CBWT2807.b1	GE272335
61916291	CBWT2807.g1	GE272336
61916292	CBWT2808.b1	GE272337
61916293	CBWT2808.g1	GE272338
61916294	CBWT2810.g1	GE272339
61916295	CBWT2811.b1	GE272340
61916296	CBWT2811.g1	GE272341
61916297	CBWT2812.b1	GE272342
61916298	CBWT2812.g1	GE272343
61916299	CBWT2813.b1	GE272344
61916300	CBWT2813.g1	GE272345
61916301	CBWT2814.b1	GE272346
61916302	CBWT2814.g1	GE272347
61916303	CBWT2815.b1	GE272348
61916304	CBWT2815.g1	GE272349
61916305	CBWT2816.b1	GE272350
61916306	CBWT2816.g1	GE272351
61916307	CBWT2819.b1	GE272352
61916308	CBWT2819.g1	GE272353
61916309	CBWT2820.b1	GE272354
61916310	CBWT2820.g1	GE272355
61916311	CBWT2821.b1	GE272356
61916312	CBWT2821.g1	GE272357
61916313	CBWT2823.b1	GE272358
61916314	CBWT2823.g1	GE272359
61916315	CBWT2825.b1	GE272360
61916316	CBWT2825.g1	GE272361
61916317	CBWT2826.b1	GE272362
61916318	CBWT2826.g1	GE272363
61916319	CBWT2827.b1	GE272364
61916320	CBWT2827.g1	GE272365
61916321	CBWT2828.b1	GE272366
61916322	CBWT2828.g1	GE272367
61916323	CBWT2829.b1	GE272368
61916324	CBWT2829.g1	GE272369
61916325	CBWT2831.b1	GE272370
61916326	CBWT2832.b1	GE272371
61916327	CBWT2832.g1	GE272372
61916328	CBWT2833.b1	GE272373
61916329	CBWT2833.g1	GE272374
61916330	CBWT2834.b1	GE272375
61916331	CBWT2834.g1	GE272376
61916332	CBWT2835.b1	GE272377
61916333	CBWT2835.g1	GE272378
61916334	CBWT2836.b1	GE272379
61916335	CBWT2836.g1	GE272380
61916336	CBWT2837.b1	GE272381
61916337	CBWT2837.g1	GE272382
61916338	CBWT2838.b1	GE272383
61916339	CBWT2838.g1	GE272384
61916340	CBWT2839.b1	GE272385

61916341	CBWT2839.g1	GE272386
61916342	CBWT2841.b1	GE272387
61916343	CBWT2841.g1	GE272388
61916344	CBWT2842.b1	GE272389
61916345	CBWT2842.g1	GE272390
61916346	CBWT2843.b1	GE272391
61916347	CBWT2843.g1	GE272392
61916348	CBWT2844.b1	GE272393
61916349	CBWT2844.g1	GE272394
61916350	CBWT2845.b1	GE272395
61916351	CBWT2848.b1	GE272396
61916352	CBWT2848.g1	GE272397
61916353	CBWT2849.b1	GE272398
61916354	CBWT2849.g1	GE272399
61916355	CBWT2850.b1	GE272400
61916356	CBWT2850.g1	GE272401
61916357	CBWT2851.b1	GE272402
61916358	CBWT2851.g1	GE272403
61916359	CBWT2853.b1	GE272404
61916360	CBWT2853.g1	GE272405
61916361	CBWT2854.b1	GE272406
61916362	CBWT2854.g1	GE272407
61916363	CBWT2855.b1	GE272408
61916364	CBWT2855.g1	GE272409
61916365	CBWT2856.b1	GE272410
61916366	CBWT2856.g1	GE272411
61916367	CBWT2857.b1	GE272412
61916368	CBWT2857.g1	GE272413
61916369	CBWT2858.b1	GE272414
61916370	CBWT2858.g1	GE272415
61916371	CBWT2859.b1	GE272416
61916372	CBWT2860.b1	GE272417
61916373	CBWT2860.g1	GE272418
61916374	CBWT2861.b1	GE272419
61916375	CBWT2863.b1	GE272420
61916376	CBWT2863.g1	GE272421
61916377	CBWT2864.b1	GE272422
61916378	CBWT2864.g1	GE272423
61916379	CBWT2866.b1	GE272424
61916380	CBWT2867.b1	GE272425
61916381	CBWT2867.g1	GE272426
61916382	CBWT2868.b1	GE272427
61916383	CBWT2868.g1	GE272428
61916384	CBWT2870.b1	GE272429
61916385	CBWT2870.g1	GE272430
61916386	CBWT2871.b1	GE272431
61916387	CBWT2871.g1	GE272432
61916388	CBWT2873.b1	GE272433
61916389	CBWT2873.g1	GE272434
61916390	CBWT2875.b1	GE272435
61916391	CBWT2875.g1	GE272436
61916392	CBWT2878.b1	GE272437
61916393	CBWT2878.g1	GE272438
61916394	CBWT2879.b1	GE272439
61916395	CBWT2881.b1	GE272440
61916396	CBWT2881.g1	GE272441
61916397	CBWT2882.b1	GE272442
61916398	CBWT2882.g1	GE272443
61916399	CBWT2886.b1	GE272444
61916400	CBWT2886.g1	GE272445
61916401	CBWT2888.b1	GE272446
61916402	CBWT2888.g1	GE272447
61916403	CBWT2889.b1	GE272448

61916404	CBWT2889.g1	GE272449
61916405	CBWT2892.g1	GE272450
61916406	CBWT2893.b1	GE272451
61916407	CBWT2893.g1	GE272452
61916408	CBWT2894.b1	GE272453
61916409	CBWT2894.g1	GE272454
61916410	CBWT2895.b1	GE272455
61916411	CBWT2895.g1	GE272456
61916412	CBWT2896.b1	GE272457
61916413	CBWT2896.g1	GE272458
61916414	CBWT2897.b1	GE272459
61916415	CBWT2897.g1	GE272460
61916416	CBWT2898.b1	GE272461
61916417	CBWT2898.g1	GE272462
61916418	CBWT2899.b1	GE272463
61916419	CBWT2899.g1	GE272464
61916420	CBWT2901.b1	GE272465
61916421	CBWT2901.g1	GE272466
61916422	CBWT2902.b1	GE272467
61916423	CBWT2902.g1	GE272468
61916424	CBWT2904.b1	GE272469
61916425	CBWT2904.g1	GE272470
61916426	CBWT2905.b1	GE272471
61916427	CBWT2905.g1	GE272472
61916428	CBWT2906.b1	GE272473
61916429	CBWT2906.g1	GE272474
61916430	CBWT2907.b1	GE272475
61916431	CBWT2907.g1	GE272476
61916432	CBWT2909.b1	GE272477
61916433	CBWT2909.g1	GE272478
61916434	CBWT2910.b1	GE272479
61916435	CBWT2910.g1	GE272480
61916436	CBWT2912.b1	GE272481
61916437	CBWT2912.g1	GE272482
61916438	CBWT2914.b1	GE272483
61916439	CBWT2914.g1	GE272484
61916440	CBWT2916.b1	GE272485
61916441	CBWT2916.g1	GE272486
61916442	CBWT2917.b1	GE272487
61916443	CBWT2917.g1	GE272488
61916444	CBWT2918.b1	GE272489
61916445	CBWT2918.g1	GE272490
61916446	CBWT2919.b1	GE272491
61916447	CBWT2920.b1	GE272492
61916448	CBWT2920.g1	GE272493
61916449	CBWT2921.b1	GE272494
61916450	CBWT2921.g1	GE272495
61916451	CBWT2922.b1	GE272496
61916452	CBWT2922.g1	GE272497
61916453	CBWT2923.b1	GE272498
61916454	CBWT2923.g1	GE272499
61916455	CBWT2924.b1	GE272500
61916456	CBWT2924.g1	GE272501
61916457	CBWT2926.b1	GE272502
61916458	CBWT2926.g1	GE272503
61916459	CBWT2928.b1	GE272504
61916460	CBWT2928.g1	GE272505
61916461	CBWT2929.b1	GE272506
61916462	CBWT2929.g1	GE272507
61916463	CBWT2931.b1	GE272508
61916464	CBWT2931.g1	GE272509
61916465	CBWT2933.b1	GE272510
61916466	CBWT2933.g1	GE272511

61916467	CBWT2934.b1	GE272512
61916468	CBWT2934.g1	GE272513
61916469	CBWT2935.b1	GE272514
61916470	CBWT2937.b1	GE272515
61916471	CBWT2937.g1	GE272516
61916472	CBWT2938.b1	GE272517
61916473	CBWT2938.g1	GE272518
61916474	CBWT2940.g1	GE272519
61916475	CBWT2942.b1	GE272520
61916476	CBWT2945.b1	GE272521
61916477	CBWT2945.g1	GE272522
61916478	CBWT2946.b1	GE272523
61916479	CBWT2946.g1	GE272524
61916480	CBWT2947.b1	GE272525
61916481	CBWT2947.g1	GE272526
61916482	CBWT2948.b1	GE272527
61916483	CBWT2948.g1	GE272528
61916484	CBWT2949.b1	GE272529
61916485	CBWT2949.g1	GE272530
61916486	CBWT2951.b1	GE272531
61916487	CBWT2951.g1	GE272532
61916488	CBWT2953.b1	GE272533
61916489	CBWT2953.g1	GE272534
61916490	CBWT2955.b1	GE272535
61916491	CBWT2955.g1	GE272536
61916492	CBWT2956.b1	GE272537
61916493	CBWT2956.g1	GE272538
61916494	CBWT2958.g1	GE272539
61916495	CBWT2959.b1	GE272540
61916496	CBWT2959.g1	GE272541
61916497	CBWT2960.b1	GE272542
61916498	CBWT2960.g1	GE272543
61916499	CBWT2961.b1	GE272544
61916500	CBWT2961.g1	GE272545
61916501	CBWT2963.b1	GE272546
61916502	CBWT2966.b1	GE272547
61916503	CBWT2966.g1	GE272548
61916504	CBWT2967.b1	GE272549
61916505	CBWT2967.g1	GE272550
61916506	CBWT2968.b1	GE272551
61916507	CBWT2968.g1	GE272552
61916508	CBWT2969.g1	GE272553
61916509	CBWT2970.b1	GE272554
61916510	CBWT2970.g1	GE272555
61916511	CBWT2971.b1	GE272556
61916512	CBWT2971.g1	GE272557
61916513	CBWT2972.b1	GE272558
61916514	CBWT2972.g1	GE272559
61916515	CBWT2973.b1	GE272560
61916516	CBWT2973.g1	GE272561
61916517	CBWT2974.b1	GE272562
61916518	CBWT2974.g1	GE272563
61916519	CBWT2975.b1	GE272564
61916520	CBWT2975.g1	GE272565
61916521	CBWT2977.b1	GE272566
61916522	CBWT2977.g1	GE272567
61916523	CBWT2979.g1	GE272568
61916524	CBWT2980.b1	GE272569
61916525	CBWT2980.g1	GE272570
61916526	CBWT2981.b1	GE272571
61916527	CBWT2981.g1	GE272572
61916528	CBWT2982.b1	GE272573
61916529	CBWT2982.g1	GE272574

61916530	CBWT2983.b1	GE272575
61916531	CBWT2983.g1	GE272576
61916532	CBWT2984.b1	GE272577
61916533	CBWT2984.g1	GE272578
61916534	CBWT2985.b1	GE272579
61916535	CBWT2985.g1	GE272580
61916536	CBWT2987.b1	GE272581
61916537	CBWT2987.g1	GE272582
61916538	CBWT2988.b1	GE272583
61916539	CBWT2989.b1	GE272584
61916540	CBWT2989.g1	GE272585
61916541	CBWT2990.b1	GE272586
61916542	CBWT2990.g1	GE272587
61916543	CBWT2991.b1	GE272588
61916544	CBWT2991.g1	GE272589
61916545	CBWT2992.b1	GE272590
61916546	CBWT2992.g1	GE272591
61916547	CBWT2993.b1	GE272592
61916548	CBWT2993.g1	GE272593
61916549	CBWT2994.b1	GE272594
61916550	CBWT2994.g1	GE272595
61916551	CBWT2995.g1	GE272596
61916552	CBWT2996.b1	GE272597
61916553	CBWT2996.g1	GE272598
61916554	CBWT2998.b1	GE272599
61916555	CBWT2998.g1	GE272600
61916556	CBWT2999.b1	GE272601
61916557	CBWT3000.b1	GE272602
61916558	CBWT3000.g1	GE272603
61916559	CBWT3002.b1	GE272604
61916560	CBWT3004.b1	GE272605
61916561	CBWT3004.g1	GE272606
61916562	CBWT3005.b1	GE272607
61916563	CBWT3005.g1	GE272608
61916564	CBWT3007.b1	GE272609
61916565	CBWT3007.g1	GE272610
61916566	CBWT3009.b1	GE272611
61916567	CBWT3009.g1	GE272612
61916568	CBWT3012.b1	GE272613
61916569	CBWT3012.g1	GE272614
61916570	CBWT3013.b1	GE272615
61916571	CBWT3013.g1	GE272616
61916572	CBWT3014.b1	GE272617
61916573	CBWT3016.b1	GE272618
61916574	CBWT3016.g1	GE272619
61916575	CBWT3017.b1	GE272620
61916576	CBWT3017.g1	GE272621
61916577	CBWT3019.b1	GE272622
61916578	CBWT3020.b1	GE272623
61916579	CBWT3020.g1	GE272624
61916580	CBWT3022.b1	GE272625
61916581	CBWT3023.b1	GE272626
61916582	CBWT3023.g1	GE272627
61916583	CBWT3024.b1	GE272628
61916584	CBWT3024.g1	GE272629
61916585	CBWT3025.b1	GE272630
61916586	CBWT3026.b1	GE272631
61916587	CBWT3026.g1	GE272632
61916588	CBWT3027.b1	GE272633
61916589	CBWT3027.g1	GE272634
61916590	CBWT3028.b1	GE272635
61916591	CBWT3028.g1	GE272636
61916592	CBWT3029.b1	GE272637

61916593	CBWT3029.g1	GE272638
61916594	CBWT3030.b1	GE272639
61916595	CBWT3030.g1	GE272640
61916596	CBWT3031.b1	GE272641
61916597	CBWT3031.g1	GE272642
61916598	CBWT3032.b1	GE272643
61916599	CBWT3032.g1	GE272644
61916600	CBWT3033.b1	GE272645
61916601	CBWT3033.g1	GE272646
61916602	CBWT3034.b1	GE272647
61916603	CBWT3034.g1	GE272648
61916604	CBWT3035.b1	GE272649
61916605	CBWT3035.g1	GE272650
61916606	CBWT3037.b1	GE272651
61916607	CBWT3037.g1	GE272652
61916608	CBWT3040.b1	GE272653
61916609	CBWT3040.g1	GE272654
61916610	CBWT3041.b1	GE272655
61916611	CBWT3041.g1	GE272656
61916612	CBWT3042.b1	GE272657
61916613	CBWT3042.g1	GE272658
61916614	CBWT3044.b1	GE272659
61916615	CBWT3044.g1	GE272660
61916616	CBWT3045.b1	GE272661
61916617	CBWT3045.g1	GE272662
61916618	CBWT3046.b1	GE272663
61916619	CBWT3046.g1	GE272664
61916620	CBWT3047.g1	GE272665
61916621	CBWT3048.b1	GE272666
61916622	CBWT3048.g1	GE272667
61916623	CBWT3050.b1	GE272668
61916624	CBWT3050.g1	GE272669
61916625	CBWT3051.b1	GE272670
61916626	CBWT3051.g1	GE272671
61916627	CBWT3052.b1	GE272672
61916628	CBWT3052.g1	GE272673
61916629	CBWT3054.g1	GE272674
61916630	CBWT3056.b1	GE272675
61916631	CBWT3056.g1	GE272676
61916632	CBWT3057.g1	GE272677
61916633	CBWT3058.b1	GE272678
61916634	CBWT3058.g1	GE272679
61916635	CBWT3059.b1	GE272680
61916636	CBWT3060.b1	GE272681
61916637	CBWT3060.g1	GE272682
61916638	CBWT3062.b1	GE272683
61916639	CBWT3062.g1	GE272684
61916640	CBWT3063.b1	GE272685
61916641	CBWT3063.g1	GE272686
61916642	CBWT3064.b1	GE272687
61916643	CBWT3064.g1	GE272688
61916644	CBWT3066.b1	GE272689
61916645	CBWT3066.g1	GE272690
61916646	CBWT3068.b1	GE272691
61916647	CBWT3068.g1	GE272692
61916648	CBWT3069.b1	GE272693
61916649	CBWT3069.g1	GE272694
61916650	CBWT3070.b1	GE272695
61916651	CBWT3070.g1	GE272696
61916652	CBWT3072.b1	GE272697
61916653	CBWT3072.g1	GE272698
61916654	CBWT3074.b1	GE272699
61916655	CBWT3074.g1	GE272700

61916656	CBWT3075.b1	GE272701
61916657	CBWT3075.g1	GE272702
61916658	CBWT3076.g1	GE272703
61916659	CBWT3077.b1	GE272704
61916660	CBWT3077.g1	GE272705
61916661	CBWT3078.b1	GE272706
61916662	CBWT3078.g1	GE272707
61916663	CBWT3081.b1	GE272708
61916664	CBWT3081.g1	GE272709
61916665	CBWT3084.b1	GE272710
61916666	CBWT3084.g1	GE272711
61916667	CBWT3085.b1	GE272712
61916668	CBWT3085.g1	GE272713
61916669	CBWT3089.b1	GE272714
61916670	CBWT3089.g1	GE272715
61916671	CBWT3091.g1	GE272716
61916672	CBWT3092.b1	GE272717
61916673	CBWT3092.g1	GE272718
61916674	CBWT3093.b1	GE272719
61916675	CBWT3093.g1	GE272720
61916676	CBWT3096.b1	GE272721
61916677	CBWT3096.g1	GE272722
61916678	CBWT3097.b1	GE272723
61916679	CBWT3098.g1	GE272724
61916680	CBWT3099.b1	GE272725
61916681	CBWT3099.g1	GE272726
61916682	CBWT3100.b1	GE272727
61916683	CBWT3100.g1	GE272728
61916684	CBWT3102.b1	GE272729
61916685	CBWT3102.g1	GE272730
61916686	CBWT3106.b1	GE272731
61916687	CBWT3106.g1	GE272732
61916688	CBWT3107.b1	GE272733
61916689	CBWT3108.b1	GE272734
61916690	CBWT3109.b1	GE272735
61916691	CBWT3109.g1	GE272736
61916692	CBWT3110.b1	GE272737
61916693	CBWT3110.g1	GE272738
61916694	CBWT3112.b1	GE272739
61916695	CBWT3112.g1	GE272740
61916696	CBWT3113.g1	GE272741
61916697	CBWT3114.b1	GE272742
61916698	CBWT3114.g1	GE272743
61916699	CBWT3116.b1	GE272744
61916700	CBWT3116.g1	GE272745
61916701	CBWT3118.b1	GE272746
61916702	CBWT3118.g1	GE272747
61916703	CBWT3119.b1	GE272748
61916704	CBWT3119.g1	GE272749
61916705	CBWT3120.b1	GE272750
61916706	CBWT3120.g1	GE272751
61916707	CBWT3121.b1	GE272752
61916708	CBWT3121.g1	GE272753
61916709	CBWT3123.b1	GE272754
61916710	CBWT3123.g1	GE272755
61916711	CBWT3124.b1	GE272756
61916712	CBWT3126.b1	GE272757
61916713	CBWT3126.g1	GE272758
61916714	CBWT3127.b1	GE272759
61916715	CBWT3127.g1	GE272760
61916716	CBWT3128.b1	GE272761
61916717	CBWT3128.g1	GE272762
61916718	CBWT3130.g1	GE272763

61916719	CBWT3131.b1	GE272764
61916720	CBWT3132.b1	GE272765
61916721	CBWT3132.g1	GE272766
61916722	CBWT3134.b1	GE272767
61916723	CBWT3134.g1	GE272768
61916724	CBWT3135.b1	GE272769
61916725	CBWT3135.g1	GE272770
61916726	CBWT3137.b1	GE272771
61916727	CBWT3137.g1	GE272772
61916728	CBWT3138.b1	GE272773
61916729	CBWT3138.g1	GE272774
61916730	CBWT3140.b1	GE272775
61916731	CBWT3140.g1	GE272776
61916732	CBWT3141.b1	GE272777
61916733	CBWT3141.g1	GE272778
61916734	CBWT3142.b1	GE272779
61916735	CBWT3142.g1	GE272780
61916736	CBWT3143.b1	GE272781
61916737	CBWT3143.g1	GE272782
61916738	CBWT3144.b1	GE272783
61916739	CBWT3144.g1	GE272784
61916740	CBWT3145.b1	GE272785
61916741	CBWT3145.g1	GE272786
61916742	CBWT3151.b1	GE272787
61916743	CBWT3151.g1	GE272788
61916744	CBWT3152.b1	GE272789
61916745	CBWT3152.g1	GE272790
61916746	CBWT3153.b1	GE272791
61916747	CBWT3153.g1	GE272792
61916748	CBWT3154.b1	GE272793
61916749	CBWT3155.b1	GE272794
61916750	CBWT3155.g1	GE272795
61916751	CBWT3158.b1	GE272796
61916752	CBWT3158.g1	GE272797
61916753	CBWT3160.b1	GE272798
61916754	CBWT3160.g1	GE272799
61916755	CBWT3161.b1	GE272800
61916756	CBWT3161.g1	GE272801
61916757	CBWT3162.b1	GE272802
61916758	CBWT3162.g1	GE272803
61916759	CBWT3163.b1	GE272804
61916760	CBWT3163.g1	GE272805
61916761	CBWT3164.b1	GE272806
61916762	CBWT3164.g1	GE272807
61916763	CBWT3165.b1	GE272808
61916764	CBWT3165.g1	GE272809
61916765	CBWT3168.b1	GE272810
61916766	CBWT3168.g1	GE272811
61916767	CBWT3169.b1	GE272812
61916768	CBWT3169.g1	GE272813
61916769	CBWT3170.b1	GE272814
61916770	CBWT3170.g1	GE272815
61916771	CBWT3172.b1	GE272816
61916772	CBWT3172.g1	GE272817
61916773	CBWT3173.b1	GE272818
61916774	CBWT3174.b1	GE272819
61916775	CBWT3174.g1	GE272820
61916776	CBWT3175.b1	GE272821
61916777	CBWT3175.g1	GE272822
61916778	CBWT3177.b1	GE272823
61916779	CBWT3177.g1	GE272824
61916780	CBWT3178.b1	GE272825
61916781	CBWT3179.b1	GE272826

61916782	CBWT3179.g1	GE272827
61916783	CBWT3180.b1	GE272828
61916784	CBWT3180.g1	GE272829
61916785	CBWT3181.b1	GE272830
61916786	CBWT3181.g1	GE272831
61916787	CBWT3183.b1	GE272832
61916788	CBWT3183.g1	GE272833
61916789	CBWT3184.b1	GE272834
61916790	CBWT3184.g1	GE272835
61916791	CBWT3185.b1	GE272836
61916792	CBWT3185.g1	GE272837
61916793	CBWT3186.b1	GE272838
61916794	CBWT3186.g1	GE272839
61916795	CBWT3187.b1	GE272840
61916796	CBWT3188.b1	GE272841
61916797	CBWT3188.g1	GE272842
61916798	CBWT3189.b1	GE272843
61916799	CBWT3189.g1	GE272844
61916800	CBWT3190.b1	GE272845
61916801	CBWT3190.g1	GE272846
61916802	CBWT3192.b1	GE272847
61916803	CBWT3192.g1	GE272848
61916804	CBWT3193.b1	GE272849
61916805	CBWT3193.g1	GE272850
61916806	CBWT3194.b1	GE272851
61916807	CBWT3194.g1	GE272852
61916808	CBWT3195.b1	GE272853
61916809	CBWT3195.g1	GE272854
61916810	CBWT3197.b1	GE272855
61916811	CBWT3197.g1	GE272856
61916812	CBWT3198.b1	GE272857
61916813	CBWT3198.g1	GE272858
61916814	CBWT3199.b1	GE272859
61916815	CBWT3200.b1	GE272860
61916816	CBWT3200.g1	GE272861
61916817	CBWT3201.b1	GE272862
61916818	CBWT3201.g1	GE272863
61916819	CBWT3202.b1	GE272864
61916820	CBWT3202.g1	GE272865
61916821	CBWT3203.b1	GE272866
61916822	CBWT3204.b1	GE272867
61916823	CBWT3204.g1	GE272868
61916824	CBWT3207.b1	GE272869
61916825	CBWT3207.g1	GE272870
61916826	CBWT3208.b1	GE272871
61916827	CBWT3208.g1	GE272872
61916828	CBWT3209.b1	GE272873
61916829	CBWT3209.g1	GE272874
61916830	CBWT3212.b1	GE272875
61916831	CBWT3212.g1	GE272876
61916832	CBWT3214.b1	GE272877
61916833	CBWT3214.g1	GE272878
61916834	CBWT3216.b1	GE272879
61916835	CBWT3216.g1	GE272880
61916836	CBWT3217.b1	GE272881
61916837	CBWT3217.g1	GE272882
61916838	CBWT3219.b1	GE272883
61916839	CBWT3219.g1	GE272884
61916840	CBWT3221.b1	GE272885
61916841	CBWT3221.g1	GE272886
61916842	CBWT3222.g1	GE272887
61916843	CBWT3223.b1	GE272888
61916844	CBWT3223.g1	GE272889

61916845	CBWT3224.b1	GE272890
61916846	CBWT3224.g1	GE272891
61916847	CBWT3226.b1	GE272892
61916848	CBWT3226.g1	GE272893
61916849	CBWT3228.b1	GE272894
61916850	CBWT3228.g1	GE272895
61916851	CBWT3230.b1	GE272896
61916852	CBWT3230.g1	GE272897
61916853	CBWT3231.b1	GE272898
61916854	CBWT3232.b1	GE272899
61916855	CBWT3232.g1	GE272900
61916856	CBWT3234.b1	GE272901
61916857	CBWT3234.g1	GE272902
61916858	CBWT3235.b1	GE272903
61916859	CBWT3235.g1	GE272904
61916860	CBWT3236.b1	GE272905
61916861	CBWT3236.g1	GE272906
61916862	CBWT3237.b1	GE272907
61916863	CBWT3237.g1	GE272908
61916864	CBWT3238.b1	GE272909
61916865	CBWT3238.g1	GE272910
61916866	CBWT3240.b1	GE272911
61916867	CBWT3240.g1	GE272912
61916868	CBWT3242.b1	GE272913
61916869	CBWT3242.g1	GE272914
61916870	CBWT3243.b1	GE272915
61916871	CBWT3243.g1	GE272916
61916872	CBWT3244.b1	GE272917
61916873	CBWT3244.g1	GE272918
61916874	CBWT3245.b1	GE272919
61916875	CBWT3245.g1	GE272920
61916876	CBWT3246.b1	GE272921
61916877	CBWT3246.g1	GE272922
61916878	CBWT3247.g1	GE272923
61916879	CBWT3248.b1	GE272924
61916880	CBWT3248.g1	GE272925
61916881	CBWT3249.b1	GE272926
61916882	CBWT3249.g1	GE272927
61916883	CBWT3250.b1	GE272928
61916884	CBWT3251.b1	GE272929
61916885	CBWT3251.g1	GE272930
61916886	CBWT3252.b1	GE272931
61916887	CBWT3252.g1	GE272932
61916888	CBWT3253.b1	GE272933
61916889	CBWT3253.g1	GE272934
61916890	CBWT3256.b1	GE272935
61916891	CBWT3256.g1	GE272936
61916892	CBWT3257.b1	GE272937
61916893	CBWT3257.g1	GE272938
61916894	CBWT3258.b1	GE272939
61916895	CBWT3258.g1	GE272940
61916896	CBWT3259.b1	GE272941
61916897	CBWT3259.g1	GE272942
61916898	CBWT3260.b1	GE272943
61916899	CBWT3260.g1	GE272944
61916900	CBWT3263.b1	GE272945
61916901	CBWT3263.g1	GE272946
61916902	CBWT3264.b1	GE272947
61916903	CBWT3265.b1	GE272948
61916904	CBWT3265.g1	GE272949
61916905	CBWT3266.b1	GE272950
61916906	CBWT3266.g1	GE272951
61916907	CBWT3267.b1	GE272952

61916908	CBWT3267.g1	GE272953
61916909	CBWT3268.b1	GE272954
61916910	CBWT3268.g1	GE272955
61916911	CBWT3269.b1	GE272956
61916912	CBWT3269.g1	GE272957
61916913	CBWT3270.b1	GE272958
61916914	CBWT3270.g1	GE272959
61916915	CBWT3271.b1	GE272960
61916916	CBWT3271.g1	GE272961
61916917	CBWT3273.b1	GE272962
61916918	CBWT3273.g1	GE272963
61916919	CBWT3275.b1	GE272964
61916920	CBWT3275.g1	GE272965
61916921	CBWT3278.b1	GE272966
61916922	CBWT3279.b1	GE272967
61916923	CBWT3279.g1	GE272968
61916924	CBWT3280.b1	GE272969
61916925	CBWT3280.g1	GE272970
61916926	CBWT3284.g1	GE272971
61916927	CBWT3285.b1	GE272972
61916928	CBWT3286.b1	GE272973
61916929	CBWT3286.g1	GE272974
61916930	CBWT3289.b1	GE272975
61916931	CBWT3289.g1	GE272976
61916932	CBWT3290.b1	GE272977
61916933	CBWT3290.g1	GE272978
61916934	CBWT3291.g1	GE272979
61916935	CBWT3292.b1	GE272980
61916936	CBWT3292.g1	GE272981
61916937	CBWT3293.b1	GE272982
61916938	CBWT3293.g1	GE272983
61916939	CBWT3294.b1	GE272984
61916940	CBWT3294.g1	GE272985
61916941	CBWT3295.b1	GE272986
61916942	CBWT3295.g1	GE272987
61916943	CBWT3297.b1	GE272988
61916944	CBWT3297.g1	GE272989
61916945	CBWT3298.b1	GE272990
61916946	CBWT3298.g1	GE272991
61916947	CBWT3299.b1	GE272992
61916948	CBWT3300.b1	GE272993
61916949	CBWT3300.g1	GE272994
61916950	CBWT3301.b1	GE272995
61916951	CBWT3301.g1	GE272996
61916952	CBWT3302.b1	GE272997
61916953	CBWT3302.g1	GE272998
61916954	CBWT3303.b1	GE272999
61916955	CBWT3303.g1	GE273000
61916956	CBWT3304.b1	GE273001
61916957	CBWT3304.g1	GE273002
61916958	CBWT3305.b1	GE273003
61916959	CBWT3305.g1	GE273004
61916960	CBWT3306.b1	GE273005
61916961	CBWT3306.g1	GE273006
61916962	CBWT3307.b1	GE273007
61916963	CBWT3311.b1	GE273008
61916964	CBWT3311.g1	GE273009
61916965	CBWT3313.b1	GE273010
61916966	CBWT3313.g1	GE273011
61916967	CBWT3314.b1	GE273012
61916968	CBWT3314.g1	GE273013
61916969	CBWT3317.b1	GE273014
61916970	CBWT3317.g1	GE273015

61916971	CBWT3319.b1	GE273016
61916972	CBWT3319.g1	GE273017
61916973	CBWT3320.b1	GE273018
61916974	CBWT3320.g1	GE273019
61916975	CBWT3321.b1	GE273020
61916976	CBWT3321.g1	GE273021
61916977	CBWT3322.b1	GE273022
61916978	CBWT3324.b1	GE273023
61916979	CBWT3325.b1	GE273024
61916980	CBWT3325.g1	GE273025
61916981	CBWT3326.b1	GE273026
61916982	CBWT3326.g1	GE273027
61916983	CBWT3327.b1	GE273028
61916984	CBWT3328.b1	GE273029
61916985	CBWT3328.g1	GE273030
61916986	CBWT3330.b1	GE273031
61916987	CBWT3330.g1	GE273032
61916988	CBWT3331.g1	GE273033
61916989	CBWT3333.b1	GE273034
61916990	CBWT3333.g1	GE273035
61916991	CBWT3334.b1	GE273036
61916992	CBWT3334.g1	GE273037
61916993	CBWT3335.b1	GE273038
61916994	CBWT3335.g1	GE273039
61916995	CBWT3338.b1	GE273040
61916996	CBWT3338.g1	GE273041
61916997	CBWT3339.b1	GE273042
61916998	CBWT3339.g1	GE273043
61916999	CBWT3340.b1	GE273044
61917000	CBWT3340.g1	GE273045
61917001	CBWT3343.b1	GE273046
61917002	CBWT3343.g1	GE273047
61917003	CBWT3345.b1	GE273048
61917004	CBWT3345.g1	GE273049
61917005	CBWT3346.b1	GE273050
61917006	CBWT3346.g1	GE273051
61917007	CBWT3348.g1	GE273052
61917008	CBWT3350.b1	GE273053
61917009	CBWT3350.g1	GE273054
61917010	CBWT3354.b1	GE273055
61917011	CBWT3354.g1	GE273056
61917012	CBWT3355.b1	GE273057
61917013	CBWT3355.g1	GE273058
61917014	CBWT3356.g1	GE273059
61917015	CBWT3358.b1	GE273060
61917016	CBWT3358.g1	GE273061
61917017	CBWT3359.b1	GE273062
61917018	CBWT3359.g1	GE273063
61917019	CBWT3360.b1	GE273064
61917020	CBWT3360.g1	GE273065
61917021	CBWT3361.b1	GE273066
61917022	CBWT3361.g1	GE273067
61917023	CBWT3362.b1	GE273068
61917024	CBWT3362.g1	GE273069
61917025	CBWT3363.b1	GE273070
61917026	CBWT3363.g1	GE273071
61917027	CBWT3364.b1	GE273072
61917028	CBWT3365.g1	GE273073
61917029	CBWT3367.b1	GE273074
61917030	CBWT3367.g1	GE273075
61917031	CBWT3369.b1	GE273076
61917032	CBWT3369.g1	GE273077
61917033	CBWT3370.g1	GE273078

61917034	CBWT3371.b1	GE273079
61917035	CBWT3373.b1	GE273080
61917036	CBWT3373.g1	GE273081
61917037	CBWT3374.g1	GE273082
61917038	CBWT3375.b1	GE273083
61917039	CBWT3375.g1	GE273084
61917040	CBWT3377.b1	GE273085
61917041	CBWT3377.g1	GE273086
61917042	CBWT3378.b1	GE273087
61917043	CBWT3378.g1	GE273088
61917044	CBWT3379.b1	GE273089
61917045	CBWT3379.g1	GE273090
61917046	CBWT3381.b1	GE273091
61917047	CBWT3381.g1	GE273092
61917048	CBWT3382.b1	GE273093
61917049	CBWT3382.g1	GE273094
61917050	CBWT3383.b1	GE273095
61917051	CBWT3383.g1	GE273096
61917052	CBWT3385.b1	GE273097
61917053	CBWT3385.g1	GE273098
61917054	CBWT3386.b1	GE273099
61917055	CBWT3386.g1	GE273100
61917056	CBWT3388.b1	GE273101
61917057	CBWT3388.g1	GE273102
61917058	CBWT3389.b1	GE273103
61917059	CBWT3389.g1	GE273104
61917060	CBWT3390.b1	GE273105
61917061	CBWT3392.b1	GE273106
61917062	CBWT3392.g1	GE273107
61917063	CBWT3393.b1	GE273108
61917064	CBWT3393.g1	GE273109
61917065	CBWT3395.b1	GE273110
61917066	CBWT3395.g1	GE273111
61917067	CBWT3396.b1	GE273112
61917068	CBWT3398.b1	GE273113
61917069	CBWT3398.g1	GE273114
61917070	CBWT3399.b1	GE273115
61917071	CBWT3399.g1	GE273116
61917072	CBWT3400.b1	GE273117
61917073	CBWT3400.g1	GE273118
61917074	CBWT3401.b1	GE273119
61917075	CBWT3401.g1	GE273120
61917076	CBWT3402.g1	GE273121
61917077	CBWT3403.g1	GE273122
61917078	CBWT3406.b1	GE273123
61917079	CBWT3406.g1	GE273124
61917080	CBWT3407.b1	GE273125
61917081	CBWT3407.g1	GE273126
61917082	CBWT3408.g1	GE273127
61917083	CBWT3409.b1	GE273128
61917084	CBWT3409.g1	GE273129
61917085	CBWT3410.b1	GE273130
61917086	CBWT3410.g1	GE273131
61917087	CBWT3411.b1	GE273132
61917088	CBWT3411.g1	GE273133
61917089	CBWT3412.b1	GE273134
61917090	CBWT3412.g1	GE273135
61917091	CBWT3413.b1	GE273136
61917092	CBWT3413.g1	GE273137
61917093	CBWT3414.b1	GE273138
61917094	CBWT3414.g1	GE273139
61917095	CBWT3415.b1	GE273140
61917096	CBWT3415.g1	GE273141

61917097	CBWT3416.b1	GE273142
61917098	CBWT3416.g1	GE273143
61917099	CBWT3418.b1	GE273144
61917100	CBWT3419.b1	GE273145
61917101	CBWT3419.g1	GE273146
61917102	CBWT3422.b1	GE273147
61917103	CBWT3422.g1	GE273148
61917104	CBWT3423.g1	GE273149
61917105	CBWT3424.b1	GE273150
61917106	CBWT3424.g1	GE273151
61917107	CBWT3425.b1	GE273152
61917108	CBWT3425.g1	GE273153
61917109	CBWT3426.b1	GE273154
61917110	CBWT3426.g1	GE273155
61917111	CBWT3429.b1	GE273156
61917112	CBWT3429.g1	GE273157
61917113	CBWT3432.b1	GE273158
61917114	CBWT3432.g1	GE273159
61917115	CBWT3433.b1	GE273160
61917116	CBWT3433.g1	GE273161
61917117	CBWT3434.b1	GE273162
61917118	CBWT3434.g1	GE273163
61917119	CBWT3435.b1	GE273164
61917120	CBWT3435.g1	GE273165
61917121	CBWT3436.g1	GE273166
61917122	CBWT3438.b1	GE273167
61917123	CBWT3438.g1	GE273168
61917124	CBWT3439.b1	GE273169
61917125	CBWT3439.g1	GE273170
61917126	CBWT3443.b1	GE273171
61917127	CBWT3443.g1	GE273172
61917128	CBWT3445.g1	GE273173
61917129	CBWT3446.b1	GE273174
61917130	CBWT3446.g1	GE273175
61917131	CBWT3447.b1	GE273176
61917132	CBWT3447.g1	GE273177
61917133	CBWT3448.b1	GE273178
61917134	CBWT3448.g1	GE273179
61917135	CBWT3449.b1	GE273180
61917136	CBWT3449.g1	GE273181
61917137	CBWT3450.b1	GE273182
61917138	CBWT3450.g1	GE273183
61917139	CBWT3451.b1	GE273184
61917140	CBWT3451.g1	GE273185
61917141	CBWT3452.b1	GE273186
61917142	CBWT3452.g1	GE273187
61917143	CBWT3453.b1	GE273188
61917144	CBWT3453.g1	GE273189
61917145	CBWT3455.b1	GE273190
61917146	CBWT3455.g1	GE273191
61917147	CBWT3457.b1	GE273192
61917148	CBWT3457.g1	GE273193
61917149	CBWT3458.b1	GE273194
61917150	CBWT3458.g1	GE273195
61917151	CBWT3461.b1	GE273196
61917152	CBWT3461.g1	GE273197
61917153	CBWT3462.g1	GE273198
61917154	CBWT3465.b1	GE273199
61917155	CBWT3465.g1	GE273200
61917156	CBWT3466.b1	GE273201
61917157	CBWT3466.g1	GE273202
61917158	CBWT3468.b1	GE273203
61917159	CBWT3469.b1	GE273204

61917160	CBWT3469.g1	GE273205
61917161	CBWT3470.b1	GE273206
61917162	CBWT3470.g1	GE273207
61917163	CBWT3471.b1	GE273208
61917164	CBWT3471.g1	GE273209
61917165	CBWT3472.b1	GE273210
61917166	CBWT3472.g1	GE273211
61917167	CBWT3476.b1	GE273212
61917168	CBWT3476.g1	GE273213
61917169	CBWT3481.b1	GE273214
61917170	CBWT3481.g1	GE273215
61917171	CBWT3482.b1	GE273216
61917172	CBWT3482.g1	GE273217
61917173	CBWT3483.b1	GE273218
61917174	CBWT3483.g1	GE273219
61917175	CBWT3484.b1	GE273220
61917176	CBWT3485.g1	GE273221
61917177	CBWT3489.b1	GE273222
61917178	CBWT3489.g1	GE273223
61917179	CBWT3490.b1	GE273224
61917180	CBWT3490.g1	GE273225
61917181	CBWT3491.b1	GE273226
61917182	CBWT3491.g1	GE273227
61917183	CBWT3492.b1	GE273228
61917184	CBWT3492.g1	GE273229
61917185	CBWT3494.b1	GE273230
61917186	CBWT3494.g1	GE273231
61917187	CBWT3496.b1	GE273232
61917188	CBWT3496.g1	GE273233
61917189	CBWT3498.b1	GE273234
61917190	CBWT3498.g1	GE273235
61917191	CBWT3499.b1	GE273236
61917192	CBWT3502.b1	GE273237
61917193	CBWT3502.g1	GE273238
61917194	CBWT3503.b1	GE273239
61917195	CBWT3503.g1	GE273240
61917196	CBWT3504.b1	GE273241
61917197	CBWT3504.g1	GE273242
61917198	CBWT3505.b1	GE273243
61917199	CBWT3505.g1	GE273244
61917200	CBWT3506.b1	GE273245
61917201	CBWT3506.g1	GE273246
61917202	CBWT3508.b1	GE273247
61917203	CBWT3508.g1	GE273248
61917204	CBWT3510.b1	GE273249
61917205	CBWT3510.g1	GE273250
61917206	CBWT3511.b1	GE273251
61917207	CBWT3511.g1	GE273252
61917208	CBWT3512.b1	GE273253
61917209	CBWT3512.g1	GE273254
61917210	CBWT3513.b1	GE273255
61917211	CBWT3513.g1	GE273256
61917212	CBWT3516.b1	GE273257
61917213	CBWT3516.g1	GE273258
61917214	CBWT3517.b1	GE273259
61917215	CBWT3517.g1	GE273260
61917216	CBWT3518.b1	GE273261
61917217	CBWT3518.g1	GE273262
61917218	CBWT3519.b1	GE273263
61917219	CBWT3519.g1	GE273264
61917220	CBWT3520.b1	GE273265
61917221	CBWT3520.g1	GE273266
61917222	CBWT3521.b1	GE273267

61917223	CBWT3521.g1	GE273268
61917224	CBWT3522.b1	GE273269
61917225	CBWT3522.g1	GE273270
61917226	CBWT3525.b1	GE273271
61917227	CBWT3525.g1	GE273272
61917228	CBWT3526.b1	GE273273
61917229	CBWT3527.b1	GE273274
61917230	CBWT3527.g1	GE273275
61917231	CBWT3530.g1	GE273276
61917232	CBWT3532.b1	GE273277
61917233	CBWT3532.g1	GE273278
61917234	CBWT3533.b1	GE273279
61917235	CBWT3533.g1	GE273280
61917236	CBWT3534.b1	GE273281
61917237	CBWT3534.g1	GE273282
61917238	CBWT3536.b1	GE273283
61917239	CBWT3537.b1	GE273284
61917240	CBWT3538.b1	GE273285
61917241	CBWT3540.b1	GE273286
61917242	CBWT3540.g1	GE273287
61917243	CBWT3543.b1	GE273288
61917244	CBWT3543.g1	GE273289
61917245	CBWT3544.b1	GE273290
61917246	CBWT3544.g1	GE273291
61917247	CBWT3545.b1	GE273292
61917248	CBWT3545.g1	GE273293
61917249	CBWT3546.b1	GE273294
61917250	CBWT3546.g1	GE273295
61917251	CBWT3547.b1	GE273296
61917252	CBWT3548.b1	GE273297
61917253	CBWT3548.g1	GE273298
61917254	CBWT3549.b1	GE273299
61917255	CBWT3549.g1	GE273300
61917256	CBWT3550.b1	GE273301
61917257	CBWT3550.g1	GE273302
61917258	CBWT3551.b1	GE273303
61917259	CBWT3551.g1	GE273304
61917260	CBWT3554.b1	GE273305
61917261	CBWT3555.b1	GE273306
61917262	CBWT3555.g1	GE273307
61917263	CBWT3556.b1	GE273308
61917264	CBWT3556.g1	GE273309
61917265	CBWT3557.b1	GE273310
61917266	CBWT3557.g1	GE273311
61917267	CBWT3558.b1	GE273312
61917268	CBWT3558.g1	GE273313
61917269	CBWT3559.b1	GE273314
61917270	CBWT3559.g1	GE273315
61917271	CBWT3560.g1	GE273316
61917272	CBWT3561.b1	GE273317
61917273	CBWT3562.b1	GE273318
61917274	CBWT3562.g1	GE273319
61917275	CBWT3563.b1	GE273320
61917276	CBWT3563.g1	GE273321
61917277	CBWT3564.g1	GE273322
61917278	CBWT3565.b1	GE273323
61917279	CBWT3567.b1	GE273324
61917280	CBWT3567.g1	GE273325
61917281	CBWT3568.b1	GE273326
61917282	CBWT3568.g1	GE273327
61917283	CBWT3569.g1	GE273328
61917284	CBWT3570.b1	GE273329
61917285	CBWT3570.g1	GE273330

61917286	CBWT3571.b1	GE273331
61917287	CBWT3573.b1	GE273332
61917288	CBWT3573.g1	GE273333
61917289	CBWT3576.b1	GE273334
61917290	CBWT3576.g1	GE273335
61917291	CBWT3577.b1	GE273336
61917292	CBWT3577.g1	GE273337
61917293	CBWT3579.b1	GE273338
61917294	CBWT3579.g1	GE273339
61917295	CBWT3580.b1	GE273340
61917296	CBWT3580.g1	GE273341
61917297	CBWT3581.b1	GE273342
61917298	CBWT3581.g1	GE273343
61917299	CBWT3583.b1	GE273344
61917300	CBWT3583.g1	GE273345
61917301	CBWT3584.g1	GE273346
61917302	CBWT3585.b1	GE273347
61917303	CBWT3585.g1	GE273348
61917304	CBWT3587.b1	GE273349
61917305	CBWT3587.g1	GE273350
61917306	CBWT3590.b1	GE273351
61917307	CBWT3590.g1	GE273352
61917308	CBWT3591.b1	GE273353
61917309	CBWT3591.g1	GE273354
61917310	CBWT3592.b1	GE273355
61917311	CBWT3592.g1	GE273356
61917312	CBWT3594.b1	GE273357
61917313	CBWT3594.g1	GE273358
61917314	CBWT3595.b1	GE273359
61917315	CBWT3596.b1	GE273360
61917316	CBWT3596.g1	GE273361
61917317	CBWT3597.b1	GE273362
61917318	CBWT3597.g1	GE273363
61917319	CBWT3599.b1	GE273364
61917320	CBWT3599.g1	GE273365
61917321	CBWT3600.b1	GE273366
61917322	CBWT3600.g1	GE273367
61917323	CBWT3601.b1	GE273368
61917324	CBWT3601.g1	GE273369
61917325	CBWT3602.b1	GE273370
61917326	CBWT3602.g1	GE273371
61917327	CBWT3603.g1	GE273372
61917328	CBWT3604.b1	GE273373
61917329	CBWT3604.g1	GE273374
61917330	CBWT3606.g1	GE273375
61917331	CBWT3607.b1	GE273376
61917332	CBWT3607.g1	GE273377
61917333	CBWT3608.b1	GE273378
61917334	CBWT3608.g1	GE273379
61917335	CBWT3609.b1	GE273380
61917336	CBWT3609.g1	GE273381
61917337	CBWT3612.g1	GE273382
61917338	CBWT3617.b1	GE273383
61917339	CBWT3617.g1	GE273384
61917340	CBWT3618.b1	GE273385
61917341	CBWT3618.g1	GE273386
61917342	CBWT3620.b1	GE273387
61917343	CBWT3620.g1	GE273388
61917344	CBWT3621.b1	GE273389
61917345	CBWT3621.g1	GE273390
61917346	CBWT3622.b1	GE273391
61917347	CBWT3622.g1	GE273392
61917348	CBWT3625.g1	GE273393

61917349	CBWT3626.g1	GE273394
61917350	CBWT3629.b1	GE273395
61917351	CBWT3629.g1	GE273396
61917352	CBWT3630.b1	GE273397
61917353	CBWT3630.g1	GE273398
61917354	CBWT3631.b1	GE273399
61917355	CBWT3631.g1	GE273400
61917356	CBWT3632.b1	GE273401
61917357	CBWT3632.g1	GE273402
61917358	CBWT3633.b1	GE273403
61917359	CBWT3633.g1	GE273404
61917360	CBWT3634.b1	GE273405
61917361	CBWT3634.g1	GE273406
61917362	CBWT3636.b1	GE273407
61917363	CBWT3636.g1	GE273408
61917364	CBWT3639.b1	GE273409
61917365	CBWT3639.g1	GE273410
61917366	CBWT3641.b1	GE273411
61917367	CBWT3641.g1	GE273412
61917368	CBWT3643.b1	GE273413
61917369	CBWT3643.g1	GE273414
61917370	CBWT3646.b1	GE273415
61917371	CBWT3646.g1	GE273416
61917372	CBWT3647.b1	GE273417
61917373	CBWT3647.g1	GE273418
61917374	CBWT3648.b1	GE273419
61917375	CBWT3648.g1	GE273420
61917376	CBWT3649.b1	GE273421
61917377	CBWT3649.g1	GE273422
61917378	CBWT3652.b1	GE273423
61917379	CBWT3652.g1	GE273424
61917380	CBWT3653.b1	GE273425
61917381	CBWT3653.g1	GE273426
61917382	CBWT3654.b1	GE273427
61917383	CBWT3654.g1	GE273428
61917384	CBWT3655.b1	GE273429
61917385	CBWT3655.g1	GE273430
61917386	CBWT3656.b1	GE273431
61917387	CBWT3656.g1	GE273432
61917388	CBWT3657.b1	GE273433
61917389	CBWT3657.g1	GE273434
61917390	CBWT3658.b1	GE273435
61917391	CBWT3658.g1	GE273436
61917392	CBWT3661.b1	GE273437
61917393	CBWT3661.g1	GE273438
61917394	CBWT3663.b1	GE273439
61917395	CBWT3663.g1	GE273440
61917396	CBWT3664.b1	GE273441
61917397	CBWT3664.g1	GE273442
61917398	CBWT3665.b1	GE273443
61917399	CBWT3665.g1	GE273444
61917400	CBWT3667.b1	GE273445
61917401	CBWT3667.g1	GE273446
61917402	CBWT3668.b1	GE273447
61917403	CBWT3669.b1	GE273448
61917404	CBWT3669.g1	GE273449
61917405	CBWT3670.b1	GE273450
61917406	CBWT3670.g1	GE273451
61917407	CBWT3672.b1	GE273452
61917408	CBWT3672.g1	GE273453
61917409	CBWT3673.b1	GE273454
61917410	CBWT3673.g1	GE273455
61917411	CBWT3675.b1	GE273456

61917412	CBWT3675.g1	GE273457
61917413	CBWT3676.b1	GE273458
61917414	CBWT3676.g1	GE273459
61917415	CBWT3678.b1	GE273460
61917416	CBWT3678.g1	GE273461
61917417	CBWT3680.b1	GE273462
61917418	CBWT3680.g1	GE273463
61917419	CBWT3681.b1	GE273464
61917420	CBWT3681.g1	GE273465
61917421	CBWT3682.g1	GE273466
61917422	CBWT3684.b1	GE273467
61917423	CBWT3687.b1	GE273468
61917424	CBWT3687.g1	GE273469
61917425	CBWT3690.b1	GE273470
61917426	CBWT3690.g1	GE273471
61917427	CBWT3694.b1	GE273472
61917428	CBWT3694.g1	GE273473
61917429	CBWT3696.b1	GE273474
61917430	CBWT3696.g1	GE273475
61917431	CBWT3697.b1	GE273476
61917432	CBWT3697.g1	GE273477
61917433	CBWT3699.b1	GE273478
61917434	CBWT3699.g1	GE273479
61917435	CBWT3700.g1	GE273480
61917436	CBWT3701.b1	GE273481
61917437	CBWT3701.g1	GE273482
61917438	CBWT3702.b1	GE273483
61917439	CBWT3702.g1	GE273484
61917440	CBWT3706.g1	GE273485
61917441	CBWT3707.g1	GE273486
61917442	CBWT3711.b1	GE273487
61917443	CBWT3712.b1	GE273488
61917444	CBWT3712.g1	GE273489
61917445	CBWT3715.g1	GE273490
61917446	CBWT3716.b1	GE273491
61917447	CBWT3716.g1	GE273492
61917448	CBWT3717.b1	GE273493
61917449	CBWT3717.g1	GE273494
61917450	CBWT3718.b1	GE273495
61917451	CBWT3718.g1	GE273496
61917452	CBWT3720.b1	GE273497
61917453	CBWT3720.g1	GE273498
61917454	CBWT3721.b1	GE273499
61917455	CBWT3721.g1	GE273500
61917456	CBWT3723.b1	GE273501
61917457	CBWT3723.g1	GE273502
61917458	CBWT3724.b1	GE273503
61917459	CBWT3724.g1	GE273504
61917460	CBWT3726.b1	GE273505
61917461	CBWT3726.g1	GE273506
61917462	CBWT3727.b1	GE273507
61917463	CBWT3727.g1	GE273508
61917464	CBWT3728.b1	GE273509
61917465	CBWT3728.g1	GE273510
61917466	CBWT3729.b1	GE273511
61917467	CBWT3729.g1	GE273512
61917468	CBWT3731.b1	GE273513
61917469	CBWT3731.g1	GE273514
61917470	CBWT3732.b1	GE273515
61917471	CBWT3732.g1	GE273516
61917472	CBWT3733.g1	GE273517
61917473	CBWT3734.b1	GE273518
61917474	CBWT3734.g1	GE273519

61917475	CBWT3735.b1	GE273520
61917476	CBWT3735.g1	GE273521
61917477	CBWT3736.b1	GE273522
61917478	CBWT3736.g1	GE273523
61917479	CBWT3737.g1	GE273524
61917480	CBWT3738.b1	GE273525
61917481	CBWT3738.g1	GE273526
61917482	CBWT3739.b1	GE273527
61917483	CBWT3739.g1	GE273528
61917484	CBWT3740.b1	GE273529
61917485	CBWT3742.b1	GE273530
61917486	CBWT3742.g1	GE273531
61917487	CBWT3743.g1	GE273532
61917488	CBWT3745.b1	GE273533
61917489	CBWT3745.g1	GE273534
61917490	CBWT3748.b1	GE273535
61917491	CBWT3748.g1	GE273536
61917492	CBWT3750.b1	GE273537
61917493	CBWT3750.g1	GE273538
61917494	CBWT3751.b1	GE273539
61917495	CBWT3751.g1	GE273540
61917496	CBWT3754.b1	GE273541
61917497	CBWT3755.b1	GE273542
61917498	CBWT3755.g1	GE273543
61917499	CBWT3756.g1	GE273544
61917500	CBWT3758.b1	GE273545
61917501	CBWT3758.g1	GE273546
61917502	CBWT3760.g1	GE273547
61917503	CBWT3761.b1	GE273548
61917504	CBWT3761.g1	GE273549
61917505	CBWT3763.b1	GE273550
61917506	CBWT3763.g1	GE273551
61917507	CBWT3765.b1	GE273552
61917508	CBWT3766.b1	GE273553
61917509	CBWT3766.g1	GE273554
61917510	CBWT3768.b1	GE273555
61917511	CBWT3768.g1	GE273556
61917512	CBWT3769.b1	GE273557
61917513	CBWT3769.g1	GE273558
61917514	CBWT3770.b1	GE273559
61917515	CBWT3770.g1	GE273560
61917516	CBWT3772.b1	GE273561
61917517	CBWT3774.b1	GE273562
61917518	CBWT3774.g1	GE273563
61917519	CBWT3776.b1	GE273564
61917520	CBWT3776.g1	GE273565
61917521	CBWT3780.b1	GE273566
61917522	CBWT3780.g1	GE273567
61917523	CBWT3781.g1	GE273568
61917524	CBWT3782.g1	GE273569
61917525	CBWT3783.b1	GE273570
61917526	CBWT3783.g1	GE273571
61917527	CBWT3784.b1	GE273572
61917528	CBWT3784.g1	GE273573
61917529	CBWT3787.b1	GE273574
61917530	CBWT3787.g1	GE273575
61917531	CBWT3788.b1	GE273576
61917532	CBWT3791.g1	GE273577
61917533	CBWT3792.b1	GE273578
61917534	CBWT3794.b1	GE273579
61917535	CBWT3794.g1	GE273580
61917536	CBWT3797.b1	GE273581
61917537	CBWT3799.g1	GE273582

61917538	CBWT3804.b1	GE273583
61917539	CBWT3804.g1	GE273584
61917540	CBWT3805.b1	GE273585
61917541	CBWT3805.g1	GE273586
61917542	CBWT3806.b1	GE273587
61917543	CBWT3806.g1	GE273588
61917544	CBWT3807.b1	GE273589
61917545	CBWT3807.g1	GE273590
61917546	CBWT3809.b1	GE273591
61917547	CBWT3809.g1	GE273592
61917548	CBWT3810.b1	GE273593
61917549	CBWT3810.g1	GE273594
61917550	CBWT3811.b1	GE273595
61917551	CBWT3812.b1	GE273596
61917552	CBWT3812.g1	GE273597
61917553	CBWT3816.b1	GE273598
61917554	CBWT3816.g1	GE273599
61917555	CBWT3817.g1	GE273600
61917556	CBWT3818.b1	GE273601
61917557	CBWT3818.g1	GE273602
61917558	CBWT3819.b1	GE273603
61917559	CBWT3821.b1	GE273604
61917560	CBWT3821.g1	GE273605
61917561	CBWT3822.b1	GE273606
61917562	CBWT3822.g1	GE273607
61917563	CBWT3824.b1	GE273608
61917564	CBWT3824.g1	GE273609
61917565	CBWT3825.b1	GE273610
61917566	CBWT3825.g1	GE273611
61917567	CBWT3826.b1	GE273612
61917568	CBWT3826.g1	GE273613
61917569	CBWT3831.b1	GE273614
61917570	CBWT3831.g1	GE273615
61917571	CBWT3832.b1	GE273616
61917572	CBWT3832.g1	GE273617
61917573	CBWT3834.b1	GE273618
61917574	CBWT3834.g1	GE273619
61917575	CBWT3835.b1	GE273620
61917576	CBWT3835.g1	GE273621
61917577	CBWT3836.b1	GE273622
61917578	CBWT3836.g1	GE273623
61917579	CBWT3837.b1	GE273624
61917580	CBWT3837.g1	GE273625
61917581	CBWT3839.b1	GE273626
61917582	CBWT3844.b1	GE273627
61917583	CBWT3844.g1	GE273628
61917584	CBWT3846.b1	GE273629
61917585	CBWT3846.g1	GE273630
61917586	CBWT3847.b1	GE273631
61917587	CBWT3847.g1	GE273632
61917588	CBWT3848.b1	GE273633
61917589	CBWT3848.g1	GE273634
61917590	CBWT3849.g1	GE273635
61917591	CBWT3851.b1	GE273636
61917592	CBWT3851.g1	GE273637
61917593	CBWT3852.b1	GE273638
61917594	CBWT3852.g1	GE273639
61917595	CBWT3855.g1	GE273640
61917596	CBWT3856.b1	GE273641
61917597	CBWT3856.g1	GE273642
61917598	CBWT3857.g1	GE273643
61917599	CBWT3860.b1	GE273644
61917600	CBWT3860.g1	GE273645

61917601	CBWT3861.b1	GE273646
61917602	CBWT3861.g1	GE273647
61917603	CBWT3862.g1	GE273648
61917604	CBWT3864.b1	GE273649
61917605	CBWT3864.g1	GE273650
61917606	CBWT3865.b1	GE273651
61917607	CBWT3865.g1	GE273652
61917608	CBWT3868.b1	GE273653
61917609	CBWT3868.g1	GE273654
61917610	CBWT3869.b1	GE273655
61917611	CBWT3869.g1	GE273656
61917612	CBWT387.b1	GE273657
61917613	CBWT387.g1	GE273658
61917614	CBWT3871.b1	GE273659
61917615	CBWT3871.g1	GE273660
61917616	CBWT3872.b1	GE273661
61917617	CBWT3872.g1	GE273662
61917618	CBWT3873.b1	GE273663
61917619	CBWT3873.g1	GE273664
61917620	CBWT3874.b1	GE273665
61917621	CBWT3874.g1	GE273666
61917622	CBWT3875.b1	GE273667
61917623	CBWT3875.g1	GE273668
61917624	CBWT3876.b1	GE273669
61917625	CBWT3876.g1	GE273670
61917626	CBWT3877.b1	GE273671
61917627	CBWT3877.g1	GE273672
61917628	CBWT3878.b1	GE273673
61917629	CBWT3878.g1	GE273674
61917630	CBWT3880.b1	GE273675
61917631	CBWT3881.b1	GE273676
61917632	CBWT3881.g1	GE273677
61917633	CBWT3882.b1	GE273678
61917634	CBWT3882.g1	GE273679
61917635	CBWT3885.b1	GE273680
61917636	CBWT3885.g1	GE273681
61917637	CBWT3888.b1	GE273682
61917638	CBWT3888.g1	GE273683
61917639	CBWT3890.b1	GE273684
61917640	CBWT3890.g1	GE273685
61917641	CBWT3891.b1	GE273686
61917642	CBWT3891.g1	GE273687
61917643	CBWT3892.b1	GE273688
61917644	CBWT3892.g1	GE273689
61917645	CBWT3893.b1	GE273690
61917646	CBWT3893.g1	GE273691
61917647	CBWT3896.b1	GE273692
61917648	CBWT3896.g1	GE273693
61917649	CBWT3897.b1	GE273694
61917650	CBWT3897.g1	GE273695
61917651	CBWT3898.b1	GE273696
61917652	CBWT3898.g1	GE273697
61917653	CBWT3899.b1	GE273698
61917654	CBWT3899.g1	GE273699
61917655	CBWT390.b6	GE273700
61917656	CBWT390.g11	GE273701
61917657	CBWT3902.b1	GE273702
61917658	CBWT3902.g1	GE273703
61917659	CBWT3903.b1	GE273704
61917660	CBWT3903.g1	GE273705
61917661	CBWT3904.b1	GE273706
61917662	CBWT3904.g1	GE273707
61917663	CBWT3905.b1	GE273708

61917664	CBWT3906.b1	GE273709
61917665	CBWT3906.g1	GE273710
61917666	CBWT3907.b1	GE273711
61917667	CBWT3907.g1	GE273712
61917668	CBWT3908.b1	GE273713
61917669	CBWT3908.g1	GE273714
61917670	CBWT3910.b1	GE273715
61917671	CBWT3910.g1	GE273716
61917672	CBWT3911.b1	GE273717
61917673	CBWT3911.g1	GE273718
61917674	CBWT3912.b1	GE273719
61917675	CBWT3912.g1	GE273720
61917676	CBWT3913.b1	GE273721
61917677	CBWT3913.g1	GE273722
61917678	CBWT3914.b1	GE273723
61917679	CBWT3914.g1	GE273724
61917680	CBWT3915.g1	GE273725
61917681	CBWT3917.b1	GE273726
61917682	CBWT3920.b1	GE273727
61917683	CBWT3920.g1	GE273728
61917684	CBWT3921.b1	GE273729
61917685	CBWT3921.g1	GE273730
61917686	CBWT3923.g1	GE273731
61917687	CBWT3924.b1	GE273732
61917688	CBWT3924.g1	GE273733
61917689	CBWT3926.b1	GE273734
61917690	CBWT3926.g1	GE273735
61917691	CBWT3927.b1	GE273736
61917692	CBWT3927.g1	GE273737
61917693	CBWT3929.b1	GE273738
61917694	CBWT3929.g1	GE273739
61917695	CBWT3930.g1	GE273740
61917696	CBWT3931.b1	GE273741
61917697	CBWT3931.g1	GE273742
61917698	CBWT3932.b1	GE273743
61917699	CBWT3933.b1	GE273744
61917700	CBWT3936.b1	GE273745
61917701	CBWT3936.g1	GE273746
61917702	CBWT3937.b1	GE273747
61917703	CBWT3937.g1	GE273748
61917704	CBWT3938.b1	GE273749
61917705	CBWT3938.g1	GE273750
61917706	CBWT3940.b1	GE273751
61917707	CBWT3940.g1	GE273752
61917708	CBWT3941.b1	GE273753
61917709	CBWT3942.b1	GE273754
61917710	CBWT3942.g1	GE273755
61917711	CBWT3943.b1	GE273756
61917712	CBWT3943.g1	GE273757
61917713	CBWT3945.b1	GE273758
61917714	CBWT3945.g1	GE273759
61917715	CBWT3946.b1	GE273760
61917716	CBWT3946.g1	GE273761
61917717	CBWT3947.b1	GE273762
61917718	CBWT3947.g1	GE273763
61917719	CBWT3948.b1	GE273764
61917720	CBWT3948.g1	GE273765
61917721	CBWT3949.b1	GE273766
61917722	CBWT3949.g1	GE273767
61917723	CBWT3950.b1	GE273768
61917724	CBWT3950.g1	GE273769
61917725	CBWT3951.b1	GE273770
61917726	CBWT3951.g1	GE273771

61917727	CBWT3956.b1	GE273772
61917728	CBWT3956.g1	GE273773
61917729	CBWT3957.b1	GE273774
61917730	CBWT3957.g1	GE273775
61917731	CBWT3958.b1	GE273776
61917732	CBWT3958.g1	GE273777
61917733	CBWT3959.b1	GE273778
61917734	CBWT3959.g1	GE273779
61917735	CBWT3962.b1	GE273780
61917736	CBWT3962.g1	GE273781
61917737	CBWT3963.b1	GE273782
61917738	CBWT3963.g1	GE273783
61917739	CBWT3964.b1	GE273784
61917740	CBWT3964.g1	GE273785
61917741	CBWT3968.b1	GE273786
61917742	CBWT3968.g1	GE273787
61917743	CBWT3970.b1	GE273788
61917744	CBWT3970.g1	GE273789
61917745	CBWT3972.b1	GE273790
61917746	CBWT3972.g1	GE273791
61917747	CBWT3973.b1	GE273792
61917748	CBWT3973.g1	GE273793
61917749	CBWT3974.b1	GE273794
61917750	CBWT3974.g1	GE273795
61917751	CBWT3975.b1	GE273796
61917752	CBWT3975.g1	GE273797
61917753	CBWT3976.b1	GE273798
61917754	CBWT3976.g1	GE273799
61917755	CBWT3977.b1	GE273800
61917756	CBWT3977.g1	GE273801
61917757	CBWT3978.b1	GE273802
61917758	CBWT3978.g1	GE273803
61917759	CBWT3980.b1	GE273804
61917760	CBWT3980.g1	GE273805
61917761	CBWT3982.b1	GE273806
61917762	CBWT3982.g1	GE273807
61917763	CBWT3983.b1	GE273808
61917764	CBWT3983.g1	GE273809
61917765	CBWT3984.g1	GE273810
61917766	CBWT3985.b1	GE273811
61917767	CBWT3985.g1	GE273812
61917768	CBWT3986.b1	GE273813
61917769	CBWT3986.g1	GE273814
61917770	CBWT3987.g1	GE273815
61917771	CBWT3990.b1	GE273816
61917772	CBWT3990.g1	GE273817
61917773	CBWT3992.b1	GE273818
61917774	CBWT3992.g1	GE273819
61917775	CBWT3993.b1	GE273820
61917776	CBWT3993.g1	GE273821
61917777	CBWT3994.b1	GE273822
61917778	CBWT3994.g1	GE273823
61917779	CBWT3995.b1	GE273824
61917780	CBWT3995.g1	GE273825
61917781	CBWT3998.b1	GE273826
61917782	CBWT3999.b1	GE273827
61917783	CBWT3999.g1	GE273828
61917784	CBWT4002.b1	GE273829
61917785	CBWT4002.g1	GE273830
61917786	CBWT4003.b1	GE273831
61917787	CBWT4003.g1	GE273832
61917788	CBWT4005.b1	GE273833
61917789	CBWT4005.g1	GE273834

61917790	CBWT4006.b1	GE273835
61917791	CBWT4006.g1	GE273836
61917792	CBWT4007.b1	GE273837
61917793	CBWT4007.g1	GE273838
61917794	CBWT4008.b1	GE273839
61917795	CBWT4008.g1	GE273840
61917796	CBWT4009.b1	GE273841
61917797	CBWT4009.g1	GE273842
61917798	CBWT4010.b1	GE273843
61917799	CBWT4010.g1	GE273844
61917800	CBWT4012.b1	GE273845
61917801	CBWT4012.g1	GE273846
61917802	CBWT4014.b1	GE273847
61917803	CBWT4014.g1	GE273848
61917804	CBWT4015.b1	GE273849
61917805	CBWT4015.g1	GE273850
61917806	CBWT4018.b1	GE273851
61917807	CBWT4018.g1	GE273852
61917808	CBWT4019.b1	GE273853
61917809	CBWT4019.g1	GE273854
61917810	CBWT4021.b1	GE273855
61917811	CBWT4021.g1	GE273856
61917812	CBWT4022.g1	GE273857
61917813	CBWT4024.b1	GE273858
61917814	CBWT4024.g1	GE273859
61917815	CBWT4025.b1	GE273860
61917816	CBWT4025.g1	GE273861
61917817	CBWT4028.b1	GE273862
61917818	CBWT4028.g1	GE273863
61917819	CBWT4032.b1	GE273864
61917820	CBWT4032.g1	GE273865
61917821	CBWT4033.b1	GE273866
61917822	CBWT4033.g1	GE273867
61917823	CBWT4034.b1	GE273868
61917824	CBWT4034.g1	GE273869
61917825	CBWT4036.b1	GE273870
61917826	CBWT4036.g1	GE273871
61917827	CBWT4037.b1	GE273872
61917828	CBWT4037.g1	GE273873
61917829	CBWT4038.b1	GE273874
61917830	CBWT4038.g1	GE273875
61917831	CBWT4041.b1	GE273876
61917832	CBWT4041.g1	GE273877
61917833	CBWT4042.b1	GE273878
61917834	CBWT4042.g1	GE273879
61917835	CBWT4043.b1	GE273880
61917836	CBWT4043.g1	GE273881
61917837	CBWT4044.b1	GE273882
61917838	CBWT4044.g1	GE273883
61917839	CBWT4045.b1	GE273884
61917840	CBWT4045.g1	GE273885
61917841	CBWT4046.b1	GE273886
61917842	CBWT4046.g1	GE273887
61917843	CBWT4047.b1	GE273888
61917844	CBWT4047.g1	GE273889
61917845	CBWT4048.g1	GE273890
61917846	CBWT4049.g1	GE273891
61917847	CBWT405.b11	GE273892
61917848	CBWT4050.b1	GE273893
61917849	CBWT4050.g1	GE273894
61917850	CBWT4053.b1	GE273895
61917851	CBWT4054.b1	GE273896
61917852	CBWT4054.g1	GE273897

61917853	CBWT4055.b1	GE273898
61917854	CBWT4055.g1	GE273899
61917855	CBWT4057.b1	GE273900
61917856	CBWT4057.g1	GE273901
61917857	CBWT4058.b1	GE273902
61917858	CBWT4058.g1	GE273903
61917859	CBWT4059.b1	GE273904
61917860	CBWT4059.g1	GE273905
61917861	CBWT406.b1	GE273906
61917862	CBWT406.g6	GE273907
61917863	CBWT4060.b1	GE273908
61917864	CBWT4060.g1	GE273909
61917865	CBWT4061.b1	GE273910
61917866	CBWT4061.g1	GE273911
61917867	CBWT4062.b1	GE273912
61917868	CBWT4062.g1	GE273913
61917869	CBWT4063.b1	GE273914
61917870	CBWT4063.g1	GE273915
61917871	CBWT4065.b1	GE273916
61917872	CBWT4065.g1	GE273917
61917873	CBWT4066.g1	GE273918
61917874	CBWT4067.b1	GE273919
61917875	CBWT4067.g1	GE273920
61917876	CBWT4068.b1	GE273921
61917877	CBWT4068.g1	GE273922
61917878	CBWT4069.b1	GE273923
61917879	CBWT4069.g1	GE273924
61917880	CBWT4070.b1	GE273925
61917881	CBWT4070.g1	GE273926
61917882	CBWT4073.b1	GE273927
61917883	CBWT4073.g1	GE273928
61917884	CBWT4074.b1	GE273929
61917885	CBWT4074.g1	GE273930
61917886	CBWT4076.b1	GE273931
61917887	CBWT4076.g1	GE273932
61917888	CBWT4077.b1	GE273933
61917889	CBWT4077.g1	GE273934
61917890	CBWT4079.b1	GE273935
61917891	CBWT4079.g1	GE273936
61917892	CBWT4081.b1	GE273937
61917893	CBWT4082.b1	GE273938
61917894	CBWT4082.g1	GE273939
61917895	CBWT4083.b1	GE273940
61917896	CBWT4083.g1	GE273941
61917897	CBWT4086.b1	GE273942
61917898	CBWT4086.g1	GE273943
61917899	CBWT4087.b1	GE273944
61917900	CBWT4087.g1	GE273945
61917901	CBWT4088.b1	GE273946
61917902	CBWT4088.g1	GE273947
61917903	CBWT4090.b1	GE273948
61917904	CBWT4090.g1	GE273949
61917905	CBWT4091.b1	GE273950
61917906	CBWT4092.b1	GE273951
61917907	CBWT4092.g1	GE273952
61917908	CBWT4093.b1	GE273953
61917909	CBWT4094.b1	GE273954
61917910	CBWT4095.b1	GE273955
61917911	CBWT4095.g1	GE273956
61917912	CBWT4096.b1	GE273957
61917913	CBWT4096.g1	GE273958
61917914	CBWT4097.g1	GE273959
61917915	CBWT4098.b1	GE273960

61917916	CBWT4098.g1	GE273961
61917917	CBWT4099.g1	GE273962
61917918	CBWT410.b11	GE273963
61917919	CBWT410.g11	GE273964
61917920	CBWT4100.b1	GE273965
61917921	CBWT4102.b1	GE273966
61917922	CBWT4102.g1	GE273967
61917923	CBWT4103.b1	GE273968
61917924	CBWT4103.g1	GE273969
61917925	CBWT4104.b1	GE273970
61917926	CBWT4104.g1	GE273971
61917927	CBWT4107.b1	GE273972
61917928	CBWT4108.b1	GE273973
61917929	CBWT4108.g1	GE273974
61917930	CBWT4110.b1	GE273975
61917931	CBWT4110.g1	GE273976
61917932	CBWT4111.b1	GE273977
61917933	CBWT4111.g1	GE273978
61917934	CBWT4112.b1	GE273979
61917935	CBWT4112.g1	GE273980
61917936	CBWT4113.b1	GE273981
61917937	CBWT4113.g1	GE273982
61917938	CBWT4115.b1	GE273983
61917939	CBWT4115.g1	GE273984
61917940	CBWT4116.b1	GE273985
61917941	CBWT4116.g1	GE273986
61917942	CBWT4118.g1	GE273987
61917943	CBWT4128.b1	GE273988
61917944	CBWT4128.g1	GE273989
61917945	CBWT4129.b1	GE273990
61917946	CBWT4129.g1	GE273991
61917947	CBWT413.b1	GE273992
61917948	CBWT413.g11	GE273993
61917949	CBWT4132.b1	GE273994
61917950	CBWT4132.g1	GE273995
61917951	CBWT4134.b1	GE273996
61917952	CBWT4134.g1	GE273997
61917953	CBWT4135.b1	GE273998
61917954	CBWT4135.g1	GE273999
61917955	CBWT4137.b1	GE274000
61917956	CBWT4137.g1	GE274001
61917957	CBWT4138.b1	GE274002
61917958	CBWT4138.g1	GE274003
61917959	CBWT4139.b1	GE274004
61917960	CBWT4139.g1	GE274005
61917961	CBWT4140.b1	GE274006
61917962	CBWT4140.g1	GE274007
61917963	CBWT4141.b1	GE274008
61917964	CBWT4141.g1	GE274009
61917965	CBWT4142.b1	GE274010
61917966	CBWT4142.g1	GE274011
61917967	CBWT4144.b1	GE274012
61917968	CBWT4144.g1	GE274013
61917969	CBWT4145.b1	GE274014
61917970	CBWT4146.b1	GE274015
61917971	CBWT4146.g1	GE274016
61917972	CBWT4147.b1	GE274017
61917973	CBWT4147.g1	GE274018
61917974	CBWT4148.b1	GE274019
61917975	CBWT4148.g1	GE274020
61917976	CBWT4150.b1	GE274021
61917977	CBWT4150.g1	GE274022
61917978	CBWT4151.b1	GE274023

61917979	CBWT4151.g1	GE274024
61917980	CBWT4154.g1	GE274025
61917981	CBWT4155.b1	GE274026
61917982	CBWT4155.g1	GE274027
61917983	CBWT4157.b1	GE274028
61917984	CBWT4157.g1	GE274029
61917985	CBWT4158.b1	GE274030
61917986	CBWT4158.g1	GE274031
61917987	CBWT4161.b1	GE274032
61917988	CBWT4161.g1	GE274033
61917989	CBWT4162.b1	GE274034
61917990	CBWT4162.g1	GE274035
61917991	CBWT4163.b1	GE274036
61917992	CBWT4163.g1	GE274037
61917993	CBWT4164.b1	GE274038
61917994	CBWT4164.g1	GE274039
61917995	CBWT4167.b1	GE274040
61917996	CBWT4167.g1	GE274041
61917997	CBWT4168.g1	GE274042
61917998	CBWT4169.b1	GE274043
61917999	CBWT4169.g1	GE274044
61918000	CBWT4171.b1	GE274045
61918001	CBWT4171.g1	GE274046
61918002	CBWT4173.b1	GE274047
61918003	CBWT4173.g1	GE274048
61918004	CBWT4174.b1	GE274049
61918005	CBWT4174.g1	GE274050
61918006	CBWT4176.b1	GE274051
61918007	CBWT4176.g1	GE274052
61918008	CBWT4178.b1	GE274053
61918009	CBWT4178.g1	GE274054
61918010	CBWT4179.b1	GE274055
61918011	CBWT4179.g1	GE274056
61918012	CBWT418.b1	GE274057
61918013	CBWT418.g11	GE274058
61918014	CBWT4180.b1	GE274059
61918015	CBWT4182.b1	GE274060
61918016	CBWT4182.g1	GE274061
61918017	CBWT4183.b1	GE274062
61918018	CBWT4183.g1	GE274063
61918019	CBWT4185.b1	GE274064
61918020	CBWT4186.b1	GE274065
61918021	CBWT4186.g1	GE274066
61918022	CBWT4187.b1	GE274067
61918023	CBWT4187.g1	GE274068
61918024	CBWT4188.b1	GE274069
61918025	CBWT4188.g1	GE274070
61918026	CBWT4189.b1	GE274071
61918027	CBWT4189.g1	GE274072
61918028	CBWT419.b11	GE274073
61918029	CBWT419.g11	GE274074
61918030	CBWT4190.b1	GE274075
61918031	CBWT4190.g1	GE274076
61918032	CBWT4191.b1	GE274077
61918033	CBWT4191.g1	GE274078
61918034	CBWT4193.b1	GE274079
61918035	CBWT4193.g1	GE274080
61918036	CBWT4195.b1	GE274081
61918037	CBWT4196.b1	GE274082
61918038	CBWT4199.b1	GE274083
61918039	CBWT4199.g1	GE274084
61918040	CBWT420.b11	GE274085
61918041	CBWT420.g6	GE274086

61918042	CBWT4200.b1	GE274087
61918043	CBWT4200.g1	GE274088
61918044	CBWT4201.b1	GE274089
61918045	CBWT4201.g1	GE274090
61918046	CBWT4203.b1	GE274091
61918047	CBWT4203.g1	GE274092
61918048	CBWT4204.b1	GE274093
61918049	CBWT4204.g1	GE274094
61918050	CBWT4205.g1	GE274095
61918051	CBWT4206.b1	GE274096
61918052	CBWT4206.g1	GE274097
61918053	CBWT4207.b1	GE274098
61918054	CBWT4207.g1	GE274099
61918055	CBWT4208.b1	GE274100
61918056	CBWT4208.g1	GE274101
61918057	CBWT4209.b1	GE274102
61918058	CBWT4209.g1	GE274103
61918059	CBWT4211.b1	GE274104
61918060	CBWT4211.g1	GE274105
61918061	CBWT4212.b1	GE274106
61918062	CBWT4212.g1	GE274107
61918063	CBWT4213.b1	GE274108
61918064	CBWT4213.g1	GE274109
61918065	CBWT4214.b1	GE274110
61918066	CBWT4214.g1	GE274111
61918067	CBWT4215.b1	GE274112
61918068	CBWT4215.g1	GE274113
61918069	CBWT4216.b1	GE274114
61918070	CBWT4216.g1	GE274115
61918071	CBWT4217.b1	GE274116
61918072	CBWT4224.g1	GE274117
61918073	CBWT4228.b1	GE274118
61918074	CBWT4228.g1	GE274119
61918075	CBWT4229.b1	GE274120
61918076	CBWT4229.g1	GE274121
61918077	CBWT4230.g1	GE274122
61918078	CBWT4231.b1	GE274123
61918079	CBWT4231.g1	GE274124
61918080	CBWT4233.b1	GE274125
61918081	CBWT4233.g1	GE274126
61918082	CBWT4234.b1	GE274127
61918083	CBWT4234.g1	GE274128
61918084	CBWT4235.b1	GE274129
61918085	CBWT4236.b1	GE274130
61918086	CBWT4236.g1	GE274131
61918087	CBWT4237.b1	GE274132
61918088	CBWT4237.g1	GE274133
61918089	CBWT4238.b1	GE274134
61918090	CBWT4238.g1	GE274135
61918091	CBWT424.b11	GE274136
61918092	CBWT4240.b1	GE274137
61918093	CBWT4240.g1	GE274138
61918094	CBWT4241.b1	GE274139
61918095	CBWT4241.g1	GE274140
61918096	CBWT4242.g1	GE274141
61918097	CBWT4243.b1	GE274142
61918098	CBWT4243.g1	GE274143
61918099	CBWT4244.b1	GE274144
61918100	CBWT4245.b1	GE274145
61918101	CBWT4245.g1	GE274146
61918102	CBWT4246.b1	GE274147
61918103	CBWT4246.g1	GE274148
61918104	CBWT4249.b1	GE274149

61918105	CBWT4249.g1	GE274150
61918106	CBWT4250.b1	GE274151
61918107	CBWT4250.g1	GE274152
61918108	CBWT4253.b1	GE274153
61918109	CBWT4253.g1	GE274154
61918110	CBWT4254.b1	GE274155
61918111	CBWT4254.g1	GE274156
61918112	CBWT4256.b1	GE274157
61918113	CBWT4256.g1	GE274158
61918114	CBWT426.b11	GE274159
61918115	CBWT426.g11	GE274160
61918116	CBWT4261.b1	GE274161
61918117	CBWT4261.g1	GE274162
61918118	CBWT4262.b1	GE274163
61918119	CBWT4262.g1	GE274164
61918120	CBWT4265.b1	GE274165
61918121	CBWT4265.g1	GE274166
61918122	CBWT4266.b1	GE274167
61918123	CBWT4267.b1	GE274168
61918124	CBWT4267.g1	GE274169
61918125	CBWT4268.b1	GE274170
61918126	CBWT4268.g1	GE274171
61918127	CBWT4271.b1	GE274172
61918128	CBWT4271.g1	GE274173
61918129	CBWT4272.b1	GE274174
61918130	CBWT4272.g1	GE274175
61918131	CBWT4273.b1	GE274176
61918132	CBWT4273.g1	GE274177
61918133	CBWT4274.b1	GE274178
61918134	CBWT4274.g1	GE274179
61918135	CBWT4275.b1	GE274180
61918136	CBWT4277.b1	GE274181
61918137	CBWT4277.g1	GE274182
61918138	CBWT4278.b1	GE274183
61918139	CBWT4278.g1	GE274184
61918140	CBWT4280.b1	GE274185
61918141	CBWT4280.g1	GE274186
61918142	CBWT4281.b1	GE274187
61918143	CBWT4281.g1	GE274188
61918144	CBWT4282.b1	GE274189
61918145	CBWT4282.g1	GE274190
61918146	CBWT4283.b1	GE274191
61918147	CBWT4285.b1	GE274192
61918148	CBWT4285.g1	GE274193
61918149	CBWT4286.b1	GE274194
61918150	CBWT4286.g1	GE274195
61918151	CBWT4287.g1	GE274196
61918152	CBWT4288.b1	GE274197
61918153	CBWT4288.g1	GE274198
61918154	CBWT4289.b1	GE274199
61918155	CBWT4289.g1	GE274200
61918156	CBWT4290.b1	GE274201
61918157	CBWT4291.b1	GE274202
61918158	CBWT4293.b1	GE274203
61918159	CBWT4293.g1	GE274204
61918160	CBWT4294.b1	GE274205
61918161	CBWT4294.g1	GE274206
61918162	CBWT4295.b1	GE274207
61918163	CBWT4295.g1	GE274208
61918164	CBWT4296.b1	GE274209
61918165	CBWT4296.g1	GE274210
61918166	CBWT4297.b1	GE274211
61918167	CBWT4297.g1	GE274212

61918168	CBWT4299.b1	GE274213
61918169	CBWT4299.g1	GE274214
61918170	CBWT4302.b1	GE274215
61918171	CBWT4302.g1	GE274216
61918172	CBWT4303.b1	GE274217
61918173	CBWT4303.g1	GE274218
61918174	CBWT4304.b1	GE274219
61918175	CBWT4304.g1	GE274220
61918176	CBWT4306.g1	GE274221
61918177	CBWT4307.b1	GE274222
61918178	CBWT4307.g1	GE274223
61918179	CBWT4308.b1	GE274224
61918180	CBWT4308.g1	GE274225
61918181	CBWT431.b11	GE274226
61918182	CBWT431.g11	GE274227
61918183	CBWT4310.b1	GE274228
61918184	CBWT4310.g1	GE274229
61918185	CBWT4311.b1	GE274230
61918186	CBWT4311.g1	GE274231
61918187	CBWT4312.b1	GE274232
61918188	CBWT4312.g1	GE274233
61918189	CBWT4314.b1	GE274234
61918190	CBWT4317.b1	GE274235
61918191	CBWT4317.g1	GE274236
61918192	CBWT4318.b1	GE274237
61918193	CBWT4318.g1	GE274238
61918194	CBWT432.b6	GE274239
61918195	CBWT432.g11	GE274240
61918196	CBWT4321.b1	GE274241
61918197	CBWT4321.g1	GE274242
61918198	CBWT4322.b1	GE274243
61918199	CBWT4323.b1	GE274244
61918200	CBWT4323.g1	GE274245
61918201	CBWT4324.b1	GE274246
61918202	CBWT4324.g1	GE274247
61918203	CBWT4328.g1	GE274248
61918204	CBWT4329.b1	GE274249
61918205	CBWT4330.g1	GE274250
61918206	CBWT4331.b1	GE274251
61918207	CBWT4331.g1	GE274252
61918208	CBWT4332.b1	GE274253
61918209	CBWT4332.g1	GE274254
61918210	CBWT4334.b1	GE274255
61918211	CBWT4334.g1	GE274256
61918212	CBWT4335.b1	GE274257
61918213	CBWT4336.b1	GE274258
61918214	CBWT4336.g1	GE274259
61918215	CBWT4339.b1	GE274260
61918216	CBWT4339.g1	GE274261
61918217	CBWT4340.b1	GE274262
61918218	CBWT4340.g1	GE274263
61918219	CBWT4341.b1	GE274264
61918220	CBWT4341.g1	GE274265
61918221	CBWT4342.g1	GE274266
61918222	CBWT4344.b1	GE274267
61918223	CBWT4344.g1	GE274268
61918224	CBWT4345.b1	GE274269
61918225	CBWT4345.g1	GE274270
61918226	CBWT4346.b1	GE274271
61918227	CBWT4346.g1	GE274272
61918228	CBWT4347.b1	GE274273
61918229	CBWT4347.g1	GE274274
61918230	CBWT4351.b1	GE274275

61918231	CBWT4351.g1	GE274276
61918232	CBWT4352.b1	GE274277
61918233	CBWT4352.g1	GE274278
61918234	CBWT4353.b1	GE274279
61918235	CBWT4353.g1	GE274280
61918236	CBWT4355.b1	GE274281
61918237	CBWT4355.g1	GE274282
61918238	CBWT4358.b1	GE274283
61918239	CBWT4358.g1	GE274284
61918240	CBWT4359.g1	GE274285
61918241	CBWT4360.g1	GE274286
61918242	CBWT4361.b1	GE274287
61918243	CBWT4361.g1	GE274288
61918244	CBWT4362.b1	GE274289
61918245	CBWT4362.g1	GE274290
61918246	CBWT4363.b1	GE274291
61918247	CBWT4363.g1	GE274292
61918248	CBWT4364.g1	GE274293
61918249	CBWT4367.b1	GE274294
61918250	CBWT4367.g1	GE274295
61918251	CBWT4368.b1	GE274296
61918252	CBWT4368.g1	GE274297
61918253	CBWT4369.b1	GE274298
61918254	CBWT4369.g1	GE274299
61918255	CBWT4370.b1	GE274300
61918256	CBWT4370.g1	GE274301
61918257	CBWT4371.b1	GE274302
61918258	CBWT4371.g1	GE274303
61918259	CBWT4372.g1	GE274304
61918260	CBWT4373.b1	GE274305
61918261	CBWT4373.g1	GE274306
61918262	CBWT4374.b1	GE274307
61918263	CBWT4374.g1	GE274308
61918264	CBWT4375.b1	GE274309
61918265	CBWT4375.g1	GE274310
61918266	CBWT4376.b1	GE274311
61918267	CBWT4377.b1	GE274312
61918268	CBWT4377.g1	GE274313
61918269	CBWT4379.b1	GE274314
61918270	CBWT4380.b1	GE274315
61918271	CBWT4380.g1	GE274316
61918272	CBWT4382.b1	GE274317
61918273	CBWT4382.g1	GE274318
61918274	CBWT4383.b1	GE274319
61918275	CBWT4384.b1	GE274320
61918276	CBWT4384.g1	GE274321
61918277	CBWT4386.b1	GE274322
61918278	CBWT4387.b1	GE274323
61918279	CBWT4387.g1	GE274324
61918280	CBWT439.b11	GE274325
61918281	CBWT439.g11	GE274326
61918282	CBWT4391.b1	GE274327
61918283	CBWT4392.b1	GE274328
61918284	CBWT4392.g1	GE274329
61918285	CBWT4394.b1	GE274330
61918286	CBWT4394.g1	GE274331
61918287	CBWT4395.b1	GE274332
61918288	CBWT4395.g1	GE274333
61918289	CBWT4396.b1	GE274334
61918290	CBWT4396.g1	GE274335
61918291	CBWT4397.b1	GE274336
61918292	CBWT4397.g1	GE274337
61918293	CBWT4399.b1	GE274338

61918294	CBWT4399.g1	GE274339
61918295	CBWT440.b11	GE274340
61918296	CBWT440.g11	GE274341
61918297	CBWT4401.b1	GE274342
61918298	CBWT4401.g1	GE274343
61918299	CBWT4402.b1	GE274344
61918300	CBWT4402.g1	GE274345
61918301	CBWT4403.b1	GE274346
61918302	CBWT4403.g1	GE274347
61918303	CBWT4409.b1	GE274348
61918304	CBWT4410.b1	GE274349
61918305	CBWT4410.g1	GE274350
61918306	CBWT4411.b1	GE274351
61918307	CBWT4411.g1	GE274352
61918308	CBWT4412.b1	GE274353
61918309	CBWT4412.g1	GE274354
61918310	CBWT4413.b1	GE274355
61918311	CBWT4413.g1	GE274356
61918312	CBWT4414.b1	GE274357
61918313	CBWT4414.g1	GE274358
61918314	CBWT4415.b1	GE274359
61918315	CBWT4415.g1	GE274360
61918316	CBWT4416.b1	GE274361
61918317	CBWT4416.g1	GE274362
61918318	CBWT4417.b1	GE274363
61918319	CBWT4417.g1	GE274364
61918320	CBWT4422.g1	GE274365
61918321	CBWT4424.b1	GE274366
61918322	CBWT4424.g1	GE274367
61918323	CBWT4426.b1	GE274368
61918324	CBWT4426.g1	GE274369
61918325	CBWT4428.b1	GE274370
61918326	CBWT4428.g1	GE274371
61918327	CBWT4429.b1	GE274372
61918328	CBWT4429.g1	GE274373
61918329	CBWT443.b11	GE274374
61918330	CBWT443.g11	GE274375
61918331	CBWT4431.g1	GE274376
61918332	CBWT4432.b1	GE274377
61918333	CBWT4432.g1	GE274378
61918334	CBWT4433.b1	GE274379
61918335	CBWT4433.g1	GE274380
61918336	CBWT4434.g1	GE274381
61918337	CBWT4435.b1	GE274382
61918338	CBWT4435.g1	GE274383
61918339	CBWT4436.b1	GE274384
61918340	CBWT4436.g1	GE274385
61918341	CBWT4438.b1	GE274386
61918342	CBWT4438.g1	GE274387
61918343	CBWT4439.b1	GE274388
61918344	CBWT4439.g1	GE274389
61918345	CBWT4440.b1	GE274390
61918346	CBWT4440.g1	GE274391
61918347	CBWT4441.b1	GE274392
61918348	CBWT4441.g1	GE274393
61918349	CBWT4443.b1	GE274394
61918350	CBWT4443.g1	GE274395
61918351	CBWT4445.b1	GE274396
61918352	CBWT4445.g1	GE274397
61918353	CBWT4446.b1	GE274398
61918354	CBWT4446.g1	GE274399
61918355	CBWT4447.b1	GE274400
61918356	CBWT4447.g1	GE274401

61918357	CBWT4448.b1	GE274402
61918358	CBWT4448.g1	GE274403
61918359	CBWT4450.b1	GE274404
61918360	CBWT4450.g1	GE274405
61918361	CBWT4451.b1	GE274406
61918362	CBWT4451.g1	GE274407
61918363	CBWT4452.b1	GE274408
61918364	CBWT4452.g1	GE274409
61918365	CBWT4456.b1	GE274410
61918366	CBWT4456.g1	GE274411
61918367	CBWT4457.b1	GE274412
61918368	CBWT4457.g1	GE274413
61918369	CBWT4458.b1	GE274414
61918370	CBWT4458.g1	GE274415
61918371	CBWT4459.b1	GE274416
61918372	CBWT4459.g1	GE274417
61918373	CBWT446.b11	GE274418
61918374	CBWT446.g11	GE274419
61918375	CBWT4463.b1	GE274420
61918376	CBWT4463.g1	GE274421
61918377	CBWT4464.b1	GE274422
61918378	CBWT4464.g1	GE274423
61918379	CBWT4465.b1	GE274424
61918380	CBWT4465.g1	GE274425
61918381	CBWT4466.b1	GE274426
61918382	CBWT4466.g1	GE274427
61918383	CBWT4468.b1	GE274428
61918384	CBWT4468.g1	GE274429
61918385	CBWT4469.b1	GE274430
61918386	CBWT4470.b1	GE274431
61918387	CBWT4470.g1	GE274432
61918388	CBWT4473.b1	GE274433
61918389	CBWT4473.g1	GE274434
61918390	CBWT4475.b1	GE274435
61918391	CBWT4475.g1	GE274436
61918392	CBWT4477.b1	GE274437
61918393	CBWT4478.b1	GE274438
61918394	CBWT4478.g1	GE274439
61918395	CBWT4480.b1	GE274440
61918396	CBWT4480.g1	GE274441
61918397	CBWT4481.b1	GE274442
61918398	CBWT4481.g1	GE274443
61918399	CBWT4482.b1	GE274444
61918400	CBWT4482.g1	GE274445
61918401	CBWT4483.b1	GE274446
61918402	CBWT4483.g1	GE274447
61918403	CBWT4485.b1	GE274448
61918404	CBWT4485.g1	GE274449
61918405	CBWT4486.b1	GE274450
61918406	CBWT4486.g1	GE274451
61918407	CBWT4488.b1	GE274452
61918408	CBWT4488.g1	GE274453
61918409	CBWT449.b11	GE274454
61918410	CBWT449.g11	GE274455
61918411	CBWT4490.b1	GE274456
61918412	CBWT4490.g1	GE274457
61918413	CBWT4491.b1	GE274458
61918414	CBWT4491.g1	GE274459
61918415	CBWT4493.b1	GE274460
61918416	CBWT4493.g1	GE274461
61918417	CBWT4494.b1	GE274462
61918418	CBWT4494.g1	GE274463
61918419	CBWT4495.b1	GE274464

61918420	CBWT4495.g1	GE274465
61918421	CBWT4496.b1	GE274466
61918422	CBWT4496.g1	GE274467
61918423	CBWT4497.g1	GE274468
61918424	CBWT4498.b1	GE274469
61918425	CBWT4499.b1	GE274470
61918426	CBWT4499.g1	GE274471
61918427	CBWT450.b11	GE274472
61918428	CBWT450.g11	GE274473
61918429	CBWT4500.b1	GE274474
61918430	CBWT4500.g1	GE274475
61918431	CBWT4501.b1	GE274476
61918432	CBWT4501.g1	GE274477
61918433	CBWT4502.b1	GE274478
61918434	CBWT4502.g1	GE274479
61918435	CBWT4503.b1	GE274480
61918436	CBWT4503.g1	GE274481
61918437	CBWT4505.b1	GE274482
61918438	CBWT4506.b1	GE274483
61918439	CBWT4506.g1	GE274484
61918440	CBWT4507.b1	GE274485
61918441	CBWT4507.g1	GE274486
61918442	CBWT4508.b1	GE274487
61918443	CBWT4508.g1	GE274488
61918444	CBWT4510.b1	GE274489
61918445	CBWT4510.g1	GE274490
61918446	CBWT4512.b1	GE274491
61918447	CBWT4514.b1	GE274492
61918448	CBWT4514.g1	GE274493
61918449	CBWT4515.b1	GE274494
61918450	CBWT4515.g1	GE274495
61918451	CBWT4516.b1	GE274496
61918452	CBWT4516.g1	GE274497
61918453	CBWT4519.b1	GE274498
61918454	CBWT452.b6	GE274499
61918455	CBWT452.g6	GE274500
61918456	CBWT4520.b1	GE274501
61918457	CBWT4520.g1	GE274502
61918458	CBWT4521.b1	GE274503
61918459	CBWT4521.g1	GE274504
61918460	CBWT4522.b1	GE274505
61918461	CBWT4522.g1	GE274506
61918462	CBWT4523.b1	GE274507
61918463	CBWT4523.g1	GE274508
61918464	CBWT4524.b1	GE274509
61918465	CBWT4524.g1	GE274510
61918466	CBWT4525.b1	GE274511
61918467	CBWT4525.g1	GE274512
61918468	CBWT4529.b1	GE274513
61918469	CBWT453.b6	GE274514
61918470	CBWT453.g6	GE274515
61918471	CBWT4530.b1	GE274516
61918472	CBWT4530.g1	GE274517
61918473	CBWT4532.g1	GE274518
61918474	CBWT4534.g1	GE274519
61918475	CBWT4536.g1	GE274520
61918476	CBWT4537.b1	GE274521
61918477	CBWT4537.g1	GE274522
61918478	CBWT4538.b1	GE274523
61918479	CBWT4538.g1	GE274524
61918480	CBWT4540.b1	GE274525
61918481	CBWT4540.g1	GE274526
61918482	CBWT4542.b1	GE274527

61918483	CBWT4542.g1	GE274528
61918484	CBWT4545.b1	GE274529
61918485	CBWT4545.g1	GE274530
61918486	CBWT4546.b1	GE274531
61918487	CBWT4546.g1	GE274532
61918488	CBWT4547.b1	GE274533
61918489	CBWT4547.g1	GE274534
61918490	CBWT455.b11	GE274535
61918491	CBWT455.g11	GE274536
61918492	CBWT4550.b1	GE274537
61918493	CBWT4550.g1	GE274538
61918494	CBWT4551.b1	GE274539
61918495	CBWT4551.g1	GE274540
61918496	CBWT4552.b1	GE274541
61918497	CBWT4552.g1	GE274542
61918498	CBWT4553.g1	GE274543
61918499	CBWT4554.b1	GE274544
61918500	CBWT4554.g1	GE274545
61918501	CBWT4555.b1	GE274546
61918502	CBWT4555.g1	GE274547
61918503	CBWT4558.b1	GE274548
61918504	CBWT4558.g1	GE274549
61918505	CBWT4561.b1	GE274550
61918506	CBWT4561.g1	GE274551
61918507	CBWT4563.b1	GE274552
61918508	CBWT4563.g1	GE274553
61918509	CBWT4565.b1	GE274554
61918510	CBWT4565.g1	GE274555
61918511	CBWT4566.b1	GE274556
61918512	CBWT4566.g1	GE274557
61918513	CBWT4567.b1	GE274558
61918514	CBWT4567.g1	GE274559
61918515	CBWT4568.b1	GE274560
61918516	CBWT4568.g1	GE274561
61918517	CBWT4569.b1	GE274562
61918518	CBWT4569.g1	GE274563
61918519	CBWT4570.b1	GE274564
61918520	CBWT4570.g1	GE274565
61918521	CBWT4571.b1	GE274566
61918522	CBWT4571.g1	GE274567
61918523	CBWT4572.b1	GE274568
61918524	CBWT4572.g1	GE274569
61918525	CBWT4573.b1	GE274570
61918526	CBWT4573.g1	GE274571
61918527	CBWT4574.g1	GE274572
61918528	CBWT4575.b1	GE274573
61918529	CBWT4575.g1	GE274574
61918530	CBWT4577.b1	GE274575
61918531	CBWT4577.g1	GE274576
61918532	CBWT4579.b1	GE274577
61918533	CBWT4579.g1	GE274578
61918534	CBWT4581.b1	GE274579
61918535	CBWT4581.g1	GE274580
61918536	CBWT4582.b1	GE274581
61918537	CBWT4583.b1	GE274582
61918538	CBWT4583.g1	GE274583
61918539	CBWT4585.b1	GE274584
61918540	CBWT4585.g1	GE274585
61918541	CBWT4587.b1	GE274586
61918542	CBWT4587.g1	GE274587
61918543	CBWT459.b11	GE274588
61918544	CBWT459.g11	GE274589
61918545	CBWT4590.b1	GE274590

61918546	CBWT4591.b1	GE274591
61918547	CBWT4591.g1	GE274592
61918548	CBWT4592.b1	GE274593
61918549	CBWT4592.g1	GE274594
61918550	CBWT4594.b1	GE274595
61918551	CBWT4594.g1	GE274596
61918552	CBWT4595.b1	GE274597
61918553	CBWT4596.b1	GE274598
61918554	CBWT4596.g1	GE274599
61918555	CBWT4598.b1	GE274600
61918556	CBWT4598.g1	GE274601
61918557	CBWT4602.b1	GE274602
61918558	CBWT4602.g1	GE274603
61918559	CBWT4603.g1	GE274604
61918560	CBWT4604.b1	GE274605
61918561	CBWT4604.g1	GE274606
61918562	CBWT4605.b1	GE274607
61918563	CBWT4605.g1	GE274608
61918564	CBWT4606.b1	GE274609
61918565	CBWT4606.g1	GE274610
61918566	CBWT4611.b1	GE274611
61918567	CBWT4611.g1	GE274612
61918568	CBWT4612.b1	GE274613
61918569	CBWT4612.g1	GE274614
61918570	CBWT4618.b1	GE274615
61918571	CBWT4618.g1	GE274616
61918572	CBWT4619.b1	GE274617
61918573	CBWT4619.g1	GE274618
61918574	CBWT462.b11	GE274619
61918575	CBWT462.g6	GE274620
61918576	CBWT4620.b1	GE274621
61918577	CBWT4620.g1	GE274622
61918578	CBWT4622.b1	GE274623
61918579	CBWT4622.g1	GE274624
61918580	CBWT4623.b1	GE274625
61918581	CBWT4624.b1	GE274626
61918582	CBWT4624.g1	GE274627
61918583	CBWT4625.b1	GE274628
61918584	CBWT4625.g1	GE274629
61918585	CBWT4626.b1	GE274630
61918586	CBWT4629.b1	GE274631
61918587	CBWT4629.g1	GE274632
61918588	CBWT4630.b1	GE274633
61918589	CBWT4630.g1	GE274634
61918590	CBWT4631.b1	GE274635
61918591	CBWT4631.g1	GE274636
61918592	CBWT4632.b1	GE274637
61918593	CBWT4632.g1	GE274638
61918594	CBWT4633.b1	GE274639
61918595	CBWT4633.g1	GE274640
61918596	CBWT4635.b1	GE274641
61918597	CBWT4635.g1	GE274642
61918598	CBWT4636.b1	GE274643
61918599	CBWT4636.g1	GE274644
61918600	CBWT4638.b1	GE274645
61918601	CBWT4639.b1	GE274646
61918602	CBWT4639.g1	GE274647
61918603	CBWT464.b11	GE274648
61918604	CBWT464.g11	GE274649
61918605	CBWT4640.b1	GE274650
61918606	CBWT4640.g1	GE274651
61918607	CBWT4643.b1	GE274652
61918608	CBWT4643.g1	GE274653

61918609	CBWT4644.b1	GE274654
61918610	CBWT4644.g1	GE274655
61918611	CBWT4645.b1	GE274656
61918612	CBWT4646.b1	GE274657
61918613	CBWT4646.g1	GE274658
61918614	CBWT4647.b1	GE274659
61918615	CBWT4647.g1	GE274660
61918616	CBWT4648.b1	GE274661
61918617	CBWT4648.g1	GE274662
61918618	CBWT4649.b1	GE274663
61918619	CBWT4649.g1	GE274664
61918620	CBWT4650.b1	GE274665
61918621	CBWT4650.g1	GE274666
61918622	CBWT4651.b1	GE274667
61918623	CBWT4651.g1	GE274668
61918624	CBWT4653.b1	GE274669
61918625	CBWT4653.g1	GE274670
61918626	CBWT4654.b1	GE274671
61918627	CBWT4654.g1	GE274672
61918628	CBWT4655.b1	GE274673
61918629	CBWT4655.g1	GE274674
61918630	CBWT4657.b1	GE274675
61918631	CBWT4657.g1	GE274676
61918632	CBWT4658.b1	GE274677
61918633	CBWT4658.g1	GE274678
61918634	CBWT4659.b1	GE274679
61918635	CBWT4659.g1	GE274680
61918636	CBWT4661.b1	GE274681
61918637	CBWT4661.g1	GE274682
61918638	CBWT4664.b1	GE274683
61918639	CBWT4664.g1	GE274684
61918640	CBWT4665.b1	GE274685
61918641	CBWT4665.g1	GE274686
61918642	CBWT4666.g1	GE274687
61918643	CBWT4668.b1	GE274688
61918644	CBWT4668.g1	GE274689
61918645	CBWT4669.b1	GE274690
61918646	CBWT4669.g1	GE274691
61918647	CBWT467.b11	GE274692
61918648	CBWT467.g11	GE274693
61918649	CBWT4671.b1	GE274694
61918650	CBWT4671.g1	GE274695
61918651	CBWT4673.b1	GE274696
61918652	CBWT4673.g1	GE274697
61918653	CBWT4675.b1	GE274698
61918654	CBWT4675.g1	GE274699
61918655	CBWT4676.b1	GE274700
61918656	CBWT4676.g1	GE274701
61918657	CBWT4677.b1	GE274702
61918658	CBWT4677.g1	GE274703
61918659	CBWT4678.b1	GE274704
61918660	CBWT4678.g1	GE274705
61918661	CBWT4679.b1	GE274706
61918662	CBWT4679.g1	GE274707
61918663	CBWT468.b11	GE274708
61918664	CBWT468.g11	GE274709
61918665	CBWT4680.b1	GE274710
61918666	CBWT4680.g1	GE274711
61918667	CBWT4682.b1	GE274712
61918668	CBWT4682.g1	GE274713
61918669	CBWT4683.b1	GE274714
61918670	CBWT4685.b1	GE274715
61918671	CBWT4685.g1	GE274716

61918672	CBWT4690.b1	GE274717
61918673	CBWT4690.g1	GE274718
61918674	CBWT4692.b1	GE274719
61918675	CBWT4692.g1	GE274720
61918676	CBWT4693.b1	GE274721
61918677	CBWT4693.g1	GE274722
61918678	CBWT4694.b1	GE274723
61918679	CBWT4694.g1	GE274724
61918680	CBWT4695.b1	GE274725
61918681	CBWT4695.g1	GE274726
61918682	CBWT4696.b1	GE274727
61918683	CBWT4696.g1	GE274728
61918684	CBWT4700.b1	GE274729
61918685	CBWT4700.g1	GE274730
61918686	CBWT4704.b1	GE274731
61918687	CBWT4706.b1	GE274732
61918688	CBWT4706.g1	GE274733
61918689	CBWT4707.b1	GE274734
61918690	CBWT4707.g1	GE274735
61918691	CBWT4708.b1	GE274736
61918692	CBWT4708.g1	GE274737
61918693	CBWT4709.b1	GE274738
61918694	CBWT4709.g1	GE274739
61918695	CBWT471.b11	GE274740
61918696	CBWT471.g11	GE274741
61918697	CBWT4711.b1	GE274742
61918698	CBWT4711.g1	GE274743
61918699	CBWT4712.g1	GE274744
61918700	CBWT4714.b1	GE274745
61918701	CBWT4714.g1	GE274746
61918702	CBWT4715.b1	GE274747
61918703	CBWT4717.b1	GE274748
61918704	CBWT4717.g1	GE274749
61918705	CBWT4719.b1	GE274750
61918706	CBWT4719.g1	GE274751
61918707	CBWT4723.b1	GE274752
61918708	CBWT4723.g1	GE274753
61918709	CBWT4724.g1	GE274754
61918710	CBWT4726.b1	GE274755
61918711	CBWT4726.g1	GE274756
61918712	CBWT4727.b1	GE274757
61918713	CBWT4727.g1	GE274758
61918714	CBWT4728.b1	GE274759
61918715	CBWT4728.g1	GE274760
61918716	CBWT4729.b1	GE274761
61918717	CBWT4729.g1	GE274762
61918718	CBWT4730.b1	GE274763
61918719	CBWT4730.g1	GE274764
61918720	CBWT4731.b1	GE274765
61918721	CBWT4731.g1	GE274766
61918722	CBWT4732.b1	GE274767
61918723	CBWT4732.g1	GE274768
61918724	CBWT4733.b1	GE274769
61918725	CBWT4733.g1	GE274770
61918726	CBWT4734.b1	GE274771
61918727	CBWT4734.g1	GE274772
61918728	CBWT4736.b1	GE274773
61918729	CBWT4738.b1	GE274774
61918730	CBWT4738.g1	GE274775
61918731	CBWT4739.b1	GE274776
61918732	CBWT4739.g1	GE274777
61918733	CBWT4740.b1	GE274778
61918734	CBWT4740.g1	GE274779

61918735	CBWT4744.b1	GE274780
61918736	CBWT4744.g1	GE274781
61918737	CBWT4745.b1	GE274782
61918738	CBWT4745.g1	GE274783
61918739	CBWT4746.b1	GE274784
61918740	CBWT4746.g1	GE274785
61918741	CBWT4749.b1	GE274786
61918742	CBWT4749.g1	GE274787
61918743	CBWT4750.b1	GE274788
61918744	CBWT4750.g1	GE274789
61918745	CBWT4751.b1	GE274790
61918746	CBWT4751.g1	GE274791
61918747	CBWT4753.b1	GE274792
61918748	CBWT4753.g1	GE274793
61918749	CBWT4755.b1	GE274794
61918750	CBWT4755.g1	GE274795
61918751	CBWT4756.b1	GE274796
61918752	CBWT4757.g1	GE274797
61918753	CBWT4758.b1	GE274798
61918754	CBWT4758.g1	GE274799
61918755	CBWT4759.b1	GE274800
61918756	CBWT4759.g1	GE274801
61918757	CBWT4760.b1	GE274802
61918758	CBWT4760.g1	GE274803
61918759	CBWT4762.b1	GE274804
61918760	CBWT4762.g1	GE274805
61918761	CBWT4763.b1	GE274806
61918762	CBWT4763.g1	GE274807
61918763	CBWT4764.b1	GE274808
61918764	CBWT4764.g1	GE274809
61918765	CBWT4765.b1	GE274810
61918766	CBWT4765.g1	GE274811
61918767	CBWT4766.b1	GE274812
61918768	CBWT4766.g1	GE274813
61918769	CBWT4767.b1	GE274814
61918770	CBWT4767.g1	GE274815
61918771	CBWT4768.b1	GE274816
61918772	CBWT4768.g1	GE274817
61918773	CBWT4769.b1	GE274818
61918774	CBWT4769.g1	GE274819
61918775	CBWT4771.b1	GE274820
61918776	CBWT4771.g1	GE274821
61918777	CBWT4774.b1	GE274822
61918778	CBWT4774.g1	GE274823
61918779	CBWT4777.g1	GE274824
61918780	CBWT4778.b1	GE274825
61918781	CBWT4778.g1	GE274826
61918782	CBWT478.b11	GE274827
61918783	CBWT478.g11	GE274828
61918784	CBWT4780.b1	GE274829
61918785	CBWT4780.g1	GE274830
61918786	CBWT4781.b1	GE274831
61918787	CBWT4781.g1	GE274832
61918788	CBWT4782.b1	GE274833
61918789	CBWT4782.g1	GE274834
61918790	CBWT4785.b1	GE274835
61918791	CBWT4785.g1	GE274836
61918792	CBWT4786.b1	GE274837
61918793	CBWT4786.g1	GE274838
61918794	CBWT4787.b1	GE274839
61918795	CBWT4787.g1	GE274840
61918796	CBWT4788.b1	GE274841
61918797	CBWT4788.g1	GE274842

61918798	CBWT4789.b1	GE274843
61918799	CBWT4789.g1	GE274844
61918800	CBWT479.b11	GE274845
61918801	CBWT479.g11	GE274846
61918802	CBWT4794.b1	GE274847
61918803	CBWT4794.g1	GE274848
61918804	CBWT4797.g1	GE274849
61918805	CBWT4798.b1	GE274850
61918806	CBWT4798.g1	GE274851
61918807	CBWT4799.b1	GE274852
61918808	CBWT4799.g1	GE274853
61918809	CBWT4800.b1	GE274854
61918810	CBWT4800.g1	GE274855
61918811	CBWT4802.b1	GE274856
61918812	CBWT4802.g1	GE274857
61918813	CBWT4803.b1	GE274858
61918814	CBWT4803.g1	GE274859
61918815	CBWT4807.b1	GE274860
61918816	CBWT4807.g1	GE274861
61918817	CBWT481.b1	GE274862
61918818	CBWT481.g11	GE274863
61918819	CBWT4811.b1	GE274864
61918820	CBWT4811.g1	GE274865
61918821	CBWT4812.b1	GE274866
61918822	CBWT4812.g1	GE274867
61918823	CBWT4814.b1	GE274868
61918824	CBWT4816.b1	GE274869
61918825	CBWT4816.g1	GE274870
61918826	CBWT4817.b1	GE274871
61918827	CBWT4817.g1	GE274872
61918828	CBWT4818.b1	GE274873
61918829	CBWT4818.g1	GE274874
61918830	CBWT4819.b1	GE274875
61918831	CBWT4819.g1	GE274876
61918832	CBWT482.b6	GE274877
61918833	CBWT482.g1	GE274878
61918834	CBWT4820.b1	GE274879
61918835	CBWT4820.g1	GE274880
61918836	CBWT4821.b1	GE274881
61918837	CBWT4821.g1	GE274882
61918838	CBWT4823.b1	GE274883
61918839	CBWT4823.g1	GE274884
61918840	CBWT4825.g1	GE274885
61918841	CBWT4826.b1	GE274886
61918842	CBWT4826.g1	GE274887
61918843	CBWT4827.b1	GE274888
61918844	CBWT4827.g1	GE274889
61918845	CBWT4828.b1	GE274890
61918846	CBWT4828.g1	GE274891
61918847	CBWT4829.b1	GE274892
61918848	CBWT4829.g1	GE274893
61918849	CBWT483.b11	GE274894
61918850	CBWT483.g11	GE274895
61918851	CBWT4831.b1	GE274896
61918852	CBWT4831.g1	GE274897
61918853	CBWT4832.g1	GE274898
61918854	CBWT4833.b1	GE274899
61918855	CBWT4833.g1	GE274900
61918856	CBWT4834.b1	GE274901
61918857	CBWT4834.g1	GE274902
61918858	CBWT4835.b1	GE274903
61918859	CBWT4835.g1	GE274904
61918860	CBWT4836.b1	GE274905

61918861	CBWT4836.g1	GE274906
61918862	CBWT4840.b1	GE274907
61918863	CBWT4840.g1	GE274908
61918864	CBWT4841.b1	GE274909
61918865	CBWT4841.g1	GE274910
61918866	CBWT4842.b1	GE274911
61918867	CBWT4842.g1	GE274912
61918868	CBWT4843.b1	GE274913
61918869	CBWT4843.g1	GE274914
61918870	CBWT4845.b1	GE274915
61918871	CBWT4845.g1	GE274916
61918872	CBWT4846.b1	GE274917
61918873	CBWT4846.g1	GE274918
61918874	CBWT4847.b1	GE274919
61918875	CBWT4847.g1	GE274920
61918876	CBWT4848.b1	GE274921
61918877	CBWT4848.g1	GE274922
61918878	CBWT4849.b1	GE274923
61918879	CBWT4849.g1	GE274924
61918880	CBWT4850.b1	GE274925
61918881	CBWT4850.g1	GE274926
61918882	CBWT4851.b1	GE274927
61918883	CBWT4856.b1	GE274928
61918884	CBWT4856.g1	GE274929
61918885	CBWT4857.b1	GE274930
61918886	CBWT4857.g1	GE274931
61918887	CBWT4858.b1	GE274932
61918888	CBWT4858.g1	GE274933
61918889	CBWT4859.b1	GE274934
61918890	CBWT4859.g1	GE274935
61918891	CBWT4861.b1	GE274936
61918892	CBWT4861.g1	GE274937
61918893	CBWT4863.g1	GE274938
61918894	CBWT4865.b1	GE274939
61918895	CBWT4865.g1	GE274940
61918896	CBWT4866.b1	GE274941
61918897	CBWT4866.g1	GE274942
61918898	CBWT4867.b1	GE274943
61918899	CBWT4867.g1	GE274944
61918900	CBWT4868.b1	GE274945
61918901	CBWT4868.g1	GE274946
61918902	CBWT4870.b1	GE274947
61918903	CBWT4870.g1	GE274948
61918904	CBWT4872.b1	GE274949
61918905	CBWT4872.g1	GE274950
61918906	CBWT4874.g1	GE274951
61918907	CBWT4875.b1	GE274952
61918908	CBWT4875.g1	GE274953
61918909	CBWT4876.b1	GE274954
61918910	CBWT4876.g1	GE274955
61918911	CBWT4877.b1	GE274956
61918912	CBWT4877.g1	GE274957
61918913	CBWT4878.b1	GE274958
61918914	CBWT4878.g1	GE274959
61918915	CBWT4879.b1	GE274960
61918916	CBWT4879.g1	GE274961
61918917	CBWT4880.b1	GE274962
61918918	CBWT4880.g1	GE274963
61918919	CBWT4882.b1	GE274964
61918920	CBWT4882.g1	GE274965
61918921	CBWT4886.b1	GE274966
61918922	CBWT4886.g1	GE274967
61918923	CBWT4887.b1	GE274968

61918924	CBWT4887.g1	GE274969
61918925	CBWT4889.g1	GE274970
61918926	CBWT489.b1	GE274971
61918927	CBWT489.g1	GE274972
61918928	CBWT4890.b1	GE274973
61918929	CBWT4890.g1	GE274974
61918930	CBWT4891.g1	GE274975
61918931	CBWT4892.b1	GE274976
61918932	CBWT4892.g1	GE274977
61918933	CBWT4893.b1	GE274978
61918934	CBWT4893.g1	GE274979
61918935	CBWT4895.b1	GE274980
61918936	CBWT4895.g1	GE274981
61918937	CBWT4898.b1	GE274982
61918938	CBWT4898.g1	GE274983
61918939	CBWT4902.b1	GE274984
61918940	CBWT4902.g1	GE274985
61918941	CBWT4903.b1	GE274986
61918942	CBWT4903.g1	GE274987
61918943	CBWT4905.b1	GE274988
61918944	CBWT4905.g1	GE274989
61918945	CBWT4906.b1	GE274990
61918946	CBWT4906.g1	GE274991
61918947	CBWT4907.b1	GE274992
61918948	CBWT4907.g1	GE274993
61918949	CBWT4908.b1	GE274994
61918950	CBWT4908.g1	GE274995
61918951	CBWT491.b1	GE274996
61918952	CBWT491.g6	GE274997
61918953	CBWT4910.b1	GE274998
61918954	CBWT4912.b1	GE274999
61918955	CBWT4912.g1	GE275000
61918956	CBWT4913.b1	GE275001
61918957	CBWT4913.g1	GE275002
61918958	CBWT4914.b1	GE275003
61918959	CBWT4914.g1	GE275004
61918960	CBWT4915.b1	GE275005
61918961	CBWT4916.b1	GE275006
61918962	CBWT4918.b1	GE275007
61918963	CBWT4918.g1	GE275008
61918964	CBWT4919.b1	GE275009
61918965	CBWT4919.g1	GE275010
61918966	CBWT492.b1	GE275011
61918967	CBWT492.g1	GE275012
61918968	CBWT4920.b1	GE275013
61918969	CBWT4920.g1	GE275014
61918970	CBWT4921.b1	GE275015
61918971	CBWT4921.g1	GE275016
61918972	CBWT4922.b1	GE275017
61918973	CBWT4922.g1	GE275018
61918974	CBWT4925.b1	GE275019
61918975	CBWT4925.g1	GE275020
61918976	CBWT4926.b1	GE275021
61918977	CBWT4926.g1	GE275022
61918978	CBWT4927.b1	GE275023
61918979	CBWT4927.g1	GE275024
61918980	CBWT4928.b1	GE275025
61918981	CBWT4928.g1	GE275026
61918982	CBWT4929.b1	GE275027
61918983	CBWT4929.g1	GE275028
61918984	CBWT493.b6	GE275029
61918985	CBWT493.g6	GE275030
61918986	CBWT4930.b1	GE275031

61918987	CBWT4931.b1	GE275032
61918988	CBWT4931.g1	GE275033
61918989	CBWT4932.b1	GE275034
61918990	CBWT4932.g1	GE275035
61918991	CBWT4937.b1	GE275036
61918992	CBWT4937.g1	GE275037
61918993	CBWT4938.b1	GE275038
61918994	CBWT4938.g1	GE275039
61918995	CBWT4939.b1	GE275040
61918996	CBWT4939.g1	GE275041
61918997	CBWT4940.b1	GE275042
61918998	CBWT4940.g1	GE275043
61918999	CBWT4942.b1	GE275044
61919000	CBWT4942.g1	GE275045
61919001	CBWT4943.b1	GE275046
61919002	CBWT4943.g1	GE275047
61919003	CBWT4944.b1	GE275048
61919004	CBWT4944.g1	GE275049
61919005	CBWT4945.b1	GE275050
61919006	CBWT4945.g1	GE275051
61919007	CBWT4946.b1	GE275052
61919008	CBWT4946.g1	GE275053
61919009	CBWT4947.g1	GE275054
61919010	CBWT4949.b1	GE275055
61919011	CBWT4949.g1	GE275056
61919012	CBWT495.b11	GE275057
61919013	CBWT495.g11	GE275058
61919014	CBWT4950.b1	GE275059
61919015	CBWT4950.g1	GE275060
61919016	CBWT4951.b1	GE275061
61919017	CBWT4951.g1	GE275062
61919018	CBWT4955.b1	GE275063
61919019	CBWT4955.g1	GE275064
61919020	CBWT4956.b1	GE275065
61919021	CBWT4956.g1	GE275066
61919022	CBWT4957.b1	GE275067
61919023	CBWT4957.g1	GE275068
61919024	CBWT4959.b1	GE275069
61919025	CBWT4959.g1	GE275070
61919026	CBWT496.b1	GE275071
61919027	CBWT496.g1	GE275072
61919028	CBWT4960.b1	GE275073
61919029	CBWT4960.g1	GE275074
61919030	CBWT4961.b1	GE275075
61919031	CBWT4961.g1	GE275076
61919032	CBWT4962.g1	GE275077
61919033	CBWT4963.b1	GE275078
61919034	CBWT4963.g1	GE275079
61919035	CBWT4965.b1	GE275080
61919036	CBWT4965.g1	GE275081
61919037	CBWT4966.b1	GE275082
61919038	CBWT4966.g1	GE275083
61919039	CBWT4969.b1	GE275084
61919040	CBWT4969.g1	GE275085
61919041	CBWT4970.b1	GE275086
61919042	CBWT4970.g1	GE275087
61919043	CBWT4971.b1	GE275088
61919044	CBWT4971.g1	GE275089
61919045	CBWT4972.b1	GE275090
61919046	CBWT4972.g1	GE275091
61919047	CBWT4974.b1	GE275092
61919048	CBWT4974.g1	GE275093
61919049	CBWT4975.b1	GE275094

61919050	CBWT4975.g1	GE275095
61919051	CBWT4976.b1	GE275096
61919052	CBWT4976.g1	GE275097
61919053	CBWT4977.b1	GE275098
61919054	CBWT4977.g1	GE275099
61919055	CBWT4979.b1	GE275100
61919056	CBWT4979.g1	GE275101
61919057	CBWT498.b6	GE275102
61919058	CBWT498.g1	GE275103
61919059	CBWT4981.b1	GE275104
61919060	CBWT4981.g1	GE275105
61919061	CBWT4983.b1	GE275106
61919062	CBWT4983.g1	GE275107
61919063	CBWT4984.b1	GE275108
61919064	CBWT4984.g1	GE275109
61919065	CBWT4986.b1	GE275110
61919066	CBWT4986.g1	GE275111
61919067	CBWT4987.b1	GE275112
61919068	CBWT4987.g1	GE275113
61919069	CBWT4988.b1	GE275114
61919070	CBWT4988.g1	GE275115
61919071	CBWT499.b11	GE275116
61919072	CBWT4991.b1	GE275117
61919073	CBWT4991.g1	GE275118
61919074	CBWT4992.g1	GE275119
61919075	CBWT4994.b1	GE275120
61919076	CBWT4994.g1	GE275121
61919077	CBWT4995.b1	GE275122
61919078	CBWT4995.g1	GE275123
61919079	CBWT4996.b1	GE275124
61919080	CBWT4996.g1	GE275125
61919081	CBWT4997.b1	GE275126
61919082	CBWT4997.g1	GE275127
61919083	CBWT4999.b1	GE275128
61919084	CBWT5001.b1	GE275129
61919085	CBWT5002.b1	GE275130
61919086	CBWT5003.b1	GE275131
61919087	CBWT5003.g1	GE275132
61919088	CBWT5004.b1	GE275133
61919089	CBWT5004.g1	GE275134
61919090	CBWT5007.b1	GE275135
61919091	CBWT5007.g1	GE275136
61919092	CBWT5008.b1	GE275137
61919093	CBWT5009.b1	GE275138
61919094	CBWT5009.g1	GE275139
61919095	CBWT501.b11	GE275140
61919096	CBWT501.g1	GE275141
61919097	CBWT5010.b1	GE275142
61919098	CBWT5010.g1	GE275143
61919099	CBWT5011.b1	GE275144
61919100	CBWT5011.g1	GE275145
61919101	CBWT5012.b1	GE275146
61919102	CBWT5013.b1	GE275147
61919103	CBWT5013.g1	GE275148
61919104	CBWT5014.b1	GE275149
61919105	CBWT5014.g1	GE275150
61919106	CBWT5015.b1	GE275151
61919107	CBWT5015.g1	GE275152
61919108	CBWT5019.b1	GE275153
61919109	CBWT5019.g1	GE275154
61919110	CBWT502.b11	GE275155
61919111	CBWT5020.b1	GE275156
61919112	CBWT5020.g1	GE275157

61919113	CBWT5021.b1	GE275158
61919114	CBWT5021.g1	GE275159
61919115	CBWT5023.b1	GE275160
61919116	CBWT5023.g1	GE275161
61919117	CBWT5024.b1	GE275162
61919118	CBWT5024.g1	GE275163
61919119	CBWT5025.b1	GE275164
61919120	CBWT5025.g1	GE275165
61919121	CBWT5026.b1	GE275166
61919122	CBWT5029.b1	GE275167
61919123	CBWT5029.g1	GE275168
61919124	CBWT5031.b1	GE275169
61919125	CBWT5031.g1	GE275170
61919126	CBWT5032.b1	GE275171
61919127	CBWT5032.g1	GE275172
61919128	CBWT5033.b1	GE275173
61919129	CBWT5033.g1	GE275174
61919130	CBWT5035.g1	GE275175
61919131	CBWT5036.b1	GE275176
61919132	CBWT5036.g1	GE275177
61919133	CBWT5037.b1	GE275178
61919134	CBWT5037.g1	GE275179
61919135	CBWT5038.b1	GE275180
61919136	CBWT5038.g1	GE275181
61919137	CBWT504.b1	GE275182
61919138	CBWT504.g6	GE275183
61919139	CBWT5040.b1	GE275184
61919140	CBWT5040.g1	GE275185
61919141	CBWT5041.b1	GE275186
61919142	CBWT5042.b1	GE275187
61919143	CBWT5042.g1	GE275188
61919144	CBWT5043.b1	GE275189
61919145	CBWT5043.g1	GE275190
61919146	CBWT5045.g1	GE275191
61919147	CBWT5047.b1	GE275192
61919148	CBWT5047.g1	GE275193
61919149	CBWT5048.b1	GE275194
61919150	CBWT5048.g1	GE275195
61919151	CBWT5049.b1	GE275196
61919152	CBWT5049.g1	GE275197
61919153	CBWT5051.b1	GE275198
61919154	CBWT5051.g1	GE275199
61919155	CBWT5052.b1	GE275200
61919156	CBWT5052.g1	GE275201
61919157	CBWT5054.g1	GE275202
61919158	CBWT5055.b1	GE275203
61919159	CBWT5056.b1	GE275204
61919160	CBWT5057.b1	GE275205
61919161	CBWT5057.g1	GE275206
61919162	CBWT5058.b1	GE275207
61919163	CBWT5058.g1	GE275208
61919164	CBWT5059.b1	GE275209
61919165	CBWT5059.g1	GE275210
61919166	CBWT5060.b1	GE275211
61919167	CBWT5060.g1	GE275212
61919168	CBWT5061.b1	GE275213
61919169	CBWT5061.g1	GE275214
61919170	CBWT5062.g1	GE275215
61919171	CBWT5064.b1	GE275216
61919172	CBWT5064.g1	GE275217
61919173	CBWT5066.b1	GE275218
61919174	CBWT5066.g1	GE275219
61919175	CBWT5067.g1	GE275220

61919176	CBWT5068.g1	GE275221
61919177	CBWT5069.b1	GE275222
61919178	CBWT5069.g1	GE275223
61919179	CBWT5070.b1	GE275224
61919180	CBWT5070.g1	GE275225
61919181	CBWT5071.b1	GE275226
61919182	CBWT5071.g1	GE275227
61919183	CBWT5072.b1	GE275228
61919184	CBWT5072.g1	GE275229
61919185	CBWT5076.b1	GE275230
61919186	CBWT5076.g1	GE275231
61919187	CBWT5077.b1	GE275232
61919188	CBWT5077.g1	GE275233
61919189	CBWT5078.b1	GE275234
61919190	CBWT5078.g1	GE275235
61919191	CBWT5079.b1	GE275236
61919192	CBWT5079.g1	GE275237
61919193	CBWT5080.b1	GE275238
61919194	CBWT5080.g1	GE275239
61919195	CBWT5081.b1	GE275240
61919196	CBWT5084.b1	GE275241
61919197	CBWT5084.g1	GE275242
61919198	CBWT5085.b1	GE275243
61919199	CBWT5085.g1	GE275244
61919200	CBWT5086.b1	GE275245
61919201	CBWT5086.g1	GE275246
61919202	CBWT5088.b1	GE275247
61919203	CBWT5089.b1	GE275248
61919204	CBWT5089.g1	GE275249
61919205	CBWT5090.b1	GE275250
61919206	CBWT5090.g1	GE275251
61919207	CBWT5092.b1	GE275252
61919208	CBWT5092.g1	GE275253
61919209	CBWT5097.b1	GE275254
61919210	CBWT5097.g1	GE275255
61919211	CBWT5098.b1	GE275256
61919212	CBWT5098.g1	GE275257
61919213	CBWT5099.b1	GE275258
61919214	CBWT5099.g1	GE275259
61919215	CBWT5100.b1	GE275260
61919216	CBWT5100.g1	GE275261
61919217	CBWT5101.b1	GE275262
61919218	CBWT5101.g1	GE275263
61919219	CBWT5102.b1	GE275264
61919220	CBWT5102.g1	GE275265
61919221	CBWT5103.b1	GE275266
61919222	CBWT5103.g1	GE275267
61919223	CBWT5104.b1	GE275268
61919224	CBWT5104.g1	GE275269
61919225	CBWT5105.b1	GE275270
61919226	CBWT5105.g1	GE275271
61919227	CBWT5106.b1	GE275272
61919228	CBWT5106.g1	GE275273
61919229	CBWT5107.b1	GE275274
61919230	CBWT5107.g1	GE275275
61919231	CBWT5109.b1	GE275276
61919232	CBWT5109.g1	GE275277
61919233	CBWT511.b6	GE275278
61919234	CBWT511.g6	GE275279
61919235	CBWT5110.b1	GE275280
61919236	CBWT5110.g1	GE275281
61919237	CBWT5112.b1	GE275282
61919238	CBWT5115.b1	GE275283

61919239	CBWT5115.g1	GE275284
61919240	CBWT5116.b1	GE275285
61919241	CBWT5116.g1	GE275286
61919242	CBWT5117.b1	GE275287
61919243	CBWT5117.g1	GE275288
61919244	CBWT5118.b1	GE275289
61919245	CBWT5118.g1	GE275290
61919246	CBWT5120.b1	GE275291
61919247	CBWT5120.g1	GE275292
61919248	CBWT5122.b1	GE275293
61919249	CBWT5122.g1	GE275294
61919250	CBWT5124.b1	GE275295
61919251	CBWT5124.g1	GE275296
61919252	CBWT5125.b1	GE275297
61919253	CBWT5125.g1	GE275298
61919254	CBWT5126.b1	GE275299
61919255	CBWT5126.g1	GE275300
61919256	CBWT5127.b1	GE275301
61919257	CBWT5127.g1	GE275302
61919258	CBWT513.b11	GE275303
61919259	CBWT513.g11	GE275304
61919260	CBWT5130.b1	GE275305
61919261	CBWT5130.g1	GE275306
61919262	CBWT5131.b1	GE275307
61919263	CBWT5132.b1	GE275308
61919264	CBWT5132.g1	GE275309
61919265	CBWT5134.b1	GE275310
61919266	CBWT5134.g1	GE275311
61919267	CBWT5136.b1	GE275312
61919268	CBWT5136.g1	GE275313
61919269	CBWT5137.b1	GE275314
61919270	CBWT5137.g1	GE275315
61919271	CBWT5138.b1	GE275316
61919272	CBWT5138.g1	GE275317
61919273	CBWT5141.b1	GE275318
61919274	CBWT5141.g1	GE275319
61919275	CBWT5142.b1	GE275320
61919276	CBWT5142.g1	GE275321
61919277	CBWT5144.b1	GE275322
61919278	CBWT5144.g1	GE275323
61919279	CBWT5146.b1	GE275324
61919280	CBWT5148.b1	GE275325
61919281	CBWT5148.g1	GE275326
61919282	CBWT5149.g1	GE275327
61919283	CBWT515.b6	GE275328
61919284	CBWT515.g6	GE275329
61919285	CBWT5150.b1	GE275330
61919286	CBWT5150.g1	GE275331
61919287	CBWT5151.b1	GE275332
61919288	CBWT5151.g1	GE275333
61919289	CBWT5152.b1	GE275334
61919290	CBWT5152.g1	GE275335
61919291	CBWT5154.b1	GE275336
61919292	CBWT5154.g1	GE275337
61919293	CBWT5155.b1	GE275338
61919294	CBWT5156.b1	GE275339
61919295	CBWT5156.g1	GE275340
61919296	CBWT5157.b1	GE275341
61919297	CBWT5157.g1	GE275342
61919298	CBWT5158.b1	GE275343
61919299	CBWT5158.g1	GE275344
61919300	CBWT5159.b1	GE275345
61919301	CBWT5159.g1	GE275346

61919302	CBWT5160.b1	GE275347
61919303	CBWT5160.g1	GE275348
61919304	CBWT5161.b1	GE275349
61919305	CBWT5161.g1	GE275350
61919306	CBWT5162.b1	GE275351
61919307	CBWT5162.g1	GE275352
61919308	CBWT5165.b1	GE275353
61919309	CBWT5165.g1	GE275354
61919310	CBWT5166.b1	GE275355
61919311	CBWT5166.g1	GE275356
61919312	CBWT5167.b1	GE275357
61919313	CBWT5167.g1	GE275358
61919314	CBWT5168.b1	GE275359
61919315	CBWT5168.g1	GE275360
61919316	CBWT5170.b1	GE275361
61919317	CBWT5170.g1	GE275362
61919318	CBWT5171.b1	GE275363
61919319	CBWT5172.b1	GE275364
61919320	CBWT5172.g1	GE275365
61919321	CBWT5173.b1	GE275366
61919322	CBWT5173.g1	GE275367
61919323	CBWT5174.b1	GE275368
61919324	CBWT5174.g1	GE275369
61919325	CBWT5175.b1	GE275370
61919326	CBWT5175.g1	GE275371
61919327	CBWT5176.b1	GE275372
61919328	CBWT5176.g1	GE275373
61919329	CBWT5178.b1	GE275374
61919330	CBWT5179.b1	GE275375
61919331	CBWT5179.g1	GE275376
61919332	CBWT5180.g1	GE275377
61919333	CBWT5181.b1	GE275378
61919334	CBWT5181.g1	GE275379
61919335	CBWT5182.b1	GE275380
61919336	CBWT5182.g1	GE275381
61919337	CBWT5183.g1	GE275382
61919338	CBWT5186.b1	GE275383
61919339	CBWT5186.g1	GE275384
61919340	CBWT5189.b1	GE275385
61919341	CBWT5189.g1	GE275386
61919342	CBWT5190.b1	GE275387
61919343	CBWT5190.g1	GE275388
61919344	CBWT5191.b1	GE275389
61919345	CBWT5191.g1	GE275390
61919346	CBWT5193.b1	GE275391
61919347	CBWT5193.g1	GE275392
61919348	CBWT5194.b1	GE275393
61919349	CBWT5194.g1	GE275394
61919350	CBWT5195.b1	GE275395
61919351	CBWT5195.g1	GE275396
61919352	CBWT5197.b1	GE275397
61919353	CBWT5197.g1	GE275398
61919354	CBWT5198.b1	GE275399
61919355	CBWT5198.g1	GE275400
61919356	CBWT520.g6	GE275401
61919357	CBWT5201.b1	GE275402
61919358	CBWT5201.g1	GE275403
61919359	CBWT5203.b1	GE275404
61919360	CBWT5203.g1	GE275405
61919361	CBWT5207.b1	GE275406
61919362	CBWT5207.g1	GE275407
61919363	CBWT5208.b1	GE275408
61919364	CBWT5208.g1	GE275409

61919365	CBWT5209.b1	GE275410
61919366	CBWT5209.g1	GE275411
61919367	CBWT5211.g1	GE275412
61919368	CBWT5212.b1	GE275413
61919369	CBWT5212.g1	GE275414
61919370	CBWT5214.b1	GE275415
61919371	CBWT5214.g1	GE275416
61919372	CBWT5215.b1	GE275417
61919373	CBWT5215.g1	GE275418
61919374	CBWT5217.b1	GE275419
61919375	CBWT5217.g1	GE275420
61919376	CBWT5218.b1	GE275421
61919377	CBWT5218.g1	GE275422
61919378	CBWT5221.b1	GE275423
61919379	CBWT5221.g1	GE275424
61919380	CBWT5223.b1	GE275425
61919381	CBWT5223.g1	GE275426
61919382	CBWT5224.b1	GE275427
61919383	CBWT5224.g1	GE275428
61919384	CBWT5225.b1	GE275429
61919385	CBWT5225.g1	GE275430
61919386	CBWT5227.b1	GE275431
61919387	CBWT5227.g1	GE275432
61919388	CBWT5228.b1	GE275433
61919389	CBWT5228.g1	GE275434
61919390	CBWT5231.b1	GE275435
61919391	CBWT5231.g1	GE275436
61919392	CBWT5232.b1	GE275437
61919393	CBWT5232.g1	GE275438
61919394	CBWT5234.b1	GE275439
61919395	CBWT5234.g1	GE275440
61919396	CBWT5235.b1	GE275441
61919397	CBWT5235.g1	GE275442
61919398	CBWT5236.b1	GE275443
61919399	CBWT5236.g1	GE275444
61919400	CBWT5239.b1	GE275445
61919401	CBWT5239.g1	GE275446
61919402	CBWT5240.b1	GE275447
61919403	CBWT5241.b1	GE275448
61919404	CBWT5241.g1	GE275449
61919405	CBWT5242.b1	GE275450
61919406	CBWT5242.g1	GE275451
61919407	CBWT5244.b1	GE275452
61919408	CBWT5244.g1	GE275453
61919409	CBWT5245.b1	GE275454
61919410	CBWT5245.g1	GE275455
61919411	CBWT5246.b1	GE275456
61919412	CBWT5246.g1	GE275457
61919413	CBWT5247.b1	GE275458
61919414	CBWT5247.g1	GE275459
61919415	CBWT5248.b1	GE275460
61919416	CBWT5248.g1	GE275461
61919417	CBWT5249.b1	GE275462
61919418	CBWT5249.g1	GE275463
61919419	CBWT5250.b1	GE275464
61919420	CBWT5250.g1	GE275465
61919421	CBWT5252.b1	GE275466
61919422	CBWT5253.b1	GE275467
61919423	CBWT5253.g1	GE275468
61919424	CBWT5255.b1	GE275469
61919425	CBWT5255.g1	GE275470
61919426	CBWT5257.b1	GE275471
61919427	CBWT5257.g1	GE275472

61919428	CBWT5258.b1	GE275473
61919429	CBWT5258.g1	GE275474
61919430	CBWT5260.b1	GE275475
61919431	CBWT5261.b1	GE275476
61919432	CBWT5261.g1	GE275477
61919433	CBWT5262.b1	GE275478
61919434	CBWT5262.g1	GE275479
61919435	CBWT5263.g1	GE275480
61919436	CBWT5264.b1	GE275481
61919437	CBWT5264.g1	GE275482
61919438	CBWT5265.b1	GE275483
61919439	CBWT5265.g1	GE275484
61919440	CBWT5266.b1	GE275485
61919441	CBWT5266.g1	GE275486
61919442	CBWT5268.b1	GE275487
61919443	CBWT5268.g1	GE275488
61919444	CBWT5269.g1	GE275489
61919445	CBWT5272.b1	GE275490
61919446	CBWT5272.g1	GE275491
61919447	CBWT5275.b1	GE275492
61919448	CBWT5275.g1	GE275493
61919449	CBWT5276.b1	GE275494
61919450	CBWT5276.g1	GE275495
61919451	CBWT5278.b1	GE275496
61919452	CBWT5278.g1	GE275497
61919453	CBWT5279.b1	GE275498
61919454	CBWT5279.g1	GE275499
61919455	CBWT5281.b1	GE275500
61919456	CBWT5281.g1	GE275501
61919457	CBWT5282.g1	GE275502
61919458	CBWT5284.b1	GE275503
61919459	CBWT5284.g1	GE275504
61919460	CBWT5285.b1	GE275505
61919461	CBWT5285.g1	GE275506
61919462	CBWT5287.b1	GE275507
61919463	CBWT5287.g1	GE275508
61919464	CBWT5289.b1	GE275509
61919465	CBWT5289.g1	GE275510
61919466	CBWT5291.b1	GE275511
61919467	CBWT5291.g1	GE275512
61919468	CBWT5292.g1	GE275513
61919469	CBWT5294.b1	GE275514
61919470	CBWT5294.g1	GE275515
61919471	CBWT5295.b1	GE275516
61919472	CBWT5295.g1	GE275517
61919473	CBWT5296.b1	GE275518
61919474	CBWT5296.g1	GE275519
61919475	CBWT5298.b1	GE275520
61919476	CBWT5298.g1	GE275521
61919477	CBWT5299.b1	GE275522
61919478	CBWT5299.g1	GE275523
61919479	CBWT5300.b1	GE275524
61919480	CBWT5301.b1	GE275525
61919481	CBWT5301.g1	GE275526
61919482	CBWT5302.b1	GE275527
61919483	CBWT5302.g1	GE275528
61919484	CBWT5303.b1	GE275529
61919485	CBWT5303.g1	GE275530
61919486	CBWT5304.b1	GE275531
61919487	CBWT5304.g1	GE275532
61919488	CBWT5305.b1	GE275533
61919489	CBWT5306.b1	GE275534
61919490	CBWT5306.g1	GE275535

61919491	CBWT5308.b1	GE275536
61919492	CBWT5308.g1	GE275537
61919493	CBWT531.b11	GE275538
61919494	CBWT531.g11	GE275539
61919495	CBWT5310.b1	GE275540
61919496	CBWT5310.g1	GE275541
61919497	CBWT5313.b1	GE275542
61919498	CBWT5313.g1	GE275543
61919499	CBWT5314.b1	GE275544
61919500	CBWT5314.g1	GE275545
61919501	CBWT5317.b1	GE275546
61919502	CBWT5317.g1	GE275547
61919503	CBWT5319.b1	GE275548
61919504	CBWT5319.g1	GE275549
61919505	CBWT5321.b1	GE275550
61919506	CBWT5321.g1	GE275551
61919507	CBWT5322.b1	GE275552
61919508	CBWT5322.g1	GE275553
61919509	CBWT5324.b1	GE275554
61919510	CBWT5324.g1	GE275555
61919511	CBWT5326.b1	GE275556
61919512	CBWT5326.g1	GE275557
61919513	CBWT5327.b1	GE275558
61919514	CBWT5327.g1	GE275559
61919515	CBWT5328.b1	GE275560
61919516	CBWT5328.g1	GE275561
61919517	CBWT5329.b1	GE275562
61919518	CBWT5329.g1	GE275563
61919519	CBWT533.b1	GE275564
61919520	CBWT533.g1	GE275565
61919521	CBWT5330.b1	GE275566
61919522	CBWT5330.g1	GE275567
61919523	CBWT5331.b1	GE275568
61919524	CBWT5331.g1	GE275569
61919525	CBWT5333.b1	GE275570
61919526	CBWT5333.g1	GE275571
61919527	CBWT5334.b1	GE275572
61919528	CBWT5334.g1	GE275573
61919529	CBWT5336.g1	GE275574
61919530	CBWT5337.g1	GE275575
61919531	CBWT5338.b1	GE275576
61919532	CBWT5338.g1	GE275577
61919533	CBWT5340.b1	GE275578
61919534	CBWT5340.g1	GE275579
61919535	CBWT5341.b1	GE275580
61919536	CBWT5341.g1	GE275581
61919537	CBWT5342.b1	GE275582
61919538	CBWT5342.g1	GE275583
61919539	CBWT5343.b1	GE275584
61919540	CBWT5343.g1	GE275585
61919541	CBWT5345.b1	GE275586
61919542	CBWT5345.g1	GE275587
61919543	CBWT5346.b1	GE275588
61919544	CBWT5349.b1	GE275589
61919545	CBWT5349.g1	GE275590
61919546	CBWT5350.g1	GE275591
61919547	CBWT5351.b1	GE275592
61919548	CBWT5351.g1	GE275593
61919549	CBWT5352.b1	GE275594
61919550	CBWT5352.g1	GE275595
61919551	CBWT5353.b1	GE275596
61919552	CBWT5353.g1	GE275597
61919553	CBWT5354.b1	GE275598

61919554	CBWT5354.g1	GE275599
61919555	CBWT5357.b1	GE275600
61919556	CBWT5357.g1	GE275601
61919557	CBWT5358.b1	GE275602
61919558	CBWT5358.g1	GE275603
61919559	CBWT5359.b1	GE275604
61919560	CBWT536.b11	GE275605
61919561	CBWT536.g1	GE275606
61919562	CBWT5360.b1	GE275607
61919563	CBWT5360.g1	GE275608
61919564	CBWT5361.b1	GE275609
61919565	CBWT5361.g1	GE275610
61919566	CBWT5362.b1	GE275611
61919567	CBWT5362.g1	GE275612
61919568	CBWT5363.b1	GE275613
61919569	CBWT5365.b1	GE275614
61919570	CBWT5365.g1	GE275615
61919571	CBWT5368.b1	GE275616
61919572	CBWT5368.g1	GE275617
61919573	CBWT537.b11	GE275618
61919574	CBWT537.g11	GE275619
61919575	CBWT5370.b1	GE275620
61919576	CBWT5370.g1	GE275621
61919577	CBWT5371.b1	GE275622
61919578	CBWT5371.g1	GE275623
61919579	CBWT5372.b1	GE275624
61919580	CBWT5372.g1	GE275625
61919581	CBWT538.b11	GE275626
61919582	CBWT538.g11	GE275627
61919583	CBWT539.b1	GE275628
61919584	CBWT539.g1	GE275629
61919585	CBWT546.g11	GE275630
61919586	CBWT547.b11	GE275631
61919587	CBWT547.g11	GE275632
61919588	CBWT552.b11	GE275633
61919589	CBWT552.g11	GE275634
61919590	CBWT553.b11	GE275635
61919591	CBWT553.g11	GE275636
61919592	CBWT555.b1	GE275637
61919593	CBWT555.g11	GE275638
61919594	CBWT557.b11	GE275639
61919595	CBWT557.g11	GE275640
61919596	CBWT560.b11	GE275641
61919597	CBWT561.b11	GE275642
61919598	CBWT561.g11	GE275643
61919599	CBWT564.b6	GE275644
61919600	CBWT564.g11	GE275645
61919601	CBWT565.b11	GE275646
61919602	CBWT565.g11	GE275647
61919603	CBWT568.b11	GE275648
61919604	CBWT568.g11	GE275649
61919605	CBWT574.b11	GE275650
61919606	CBWT574.g11	GE275651
61919607	CBWT577.b1	GE275652
61919608	CBWT577.g1	GE275653
61919609	CBWT578.b11	GE275654
61919610	CBWT578.g11	GE275655
61919611	CBWT579.b11	GE275656
61919612	CBWT579.g11	GE275657
61919613	CBWT585.b11	GE275658
61919614	CBWT585.g1	GE275659
61919615	CBWT586.b6	GE275660
61919616	CBWT586.g11	GE275661

61919617	CBWT592.b1	GE275662
61919618	CBWT592.g1	GE275663
61919619	CBWT594.b11	GE275664
61919620	CBWT594.g1	GE275665
61919621	CBWT595.b6	GE275666
61919622	CBWT595.g6	GE275667
61919623	CBWT596.b11	GE275668
61919624	CBWT596.g6	GE275669
61919625	CBWT597.b11	GE275670
61919626	CBWT597.g11	GE275671
61919627	CBWT598.b6	GE275672
61919628	CBWT598.g6	GE275673
61919629	CBWT599.b6	GE275674
61919630	CBWT602.b6	GE275675
61919631	CBWT602.g6	GE275676
61919632	CBWT604.b11	GE275677
61919633	CBWT604.g11	GE275678
61919634	CBWT606.b11	GE275679
61919635	CBWT606.g11	GE275680
61919636	CBWT607.b11	GE275681
61919637	CBWT607.g11	GE275682
61919638	CBWT608.b11	GE275683
61919639	CBWT610.b6	GE275684
61919640	CBWT610.g1	GE275685
61919641	CBWT613.b1	GE275686
61919642	CBWT613.g11	GE275687
61919643	CBWT614.b11	GE275688
61919644	CBWT614.g11	GE275689
61919645	CBWT616.b11	GE275690
61919646	CBWT616.g11	GE275691
61919647	CBWT618.b11	GE275692
61919648	CBWT618.g11	GE275693
61919649	CBWT625.b1	GE275694
61919650	CBWT625.g11	GE275695
61919651	CBWT631.b11	GE275696
61919652	CBWT631.g11	GE275697
61919653	CBWT632.b11	GE275698
61919654	CBWT632.g11	GE275699
61919655	CBWT637.b6	GE275700
61919656	CBWT637.g11	GE275701
61919657	CBWT638.b11	GE275702
61919658	CBWT638.g11	GE275703
61919659	CBWT639.b11	GE275704
61919660	CBWT639.g11	GE275705
61919661	CBWT640.b11	GE275706
61919662	CBWT640.g6	GE275707
61919663	CBWT646.b11	GE275708
61919664	CBWT646.g6	GE275709
61919665	CBWT647.b11	GE275710
61919666	CBWT647.g11	GE275711
61919667	CBWT648.b1	GE275712
61919668	CBWT648.g11	GE275713
61919669	CBWT651.b1	GE275714
61919670	CBWT651.g11	GE275715
61919671	CBWT653.b1	GE275716
61919672	CBWT653.g11	GE275717
61919673	CBWT654.b11	GE275718
61919674	CBWT654.g11	GE275719
61919675	CBWT655.b11	GE275720
61919676	CBWT655.g11	GE275721
61919677	CBWT656.b11	GE275722
61919678	CBWT656.g11	GE275723
61919679	CBWT660.b11	GE275724

61919680	CBWT661.b11	GE275725
61919681	CBWT662.b6	GE275726
61919682	CBWT665.b11	GE275727
61919683	CBWT665.g1	GE275728
61919684	CBWT667.b11	GE275729
61919685	CBWT667.g11	GE275730
61919686	CBWT672.b11	GE275731
61919687	CBWT672.g1	GE275732
61919688	CBWT673.b11	GE275733
61919689	CBWT673.g6	GE275734
61919690	CBWT675.b11	GE275735
61919691	CBWT675.g11	GE275736
61919692	CBWT677.b6	GE275737
61919693	CBWT677.g11	GE275738
61919694	CBWT682.b6	GE275739
61919695	CBWT682.g11	GE275740
61919696	CBWT691.b11	GE275741
61919697	CBWT691.g6	GE275742
61919698	CBWT692.b6	GE275743
61919699	CBWT692.g6	GE275744
61919700	CBWT695.b1	GE275745
61919701	CBWT697.b11	GE275746
61919702	CBWT697.g6	GE275747
61919703	CBWT698.b11	GE275748
61919704	CBWT699.b11	GE275749
61919705	CBWT699.g11	GE275750
61919706	CBWT706.b11	GE275751
61919707	CBWT706.g6	GE275752
61919708	CBWT710.b11	GE275753
61919709	CBWT710.g11	GE275754
61919710	CBWT711.b11	GE275755
61919711	CBWT711.g11	GE275756
61919712	CBWT712.b11	GE275757
61919713	CBWT712.g11	GE275758
61919714	CBWT716.b11	GE275759
61919715	CBWT716.g11	GE275760
61919716	CBWT717.b1	GE275761
61919717	CBWT717.g11	GE275762
61919718	CBWT725.b11	GE275763
61919719	CBWT725.g6	GE275764
61919720	CBWT728.b11	GE275765
61919721	CBWT728.g11	GE275766
61919722	CBWT729.g11	GE275767
61919723	CBWT732.b1	GE275768
61919724	CBWT733.b11	GE275769
61919725	CBWT733.g11	GE275770
61919726	CBWT737.b1	GE275771
61919727	CBWT737.g1	GE275772
61919728	CBWT739.b11	GE275773
61919729	CBWT739.g11	GE275774
61919730	CBWT740.b11	GE275775
61919731	CBWT740.g11	GE275776
61919732	CBWT741.b1	GE275777
61919733	CBWT744.b11	GE275778
61919734	CBWT744.g11	GE275779
61919735	CBWT745.b11	GE275780
61919736	CBWT745.g1	GE275781
61919737	CBWT748.b11	GE275782
61919738	CBWT748.g6	GE275783
61919739	CBWT750.b11	GE275784
61919740	CBWT753.b1	GE275785
61919741	CBWT753.g1	GE275786
61919742	CBWT754.b11	GE275787

61919743	CBWT754.g11	GE275788
61919744	CBWT755.b11	GE275789
61919745	CBWT755.g11	GE275790
61919746	CBWT757.b11	GE275791
61919747	CBWT757.g11	GE275792
61919748	CBWT767.b1	GE275793
61919749	CBWT767.g1	GE275794
61919750	CBYP1000.g1	GE275795
61919751	CBYP1001.g1	GE275796
61919752	CBYP1002.g1	GE275797
61919753	CBYP1003.g1	GE275798
61919754	CBYP1004.g1	GE275799
61919755	CBYP1006.g1	GE275800
61919756	CBYP1007.g1	GE275801
61919757	CBYP1008.g1	GE275802
61919758	CBYP1009.g1	GE275803
61919759	CBYP1010.g1	GE275804
61919760	CBYP1011.g1	GE275805
61919761	CBYP1012.g1	GE275806
61919762	CBYP1013.g1	GE275807
61919763	CBYP1014.g1	GE275808
61919764	CBYP1015.g1	GE275809
61919765	CBYP1016.g1	GE275810
61919766	CBYP1017.g1	GE275811
61919767	CBYP1018.g1	GE275812
61919768	CBYP1019.g1	GE275813
61919769	CBYP1020.g1	GE275814
61919770	CBYP1021.g1	GE275815
61919771	CBYP1022.g1	GE275816
61919772	CBYP1024.g1	GE275817
61919773	CBYP1025.g1	GE275818
61919774	CBYP1027.g1	GE275819
61919775	CBYP1028.g1	GE275820
61919776	CBYP1029.g1	GE275821
61919777	CBYP1030.g1	GE275822
61919778	CBYP1032.g1	GE275823
61919779	CBYP1033.g1	GE275824
61919780	CBYP1035.g1	GE275825
61919781	CBYP1038.g1	GE275826
61919782	CBYP1040.g1	GE275827
61919783	CBYP1041.g1	GE275828
61919784	CBYP1042.g1	GE275829
61919785	CBYP1044.g1	GE275830
61919786	CBYP1045.g1	GE275831
61919787	CBYP1046.g1	GE275832
61919788	CBYP1047.g1	GE275833
61919789	CBYP1048.g1	GE275834
61919790	CBYP1049.g1	GE275835
61919791	CBYP1050.g1	GE275836
61919792	CBYP1051.g1	GE275837
61919793	CBYP1052.g1	GE275838
61919794	CBYP1053.g1	GE275839
61919795	CBYP1054.g1	GE275840
61919796	CBYP1055.g1	GE275841
61919797	CBYP1057.g1	GE275842
61919798	CBYP1058.g1	GE275843
61919799	CBYP1060.g1	GE275844
61919800	CBYP1061.g1	GE275845
61919801	CBYP1062.g1	GE275846
61919802	CBYP1063.g1	GE275847
61919803	CBYP1064.g1	GE275848
61919804	CBYP1065.g1	GE275849
61919805	CBYP1067.g1	GE275850

61919806	CBYP1070.g1	GE275851
61919807	CBYP1071.g1	GE275852
61919808	CBYP1072.g1	GE275853
61919809	CBYP1073.g1	GE275854
61919810	CBYP1074.g1	GE275855
61919811	CBYP1075.g1	GE275856
61919812	CBYP1076.g1	GE275857
61919813	CBYP1077.g1	GE275858
61919814	CBYP1078.g1	GE275859
61919815	CBYP1079.g1	GE275860
61919816	CBYP1080.g1	GE275861
61919817	CBYP1081.g1	GE275862
61919818	CBYP1082.g1	GE275863
61919819	CBYP1083.g1	GE275864
61919820	CBYP1084.g1	GE275865
61919821	CBYP1085.g1	GE275866
61919822	CBYP1086.g1	GE275867
61919823	CBYP1087.g1	GE275868
61919824	CBYP1088.g1	GE275869
61919825	CBYP1089.g1	GE275870
61919826	CBYP1090.g1	GE275871
61919827	CBYP1091.g1	GE275872
61919828	CBYP1092.g1	GE275873
61919829	CBYP1093.g1	GE275874
61919830	CBYP1094.g1	GE275875
61919831	CBYP1095.g1	GE275876
61919832	CBYP1096.g1	GE275877
61919833	CBYP1098.g1	GE275878
61919834	CBYP1099.g1	GE275879
61919835	CBYP1100.g1	GE275880
61919836	CBYP1101.g1	GE275881
61919837	CBYP1104.g1	GE275882
61919838	CBYP1105.g1	GE275883
61919839	CBYP1106.g1	GE275884
61919840	CBYP1107.g1	GE275885
61919841	CBYP1109.g1	GE275886
61919842	CBYP1110.g1	GE275887
61919843	CBYP1111.g1	GE275888
61919844	CBYP1112.g1	GE275889
61919845	CBYP1113.g1	GE275890
61919846	CBYP1114.g1	GE275891
61919847	CBYP1115.g1	GE275892
61919848	CBYP1116.g1	GE275893
61919849	CBYP1117.g1	GE275894
61919850	CBYP1118.g1	GE275895
61919851	CBYP1119.g1	GE275896
61919852	CBYP1120.g1	GE275897
61919853	CBYP1121.g1	GE275898
61919854	CBYP1123.g1	GE275899
61919855	CBYP1124.g1	GE275900
61919856	CBYP1125.g1	GE275901
61919857	CBYP1126.g1	GE275902
61919858	CBYP1127.g1	GE275903
61919859	CBYP1128.g1	GE275904
61919860	CBYP1129.g1	GE275905
61919861	CBYP1130.g1	GE275906
61919862	CBYP1131.g1	GE275907
61919863	CBYP1132.g1	GE275908
61919864	CBYP1134.g1	GE275909
61919865	CBYP1135.g1	GE275910
61919866	CBYP1136.g1	GE275911
61919867	CBYP1137.g1	GE275912
61919868	CBYP1138.g1	GE275913

61919869	CBYP1139.g1	GE275914
61919870	CBYP1140.g1	GE275915
61919871	CBYP1141.g1	GE275916
61919872	CBYP1142.g1	GE275917
61919873	CBYP1143.g1	GE275918
61919874	CBYP1144.g1	GE275919
61919875	CBYP1145.g1	GE275920
61919876	CBYP1146.g1	GE275921
61919877	CBYP1147.g1	GE275922
61919878	CBYP1148.g1	GE275923
61919879	CBYP1149.g1	GE275924
61919880	CBYP1150.g1	GE275925
61919881	CBYP1153.b1	GE275926
61919882	CBYP1153.g1	GE275927
61919883	CBYP1154.b1	GE275928
61919884	CBYP1154.g1	GE275929
61919885	CBYP1155.b1	GE275930
61919886	CBYP1155.g1	GE275931
61919887	CBYP1156.b1	GE275932
61919888	CBYP1157.b1	GE275933
61919889	CBYP1157.g1	GE275934
61919890	CBYP1158.b1	GE275935
61919891	CBYP1158.g1	GE275936
61919892	CBYP1159.g1	GE275937
61919893	CBYP1160.b1	GE275938
61919894	CBYP1160.g1	GE275939
61919895	CBYP1161.b1	GE275940
61919896	CBYP1161.g1	GE275941
61919897	CBYP1162.b1	GE275942
61919898	CBYP1163.b1	GE275943
61919899	CBYP1163.g1	GE275944
61919900	CBYP1164.b1	GE275945
61919901	CBYP1164.g1	GE275946
61919902	CBYP1165.b1	GE275947
61919903	CBYP1165.g1	GE275948
61919904	CBYP1166.b1	GE275949
61919905	CBYP1166.g1	GE275950
61919906	CBYP1167.b1	GE275951
61919907	CBYP1167.g1	GE275952
61919908	CBYP1168.b1	GE275953
61919909	CBYP1169.b1	GE275954
61919910	CBYP1169.g1	GE275955
61919911	CBYP1170.b1	GE275956
61919912	CBYP1170.g1	GE275957
61919913	CBYP1172.g1	GE275958
61919914	CBYP1173.b1	GE275959
61919915	CBYP1173.g1	GE275960
61919916	CBYP1175.b1	GE275961
61919917	CBYP1175.g1	GE275962
61919918	CBYP1176.b1	GE275963
61919919	CBYP1176.g1	GE275964
61919920	CBYP1177.b1	GE275965
61919921	CBYP1179.b1	GE275966
61919922	CBYP1179.g1	GE275967
61919923	CBYP1180.b1	GE275968
61919924	CBYP1180.g1	GE275969
61919925	CBYP1181.b1	GE275970
61919926	CBYP1181.g1	GE275971
61919927	CBYP1182.b1	GE275972
61919928	CBYP1182.g1	GE275973
61919929	CBYP1183.g1	GE275974
61919930	CBYP1185.b1	GE275975
61919931	CBYP1185.g1	GE275976

61919932	CBYP1186.b1	GE275977
61919933	CBYP1186.g1	GE275978
61919934	CBYP1187.b1	GE275979
61919935	CBYP1187.g1	GE275980
61919936	CBYP1188.b1	GE275981
61919937	CBYP1188.g1	GE275982
61919938	CBYP1189.b1	GE275983
61919939	CBYP1189.g1	GE275984
61919940	CBYP1190.b1	GE275985
61919941	CBYP1190.g1	GE275986
61919942	CBYP1191.b1	GE275987
61919943	CBYP1191.g1	GE275988
61919944	CBYP1192.b1	GE275989
61919945	CBYP1192.g1	GE275990
61919946	CBYP1193.b1	GE275991
61919947	CBYP1193.g1	GE275992
61919948	CBYP1194.b1	GE275993
61919949	CBYP1194.g1	GE275994
61919950	CBYP1195.b1	GE275995
61919951	CBYP1195.g1	GE275996
61919952	CBYP1196.b1	GE275997
61919953	CBYP1196.g1	GE275998
61919954	CBYP1198.g1	GE275999
61919955	CBYP1199.g1	GE276000
61919956	CBYP1200.b1	GE276001
61919957	CBYP1200.g1	GE276002
61919958	CBYP1201.b1	GE276003
61919959	CBYP1201.g1	GE276004
61919960	CBYP1202.b1	GE276005
61919961	CBYP1202.g1	GE276006
61919962	CBYP1203.b1	GE276007
61919963	CBYP1203.g1	GE276008
61919964	CBYP1204.b1	GE276009
61919965	CBYP1204.g1	GE276010
61919966	CBYP1205.b1	GE276011
61919967	CBYP1205.g1	GE276012
61919968	CBYP1207.b1	GE276013
61919969	CBYP1207.g1	GE276014
61919970	CBYP1208.b1	GE276015
61919971	CBYP1208.g1	GE276016
61919972	CBYP1209.b1	GE276017
61919973	CBYP1209.g1	GE276018
61919974	CBYP1210.g1	GE276019
61919975	CBYP1211.b1	GE276020
61919976	CBYP1211.g1	GE276021
61919977	CBYP1212.b1	GE276022
61919978	CBYP1212.g1	GE276023
61919979	CBYP1213.g1	GE276024
61919980	CBYP1214.b1	GE276025
61919981	CBYP1214.g1	GE276026
61919982	CBYP1215.b1	GE276027
61919983	CBYP1216.b1	GE276028
61919984	CBYP1216.g1	GE276029
61919985	CBYP1217.b1	GE276030
61919986	CBYP1217.g1	GE276031
61919987	CBYP1218.b1	GE276032
61919988	CBYP1219.b1	GE276033
61919989	CBYP1219.g1	GE276034
61919990	CBYP1220.b1	GE276035
61919991	CBYP1221.b1	GE276036
61919992	CBYP1221.g1	GE276037
61919993	CBYP1222.b1	GE276038
61919994	CBYP1223.b1	GE276039

61919995	CBYP1223.g1	GE276040
61919996	CBYP1224.b1	GE276041
61919997	CBYP1224.g1	GE276042
61919998	CBYP1225.b1	GE276043
61919999	CBYP1225.g1	GE276044
61920000	CBYP1226.b1	GE276045
61920001	CBYP1226.g1	GE276046
61920002	CBYP1227.b1	GE276047
61920003	CBYP1227.g1	GE276048
61920004	CBYP1229.g1	GE276049
61920005	CBYP1230.b1	GE276050
61920006	CBYP1230.g1	GE276051
61920007	CBYP1231.b1	GE276052
61920008	CBYP1232.b1	GE276053
61920009	CBYP1232.g1	GE276054
61920010	CBYP1233.b1	GE276055
61920011	CBYP1234.b1	GE276056
61920012	CBYP1234.g1	GE276057
61920013	CBYP1235.b1	GE276058
61920014	CBYP1235.g1	GE276059
61920015	CBYP1237.b1	GE276060
61920016	CBYP1237.g1	GE276061
61920017	CBYP1238.b1	GE276062
61920018	CBYP1238.g1	GE276063
61920019	CBYP1239.b1	GE276064
61920020	CBYP1239.g1	GE276065
61920021	CBYP1240.b1	GE276066
61920022	CBYP1240.g1	GE276067
61920023	CBYP1241.b1	GE276068
61920024	CBYP1241.g1	GE276069
61920025	CBYP1242.b1	GE276070
61920026	CBYP1242.g1	GE276071
61920027	CBYP1243.b1	GE276072
61920028	CBYP1243.g1	GE276073
61920029	CBYP1244.b1	GE276074
61920030	CBYP1244.g1	GE276075
61920031	CBYP1245.b1	GE276076
61920032	CBYP1245.g1	GE276077
61920033	CBYP1246.b1	GE276078
61920034	CBYP1246.g1	GE276079
61920035	CBYP1248.b1	GE276080
61920036	CBYP1249.b1	GE276081
61920037	CBYP1250.b1	GE276082
61920038	CBYP1250.g1	GE276083
61920039	CBYP1251.b1	GE276084
61920040	CBYP1251.g1	GE276085
61920041	CBYP1252.b1	GE276086
61920042	CBYP1252.g1	GE276087
61920043	CBYP1253.b1	GE276088
61920044	CBYP1253.g1	GE276089
61920045	CBYP1254.b1	GE276090
61920046	CBYP1254.g1	GE276091
61920047	CBYP1255.b1	GE276092
61920048	CBYP1255.g1	GE276093
61920049	CBYP1256.b1	GE276094
61920050	CBYP1256.g1	GE276095
61920051	CBYP1257.b1	GE276096
61920052	CBYP1257.g1	GE276097
61920053	CBYP1258.b1	GE276098
61920054	CBYP1258.g1	GE276099
61920055	CBYP1259.b1	GE276100
61920056	CBYP1259.g1	GE276101
61920057	CBYP1260.b1	GE276102

61920058	CBYP1260.g1	GE276103
61920059	CBYP1261.b1	GE276104
61920060	CBYP1261.g1	GE276105
61920061	CBYP1262.b1	GE276106
61920062	CBYP1262.g1	GE276107
61920063	CBYP1263.b1	GE276108
61920064	CBYP1263.g1	GE276109
61920065	CBYP1264.b1	GE276110
61920066	CBYP1264.g1	GE276111
61920067	CBYP1265.b1	GE276112
61920068	CBYP1265.g1	GE276113
61920069	CBYP1266.b1	GE276114
61920070	CBYP1266.g1	GE276115
61920071	CBYP1267.b1	GE276116
61920072	CBYP1267.g1	GE276117
61920073	CBYP1268.b1	GE276118
61920074	CBYP1269.b1	GE276119
61920075	CBYP1269.g1	GE276120
61920076	CBYP1270.g1	GE276121
61920077	CBYP1271.b1	GE276122
61920078	CBYP1271.g1	GE276123
61920079	CBYP1272.b1	GE276124
61920080	CBYP1272.g1	GE276125
61920081	CBYP1273.b1	GE276126
61920082	CBYP1274.b1	GE276127
61920083	CBYP1274.g1	GE276128
61920084	CBYP1275.b1	GE276129
61920085	CBYP1275.g1	GE276130
61920086	CBYP1276.b1	GE276131
61920087	CBYP1276.g1	GE276132
61920088	CBYP1277.b1	GE276133
61920089	CBYP1277.g1	GE276134
61920090	CBYP1278.b1	GE276135
61920091	CBYP1278.g1	GE276136
61920092	CBYP1279.b1	GE276137
61920093	CBYP1279.g1	GE276138
61920094	CBYP1280.b1	GE276139
61920095	CBYP1280.g1	GE276140
61920096	CBYP1281.b1	GE276141
61920097	CBYP1281.g1	GE276142
61920098	CBYP1282.b1	GE276143
61920099	CBYP1282.g1	GE276144
61920100	CBYP1283.b1	GE276145
61920101	CBYP1283.g1	GE276146
61920102	CBYP1284.b1	GE276147
61920103	CBYP1284.g1	GE276148
61920104	CBYP1285.b1	GE276149
61920105	CBYP1285.g1	GE276150
61920106	CBYP1286.b1	GE276151
61920107	CBYP1286.g1	GE276152
61920108	CBYP1287.b1	GE276153
61920109	CBYP1287.g1	GE276154
61920110	CBYP1288.b1	GE276155
61920111	CBYP1288.g1	GE276156
61920112	CBYP1289.b1	GE276157
61920113	CBYP1289.g1	GE276158
61920114	CBYP1290.g1	GE276159
61920115	CBYP1291.b1	GE276160
61920116	CBYP1291.g1	GE276161
61920117	CBYP1293.b1	GE276162
61920118	CBYP1293.g1	GE276163
61920119	CBYP1294.g1	GE276164
61920120	CBYP1295.b1	GE276165

61920121	CBYP1295.g1	GE276166
61920122	CBYP1296.g1	GE276167
61920123	CBYP1297.b1	GE276168
61920124	CBYP1297.g1	GE276169
61920125	CBYP1298.b1	GE276170
61920126	CBYP1298.g1	GE276171
61920127	CBYP1299.b1	GE276172
61920128	CBYP1299.g1	GE276173
61920129	CBYP1300.b1	GE276174
61920130	CBYP1301.b1	GE276175
61920131	CBYP1301.g1	GE276176
61920132	CBYP1302.b1	GE276177
61920133	CBYP1302.g1	GE276178
61920134	CBYP1303.b1	GE276179
61920135	CBYP1303.g1	GE276180
61920136	CBYP1304.b1	GE276181
61920137	CBYP1304.g1	GE276182
61920138	CBYP1305.b1	GE276183
61920139	CBYP1305.g1	GE276184
61920140	CBYP1306.b1	GE276185
61920141	CBYP1306.g1	GE276186
61920142	CBYP1307.b1	GE276187
61920143	CBYP1308.b1	GE276188
61920144	CBYP1308.g1	GE276189
61920145	CBYP1309.b1	GE276190
61920146	CBYP1310.b1	GE276191
61920147	CBYP1310.g1	GE276192
61920148	CBYP1311.b1	GE276193
61920149	CBYP1311.g1	GE276194
61920150	CBYP1312.b1	GE276195
61920151	CBYP1313.b1	GE276196
61920152	CBYP1313.g1	GE276197
61920153	CBYP1314.b1	GE276198
61920154	CBYP1314.g1	GE276199
61920155	CBYP1315.b1	GE276200
61920156	CBYP1315.g1	GE276201
61920157	CBYP1316.b1	GE276202
61920158	CBYP1316.g1	GE276203
61920159	CBYP1317.b1	GE276204
61920160	CBYP1317.g1	GE276205
61920161	CBYP1318.b1	GE276206
61920162	CBYP1318.g1	GE276207
61920163	CBYP1319.b1	GE276208
61920164	CBYP1319.g1	GE276209
61920165	CBYP1320.b1	GE276210
61920166	CBYP1320.g1	GE276211
61920167	CBYP1321.b1	GE276212
61920168	CBYP1321.g1	GE276213
61920169	CBYP1322.b1	GE276214
61920170	CBYP1322.g1	GE276215
61920171	CBYP1323.b1	GE276216
61920172	CBYP1323.g1	GE276217
61920173	CBYP1324.b1	GE276218
61920174	CBYP1324.g1	GE276219
61920175	CBYP1325.b1	GE276220
61920176	CBYP1325.g1	GE276221
61920177	CBYP1326.b1	GE276222
61920178	CBYP1326.g1	GE276223
61920179	CBYP1327.b1	GE276224
61920180	CBYP1327.g1	GE276225
61920181	CBYP1328.b1	GE276226
61920182	CBYP1328.g1	GE276227
61920183	CBYP1329.b1	GE276228

61920184	CBYP1329.g1	GE276229
61920185	CBYP1330.b1	GE276230
61920186	CBYP1330.g1	GE276231
61920187	CBYP1331.b1	GE276232
61920188	CBYP1331.g1	GE276233
61920189	CBYP1332.b1	GE276234
61920190	CBYP1332.g1	GE276235
61920191	CBYP1333.b1	GE276236
61920192	CBYP1333.g1	GE276237
61920193	CBYP1334.b1	GE276238
61920194	CBYP1334.g1	GE276239
61920195	CBYP1335.b1	GE276240
61920196	CBYP1335.g1	GE276241
61920197	CBYP1336.b1	GE276242
61920198	CBYP1336.g1	GE276243
61920199	CBYP1337.g1	GE276244
61920200	CBYP1338.b1	GE276245
61920201	CBYP1338.g1	GE276246
61920202	CBYP1339.b1	GE276247
61920203	CBYP1339.g1	GE276248
61920204	CBYP1340.b1	GE276249
61920205	CBYP1340.g1	GE276250
61920206	CBYP1342.b1	GE276251
61920207	CBYP1342.g1	GE276252
61920208	CBYP1343.b1	GE276253
61920209	CBYP1343.g1	GE276254
61920210	CBYP1344.b1	GE276255
61920211	CBYP1344.g1	GE276256
61920212	CBYP1345.g1	GE276257
61920213	CBYP1346.b1	GE276258
61920214	CBYP1346.g1	GE276259
61920215	CBYP1347.b1	GE276260
61920216	CBYP1347.g1	GE276261
61920217	CBYP1348.b1	GE276262
61920218	CBYP1349.b1	GE276263
61920219	CBYP1349.g1	GE276264
61920220	CBYP1350.b1	GE276265
61920221	CBYP1350.g1	GE276266
61920222	CBYP1351.g1	GE276267
61920223	CBYP1352.b1	GE276268
61920224	CBYP1352.g1	GE276269
61920225	CBYP1353.b1	GE276270
61920226	CBYP1353.g1	GE276271
61920227	CBYP1354.b1	GE276272
61920228	CBYP1354.g1	GE276273
61920229	CBYP1355.b1	GE276274
61920230	CBYP1355.g1	GE276275
61920231	CBYP1356.b1	GE276276
61920232	CBYP1356.g1	GE276277
61920233	CBYP1357.b1	GE276278
61920234	CBYP1358.b1	GE276279
61920235	CBYP1358.g1	GE276280
61920236	CBYP1360.b1	GE276281
61920237	CBYP1360.g1	GE276282
61920238	CBYP1361.b1	GE276283
61920239	CBYP1361.g1	GE276284
61920240	CBYP1362.b1	GE276285
61920241	CBYP1362.g1	GE276286
61920242	CBYP1363.b1	GE276287
61920243	CBYP1363.g1	GE276288
61920244	CBYP1364.b1	GE276289
61920245	CBYP1364.g1	GE276290
61920246	CBYP1365.b1	GE276291

61920247	CBYP1365.g1	GE276292
61920248	CBYP1367.b1	GE276293
61920249	CBYP1368.b1	GE276294
61920250	CBYP1368.g1	GE276295
61920251	CBYP1369.b1	GE276296
61920252	CBYP1369.g1	GE276297
61920253	CBYP1370.b1	GE276298
61920254	CBYP1370.g1	GE276299
61920255	CBYP1371.b1	GE276300
61920256	CBYP1371.g1	GE276301
61920257	CBYP1372.b1	GE276302
61920258	CBYP1372.g1	GE276303
61920259	CBYP1373.b1	GE276304
61920260	CBYP1373.g1	GE276305
61920261	CBYP1374.b1	GE276306
61920262	CBYP1374.g1	GE276307
61920263	CBYP1375.b1	GE276308
61920264	CBYP1375.g1	GE276309
61920265	CBYP1376.b1	GE276310
61920266	CBYP1376.g1	GE276311
61920267	CBYP1377.b1	GE276312
61920268	CBYP1378.b1	GE276313
61920269	CBYP1378.g1	GE276314
61920270	CBYP1379.g1	GE276315
61920271	CBYP1380.b1	GE276316
61920272	CBYP1380.g1	GE276317
61920273	CBYP1381.b1	GE276318
61920274	CBYP1381.g1	GE276319
61920275	CBYP1382.b1	GE276320
61920276	CBYP1383.b1	GE276321
61920277	CBYP1383.g1	GE276322
61920278	CBYP1385.b1	GE276323
61920279	CBYP1385.g1	GE276324
61920280	CBYP1386.b1	GE276325
61920281	CBYP1386.g1	GE276326
61920282	CBYP1387.b1	GE276327
61920283	CBYP1388.b1	GE276328
61920284	CBYP1388.g1	GE276329
61920285	CBYP1389.b1	GE276330
61920286	CBYP1389.g1	GE276331
61920287	CBYP1390.b1	GE276332
61920288	CBYP1391.b1	GE276333
61920289	CBYP1391.g1	GE276334
61920290	CBYP1392.b1	GE276335
61920291	CBYP1392.g1	GE276336
61920292	CBYP1393.b1	GE276337
61920293	CBYP1393.g1	GE276338
61920294	CBYP1394.b1	GE276339
61920295	CBYP1394.g1	GE276340
61920296	CBYP1395.b1	GE276341
61920297	CBYP1395.g1	GE276342
61920298	CBYP1396.g1	GE276343
61920299	CBYP1397.b1	GE276344
61920300	CBYP1397.g1	GE276345
61920301	CBYP1398.b1	GE276346
61920302	CBYP1398.g1	GE276347
61920303	CBYP1399.b1	GE276348
61920304	CBYP1399.g1	GE276349
61920305	CBYP1400.b1	GE276350
61920306	CBYP1400.g1	GE276351
61920307	CBYP1401.b1	GE276352
61920308	CBYP1401.g1	GE276353
61920309	CBYP1403.b1	GE276354

61920310	CBYP1404.b1	GE276355
61920311	CBYP1404.g1	GE276356
61920312	CBYP1405.b1	GE276357
61920313	CBYP1405.g1	GE276358
61920314	CBYP1406.b1	GE276359
61920315	CBYP1406.g1	GE276360
61920316	CBYP1407.b1	GE276361
61920317	CBYP1407.g1	GE276362
61920318	CBYP1408.b1	GE276363
61920319	CBYP1408.g1	GE276364
61920320	CBYP1409.b1	GE276365
61920321	CBYP1409.g1	GE276366
61920322	CBYP1410.b1	GE276367
61920323	CBYP1412.b1	GE276368
61920324	CBYP1414.b1	GE276369
61920325	CBYP1414.g1	GE276370
61920326	CBYP1415.g1	GE276371
61920327	CBYP1416.g1	GE276372
61920328	CBYP1417.b1	GE276373
61920329	CBYP1417.g1	GE276374
61920330	CBYP1418.b1	GE276375
61920331	CBYP1419.b1	GE276376
61920332	CBYP1419.g1	GE276377
61920333	CBYP1420.g1	GE276378
61920334	CBYP1421.b1	GE276379
61920335	CBYP1421.g1	GE276380
61920336	CBYP1423.b1	GE276381
61920337	CBYP1423.g1	GE276382
61920338	CBYP1424.b1	GE276383
61920339	CBYP1424.g1	GE276384
61920340	CBYP1425.b1	GE276385
61920341	CBYP1425.g1	GE276386
61920342	CBYP1426.b1	GE276387
61920343	CBYP1426.g1	GE276388
61920344	CBYP1428.b1	GE276389
61920345	CBYP1428.g1	GE276390
61920346	CBYP1429.g1	GE276391
61920347	CBYP1430.g1	GE276392
61920348	CBYP1431.b1	GE276393
61920349	CBYP1431.g1	GE276394
61920350	CBYP1432.b1	GE276395
61920351	CBYP1432.g1	GE276396
61920352	CBYP1433.b1	GE276397
61920353	CBYP1433.g1	GE276398
61920354	CBYP1434.b1	GE276399
61920355	CBYP1434.g1	GE276400
61920356	CBYP1435.b1	GE276401
61920357	CBYP1435.g1	GE276402
61920358	CBYP1436.b1	GE276403
61920359	CBYP1436.g1	GE276404
61920360	CBYP1437.b1	GE276405
61920361	CBYP1437.g1	GE276406
61920362	CBYP1438.b1	GE276407
61920363	CBYP1438.g1	GE276408
61920364	CBYP1439.b1	GE276409
61920365	CBYP1439.g1	GE276410
61920366	CBYP1440.b1	GE276411
61920367	CBYP1440.g1	GE276412
61920368	CBYP1441.b1	GE276413
61920369	CBYP1441.g1	GE276414
61920370	CBYP1443.b1	GE276415
61920371	CBYP1443.g1	GE276416
61920372	CBYP1444.b1	GE276417

61920373	CBYP1444.g1	GE276418
61920374	CBYP1445.b1	GE276419
61920375	CBYP1446.b1	GE276420
61920376	CBYP1446.g1	GE276421
61920377	CBYP1447.b1	GE276422
61920378	CBYP1447.g1	GE276423
61920379	CBYP1448.b1	GE276424
61920380	CBYP1449.b1	GE276425
61920381	CBYP1449.g1	GE276426
61920382	CBYP1450.b1	GE276427
61920383	CBYP1450.g1	GE276428
61920384	CBYP1451.b1	GE276429
61920385	CBYP1455.b1	GE276430
61920386	CBYP1455.g1	GE276431
61920387	CBYP1456.b1	GE276432
61920388	CBYP1456.g1	GE276433
61920389	CBYP1457.g1	GE276434
61920390	CBYP1458.b1	GE276435
61920391	CBYP1458.g1	GE276436
61920392	CBYP1459.b1	GE276437
61920393	CBYP1459.g1	GE276438
61920394	CBYP1460.b1	GE276439
61920395	CBYP1460.g1	GE276440
61920396	CBYP1461.g1	GE276441
61920397	CBYP1462.b1	GE276442
61920398	CBYP1462.g1	GE276443
61920399	CBYP1463.b1	GE276444
61920400	CBYP1463.g1	GE276445
61920401	CBYP1466.b1	GE276446
61920402	CBYP1466.g1	GE276447
61920403	CBYP1467.b1	GE276448
61920404	CBYP1467.g1	GE276449
61920405	CBYP1468.b1	GE276450
61920406	CBYP1468.g1	GE276451
61920407	CBYP1469.b1	GE276452
61920408	CBYP1469.g1	GE276453
61920409	CBYP1470.b1	GE276454
61920410	CBYP1470.g1	GE276455
61920411	CBYP1471.b1	GE276456
61920412	CBYP1471.g1	GE276457
61920413	CBYP1472.b1	GE276458
61920414	CBYP1472.g1	GE276459
61920415	CBYP1473.b1	GE276460
61920416	CBYP1473.g1	GE276461
61920417	CBYP1474.b1	GE276462
61920418	CBYP1474.g1	GE276463
61920419	CBYP1475.b1	GE276464
61920420	CBYP1475.g1	GE276465
61920421	CBYP1476.b1	GE276466
61920422	CBYP1476.g1	GE276467
61920423	CBYP1477.b1	GE276468
61920424	CBYP1477.g1	GE276469
61920425	CBYP1478.b1	GE276470
61920426	CBYP1479.b1	GE276471
61920427	CBYP1479.g1	GE276472
61920428	CBYP1480.b1	GE276473
61920429	CBYP1480.g1	GE276474
61920430	CBYP1481.b1	GE276475
61920431	CBYP1482.b1	GE276476
61920432	CBYP1482.g1	GE276477
61920433	CBYP1483.b1	GE276478
61920434	CBYP1483.g1	GE276479
61920435	CBYP1484.b1	GE276480

61920436	CBYP1484.g1	GE276481
61920437	CBYP1485.b1	GE276482
61920438	CBYP1485.g1	GE276483
61920439	CBYP1486.b1	GE276484
61920440	CBYP1486.g1	GE276485
61920441	CBYP1487.b1	GE276486
61920442	CBYP1487.g1	GE276487
61920443	CBYP1488.b1	GE276488
61920444	CBYP1488.g1	GE276489
61920445	CBYP1489.b1	GE276490
61920446	CBYP1489.g1	GE276491
61920447	CBYP1490.b1	GE276492
61920448	CBYP1490.g1	GE276493
61920449	CBYP1491.b1	GE276494
61920450	CBYP1492.g1	GE276495
61920451	CBYP1493.b1	GE276496
61920452	CBYP1494.b1	GE276497
61920453	CBYP1494.g1	GE276498
61920454	CBYP1495.b1	GE276499
61920455	CBYP1495.g1	GE276500
61920456	CBYP1496.b1	GE276501
61920457	CBYP1496.g1	GE276502
61920458	CBYP1497.b1	GE276503
61920459	CBYP1497.g1	GE276504
61920460	CBYP1498.b1	GE276505
61920461	CBYP1498.g1	GE276506
61920462	CBYP1499.b1	GE276507
61920463	CBYP1499.g1	GE276508
61920464	CBYP1500.b1	GE276509
61920465	CBYP1500.g1	GE276510
61920466	CBYP1501.g1	GE276511
61920467	CBYP1502.b1	GE276512
61920468	CBYP1502.g1	GE276513
61920469	CBYP1503.b1	GE276514
61920470	CBYP1503.g1	GE276515
61920471	CBYP1504.g1	GE276516
61920472	CBYP1505.b1	GE276517
61920473	CBYP1505.g1	GE276518
61920474	CBYP1506.b1	GE276519
61920475	CBYP1506.g1	GE276520
61920476	CBYP1507.b1	GE276521
61920477	CBYP1507.g1	GE276522
61920478	CBYP1508.b1	GE276523
61920479	CBYP1509.b1	GE276524
61920480	CBYP1509.g1	GE276525
61920481	CBYP1510.b1	GE276526
61920482	CBYP1510.g1	GE276527
61920483	CBYP1511.b1	GE276528
61920484	CBYP1514.b1	GE276529
61920485	CBYP1514.g1	GE276530
61920486	CBYP1515.b1	GE276531
61920487	CBYP1515.g1	GE276532
61920488	CBYP1516.b1	GE276533
61920489	CBYP1516.g1	GE276534
61920490	CBYP1517.b1	GE276535
61920491	CBYP1517.g1	GE276536
61920492	CBYP1518.b1	GE276537
61920493	CBYP1518.g1	GE276538
61920494	CBYP1520.b1	GE276539
61920495	CBYP1520.g1	GE276540
61920496	CBYP1521.b1	GE276541
61920497	CBYP1521.g1	GE276542
61920498	CBYP1522.b1	GE276543

61920499	CBYP1522.g1	GE276544
61920500	CBYP1523.b1	GE276545
61920501	CBYP1523.g1	GE276546
61920502	CBYP1524.b1	GE276547
61920503	CBYP1524.g1	GE276548
61920504	CBYP1525.b1	GE276549
61920505	CBYP1525.g1	GE276550
61920506	CBYP1527.b1	GE276551
61920507	CBYP1527.g1	GE276552
61920508	CBYP1528.b1	GE276553
61920509	CBYP1528.g1	GE276554
61920510	CBYP1529.b1	GE276555
61920511	CBYP1529.g1	GE276556
61920512	CBYP1530.b1	GE276557
61920513	CBYP1530.g1	GE276558
61920514	CBYP1531.b1	GE276559
61920515	CBYP1532.b1	GE276560
61920516	CBYP1532.g1	GE276561
61920517	CBYP1533.b1	GE276562
61920518	CBYP1533.g1	GE276563
61920519	CBYP1534.b1	GE276564
61920520	CBYP1534.g1	GE276565
61920521	CBYP1535.b1	GE276566
61920522	CBYP1535.g1	GE276567
61920523	CBYP1537.b1	GE276568
61920524	CBYP1537.g1	GE276569
61920525	CBYP1538.b1	GE276570
61920526	CBYP1538.g1	GE276571
61920527	CBYP1539.b1	GE276572
61920528	CBYP1539.g1	GE276573
61920529	CBYP1540.g1	GE276574
61920530	CBYP1541.b1	GE276575
61920531	CBYP1541.g1	GE276576
61920532	CBYP1542.b1	GE276577
61920533	CBYP1542.g1	GE276578
61920534	CBYP1543.b1	GE276579
61920535	CBYP1543.g1	GE276580
61920536	CBYP1544.b1	GE276581
61920537	CBYP1544.g1	GE276582
61920538	CBYP1545.b1	GE276583
61920539	CBYP1545.g1	GE276584
61920540	CBYP1546.b1	GE276585
61920541	CBYP1546.g1	GE276586
61920542	CBYP1547.b1	GE276587
61920543	CBYP1547.g1	GE276588
61920544	CBYP1549.b1	GE276589
61920545	CBYP1549.g1	GE276590
61920546	CBYP1550.g1	GE276591
61920547	CBYP1551.b1	GE276592
61920548	CBYP1551.g1	GE276593
61920549	CBYP1552.b1	GE276594
61920550	CBYP1552.g1	GE276595
61920551	CBYP1553.b1	GE276596
61920552	CBYP1553.g1	GE276597
61920553	CBYP1554.b1	GE276598
61920554	CBYP1554.g1	GE276599
61920555	CBYP1555.b1	GE276600
61920556	CBYP1555.g1	GE276601
61920557	CBYP1556.b1	GE276602
61920558	CBYP1556.g1	GE276603
61920559	CBYP1557.b1	GE276604
61920560	CBYP1558.b1	GE276605
61920561	CBYP1558.g1	GE276606

61920562	CBYP1559.b1	GE276607
61920563	CBYP1560.b1	GE276608
61920564	CBYP1560.g1	GE276609
61920565	CBYP1562.b1	GE276610
61920566	CBYP1562.g1	GE276611
61920567	CBYP1563.b1	GE276612
61920568	CBYP1563.g1	GE276613
61920569	CBYP1565.b1	GE276614
61920570	CBYP1565.g1	GE276615
61920571	CBYP1566.b1	GE276616
61920572	CBYP1566.g1	GE276617
61920573	CBYP1567.b1	GE276618
61920574	CBYP1567.g1	GE276619
61920575	CBYP1568.b1	GE276620
61920576	CBYP1568.g1	GE276621
61920577	CBYP1569.b1	GE276622
61920578	CBYP1569.g1	GE276623
61920579	CBYP1570.b1	GE276624
61920580	CBYP1570.g1	GE276625
61920581	CBYP1572.b1	GE276626
61920582	CBYP1573.b1	GE276627
61920583	CBYP1574.b1	GE276628
61920584	CBYP1574.g1	GE276629
61920585	CBYP1575.g1	GE276630
61920586	CBYP1576.b1	GE276631
61920587	CBYP1576.g1	GE276632
61920588	CBYP1577.b1	GE276633
61920589	CBYP1578.b1	GE276634
61920590	CBYP1578.g1	GE276635
61920591	CBYP1579.b1	GE276636
61920592	CBYP1579.g1	GE276637
61920593	CBYP1580.b1	GE276638
61920594	CBYP1580.g1	GE276639
61920595	CBYP1581.b1	GE276640
61920596	CBYP1581.g1	GE276641
61920597	CBYP1582.b1	GE276642
61920598	CBYP1582.g1	GE276643
61920599	CBYP1583.b1	GE276644
61920600	CBYP1583.g1	GE276645
61920601	CBYP1584.b1	GE276646
61920602	CBYP1584.g1	GE276647
61920603	CBYP1585.b1	GE276648
61920604	CBYP1585.g1	GE276649
61920605	CBYP1586.b1	GE276650
61920606	CBYP1587.b1	GE276651
61920607	CBYP1587.g1	GE276652
61920608	CBYP1588.b1	GE276653
61920609	CBYP1588.g1	GE276654
61920610	CBYP1590.b1	GE276655
61920611	CBYP1590.g1	GE276656
61920612	CBYP1591.g1	GE276657
61920613	CBYP1592.b1	GE276658
61920614	CBYP1592.g1	GE276659
61920615	CBYP1593.b1	GE276660
61920616	CBYP1593.g1	GE276661
61920617	CBYP1594.b1	GE276662
61920618	CBYP1594.g1	GE276663
61920619	CBYP1595.b1	GE276664
61920620	CBYP1595.g1	GE276665
61920621	CBYP1596.b1	GE276666
61920622	CBYP1596.g1	GE276667
61920623	CBYP1597.b1	GE276668
61920624	CBYP1597.g1	GE276669

61920625	CBYP1598.g1	GE276670
61920626	CBYP1600.g1	GE276671
61920627	CBYP1601.b1	GE276672
61920628	CBYP1601.g1	GE276673
61920629	CBYP1602.b1	GE276674
61920630	CBYP1602.g1	GE276675
61920631	CBYP1603.b1	GE276676
61920632	CBYP1603.g1	GE276677
61920633	CBYP1604.b1	GE276678
61920634	CBYP1604.g1	GE276679
61920635	CBYP1605.b1	GE276680
61920636	CBYP1605.g1	GE276681
61920637	CBYP1606.b1	GE276682
61920638	CBYP1606.g1	GE276683
61920639	CBYP1607.b1	GE276684
61920640	CBYP1607.g1	GE276685
61920641	CBYP1608.b1	GE276686
61920642	CBYP1608.g1	GE276687
61920643	CBYP1609.b1	GE276688
61920644	CBYP1609.g1	GE276689
61920645	CBYP1610.b1	GE276690
61920646	CBYP1610.g1	GE276691
61920647	CBYP1611.b1	GE276692
61920648	CBYP1611.g1	GE276693
61920649	CBYP1612.b1	GE276694
61920650	CBYP1612.g1	GE276695
61920651	CBYP1613.b1	GE276696
61920652	CBYP1613.g1	GE276697
61920653	CBYP1614.b1	GE276698
61920654	CBYP1614.g1	GE276699
61920655	CBYP1615.b1	GE276700
61920656	CBYP1615.g1	GE276701
61920657	CBYP1616.b1	GE276702
61920658	CBYP1616.g1	GE276703
61920659	CBYP1617.b1	GE276704
61920660	CBYP1617.g1	GE276705
61920661	CBYP1618.b1	GE276706
61920662	CBYP1618.g1	GE276707
61920663	CBYP1619.b1	GE276708
61920664	CBYP1619.g1	GE276709
61920665	CBYP1620.b1	GE276710
61920666	CBYP1620.g1	GE276711
61920667	CBYP1622.b1	GE276712
61920668	CBYP1622.g1	GE276713
61920669	CBYP1623.b1	GE276714
61920670	CBYP1623.g1	GE276715
61920671	CBYP1624.b1	GE276716
61920672	CBYP1624.g1	GE276717
61920673	CBYP1625.b1	GE276718
61920674	CBYP1626.b1	GE276719
61920675	CBYP1626.g1	GE276720
61920676	CBYP1627.b1	GE276721
61920677	CBYP1627.g1	GE276722
61920678	CBYP1628.b1	GE276723
61920679	CBYP1628.g1	GE276724
61920680	CBYP1629.b1	GE276725
61920681	CBYP1629.g1	GE276726
61920682	CBYP1630.b1	GE276727
61920683	CBYP1630.g1	GE276728
61920684	CBYP1631.b1	GE276729
61920685	CBYP1631.g1	GE276730
61920686	CBYP1632.b1	GE276731
61920687	CBYP1633.b1	GE276732

61920688	CBYP1633.g1	GE276733
61920689	CBYP1634.b1	GE276734
61920690	CBYP1634.g1	GE276735
61920691	CBYP1635.b1	GE276736
61920692	CBYP1635.g1	GE276737
61920693	CBYP1636.b1	GE276738
61920694	CBYP1636.g1	GE276739
61920695	CBYP1637.b1	GE276740
61920696	CBYP1637.g1	GE276741
61920697	CBYP1638.b1	GE276742
61920698	CBYP1638.g1	GE276743
61920699	CBYP1639.b1	GE276744
61920700	CBYP1639.g1	GE276745
61920701	CBYP1640.b1	GE276746
61920702	CBYP1640.g1	GE276747
61920703	CBYP1641.b1	GE276748
61920704	CBYP1641.g1	GE276749
61920705	CBYP1642.b1	GE276750
61920706	CBYP1642.g1	GE276751
61920707	CBYP1644.b1	GE276752
61920708	CBYP1644.g1	GE276753
61920709	CBYP1645.b1	GE276754
61920710	CBYP1645.g1	GE276755
61920711	CBYP1646.b1	GE276756
61920712	CBYP1646.g1	GE276757
61920713	CBYP1647.b1	GE276758
61920714	CBYP1647.g1	GE276759
61920715	CBYP1648.b1	GE276760
61920716	CBYP1648.g1	GE276761
61920717	CBYP1649.b1	GE276762
61920718	CBYP1649.g1	GE276763
61920719	CBYP1650.b1	GE276764
61920720	CBYP1650.g1	GE276765
61920721	CBYP1651.b1	GE276766
61920722	CBYP1651.g1	GE276767
61920723	CBYP1652.b1	GE276768
61920724	CBYP1652.g1	GE276769
61920725	CBYP1653.b1	GE276770
61920726	CBYP1653.g1	GE276771
61920727	CBYP1654.b1	GE276772
61920728	CBYP1654.g1	GE276773
61920729	CBYP1655.b1	GE276774
61920730	CBYP1655.g1	GE276775
61920731	CBYP1656.b1	GE276776
61920732	CBYP1656.g1	GE276777
61920733	CBYP1657.b1	GE276778
61920734	CBYP1657.g1	GE276779
61920735	CBYP1659.g1	GE276780
61920736	CBYP1660.b1	GE276781
61920737	CBYP1660.g1	GE276782
61920738	CBYP1661.b1	GE276783
61920739	CBYP1661.g1	GE276784
61920740	CBYP1662.b1	GE276785
61920741	CBYP1662.g1	GE276786
61920742	CBYP1663.b1	GE276787
61920743	CBYP1663.g1	GE276788
61920744	CBYP1664.b1	GE276789
61920745	CBYP1665.b1	GE276790
61920746	CBYP1665.g1	GE276791
61920747	CBYP1667.b1	GE276792
61920748	CBYP1667.g1	GE276793
61920749	CBYP1668.b1	GE276794
61920750	CBYP1668.g1	GE276795

61920751	CBYP1669.b1	GE276796
61920752	CBYP1669.g1	GE276797
61920753	CBYP1670.b1	GE276798
61920754	CBYP1670.g1	GE276799
61920755	CBYP1671.b1	GE276800
61920756	CBYP1671.g1	GE276801
61920757	CBYP1672.b1	GE276802
61920758	CBYP1672.g1	GE276803
61920759	CBYP1673.b1	GE276804
61920760	CBYP1673.g1	GE276805
61920761	CBYP1674.b1	GE276806
61920762	CBYP1674.g1	GE276807
61920763	CBYP1675.b1	GE276808
61920764	CBYP1675.g1	GE276809
61920765	CBYP1676.b1	GE276810
61920766	CBYP1676.g1	GE276811
61920767	CBYP1677.b1	GE276812
61920768	CBYP1677.g1	GE276813
61920769	CBYP1678.b1	GE276814
61920770	CBYP1678.g1	GE276815
61920771	CBYP1679.b1	GE276816
61920772	CBYP1679.g1	GE276817
61920773	CBYP1680.b1	GE276818
61920774	CBYP1680.g1	GE276819
61920775	CBYP1681.b1	GE276820
61920776	CBYP1681.g1	GE276821
61920777	CBYP1683.b1	GE276822
61920778	CBYP1683.g1	GE276823
61920779	CBYP1684.b1	GE276824
61920780	CBYP1684.g1	GE276825
61920781	CBYP1685.b1	GE276826
61920782	CBYP1685.g1	GE276827
61920783	CBYP1686.b1	GE276828
61920784	CBYP1686.g1	GE276829
61920785	CBYP1687.b1	GE276830
61920786	CBYP1687.g1	GE276831
61920787	CBYP1688.b1	GE276832
61920788	CBYP1689.b1	GE276833
61920789	CBYP1689.g1	GE276834
61920790	CBYP1690.b1	GE276835
61920791	CBYP1690.g1	GE276836
61920792	CBYP1691.b1	GE276837
61920793	CBYP1691.g1	GE276838
61920794	CBYP1692.b1	GE276839
61920795	CBYP1692.g1	GE276840
61920796	CBYP1693.b1	GE276841
61920797	CBYP1693.g1	GE276842
61920798	CBYP1694.b1	GE276843
61920799	CBYP1694.g1	GE276844
61920800	CBYP1695.b1	GE276845
61920801	CBYP1695.g1	GE276846
61920802	CBYP1696.b1	GE276847
61920803	CBYP1696.g1	GE276848
61920804	CBYP1697.b1	GE276849
61920805	CBYP1697.g1	GE276850
61920806	CBYP1698.b1	GE276851
61920807	CBYP1698.g1	GE276852
61920808	CBYP1699.g1	GE276853
61920809	CBYP1700.b1	GE276854
61920810	CBYP1700.g1	GE276855
61920811	CBYP1701.b1	GE276856
61920812	CBYP1701.g1	GE276857
61920813	CBYP1702.b1	GE276858

61920814	CBYP1702.g1	GE276859
61920815	CBYP1703.b1	GE276860
61920816	CBYP1703.g1	GE276861
61920817	CBYP1704.b1	GE276862
61920818	CBYP1704.g1	GE276863
61920819	CBYP1705.b1	GE276864
61920820	CBYP1705.g1	GE276865
61920821	CBYP1707.b1	GE276866
61920822	CBYP1707.g1	GE276867
61920823	CBYP1708.b1	GE276868
61920824	CBYP1708.g1	GE276869
61920825	CBYP1709.b1	GE276870
61920826	CBYP1709.g1	GE276871
61920827	CBYP1710.b1	GE276872
61920828	CBYP1710.g1	GE276873
61920829	CBYP1711.g1	GE276874
61920830	CBYP1712.b1	GE276875
61920831	CBYP1712.g1	GE276876
61920832	CBYP1713.b1	GE276877
61920833	CBYP1713.g1	GE276878
61920834	CBYP1714.b1	GE276879
61920835	CBYP1714.g1	GE276880
61920836	CBYP1715.b1	GE276881
61920837	CBYP1715.g1	GE276882
61920838	CBYP1716.b1	GE276883
61920839	CBYP1716.g1	GE276884
61920840	CBYP1717.b1	GE276885
61920841	CBYP1717.g1	GE276886
61920842	CBYP1718.b1	GE276887
61920843	CBYP1718.g1	GE276888
61920844	CBYP1719.b1	GE276889
61920845	CBYP1719.g1	GE276890
61920846	CBYP1720.b1	GE276891
61920847	CBYP1720.g1	GE276892
61920848	CBYP1721.b1	GE276893
61920849	CBYP1721.g1	GE276894
61920850	CBYP1722.b1	GE276895
61920851	CBYP1722.g1	GE276896
61920852	CBYP1723.b1	GE276897
61920853	CBYP1723.g1	GE276898
61920854	CBYP1724.b1	GE276899
61920855	CBYP1724.g1	GE276900
61920856	CBYP1725.b1	GE276901
61920857	CBYP1725.g1	GE276902
61920858	CBYP1726.b1	GE276903
61920859	CBYP1726.g1	GE276904
61920860	CBYP1727.b1	GE276905
61920861	CBYP1728.b1	GE276906
61920862	CBYP1728.g1	GE276907
61920863	CBYP1729.b1	GE276908
61920864	CBYP1729.g1	GE276909
61920865	CBYP1730.b1	GE276910
61920866	CBYP1730.g1	GE276911
61920867	CBYP1731.b1	GE276912
61920868	CBYP1731.g1	GE276913
61920869	CBYP1732.b1	GE276914
61920870	CBYP1732.g1	GE276915
61920871	CBYP1733.g1	GE276916
61920872	CBYP1734.b1	GE276917
61920873	CBYP1734.g1	GE276918
61920874	CBYP1735.b1	GE276919
61920875	CBYP1735.g1	GE276920
61920876	CBYP1736.b1	GE276921

61920877	CBYP1736.g1	GE276922
61920878	CBYP1737.b1	GE276923
61920879	CBYP1737.g1	GE276924
61920880	CBYP1738.b1	GE276925
61920881	CBYP1738.g1	GE276926
61920882	CBYP1739.b1	GE276927
61920883	CBYP1739.g1	GE276928
61920884	CBYP1740.b1	GE276929
61920885	CBYP1740.g1	GE276930
61920886	CBYP1741.b1	GE276931
61920887	CBYP1741.g1	GE276932
61920888	CBYP1742.b1	GE276933
61920889	CBYP1742.g1	GE276934
61920890	CBYP1743.b1	GE276935
61920891	CBYP1743.g1	GE276936
61920892	CBYP1744.b1	GE276937
61920893	CBYP1744.g1	GE276938
61920894	CBYP1745.b1	GE276939
61920895	CBYP1745.g1	GE276940
61920896	CBYP1746.b1	GE276941
61920897	CBYP1746.g1	GE276942
61920898	CBYP1747.b1	GE276943
61920899	CBYP1747.g1	GE276944
61920900	CBYP1748.b1	GE276945
61920901	CBYP1748.g1	GE276946
61920902	CBYP1749.b1	GE276947
61920903	CBYP1749.g1	GE276948
61920904	CBYP1751.b1	GE276949
61920905	CBYP1751.g1	GE276950
61920906	CBYP1752.b1	GE276951
61920907	CBYP1752.g1	GE276952
61920908	CBYP1753.b1	GE276953
61920909	CBYP1753.g1	GE276954
61920910	CBYP1754.b1	GE276955
61920911	CBYP1754.g1	GE276956
61920912	CBYP1755.b1	GE276957
61920913	CBYP1755.g1	GE276958
61920914	CBYP1756.b1	GE276959
61920915	CBYP1756.g1	GE276960
61920916	CBYP1757.g1	GE276961
61920917	CBYP1758.b1	GE276962
61920918	CBYP1758.g1	GE276963
61920919	CBYP1759.b1	GE276964
61920920	CBYP1759.g1	GE276965
61920921	CBYP1760.b1	GE276966
61920922	CBYP1760.g1	GE276967
61920923	CBYP1761.b1	GE276968
61920924	CBYP1761.g1	GE276969
61920925	CBYP1762.b1	GE276970
61920926	CBYP1762.g1	GE276971
61920927	CBYP1763.b1	GE276972
61920928	CBYP1763.g1	GE276973
61920929	CBYP1764.b1	GE276974
61920930	CBYP1764.g1	GE276975
61920931	CBYP1765.b1	GE276976
61920932	CBYP1765.g1	GE276977
61920933	CBYP1766.b1	GE276978
61920934	CBYP1766.g1	GE276979
61920935	CBYP1767.b1	GE276980
61920936	CBYP1767.g1	GE276981
61920937	CBYP1769.b1	GE276982
61920938	CBYP1769.g1	GE276983
61920939	CBYP1770.g1	GE276984

61920940	CBYP1771.b1	GE276985
61920941	CBYP1771.g1	GE276986
61920942	CBYP1772.b1	GE276987
61920943	CBYP1772.g1	GE276988
61920944	CBYP1773.b1	GE276989
61920945	CBYP1773.g1	GE276990
61920946	CBYP1774.b1	GE276991
61920947	CBYP1774.g1	GE276992
61920948	CBYP1776.b1	GE276993
61920949	CBYP1776.g1	GE276994
61920950	CBYP1777.g1	GE276995
61920951	CBYP1778.b1	GE276996
61920952	CBYP1778.g1	GE276997
61920953	CBYP1779.b1	GE276998
61920954	CBYP1779.g1	GE276999
61920955	CBYP1780.b1	GE277000
61920956	CBYP1780.g1	GE277001
61920957	CBYP1781.g1	GE277002
61920958	CBYP1782.b1	GE277003
61920959	CBYP1782.g1	GE277004
61920960	CBYP1783.b1	GE277005
61920961	CBYP1783.g1	GE277006
61920962	CBYP1784.b1	GE277007
61920963	CBYP1784.g1	GE277008
61920964	CBYP1785.b1	GE277009
61920965	CBYP1785.g1	GE277010
61920966	CBYP1787.b1	GE277011
61920967	CBYP1787.g1	GE277012
61920968	CBYP1788.b1	GE277013
61920969	CBYP1788.g1	GE277014
61920970	CBYP1789.b1	GE277015
61920971	CBYP1789.g1	GE277016
61920972	CBYP1790.b1	GE277017
61920973	CBYP1790.g1	GE277018
61920974	CBYP1791.b1	GE277019
61920975	CBYP1791.g1	GE277020
61920976	CBYP1792.b1	GE277021
61920977	CBYP1793.g1	GE277022
61920978	CBYP1794.b1	GE277023
61920979	CBYP1794.g1	GE277024
61920980	CBYP1795.b1	GE277025
61920981	CBYP1796.b1	GE277026
61920982	CBYP1796.g1	GE277027
61920983	CBYP1797.b1	GE277028
61920984	CBYP1797.g1	GE277029
61920985	CBYP1798.b1	GE277030
61920986	CBYP1798.g1	GE277031
61920987	CBYP1799.b1	GE277032
61920988	CBYP1799.g1	GE277033
61920989	CBYP1800.b1	GE277034
61920990	CBYP1800.g1	GE277035
61920991	CBYP1802.b1	GE277036
61920992	CBYP1802.g1	GE277037
61920993	CBYP1803.b1	GE277038
61920994	CBYP1803.g1	GE277039
61920995	CBYP1804.b1	GE277040
61920996	CBYP1804.g1	GE277041
61920997	CBYP1805.b1	GE277042
61920998	CBYP1805.g1	GE277043
61920999	CBYP1806.b1	GE277044
61921000	CBYP1806.g1	GE277045
61921001	CBYP1807.b1	GE277046
61921002	CBYP1807.g1	GE277047

61921003	CBYP1809.b1	GE277048
61921004	CBYP1809.g1	GE277049
61921005	CBYP1810.b1	GE277050
61921006	CBYP1810.g1	GE277051
61921007	CBYP1811.b1	GE277052
61921008	CBYP1811.g1	GE277053
61921009	CBYP1812.g1	GE277054
61921010	CBYP1813.b1	GE277055
61921011	CBYP1813.g1	GE277056
61921012	CBYP1814.b1	GE277057
61921013	CBYP1814.g1	GE277058
61921014	CBYP1815.b1	GE277059
61921015	CBYP1815.g1	GE277060
61921016	CBYP1816.b1	GE277061
61921017	CBYP1816.g1	GE277062
61921018	CBYP1818.b1	GE277063
61921019	CBYP1818.g1	GE277064
61921020	CBYP1819.b1	GE277065
61921021	CBYP1819.g1	GE277066
61921022	CBYP1820.b1	GE277067
61921023	CBYP1820.g1	GE277068
61921024	CBYP1821.b1	GE277069
61921025	CBYP1821.g1	GE277070
61921026	CBYP1822.b1	GE277071
61921027	CBYP1822.g1	GE277072
61921028	CBYP1823.b1	GE277073
61921029	CBYP1823.g1	GE277074
61921030	CBYP1824.b1	GE277075
61921031	CBYP1825.b1	GE277076
61921032	CBYP1825.g1	GE277077
61921033	CBYP1826.b1	GE277078
61921034	CBYP1826.g1	GE277079
61921035	CBYP1827.b1	GE277080
61921036	CBYP1829.b1	GE277081
61921037	CBYP1829.g1	GE277082
61921038	CBYP1830.b1	GE277083
61921039	CBYP1830.g1	GE277084
61921040	CBYP1833.b1	GE277085
61921041	CBYP1833.g1	GE277086
61921042	CBYP1835.b1	GE277087
61921043	CBYP1836.b1	GE277088
61921044	CBYP1836.g1	GE277089
61921045	CBYP1837.b1	GE277090
61921046	CBYP1837.g1	GE277091
61921047	CBYP1838.b1	GE277092
61921048	CBYP1838.g1	GE277093
61921049	CBYP1840.b1	GE277094
61921050	CBYP1840.g1	GE277095
61921051	CBYP1841.b1	GE277096
61921052	CBYP1841.g1	GE277097
61921053	CBYP1842.b1	GE277098
61921054	CBYP1842.g1	GE277099
61921055	CBYP1843.b1	GE277100
61921056	CBYP1843.g1	GE277101
61921057	CBYP1844.b1	GE277102
61921058	CBYP1844.g1	GE277103
61921059	CBYP1846.b1	GE277104
61921060	CBYP1847.b1	GE277105
61921061	CBYP1847.g1	GE277106
61921062	CBYP1848.b1	GE277107
61921063	CBYP1848.g1	GE277108
61921064	CBYP1849.g1	GE277109
61921065	CBYP1850.b1	GE277110

61921066	CBYP1850.g1	GE277111
61921067	CBYP1851.b1	GE277112
61921068	CBYP1851.g1	GE277113
61921069	CBYP1852.b1	GE277114
61921070	CBYP1852.g1	GE277115
61921071	CBYP1853.b1	GE277116
61921072	CBYP1854.b1	GE277117
61921073	CBYP1854.g1	GE277118
61921074	CBYP1855.b1	GE277119
61921075	CBYP1856.b1	GE277120
61921076	CBYP1856.g1	GE277121
61921077	CBYP1857.b1	GE277122
61921078	CBYP1857.g1	GE277123
61921079	CBYP1858.b1	GE277124
61921080	CBYP1858.g1	GE277125
61921081	CBYP1859.b1	GE277126
61921082	CBYP1859.g1	GE277127
61921083	CBYP1860.b1	GE277128
61921084	CBYP1860.g1	GE277129
61921085	CBYP1861.b1	GE277130
61921086	CBYP1861.g1	GE277131
61921087	CBYP1862.b1	GE277132
61921088	CBYP1862.g1	GE277133
61921089	CBYP1863.b1	GE277134
61921090	CBYP1863.g1	GE277135
61921091	CBYP1864.b1	GE277136
61921092	CBYP1864.g1	GE277137
61921093	CBYP1865.b1	GE277138
61921094	CBYP1865.g1	GE277139
61921095	CBYP1866.b1	GE277140
61921096	CBYP1866.g1	GE277141
61921097	CBYP1867.b1	GE277142
61921098	CBYP1867.g1	GE277143
61921099	CBYP1868.b1	GE277144
61921100	CBYP1868.g1	GE277145
61921101	CBYP1869.b1	GE277146
61921102	CBYP1869.g1	GE277147
61921103	CBYP1870.b1	GE277148
61921104	CBYP1870.g1	GE277149
61921105	CBYP1871.b1	GE277150
61921106	CBYP1871.g1	GE277151
61921107	CBYP1872.b1	GE277152
61921108	CBYP1872.g1	GE277153
61921109	CBYP1874.b1	GE277154
61921110	CBYP1874.g1	GE277155
61921111	CBYP1875.b1	GE277156
61921112	CBYP1875.g1	GE277157
61921113	CBYP1876.b1	GE277158
61921114	CBYP1876.g1	GE277159
61921115	CBYP1877.b1	GE277160
61921116	CBYP1877.g1	GE277161
61921117	CBYP1878.b1	GE277162
61921118	CBYP1878.g1	GE277163
61921119	CBYP1879.b1	GE277164
61921120	CBYP1879.g1	GE277165
61921121	CBYP1880.b1	GE277166
61921122	CBYP1880.g1	GE277167
61921123	CBYP1881.b1	GE277168
61921124	CBYP1881.g1	GE277169
61921125	CBYP1882.b1	GE277170
61921126	CBYP1882.g1	GE277171
61921127	CBYP1883.b1	GE277172
61921128	CBYP1883.g1	GE277173

61921129	CBYP1884.b1	GE277174
61921130	CBYP1884.g1	GE277175
61921131	CBYP1885.b1	GE277176
61921132	CBYP1885.g1	GE277177
61921133	CBYP1887.b1	GE277178
61921134	CBYP1887.g1	GE277179
61921135	CBYP1888.b1	GE277180
61921136	CBYP1888.g1	GE277181
61921137	CBYP1889.b1	GE277182
61921138	CBYP1889.g1	GE277183
61921139	CBYP1890.b1	GE277184
61921140	CBYP1890.g1	GE277185
61921141	CBYP1891.g1	GE277186
61921142	CBYP1892.b1	GE277187
61921143	CBYP1892.g1	GE277188
61921144	CBYP1893.b1	GE277189
61921145	CBYP1893.g1	GE277190
61921146	CBYP1894.b1	GE277191
61921147	CBYP1894.g1	GE277192
61921148	CBYP1895.b1	GE277193
61921149	CBYP1895.g1	GE277194
61921150	CBYP1896.b1	GE277195
61921151	CBYP1896.g1	GE277196
61921152	CBYP1897.b1	GE277197
61921153	CBYP1897.g1	GE277198
61921154	CBYP1898.b1	GE277199
61921155	CBYP1898.g1	GE277200
61921156	CBYP1899.b1	GE277201
61921157	CBYP1899.g1	GE277202
61921158	CBYP1900.b1	GE277203
61921159	CBYP1900.g1	GE277204
61921160	CBYP1901.b1	GE277205
61921161	CBYP1901.g1	GE277206
61921162	CBYP1902.b1	GE277207
61921163	CBYP1902.g1	GE277208
61921164	CBYP1903.b1	GE277209
61921165	CBYP1904.b1	GE277210
61921166	CBYP1904.g1	GE277211
61921167	CBYP1905.b1	GE277212
61921168	CBYP1905.g1	GE277213
61921169	CBYP1906.b1	GE277214
61921170	CBYP1906.g1	GE277215
61921171	CBYP1909.b1	GE277216
61921172	CBYP1909.g1	GE277217
61921173	CBYP1910.b1	GE277218
61921174	CBYP1910.g1	GE277219
61921175	CBYP1911.b1	GE277220
61921176	CBYP1911.g1	GE277221
61921177	CBYP1912.b1	GE277222
61921178	CBYP1912.g1	GE277223
61921179	CBYP1913.b1	GE277224
61921180	CBYP1913.g1	GE277225
61921181	CBYP1914.b1	GE277226
61921182	CBYP1914.g1	GE277227
61921183	CBYP1915.g1	GE277228
61921184	CBYP1916.b1	GE277229
61921185	CBYP1916.g1	GE277230
61921186	CBYP1917.g1	GE277231
61921187	CBYP1918.b1	GE277232
61921188	CBYP1918.g1	GE277233
61921189	CBYP1919.b1	GE277234
61921190	CBYP1921.b1	GE277235
61921191	CBYP1921.g1	GE277236

61921192	CBYP1923.b1	GE277237
61921193	CBYP1923.g1	GE277238
61921194	CBYP1924.b1	GE277239
61921195	CBYP1924.g1	GE277240
61921196	CBYP1925.b1	GE277241
61921197	CBYP1925.g1	GE277242
61921198	CBYP1926.b1	GE277243
61921199	CBYP1926.g1	GE277244
61921200	CBYP1927.b1	GE277245
61921201	CBYP1927.g1	GE277246
61921202	CBYP1928.b1	GE277247
61921203	CBYP1928.g1	GE277248
61921204	CBYP1929.b1	GE277249
61921205	CBYP1929.g1	GE277250
61921206	CBYP1931.b1	GE277251
61921207	CBYP1931.g1	GE277252
61921208	CBYP1932.b1	GE277253
61921209	CBYP1932.g1	GE277254
61921210	CBYP1933.b1	GE277255
61921211	CBYP1933.g1	GE277256
61921212	CBYP1934.b1	GE277257
61921213	CBYP1934.g1	GE277258
61921214	CBYP1935.b1	GE277259
61921215	CBYP1935.g1	GE277260
61921216	CBYP1936.b1	GE277261
61921217	CBYP1936.g1	GE277262
61921218	CBYP1937.b1	GE277263
61921219	CBYP1938.b1	GE277264
61921220	CBYP1938.g1	GE277265
61921221	CBYP1939.b1	GE277266
61921222	CBYP1939.g1	GE277267
61921223	CBYP1940.b1	GE277268
61921224	CBYP1941.b1	GE277269
61921225	CBYP1941.g1	GE277270
61921226	CBYP1942.b1	GE277271
61921227	CBYP1942.g1	GE277272
61921228	CBYP1943.b1	GE277273
61921229	CBYP1943.g1	GE277274
61921230	CBYP1944.b1	GE277275
61921231	CBYP1944.g1	GE277276
61921232	CBYP1945.b1	GE277277
61921233	CBYP1945.g1	GE277278
61921234	CBYP1946.b1	GE277279
61921235	CBYP1947.b1	GE277280
61921236	CBYP1947.g1	GE277281
61921237	CBYP1948.b1	GE277282
61921238	CBYP1948.g1	GE277283
61921239	CBYP1949.b1	GE277284
61921240	CBYP1949.g1	GE277285
61921241	CBYP1950.b1	GE277286
61921242	CBYP1950.g1	GE277287
61921243	CBYP1951.b1	GE277288
61921244	CBYP1951.g1	GE277289
61921245	CBYP1952.b1	GE277290
61921246	CBYP1952.g1	GE277291
61921247	CBYP1953.b1	GE277292
61921248	CBYP1953.g1	GE277293
61921249	CBYP1954.b1	GE277294
61921250	CBYP1955.b1	GE277295
61921251	CBYP1955.g1	GE277296
61921252	CBYP1956.b1	GE277297
61921253	CBYP1956.g1	GE277298
61921254	CBYP1957.b1	GE277299

61921255	CBYP1957.g1	GE277300
61921256	CBYP1958.b1	GE277301
61921257	CBYP1958.g1	GE277302
61921258	CBYP1959.b1	GE277303
61921259	CBYP1959.g1	GE277304
61921260	CBYP1960.b1	GE277305
61921261	CBYP1960.g1	GE277306
61921262	CBYP1962.b1	GE277307
61921263	CBYP1962.g1	GE277308
61921264	CBYP1963.b1	GE277309
61921265	CBYP1963.g1	GE277310
61921266	CBYP1964.b1	GE277311
61921267	CBYP1964.g1	GE277312
61921268	CBYP1965.b1	GE277313
61921269	CBYP1965.g1	GE277314
61921270	CBYP1966.b1	GE277315
61921271	CBYP1966.g1	GE277316
61921272	CBYP1967.b1	GE277317
61921273	CBYP1967.g1	GE277318
61921274	CBYP1969.b1	GE277319
61921275	CBYP1969.g1	GE277320
61921276	CBYP1970.b1	GE277321
61921277	CBYP1971.g1	GE277322
61921278	CBYP1972.b1	GE277323
61921279	CBYP1972.g1	GE277324
61921280	CBYP1973.b1	GE277325
61921281	CBYP1973.g1	GE277326
61921282	CBYP1974.b1	GE277327
61921283	CBYP1974.g1	GE277328
61921284	CBYP1975.g1	GE277329
61921285	CBYP1976.g1	GE277330
61921286	CBYP1977.b1	GE277331
61921287	CBYP1977.g1	GE277332
61921288	CBYP1978.b1	GE277333
61921289	CBYP1978.g1	GE277334
61921290	CBYP1979.b1	GE277335
61921291	CBYP1979.g1	GE277336
61921292	CBYP1980.b1	GE277337
61921293	CBYP1980.g1	GE277338
61921294	CBYP1983.b1	GE277339
61921295	CBYP1983.g1	GE277340
61921296	CBYP1984.b1	GE277341
61921297	CBYP1985.b1	GE277342
61921298	CBYP1985.g1	GE277343
61921299	CBYP1986.b1	GE277344
61921300	CBYP1986.g1	GE277345
61921301	CBYP1987.b1	GE277346
61921302	CBYP1987.g1	GE277347
61921303	CBYP1988.b1	GE277348
61921304	CBYP1989.b1	GE277349
61921305	CBYP1989.g1	GE277350
61921306	CBYP1990.b1	GE277351
61921307	CBYP1990.g1	GE277352
61921308	CBYP1991.b1	GE277353
61921309	CBYP1991.g1	GE277354
61921310	CBYP1992.b1	GE277355
61921311	CBYP1992.g1	GE277356
61921312	CBYP1993.b1	GE277357
61921313	CBYP1993.g1	GE277358
61921314	CBYP1994.b1	GE277359
61921315	CBYP1994.g1	GE277360
61921316	CBYP1995.b1	GE277361
61921317	CBYP1995.g1	GE277362

61921318	CBYP1996.b1	GE277363
61921319	CBYP1996.g1	GE277364
61921320	CBYP1997.b1	GE277365
61921321	CBYP1997.g1	GE277366
61921322	CBYP1998.b1	GE277367
61921323	CBYP1998.g1	GE277368
61921324	CBYP1999.b1	GE277369
61921325	CBYP1999.g1	GE277370
61921326	CBYP2000.b1	GE277371
61921327	CBYP2000.g1	GE277372
61921328	CBYP2001.b1	GE277373
61921329	CBYP2001.g1	GE277374
61921330	CBYP2002.b1	GE277375
61921331	CBYP2002.g1	GE277376
61921332	CBYP2004.b1	GE277377
61921333	CBYP2004.g1	GE277378
61921334	CBYP2005.b1	GE277379
61921335	CBYP2005.g1	GE277380
61921336	CBYP2006.b1	GE277381
61921337	CBYP2006.g1	GE277382
61921338	CBYP2007.b1	GE277383
61921339	CBYP2007.g1	GE277384
61921340	CBYP2008.b1	GE277385
61921341	CBYP2008.g1	GE277386
61921342	CBYP2009.b1	GE277387
61921343	CBYP2009.g1	GE277388
61921344	CBYP2010.b1	GE277389
61921345	CBYP2010.g1	GE277390
61921346	CBYP2011.b1	GE277391
61921347	CBYP2011.g1	GE277392
61921348	CBYP2012.b1	GE277393
61921349	CBYP2012.g1	GE277394
61921350	CBYP2013.b1	GE277395
61921351	CBYP2013.g1	GE277396
61921352	CBYP2014.b1	GE277397
61921353	CBYP2014.g1	GE277398
61921354	CBYP2015.b1	GE277399
61921355	CBYP2015.g1	GE277400
61921356	CBYP2016.b1	GE277401
61921357	CBYP2016.g1	GE277402
61921358	CBYP2017.b1	GE277403
61921359	CBYP2017.g1	GE277404
61921360	CBYP2018.b1	GE277405
61921361	CBYP2018.g1	GE277406
61921362	CBYP2019.b1	GE277407
61921363	CBYP2019.g1	GE277408
61921364	CBYP2020.b1	GE277409
61921365	CBYP2020.g1	GE277410
61921366	CBYP2021.b1	GE277411
61921367	CBYP2021.g1	GE277412
61921368	CBYP2022.b1	GE277413
61921369	CBYP2022.g1	GE277414
61921370	CBYP2023.b1	GE277415
61921371	CBYP2023.g1	GE277416
61921372	CBYP2024.b1	GE277417
61921373	CBYP2025.b1	GE277418
61921374	CBYP2025.g1	GE277419
61921375	CBYP2026.b1	GE277420
61921376	CBYP2026.g1	GE277421
61921377	CBYP2027.b1	GE277422
61921378	CBYP2027.g1	GE277423
61921379	CBYP2028.b1	GE277424
61921380	CBYP2028.g1	GE277425

61921381	CBYP2030.b1	GE277426
61921382	CBYP2030.g1	GE277427
61921383	CBYP2031.b1	GE277428
61921384	CBYP2032.b1	GE277429
61921385	CBYP2032.g1	GE277430
61921386	CBYP2033.b1	GE277431
61921387	CBYP2033.g1	GE277432
61921388	CBYP2034.b1	GE277433
61921389	CBYP2034.g1	GE277434
61921390	CBYP2036.b1	GE277435
61921391	CBYP2036.g1	GE277436
61921392	CBYP2037.b1	GE277437
61921393	CBYP2037.g1	GE277438
61921394	CBYP2038.b1	GE277439
61921395	CBYP2038.g1	GE277440
61921396	CBYP2039.b1	GE277441
61921397	CBYP2039.g1	GE277442
61921398	CBYP2040.b1	GE277443
61921399	CBYP2040.g1	GE277444
61921400	CBYP2041.b1	GE277445
61921401	CBYP2041.g1	GE277446
61921402	CBYP2042.b1	GE277447
61921403	CBYP2042.g1	GE277448
61921404	CBYP2043.b1	GE277449
61921405	CBYP2044.b1	GE277450
61921406	CBYP2044.g1	GE277451
61921407	CBYP2045.b1	GE277452
61921408	CBYP2045.g1	GE277453
61921409	CBYP2046.b1	GE277454
61921410	CBYP2046.g1	GE277455
61921411	CBYP2048.b1	GE277456
61921412	CBYP2048.g1	GE277457
61921413	CBYP2049.b1	GE277458
61921414	CBYP2049.g1	GE277459
61921415	CBYP2050.b1	GE277460
61921416	CBYP2050.g1	GE277461
61921417	CBYP2051.b1	GE277462
61921418	CBYP2051.g1	GE277463
61921419	CBYP2052.b1	GE277464
61921420	CBYP2052.g1	GE277465
61921421	CBYP2053.b1	GE277466
61921422	CBYP2053.g1	GE277467
61921423	CBYP2054.b1	GE277468
61921424	CBYP2054.g1	GE277469
61921425	CBYP2055.b1	GE277470
61921426	CBYP2055.g1	GE277471
61921427	CBYP2056.b1	GE277472
61921428	CBYP2056.g1	GE277473
61921429	CBYP2057.b1	GE277474
61921430	CBYP2057.g1	GE277475
61921431	CBYP2058.b1	GE277476
61921432	CBYP2058.g1	GE277477
61921433	CBYP2059.b1	GE277478
61921434	CBYP2059.g1	GE277479
61921435	CBYP2060.b1	GE277480
61921436	CBYP2060.g1	GE277481
61921437	CBYP2061.b1	GE277482
61921438	CBYP2061.g1	GE277483
61921439	CBYP2062.b1	GE277484
61921440	CBYP2062.g1	GE277485
61921441	CBYP2063.b1	GE277486
61921442	CBYP2063.g1	GE277487
61921443	CBYP2064.b1	GE277488

61921444	CBYP2064.g1	GE277489
61921445	CBYP2065.b1	GE277490
61921446	CBYP2065.g1	GE277491
61921447	CBYP2066.g1	GE277492
61921448	CBYP2067.g1	GE277493
61921449	CBYP2068.b1	GE277494
61921450	CBYP2068.g1	GE277495
61921451	CBYP2069.b1	GE277496
61921452	CBYP2070.b1	GE277497
61921453	CBYP2070.g1	GE277498
61921454	CBYP2071.b1	GE277499
61921455	CBYP2071.g1	GE277500
61921456	CBYP2072.b1	GE277501
61921457	CBYP2072.g1	GE277502
61921458	CBYP2073.b1	GE277503
61921459	CBYP2073.g1	GE277504
61921460	CBYP2074.b1	GE277505
61921461	CBYP2074.g1	GE277506
61921462	CBYP2075.g1	GE277507
61921463	CBYP2076.b1	GE277508
61921464	CBYP2076.g1	GE277509
61921465	CBYP2077.b1	GE277510
61921466	CBYP2077.g1	GE277511
61921467	CBYP2078.g1	GE277512
61921468	CBYP2079.b1	GE277513
61921469	CBYP2079.g1	GE277514
61921470	CBYP2080.b1	GE277515
61921471	CBYP2080.g1	GE277516
61921472	CBYP2081.b1	GE277517
61921473	CBYP2081.g1	GE277518
61921474	CBYP2083.g1	GE277519
61921475	CBYP2084.b1	GE277520
61921476	CBYP2084.g1	GE277521
61921477	CBYP2085.b1	GE277522
61921478	CBYP2085.g1	GE277523
61921479	CBYP2086.b1	GE277524
61921480	CBYP2086.g1	GE277525
61921481	CBYP2087.b1	GE277526
61921482	CBYP2087.g1	GE277527
61921483	CBYP2088.b1	GE277528
61921484	CBYP2088.g1	GE277529
61921485	CBYP2089.b1	GE277530
61921486	CBYP2089.g1	GE277531
61921487	CBYP2090.b1	GE277532
61921488	CBYP2090.g1	GE277533
61921489	CBYP2091.b1	GE277534
61921490	CBYP2091.g1	GE277535
61921491	CBYP2092.g1	GE277536
61921492	CBYP2093.g1	GE277537
61921493	CBYP2095.b1	GE277538
61921494	CBYP2095.g1	GE277539
61921495	CBYP2096.b1	GE277540
61921496	CBYP2096.g1	GE277541
61921497	CBYP2097.b1	GE277542
61921498	CBYP2097.g1	GE277543
61921499	CBYP2098.b1	GE277544
61921500	CBYP2098.g1	GE277545
61921501	CBYP2099.b1	GE277546
61921502	CBYP2099.g1	GE277547
61921503	CBYP2100.b1	GE277548
61921504	CBYP2100.g1	GE277549
61921505	CBYP2101.b1	GE277550
61921506	CBYP2101.g1	GE277551

61921507	CBYP2102.b1	GE277552
61921508	CBYP2103.b1	GE277553
61921509	CBYP2103.g1	GE277554
61921510	CBYP2104.b1	GE277555
61921511	CBYP2104.g1	GE277556
61921512	CBYP2105.b1	GE277557
61921513	CBYP2105.g1	GE277558
61921514	CBYP2106.b1	GE277559
61921515	CBYP2106.g1	GE277560
61921516	CBYP2108.b1	GE277561
61921517	CBYP2108.g1	GE277562
61921518	CBYP2109.b1	GE277563
61921519	CBYP2109.g1	GE277564
61921520	CBYP2110.b1	GE277565
61921521	CBYP2110.g1	GE277566
61921522	CBYP2111.b1	GE277567
61921523	CBYP2111.g1	GE277568
61921524	CBYP2112.b1	GE277569
61921525	CBYP2112.g1	GE277570
61921526	CBYP2113.b1	GE277571
61921527	CBYP2113.g1	GE277572
61921528	CBYP2114.b1	GE277573
61921529	CBYP2114.g1	GE277574
61921530	CBYP2115.b1	GE277575
61921531	CBYP2115.g1	GE277576
61921532	CBYP2116.b1	GE277577
61921533	CBYP2116.g1	GE277578
61921534	CBYP2117.b1	GE277579
61921535	CBYP2117.g1	GE277580
61921536	CBYP2118.b1	GE277581
61921537	CBYP2118.g1	GE277582
61921538	CBYP2119.b1	GE277583
61921539	CBYP2119.g1	GE277584
61921540	CBYP2120.b1	GE277585
61921541	CBYP2120.g1	GE277586
61921542	CBYP2121.b1	GE277587
61921543	CBYP2121.g1	GE277588
61921544	CBYP2122.b1	GE277589
61921545	CBYP2122.g1	GE277590
61921546	CBYP2123.b1	GE277591
61921547	CBYP2123.g1	GE277592
61921548	CBYP2124.b1	GE277593
61921549	CBYP2124.g1	GE277594
61921550	CBYP2125.b1	GE277595
61921551	CBYP2125.g1	GE277596
61921552	CBYP2126.b1	GE277597
61921553	CBYP2126.g1	GE277598
61921554	CBYP2127.b1	GE277599
61921555	CBYP2127.g1	GE277600
61921556	CBYP2128.b1	GE277601
61921557	CBYP2128.g1	GE277602
61921558	CBYP2129.b1	GE277603
61921559	CBYP2129.g1	GE277604
61921560	CBYP2130.b1	GE277605
61921561	CBYP2130.g1	GE277606
61921562	CBYP2131.b1	GE277607
61921563	CBYP2131.g1	GE277608
61921564	CBYP2132.b1	GE277609
61921565	CBYP2132.g1	GE277610
61921566	CBYP2133.b1	GE277611
61921567	CBYP2133.g1	GE277612
61921568	CBYP2135.b1	GE277613
61921569	CBYP2135.g1	GE277614

61921570	CBYP2136.b1	GE277615
61921571	CBYP2136.g1	GE277616
61921572	CBYP2137.b1	GE277617
61921573	CBYP2137.g1	GE277618
61921574	CBYP2138.g1	GE277619
61921575	CBYP2139.b1	GE277620
61921576	CBYP2139.g1	GE277621
61921577	CBYP2140.b1	GE277622
61921578	CBYP2140.g1	GE277623
61921579	CBYP2142.b1	GE277624
61921580	CBYP2142.g1	GE277625
61921581	CBYP2143.b1	GE277626
61921582	CBYP2143.g1	GE277627
61921583	CBYP2144.b1	GE277628
61921584	CBYP2144.g1	GE277629
61921585	CBYP2145.b1	GE277630
61921586	CBYP2145.g1	GE277631
61921587	CBYP2146.b1	GE277632
61921588	CBYP2146.g1	GE277633
61921589	CBYP2147.b1	GE277634
61921590	CBYP2148.b1	GE277635
61921591	CBYP2148.g1	GE277636
61921592	CBYP2149.g1	GE277637
61921593	CBYP2150.b1	GE277638
61921594	CBYP2150.g1	GE277639
61921595	CBYP2151.b1	GE277640
61921596	CBYP2151.g1	GE277641
61921597	CBYP2152.b1	GE277642
61921598	CBYP2152.g1	GE277643
61921599	CBYP2153.b1	GE277644
61921600	CBYP2153.g1	GE277645
61921601	CBYP2154.b1	GE277646
61921602	CBYP2154.g1	GE277647
61921603	CBYP2155.b1	GE277648
61921604	CBYP2155.g1	GE277649
61921605	CBYP2156.b1	GE277650
61921606	CBYP2156.g1	GE277651
61921607	CBYP2157.b1	GE277652
61921608	CBYP2157.g1	GE277653
61921609	CBYP2158.b1	GE277654
61921610	CBYP2158.g1	GE277655
61921611	CBYP2159.b1	GE277656
61921612	CBYP2159.g1	GE277657
61921613	CBYP2160.g1	GE277658
61921614	CBYP2161.b1	GE277659
61921615	CBYP2163.b1	GE277660
61921616	CBYP2163.g1	GE277661
61921617	CBYP2164.b1	GE277662
61921618	CBYP2164.g1	GE277663
61921619	CBYP2165.b1	GE277664
61921620	CBYP2165.g1	GE277665
61921621	CBYP2166.b1	GE277666
61921622	CBYP2166.g1	GE277667
61921623	CBYP2167.b1	GE277668
61921624	CBYP2167.g1	GE277669
61921625	CBYP2168.b1	GE277670
61921626	CBYP2168.g1	GE277671
61921627	CBYP2169.b1	GE277672
61921628	CBYP2169.g1	GE277673
61921629	CBYP2170.b1	GE277674
61921630	CBYP2170.g1	GE277675
61921631	CBYP2171.b1	GE277676
61921632	CBYP2171.g1	GE277677

61921633	CBYP2172.b1	GE277678
61921634	CBYP2172.g1	GE277679
61921635	CBYP2173.b1	GE277680
61921636	CBYP2173.g1	GE277681
61921637	CBYP2174.b1	GE277682
61921638	CBYP2174.g1	GE277683
61921639	CBYP2176.b1	GE277684
61921640	CBYP2176.g1	GE277685
61921641	CBYP2177.b1	GE277686
61921642	CBYP2177.g1	GE277687
61921643	CBYP2178.b1	GE277688
61921644	CBYP2178.g1	GE277689
61921645	CBYP2179.b1	GE277690
61921646	CBYP2179.g1	GE277691
61921647	CBYP2181.b1	GE277692
61921648	CBYP2181.g1	GE277693
61921649	CBYP2182.b1	GE277694
61921650	CBYP2182.g1	GE277695
61921651	CBYP2183.b1	GE277696
61921652	CBYP2183.g1	GE277697
61921653	CBYP2184.b1	GE277698
61921654	CBYP2184.g1	GE277699
61921655	CBYP2185.b1	GE277700
61921656	CBYP2185.g1	GE277701
61921657	CBYP2186.b1	GE277702
61921658	CBYP2186.g1	GE277703
61921659	CBYP2187.b1	GE277704
61921660	CBYP2187.g1	GE277705
61921661	CBYP2188.b1	GE277706
61921662	CBYP2188.g1	GE277707
61921663	CBYP2189.b1	GE277708
61921664	CBYP2189.g1	GE277709
61921665	CBYP2190.b1	GE277710
61921666	CBYP2190.g1	GE277711
61921667	CBYP2191.g1	GE277712
61921668	CBYP2192.b1	GE277713
61921669	CBYP2192.g1	GE277714
61921670	CBYP2193.b1	GE277715
61921671	CBYP2193.g1	GE277716
61921672	CBYP2194.b1	GE277717
61921673	CBYP2194.g1	GE277718
61921674	CBYP2195.b1	GE277719
61921675	CBYP2195.g1	GE277720
61921676	CBYP2197.b1	GE277721
61921677	CBYP2197.g1	GE277722
61921678	CBYP2198.b1	GE277723
61921679	CBYP2198.g1	GE277724
61921680	CBYP2199.b1	GE277725
61921681	CBYP2199.g1	GE277726
61921682	CBYP2200.b1	GE277727
61921683	CBYP2200.g1	GE277728
61921684	CBYP2201.b1	GE277729
61921685	CBYP2201.g1	GE277730
61921686	CBYP2202.b1	GE277731
61921687	CBYP2202.g1	GE277732
61921688	CBYP2203.b1	GE277733
61921689	CBYP2203.g1	GE277734
61921690	CBYP2204.b1	GE277735
61921691	CBYP2204.g1	GE277736
61921692	CBYP2205.b1	GE277737
61921693	CBYP2205.g1	GE277738
61921694	CBYP2206.b1	GE277739
61921695	CBYP2206.g1	GE277740

61921696	CBYP2207.b1	GE277741
61921697	CBYP2207.g1	GE277742
61921698	CBYP2208.b1	GE277743
61921699	CBYP2208.g1	GE277744
61921700	CBYP2209.b1	GE277745
61921701	CBYP2209.g1	GE277746
61921702	CBYP2210.b1	GE277747
61921703	CBYP2210.g1	GE277748
61921704	CBYP2211.b1	GE277749
61921705	CBYP2211.g1	GE277750
61921706	CBYP2212.b1	GE277751
61921707	CBYP2212.g1	GE277752
61921708	CBYP2213.b1	GE277753
61921709	CBYP2213.g1	GE277754
61921710	CBYP2214.b1	GE277755
61921711	CBYP2215.b1	GE277756
61921712	CBYP2217.b1	GE277757
61921713	CBYP2217.g1	GE277758
61921714	CBYP2218.b1	GE277759
61921715	CBYP2218.g1	GE277760
61921716	CBYP2219.b1	GE277761
61921717	CBYP2219.g1	GE277762
61921718	CBYP2220.b1	GE277763
61921719	CBYP2220.g1	GE277764
61921720	CBYP2221.b1	GE277765
61921721	CBYP2221.g1	GE277766
61921722	CBYP2222.b1	GE277767
61921723	CBYP2222.g1	GE277768
61921724	CBYP2223.b1	GE277769
61921725	CBYP2223.g1	GE277770
61921726	CBYP2224.b1	GE277771
61921727	CBYP2224.g1	GE277772
61921728	CBYP2225.b1	GE277773
61921729	CBYP2225.g1	GE277774
61921730	CBYP2226.b1	GE277775
61921731	CBYP2226.g1	GE277776
61921732	CBYP2228.b1	GE277777
61921733	CBYP2228.g1	GE277778
61921734	CBYP2229.b1	GE277779
61921735	CBYP2229.g1	GE277780
61921736	CBYP2230.b1	GE277781
61921737	CBYP2230.g1	GE277782
61921738	CBYP2231.b1	GE277783
61921739	CBYP2231.g1	GE277784
61921740	CBYP2232.b1	GE277785
61921741	CBYP2233.b1	GE277786
61921742	CBYP2233.g1	GE277787
61921743	CBYP2234.b1	GE277788
61921744	CBYP2234.g1	GE277789
61921745	CBYP2235.b1	GE277790
61921746	CBYP2235.g1	GE277791
61921747	CBYP2236.b1	GE277792
61921748	CBYP2236.g1	GE277793
61921749	CBYP2237.b1	GE277794
61921750	CBYP2237.g1	GE277795
61921751	CBYP2238.b1	GE277796
61921752	CBYP2239.b1	GE277797
61921753	CBYP2239.g1	GE277798
61921754	CBYP2241.b1	GE277799
61921755	CBYP2241.g1	GE277800
61921756	CBYP2242.b1	GE277801
61921757	CBYP2242.g1	GE277802
61921758	CBYP2243.b1	GE277803

61921759	CBYP2243.g1	GE277804
61921760	CBYP2245.b1	GE277805
61921761	CBYP2245.g1	GE277806
61921762	CBYP2246.b1	GE277807
61921763	CBYP2246.g1	GE277808
61921764	CBYP2247.b1	GE277809
61921765	CBYP2247.g1	GE277810
61921766	CBYP2249.b1	GE277811
61921767	CBYP2249.g1	GE277812
61921768	CBYP2250.b1	GE277813
61921769	CBYP2250.g1	GE277814
61921770	CBYP2251.b1	GE277815
61921771	CBYP2251.g1	GE277816
61921772	CBYP2252.b1	GE277817
61921773	CBYP2252.g1	GE277818
61921774	CBYP2253.b1	GE277819
61921775	CBYP2253.g1	GE277820
61921776	CBYP2255.b1	GE277821
61921777	CBYP2255.g1	GE277822
61921778	CBYP2256.b1	GE277823
61921779	CBYP2257.b1	GE277824
61921780	CBYP2257.g1	GE277825
61921781	CBYP2259.b1	GE277826
61921782	CBYP2259.g1	GE277827
61921783	CBYP2260.b1	GE277828
61921784	CBYP2260.g1	GE277829
61921785	CBYP2261.b1	GE277830
61921786	CBYP2261.g1	GE277831
61921787	CBYP2262.b1	GE277832
61921788	CBYP2262.g1	GE277833
61921789	CBYP2263.b1	GE277834
61921790	CBYP2263.g1	GE277835
61921791	CBYP2264.b1	GE277836
61921792	CBYP2264.g1	GE277837
61921793	CBYP2265.b1	GE277838
61921794	CBYP2265.g1	GE277839
61921795	CBYP2266.b1	GE277840
61921796	CBYP2266.g1	GE277841
61921797	CBYP2267.b1	GE277842
61921798	CBYP2267.g1	GE277843
61921799	CBYP2268.b1	GE277844
61921800	CBYP2268.g1	GE277845
61921801	CBYP2269.b1	GE277846
61921802	CBYP2269.g1	GE277847
61921803	CBYP2270.b1	GE277848
61921804	CBYP2270.g1	GE277849
61921805	CBYP2271.b1	GE277850
61921806	CBYP2271.g1	GE277851
61921807	CBYP2272.b1	GE277852
61921808	CBYP2272.g1	GE277853
61921809	CBYP2273.b1	GE277854
61921810	CBYP2273.g1	GE277855
61921811	CBYP2274.b1	GE277856
61921812	CBYP2275.b1	GE277857
61921813	CBYP2275.g1	GE277858
61921814	CBYP2276.g1	GE277859
61921815	CBYP2277.b1	GE277860
61921816	CBYP2277.g1	GE277861
61921817	CBYP2278.b1	GE277862
61921818	CBYP2278.g1	GE277863
61921819	CBYP2279.b1	GE277864
61921820	CBYP2279.g1	GE277865
61921821	CBYP2280.b1	GE277866

61921822	CBYP2280.g1	GE277867
61921823	CBYP2281.b1	GE277868
61921824	CBYP2281.g1	GE277869
61921825	CBYP2282.b1	GE277870
61921826	CBYP2282.g1	GE277871
61921827	CBYP2283.b1	GE277872
61921828	CBYP2283.g1	GE277873
61921829	CBYP2284.b1	GE277874
61921830	CBYP2284.g1	GE277875
61921831	CBYP2285.b1	GE277876
61921832	CBYP2285.g1	GE277877
61921833	CBYP2286.b1	GE277878
61921834	CBYP2286.g1	GE277879
61921835	CBYP2287.b1	GE277880
61921836	CBYP2287.g1	GE277881
61921837	CBYP2288.b1	GE277882
61921838	CBYP2288.g1	GE277883
61921839	CBYP2289.b1	GE277884
61921840	CBYP2289.g1	GE277885
61921841	CBYP2290.b1	GE277886
61921842	CBYP2290.g1	GE277887
61921843	CBYP2292.b1	GE277888
61921844	CBYP2292.g1	GE277889
61921845	CBYP2293.b1	GE277890
61921846	CBYP2293.g1	GE277891
61921847	CBYP2294.b1	GE277892
61921848	CBYP2295.b1	GE277893
61921849	CBYP2295.g1	GE277894
61921850	CBYP2296.b1	GE277895
61921851	CBYP2296.g1	GE277896
61921852	CBYP2297.b1	GE277897
61921853	CBYP2297.g1	GE277898
61921854	CBYP2298.b1	GE277899
61921855	CBYP2298.g1	GE277900
61921856	CBYP2299.b1	GE277901
61921857	CBYP2299.g1	GE277902
61921858	CBYP2300.b1	GE277903
61921859	CBYP2300.g1	GE277904
61921860	CBYP2301.b1	GE277905
61921861	CBYP2301.g1	GE277906
61921862	CBYP2302.b1	GE277907
61921863	CBYP2302.g1	GE277908
61921864	CBYP2303.b1	GE277909
61921865	CBYP2303.g1	GE277910
61921866	CBYP2305.b1	GE277911
61921867	CBYP2305.g1	GE277912
61921868	CBYP2306.b1	GE277913
61921869	CBYP2306.g1	GE277914
61921870	CBYP2307.b1	GE277915
61921871	CBYP2307.g1	GE277916
61921872	CBYP2308.b1	GE277917
61921873	CBYP2308.g1	GE277918
61921874	CBYP2309.b1	GE277919
61921875	CBYP2309.g1	GE277920
61921876	CBYP2310.b1	GE277921
61921877	CBYP2310.g1	GE277922
61921878	CBYP2311.b1	GE277923
61921879	CBYP2311.g1	GE277924
61921880	CBYP2312.b1	GE277925
61921881	CBYP2312.g1	GE277926
61921882	CBYP2313.b1	GE277927
61921883	CBYP2313.g1	GE277928
61921884	CBYP2314.b1	GE277929

61921885	CBYP2314.g1	GE277930
61921886	CBYP2315.b1	GE277931
61921887	CBYP2315.g1	GE277932
61921888	CBYP2316.b1	GE277933
61921889	CBYP2316.g1	GE277934
61921890	CBYP2317.b1	GE277935
61921891	CBYP2317.g1	GE277936
61921892	CBYP2318.b1	GE277937
61921893	CBYP2318.g1	GE277938
61921894	CBYP2319.b1	GE277939
61921895	CBYP2319.g1	GE277940
61921896	CBYP2321.b1	GE277941
61921897	CBYP2321.g1	GE277942
61921898	CBYP2322.b1	GE277943
61921899	CBYP2322.g1	GE277944
61921900	CBYP2323.b1	GE277945
61921901	CBYP2323.g1	GE277946
61921902	CBYP2324.b1	GE277947
61921903	CBYP2324.g1	GE277948
61921904	CBYP2327.b1	GE277949
61921905	CBYP2327.g1	GE277950
61921906	CBYP2328.b1	GE277951
61921907	CBYP2328.g1	GE277952
61921908	CBYP2329.b1	GE277953
61921909	CBYP2329.g1	GE277954
61921910	CBYP2330.b1	GE277955
61921911	CBYP2330.g1	GE277956
61921912	CBYP2331.b1	GE277957
61921913	CBYP2331.g1	GE277958
61921914	CBYP2332.b1	GE277959
61921915	CBYP2332.g1	GE277960
61921916	CBYP2333.b1	GE277961
61921917	CBYP2333.g1	GE277962
61921918	CBYP2334.b1	GE277963
61921919	CBYP2334.g1	GE277964
61921920	CBYP2336.b1	GE277965
61921921	CBYP2336.g1	GE277966
61921922	CBYP2337.b1	GE277967
61921923	CBYP2337.g1	GE277968
61921924	CBYP2338.b1	GE277969
61921925	CBYP2338.g1	GE277970
61921926	CBYP2339.b1	GE277971
61921927	CBYP2339.g1	GE277972
61921928	CBYP2340.b1	GE277973
61921929	CBYP2340.g1	GE277974
61921930	CBYP2341.b1	GE277975
61921931	CBYP2342.b1	GE277976
61921932	CBYP2342.g1	GE277977
61921933	CBYP2343.b1	GE277978
61921934	CBYP2344.b1	GE277979
61921935	CBYP2344.g1	GE277980
61921936	CBYP2345.b1	GE277981
61921937	CBYP2345.g1	GE277982
61921938	CBYP2346.b1	GE277983
61921939	CBYP2346.g1	GE277984
61921940	CBYP2347.b1	GE277985
61921941	CBYP2347.g1	GE277986
61921942	CBYP2348.b1	GE277987
61921943	CBYP2348.g1	GE277988
61921944	CBYP2350.b1	GE277989
61921945	CBYP2350.g1	GE277990
61921946	CBYP2351.b1	GE277991
61921947	CBYP2352.b1	GE277992

61921948	CBYP2352.g1	GE277993
61921949	CBYP2353.b1	GE277994
61921950	CBYP2355.b1	GE277995
61921951	CBYP2355.g1	GE277996
61921952	CBYP2356.b1	GE277997
61921953	CBYP2356.g1	GE277998
61921954	CBYP2357.b1	GE277999
61921955	CBYP2357.g1	GE278000
61921956	CBYP2358.b1	GE278001
61921957	CBYP2358.g1	GE278002
61921958	CBYP2359.b1	GE278003
61921959	CBYP2359.g1	GE278004
61921960	CBYP2360.b1	GE278005
61921961	CBYP2360.g1	GE278006
61921962	CBYP2361.b1	GE278007
61921963	CBYP2361.g1	GE278008
61921964	CBYP2362.b1	GE278009
61921965	CBYP2362.g1	GE278010
61921966	CBYP2363.b1	GE278011
61921967	CBYP2363.g1	GE278012
61921968	CBYP2364.b1	GE278013
61921969	CBYP2364.g1	GE278014
61921970	CBYP2365.b1	GE278015
61921971	CBYP2365.g1	GE278016
61921972	CBYP2366.b1	GE278017
61921973	CBYP2366.g1	GE278018
61921974	CBYP2367.b1	GE278019
61921975	CBYP2367.g1	GE278020
61921976	CBYP2368.b1	GE278021
61921977	CBYP2368.g1	GE278022
61921978	CBYP2371.b1	GE278023
61921979	CBYP2371.g1	GE278024
61921980	CBYP2372.b1	GE278025
61921981	CBYP2372.g1	GE278026
61921982	CBYP2373.b1	GE278027
61921983	CBYP2373.g1	GE278028
61921984	CBYP2374.b1	GE278029
61921985	CBYP2374.g1	GE278030
61921986	CBYP2376.b1	GE278031
61921987	CBYP2376.g1	GE278032
61921988	CBYP2377.b1	GE278033
61921989	CBYP2377.g1	GE278034
61921990	CBYP2378.b1	GE278035
61921991	CBYP2378.g1	GE278036
61921992	CBYP2379.b1	GE278037
61921993	CBYP2379.g1	GE278038
61921994	CBYP2381.b1	GE278039
61921995	CBYP2381.g1	GE278040
61921996	CBYP2382.b1	GE278041
61921997	CBYP2382.g1	GE278042
61921998	CBYP2383.b1	GE278043
61921999	CBYP2383.g1	GE278044
61922000	CBYP2384.b1	GE278045
61922001	CBYP2384.g1	GE278046
61922002	CBYP2385.b1	GE278047
61922003	CBYP2385.g1	GE278048
61922004	CBYP2386.b1	GE278049
61922005	CBYP2386.g1	GE278050
61922006	CBYP2387.b1	GE278051
61922007	CBYP2387.g1	GE278052
61922008	CBYP2388.b1	GE278053
61922009	CBYP2388.g1	GE278054
61922010	CBYP2389.b1	GE278055

61922011	CBYP2389.g1	GE278056
61922012	CBYP2391.b1	GE278057
61922013	CBYP2391.g1	GE278058
61922014	CBYP2392.b1	GE278059
61922015	CBYP2392.g1	GE278060
61922016	CBYP2393.b1	GE278061
61922017	CBYP2393.g1	GE278062
61922018	CBYP2394.b1	GE278063
61922019	CBYP2394.g1	GE278064
61922020	CBYP2395.b1	GE278065
61922021	CBYP2395.g1	GE278066
61922022	CBYP2396.b1	GE278067
61922023	CBYP2396.g1	GE278068
61922024	CBYP2397.b1	GE278069
61922025	CBYP2397.g1	GE278070
61922026	CBYP2398.b1	GE278071
61922027	CBYP2398.g1	GE278072
61922028	CBYP2399.b1	GE278073
61922029	CBYP2401.b1	GE278074
61922030	CBYP2401.g1	GE278075
61922031	CBYP2402.b1	GE278076
61922032	CBYP2402.g1	GE278077
61922033	CBYP2404.b1	GE278078
61922034	CBYP2404.g1	GE278079
61922035	CBYP2405.b1	GE278080
61922036	CBYP2405.g1	GE278081
61922037	CBYP2406.b1	GE278082
61922038	CBYP2406.g1	GE278083
61922039	CBYP2407.b1	GE278084
61922040	CBYP2407.g1	GE278085
61922041	CBYP2408.b1	GE278086
61922042	CBYP2408.g1	GE278087
61922043	CBYP2410.b1	GE278088
61922044	CBYP2410.g1	GE278089
61922045	CBYP2411.b1	GE278090
61922046	CBYP2411.g1	GE278091
61922047	CBYP2412.b1	GE278092
61922048	CBYP2412.g1	GE278093
61922049	CBYP2413.b1	GE278094
61922050	CBYP2413.g1	GE278095
61922051	CBYP2414.b1	GE278096
61922052	CBYP2414.g1	GE278097
61922053	CBYP2415.b1	GE278098
61922054	CBYP2415.g1	GE278099
61922055	CBYP2416.b1	GE278100
61922056	CBYP2417.b1	GE278101
61922057	CBYP2417.g1	GE278102
61922058	CBYP2418.b1	GE278103
61922059	CBYP2418.g1	GE278104
61922060	CBYP2419.b1	GE278105
61922061	CBYP2419.g1	GE278106
61922062	CBYP2420.b1	GE278107
61922063	CBYP2420.g1	GE278108
61922064	CBYP2421.b1	GE278109
61922065	CBYP2421.g1	GE278110
61922066	CBYP2422.b1	GE278111
61922067	CBYP2423.b1	GE278112
61922068	CBYP2423.g1	GE278113
61922069	CBYP2425.b1	GE278114
61922070	CBYP2425.g1	GE278115
61922071	CBYP2426.b1	GE278116
61922072	CBYP2426.g1	GE278117
61922073	CBYP2427.b1	GE278118

61922074	CBYP2427.g1	GE278119
61922075	CBYP2428.b1	GE278120
61922076	CBYP2429.b1	GE278121
61922077	CBYP2429.g1	GE278122
61922078	CBYP2430.b1	GE278123
61922079	CBYP2430.g1	GE278124
61922080	CBYP2431.b1	GE278125
61922081	CBYP2431.g1	GE278126
61922082	CBYP2432.b1	GE278127
61922083	CBYP2432.g1	GE278128
61922084	CBYP2433.b1	GE278129
61922085	CBYP2433.g1	GE278130
61922086	CBYP2434.b1	GE278131
61922087	CBYP2435.b1	GE278132
61922088	CBYP2435.g1	GE278133
61922089	CBYP2436.b1	GE278134
61922090	CBYP2436.g1	GE278135
61922091	CBYP2437.b1	GE278136
61922092	CBYP2437.g1	GE278137
61922093	CBYP2438.b1	GE278138
61922094	CBYP2438.g1	GE278139
61922095	CBYP2439.b1	GE278140
61922096	CBYP2439.g1	GE278141
61922097	CBYP2440.b1	GE278142
61922098	CBYP2440.g1	GE278143
61922099	CBYP2441.b1	GE278144
61922100	CBYP2441.g1	GE278145
61922101	CBYP2442.b1	GE278146
61922102	CBYP2442.g1	GE278147
61922103	CBYP2443.b1	GE278148
61922104	CBYP2443.g1	GE278149
61922105	CBYP2444.b1	GE278150
61922106	CBYP2444.g1	GE278151
61922107	CBYP2445.g1	GE278152
61922108	CBYP2448.b1	GE278153
61922109	CBYP2448.g1	GE278154
61922110	CBYP2449.b1	GE278155
61922111	CBYP2449.g1	GE278156
61922112	CBYP2450.b1	GE278157
61922113	CBYP2450.g1	GE278158
61922114	CBYP2451.b1	GE278159
61922115	CBYP2452.b1	GE278160
61922116	CBYP2452.g1	GE278161
61922117	CBYP2453.b1	GE278162
61922118	CBYP2453.g1	GE278163
61922119	CBYP2455.b1	GE278164
61922120	CBYP2455.g1	GE278165
61922121	CBYP2456.b1	GE278166
61922122	CBYP2456.g1	GE278167
61922123	CBYP2457.b1	GE278168
61922124	CBYP2457.g1	GE278169
61922125	CBYP2458.b1	GE278170
61922126	CBYP2458.g1	GE278171
61922127	CBYP2459.b1	GE278172
61922128	CBYP2459.g1	GE278173
61922129	CBYP2460.b1	GE278174
61922130	CBYP2460.g1	GE278175
61922131	CBYP2461.b1	GE278176
61922132	CBYP2461.g1	GE278177
61922133	CBYP2462.b1	GE278178
61922134	CBYP2462.g1	GE278179
61922135	CBYP2463.b1	GE278180
61922136	CBYP2463.g1	GE278181

61922137	CBYP2465.b1	GE278182
61922138	CBYP2465.g1	GE278183
61922139	CBYP2468.g1	GE278184
61922140	CBYP2469.b1	GE278185
61922141	CBYP2469.g1	GE278186
61922142	CBYP2470.b1	GE278187
61922143	CBYP2470.g1	GE278188
61922144	CBYP2471.b1	GE278189
61922145	CBYP2471.g1	GE278190
61922146	CBYP2472.b1	GE278191
61922147	CBYP2472.g1	GE278192
61922148	CBYP2473.b1	GE278193
61922149	CBYP2473.g1	GE278194
61922150	CBYP2474.b1	GE278195
61922151	CBYP2474.g1	GE278196
61922152	CBYP2475.g1	GE278197
61922153	CBYP2476.b1	GE278198
61922154	CBYP2476.g1	GE278199
61922155	CBYP2477.b1	GE278200
61922156	CBYP2477.g1	GE278201
61922157	CBYP2478.b1	GE278202
61922158	CBYP2478.g1	GE278203
61922159	CBYP2479.b1	GE278204
61922160	CBYP2479.g1	GE278205
61922161	CBYP2480.b1	GE278206
61922162	CBYP2480.g1	GE278207
61922163	CBYP2483.b1	GE278208
61922164	CBYP2483.g1	GE278209
61922165	CBYP2484.b1	GE278210
61922166	CBYP2484.g1	GE278211
61922167	CBYP2485.b1	GE278212
61922168	CBYP2485.g1	GE278213
61922169	CBYP2486.b1	GE278214
61922170	CBYP2486.g1	GE278215
61922171	CBYP2487.b1	GE278216
61922172	CBYP2487.g1	GE278217
61922173	CBYP2488.b1	GE278218
61922174	CBYP2488.g1	GE278219
61922175	CBYP2489.b1	GE278220
61922176	CBYP2489.g1	GE278221
61922177	CBYP2490.b1	GE278222
61922178	CBYP2490.g1	GE278223
61922179	CBYP2491.b1	GE278224
61922180	CBYP2491.g1	GE278225
61922181	CBYP2492.b1	GE278226
61922182	CBYP2492.g1	GE278227
61922183	CBYP2493.b1	GE278228
61922184	CBYP2493.g1	GE278229
61922185	CBYP2495.b1	GE278230
61922186	CBYP2497.b1	GE278231
61922187	CBYP2497.g1	GE278232
61922188	CBYP2498.b1	GE278233
61922189	CBYP2498.g1	GE278234
61922190	CBYP2499.b1	GE278235
61922191	CBYP2499.g1	GE278236
61922192	CBYP2500.b1	GE278237
61922193	CBYP2500.g1	GE278238
61922194	CBYP2501.b1	GE278239
61922195	CBYP2501.g1	GE278240
61922196	CBYP2502.b1	GE278241
61922197	CBYP2502.g1	GE278242
61922198	CBYP2503.b1	GE278243
61922199	CBYP2503.g1	GE278244

61922200	CBYP2504.b1	GE278245
61922201	CBYP2504.g1	GE278246
61922202	CBYP2505.b1	GE278247
61922203	CBYP2506.b1	GE278248
61922204	CBYP2506.g1	GE278249
61922205	CBYP2507.b1	GE278250
61922206	CBYP2508.b1	GE278251
61922207	CBYP2508.g1	GE278252
61922208	CBYP2509.b1	GE278253
61922209	CBYP2509.g1	GE278254
61922210	CBYP2510.b1	GE278255
61922211	CBYP2510.g1	GE278256
61922212	CBYP2511.b1	GE278257
61922213	CBYP2511.g1	GE278258
61922214	CBYP2512.b1	GE278259
61922215	CBYP2512.g1	GE278260
61922216	CBYP2513.b1	GE278261
61922217	CBYP2513.g1	GE278262
61922218	CBYP2514.b1	GE278263
61922219	CBYP2514.g1	GE278264
61922220	CBYP2515.b1	GE278265
61922221	CBYP2515.g1	GE278266
61922222	CBYP2517.b1	GE278267
61922223	CBYP2517.g1	GE278268
61922224	CBYP2518.b1	GE278269
61922225	CBYP2518.g1	GE278270
61922226	CBYP2519.b1	GE278271
61922227	CBYP2519.g1	GE278272
61922228	CBYP2520.b1	GE278273
61922229	CBYP2520.g1	GE278274
61922230	CBYP2521.b1	GE278275
61922231	CBYP2521.g1	GE278276
61922232	CBYP2522.b1	GE278277
61922233	CBYP2522.g1	GE278278
61922234	CBYP2523.b1	GE278279
61922235	CBYP2523.g1	GE278280
61922236	CBYP2524.b1	GE278281
61922237	CBYP2524.g1	GE278282
61922238	CBYP2525.b1	GE278283
61922239	CBYP2525.g1	GE278284
61922240	CBYP2526.b1	GE278285
61922241	CBYP2526.g1	GE278286
61922242	CBYP2527.b1	GE278287
61922243	CBYP2527.g1	GE278288
61922244	CBYP2528.b1	GE278289
61922245	CBYP2528.g1	GE278290
61922246	CBYP2529.b1	GE278291
61922247	CBYP2529.g1	GE278292
61922248	CBYP2530.b1	GE278293
61922249	CBYP2530.g1	GE278294
61922250	CBYP2531.b1	GE278295
61922251	CBYP2531.g1	GE278296
61922252	CBYP2532.b1	GE278297
61922253	CBYP2532.g1	GE278298
61922254	CBYP2533.b1	GE278299
61922255	CBYP2533.g1	GE278300
61922256	CBYP2534.b1	GE278301
61922257	CBYP2534.g1	GE278302
61922258	CBYP2535.b1	GE278303
61922259	CBYP2535.g1	GE278304
61922260	CBYP2536.b1	GE278305
61922261	CBYP2536.g1	GE278306
61922262	CBYP2537.b1	GE278307

61922263	CBYP2537.g1	GE278308
61922264	CBYP2538.b1	GE278309
61922265	CBYP2538.g1	GE278310
61922266	CBYP2540.b1	GE278311
61922267	CBYP2540.g1	GE278312
61922268	CBYP2541.b1	GE278313
61922269	CBYP2541.g1	GE278314
61922270	CBYP2542.b1	GE278315
61922271	CBYP2542.g1	GE278316
61922272	CBYP2543.b1	GE278317
61922273	CBYP2543.g1	GE278318
61922274	CBYP2544.b1	GE278319
61922275	CBYP2544.g1	GE278320
61922276	CBYP2545.b1	GE278321
61922277	CBYP2545.g1	GE278322
61922278	CBYP2546.b1	GE278323
61922279	CBYP2546.g1	GE278324
61922280	CBYP2547.b1	GE278325
61922281	CBYP2547.g1	GE278326
61922282	CBYP2548.b1	GE278327
61922283	CBYP2548.g1	GE278328
61922284	CBYP2549.b1	GE278329
61922285	CBYP2549.g1	GE278330
61922286	CBYP2550.b1	GE278331
61922287	CBYP2550.g1	GE278332
61922288	CBYP2552.b1	GE278333
61922289	CBYP2553.b1	GE278334
61922290	CBYP2553.g1	GE278335
61922291	CBYP2554.b1	GE278336
61922292	CBYP2554.g1	GE278337
61922293	CBYP2555.b1	GE278338
61922294	CBYP2556.b1	GE278339
61922295	CBYP2558.b1	GE278340
61922296	CBYP2558.g1	GE278341
61922297	CBYP2559.b1	GE278342
61922298	CBYP2559.g1	GE278343
61922299	CBYP2560.b1	GE278344
61922300	CBYP2560.g1	GE278345
61922301	CBYP2561.b1	GE278346
61922302	CBYP2561.g1	GE278347
61922303	CBYP2563.b1	GE278348
61922304	CBYP2563.g1	GE278349
61922305	CBYP2564.b1	GE278350
61922306	CBYP2565.b1	GE278351
61922307	CBYP2565.g1	GE278352
61922308	CBYP2566.g1	GE278353
61922309	CBYP2567.b1	GE278354
61922310	CBYP2568.b1	GE278355
61922311	CBYP2568.g1	GE278356
61922312	CBYP2569.b1	GE278357
61922313	CBYP2570.b1	GE278358
61922314	CBYP2570.g1	GE278359
61922315	CBYP2571.b1	GE278360
61922316	CBYP2572.b1	GE278361
61922317	CBYP2572.g1	GE278362
61922318	CBYP2573.b1	GE278363
61922319	CBYP2573.g1	GE278364
61922320	CBYP2574.b1	GE278365
61922321	CBYP2574.g1	GE278366
61922322	CBYP2575.b1	GE278367
61922323	CBYP2575.g1	GE278368
61922324	CBYP2576.b1	GE278369
61922325	CBYP2576.g1	GE278370

61922326	CBYP2577.b1	GE278371
61922327	CBYP2577.g1	GE278372
61922328	CBYP2578.b1	GE278373
61922329	CBYP2578.g1	GE278374
61922330	CBYP2579.b1	GE278375
61922331	CBYP2579.g1	GE278376
61922332	CBYP2580.b1	GE278377
61922333	CBYP2580.g1	GE278378
61922334	CBYP2581.b1	GE278379
61922335	CBYP2581.g1	GE278380
61922336	CBYP2582.b1	GE278381
61922337	CBYP2582.g1	GE278382
61922338	CBYP2583.b1	GE278383
61922339	CBYP2583.g1	GE278384
61922340	CBYP2584.b1	GE278385
61922341	CBYP2584.g1	GE278386
61922342	CBYP2585.g1	GE278387
61922343	CBYP2586.b1	GE278388
61922344	CBYP2586.g1	GE278389
61922345	CBYP2587.b1	GE278390
61922346	CBYP2587.g1	GE278391
61922347	CBYP2588.b1	GE278392
61922348	CBYP2588.g1	GE278393
61922349	CBYP2589.b1	GE278394
61922350	CBYP2589.g1	GE278395
61922351	CBYP2591.b1	GE278396
61922352	CBYP2594.b1	GE278397
61922353	CBYP2594.g1	GE278398
61922354	CBYP2595.b1	GE278399
61922355	CBYP2595.g1	GE278400
61922356	CBYP2596.b1	GE278401
61922357	CBYP2596.g1	GE278402
61922358	CBYP2597.b1	GE278403
61922359	CBYP2597.g1	GE278404
61922360	CBYP2598.b1	GE278405
61922361	CBYP2598.g1	GE278406
61922362	CBYP2599.b1	GE278407
61922363	CBYP2599.g1	GE278408
61922364	CBYP2600.b1	GE278409
61922365	CBYP2601.b1	GE278410
61922366	CBYP2601.g1	GE278411
61922367	CBYP2602.b1	GE278412
61922368	CBYP2602.g1	GE278413
61922369	CBYP2603.b1	GE278414
61922370	CBYP2603.g1	GE278415
61922371	CBYP2604.b1	GE278416
61922372	CBYP2604.g1	GE278417
61922373	CBYP2605.b1	GE278418
61922374	CBYP2605.g1	GE278419
61922375	CBYP2606.g1	GE278420
61922376	CBYP2607.b1	GE278421
61922377	CBYP2607.g1	GE278422
61922378	CBYP2608.b1	GE278423
61922379	CBYP2608.g1	GE278424
61922380	CBYP2609.b1	GE278425
61922381	CBYP2609.g1	GE278426
61922382	CBYP2610.b1	GE278427
61922383	CBYP2611.b1	GE278428
61922384	CBYP2611.g1	GE278429
61922385	CBYP2612.b1	GE278430
61922386	CBYP2612.g1	GE278431
61922387	CBYP2613.b1	GE278432
61922388	CBYP2613.g1	GE278433

61922389	CBYP2614.b1	GE278434
61922390	CBYP2614.g1	GE278435
61922391	CBYP2615.b1	GE278436
61922392	CBYP2615.g1	GE278437
61922393	CBYP2616.b1	GE278438
61922394	CBYP2616.g1	GE278439
61922395	CBYP2617.b1	GE278440
61922396	CBYP2617.g1	GE278441
61922397	CBYP2619.b1	GE278442
61922398	CBYP2619.g1	GE278443
61922399	CBYP2620.b1	GE278444
61922400	CBYP2620.g1	GE278445
61922401	CBYP2621.b1	GE278446
61922402	CBYP2621.g1	GE278447
61922403	CBYP2622.b1	GE278448
61922404	CBYP2622.g1	GE278449
61922405	CBYP2623.b1	GE278450
61922406	CBYP2623.g1	GE278451
61922407	CBYP2624.b1	GE278452
61922408	CBYP2624.g1	GE278453
61922409	CBYP2625.b1	GE278454
61922410	CBYP2625.g1	GE278455
61922411	CBYP2626.b1	GE278456
61922412	CBYP2626.g1	GE278457
61922413	CBYP2627.b1	GE278458
61922414	CBYP2627.g1	GE278459
61922415	CBYP2628.b1	GE278460
61922416	CBYP2629.b1	GE278461
61922417	CBYP2629.g1	GE278462
61922418	CBYP2630.b1	GE278463
61922419	CBYP2630.g1	GE278464
61922420	CBYP2631.b1	GE278465
61922421	CBYP2631.g1	GE278466
61922422	CBYP2632.b1	GE278467
61922423	CBYP2632.g1	GE278468
61922424	CBYP2633.b1	GE278469
61922425	CBYP2633.g1	GE278470
61922426	CBYP2634.b1	GE278471
61922427	CBYP2634.g1	GE278472
61922428	CBYP2635.b1	GE278473
61922429	CBYP2635.g1	GE278474
61922430	CBYP2636.b1	GE278475
61922431	CBYP2636.g1	GE278476
61922432	CBYP2637.b1	GE278477
61922433	CBYP2637.g1	GE278478
61922434	CBYP2638.b1	GE278479
61922435	CBYP2638.g1	GE278480
61922436	CBYP2639.b1	GE278481
61922437	CBYP2641.b1	GE278482
61922438	CBYP2641.g1	GE278483
61922439	CBYP2642.b1	GE278484
61922440	CBYP2642.g1	GE278485
61922441	CBYP2643.b1	GE278486
61922442	CBYP2643.g1	GE278487
61922443	CBYP2644.b1	GE278488
61922444	CBYP2644.g1	GE278489
61922445	CBYP2645.b1	GE278490
61922446	CBYP2645.g1	GE278491
61922447	CBYP2646.b1	GE278492
61922448	CBYP2646.g1	GE278493
61922449	CBYP2647.b1	GE278494
61922450	CBYP2647.g1	GE278495
61922451	CBYP2648.b1	GE278496

61922452	CBYP2648.g1	GE278497
61922453	CBYP2649.b1	GE278498
61922454	CBYP2649.g1	GE278499
61922455	CBYP2650.b1	GE278500
61922456	CBYP2650.g1	GE278501
61922457	CBYP2651.b1	GE278502
61922458	CBYP2651.g1	GE278503
61922459	CBYP2652.b1	GE278504
61922460	CBYP2653.b1	GE278505
61922461	CBYP2653.g1	GE278506
61922462	CBYP2654.b1	GE278507
61922463	CBYP2654.g1	GE278508
61922464	CBYP2655.b1	GE278509
61922465	CBYP2655.g1	GE278510
61922466	CBYP2656.b1	GE278511
61922467	CBYP2656.g1	GE278512
61922468	CBYP2657.b1	GE278513
61922469	CBYP2657.g1	GE278514
61922470	CBYP2659.b1	GE278515
61922471	CBYP2659.g1	GE278516
61922472	CBYP2660.b1	GE278517
61922473	CBYP2660.g1	GE278518
61922474	CBYP2661.b1	GE278519
61922475	CBYP2661.g1	GE278520
61922476	CBYP2662.b1	GE278521
61922477	CBYP2662.g1	GE278522
61922478	CBYP2663.b1	GE278523
61922479	CBYP2664.b1	GE278524
61922480	CBYP2664.g1	GE278525
61922481	CBYP2665.b1	GE278526
61922482	CBYP2665.g1	GE278527
61922483	CBYP2666.b1	GE278528
61922484	CBYP2666.g1	GE278529
61922485	CBYP2667.b1	GE278530
61922486	CBYP2667.g1	GE278531
61922487	CBYP2668.b1	GE278532
61922488	CBYP2668.g1	GE278533
61922489	CBYP2669.b1	GE278534
61922490	CBYP2669.g1	GE278535
61922491	CBYP2670.b1	GE278536
61922492	CBYP2670.g1	GE278537
61922493	CBYP2671.b1	GE278538
61922494	CBYP2671.g1	GE278539
61922495	CBYP2672.b1	GE278540
61922496	CBYP2672.g1	GE278541
61922497	CBYP2673.b1	GE278542
61922498	CBYP2673.g1	GE278543
61922499	CBYP2674.b1	GE278544
61922500	CBYP2674.g1	GE278545
61922501	CBYP2675.b1	GE278546
61922502	CBYP2676.b1	GE278547
61922503	CBYP2676.g1	GE278548
61922504	CBYP2677.b1	GE278549
61922505	CBYP2677.g1	GE278550
61922506	CBYP2678.b1	GE278551
61922507	CBYP2678.g1	GE278552
61922508	CBYP2679.b1	GE278553
61922509	CBYP2679.g1	GE278554
61922510	CBYP2680.b1	GE278555
61922511	CBYP2680.g1	GE278556
61922512	CBYP2681.b1	GE278557
61922513	CBYP2681.g1	GE278558
61922514	CBYP2682.b1	GE278559

61922515	CBYP2682.g1	GE278560
61922516	CBYP2683.b1	GE278561
61922517	CBYP2683.g1	GE278562
61922518	CBYP2684.b1	GE278563
61922519	CBYP2684.g1	GE278564
61922520	CBYP2685.b1	GE278565
61922521	CBYP2685.g1	GE278566
61922522	CBYP2686.b1	GE278567
61922523	CBYP2686.g1	GE278568
61922524	CBYP2687.b1	GE278569
61922525	CBYP2687.g1	GE278570
61922526	CBYP2689.b1	GE278571
61922527	CBYP2689.g1	GE278572
61922528	CBYP2690.b1	GE278573
61922529	CBYP2690.g1	GE278574
61922530	CBYP2691.b1	GE278575
61922531	CBYP2692.b1	GE278576
61922532	CBYP2692.g1	GE278577
61922533	CBYP2693.b1	GE278578
61922534	CBYP2693.g1	GE278579
61922535	CBYP2694.b1	GE278580
61922536	CBYP2694.g1	GE278581
61922537	CBYP2695.b1	GE278582
61922538	CBYP2695.g1	GE278583
61922539	CBYP2696.b1	GE278584
61922540	CBYP2696.g1	GE278585
61922541	CBYP2697.b1	GE278586
61922542	CBYP2697.g1	GE278587
61922543	CBYP2698.b1	GE278588
61922544	CBYP2699.b1	GE278589
61922545	CBYP2699.g1	GE278590
61922546	CBYP2700.b1	GE278591
61922547	CBYP2700.g1	GE278592
61922548	CBYP2701.b1	GE278593
61922549	CBYP2702.b1	GE278594
61922550	CBYP2702.g1	GE278595
61922551	CBYP2703.b1	GE278596
61922552	CBYP2703.g1	GE278597
61922553	CBYP2704.b1	GE278598
61922554	CBYP2704.g1	GE278599
61922555	CBYP2705.b1	GE278600
61922556	CBYP2705.g1	GE278601
61922557	CBYP2706.b1	GE278602
61922558	CBYP2706.g1	GE278603
61922559	CBYP2707.b1	GE278604
61922560	CBYP2707.g1	GE278605
61922561	CBYP2708.b1	GE278606
61922562	CBYP2708.g1	GE278607
61922563	CBYP2709.b1	GE278608
61922564	CBYP2709.g1	GE278609
61922565	CBYP2710.b1	GE278610
61922566	CBYP2710.g1	GE278611
61922567	CBYP2711.b1	GE278612
61922568	CBYP2711.g1	GE278613
61922569	CBYP2712.b1	GE278614
61922570	CBYP2714.b1	GE278615
61922571	CBYP2714.g1	GE278616
61922572	CBYP2715.b1	GE278617
61922573	CBYP2715.g1	GE278618
61922574	CBYP2716.b1	GE278619
61922575	CBYP2716.g1	GE278620
61922576	CBYP2717.b1	GE278621
61922577	CBYP2717.g1	GE278622

61922578	CBYP2718.b1	GE278623
61922579	CBYP2718.g1	GE278624
61922580	CBYP2719.b1	GE278625
61922581	CBYP2719.g1	GE278626
61922582	CBYP2720.b1	GE278627
61922583	CBYP2720.g1	GE278628
61922584	CBYP2721.b1	GE278629
61922585	CBYP2721.g1	GE278630
61922586	CBYP2722.b1	GE278631
61922587	CBYP2722.g1	GE278632
61922588	CBYP2723.b1	GE278633
61922589	CBYP2723.g1	GE278634
61922590	CBYP2724.b1	GE278635
61922591	CBYP2724.g1	GE278636
61922592	CBYP2725.b1	GE278637
61922593	CBYP2726.b1	GE278638
61922594	CBYP2726.g1	GE278639
61922595	CBYP2727.b1	GE278640
61922596	CBYP2727.g1	GE278641
61922597	CBYP2728.b1	GE278642
61922598	CBYP2728.g1	GE278643
61922599	CBYP2729.b1	GE278644
61922600	CBYP2729.g1	GE278645
61922601	CBYP2730.b1	GE278646
61922602	CBYP2730.g1	GE278647
61922603	CBYP2731.b1	GE278648
61922604	CBYP2731.g1	GE278649
61922605	CBYP2733.b1	GE278650
61922606	CBYP2733.g1	GE278651
61922607	CBYP2734.b1	GE278652
61922608	CBYP2736.b1	GE278653
61922609	CBYP2736.g1	GE278654
61922610	CBYP2737.b1	GE278655
61922611	CBYP2737.g1	GE278656
61922612	CBYP2739.g1	GE278657
61922613	CBYP2740.b1	GE278658
61922614	CBYP2740.g1	GE278659
61922615	CBYP2742.b1	GE278660
61922616	CBYP2743.b1	GE278661
61922617	CBYP2743.g1	GE278662
61922618	CBYP2745.b1	GE278663
61922619	CBYP2745.g1	GE278664
61922620	CBYP2746.b1	GE278665
61922621	CBYP2746.g1	GE278666
61922622	CBYP2747.b1	GE278667
61922623	CBYP2747.g1	GE278668
61922624	CBYP2748.b1	GE278669
61922625	CBYP2748.g1	GE278670
61922626	CBYP2749.b1	GE278671
61922627	CBYP2749.g1	GE278672
61922628	CBYP2751.b1	GE278673
61922629	CBYP2751.g1	GE278674
61922630	CBYP2752.b1	GE278675
61922631	CBYP2753.b1	GE278676
61922632	CBYP2753.g1	GE278677
61922633	CBYP2755.b1	GE278678
61922634	CBYP2755.g1	GE278679
61922635	CBYP2756.b1	GE278680
61922636	CBYP2756.g1	GE278681
61922637	CBYP2757.g1	GE278682
61922638	CBYP2758.b1	GE278683
61922639	CBYP2758.g1	GE278684
61922640	CBYP2759.b1	GE278685

61922641	CBYP2759.g1	GE278686
61922642	CBYP2760.b1	GE278687
61922643	CBYP2760.g1	GE278688
61922644	CBYP2761.b1	GE278689
61922645	CBYP2761.g1	GE278690
61922646	CBYP2762.b1	GE278691
61922647	CBYP2762.g1	GE278692
61922648	CBYP2763.b1	GE278693
61922649	CBYP2763.g1	GE278694
61922650	CBYP2764.b1	GE278695
61922651	CBYP2764.g1	GE278696
61922652	CBYP2765.b1	GE278697
61922653	CBYP2765.g1	GE278698
61922654	CBYP2766.b1	GE278699
61922655	CBYP2766.g1	GE278700
61922656	CBYP2767.b1	GE278701
61922657	CBYP2767.g1	GE278702
61922658	CBYP2768.b1	GE278703
61922659	CBYP2768.g1	GE278704
61922660	CBYP2769.b1	GE278705
61922661	CBYP2769.g1	GE278706
61922662	CBYP2770.b1	GE278707
61922663	CBYP2770.g1	GE278708
61922664	CBYP2771.b1	GE278709
61922665	CBYP2771.g1	GE278710
61922666	CBYP2772.b1	GE278711
61922667	CBYP2772.g1	GE278712
61922668	CBYP2773.b1	GE278713
61922669	CBYP2773.g1	GE278714
61922670	CBYP2774.b1	GE278715
61922671	CBYP2774.g1	GE278716
61922672	CBYP2775.b1	GE278717
61922673	CBYP2775.g1	GE278718
61922674	CBYP2776.b1	GE278719
61922675	CBYP2776.g1	GE278720
61922676	CBYP2778.b1	GE278721
61922677	CBYP2778.g1	GE278722
61922678	CBYP2780.b1	GE278723
61922679	CBYP2780.g1	GE278724
61922680	CBYP2781.b1	GE278725
61922681	CBYP2781.g1	GE278726
61922682	CBYP2782.b1	GE278727
61922683	CBYP2782.g1	GE278728
61922684	CBYP2783.b1	GE278729
61922685	CBYP2783.g1	GE278730
61922686	CBYP2784.b1	GE278731
61922687	CBYP2784.g1	GE278732
61922688	CBYP2786.b1	GE278733
61922689	CBYP2786.g1	GE278734
61922690	CBYP2787.b1	GE278735
61922691	CBYP2787.g1	GE278736
61922692	CBYP2788.b1	GE278737
61922693	CBYP2788.g1	GE278738
61922694	CBYP2789.b1	GE278739
61922695	CBYP2789.g1	GE278740
61922696	CBYP2790.b1	GE278741
61922697	CBYP2791.b1	GE278742
61922698	CBYP2791.g1	GE278743
61922699	CBYP2792.b1	GE278744
61922700	CBYP2792.g1	GE278745
61922701	CBYP2793.b1	GE278746
61922702	CBYP2793.g1	GE278747
61922703	CBYP2794.b1	GE278748

61922704	CBYP2794.g1	GE278749
61922705	CBYP2795.b1	GE278750
61922706	CBYP2795.g1	GE278751
61922707	CBYP2796.g1	GE278752
61922708	CBYP2797.b1	GE278753
61922709	CBYP2797.g1	GE278754
61922710	CBYP2798.b1	GE278755
61922711	CBYP2798.g1	GE278756
61922712	CBYP2799.b1	GE278757
61922713	CBYP2799.g1	GE278758
61922714	CBYP2800.b1	GE278759
61922715	CBYP2800.g1	GE278760
61922716	CBYP2801.b1	GE278761
61922717	CBYP2801.g1	GE278762
61922718	CBYP2802.b1	GE278763
61922719	CBYP2802.g1	GE278764
61922720	CBYP2803.b1	GE278765
61922721	CBYP2803.g1	GE278766
61922722	CBYP2804.b1	GE278767
61922723	CBYP2804.g1	GE278768
61922724	CBYP2805.b1	GE278769
61922725	CBYP2805.g1	GE278770
61922726	CBYP2806.b1	GE278771
61922727	CBYP2806.g1	GE278772
61922728	CBYP2807.b1	GE278773
61922729	CBYP2807.g1	GE278774
61922730	CBYP2809.b1	GE278775
61922731	CBYP2809.g1	GE278776
61922732	CBYP2810.b1	GE278777
61922733	CBYP2810.g1	GE278778
61922734	CBYP2811.b1	GE278779
61922735	CBYP2811.g1	GE278780
61922736	CBYP2812.g1	GE278781
61922737	CBYP2813.b1	GE278782
61922738	CBYP2813.g1	GE278783
61922739	CBYP2815.b1	GE278784
61922740	CBYP2815.g1	GE278785
61922741	CBYP2816.b1	GE278786
61922742	CBYP2816.g1	GE278787
61922743	CBYP2818.b1	GE278788
61922744	CBYP2818.g1	GE278789
61922745	CBYP2819.b1	GE278790
61922746	CBYP2819.g1	GE278791
61922747	CBYP2820.b1	GE278792
61922748	CBYP2820.g1	GE278793
61922749	CBYP2821.b1	GE278794
61922750	CBYP2821.g1	GE278795
61922751	CBYP2823.b1	GE278796
61922752	CBYP2823.g1	GE278797
61922753	CBYP2825.b1	GE278798
61922754	CBYP2825.g1	GE278799
61922755	CBYP2826.b1	GE278800
61922756	CBYP2826.g1	GE278801
61922757	CBYP2827.b1	GE278802
61922758	CBYP2827.g1	GE278803
61922759	CBYP2828.b1	GE278804
61922760	CBYP2829.b1	GE278805
61922761	CBYP2829.g1	GE278806
61922762	CBYP2830.b1	GE278807
61922763	CBYP2830.g1	GE278808
61922764	CBYP2831.b1	GE278809
61922765	CBYP2831.g1	GE278810
61922766	CBYP2833.b1	GE278811

61922767	CBYP2833.g1	GE278812
61922768	CBYP2834.b1	GE278813
61922769	CBYP2834.g1	GE278814
61922770	CBYP2835.b1	GE278815
61922771	CBYP2835.g1	GE278816
61922772	CBYP2836.b1	GE278817
61922773	CBYP2836.g1	GE278818
61922774	CBYP2838.b1	GE278819
61922775	CBYP2839.b1	GE278820
61922776	CBYP2839.g1	GE278821
61922777	CBYP2840.b1	GE278822
61922778	CBYP2840.g1	GE278823
61922779	CBYP2841.b1	GE278824
61922780	CBYP2841.g1	GE278825
61922781	CBYP2842.b1	GE278826
61922782	CBYP2842.g1	GE278827
61922783	CBYP2843.b1	GE278828
61922784	CBYP2843.g1	GE278829
61922785	CBYP2844.b1	GE278830
61922786	CBYP2844.g1	GE278831
61922787	CBYP2845.b1	GE278832
61922788	CBYP2845.g1	GE278833
61922789	CBYP2847.b1	GE278834
61922790	CBYP2847.g1	GE278835
61922791	CBYP2848.b1	GE278836
61922792	CBYP2848.g1	GE278837
61922793	CBYP2849.b1	GE278838
61922794	CBYP2849.g1	GE278839
61922795	CBYP2850.b1	GE278840
61922796	CBYP2850.g1	GE278841
61922797	CBYP2851.b1	GE278842
61922798	CBYP2852.b1	GE278843
61922799	CBYP2852.g1	GE278844
61922800	CBYP2853.b1	GE278845
61922801	CBYP2853.g1	GE278846
61922802	CBYP2854.b1	GE278847
61922803	CBYP2854.g1	GE278848
61922804	CBYP2855.b1	GE278849
61922805	CBYP2855.g1	GE278850
61922806	CBYP2856.b1	GE278851
61922807	CBYP2856.g1	GE278852
61922808	CBYP2858.b1	GE278853
61922809	CBYP2858.g1	GE278854
61922810	CBYP2859.b1	GE278855
61922811	CBYP2859.g1	GE278856
61922812	CBYP2860.b1	GE278857
61922813	CBYP2861.g1	GE278858
61922814	CBYP2862.b1	GE278859
61922815	CBYP2862.g1	GE278860
61922816	CBYP2863.b1	GE278861
61922817	CBYP2863.g1	GE278862
61922818	CBYP2864.b1	GE278863
61922819	CBYP2865.b1	GE278864
61922820	CBYP2865.g1	GE278865
61922821	CBYP2866.b1	GE278866
61922822	CBYP2866.g1	GE278867
61922823	CBYP2867.b1	GE278868
61922824	CBYP2867.g1	GE278869
61922825	CBYP2868.b1	GE278870
61922826	CBYP2868.g1	GE278871
61922827	CBYP2869.b1	GE278872
61922828	CBYP2869.g1	GE278873
61922829	CBYP2870.g1	GE278874

61922830	CBYP2871.b1	GE278875
61922831	CBYP2871.g1	GE278876
61922832	CBYP2872.b1	GE278877
61922833	CBYP2873.b1	GE278878
61922834	CBYP2873.g1	GE278879
61922835	CBYP2874.b1	GE278880
61922836	CBYP2874.g1	GE278881
61922837	CBYP2875.b1	GE278882
61922838	CBYP2875.g1	GE278883
61922839	CBYP2876.b1	GE278884
61922840	CBYP2876.g1	GE278885
61922841	CBYP2877.b1	GE278886
61922842	CBYP2877.g1	GE278887
61922843	CBYP2878.b1	GE278888
61922844	CBYP2879.b1	GE278889
61922845	CBYP2879.g1	GE278890
61922846	CBYP2880.b1	GE278891
61922847	CBYP2880.g1	GE278892
61922848	CBYP2881.b1	GE278893
61922849	CBYP2881.g1	GE278894
61922850	CBYP2882.b1	GE278895
61922851	CBYP2882.g1	GE278896
61922852	CBYP2883.b1	GE278897
61922853	CBYP2884.b1	GE278898
61922854	CBYP2886.g1	GE278899
61922855	CBYP2887.b1	GE278900
61922856	CBYP2887.g1	GE278901
61922857	CBYP2888.b1	GE278902
61922858	CBYP2888.g1	GE278903
61922859	CBYP2889.b1	GE278904
61922860	CBYP2889.g1	GE278905
61922861	CBYP2890.b1	GE278906
61922862	CBYP2890.g1	GE278907
61922863	CBYP2891.b1	GE278908
61922864	CBYP2891.g1	GE278909
61922865	CBYP2892.b1	GE278910
61922866	CBYP2892.g1	GE278911
61922867	CBYP2893.b1	GE278912
61922868	CBYP2893.g1	GE278913
61922869	CBYP2894.b1	GE278914
61922870	CBYP2894.g1	GE278915
61922871	CBYP2895.b1	GE278916
61922872	CBYP2895.g1	GE278917
61922873	CBYP2896.b1	GE278918
61922874	CBYP2896.g1	GE278919
61922875	CBYP2897.b1	GE278920
61922876	CBYP2897.g1	GE278921
61922877	CBYP2898.b1	GE278922
61922878	CBYP2898.g1	GE278923
61922879	CBYP2899.b1	GE278924
61922880	CBYP2899.g1	GE278925
61922881	CBYP2900.b1	GE278926
61922882	CBYP2900.g1	GE278927
61922883	CBYP2901.b1	GE278928
61922884	CBYP2901.g1	GE278929
61922885	CBYP2903.g1	GE278930
61922886	CBYP2904.b1	GE278931
61922887	CBYP2904.g1	GE278932
61922888	CBYP2905.b1	GE278933
61922889	CBYP2907.b1	GE278934
61922890	CBYP2907.g1	GE278935
61922891	CBYP2908.b1	GE278936
61922892	CBYP2908.g1	GE278937

61922893	CBYP2909.b1	GE278938
61922894	CBYP2909.g1	GE278939
61922895	CBYP2910.b1	GE278940
61922896	CBYP2910.g1	GE278941
61922897	CBYP2911.b1	GE278942
61922898	CBYP2911.g1	GE278943
61922899	CBYP2912.b1	GE278944
61922900	CBYP2912.g1	GE278945
61922901	CBYP2913.b1	GE278946
61922902	CBYP2913.g1	GE278947
61922903	CBYP2914.b1	GE278948
61922904	CBYP2914.g1	GE278949
61922905	CBYP2915.b1	GE278950
61922906	CBYP2915.g1	GE278951
61922907	CBYP2916.b1	GE278952
61922908	CBYP2916.g1	GE278953
61922909	CBYP2918.b1	GE278954
61922910	CBYP2918.g1	GE278955
61922911	CBYP2919.b1	GE278956
61922912	CBYP2919.g1	GE278957
61922913	CBYP2920.b1	GE278958
61922914	CBYP2920.g1	GE278959
61922915	CBYP2921.b1	GE278960
61922916	CBYP2921.g1	GE278961
61922917	CBYP2923.b1	GE278962
61922918	CBYP2923.g1	GE278963
61922919	CBYP2924.b1	GE278964
61922920	CBYP2924.g1	GE278965
61922921	CBYP2925.b1	GE278966
61922922	CBYP2925.g1	GE278967
61922923	CBYP2926.b1	GE278968
61922924	CBYP2926.g1	GE278969
61922925	CBYP2927.b1	GE278970
61922926	CBYP2927.g1	GE278971
61922927	CBYP2929.b1	GE278972
61922928	CBYP2929.g1	GE278973
61922929	CBYP2930.b1	GE278974
61922930	CBYP2930.g1	GE278975
61922931	CBYP2931.b1	GE278976
61922932	CBYP2931.g1	GE278977
61922933	CBYP2932.b1	GE278978
61922934	CBYP2932.g1	GE278979
61922935	CBYP2934.b1	GE278980
61922936	CBYP2935.b1	GE278981
61922937	CBYP2935.g1	GE278982
61922938	CBYP2936.b1	GE278983
61922939	CBYP2936.g1	GE278984
61922940	CBYP2937.b1	GE278985
61922941	CBYP2937.g1	GE278986
61922942	CBYP2939.b1	GE278987
61922943	CBYP2939.g1	GE278988
61922944	CBYP2940.b1	GE278989
61922945	CBYP2941.b1	GE278990
61922946	CBYP2941.g1	GE278991
61922947	CBYP2942.b1	GE278992
61922948	CBYP2942.g1	GE278993
61922949	CBYP2943.b1	GE278994
61922950	CBYP2943.g1	GE278995
61922951	CBYP2944.b1	GE278996
61922952	CBYP2944.g1	GE278997
61922953	CBYP2945.b1	GE278998
61922954	CBYP2945.g1	GE278999
61922955	CBYP2946.b1	GE279000

61922956	CBYP2946.g1	GE279001
61922957	CBYP2947.b1	GE279002
61922958	CBYP2947.g1	GE279003
61922959	CBYP2948.b1	GE279004
61922960	CBYP2948.g1	GE279005
61922961	CBYP2949.b1	GE279006
61922962	CBYP2949.g1	GE279007
61922963	CBYP2950.b1	GE279008
61922964	CBYP2950.g1	GE279009
61922965	CBYP2951.b1	GE279010
61922966	CBYP2951.g1	GE279011
61922967	CBYP2953.b1	GE279012
61922968	CBYP2953.g1	GE279013
61922969	CBYP2954.b1	GE279014
61922970	CBYP2954.g1	GE279015
61922971	CBYP2955.b1	GE279016
61922972	CBYP2955.g1	GE279017
61922973	CBYP2956.b1	GE279018
61922974	CBYP2956.g1	GE279019
61922975	CBYP2957.b1	GE279020
61922976	CBYP2957.g1	GE279021
61922977	CBYP2958.b1	GE279022
61922978	CBYP2959.b1	GE279023
61922979	CBYP2959.g1	GE279024
61922980	CBYP2960.b1	GE279025
61922981	CBYP2960.g1	GE279026
61922982	CBYP2961.b1	GE279027
61922983	CBYP2961.g1	GE279028
61922984	CBYP2962.b1	GE279029
61922985	CBYP2962.g1	GE279030
61922986	CBYP2963.b1	GE279031
61922987	CBYP2963.g1	GE279032
61922988	CBYP2964.b1	GE279033
61922989	CBYP2964.g1	GE279034
61922990	CBYP2965.b1	GE279035
61922991	CBYP2965.g1	GE279036
61922992	CBYP2966.b1	GE279037
61922993	CBYP2966.g1	GE279038
61922994	CBYP2967.b1	GE279039
61922995	CBYP2967.g1	GE279040
61922996	CBYP2968.b1	GE279041
61922997	CBYP2968.g1	GE279042
61922998	CBYP2969.b1	GE279043
61922999	CBYP2969.g1	GE279044
61923000	CBYP2970.b1	GE279045
61923001	CBYP2970.g1	GE279046
61923002	CBYP2971.b1	GE279047
61923003	CBYP2971.g1	GE279048
61923004	CBYP2972.b1	GE279049
61923005	CBYP2972.g1	GE279050
61923006	CBYP2973.b1	GE279051
61923007	CBYP2973.g1	GE279052
61923008	CBYP2974.b1	GE279053
61923009	CBYP2974.g1	GE279054
61923010	CBYP2975.b1	GE279055
61923011	CBYP2975.g1	GE279056
61923012	CBYP2977.b1	GE279057
61923013	CBYP2977.g1	GE279058
61923014	CBYP2978.b1	GE279059
61923015	CBYP2978.g1	GE279060
61923016	CBYP2979.b1	GE279061
61923017	CBYP2979.g1	GE279062
61923018	CBYP2980.b1	GE279063

61923019	CBYP2980.g1	GE279064
61923020	CBYP2981.b1	GE279065
61923021	CBYP2981.g1	GE279066
61923022	CBYP2982.b1	GE279067
61923023	CBYP2982.g1	GE279068
61923024	CBYP2983.b1	GE279069
61923025	CBYP2983.g1	GE279070
61923026	CBYP2984.b1	GE279071
61923027	CBYP2984.g1	GE279072
61923028	CBYP2985.b1	GE279073
61923029	CBYP2985.g1	GE279074
61923030	CBYP2986.b1	GE279075
61923031	CBYP2986.g1	GE279076
61923032	CBYP2987.b1	GE279077
61923033	CBYP2987.g1	GE279078
61923034	CBYP2988.b1	GE279079
61923035	CBYP2988.g1	GE279080
61923036	CBYP2989.b1	GE279081
61923037	CBYP2989.g1	GE279082
61923038	CBYP2990.b1	GE279083
61923039	CBYP2990.g1	GE279084
61923040	CBYP2992.b1	GE279085
61923041	CBYP2992.g1	GE279086
61923042	CBYP2993.b1	GE279087
61923043	CBYP2993.g1	GE279088
61923044	CBYP2994.b1	GE279089
61923045	CBYP2994.g1	GE279090
61923046	CBYP2995.b1	GE279091
61923047	CBYP2995.g1	GE279092
61923048	CBYP2996.b1	GE279093
61923049	CBYP2996.g1	GE279094
61923050	CBYP2997.b1	GE279095
61923051	CBYP2997.g1	GE279096
61923052	CBYP2998.b1	GE279097
61923053	CBYP2998.g1	GE279098
61923054	CBYP2999.b1	GE279099
61923055	CBYP2999.g1	GE279100
61923056	CBYP3000.b1	GE279101
61923057	CBYP3000.g1	GE279102
61923058	CBYP3001.b1	GE279103
61923059	CBYP3001.g1	GE279104
61923060	CBYP3002.b1	GE279105
61923061	CBYP3002.g1	GE279106
61923062	CBYP3003.b1	GE279107
61923063	CBYP3003.g1	GE279108
61923064	CBYP3004.b1	GE279109
61923065	CBYP3004.g1	GE279110
61923066	CBYP3006.b1	GE279111
61923067	CBYP3006.g1	GE279112
61923068	CBYP3007.b1	GE279113
61923069	CBYP3007.g1	GE279114
61923070	CBYP3008.b1	GE279115
61923071	CBYP3008.g1	GE279116
61923072	CBYP3009.b1	GE279117
61923073	CBYP3009.g1	GE279118
61923074	CBYP3010.b1	GE279119
61923075	CBYP3010.g1	GE279120
61923076	CBYP3011.b1	GE279121
61923077	CBYP3011.g1	GE279122
61923078	CBYP3012.b1	GE279123
61923079	CBYP3012.g1	GE279124
61923080	CBYP3013.b1	GE279125
61923081	CBYP3013.g1	GE279126

61923082	CBYP3014.b1	GE279127
61923083	CBYP3014.g1	GE279128
61923084	CBYP3015.b1	GE279129
61923085	CBYP3015.g1	GE279130
61923086	CBYP3016.b1	GE279131
61923087	CBYP3016.g1	GE279132
61923088	CBYP3017.b1	GE279133
61923089	CBYP3017.g1	GE279134
61923090	CBYP3018.b1	GE279135
61923091	CBYP3018.g1	GE279136
61923092	CBYP3019.b1	GE279137
61923093	CBYP3019.g1	GE279138
61923094	CBYP3020.b1	GE279139
61923095	CBYP3020.g1	GE279140
61923096	CBYP3021.b1	GE279141
61923097	CBYP3021.g1	GE279142
61923098	CBYP3023.b1	GE279143
61923099	CBYP3023.g1	GE279144
61923100	CBYP3024.b1	GE279145
61923101	CBYP3024.g1	GE279146
61923102	CBYP3025.b1	GE279147
61923103	CBYP3025.g1	GE279148
61923104	CBYP3026.b1	GE279149
61923105	CBYP3026.g1	GE279150
61923106	CBYP3028.b1	GE279151
61923107	CBYP3028.g1	GE279152
61923108	CBYP3030.b1	GE279153
61923109	CBYP3030.g1	GE279154
61923110	CBYP3031.b1	GE279155
61923111	CBYP3031.g1	GE279156
61923112	CBYP3034.b1	GE279157
61923113	CBYP3034.g1	GE279158
61923114	CBYP3035.b1	GE279159
61923115	CBYP3036.b1	GE279160
61923116	CBYP3036.g1	GE279161
61923117	CBYP3038.b1	GE279162
61923118	CBYP3038.g1	GE279163
61923119	CBYP3039.b1	GE279164
61923120	CBYP3039.g1	GE279165
61923121	CBYP3040.b1	GE279166
61923122	CBYP3040.g1	GE279167
61923123	CBYP3041.b1	GE279168
61923124	CBYP3041.g1	GE279169
61923125	CBYP3042.b1	GE279170
61923126	CBYP3042.g1	GE279171
61923127	CBYP3043.b1	GE279172
61923128	CBYP3043.g1	GE279173
61923129	CBYP3044.b1	GE279174
61923130	CBYP3044.g1	GE279175
61923131	CBYP3045.b1	GE279176
61923132	CBYP3045.g1	GE279177
61923133	CBYP3047.b1	GE279178
61923134	CBYP3048.b1	GE279179
61923135	CBYP3049.b1	GE279180
61923136	CBYP3049.g1	GE279181
61923137	CBYP3050.b1	GE279182
61923138	CBYP3050.g1	GE279183
61923139	CBYP3051.b1	GE279184
61923140	CBYP3051.g1	GE279185
61923141	CBYP3052.b1	GE279186
61923142	CBYP3052.g1	GE279187
61923143	CBYP3053.b1	GE279188
61923144	CBYP3053.g1	GE279189

61923145	CBYP3054.b1	GE279190
61923146	CBYP3054.g1	GE279191
61923147	CBYP3055.b1	GE279192
61923148	CBYP3055.g1	GE279193
61923149	CBYP3056.b1	GE279194
61923150	CBYP3056.g1	GE279195
61923151	CBYP3057.b1	GE279196
61923152	CBYP3057.g1	GE279197
61923153	CBYP3058.b1	GE279198
61923154	CBYP3058.g1	GE279199
61923155	CBYP3059.b1	GE279200
61923156	CBYP3059.g1	GE279201
61923157	CBYP3060.b1	GE279202
61923158	CBYP3060.g1	GE279203
61923159	CBYP3061.b1	GE279204
61923160	CBYP3061.g1	GE279205
61923161	CBYP3062.b1	GE279206
61923162	CBYP3064.b1	GE279207
61923163	CBYP3064.g1	GE279208
61923164	CBYP3065.b1	GE279209
61923165	CBYP3065.g1	GE279210
61923166	CBYP3066.b1	GE279211
61923167	CBYP3066.g1	GE279212
61923168	CBYP3067.b1	GE279213
61923169	CBYP3067.g1	GE279214
61923170	CBYP3068.b1	GE279215
61923171	CBYP3068.g1	GE279216
61923172	CBYP3069.b1	GE279217
61923173	CBYP3069.g1	GE279218
61923174	CBYP3070.b1	GE279219
61923175	CBYP3070.g1	GE279220
61923176	CBYP3071.b1	GE279221
61923177	CBYP3071.g1	GE279222
61923178	CBYP3073.g1	GE279223
61923179	CBYP3074.b1	GE279224
61923180	CBYP3074.g1	GE279225
61923181	CBYP3075.b1	GE279226
61923182	CBYP3075.g1	GE279227
61923183	CBYP3076.b1	GE279228
61923184	CBYP3076.g1	GE279229
61923185	CBYP3077.b1	GE279230
61923186	CBYP3077.g1	GE279231
61923187	CBYP3078.g1	GE279232
61923188	CBYP3079.b1	GE279233
61923189	CBYP3080.b1	GE279234
61923190	CBYP3080.g1	GE279235
61923191	CBYP3081.b1	GE279236
61923192	CBYP3081.g1	GE279237
61923193	CBYP3082.b1	GE279238
61923194	CBYP3082.g1	GE279239
61923195	CBYP3084.b1	GE279240
61923196	CBYP3084.g1	GE279241
61923197	CBYP3085.b1	GE279242
61923198	CBYP3085.g1	GE279243
61923199	CBYP3086.g1	GE279244
61923200	CBYP3087.b1	GE279245
61923201	CBYP3087.g1	GE279246
61923202	CBYP3088.b1	GE279247
61923203	CBYP3088.g1	GE279248
61923204	CBYP3091.b1	GE279249
61923205	CBYP3091.g1	GE279250
61923206	CBYP3092.b1	GE279251
61923207	CBYP3092.g1	GE279252

61923208	CBYP3093.b1	GE279253
61923209	CBYP3093.g1	GE279254
61923210	CBYP3094.b1	GE279255
61923211	CBYP3094.g1	GE279256
61923212	CBYP3095.b1	GE279257
61923213	CBYP3095.g1	GE279258
61923214	CBYP3096.g1	GE279259
61923215	CBYP3097.b1	GE279260
61923216	CBYP3097.g1	GE279261
61923217	CBYP3098.b1	GE279262
61923218	CBYP3098.g1	GE279263
61923219	CBYP3099.b1	GE279264
61923220	CBYP3099.g1	GE279265
61923221	CBYP3101.b1	GE279266
61923222	CBYP3101.g1	GE279267
61923223	CBYP3102.b1	GE279268
61923224	CBYP3102.g1	GE279269
61923225	CBYP3103.b1	GE279270
61923226	CBYP3103.g1	GE279271
61923227	CBYP3104.b1	GE279272
61923228	CBYP3104.g1	GE279273
61923229	CBYP3105.g1	GE279274
61923230	CBYP3106.b1	GE279275
61923231	CBYP3106.g1	GE279276
61923232	CBYP3107.b1	GE279277
61923233	CBYP3107.g1	GE279278
61923234	CBYP3109.b1	GE279279
61923235	CBYP3109.g1	GE279280
61923236	CBYP3110.b1	GE279281
61923237	CBYP3110.g1	GE279282
61923238	CBYP3111.b1	GE279283
61923239	CBYP3111.g1	GE279284
61923240	CBYP3112.b1	GE279285
61923241	CBYP3112.g1	GE279286
61923242	CBYP3113.b1	GE279287
61923243	CBYP3113.g1	GE279288
61923244	CBYP3114.b1	GE279289
61923245	CBYP3114.g1	GE279290
61923246	CBYP3115.b1	GE279291
61923247	CBYP3115.g1	GE279292
61923248	CBYP3116.b1	GE279293
61923249	CBYP3116.g1	GE279294
61923250	CBYP3117.b1	GE279295
61923251	CBYP3117.g1	GE279296
61923252	CBYP3118.b1	GE279297
61923253	CBYP3118.g1	GE279298
61923254	CBYP3119.b1	GE279299
61923255	CBYP3119.g1	GE279300
61923256	CBYP3120.b1	GE279301
61923257	CBYP3121.b1	GE279302
61923258	CBYP3122.b1	GE279303
61923259	CBYP3122.g1	GE279304
61923260	CBYP3124.b1	GE279305
61923261	CBYP3124.g1	GE279306
61923262	CBYP3125.b1	GE279307
61923263	CBYP3125.g1	GE279308
61923264	CBYP3126.b1	GE279309
61923265	CBYP3126.g1	GE279310
61923266	CBYP3127.b1	GE279311
61923267	CBYP3127.g1	GE279312
61923268	CBYP3128.b1	GE279313
61923269	CBYP3128.g1	GE279314
61923270	CBYP3129.b1	GE279315

61923271	CBYP3130.b1	GE279316
61923272	CBYP3130.g1	GE279317
61923273	CBYP3131.b1	GE279318
61923274	CBYP3131.g1	GE279319
61923275	CBYP3132.b1	GE279320
61923276	CBYP3132.g1	GE279321
61923277	CBYP3133.b1	GE279322
61923278	CBYP3133.g1	GE279323
61923279	CBYP3134.b1	GE279324
61923280	CBYP3134.g1	GE279325
61923281	CBYP3135.g1	GE279326
61923282	CBYP3136.b1	GE279327
61923283	CBYP3136.g1	GE279328
61923284	CBYP3137.b1	GE279329
61923285	CBYP3137.g1	GE279330
61923286	CBYP3138.b1	GE279331
61923287	CBYP3138.g1	GE279332
61923288	CBYP3139.b1	GE279333
61923289	CBYP3139.g1	GE279334
61923290	CBYP3140.b1	GE279335
61923291	CBYP3143.g1	GE279336
61923292	CBYP3144.b1	GE279337
61923293	CBYP3144.g1	GE279338
61923294	CBYP3146.b1	GE279339
61923295	CBYP3146.g1	GE279340
61923296	CBYP3147.b1	GE279341
61923297	CBYP3147.g1	GE279342
61923298	CBYP3149.b1	GE279343
61923299	CBYP3149.g1	GE279344
61923300	CBYP3151.b1	GE279345
61923301	CBYP3151.g1	GE279346
61923302	CBYP3152.b1	GE279347
61923303	CBYP3152.g1	GE279348
61923304	CBYP3153.b1	GE279349
61923305	CBYP3153.g1	GE279350
61923306	CBYP3154.b1	GE279351
61923307	CBYP3154.g1	GE279352
61923308	CBYP3155.b1	GE279353
61923309	CBYP3155.g1	GE279354
61923310	CBYP3156.b1	GE279355
61923311	CBYP3156.g1	GE279356
61923312	CBYP3157.b1	GE279357
61923313	CBYP3157.g1	GE279358
61923314	CBYP3158.b1	GE279359
61923315	CBYP3159.b1	GE279360
61923316	CBYP3159.g1	GE279361
61923317	CBYP3160.b1	GE279362
61923318	CBYP3160.g1	GE279363
61923319	CBYP3161.b1	GE279364
61923320	CBYP3161.g1	GE279365
61923321	CBYP3162.b1	GE279366
61923322	CBYP3162.g1	GE279367
61923323	CBYP3163.b1	GE279368
61923324	CBYP3163.g1	GE279369
61923325	CBYP3164.b1	GE279370
61923326	CBYP3164.g1	GE279371
61923327	CBYP3167.b1	GE279372
61923328	CBYP3167.g1	GE279373
61923329	CBYP3168.b1	GE279374
61923330	CBYP3168.g1	GE279375
61923331	CBYP3169.b1	GE279376
61923332	CBYP3169.g1	GE279377
61923333	CBYP3170.b1	GE279378

61923334	CBYP3170.g1	GE279379
61923335	CBYP3171.b1	GE279380
61923336	CBYP3171.g1	GE279381
61923337	CBYP3172.b1	GE279382
61923338	CBYP3172.g1	GE279383
61923339	CBYP3173.b1	GE279384
61923340	CBYP3174.b1	GE279385
61923341	CBYP3174.g1	GE279386
61923342	CBYP3175.b1	GE279387
61923343	CBYP3175.g1	GE279388
61923344	CBYP3176.b1	GE279389
61923345	CBYP3177.b1	GE279390
61923346	CBYP3177.g1	GE279391
61923347	CBYP3178.b1	GE279392
61923348	CBYP3178.g1	GE279393
61923349	CBYP3179.b1	GE279394
61923350	CBYP3179.g1	GE279395
61923351	CBYP3180.b1	GE279396
61923352	CBYP3180.g1	GE279397
61923353	CBYP3181.b1	GE279398
61923354	CBYP3181.g1	GE279399
61923355	CBYP3182.b1	GE279400
61923356	CBYP3182.g1	GE279401
61923357	CBYP3183.b1	GE279402
61923358	CBYP3183.g1	GE279403
61923359	CBYP3184.b1	GE279404
61923360	CBYP3184.g1	GE279405
61923361	CBYP3186.b1	GE279406
61923362	CBYP3186.g1	GE279407
61923363	CBYP3187.b1	GE279408
61923364	CBYP3187.g1	GE279409
61923365	CBYP3188.b1	GE279410
61923366	CBYP3188.g1	GE279411
61923367	CBYP3189.b1	GE279412
61923368	CBYP3189.g1	GE279413
61923369	CBYP3190.b1	GE279414
61923370	CBYP3190.g1	GE279415
61923371	CBYP3191.b1	GE279416
61923372	CBYP3191.g1	GE279417
61923373	CBYP3192.b1	GE279418
61923374	CBYP3193.b1	GE279419
61923375	CBYP3193.g1	GE279420
61923376	CBYP3194.b1	GE279421
61923377	CBYP3194.g1	GE279422
61923378	CBYP3195.b1	GE279423
61923379	CBYP3195.g1	GE279424
61923380	CBYP3196.b1	GE279425
61923381	CBYP3196.g1	GE279426
61923382	CBYP3197.b1	GE279427
61923383	CBYP3197.g1	GE279428
61923384	CBYP3198.b1	GE279429
61923385	CBYP3198.g1	GE279430
61923386	CBYP3199.b1	GE279431
61923387	CBYP3199.g1	GE279432
61923388	CBYP3200.b1	GE279433
61923389	CBYP3200.g1	GE279434
61923390	CBYP3201.b1	GE279435
61923391	CBYP3201.g1	GE279436
61923392	CBYP3202.b1	GE279437
61923393	CBYP3202.g1	GE279438
61923394	CBYP3203.b1	GE279439
61923395	CBYP3203.g1	GE279440
61923396	CBYP3204.b1	GE279441

61923397	CBYP3204.g1	GE279442
61923398	CBYP3205.g1	GE279443
61923399	CBYP3206.b1	GE279444
61923400	CBYP3206.g1	GE279445
61923401	CBYP3207.b1	GE279446
61923402	CBYP3207.g1	GE279447
61923403	CBYP3209.b1	GE279448
61923404	CBYP3209.g1	GE279449
61923405	CBYP3210.b1	GE279450
61923406	CBYP3210.g1	GE279451
61923407	CBYP3211.b1	GE279452
61923408	CBYP3211.g1	GE279453
61923409	CBYP3212.b1	GE279454
61923410	CBYP3212.g1	GE279455
61923411	CBYP3213.b1	GE279456
61923412	CBYP3213.g1	GE279457
61923413	CBYP3214.b1	GE279458
61923414	CBYP3214.g1	GE279459
61923415	CBYP3216.b1	GE279460
61923416	CBYP3216.g1	GE279461
61923417	CBYP3217.b1	GE279462
61923418	CBYP3217.g1	GE279463
61923419	CBYP3218.b1	GE279464
61923420	CBYP3218.g1	GE279465
61923421	CBYP3219.b1	GE279466
61923422	CBYP3221.b1	GE279467
61923423	CBYP3221.g1	GE279468
61923424	CBYP3222.b1	GE279469
61923425	CBYP3223.b1	GE279470
61923426	CBYP3223.g1	GE279471
61923427	CBYP3224.b1	GE279472
61923428	CBYP3224.g1	GE279473
61923429	CBYP3225.b1	GE279474
61923430	CBYP3225.g1	GE279475
61923431	CBYP3226.b1	GE279476
61923432	CBYP3226.g1	GE279477
61923433	CBYP3227.b1	GE279478
61923434	CBYP3227.g1	GE279479
61923435	CBYP3228.b1	GE279480
61923436	CBYP3228.g1	GE279481
61923437	CBYP3229.b1	GE279482
61923438	CBYP3229.g1	GE279483
61923439	CBYP3230.b1	GE279484
61923440	CBYP3230.g1	GE279485
61923441	CBYP3231.b1	GE279486
61923442	CBYP3231.g1	GE279487
61923443	CBYP3232.b1	GE279488
61923444	CBYP3232.g1	GE279489
61923445	CBYP3233.b1	GE279490
61923446	CBYP3233.g1	GE279491
61923447	CBYP3234.b1	GE279492
61923448	CBYP3234.g1	GE279493
61923449	CBYP3235.b1	GE279494
61923450	CBYP3236.b1	GE279495
61923451	CBYP3236.g1	GE279496
61923452	CBYP3239.b1	GE279497
61923453	CBYP3239.g1	GE279498
61923454	CBYP3240.b1	GE279499
61923455	CBYP3241.b1	GE279500
61923456	CBYP3242.b1	GE279501
61923457	CBYP3242.g1	GE279502
61923458	CBYP3243.b1	GE279503
61923459	CBYP3243.g1	GE279504

61923460	CBYP3244.b1	GE279505
61923461	CBYP3244.g1	GE279506
61923462	CBYP3245.b1	GE279507
61923463	CBYP3245.g1	GE279508
61923464	CBYP3246.b1	GE279509
61923465	CBYP3246.g1	GE279510
61923466	CBYP3247.b1	GE279511
61923467	CBYP3247.g1	GE279512
61923468	CBYP3248.b1	GE279513
61923469	CBYP3248.g1	GE279514
61923470	CBYP3249.b1	GE279515
61923471	CBYP3249.g1	GE279516
61923472	CBYP3250.b1	GE279517
61923473	CBYP3250.g1	GE279518
61923474	CBYP3251.b1	GE279519
61923475	CBYP3251.g1	GE279520
61923476	CBYP3253.b1	GE279521
61923477	CBYP3253.g1	GE279522
61923478	CBYP3255.b1	GE279523
61923479	CBYP3255.g1	GE279524
61923480	CBYP3256.b1	GE279525
61923481	CBYP3256.g1	GE279526
61923482	CBYP3257.b1	GE279527
61923483	CBYP3257.g1	GE279528
61923484	CBYP3258.b1	GE279529
61923485	CBYP3258.g1	GE279530
61923486	CBYP3259.b1	GE279531
61923487	CBYP3259.g1	GE279532
61923488	CBYP3260.b1	GE279533
61923489	CBYP3260.g1	GE279534
61923490	CBYP3261.b1	GE279535
61923491	CBYP3261.g1	GE279536
61923492	CBYP3262.b1	GE279537
61923493	CBYP3262.g1	GE279538
61923494	CBYP3263.b1	GE279539
61923495	CBYP3264.b1	GE279540
61923496	CBYP3264.g1	GE279541
61923497	CBYP3265.b1	GE279542
61923498	CBYP3265.g1	GE279543
61923499	CBYP3266.b1	GE279544
61923500	CBYP3266.g1	GE279545
61923501	CBYP3267.b1	GE279546
61923502	CBYP3267.g1	GE279547
61923503	CBYP3268.b1	GE279548
61923504	CBYP3268.g1	GE279549
61923505	CBYP3269.b1	GE279550
61923506	CBYP3269.g1	GE279551
61923507	CBYP3270.b1	GE279552
61923508	CBYP3271.b1	GE279553
61923509	CBYP3271.g1	GE279554
61923510	CBYP3272.b1	GE279555
61923511	CBYP3272.g1	GE279556
61923512	CBYP3273.b1	GE279557
61923513	CBYP3273.g1	GE279558
61923514	CBYP3274.b1	GE279559
61923515	CBYP3274.g1	GE279560
61923516	CBYP3275.b1	GE279561
61923517	CBYP3275.g1	GE279562
61923518	CBYP3276.b1	GE279563
61923519	CBYP3276.g1	GE279564
61923520	CBYP3277.b1	GE279565
61923521	CBYP3277.g1	GE279566
61923522	CBYP3278.b1	GE279567

61923523	CBYP3278.g1	GE279568
61923524	CBYP3279.b1	GE279569
61923525	CBYP3279.g1	GE279570
61923526	CBYP3280.b1	GE279571
61923527	CBYP3280.g1	GE279572
61923528	CBYP3281.b1	GE279573
61923529	CBYP3281.g1	GE279574
61923530	CBYP3285.b1	GE279575
61923531	CBYP3285.g1	GE279576
61923532	CBYP3286.b1	GE279577
61923533	CBYP3286.g1	GE279578
61923534	CBYP3287.b1	GE279579
61923535	CBYP3287.g1	GE279580
61923536	CBYP3288.b1	GE279581
61923537	CBYP3289.b1	GE279582
61923538	CBYP3289.g1	GE279583
61923539	CBYP3290.b1	GE279584
61923540	CBYP3290.g1	GE279585
61923541	CBYP3291.b1	GE279586
61923542	CBYP3291.g1	GE279587
61923543	CBYP3292.b1	GE279588
61923544	CBYP3292.g1	GE279589
61923545	CBYP3293.b1	GE279590
61923546	CBYP3293.g1	GE279591
61923547	CBYP3294.b1	GE279592
61923548	CBYP3294.g1	GE279593
61923549	CBYP3295.b1	GE279594
61923550	CBYP3295.g1	GE279595
61923551	CBYP3296.b1	GE279596
61923552	CBYP3296.g1	GE279597
61923553	CBYP3297.b1	GE279598
61923554	CBYP3297.g1	GE279599
61923555	CBYP3298.b1	GE279600
61923556	CBYP3298.g1	GE279601
61923557	CBYP3299.b1	GE279602
61923558	CBYP3299.g1	GE279603
61923559	CBYP3300.g1	GE279604
61923560	CBYP3301.b1	GE279605
61923561	CBYP3301.g1	GE279606
61923562	CBYP3302.b1	GE279607
61923563	CBYP3302.g1	GE279608
61923564	CBYP3303.b1	GE279609
61923565	CBYP3303.g1	GE279610
61923566	CBYP3304.b1	GE279611
61923567	CBYP3304.g1	GE279612
61923568	CBYP3305.b1	GE279613
61923569	CBYP3305.g1	GE279614
61923570	CBYP3306.b1	GE279615
61923571	CBYP3306.g1	GE279616
61923572	CBYP3307.b1	GE279617
61923573	CBYP3307.g1	GE279618
61923574	CBYP3310.b1	GE279619
61923575	CBYP3310.g1	GE279620
61923576	CBYP3311.b1	GE279621
61923577	CBYP3311.g1	GE279622
61923578	CBYP3312.b1	GE279623
61923579	CBYP3312.g1	GE279624
61923580	CBYP3313.b1	GE279625
61923581	CBYP3313.g1	GE279626
61923582	CBYP3314.b1	GE279627
61923583	CBYP3314.g1	GE279628
61923584	CBYP3315.b1	GE279629
61923585	CBYP3315.g1	GE279630

61923586	CBYP3316.b1	GE279631
61923587	CBYP3316.g1	GE279632
61923588	CBYP3317.g1	GE279633
61923589	CBYP3318.b1	GE279634
61923590	CBYP3318.g1	GE279635
61923591	CBYP3319.b1	GE279636
61923592	CBYP3319.g1	GE279637
61923593	CBYP3320.b1	GE279638
61923594	CBYP3320.g1	GE279639
61923595	CBYP3321.b1	GE279640
61923596	CBYP3321.g1	GE279641
61923597	CBYP3322.b1	GE279642
61923598	CBYP3322.g1	GE279643
61923599	CBYP3323.b1	GE279644
61923600	CBYP3323.g1	GE279645
61923601	CBYP3324.b1	GE279646
61923602	CBYP3324.g1	GE279647
61923603	CBYP3325.b1	GE279648
61923604	CBYP3325.g1	GE279649
61923605	CBYP3326.b1	GE279650
61923606	CBYP3326.g1	GE279651
61923607	CBYP3327.b1	GE279652
61923608	CBYP3327.g1	GE279653
61923609	CBYP3328.b1	GE279654
61923610	CBYP3328.g1	GE279655
61923611	CBYP3329.b1	GE279656
61923612	CBYP3329.g1	GE279657
61923613	CBYP3330.b1	GE279658
61923614	CBYP3330.g1	GE279659
61923615	CBYP3331.b1	GE279660
61923616	CBYP3331.g1	GE279661
61923617	CBYP3332.g1	GE279662
61923618	CBYP3333.b1	GE279663
61923619	CBYP3333.g1	GE279664
61923620	CBYP3334.b1	GE279665
61923621	CBYP3334.g1	GE279666
61923622	CBYP3336.b1	GE279667
61923623	CBYP3336.g1	GE279668
61923624	CBYP3337.b1	GE279669
61923625	CBYP3337.g1	GE279670
61923626	CBYP3338.b1	GE279671
61923627	CBYP3338.g1	GE279672
61923628	CBYP3339.b1	GE279673
61923629	CBYP3339.g1	GE279674
61923630	CBYP3340.b1	GE279675
61923631	CBYP3340.g1	GE279676
61923632	CBYP3341.b1	GE279677
61923633	CBYP3341.g1	GE279678
61923634	CBYP3342.b1	GE279679
61923635	CBYP3342.g1	GE279680
61923636	CBYP3343.b1	GE279681
61923637	CBYP3344.b1	GE279682
61923638	CBYP3345.b1	GE279683
61923639	CBYP3345.g1	GE279684
61923640	CBYP3346.b1	GE279685
61923641	CBYP3347.b1	GE279686
61923642	CBYP3347.g1	GE279687
61923643	CBYP3348.b1	GE279688
61923644	CBYP3348.g1	GE279689
61923645	CBYP3349.b1	GE279690
61923646	CBYP3349.g1	GE279691
61923647	CBYP3350.b1	GE279692
61923648	CBYP3350.g1	GE279693

61923649	CBYP3351.b1	GE279694
61923650	CBYP3351.g1	GE279695
61923651	CBYP3352.b1	GE279696
61923652	CBYP3352.g1	GE279697
61923653	CBYP3353.b1	GE279698
61923654	CBYP3353.g1	GE279699
61923655	CBYP3354.b1	GE279700
61923656	CBYP3354.g1	GE279701
61923657	CBYP3355.b1	GE279702
61923658	CBYP3355.g1	GE279703
61923659	CBYP3356.b1	GE279704
61923660	CBYP3356.g1	GE279705
61923661	CBYP3357.b1	GE279706
61923662	CBYP3357.g1	GE279707
61923663	CBYP3358.b1	GE279708
61923664	CBYP3358.g1	GE279709
61923665	CBYP3359.b1	GE279710
61923666	CBYP3359.g1	GE279711
61923667	CBYP3360.b1	GE279712
61923668	CBYP3360.g1	GE279713
61923669	CBYP3361.b1	GE279714
61923670	CBYP3361.g1	GE279715
61923671	CBYP3362.b1	GE279716
61923672	CBYP3362.g1	GE279717
61923673	CBYP3364.b1	GE279718
61923674	CBYP3364.g1	GE279719
61923675	CBYP3365.b1	GE279720
61923676	CBYP3365.g1	GE279721
61923677	CBYP3366.b1	GE279722
61923678	CBYP3366.g1	GE279723
61923679	CBYP3367.g1	GE279724
61923680	CBYP3368.b1	GE279725
61923681	CBYP3369.b1	GE279726
61923682	CBYP3370.b1	GE279727
61923683	CBYP3370.g1	GE279728
61923684	CBYP3371.b1	GE279729
61923685	CBYP3371.g1	GE279730
61923686	CBYP3372.b1	GE279731
61923687	CBYP3372.g1	GE279732
61923688	CBYP3374.b1	GE279733
61923689	CBYP3374.g1	GE279734
61923690	CBYP3375.b1	GE279735
61923691	CBYP3375.g1	GE279736
61923692	CBYP3377.b1	GE279737
61923693	CBYP3377.g1	GE279738
61923694	CBYP3379.g1	GE279739
61923695	CBYP3380.b1	GE279740
61923696	CBYP3380.g1	GE279741
61923697	CBYP3381.b1	GE279742
61923698	CBYP3381.g1	GE279743
61923699	CBYP3382.b1	GE279744
61923700	CBYP3382.g1	GE279745
61923701	CBYP3383.b1	GE279746
61923702	CBYP3383.g1	GE279747
61923703	CBYP3384.b1	GE279748
61923704	CBYP3384.g1	GE279749
61923705	CBYP3385.b1	GE279750
61923706	CBYP3385.g1	GE279751
61923707	CBYP3386.b1	GE279752
61923708	CBYP3386.g1	GE279753
61923709	CBYP3387.b1	GE279754
61923710	CBYP3387.g1	GE279755
61923711	CBYP3388.b1	GE279756

61923712	CBYP3388.g1	GE279757
61923713	CBYP3389.b1	GE279758
61923714	CBYP3389.g1	GE279759
61923715	CBYP3390.b1	GE279760
61923716	CBYP3390.g1	GE279761
61923717	CBYP3391.b1	GE279762
61923718	CBYP3391.g1	GE279763
61923719	CBYP3392.b1	GE279764
61923720	CBYP3392.g1	GE279765
61923721	CBYP3393.b1	GE279766
61923722	CBYP3393.g1	GE279767
61923723	CBYP3394.b1	GE279768
61923724	CBYP3394.g1	GE279769
61923725	CBYP3395.b1	GE279770
61923726	CBYP3395.g1	GE279771
61923727	CBYP3396.b1	GE279772
61923728	CBYP3396.g1	GE279773
61923729	CBYP3397.g1	GE279774
61923730	CBYP3398.b1	GE279775
61923731	CBYP3398.g1	GE279776
61923732	CBYP3399.b1	GE279777
61923733	CBYP3399.g1	GE279778
61923734	CBYP3400.b1	GE279779
61923735	CBYP3400.g1	GE279780
61923736	CBYP3401.b1	GE279781
61923737	CBYP3401.g1	GE279782
61923738	CBYP3402.b1	GE279783
61923739	CBYP3402.g1	GE279784
61923740	CBYP3403.b1	GE279785
61923741	CBYP3403.g1	GE279786
61923742	CBYP3404.b1	GE279787
61923743	CBYP3404.g1	GE279788
61923744	CBYP3405.b1	GE279789
61923745	CBYP3405.g1	GE279790
61923746	CBYP3406.b1	GE279791
61923747	CBYP3406.g1	GE279792
61923748	CBYP3407.b1	GE279793
61923749	CBYP3407.g1	GE279794
61923750	CBYP3408.b1	GE279795
61923751	CBYP3408.g1	GE279796
61923752	CBYP3409.b1	GE279797
61923753	CBYP3409.g1	GE279798
61923754	CBYP3410.b1	GE279799
61923755	CBYP3410.g1	GE279800
61923756	CBYP3411.b1	GE279801
61923757	CBYP3411.g1	GE279802
61923758	CBYP3412.b1	GE279803
61923759	CBYP3412.g1	GE279804
61923760	CBYP3413.b1	GE279805
61923761	CBYP3413.g1	GE279806
61923762	CBYP3417.b1	GE279807
61923763	CBYP3417.g1	GE279808
61923764	CBYP3418.b1	GE279809
61923765	CBYP3418.g1	GE279810
61923766	CBYP3419.b1	GE279811
61923767	CBYP3419.g1	GE279812
61923768	CBYP3420.g1	GE279813
61923769	CBYP3421.b1	GE279814
61923770	CBYP3421.g1	GE279815
61923771	CBYP3422.b1	GE279816
61923772	CBYP3422.g1	GE279817
61923773	CBYP3423.b1	GE279818
61923774	CBYP3423.g1	GE279819

61923775	CBYP3424.b1	GE279820
61923776	CBYP3424.g1	GE279821
61923777	CBYP3425.b1	GE279822
61923778	CBYP3425.g1	GE279823
61923779	CBYP3426.b1	GE279824
61923780	CBYP3426.g1	GE279825
61923781	CBYP3427.b1	GE279826
61923782	CBYP3427.g1	GE279827
61923783	CBYP3428.b1	GE279828
61923784	CBYP3428.g1	GE279829
61923785	CBYP3429.b1	GE279830
61923786	CBYP3429.g1	GE279831
61923787	CBYP3430.b1	GE279832
61923788	CBYP3430.g1	GE279833
61923789	CBYP3431.b1	GE279834
61923790	CBYP3431.g1	GE279835
61923791	CBYP3432.b1	GE279836
61923792	CBYP3432.g1	GE279837
61923793	CBYP3433.b1	GE279838
61923794	CBYP3433.g1	GE279839
61923795	CBYP3434.b1	GE279840
61923796	CBYP3434.g1	GE279841
61923797	CBYP3435.b1	GE279842
61923798	CBYP3435.g1	GE279843
61923799	CBYP3436.b1	GE279844
61923800	CBYP3436.g1	GE279845
61923801	CBYP3437.b1	GE279846
61923802	CBYP3437.g1	GE279847
61923803	CBYP3438.b1	GE279848
61923804	CBYP3438.g1	GE279849
61923805	CBYP3439.b1	GE279850
61923806	CBYP3439.g1	GE279851
61923807	CBYP3441.b1	GE279852
61923808	CBYP3441.g1	GE279853
61923809	CBYP3442.g1	GE279854
61923810	CBYP3444.b1	GE279855
61923811	CBYP3444.g1	GE279856
61923812	CBYP3445.b1	GE279857
61923813	CBYP3445.g1	GE279858
61923814	CBYP3446.b1	GE279859
61923815	CBYP3446.g1	GE279860
61923816	CBYP3447.b1	GE279861
61923817	CBYP3448.b1	GE279862
61923818	CBYP3448.g1	GE279863
61923819	CBYP3449.b1	GE279864
61923820	CBYP3449.g1	GE279865
61923821	CBYP3450.g1	GE279866
61923822	CBYP3452.b1	GE279867
61923823	CBYP3452.g1	GE279868
61923824	CBYP3453.b1	GE279869
61923825	CBYP3453.g1	GE279870
61923826	CBYP3454.b1	GE279871
61923827	CBYP3454.g1	GE279872
61923828	CBYP3455.g1	GE279873
61923829	CBYP3456.g1	GE279874
61923830	CBYP3457.b1	GE279875
61923831	CBYP3457.g1	GE279876
61923832	CBYP3460.b1	GE279877
61923833	CBYP3460.g1	GE279878
61923834	CBYP3461.b1	GE279879
61923835	CBYP3463.b1	GE279880
61923836	CBYP3463.g1	GE279881
61923837	CBYP3465.b1	GE279882

61923838	CBYP3465.g1	GE279883
61923839	CBYP3466.b1	GE279884
61923840	CBYP3466.g1	GE279885
61923841	CBYP3467.b1	GE279886
61923842	CBYP3467.g1	GE279887
61923843	CBYP3468.b1	GE279888
61923844	CBYP3468.g1	GE279889
61923845	CBYP3469.b1	GE279890
61923846	CBYP3469.g1	GE279891
61923847	CBYP3470.b1	GE279892
61923848	CBYP3470.g1	GE279893
61923849	CBYP3471.b1	GE279894
61923850	CBYP3471.g1	GE279895
61923851	CBYP3472.b1	GE279896
61923852	CBYP3472.g1	GE279897
61923853	CBYP3473.g1	GE279898
61923854	CBYP3474.b1	GE279899
61923855	CBYP3474.g1	GE279900
61923856	CBYP3475.b1	GE279901
61923857	CBYP3475.g1	GE279902
61923858	CBYP3476.b1	GE279903
61923859	CBYP3476.g1	GE279904
61923860	CBYP3477.b1	GE279905
61923861	CBYP3478.b1	GE279906
61923862	CBYP3478.g1	GE279907
61923863	CBYP3479.b1	GE279908
61923864	CBYP3479.g1	GE279909
61923865	CBYP3480.g1	GE279910
61923866	CBYP3482.b1	GE279911
61923867	CBYP3482.g1	GE279912
61923868	CBYP3483.b1	GE279913
61923869	CBYP3483.g1	GE279914
61923870	CBYP3484.b1	GE279915
61923871	CBYP3484.g1	GE279916
61923872	CBYP3485.b1	GE279917
61923873	CBYP3485.g1	GE279918
61923874	CBYP3486.b1	GE279919
61923875	CBYP3486.g1	GE279920
61923876	CBYP3487.b1	GE279921
61923877	CBYP3487.g1	GE279922
61923878	CBYP3488.b1	GE279923
61923879	CBYP3488.g1	GE279924
61923880	CBYP3489.b1	GE279925
61923881	CBYP3489.g1	GE279926
61923882	CBYP3490.b1	GE279927
61923883	CBYP3490.g1	GE279928
61923884	CBYP3491.b1	GE279929
61923885	CBYP3491.g1	GE279930
61923886	CBYP3492.b1	GE279931
61923887	CBYP3492.g1	GE279932
61923888	CBYP3493.b1	GE279933
61923889	CBYP3494.b1	GE279934
61923890	CBYP3494.g1	GE279935
61923891	CBYP3495.b1	GE279936
61923892	CBYP3495.g1	GE279937
61923893	CBYP3496.b1	GE279938
61923894	CBYP3496.g1	GE279939
61923895	CBYP3497.b1	GE279940
61923896	CBYP3497.g1	GE279941
61923897	CBYP3498.b1	GE279942
61923898	CBYP3498.g1	GE279943
61923899	CBYP3499.b1	GE279944
61923900	CBYP3499.g1	GE279945

61923901	CBYP3500.b1	GE279946
61923902	CBYP3500.g1	GE279947
61923903	CBYP3501.b1	GE279948
61923904	CBYP3501.g1	GE279949
61923905	CBYP3502.b1	GE279950
61923906	CBYP3502.g1	GE279951
61923907	CBYP3503.b1	GE279952
61923908	CBYP3503.g1	GE279953
61923909	CBYP3504.b1	GE279954
61923910	CBYP3504.g1	GE279955
61923911	CBYP3505.b1	GE279956
61923912	CBYP3505.g1	GE279957
61923913	CBYP3506.b1	GE279958
61923914	CBYP3506.g1	GE279959
61923915	CBYP3507.b1	GE279960
61923916	CBYP3507.g1	GE279961
61923917	CBYP3508.b1	GE279962
61923918	CBYP3508.g1	GE279963
61923919	CBYP3509.b1	GE279964
61923920	CBYP3509.g1	GE279965
61923921	CBYP3510.b1	GE279966
61923922	CBYP3510.g1	GE279967
61923923	CBYP3511.b1	GE279968
61923924	CBYP3511.g1	GE279969
61923925	CBYP3513.b1	GE279970
61923926	CBYP3513.g1	GE279971
61923927	CBYP3515.b1	GE279972
61923928	CBYP3515.g1	GE279973
61923929	CBYP3516.b1	GE279974
61923930	CBYP3516.g1	GE279975
61923931	CBYP3518.b1	GE279976
61923932	CBYP3518.g1	GE279977
61923933	CBYP3519.b1	GE279978
61923934	CBYP3519.g1	GE279979
61923935	CBYP3520.b1	GE279980
61923936	CBYP3520.g1	GE279981
61923937	CBYP3521.b1	GE279982
61923938	CBYP3521.g1	GE279983
61923939	CBYP3522.b1	GE279984
61923940	CBYP3523.b1	GE279985
61923941	CBYP3523.g1	GE279986
61923942	CBYP3524.b1	GE279987
61923943	CBYP3524.g1	GE279988
61923944	CBYP3526.b1	GE279989
61923945	CBYP3527.b1	GE279990
61923946	CBYP3527.g1	GE279991
61923947	CBYP3528.b1	GE279992
61923948	CBYP3528.g1	GE279993
61923949	CBYP3529.b1	GE279994
61923950	CBYP3529.g1	GE279995
61923951	CBYP3530.b1	GE279996
61923952	CBYP3531.b1	GE279997
61923953	CBYP3531.g1	GE279998
61923954	CBYP3532.b1	GE279999
61923955	CBYP3532.g1	GE280000
61923956	CBYP3533.b1	GE280001
61923957	CBYP3533.g1	GE280002
61923958	CBYP3534.b1	GE280003
61923959	CBYP3534.g1	GE280004
61923960	CBYP3535.b1	GE280005
61923961	CBYP3535.g1	GE280006
61923962	CBYP3536.b1	GE280007
61923963	CBYP3536.g1	GE280008

61923964	CBYP3537.b1	GE280009
61923965	CBYP3537.g1	GE280010
61923966	CBYP3538.b1	GE280011
61923967	CBYP3538.g1	GE280012
61923968	CBYP3539.b1	GE280013
61923969	CBYP3539.g1	GE280014
61923970	CBYP3540.b1	GE280015
61923971	CBYP3540.g1	GE280016
61923972	CBYP3541.b1	GE280017
61923973	CBYP3541.g1	GE280018
61923974	CBYP3542.b1	GE280019
61923975	CBYP3544.b1	GE280020
61923976	CBYP3544.g1	GE280021
61923977	CBYP3546.b1	GE280022
61923978	CBYP3547.b1	GE280023
61923979	CBYP3547.g1	GE280024
61923980	CBYP3548.b1	GE280025
61923981	CBYP3548.g1	GE280026
61923982	CBYP3549.b1	GE280027
61923983	CBYP3549.g1	GE280028
61923984	CBYP3550.b1	GE280029
61923985	CBYP3550.g1	GE280030
61923986	CBYP3551.b1	GE280031
61923987	CBYP3551.g1	GE280032
61923988	CBYP3552.g1	GE280033
61923989	CBYP3553.b1	GE280034
61923990	CBYP3554.b1	GE280035
61923991	CBYP3554.g1	GE280036
61923992	CBYP3555.b1	GE280037
61923993	CBYP3555.g1	GE280038
61923994	CBYP3556.b1	GE280039
61923995	CBYP3556.g1	GE280040
61923996	CBYP3557.b1	GE280041
61923997	CBYP3557.g1	GE280042
61923998	CBYP3558.b1	GE280043
61923999	CBYP3558.g1	GE280044
61924000	CBYP3559.b1	GE280045
61924001	CBYP3559.g1	GE280046
61924002	CBYP3561.b1	GE280047
61924003	CBYP3561.g1	GE280048
61924004	CBYP3562.b1	GE280049
61924005	CBYP3562.g1	GE280050
61924006	CBYP3563.b1	GE280051
61924007	CBYP3563.g1	GE280052
61924008	CBYP3564.b1	GE280053
61924009	CBYP3564.g1	GE280054
61924010	CBYP3565.b1	GE280055
61924011	CBYP3565.g1	GE280056
61924012	CBYP3566.b1	GE280057
61924013	CBYP3566.g1	GE280058
61924014	CBYP3569.b1	GE280059
61924015	CBYP3569.g1	GE280060
61924016	CBYP3571.b1	GE280061
61924017	CBYP3572.g1	GE280062
61924018	CBYP3573.g1	GE280063
61924019	CBYP3574.b1	GE280064
61924020	CBYP3574.g1	GE280065
61924021	CBYP3576.b1	GE280066
61924022	CBYP3576.g1	GE280067
61924023	CBYP3577.b1	GE280068
61924024	CBYP3578.b1	GE280069
61924025	CBYP3578.g1	GE280070
61924026	CBYP3579.b1	GE280071

61924027	CBYP3579.g1	GE280072
61924028	CBYP3580.b1	GE280073
61924029	CBYP3580.g1	GE280074
61924030	CBYP3581.b1	GE280075
61924031	CBYP3582.b1	GE280076
61924032	CBYP3582.g1	GE280077
61924033	CBYP3583.b1	GE280078
61924034	CBYP3584.b1	GE280079
61924035	CBYP3584.g1	GE280080
61924036	CBYP3585.b1	GE280081
61924037	CBYP3585.g1	GE280082
61924038	CBYP3586.b1	GE280083
61924039	CBYP3586.g1	GE280084
61924040	CBYP3587.b1	GE280085
61924041	CBYP3588.b1	GE280086
61924042	CBYP3588.g1	GE280087
61924043	CBYP3589.b1	GE280088
61924044	CBYP3589.g1	GE280089
61924045	CBYP3590.b1	GE280090
61924046	CBYP3590.g1	GE280091
61924047	CBYP3591.b1	GE280092
61924048	CBYP3591.g1	GE280093
61924049	CBYP3592.b1	GE280094
61924050	CBYP3592.g1	GE280095
61924051	CBYP3593.b1	GE280096
61924052	CBYP3594.b1	GE280097
61924053	CBYP3594.g1	GE280098
61924054	CBYP3595.b1	GE280099
61924055	CBYP3595.g1	GE280100
61924056	CBYP3596.b1	GE280101
61924057	CBYP3596.g1	GE280102
61924058	CBYP3597.b1	GE280103
61924059	CBYP3597.g1	GE280104
61924060	CBYP3598.b1	GE280105
61924061	CBYP3598.g1	GE280106
61924062	CBYP3599.b1	GE280107
61924063	CBYP3599.g1	GE280108
61924064	CBYP3600.b1	GE280109
61924065	CBYP3600.g1	GE280110
61924066	CBYP3601.g1	GE280111
61924067	CBYP3602.b1	GE280112
61924068	CBYP3602.g1	GE280113
61924069	CBYP3603.b1	GE280114
61924070	CBYP3603.g1	GE280115
61924071	CBYP3604.b1	GE280116
61924072	CBYP3604.g1	GE280117
61924073	CBYP3605.b1	GE280118
61924074	CBYP3605.g1	GE280119
61924075	CBYP3606.b1	GE280120
61924076	CBYP3607.b1	GE280121
61924077	CBYP3607.g1	GE280122
61924078	CBYP3608.b1	GE280123
61924079	CBYP3608.g1	GE280124
61924080	CBYP3609.b1	GE280125
61924081	CBYP3609.g1	GE280126
61924082	CBYP3610.b1	GE280127
61924083	CBYP3611.b1	GE280128
61924084	CBYP3611.g1	GE280129
61924085	CBYP3612.b1	GE280130
61924086	CBYP3612.g1	GE280131
61924087	CBYP3613.b1	GE280132
61924088	CBYP3613.g1	GE280133
61924089	CBYP3614.b1	GE280134

61924090	CBYP3615.b1	GE280135
61924091	CBYP3615.g1	GE280136
61924092	CBYP3616.b1	GE280137
61924093	CBYP3616.g1	GE280138
61924094	CBYP3617.b1	GE280139
61924095	CBYP3617.g1	GE280140
61924096	CBYP3618.b1	GE280141
61924097	CBYP3618.g1	GE280142
61924098	CBYP3619.b1	GE280143
61924099	CBYP3619.g1	GE280144
61924100	CBYP3620.g1	GE280145
61924101	CBYP3621.b1	GE280146
61924102	CBYP3622.b1	GE280147
61924103	CBYP3622.g1	GE280148
61924104	CBYP3623.b1	GE280149
61924105	CBYP3625.b1	GE280150
61924106	CBYP3625.g1	GE280151
61924107	CBYP3626.b1	GE280152
61924108	CBYP3626.g1	GE280153
61924109	CBYP3627.b1	GE280154
61924110	CBYP3627.g1	GE280155
61924111	CBYP3628.b1	GE280156
61924112	CBYP3628.g1	GE280157
61924113	CBYP3629.b1	GE280158
61924114	CBYP3629.g1	GE280159
61924115	CBYP3630.b1	GE280160
61924116	CBYP3630.g1	GE280161
61924117	CBYP3631.b1	GE280162
61924118	CBYP3631.g1	GE280163
61924119	CBYP3632.b1	GE280164
61924120	CBYP3632.g1	GE280165
61924121	CBYP3633.b1	GE280166
61924122	CBYP3633.g1	GE280167
61924123	CBYP3634.b1	GE280168
61924124	CBYP3634.g1	GE280169
61924125	CBYP3635.b1	GE280170
61924126	CBYP3636.b1	GE280171
61924127	CBYP3636.g1	GE280172
61924128	CBYP3637.b1	GE280173
61924129	CBYP3637.g1	GE280174
61924130	CBYP3639.b1	GE280175
61924131	CBYP3639.g1	GE280176
61924132	CBYP3640.b1	GE280177
61924133	CBYP3640.g1	GE280178
61924134	CBYP3641.b1	GE280179
61924135	CBYP3641.g1	GE280180
61924136	CBYP3642.b1	GE280181
61924137	CBYP3642.g1	GE280182
61924138	CBYP3643.b1	GE280183
61924139	CBYP3643.g1	GE280184
61924140	CBYP3644.b1	GE280185
61924141	CBYP3644.g1	GE280186
61924142	CBYP3645.b1	GE280187
61924143	CBYP3645.g1	GE280188
61924144	CBYP3646.b1	GE280189
61924145	CBYP3646.g1	GE280190
61924146	CBYP3647.b1	GE280191
61924147	CBYP3647.g1	GE280192
61924148	CBYP3648.b1	GE280193
61924149	CBYP3648.g1	GE280194
61924150	CBYP3649.b1	GE280195
61924151	CBYP3649.g1	GE280196
61924152	CBYP3650.b1	GE280197

61924153	CBYP3650.g1	GE280198
61924154	CBYP3651.b1	GE280199
61924155	CBYP3652.b1	GE280200
61924156	CBYP3652.g1	GE280201
61924157	CBYP3655.b1	GE280202
61924158	CBYP3655.g1	GE280203
61924159	CBYP3656.b1	GE280204
61924160	CBYP3656.g1	GE280205
61924161	CBYP3657.b1	GE280206
61924162	CBYP3657.g1	GE280207
61924163	CBYP3658.b1	GE280208
61924164	CBYP3658.g1	GE280209
61924165	CBYP3659.b1	GE280210
61924166	CBYP3659.g1	GE280211
61924167	CBYP3660.b1	GE280212
61924168	CBYP3660.g1	GE280213
61924169	CBYP3661.b1	GE280214
61924170	CBYP3661.g1	GE280215
61924171	CBYP3662.b1	GE280216
61924172	CBYP3662.g1	GE280217
61924173	CBYP3663.b1	GE280218
61924174	CBYP3663.g1	GE280219
61924175	CBYP3664.b1	GE280220
61924176	CBYP3664.g1	GE280221
61924177	CBYP3665.b1	GE280222
61924178	CBYP3665.g1	GE280223
61924179	CBYP3666.b1	GE280224
61924180	CBYP3666.g1	GE280225
61924181	CBYP3667.b1	GE280226
61924182	CBYP3667.g1	GE280227
61924183	CBYP3668.b1	GE280228
61924184	CBYP3668.g1	GE280229
61924185	CBYP3670.b1	GE280230
61924186	CBYP3670.g1	GE280231
61924187	CBYP3671.b1	GE280232
61924188	CBYP3671.g1	GE280233
61924189	CBYP3672.b1	GE280234
61924190	CBYP3672.g1	GE280235
61924191	CBYP3673.b1	GE280236
61924192	CBYP3673.g1	GE280237
61924193	CBYP3677.b1	GE280238
61924194	CBYP3677.g1	GE280239
61924195	CBYP3678.b1	GE280240
61924196	CBYP3678.g1	GE280241
61924197	CBYP3679.b1	GE280242
61924198	CBYP3679.g1	GE280243
61924199	CBYP3680.b1	GE280244
61924200	CBYP3680.g1	GE280245
61924201	CBYP3681.g1	GE280246
61924202	CBYP3682.b1	GE280247
61924203	CBYP3682.g1	GE280248
61924204	CBYP3684.b1	GE280249
61924205	CBYP3684.g1	GE280250
61924206	CBYP3686.b1	GE280251
61924207	CBYP3686.g1	GE280252
61924208	CBYP3687.b1	GE280253
61924209	CBYP3687.g1	GE280254
61924210	CBYP3688.b1	GE280255
61924211	CBYP3689.b1	GE280256
61924212	CBYP3689.g1	GE280257
61924213	CBYP3690.b1	GE280258
61924214	CBYP3690.g1	GE280259
61924215	CBYP3691.b1	GE280260

61924216	CBYP3691.g1	GE280261
61924217	CBYP3692.b1	GE280262
61924218	CBYP3692.g1	GE280263
61924219	CBYP3693.b1	GE280264
61924220	CBYP3693.g1	GE280265
61924221	CBYP3694.b1	GE280266
61924222	CBYP3694.g1	GE280267
61924223	CBYP3695.b1	GE280268
61924224	CBYP3695.g1	GE280269
61924225	CBYP3696.b1	GE280270
61924226	CBYP3696.g1	GE280271
61924227	CBYP3698.b1	GE280272
61924228	CBYP3698.g1	GE280273
61924229	CBYP3699.b1	GE280274
61924230	CBYP3699.g1	GE280275
61924231	CBYP3700.b1	GE280276
61924232	CBYP3700.g1	GE280277
61924233	CBYP3701.b1	GE280278
61924234	CBYP3701.g1	GE280279
61924235	CBYP3702.b1	GE280280
61924236	CBYP3702.g1	GE280281
61924237	CBYP3703.b1	GE280282
61924238	CBYP3703.g1	GE280283
61924239	CBYP3704.b1	GE280284
61924240	CBYP3704.g1	GE280285
61924241	CBYP3705.b1	GE280286
61924242	CBYP3706.b1	GE280287
61924243	CBYP3706.g1	GE280288
61924244	CBYP3707.b1	GE280289
61924245	CBYP3707.g1	GE280290
61924246	CBYP3708.b1	GE280291
61924247	CBYP3708.g1	GE280292
61924248	CBYP3709.b1	GE280293
61924249	CBYP3709.g1	GE280294
61924250	CBYP3710.g1	GE280295
61924251	CBYP3711.b1	GE280296
61924252	CBYP3711.g1	GE280297
61924253	CBYP3712.b1	GE280298
61924254	CBYP3712.g1	GE280299
61924255	CBYP3713.b1	GE280300
61924256	CBYP3713.g1	GE280301
61924257	CBYP3714.b1	GE280302
61924258	CBYP3714.g1	GE280303
61924259	CBYP3715.b1	GE280304
61924260	CBYP3716.b1	GE280305
61924261	CBYP3716.g1	GE280306
61924262	CBYP3717.b1	GE280307
61924263	CBYP3717.g1	GE280308
61924264	CBYP3718.b1	GE280309
61924265	CBYP3718.g1	GE280310
61924266	CBYP3719.b1	GE280311
61924267	CBYP3719.g1	GE280312
61924268	CBYP3720.b1	GE280313
61924269	CBYP3720.g1	GE280314
61924270	CBYP3721.b1	GE280315
61924271	CBYP3721.g1	GE280316
61924272	CBYP3722.b1	GE280317
61924273	CBYP3722.g1	GE280318
61924274	CBYP3723.b1	GE280319
61924275	CBYP3723.g1	GE280320
61924276	CBYP3724.b1	GE280321
61924277	CBYP3724.g1	GE280322
61924278	CBYP3725.b1	GE280323

61924279	CBYP3725.g1	GE280324
61924280	CBYP3727.b1	GE280325
61924281	CBYP3727.g1	GE280326
61924282	CBYP3728.b1	GE280327
61924283	CBYP3728.g1	GE280328
61924284	CBYP3729.b1	GE280329
61924285	CBYP3729.g1	GE280330
61924286	CBYP3730.b1	GE280331
61924287	CBYP3730.g1	GE280332
61924288	CBYP3731.b1	GE280333
61924289	CBYP3731.g1	GE280334
61924290	CBYP3732.b1	GE280335
61924291	CBYP3732.g1	GE280336
61924292	CBYP3733.b1	GE280337
61924293	CBYP3733.g1	GE280338
61924294	CBYP3734.b1	GE280339
61924295	CBYP3734.g1	GE280340
61924296	CBYP3735.b1	GE280341
61924297	CBYP3735.g1	GE280342
61924298	CBYP3736.b1	GE280343
61924299	CBYP3736.g1	GE280344
61924300	CBYP3737.b1	GE280345
61924301	CBYP3737.g1	GE280346
61924302	CBYP3738.b1	GE280347
61924303	CBYP3738.g1	GE280348
61924304	CBYP3739.b1	GE280349
61924305	CBYP3739.g1	GE280350
61924306	CBYP3740.b1	GE280351
61924307	CBYP3740.g1	GE280352
61924308	CBYP3741.b1	GE280353
61924309	CBYP3741.g1	GE280354
61924310	CBYP3742.b1	GE280355
61924311	CBYP3742.g1	GE280356
61924312	CBYP3744.b1	GE280357
61924313	CBYP3745.b1	GE280358
61924314	CBYP3745.g1	GE280359
61924315	CBYP3746.b1	GE280360
61924316	CBYP3746.g1	GE280361
61924317	CBYP3747.b1	GE280362
61924318	CBYP3747.g1	GE280363
61924319	CBYP3748.b1	GE280364
61924320	CBYP3748.g1	GE280365
61924321	CBYP3749.b1	GE280366
61924322	CBYP3750.b1	GE280367
61924323	CBYP3750.g1	GE280368
61924324	CBYP3751.b1	GE280369
61924325	CBYP3751.g1	GE280370
61924326	CBYP3754.b1	GE280371
61924327	CBYP3754.g1	GE280372
61924328	CBYP3755.b1	GE280373
61924329	CBYP3756.b1	GE280374
61924330	CBYP3756.g1	GE280375
61924331	CBYP3757.b1	GE280376
61924332	CBYP3757.g1	GE280377
61924333	CBYP3758.b1	GE280378
61924334	CBYP3758.g1	GE280379
61924335	CBYP3760.b1	GE280380
61924336	CBYP3760.g1	GE280381
61924337	CBYP3761.g1	GE280382
61924338	CBYP3762.b1	GE280383
61924339	CBYP3762.g1	GE280384
61924340	CBYP3763.b1	GE280385
61924341	CBYP3763.g1	GE280386

61924342	CBYP3764.b1	GE280387
61924343	CBYP3764.g1	GE280388
61924344	CBYP3765.b1	GE280389
61924345	CBYP3765.g1	GE280390
61924346	CBYP3766.b1	GE280391
61924347	CBYP3766.g1	GE280392
61924348	CBYP3767.b1	GE280393
61924349	CBYP3767.g1	GE280394
61924350	CBYP3769.b1	GE280395
61924351	CBYP3769.g1	GE280396
61924352	CBYP3770.b1	GE280397
61924353	CBYP3770.g1	GE280398
61924354	CBYP3771.b1	GE280399
61924355	CBYP3771.g1	GE280400
61924356	CBYP3772.b1	GE280401
61924357	CBYP3772.g1	GE280402
61924358	CBYP3773.b1	GE280403
61924359	CBYP3773.g1	GE280404
61924360	CBYP3774.b1	GE280405
61924361	CBYP3774.g1	GE280406
61924362	CBYP3775.b1	GE280407
61924363	CBYP3775.g1	GE280408
61924364	CBYP3776.g1	GE280409
61924365	CBYP3777.b1	GE280410
61924366	CBYP3777.g1	GE280411
61924367	CBYP3778.b1	GE280412
61924368	CBYP3778.g1	GE280413
61924369	CBYP3779.b1	GE280414
61924370	CBYP3780.b1	GE280415
61924371	CBYP3780.g1	GE280416
61924372	CBYP3781.g1	GE280417
61924373	CBYP3782.b1	GE280418
61924374	CBYP3782.g1	GE280419
61924375	CBYP3784.b1	GE280420
61924376	CBYP3784.g1	GE280421
61924377	CBYP3785.b1	GE280422
61924378	CBYP3786.b1	GE280423
61924379	CBYP3786.g1	GE280424
61924380	CBYP3787.b1	GE280425
61924381	CBYP3787.g1	GE280426
61924382	CBYP3788.b1	GE280427
61924383	CBYP3788.g1	GE280428
61924384	CBYP3789.b1	GE280429
61924385	CBYP3789.g1	GE280430
61924386	CBYP3790.b1	GE280431
61924387	CBYP3790.g1	GE280432
61924388	CBYP3791.b1	GE280433
61924389	CBYP3791.g1	GE280434
61924390	CBYP3792.b1	GE280435
61924391	CBYP3792.g1	GE280436
61924392	CBYP3793.b1	GE280437
61924393	CBYP3793.g1	GE280438
61924394	CBYP3794.b1	GE280439
61924395	CBYP3794.g1	GE280440
61924396	CBYP3795.b1	GE280441
61924397	CBYP3795.g1	GE280442
61924398	CBYP3796.b1	GE280443
61924399	CBYP3796.g1	GE280444
61924400	CBYP3797.b1	GE280445
61924401	CBYP3798.b1	GE280446
61924402	CBYP3798.g1	GE280447
61924403	CBYP3799.b1	GE280448
61924404	CBYP3799.g1	GE280449

61924405	CBYP3800.g1	GE280450
61924406	CBYP3801.g1	GE280451
61924407	CBYP3802.b1	GE280452
61924408	CBYP3802.g1	GE280453
61924409	CBYP3803.b1	GE280454
61924410	CBYP3803.g1	GE280455
61924411	CBYP3804.b1	GE280456
61924412	CBYP3804.g1	GE280457
61924413	CBYP3805.b1	GE280458
61924414	CBYP3805.g1	GE280459
61924415	CBYP3806.b1	GE280460
61924416	CBYP3806.g1	GE280461
61924417	CBYP3808.b1	GE280462
61924418	CBYP3808.g1	GE280463
61924419	CBYP3810.b1	GE280464
61924420	CBYP3810.g1	GE280465
61924421	CBYP3811.b1	GE280466
61924422	CBYP3812.b1	GE280467
61924423	CBYP3812.g1	GE280468
61924424	CBYP3813.b1	GE280469
61924425	CBYP3813.g1	GE280470
61924426	CBYP3814.b1	GE280471
61924427	CBYP3814.g1	GE280472
61924428	CBYP3815.b1	GE280473
61924429	CBYP3816.b1	GE280474
61924430	CBYP3816.g1	GE280475
61924431	CBYP3817.b1	GE280476
61924432	CBYP3817.g1	GE280477
61924433	CBYP3818.b1	GE280478
61924434	CBYP3818.g1	GE280479
61924435	CBYP3819.b1	GE280480
61924436	CBYP3819.g1	GE280481
61924437	CBYP3820.b1	GE280482
61924438	CBYP3820.g1	GE280483
61924439	CBYP3821.b1	GE280484
61924440	CBYP3821.g1	GE280485
61924441	CBYP3822.b1	GE280486
61924442	CBYP3822.g1	GE280487
61924443	CBYP3823.b1	GE280488
61924444	CBYP3823.g1	GE280489
61924445	CBYP3825.b1	GE280490
61924446	CBYP3825.g1	GE280491
61924447	CBYP3826.b1	GE280492
61924448	CBYP3826.g1	GE280493
61924449	CBYP3828.b1	GE280494
61924450	CBYP3828.g1	GE280495
61924451	CBYP3830.b1	GE280496
61924452	CBYP3830.g1	GE280497
61924453	CBYP3831.b1	GE280498
61924454	CBYP3831.g1	GE280499
61924455	CBYP3832.b1	GE280500
61924456	CBYP3832.g1	GE280501
61924457	CBYP3833.b1	GE280502
61924458	CBYP3833.g1	GE280503
61924459	CBYP3834.b1	GE280504
61924460	CBYP3834.g1	GE280505
61924461	CBYP3836.b1	GE280506
61924462	CBYP3836.g1	GE280507
61924463	CBYP3837.b1	GE280508
61924464	CBYP3837.g1	GE280509
61924465	CBYP3838.b1	GE280510
61924466	CBYP3838.g1	GE280511
61924467	CBYP3841.b1	GE280512

61924468	CBYP3841.g1	GE280513
61924469	CBYP3843.b1	GE280514
61924470	CBYP3843.g1	GE280515
61924471	CBYP3845.b1	GE280516
61924472	CBYP3845.g1	GE280517
61924473	CBYP3846.b1	GE280518
61924474	CBYP3846.g1	GE280519
61924475	CBYP3847.b1	GE280520
61924476	CBYP3847.g1	GE280521
61924477	CBYP3849.b1	GE280522
61924478	CBYP3849.g1	GE280523
61924479	CBYP3850.b1	GE280524
61924480	CBYP3850.g1	GE280525
61924481	CBYP3851.b1	GE280526
61924482	CBYP3851.g1	GE280527
61924483	CBYP3852.b1	GE280528
61924484	CBYP3852.g1	GE280529
61924485	CBYP3853.b1	GE280530
61924486	CBYP3853.g1	GE280531
61924487	CBYP3854.b1	GE280532
61924488	CBYP3854.g1	GE280533
61924489	CBYP3855.b1	GE280534
61924490	CBYP3855.g1	GE280535
61924491	CBYP3856.b1	GE280536
61924492	CBYP3856.g1	GE280537
61924493	CBYP3857.b1	GE280538
61924494	CBYP3858.b1	GE280539
61924495	CBYP3859.b1	GE280540
61924496	CBYP3859.g1	GE280541
61924497	CBYP3860.b1	GE280542
61924498	CBYP3860.g1	GE280543
61924499	CBYP3861.b1	GE280544
61924500	CBYP3861.g1	GE280545
61924501	CBYP3862.b1	GE280546
61924502	CBYP3862.g1	GE280547
61924503	CBYP3863.b1	GE280548
61924504	CBYP3863.g1	GE280549
61924505	CBYP3864.b1	GE280550
61924506	CBYP3864.g1	GE280551
61924507	CBYP3865.b1	GE280552
61924508	CBYP3865.g1	GE280553
61924509	CBYP3866.b1	GE280554
61924510	CBYP3866.g1	GE280555
61924511	CBYP3867.b1	GE280556
61924512	CBYP3867.g1	GE280557
61924513	CBYP3868.b1	GE280558
61924514	CBYP3868.g1	GE280559
61924515	CBYP3869.b1	GE280560
61924516	CBYP3870.b1	GE280561
61924517	CBYP3870.g1	GE280562
61924518	CBYP3871.b1	GE280563
61924519	CBYP3871.g1	GE280564
61924520	CBYP3872.b1	GE280565
61924521	CBYP3873.b1	GE280566
61924522	CBYP3873.g1	GE280567
61924523	CBYP3874.b1	GE280568
61924524	CBYP3874.g1	GE280569
61924525	CBYP3875.b1	GE280570
61924526	CBYP3875.g1	GE280571
61924527	CBYP3876.b1	GE280572
61924528	CBYP3876.g1	GE280573
61924529	CBYP3877.b1	GE280574
61924530	CBYP3877.g1	GE280575

61924531	CBYP3879.b1	GE280576
61924532	CBYP3879.g1	GE280577
61924533	CBYP3880.b1	GE280578
61924534	CBYP3881.b1	GE280579
61924535	CBYP3881.g1	GE280580
61924536	CBYP3882.b1	GE280581
61924537	CBYP3882.g1	GE280582
61924538	CBYP3883.b1	GE280583
61924539	CBYP3883.g1	GE280584
61924540	CBYP3884.b1	GE280585
61924541	CBYP3884.g1	GE280586
61924542	CBYP3885.b1	GE280587
61924543	CBYP3885.g1	GE280588
61924544	CBYP3886.b1	GE280589
61924545	CBYP3886.g1	GE280590
61924546	CBYP3887.b1	GE280591
61924547	CBYP3887.g1	GE280592
61924548	CBYP3888.b1	GE280593
61924549	CBYP3888.g1	GE280594
61924550	CBYP3889.b1	GE280595
61924551	CBYP3889.g1	GE280596
61924552	CBYP3890.b1	GE280597
61924553	CBYP3890.g1	GE280598
61924554	CBYP3891.b1	GE280599
61924555	CBYP3891.g1	GE280600
61924556	CBYP3892.b1	GE280601
61924557	CBYP3892.g1	GE280602
61924558	CBYP3893.b1	GE280603
61924559	CBYP3893.g1	GE280604
61924560	CBYP3894.b1	GE280605
61924561	CBYP3894.g1	GE280606
61924562	CBYP3895.b1	GE280607
61924563	CBYP3895.g1	GE280608
61924564	CBYP3896.b1	GE280609
61924565	CBYP3896.g1	GE280610
61924566	CBYP3897.b1	GE280611
61924567	CBYP3897.g1	GE280612
61924568	CBYP3898.b1	GE280613
61924569	CBYP3899.b1	GE280614
61924570	CBYP3899.g1	GE280615
61924571	CBYP3900.g1	GE280616
61924572	CBYP3901.b1	GE280617
61924573	CBYP3901.g1	GE280618
61924574	CBYP3902.b1	GE280619
61924575	CBYP3902.g1	GE280620
61924576	CBYP3903.b1	GE280621
61924577	CBYP3903.g1	GE280622
61924578	CBYP3904.b1	GE280623
61924579	CBYP3904.g1	GE280624
61924580	CBYP3905.b1	GE280625
61924581	CBYP3906.b1	GE280626
61924582	CBYP3906.g1	GE280627
61924583	CBYP3907.b1	GE280628
61924584	CBYP3907.g1	GE280629
61924585	CBYP3908.b1	GE280630
61924586	CBYP3908.g1	GE280631
61924587	CBYP3911.b1	GE280632
61924588	CBYP3911.g1	GE280633
61924589	CBYP3912.b1	GE280634
61924590	CBYP3912.g1	GE280635
61924591	CBYP3913.g1	GE280636
61924592	CBYP3914.b1	GE280637
61924593	CBYP3914.g1	GE280638

61924594	CBYP3916.b1	GE280639
61924595	CBYP3917.b1	GE280640
61924596	CBYP3917.g1	GE280641
61924597	CBYP3918.b1	GE280642
61924598	CBYP3918.g1	GE280643
61924599	CBYP3919.b1	GE280644
61924600	CBYP3919.g1	GE280645
61924601	CBYP3920.b1	GE280646
61924602	CBYP3920.g1	GE280647
61924603	CBYP3922.b1	GE280648
61924604	CBYP3922.g1	GE280649
61924605	CBYP3923.b1	GE280650
61924606	CBYP3923.g1	GE280651
61924607	CBYP3925.b1	GE280652
61924608	CBYP3925.g1	GE280653
61924609	CBYP3926.b1	GE280654
61924610	CBYP3926.g1	GE280655
61924611	CBYP3927.b1	GE280656
61924612	CBYP3927.g1	GE280657
61924613	CBYP3928.b1	GE280658
61924614	CBYP3928.g1	GE280659
61924615	CBYP3929.b1	GE280660
61924616	CBYP3930.b1	GE280661
61924617	CBYP3930.g1	GE280662
61924618	CBYP3931.b1	GE280663
61924619	CBYP3932.b1	GE280664
61924620	CBYP3932.g1	GE280665
61924621	CBYP3933.b1	GE280666
61924622	CBYP3933.g1	GE280667
61924623	CBYP3934.b1	GE280668
61924624	CBYP3934.g1	GE280669
61924625	CBYP3935.b1	GE280670
61924626	CBYP3935.g1	GE280671
61924627	CBYP3936.b1	GE280672
61924628	CBYP3936.g1	GE280673
61924629	CBYP3937.b1	GE280674
61924630	CBYP3937.g1	GE280675
61924631	CBYP3938.b1	GE280676
61924632	CBYP3938.g1	GE280677
61924633	CBYP3940.b1	GE280678
61924634	CBYP3940.g1	GE280679
61924635	CBYP3941.b1	GE280680
61924636	CBYP3943.b1	GE280681
61924637	CBYP3943.g1	GE280682
61924638	CBYP3944.b1	GE280683
61924639	CBYP3944.g1	GE280684
61924640	CBYP3945.b1	GE280685
61924641	CBYP3945.g1	GE280686
61924642	CBYP3946.b1	GE280687
61924643	CBYP3946.g1	GE280688
61924644	CBYP3947.b1	GE280689
61924645	CBYP3947.g1	GE280690
61924646	CBYP3948.b1	GE280691
61924647	CBYP3948.g1	GE280692
61924648	CBYP3949.b1	GE280693
61924649	CBYP3949.g1	GE280694
61924650	CBYP3950.b1	GE280695
61924651	CBYP3950.g1	GE280696
61924652	CBYP3951.g1	GE280697
61924653	CBYP3952.b1	GE280698
61924654	CBYP3952.g1	GE280699
61924655	CBYP3953.b1	GE280700
61924656	CBYP3953.g1	GE280701

61924657	CBYP3954.b1	GE280702
61924658	CBYP3954.g1	GE280703
61924659	CBYP3955.b1	GE280704
61924660	CBYP3955.g1	GE280705
61924661	CBYP3956.b1	GE280706
61924662	CBYP3956.g1	GE280707
61924663	CBYP3957.b1	GE280708
61924664	CBYP3958.b1	GE280709
61924665	CBYP3958.g1	GE280710
61924666	CBYP3959.b1	GE280711
61924667	CBYP3959.g1	GE280712
61924668	CBYP3960.b1	GE280713
61924669	CBYP3960.g1	GE280714
61924670	CBYP3962.b1	GE280715
61924671	CBYP3962.g1	GE280716
61924672	CBYP3963.b1	GE280717
61924673	CBYP3964.b1	GE280718
61924674	CBYP3964.g1	GE280719
61924675	CBYP3965.b1	GE280720
61924676	CBYP3965.g1	GE280721
61924677	CBYP3966.b1	GE280722
61924678	CBYP3966.g1	GE280723
61924679	CBYP3967.b1	GE280724
61924680	CBYP3967.g1	GE280725
61924681	CBYP3968.b1	GE280726
61924682	CBYP3968.g1	GE280727
61924683	CBYP3969.b1	GE280728
61924684	CBYP3969.g1	GE280729
61924685	CBYP3971.b1	GE280730
61924686	CBYP3971.g1	GE280731
61924687	CBYP3973.b1	GE280732
61924688	CBYP3973.g1	GE280733
61924689	CBYP3974.b1	GE280734
61924690	CBYP3974.g1	GE280735
61924691	CBYP3975.b1	GE280736
61924692	CBYP3975.g1	GE280737
61924693	CBYP3976.b1	GE280738
61924694	CBYP3976.g1	GE280739
61924695	CBYP3977.b1	GE280740
61924696	CBYP3977.g1	GE280741
61924697	CBYP3978.g1	GE280742
61924698	CBYP3979.b1	GE280743
61924699	CBYP3979.g1	GE280744
61924700	CBYP3980.b1	GE280745
61924701	CBYP3980.g1	GE280746
61924702	CBYP3981.g1	GE280747
61924703	CBYP3984.b1	GE280748
61924704	CBYP3984.g1	GE280749
61924705	CBYP3985.b1	GE280750
61924706	CBYP3985.g1	GE280751
61924707	CBYP3986.b1	GE280752
61924708	CBYP3986.g1	GE280753
61924709	CBYP3987.b1	GE280754
61924710	CBYP3987.g1	GE280755
61924711	CBYP3988.b1	GE280756
61924712	CBYP3988.g1	GE280757
61924713	CBYP3989.b1	GE280758
61924714	CBYP3989.g1	GE280759
61924715	CBYP3990.b1	GE280760
61924716	CBYP3990.g1	GE280761
61924717	CBYP3991.b1	GE280762
61924718	CBYP3991.g1	GE280763
61924719	CBYP3992.b1	GE280764

61924720	CBYP3992.g1	GE280765
61924721	CBYP3993.b1	GE280766
61924722	CBYP3993.g1	GE280767
61924723	CBYP3994.b1	GE280768
61924724	CBYP3994.g1	GE280769
61924725	CBYP3995.b1	GE280770
61924726	CBYP3995.g1	GE280771
61924727	CBYP3996.b1	GE280772
61924728	CBYP3996.g1	GE280773
61924729	CBYP3997.b1	GE280774
61924730	CBYP3997.g1	GE280775
61924731	CBYP3999.b1	GE280776
61924732	CBYP3999.g1	GE280777
61924733	CBYP4000.b1	GE280778
61924734	CBYP4000.g1	GE280779
61924735	CBYP4002.b1	GE280780
61924736	CBYP4002.g1	GE280781
61924737	CBYP4003.b1	GE280782
61924738	CBYP4003.g1	GE280783
61924739	CBYP4004.b1	GE280784
61924740	CBYP4004.g1	GE280785
61924741	CBYP4005.b1	GE280786
61924742	CBYP4005.g1	GE280787
61924743	CBYP4006.b1	GE280788
61924744	CBYP4006.g1	GE280789
61924745	CBYP4007.b1	GE280790
61924746	CBYP4007.g1	GE280791
61924747	CBYP4008.b1	GE280792
61924748	CBYP4008.g1	GE280793
61924749	CBYP4009.b1	GE280794
61924750	CBYP4009.g1	GE280795
61924751	CBYP4011.b1	GE280796
61924752	CBYP4011.g1	GE280797
61924753	CBYP4012.b1	GE280798
61924754	CBYP4012.g1	GE280799
61924755	CBYP4013.b1	GE280800
61924756	CBYP4013.g1	GE280801
61924757	CBYP4014.b1	GE280802
61924758	CBYP4014.g1	GE280803
61924759	CBYP4015.b1	GE280804
61924760	CBYP4015.g1	GE280805
61924761	CBYP4016.g1	GE280806
61924762	CBYP4017.b1	GE280807
61924763	CBYP4017.g1	GE280808
61924764	CBYP4018.b1	GE280809
61924765	CBYP4018.g1	GE280810
61924766	CBYP4019.b1	GE280811
61924767	CBYP4019.g1	GE280812
61924768	CBYP4020.b1	GE280813
61924769	CBYP4020.g1	GE280814
61924770	CBYP4021.b1	GE280815
61924771	CBYP4021.g1	GE280816
61924772	CBYP4022.b1	GE280817
61924773	CBYP4022.g1	GE280818
61924774	CBYP4023.b1	GE280819
61924775	CBYP4023.g1	GE280820
61924776	CBYP4024.b1	GE280821
61924777	CBYP4024.g1	GE280822
61924778	CBYP4025.b1	GE280823
61924779	CBYP4025.g1	GE280824
61924780	CBYP4026.b1	GE280825
61924781	CBYP4027.b1	GE280826
61924782	CBYP4027.g1	GE280827

61924783	CBYP4028.b1	GE280828
61924784	CBYP4028.g1	GE280829
61924785	CBYP4029.b1	GE280830
61924786	CBYP4029.g1	GE280831
61924787	CBYP4030.b1	GE280832
61924788	CBYP4030.g1	GE280833
61924789	CBYP4031.b1	GE280834
61924790	CBYP4031.g1	GE280835
61924791	CBYP4032.b1	GE280836
61924792	CBYP4032.g1	GE280837
61924793	CBYP4033.b1	GE280838
61924794	CBYP4033.g1	GE280839
61924795	CBYP4034.b1	GE280840
61924796	CBYP4034.g1	GE280841
61924797	CBYP4035.b1	GE280842
61924798	CBYP4035.g1	GE280843
61924799	CBYP4036.b1	GE280844
61924800	CBYP4036.g1	GE280845
61924801	CBYP4037.b1	GE280846
61924802	CBYP4037.g1	GE280847
61924803	CBYP4038.b1	GE280848
61924804	CBYP4038.g1	GE280849
61924805	CBYP4039.b1	GE280850
61924806	CBYP4039.g1	GE280851
61924807	CBYP4040.g1	GE280852
61924808	CBYP4042.b1	GE280853
61924809	CBYP4042.g1	GE280854
61924810	CBYP4043.b1	GE280855
61924811	CBYP4043.g1	GE280856
61924812	CBYP4044.b1	GE280857
61924813	CBYP4044.g1	GE280858
61924814	CBYP4045.b1	GE280859
61924815	CBYP4045.g1	GE280860
61924816	CBYP4046.b1	GE280861
61924817	CBYP4046.g1	GE280862
61924818	CBYP4047.b1	GE280863
61924819	CBYP4047.g1	GE280864
61924820	CBYP4049.b1	GE280865
61924821	CBYP4049.g1	GE280866
61924822	CBYP4050.b1	GE280867
61924823	CBYP4050.g1	GE280868
61924824	CBYP4053.b1	GE280869
61924825	CBYP4053.g1	GE280870
61924826	CBYP4054.g1	GE280871
61924827	CBYP4055.g1	GE280872
61924828	CBYP4056.b1	GE280873
61924829	CBYP4056.g1	GE280874
61924830	CBYP4057.b1	GE280875
61924831	CBYP4057.g1	GE280876
61924832	CBYP4058.b1	GE280877
61924833	CBYP4058.g1	GE280878
61924834	CBYP4059.b1	GE280879
61924835	CBYP4059.g1	GE280880
61924836	CBYP4060.b1	GE280881
61924837	CBYP4060.g1	GE280882
61924838	CBYP4061.b1	GE280883
61924839	CBYP4061.g1	GE280884
61924840	CBYP4062.b1	GE280885
61924841	CBYP4062.g1	GE280886
61924842	CBYP4063.b1	GE280887
61924843	CBYP4063.g1	GE280888
61924844	CBYP4064.g1	GE280889
61924845	CBYP4065.b1	GE280890

61924846	CBYP4065.g1	GE280891
61924847	CBYP4066.b1	GE280892
61924848	CBYP4066.g1	GE280893
61924849	CBYP4067.b1	GE280894
61924850	CBYP4067.g1	GE280895
61924851	CBYP4068.b1	GE280896
61924852	CBYP4068.g1	GE280897
61924853	CBYP4069.b1	GE280898
61924854	CBYP4069.g1	GE280899
61924855	CBYP4071.b1	GE280900
61924856	CBYP4071.g1	GE280901
61924857	CBYP4072.b1	GE280902
61924858	CBYP4072.g1	GE280903
61924859	CBYP4073.b1	GE280904
61924860	CBYP4073.g1	GE280905
61924861	CBYP4074.b1	GE280906
61924862	CBYP4074.g1	GE280907
61924863	CBYP4075.b1	GE280908
61924864	CBYP4075.g1	GE280909
61924865	CBYP4076.b1	GE280910
61924866	CBYP4076.g1	GE280911
61924867	CBYP4077.b1	GE280912
61924868	CBYP4077.g1	GE280913
61924869	CBYP4078.b1	GE280914
61924870	CBYP4078.g1	GE280915
61924871	CBYP4079.b1	GE280916
61924872	CBYP4079.g1	GE280917
61924873	CBYP4080.b1	GE280918
61924874	CBYP4080.g1	GE280919
61924875	CBYP4081.b1	GE280920
61924876	CBYP4081.g1	GE280921
61924877	CBYP4082.b1	GE280922
61924878	CBYP4082.g1	GE280923
61924879	CBYP4083.b1	GE280924
61924880	CBYP4083.g1	GE280925
61924881	CBYP4084.b1	GE280926
61924882	CBYP4085.b1	GE280927
61924883	CBYP4085.g1	GE280928
61924884	CBYP4086.b1	GE280929
61924885	CBYP4086.g1	GE280930
61924886	CBYP4087.b1	GE280931
61924887	CBYP4087.g1	GE280932
61924888	CBYP4088.b1	GE280933
61924889	CBYP4089.g1	GE280934
61924890	CBYP4090.b1	GE280935
61924891	CBYP4090.g1	GE280936
61924892	CBYP4091.b1	GE280937
61924893	CBYP4091.g1	GE280938
61924894	CBYP4092.b1	GE280939
61924895	CBYP4092.g1	GE280940
61924896	CBYP4093.b1	GE280941
61924897	CBYP4093.g1	GE280942
61924898	CBYP4094.b1	GE280943
61924899	CBYP4094.g1	GE280944
61924900	CBYP4095.b1	GE280945
61924901	CBYP4095.g1	GE280946
61924902	CBYP4096.b1	GE280947
61924903	CBYP4096.g1	GE280948
61924904	CBYP4097.b1	GE280949
61924905	CBYP4097.g1	GE280950
61924906	CBYP4099.b1	GE280951
61924907	CBYP4099.g1	GE280952
61924908	CBYP4100.b1	GE280953

61924909	CBYP4100.g1	GE280954
61924910	CBYP4101.b1	GE280955
61924911	CBYP4101.g1	GE280956
61924912	CBYP4102.b1	GE280957
61924913	CBYP4102.g1	GE280958
61924914	CBYP4103.b1	GE280959
61924915	CBYP4103.g1	GE280960
61924916	CBYP4104.b1	GE280961
61924917	CBYP4104.g1	GE280962
61924918	CBYP4106.b1	GE280963
61924919	CBYP4106.g1	GE280964
61924920	CBYP4107.b1	GE280965
61924921	CBYP4107.g1	GE280966
61924922	CBYP4108.b1	GE280967
61924923	CBYP4108.g1	GE280968
61924924	CBYP4109.b1	GE280969
61924925	CBYP4109.g1	GE280970
61924926	CBYP4110.b1	GE280971
61924927	CBYP4110.g1	GE280972
61924928	CBYP4111.b1	GE280973
61924929	CBYP4111.g1	GE280974
61924930	CBYP4112.b1	GE280975
61924931	CBYP4112.g1	GE280976
61924932	CBYP4113.b1	GE280977
61924933	CBYP4113.g1	GE280978
61924934	CBYP4114.g1	GE280979
61924935	CBYP4115.g1	GE280980
61924936	CBYP4116.b1	GE280981
61924937	CBYP4116.g1	GE280982
61924938	CBYP4117.b1	GE280983
61924939	CBYP4117.g1	GE280984
61924940	CBYP4118.b1	GE280985
61924941	CBYP4118.g1	GE280986
61924942	CBYP4119.b1	GE280987
61924943	CBYP4119.g1	GE280988
61924944	CBYP4121.b1	GE280989
61924945	CBYP4122.g1	GE280990
61924946	CBYP4123.b1	GE280991
61924947	CBYP4123.g1	GE280992
61924948	CBYP4124.b1	GE280993
61924949	CBYP4124.g1	GE280994
61924950	CBYP4125.b1	GE280995
61924951	CBYP4125.g1	GE280996
61924952	CBYP4126.b1	GE280997
61924953	CBYP4126.g1	GE280998
61924954	CBYP4127.g1	GE280999
61924955	CBYP4128.b1	GE281000
61924956	CBYP4128.g1	GE281001
61924957	CBYP4129.b1	GE281002
61924958	CBYP4129.g1	GE281003
61924959	CBYP4130.b1	GE281004
61924960	CBYP4130.g1	GE281005
61924961	CBYP4131.b1	GE281006
61924962	CBYP4131.g1	GE281007
61924963	CBYP4132.b1	GE281008
61924964	CBYP4132.g1	GE281009
61924965	CBYP4133.b1	GE281010
61924966	CBYP4133.g1	GE281011
61924967	CBYP4134.b1	GE281012
61924968	CBYP4134.g1	GE281013
61924969	CBYP4135.b1	GE281014
61924970	CBYP4135.g1	GE281015
61924971	CBYP4136.b1	GE281016

61924972	CBYP4137.b1	GE281017
61924973	CBYP4138.b1	GE281018
61924974	CBYP4138.g1	GE281019
61924975	CBYP4139.b1	GE281020
61924976	CBYP4139.g1	GE281021
61924977	CBYP4140.b1	GE281022
61924978	CBYP4140.g1	GE281023
61924979	CBYP4141.b1	GE281024
61924980	CBYP4141.g1	GE281025
61924981	CBYP4142.b1	GE281026
61924982	CBYP4142.g1	GE281027
61924983	CBYP4143.b1	GE281028
61924984	CBYP4143.g1	GE281029
61924985	CBYP4145.b1	GE281030
61924986	CBYP4145.g1	GE281031
61924987	CBYP4146.b1	GE281032
61924988	CBYP4146.g1	GE281033
61924989	CBYP4147.b1	GE281034
61924990	CBYP4147.g1	GE281035
61924991	CBYP4148.b1	GE281036
61924992	CBYP4148.g1	GE281037
61924993	CBYP4150.b1	GE281038
61924994	CBYP4150.g1	GE281039
61924995	CBYP4151.b1	GE281040
61924996	CBYP4151.g1	GE281041
61924997	CBYP4152.b1	GE281042
61924998	CBYP4152.g1	GE281043
61924999	CBYP4153.b1	GE281044
61925000	CBYP4153.g1	GE281045
61925001	CBYP4154.b1	GE281046
61925002	CBYP4154.g1	GE281047
61925003	CBYP4155.b1	GE281048
61925004	CBYP4155.g1	GE281049
61925005	CBYP4156.b1	GE281050
61925006	CBYP4157.b1	GE281051
61925007	CBYP4157.g1	GE281052
61925008	CBYP4158.b1	GE281053
61925009	CBYP4158.g1	GE281054
61925010	CBYP4159.b1	GE281055
61925011	CBYP4160.b1	GE281056
61925012	CBYP4160.g1	GE281057
61925013	CBYP4161.b1	GE281058
61925014	CBYP4161.g1	GE281059
61925015	CBYP4162.b1	GE281060
61925016	CBYP4162.g1	GE281061
61925017	CBYP4163.b1	GE281062
61925018	CBYP4163.g1	GE281063
61925019	CBYP4164.b1	GE281064
61925020	CBYP4166.b1	GE281065
61925021	CBYP4166.g1	GE281066
61925022	CBYP4167.b1	GE281067
61925023	CBYP4167.g1	GE281068
61925024	CBYP4168.b1	GE281069
61925025	CBYP4168.g1	GE281070
61925026	CBYP4169.b1	GE281071
61925027	CBYP4169.g1	GE281072
61925028	CBYP4170.b1	GE281073
61925029	CBYP4170.g1	GE281074
61925030	CBYP4171.b1	GE281075
61925031	CBYP4171.g1	GE281076
61925032	CBYP4173.b1	GE281077
61925033	CBYP4173.g1	GE281078
61925034	CBYP4174.g1	GE281079

61925035	CBYP4175.g1	GE281080
61925036	CBYP4176.b1	GE281081
61925037	CBYP4176.g1	GE281082
61925038	CBYP4177.b1	GE281083
61925039	CBYP4178.b1	GE281084
61925040	CBYP4178.g1	GE281085
61925041	CBYP4179.b1	GE281086
61925042	CBYP4179.g1	GE281087
61925043	CBYP4181.b1	GE281088
61925044	CBYP4181.g1	GE281089
61925045	CBYP4182.b1	GE281090
61925046	CBYP4182.g1	GE281091
61925047	CBYP4183.b1	GE281092
61925048	CBYP4183.g1	GE281093
61925049	CBYP4184.b1	GE281094
61925050	CBYP4184.g1	GE281095
61925051	CBYP4185.b1	GE281096
61925052	CBYP4185.g1	GE281097
61925053	CBYP4186.b1	GE281098
61925054	CBYP4186.g1	GE281099
61925055	CBYP4187.b1	GE281100
61925056	CBYP4187.g1	GE281101
61925057	CBYP4188.b1	GE281102
61925058	CBYP4188.g1	GE281103
61925059	CBYP4189.b1	GE281104
61925060	CBYP4191.b1	GE281105
61925061	CBYP4191.g1	GE281106
61925062	CBYP4192.b1	GE281107
61925063	CBYP4192.g1	GE281108
61925064	CBYP4193.b1	GE281109
61925065	CBYP4193.g1	GE281110
61925066	CBYP4194.b1	GE281111
61925067	CBYP4194.g1	GE281112
61925068	CBYP4195.b1	GE281113
61925069	CBYP4195.g1	GE281114
61925070	CBYP4196.b1	GE281115
61925071	CBYP4196.g1	GE281116
61925072	CBYP4197.b1	GE281117
61925073	CBYP4197.g1	GE281118
61925074	CBYP4198.b1	GE281119
61925075	CBYP4199.b1	GE281120
61925076	CBYP4199.g1	GE281121
61925077	CBYP4200.b1	GE281122
61925078	CBYP4201.b1	GE281123
61925079	CBYP4201.g1	GE281124
61925080	CBYP4202.b1	GE281125
61925081	CBYP4202.g1	GE281126
61925082	CBYP4203.b1	GE281127
61925083	CBYP4203.g1	GE281128
61925084	CBYP4205.b1	GE281129
61925085	CBYP4205.g1	GE281130
61925086	CBYP4206.b1	GE281131
61925087	CBYP4206.g1	GE281132
61925088	CBYP4208.b1	GE281133
61925089	CBYP4208.g1	GE281134
61925090	CBYP4209.b1	GE281135
61925091	CBYP4209.g1	GE281136
61925092	CBYP4212.b1	GE281137
61925093	CBYP4212.g1	GE281138
61925094	CBYP4213.b1	GE281139
61925095	CBYP4213.g1	GE281140
61925096	CBYP4214.b1	GE281141
61925097	CBYP4214.g1	GE281142

61925098	CBYP4215.b1	GE281143
61925099	CBYP4215.g1	GE281144
61925100	CBYP4216.b1	GE281145
61925101	CBYP4216.g1	GE281146
61925102	CBYP4217.b1	GE281147
61925103	CBYP4218.b1	GE281148
61925104	CBYP4218.g1	GE281149
61925105	CBYP4219.b1	GE281150
61925106	CBYP4219.g1	GE281151
61925107	CBYP4220.g1	GE281152
61925108	CBYP4221.b1	GE281153
61925109	CBYP4221.g1	GE281154
61925110	CBYP4222.b1	GE281155
61925111	CBYP4222.g1	GE281156
61925112	CBYP4224.g1	GE281157
61925113	CBYP4225.g1	GE281158
61925114	CBYP4227.b1	GE281159
61925115	CBYP4227.g1	GE281160
61925116	CBYP4229.b1	GE281161
61925117	CBYP4229.g1	GE281162
61925118	CBYP4230.b1	GE281163
61925119	CBYP4230.g1	GE281164
61925120	CBYP4231.b1	GE281165
61925121	CBYP4231.g1	GE281166
61925122	CBYP4232.b1	GE281167
61925123	CBYP4232.g1	GE281168
61925124	CBYP4233.b1	GE281169
61925125	CBYP4233.g1	GE281170
61925126	CBYP4234.b1	GE281171
61925127	CBYP4234.g1	GE281172
61925128	CBYP4235.b1	GE281173
61925129	CBYP4235.g1	GE281174
61925130	CBYP4236.b1	GE281175
61925131	CBYP4236.g1	GE281176
61925132	CBYP4237.b1	GE281177
61925133	CBYP4237.g1	GE281178
61925134	CBYP4238.b1	GE281179
61925135	CBYP4238.g1	GE281180
61925136	CBYP4239.b1	GE281181
61925137	CBYP4239.g1	GE281182
61925138	CBYP4240.b1	GE281183
61925139	CBYP4240.g1	GE281184
61925140	CBYP4241.b1	GE281185
61925141	CBYP4242.b1	GE281186
61925142	CBYP4242.g1	GE281187
61925143	CBYP4243.b1	GE281188
61925144	CBYP4243.g1	GE281189
61925145	CBYP4244.b1	GE281190
61925146	CBYP4244.g1	GE281191
61925147	CBYP4245.b1	GE281192
61925148	CBYP4245.g1	GE281193
61925149	CBYP4246.b1	GE281194
61925150	CBYP4246.g1	GE281195
61925151	CBYP4248.b1	GE281196
61925152	CBYP4248.g1	GE281197
61925153	CBYP4249.b1	GE281198
61925154	CBYP4249.g1	GE281199
61925155	CBYP4250.b1	GE281200
61925156	CBYP4250.g1	GE281201
61925157	CBYP4252.b1	GE281202
61925158	CBYP4252.g1	GE281203
61925159	CBYP4253.b1	GE281204
61925160	CBYP4253.g1	GE281205

61925161	CBYP4254.b1	GE281206
61925162	CBYP4254.g1	GE281207
61925163	CBYP4255.b1	GE281208
61925164	CBYP4255.g1	GE281209
61925165	CBYP4257.b1	GE281210
61925166	CBYP4257.g1	GE281211
61925167	CBYP4258.b1	GE281212
61925168	CBYP4258.g1	GE281213
61925169	CBYP4259.b1	GE281214
61925170	CBYP4259.g1	GE281215
61925171	CBYP4260.b1	GE281216
61925172	CBYP4260.g1	GE281217
61925173	CBYP4261.b1	GE281218
61925174	CBYP4261.g1	GE281219
61925175	CBYP4262.b1	GE281220
61925176	CBYP4264.b1	GE281221
61925177	CBYP4264.g1	GE281222
61925178	CBYP4265.b1	GE281223
61925179	CBYP4265.g1	GE281224
61925180	CBYP4266.b1	GE281225
61925181	CBYP4266.g1	GE281226
61925182	CBYP4267.b1	GE281227
61925183	CBYP4267.g1	GE281228
61925184	CBYP4268.b1	GE281229
61925185	CBYP4268.g1	GE281230
61925186	CBYP4270.b1	GE281231
61925187	CBYP4270.g1	GE281232
61925188	CBYP4271.b1	GE281233
61925189	CBYP4271.g1	GE281234
61925190	CBYP4272.b1	GE281235
61925191	CBYP4272.g1	GE281236
61925192	CBYP4274.b1	GE281237
61925193	CBYP4274.g1	GE281238
61925194	CBYP4275.b1	GE281239
61925195	CBYP4276.b1	GE281240
61925196	CBYP4276.g1	GE281241
61925197	CBYP4278.b1	GE281242
61925198	CBYP4278.g1	GE281243
61925199	CBYP4279.b1	GE281244
61925200	CBYP4279.g1	GE281245
61925201	CBYP4280.b1	GE281246
61925202	CBYP4280.g1	GE281247
61925203	CBYP4281.b1	GE281248
61925204	CBYP4281.g1	GE281249
61925205	CBYP4282.b1	GE281250
61925206	CBYP4282.g1	GE281251
61925207	CBYP4283.b1	GE281252
61925208	CBYP4283.g1	GE281253
61925209	CBYP4284.b1	GE281254
61925210	CBYP4284.g1	GE281255
61925211	CBYP4285.b1	GE281256
61925212	CBYP4285.g1	GE281257
61925213	CBYP4286.b1	GE281258
61925214	CBYP4286.g1	GE281259
61925215	CBYP4287.b1	GE281260
61925216	CBYP4287.g1	GE281261
61925217	CBYP4288.g1	GE281262
61925218	CBYP4290.b1	GE281263
61925219	CBYP4290.g1	GE281264
61925220	CBYP4293.b1	GE281265
61925221	CBYP4293.g1	GE281266
61925222	CBYP4295.b1	GE281267
61925223	CBYP4295.g1	GE281268

61925224	CBYP4296.b1	GE281269
61925225	CBYP4296.g1	GE281270
61925226	CBYP4297.b1	GE281271
61925227	CBYP4297.g1	GE281272
61925228	CBYP4299.b1	GE281273
61925229	CBYP4299.g1	GE281274
61925230	CBYP4300.b1	GE281275
61925231	CBYP4300.g1	GE281276
61925232	CBYP4301.b1	GE281277
61925233	CBYP4301.g1	GE281278
61925234	CBYP4302.b1	GE281279
61925235	CBYP4302.g1	GE281280
61925236	CBYP4303.b1	GE281281
61925237	CBYP4303.g1	GE281282
61925238	CBYP4304.b1	GE281283
61925239	CBYP4304.g1	GE281284
61925240	CBYP4305.b1	GE281285
61925241	CBYP4305.g1	GE281286
61925242	CBYP4306.b1	GE281287
61925243	CBYP4306.g1	GE281288
61925244	CBYP4307.g1	GE281289
61925245	CBYP4308.b1	GE281290
61925246	CBYP4308.g1	GE281291
61925247	CBYP4309.b1	GE281292
61925248	CBYP4309.g1	GE281293
61925249	CBYP4310.b1	GE281294
61925250	CBYP4310.g1	GE281295
61925251	CBYP4312.b1	GE281296
61925252	CBYP4312.g1	GE281297
61925253	CBYP4314.b1	GE281298
61925254	CBYP4314.g1	GE281299
61925255	CBYP4315.b1	GE281300
61925256	CBYP4315.g1	GE281301
61925257	CBYP4316.b1	GE281302
61925258	CBYP4316.g1	GE281303
61925259	CBYP4317.b1	GE281304
61925260	CBYP4317.g1	GE281305
61925261	CBYP4318.b1	GE281306
61925262	CBYP4318.g1	GE281307
61925263	CBYP4319.b1	GE281308
61925264	CBYP4319.g1	GE281309
61925265	CBYP4320.b1	GE281310
61925266	CBYP4320.g1	GE281311
61925267	CBYP4321.b1	GE281312
61925268	CBYP4321.g1	GE281313
61925269	CBYP4322.b1	GE281314
61925270	CBYP4322.g1	GE281315
61925271	CBYP4323.b1	GE281316
61925272	CBYP4323.g1	GE281317
61925273	CBYP4324.b1	GE281318
61925274	CBYP4324.g1	GE281319
61925275	CBYP4325.b1	GE281320
61925276	CBYP4325.g1	GE281321
61925277	CBYP4327.b1	GE281322
61925278	CBYP4327.g1	GE281323
61925279	CBYP4328.b1	GE281324
61925280	CBYP4329.b1	GE281325
61925281	CBYP4329.g1	GE281326
61925282	CBYP4330.b1	GE281327
61925283	CBYP4330.g1	GE281328
61925284	CBYP4331.b1	GE281329
61925285	CBYP4331.g1	GE281330
61925286	CBYP4332.b1	GE281331

61925287	CBYP4332.g1	GE281332
61925288	CBYP4333.b1	GE281333
61925289	CBYP4333.g1	GE281334
61925290	CBYP4334.b1	GE281335
61925291	CBYP4334.g1	GE281336
61925292	CBYP4335.b1	GE281337
61925293	CBYP4336.b1	GE281338
61925294	CBYP4336.g1	GE281339
61925295	CBYP4337.b1	GE281340
61925296	CBYP4337.g1	GE281341
61925297	CBYP4339.b1	GE281342
61925298	CBYP4339.g1	GE281343
61925299	CBYP4340.b1	GE281344
61925300	CBYP4340.g1	GE281345
61925301	CBYP4341.b1	GE281346
61925302	CBYP4341.g1	GE281347
61925303	CBYP4342.b1	GE281348
61925304	CBYP4342.g1	GE281349
61925305	CBYP4344.b1	GE281350
61925306	CBYP4344.g1	GE281351
61925307	CBYP4345.b1	GE281352
61925308	CBYP4345.g1	GE281353
61925309	CBYP4346.b1	GE281354
61925310	CBYP4346.g1	GE281355
61925311	CBYP4347.b1	GE281356
61925312	CBYP4347.g1	GE281357
61925313	CBYP4349.b1	GE281358
61925314	CBYP4349.g1	GE281359
61925315	CBYP4350.b1	GE281360
61925316	CBYP4350.g1	GE281361
61925317	CBYP4351.b1	GE281362
61925318	CBYP4351.g1	GE281363
61925319	CBYP4352.b1	GE281364
61925320	CBYP4352.g1	GE281365
61925321	CBYP4353.b1	GE281366
61925322	CBYP4353.g1	GE281367
61925323	CBYP4354.b1	GE281368
61925324	CBYP4354.g1	GE281369
61925325	CBYP4355.b1	GE281370
61925326	CBYP4355.g1	GE281371
61925327	CBYP4356.b1	GE281372
61925328	CBYP4356.g1	GE281373
61925329	CBYP4357.b1	GE281374
61925330	CBYP4357.g1	GE281375
61925331	CBYP4358.g1	GE281376
61925332	CBYP4359.b1	GE281377
61925333	CBYP4359.g1	GE281378
61925334	CBYP4360.b1	GE281379
61925335	CBYP4360.g1	GE281380
61925336	CBYP4361.b1	GE281381
61925337	CBYP4362.b1	GE281382
61925338	CBYP4362.g1	GE281383
61925339	CBYP4363.b1	GE281384
61925340	CBYP4363.g1	GE281385
61925341	CBYP4364.b1	GE281386
61925342	CBYP4364.g1	GE281387
61925343	CBYP4365.b1	GE281388
61925344	CBYP4365.g1	GE281389
61925345	CBYP4366.b1	GE281390
61925346	CBYP4366.g1	GE281391
61925347	CBYP4367.b1	GE281392
61925348	CBYP4368.b1	GE281393
61925349	CBYP4368.g1	GE281394

61925350	CBYP4369.b1	GE281395
61925351	CBYP4369.g1	GE281396
61925352	CBYP4370.b1	GE281397
61925353	CBYP4370.g1	GE281398
61925354	CBYP4371.b1	GE281399
61925355	CBYP4371.g1	GE281400
61925356	CBYP4372.b1	GE281401
61925357	CBYP4372.g1	GE281402
61925358	CBYP4373.b1	GE281403
61925359	CBYP4373.g1	GE281404
61925360	CBYP4374.b1	GE281405
61925361	CBYP4374.g1	GE281406
61925362	CBYP4375.b1	GE281407
61925363	CBYP4375.g1	GE281408
61925364	CBYP4376.b1	GE281409
61925365	CBYP4376.g1	GE281410
61925366	CBYP4377.b1	GE281411
61925367	CBYP4377.g1	GE281412
61925368	CBYP4378.b1	GE281413
61925369	CBYP4378.g1	GE281414
61925370	CBYP4379.b1	GE281415
61925371	CBYP4379.g1	GE281416
61925372	CBYP4380.b1	GE281417
61925373	CBYP4380.g1	GE281418
61925374	CBYP4381.b1	GE281419
61925375	CBYP4381.g1	GE281420
61925376	CBYP4382.b1	GE281421
61925377	CBYP4382.g1	GE281422
61925378	CBYP4384.b1	GE281423
61925379	CBYP4384.g1	GE281424
61925380	CBYP4385.b1	GE281425
61925381	CBYP4385.g1	GE281426
61925382	CBYP4386.b1	GE281427
61925383	CBYP4386.g1	GE281428
61925384	CBYP4387.b1	GE281429
61925385	CBYP4387.g1	GE281430
61925386	CBYP4388.b1	GE281431
61925387	CBYP4388.g1	GE281432
61925388	CBYP4389.b1	GE281433
61925389	CBYP4389.g1	GE281434
61925390	CBYP4390.b1	GE281435
61925391	CBYP4390.g1	GE281436
61925392	CBYP4391.b1	GE281437
61925393	CBYP4391.g1	GE281438
61925394	CBYP4392.b1	GE281439
61925395	CBYP4392.g1	GE281440
61925396	CBYP4393.b1	GE281441
61925397	CBYP4393.g1	GE281442
61925398	CBYP4394.b1	GE281443
61925399	CBYP4394.g1	GE281444
61925400	CBYP4395.b1	GE281445
61925401	CBYP4395.g1	GE281446
61925402	CBYP4396.b1	GE281447
61925403	CBYP4396.g1	GE281448
61925404	CBYP4398.b1	GE281449
61925405	CBYP4398.g1	GE281450
61925406	CBYP4399.b1	GE281451
61925407	CBYP4399.g1	GE281452
61925408	CBYP4400.b1	GE281453
61925409	CBYP4400.g1	GE281454
61925410	CBYP4401.b1	GE281455
61925411	CBYP4401.g1	GE281456
61925412	CBYP4402.b1	GE281457

61925413	CBYP4402.g1	GE281458
61925414	CBYP4403.g1	GE281459
61925415	CBYP4404.b1	GE281460
61925416	CBYP4404.g1	GE281461
61925417	CBYP4405.b1	GE281462
61925418	CBYP4405.g1	GE281463
61925419	CBYP4406.b1	GE281464
61925420	CBYP4406.g1	GE281465
61925421	CBYP4407.b1	GE281466
61925422	CBYP4407.g1	GE281467
61925423	CBYP4408.b1	GE281468
61925424	CBYP4408.g1	GE281469
61925425	CBYP4409.b1	GE281470
61925426	CBYP4410.g1	GE281471
61925427	CBYP4411.b1	GE281472
61925428	CBYP4411.g1	GE281473
61925429	CBYP4412.b1	GE281474
61925430	CBYP4412.g1	GE281475
61925431	CBYP4413.b1	GE281476
61925432	CBYP4413.g1	GE281477
61925433	CBYP4414.b1	GE281478
61925434	CBYP4414.g1	GE281479
61925435	CBYP4415.b1	GE281480
61925436	CBYP4415.g1	GE281481
61925437	CBYP4416.b1	GE281482
61925438	CBYP4416.g1	GE281483
61925439	CBYP4417.b1	GE281484
61925440	CBYP4417.g1	GE281485
61925441	CBYP4418.b1	GE281486
61925442	CBYP4418.g1	GE281487
61925443	CBYP4419.b1	GE281488
61925444	CBYP4419.g1	GE281489
61925445	CBYP4421.b1	GE281490
61925446	CBYP4421.g1	GE281491
61925447	CBYP4422.b1	GE281492
61925448	CBYP4422.g1	GE281493
61925449	CBYP4423.b1	GE281494
61925450	CBYP4423.g1	GE281495
61925451	CBYP4424.b1	GE281496
61925452	CBYP4424.g1	GE281497
61925453	CBYP4425.b1	GE281498
61925454	CBYP4425.g1	GE281499
61925455	CBYP4426.b1	GE281500
61925456	CBYP4426.g1	GE281501
61925457	CBYP4427.b1	GE281502
61925458	CBYP4427.g1	GE281503
61925459	CBYP4428.b1	GE281504
61925460	CBYP4428.g1	GE281505
61925461	CBYP4430.b1	GE281506
61925462	CBYP4430.g1	GE281507
61925463	CBYP4431.b1	GE281508
61925464	CBYP4431.g1	GE281509
61925465	CBYP4432.b1	GE281510
61925466	CBYP4432.g1	GE281511
61925467	CBYP4433.b1	GE281512
61925468	CBYP4433.g1	GE281513
61925469	CBYP4435.b1	GE281514
61925470	CBYP4435.g1	GE281515
61925471	CBYP4436.b1	GE281516
61925472	CBYP4436.g1	GE281517
61925473	CBYP4437.b1	GE281518
61925474	CBYP4437.g1	GE281519
61925475	CBYP4439.b1	GE281520

61925476	CBYP4439.g1	GE281521
61925477	CBYP4440.b1	GE281522
61925478	CBYP4440.g1	GE281523
61925479	CBYP4441.b1	GE281524
61925480	CBYP4441.g1	GE281525
61925481	CBYP4442.b1	GE281526
61925482	CBYP4442.g1	GE281527
61925483	CBYP4443.b1	GE281528
61925484	CBYP4443.g1	GE281529
61925485	CBYP4444.b1	GE281530
61925486	CBYP4444.g1	GE281531
61925487	CBYP4445.b1	GE281532
61925488	CBYP4445.g1	GE281533
61925489	CBYP4446.b1	GE281534
61925490	CBYP4446.g1	GE281535
61925491	CBYP4448.b1	GE281536
61925492	CBYP4448.g1	GE281537
61925493	CBYP4449.b1	GE281538
61925494	CBYP4449.g1	GE281539
61925495	CBYP4451.b1	GE281540
61925496	CBYP4451.g1	GE281541
61925497	CBYP4452.b1	GE281542
61925498	CBYP4452.g1	GE281543
61925499	CBYP4453.g1	GE281544
61925500	CBYP4454.b1	GE281545
61925501	CBYP4454.g1	GE281546
61925502	CBYP4455.b1	GE281547
61925503	CBYP4455.g1	GE281548
61925504	CBYP4456.b1	GE281549
61925505	CBYP4456.g1	GE281550
61925506	CBYP4457.b1	GE281551
61925507	CBYP4457.g1	GE281552
61925508	CBYP4458.b1	GE281553
61925509	CBYP4458.g1	GE281554
61925510	CBYP4459.b1	GE281555
61925511	CBYP4459.g1	GE281556
61925512	CBYP4461.b1	GE281557
61925513	CBYP4461.g1	GE281558
61925514	CBYP4463.b1	GE281559
61925515	CBYP4463.g1	GE281560
61925516	CBYP4464.b1	GE281561
61925517	CBYP4464.g1	GE281562
61925518	CBYP4465.b1	GE281563
61925519	CBYP4466.b1	GE281564
61925520	CBYP4466.g1	GE281565
61925521	CBYP4468.b1	GE281566
61925522	CBYP4468.g1	GE281567
61925523	CBYP4469.b1	GE281568
61925524	CBYP4469.g1	GE281569
61925525	CBYP4470.b1	GE281570
61925526	CBYP4471.b1	GE281571
61925527	CBYP4471.g1	GE281572
61925528	CBYP4472.b1	GE281573
61925529	CBYP4472.g1	GE281574
61925530	CBYP4473.b1	GE281575
61925531	CBYP4473.g1	GE281576
61925532	CBYP4474.b1	GE281577
61925533	CBYP4474.g1	GE281578
61925534	CBYP4475.b1	GE281579
61925535	CBYP4475.g1	GE281580
61925536	CBYP4476.b1	GE281581
61925537	CBYP4476.g1	GE281582
61925538	CBYP4477.g1	GE281583

61925539	CBYP4478.b1	GE281584
61925540	CBYP4478.g1	GE281585
61925541	CBYP4479.b1	GE281586
61925542	CBYP4479.g1	GE281587
61925543	CBYP4480.b1	GE281588
61925544	CBYP4480.g1	GE281589
61925545	CBYP4482.b1	GE281590
61925546	CBYP4482.g1	GE281591
61925547	CBYP4483.b1	GE281592
61925548	CBYP4483.g1	GE281593
61925549	CBYP4484.b1	GE281594
61925550	CBYP4484.g1	GE281595
61925551	CBYP4485.b1	GE281596
61925552	CBYP4485.g1	GE281597
61925553	CBYP4486.b1	GE281598
61925554	CBYP4486.g1	GE281599
61925555	CBYP4487.b1	GE281600
61925556	CBYP4487.g1	GE281601
61925557	CBYP4488.b1	GE281602
61925558	CBYP4488.g1	GE281603
61925559	CBYP4489.b1	GE281604
61925560	CBYP4489.g1	GE281605
61925561	CBYP4490.b1	GE281606
61925562	CBYP4490.g1	GE281607
61925563	CBYP4491.b1	GE281608
61925564	CBYP4491.g1	GE281609
61925565	CBYP4492.b1	GE281610
61925566	CBYP4492.g1	GE281611
61925567	CBYP4493.b1	GE281612
61925568	CBYP4494.b1	GE281613
61925569	CBYP4494.g1	GE281614
61925570	CBYP4495.b1	GE281615
61925571	CBYP4495.g1	GE281616
61925572	CBYP4497.b1	GE281617
61925573	CBYP4497.g1	GE281618
61925574	CBYP4498.b1	GE281619
61925575	CBYP4498.g1	GE281620
61925576	CBYP4499.b1	GE281621
61925577	CBYP4499.g1	GE281622
61925578	CBYP4500.b1	GE281623
61925579	CBYP4500.g1	GE281624
61925580	CBYP4501.b1	GE281625
61925581	CBYP4501.g1	GE281626
61925582	CBYP4502.b1	GE281627
61925583	CBYP4502.g1	GE281628
61925584	CBYP4503.b1	GE281629
61925585	CBYP4503.g1	GE281630
61925586	CBYP4505.b1	GE281631
61925587	CBYP4505.g1	GE281632
61925588	CBYP4506.b1	GE281633
61925589	CBYP4506.g1	GE281634
61925590	CBYP4507.b1	GE281635
61925591	CBYP4507.g1	GE281636
61925592	CBYP4508.b1	GE281637
61925593	CBYP4509.b1	GE281638
61925594	CBYP4509.g1	GE281639
61925595	CBYP4510.b1	GE281640
61925596	CBYP4511.b1	GE281641
61925597	CBYP4511.g1	GE281642
61925598	CBYP4515.b1	GE281643
61925599	CBYP4515.g1	GE281644
61925600	CBYP4516.b1	GE281645
61925601	CBYP4516.g1	GE281646

61925602	CBYP4517.b1	GE281647
61925603	CBYP4517.g1	GE281648
61925604	CBYP4518.b1	GE281649
61925605	CBYP4518.g1	GE281650
61925606	CBYP4519.b1	GE281651
61925607	CBYP4519.g1	GE281652
61925608	CBYP4520.b1	GE281653
61925609	CBYP4520.g1	GE281654
61925610	CBYP4521.b1	GE281655
61925611	CBYP4521.g1	GE281656
61925612	CBYP4522.b1	GE281657
61925613	CBYP4522.g1	GE281658
61925614	CBYP4523.b1	GE281659
61925615	CBYP4524.b1	GE281660
61925616	CBYP4524.g1	GE281661
61925617	CBYP4525.b1	GE281662
61925618	CBYP4525.g1	GE281663
61925619	CBYP4526.b1	GE281664
61925620	CBYP4526.g1	GE281665
61925621	CBYP4527.b1	GE281666
61925622	CBYP4527.g1	GE281667
61925623	CBYP4528.b1	GE281668
61925624	CBYP4528.g1	GE281669
61925625	CBYP4529.b1	GE281670
61925626	CBYP4529.g1	GE281671
61925627	CBYP4530.b1	GE281672
61925628	CBYP4530.g1	GE281673
61925629	CBYP4531.b1	GE281674
61925630	CBYP4532.b1	GE281675
61925631	CBYP4532.g1	GE281676
61925632	CBYP4534.b1	GE281677
61925633	CBYP4534.g1	GE281678
61925634	CBYP4535.b1	GE281679
61925635	CBYP4535.g1	GE281680
61925636	CBYP4536.b1	GE281681
61925637	CBYP4536.g1	GE281682
61925638	CBYP4537.b1	GE281683
61925639	CBYP4537.g1	GE281684
61925640	CBYP4538.g1	GE281685
61925641	CBYP4539.b1	GE281686
61925642	CBYP4539.g1	GE281687
61925643	CBYP4540.g1	GE281688
61925644	CBYP4541.b1	GE281689
61925645	CBYP4541.g1	GE281690
61925646	CBYP4542.g1	GE281691
61925647	CBYP4543.b1	GE281692
61925648	CBYP4543.g1	GE281693
61925649	CBYP4544.b1	GE281694
61925650	CBYP4544.g1	GE281695
61925651	CBYP4545.g1	GE281696
61925652	CBYP4546.b1	GE281697
61925653	CBYP4546.g1	GE281698
61925654	CBYP4547.b1	GE281699
61925655	CBYP4547.g1	GE281700
61925656	CBYP4548.b1	GE281701
61925657	CBYP4548.g1	GE281702
61925658	CBYP4549.b1	GE281703
61925659	CBYP4549.g1	GE281704
61925660	CBYP4550.b1	GE281705
61925661	CBYP4550.g1	GE281706
61925662	CBYP4551.b1	GE281707
61925663	CBYP4551.g1	GE281708
61925664	CBYP4552.b1	GE281709

61925665	CBYP4552.g1	GE281710
61925666	CBYP4553.b1	GE281711
61925667	CBYP4553.g1	GE281712
61925668	CBYP4554.b1	GE281713
61925669	CBYP4554.g1	GE281714
61925670	CBYP4555.b1	GE281715
61925671	CBYP4555.g1	GE281716
61925672	CBYP4556.b1	GE281717
61925673	CBYP4556.g1	GE281718
61925674	CBYP4557.b1	GE281719
61925675	CBYP4557.g1	GE281720
61925676	CBYP4558.b1	GE281721
61925677	CBYP4558.g1	GE281722
61925678	CBYP4559.b1	GE281723
61925679	CBYP4559.g1	GE281724
61925680	CBYP4560.b1	GE281725
61925681	CBYP4560.g1	GE281726
61925682	CBYP4561.b1	GE281727
61925683	CBYP4561.g1	GE281728
61925684	CBYP4562.b1	GE281729
61925685	CBYP4562.g1	GE281730
61925686	CBYP4563.b1	GE281731
61925687	CBYP4564.b1	GE281732
61925688	CBYP4564.g1	GE281733
61925689	CBYP4565.b1	GE281734
61925690	CBYP4565.g1	GE281735
61925691	CBYP4566.b1	GE281736
61925692	CBYP4566.g1	GE281737
61925693	CBYP4567.b1	GE281738
61925694	CBYP4567.g1	GE281739
61925695	CBYP4568.b1	GE281740
61925696	CBYP4568.g1	GE281741
61925697	CBYP4569.b1	GE281742
61925698	CBYP4569.g1	GE281743
61925699	CBYP4570.b1	GE281744
61925700	CBYP4570.g1	GE281745
61925701	CBYP4571.b1	GE281746
61925702	CBYP4571.g1	GE281747
61925703	CBYP4572.b1	GE281748
61925704	CBYP4572.g1	GE281749
61925705	CBYP4573.b1	GE281750
61925706	CBYP4573.g1	GE281751
61925707	CBYP4574.b1	GE281752
61925708	CBYP4574.g1	GE281753
61925709	CBYP4575.b1	GE281754
61925710	CBYP4575.g1	GE281755
61925711	CBYP4576.b1	GE281756
61925712	CBYP4576.g1	GE281757
61925713	CBYP4577.b1	GE281758
61925714	CBYP4577.g1	GE281759
61925715	CBYP4578.b1	GE281760
61925716	CBYP4578.g1	GE281761
61925717	CBYP4579.b1	GE281762
61925718	CBYP4579.g1	GE281763
61925719	CBYP4580.b1	GE281764
61925720	CBYP4580.g1	GE281765
61925721	CBYP4581.b1	GE281766
61925722	CBYP4581.g1	GE281767
61925723	CBYP4582.b1	GE281768
61925724	CBYP4582.g1	GE281769
61925725	CBYP4584.b1	GE281770
61925726	CBYP4584.g1	GE281771
61925727	CBYP4585.b1	GE281772

61925728	CBYP4585.g1	GE281773
61925729	CBYP4586.b1	GE281774
61925730	CBYP4586.g1	GE281775
61925731	CBYP4587.b1	GE281776
61925732	CBYP4587.g1	GE281777
61925733	CBYP4588.b1	GE281778
61925734	CBYP4588.g1	GE281779
61925735	CBYP4589.b1	GE281780
61925736	CBYP4589.g1	GE281781
61925737	CBYP4590.b1	GE281782
61925738	CBYP4590.g1	GE281783
61925739	CBYP4591.g1	GE281784
61925740	CBYP4592.b1	GE281785
61925741	CBYP4592.g1	GE281786
61925742	CBYP4593.b1	GE281787
61925743	CBYP4593.g1	GE281788
61925744	CBYP4594.b1	GE281789
61925745	CBYP4594.g1	GE281790
61925746	CBYP4595.b1	GE281791
61925747	CBYP4595.g1	GE281792
61925748	CBYP4596.b1	GE281793
61925749	CBYP4596.g1	GE281794
61925750	CBYP4597.b1	GE281795
61925751	CBYP4597.g1	GE281796
61925752	CBYP4598.b1	GE281797
61925753	CBYP4598.g1	GE281798
61925754	CBYP4599.b1	GE281799
61925755	CBYP4599.g1	GE281800
61925756	CBYP4600.b1	GE281801
61925757	CBYP4600.g1	GE281802
61925758	CBYP4601.b1	GE281803
61925759	CBYP4601.g1	GE281804
61925760	CBYP4602.b1	GE281805
61925761	CBYP4602.g1	GE281806
61925762	CBYP4603.b1	GE281807
61925763	CBYP4603.g1	GE281808
61925764	CBYP4604.b1	GE281809
61925765	CBYP4604.g1	GE281810
61925766	CBYP4605.b1	GE281811
61925767	CBYP4605.g1	GE281812
61925768	CBYP4606.b1	GE281813
61925769	CBYP4606.g1	GE281814
61925770	CBYP4607.g1	GE281815
61925771	CBYP4609.b1	GE281816
61925772	CBYP4609.g1	GE281817
61925773	CBYP4610.b1	GE281818
61925774	CBYP4610.g1	GE281819
61925775	CBYP4611.b1	GE281820
61925776	CBYP4611.g1	GE281821
61925777	CBYP4613.b1	GE281822
61925778	CBYP4613.g1	GE281823
61925779	CBYP4614.b1	GE281824
61925780	CBYP4614.g1	GE281825
61925781	CBYP4615.b1	GE281826
61925782	CBYP4615.g1	GE281827
61925783	CBYP4617.b1	GE281828
61925784	CBYP4617.g1	GE281829
61925785	CBYP4618.b1	GE281830
61925786	CBYP4618.g1	GE281831
61925787	CBYP4619.b1	GE281832
61925788	CBYP4619.g1	GE281833
61925789	CBYP4620.b1	GE281834
61925790	CBYP4620.g1	GE281835

61925791	CBYP4621.b1	GE281836
61925792	CBYP4621.g1	GE281837
61925793	CBYP4622.b1	GE281838
61925794	CBYP4622.g1	GE281839
61925795	CBYP4623.b1	GE281840
61925796	CBYP4623.g1	GE281841
61925797	CBYP4624.b1	GE281842
61925798	CBYP4624.g1	GE281843
61925799	CBYP4625.b1	GE281844
61925800	CBYP4625.g1	GE281845
61925801	CBYP4626.b1	GE281846
61925802	CBYP4626.g1	GE281847
61925803	CBYP4627.b1	GE281848
61925804	CBYP4627.g1	GE281849
61925805	CBYP4628.b1	GE281850
61925806	CBYP4628.g1	GE281851
61925807	CBYP4629.b1	GE281852
61925808	CBYP4629.g1	GE281853
61925809	CBYP4630.b1	GE281854
61925810	CBYP4630.g1	GE281855
61925811	CBYP4631.b1	GE281856
61925812	CBYP4631.g1	GE281857
61925813	CBYP4632.b1	GE281858
61925814	CBYP4632.g1	GE281859
61925815	CBYP4633.b1	GE281860
61925816	CBYP4633.g1	GE281861
61925817	CBYP4634.b1	GE281862
61925818	CBYP4634.g1	GE281863
61925819	CBYP4635.b1	GE281864
61925820	CBYP4635.g1	GE281865
61925821	CBYP4636.b1	GE281866
61925822	CBYP4636.g1	GE281867
61925823	CBYP4637.b1	GE281868
61925824	CBYP4637.g1	GE281869
61925825	CBYP4638.b1	GE281870
61925826	CBYP4638.g1	GE281871
61925827	CBYP4639.g1	GE281872
61925828	CBYP4640.b1	GE281873
61925829	CBYP4642.b1	GE281874
61925830	CBYP4642.g1	GE281875
61925831	CBYP4643.b1	GE281876
61925832	CBYP4643.g1	GE281877
61925833	CBYP4644.b1	GE281878
61925834	CBYP4644.g1	GE281879
61925835	CBYP4645.b1	GE281880
61925836	CBYP4645.g1	GE281881
61925837	CBYP4647.b1	GE281882
61925838	CBYP4647.g1	GE281883
61925839	CBYP4648.b1	GE281884
61925840	CBYP4648.g1	GE281885
61925841	CBYP4649.b1	GE281886
61925842	CBYP4649.g1	GE281887
61925843	CBYP4650.g1	GE281888
61925844	CBYP4651.b1	GE281889
61925845	CBYP4651.g1	GE281890
61925846	CBYP4652.b1	GE281891
61925847	CBYP4652.g1	GE281892
61925848	CBYP4653.b1	GE281893
61925849	CBYP4653.g1	GE281894
61925850	CBYP4654.b1	GE281895
61925851	CBYP4654.g1	GE281896
61925852	CBYP4655.b1	GE281897
61925853	CBYP4656.b1	GE281898

61925854	CBYP4656.g1	GE281899
61925855	CBYP4657.b1	GE281900
61925856	CBYP4657.g1	GE281901
61925857	CBYP4658.b1	GE281902
61925858	CBYP4658.g1	GE281903
61925859	CBYP4659.b1	GE281904
61925860	CBYP4659.g1	GE281905
61925861	CBYP4660.b1	GE281906
61925862	CBYP4660.g1	GE281907
61925863	CBYP4661.b1	GE281908
61925864	CBYP4661.g1	GE281909
61925865	CBYP4663.b1	GE281910
61925866	CBYP4663.g1	GE281911
61925867	CBYP4664.b1	GE281912
61925868	CBYP4664.g1	GE281913
61925869	CBYP4665.b1	GE281914
61925870	CBYP4665.g1	GE281915
61925871	CBYP4666.b1	GE281916
61925872	CBYP4666.g1	GE281917
61925873	CBYP4667.b1	GE281918
61925874	CBYP4667.g1	GE281919
61925875	CBYP4669.b1	GE281920
61925876	CBYP4669.g1	GE281921
61925877	CBYP4671.b1	GE281922
61925878	CBYP4671.g1	GE281923
61925879	CBYP4672.b1	GE281924
61925880	CBYP4672.g1	GE281925
61925881	CBYP4673.b1	GE281926
61925882	CBYP4673.g1	GE281927
61925883	CBYP4674.b1	GE281928
61925884	CBYP4674.g1	GE281929
61925885	CBYP4675.b1	GE281930
61925886	CBYP4675.g1	GE281931
61925887	CBYP4676.b1	GE281932
61925888	CBYP4676.g1	GE281933
61925889	CBYP4678.g1	GE281934
61925890	CBYP4680.b1	GE281935
61925891	CBYP4680.g1	GE281936
61925892	CBYP4681.b1	GE281937
61925893	CBYP4681.g1	GE281938
61925894	CBYP4682.b1	GE281939
61925895	CBYP4682.g1	GE281940
61925896	CBYP4683.b1	GE281941
61925897	CBYP4683.g1	GE281942
61925898	CBYP4684.b1	GE281943
61925899	CBYP4684.g1	GE281944
61925900	CBYP4685.g1	GE281945
61925901	CBYP4687.b1	GE281946
61925902	CBYP4687.g1	GE281947
61925903	CBYP4688.b1	GE281948
61925904	CBYP4688.g1	GE281949
61925905	CBYP4689.b1	GE281950
61925906	CBYP4689.g1	GE281951
61925907	CBYP4690.b1	GE281952
61925908	CBYP4690.g1	GE281953
61925909	CBYP4691.b1	GE281954
61925910	CBYP4691.g1	GE281955
61925911	CBYP4692.b1	GE281956
61925912	CBYP4692.g1	GE281957
61925913	CBYP4693.b1	GE281958
61925914	CBYP4693.g1	GE281959
61925915	CBYP4695.b1	GE281960
61925916	CBYP4695.g1	GE281961

61925917	CBYP4696.b1	GE281962
61925918	CBYP4696.g1	GE281963
61925919	CBYP4698.b1	GE281964
61925920	CBYP4698.g1	GE281965
61925921	CBYP4699.b1	GE281966
61925922	CBYP4700.b1	GE281967
61925923	CBYP4700.g1	GE281968
61925924	CBYP4701.b1	GE281969
61925925	CBYP4701.g1	GE281970
61925926	CBYP4702.b1	GE281971
61925927	CBYP4702.g1	GE281972
61925928	CBYP4703.b1	GE281973
61925929	CBYP4703.g1	GE281974
61925930	CBYP4704.b1	GE281975
61925931	CBYP4704.g1	GE281976
61925932	CBYP4705.g1	GE281977
61925933	CBYP4706.b1	GE281978
61925934	CBYP4706.g1	GE281979
61925935	CBYP4707.b1	GE281980
61925936	CBYP4707.g1	GE281981
61925937	CBYP4708.b1	GE281982
61925938	CBYP4708.g1	GE281983
61925939	CBYP4709.b1	GE281984
61925940	CBYP4709.g1	GE281985
61925941	CBYP4711.b1	GE281986
61925942	CBYP4711.g1	GE281987
61925943	CBYP4712.b1	GE281988
61925944	CBYP4712.g1	GE281989
61925945	CBYP4713.b1	GE281990
61925946	CBYP4713.g1	GE281991
61925947	CBYP4714.b1	GE281992
61925948	CBYP4714.g1	GE281993
61925949	CBYP4715.b1	GE281994
61925950	CBYP4715.g1	GE281995
61925951	CBYP4716.b1	GE281996
61925952	CBYP4716.g1	GE281997
61925953	CBYP4717.b1	GE281998
61925954	CBYP4717.g1	GE281999
61925955	CBYP4718.b1	GE282000
61925956	CBYP4718.g1	GE282001
61925957	CBYP4719.b1	GE282002
61925958	CBYP4720.b1	GE282003
61925959	CBYP4720.g1	GE282004
61925960	CBYP4721.g1	GE282005
61925961	CBYP4722.b1	GE282006
61925962	CBYP4722.g1	GE282007
61925963	CBYP4723.b1	GE282008
61925964	CBYP4723.g1	GE282009
61925965	CBYP4724.b1	GE282010
61925966	CBYP4724.g1	GE282011
61925967	CBYP4725.b1	GE282012
61925968	CBYP4725.g1	GE282013
61925969	CBYP4727.b1	GE282014
61925970	CBYP4727.g1	GE282015
61925971	CBYP4728.b1	GE282016
61925972	CBYP4729.b1	GE282017
61925973	CBYP4730.b1	GE282018
61925974	CBYP4730.g1	GE282019
61925975	CBYP4731.b1	GE282020
61925976	CBYP4731.g1	GE282021
61925977	CBYP4732.b1	GE282022
61925978	CBYP4732.g1	GE282023
61925979	CBYP4733.b1	GE282024

61925980	CBYP4733.g1	GE282025
61925981	CBYP4734.b1	GE282026
61925982	CBYP4734.g1	GE282027
61925983	CBYP4735.b1	GE282028
61925984	CBYP4735.g1	GE282029
61925985	CBYP4736.b1	GE282030
61925986	CBYP4736.g1	GE282031
61925987	CBYP4737.b1	GE282032
61925988	CBYP4737.g1	GE282033
61925989	CBYP4738.b1	GE282034
61925990	CBYP4738.g1	GE282035
61925991	CBYP4739.b1	GE282036
61925992	CBYP4740.b1	GE282037
61925993	CBYP4740.g1	GE282038
61925994	CBYP4741.b1	GE282039
61925995	CBYP4741.g1	GE282040
61925996	CBYP4742.b1	GE282041
61925997	CBYP4742.g1	GE282042
61925998	CBYP4743.b1	GE282043
61925999	CBYP4743.g1	GE282044
61926000	CBYP4745.b1	GE282045
61926001	CBYP4745.g1	GE282046
61926002	CBYP4746.b1	GE282047
61926003	CBYP4746.g1	GE282048
61926004	CBYP4748.b1	GE282049
61926005	CBYP4748.g1	GE282050
61926006	CBYP4749.b1	GE282051
61926007	CBYP4749.g1	GE282052
61926008	CBYP4750.b1	GE282053
61926009	CBYP4750.g1	GE282054
61926010	CBYP4751.b1	GE282055
61926011	CBYP4751.g1	GE282056
61926012	CBYP4752.b1	GE282057
61926013	CBYP4752.g1	GE282058
61926014	CBYP4753.g1	GE282059
61926015	CBYP4754.b1	GE282060
61926016	CBYP4755.b1	GE282061
61926017	CBYP4755.g1	GE282062
61926018	CBYP4757.b1	GE282063
61926019	CBYP4757.g1	GE282064
61926020	CBYP4759.b1	GE282065
61926021	CBYP4759.g1	GE282066
61926022	CBYP4763.g1	GE282067
61926023	CBYP4764.b1	GE282068
61926024	CBYP4764.g1	GE282069
61926025	CBYP4765.b1	GE282070
61926026	CBYP4765.g1	GE282071
61926027	CBYP4766.b1	GE282072
61926028	CBYP4766.g1	GE282073
61926029	CBYP4767.b1	GE282074
61926030	CBYP4767.g1	GE282075
61926031	CBYP4768.b1	GE282076
61926032	CBYP4768.g1	GE282077
61926033	CBYP4769.b1	GE282078
61926034	CBYP4769.g1	GE282079
61926035	CBYP4770.b1	GE282080
61926036	CBYP4770.g1	GE282081
61926037	CBYP4772.b1	GE282082
61926038	CBYP4772.g1	GE282083
61926039	CBYP4773.b1	GE282084
61926040	CBYP4773.g1	GE282085
61926041	CBYP4774.b1	GE282086
61926042	CBYP4774.g1	GE282087

61926043	CBYP4775.b1	GE282088
61926044	CBYP4775.g1	GE282089
61926045	CBYP4776.b1	GE282090
61926046	CBYP4776.g1	GE282091
61926047	CBYP4777.b1	GE282092
61926048	CBYP4777.g1	GE282093
61926049	CBYP4778.b1	GE282094
61926050	CBYP4778.g1	GE282095
61926051	CBYP4779.b1	GE282096
61926052	CBYP4779.g1	GE282097
61926053	CBYP4780.b1	GE282098
61926054	CBYP4780.g1	GE282099
61926055	CBYP4781.b1	GE282100
61926056	CBYP4781.g1	GE282101
61926057	CBYP4782.b1	GE282102
61926058	CBYP4782.g1	GE282103
61926059	CBYP4783.b1	GE282104
61926060	CBYP4783.g1	GE282105
61926061	CBYP4784.b1	GE282106
61926062	CBYP4784.g1	GE282107
61926063	CBYP4785.g1	GE282108
61926064	CBYP4786.b1	GE282109
61926065	CBYP4786.g1	GE282110
61926066	CBYP4787.b1	GE282111
61926067	CBYP4787.g1	GE282112
61926068	CBYP4788.b1	GE282113
61926069	CBYP4788.g1	GE282114
61926070	CBYP4789.b1	GE282115
61926071	CBYP4789.g1	GE282116
61926072	CBYP4790.b1	GE282117
61926073	CBYP4790.g1	GE282118
61926074	CBYP4791.b1	GE282119
61926075	CBYP4791.g1	GE282120
61926076	CBYP4792.b1	GE282121
61926077	CBYP4792.g1	GE282122
61926078	CBYP4793.g1	GE282123
61926079	CBYP4794.b1	GE282124
61926080	CBYP4794.g1	GE282125
61926081	CBYP4795.b1	GE282126
61926082	CBYP4795.g1	GE282127
61926083	CBYP4796.b1	GE282128
61926084	CBYP4796.g1	GE282129
61926085	CBYP4797.b1	GE282130
61926086	CBYP4797.g1	GE282131
61926087	CBYP4798.b1	GE282132
61926088	CBYP4798.g1	GE282133
61926089	CBYP4799.g1	GE282134
61926090	CBYP4800.b1	GE282135
61926091	CBYP4800.g1	GE282136
61926092	CBYP4802.b1	GE282137
61926093	CBYP4802.g1	GE282138
61926094	CBYP4803.b1	GE282139
61926095	CBYP4803.g1	GE282140
61926096	CBYP4804.b1	GE282141
61926097	CBYP4804.g1	GE282142
61926098	CBYP4805.b1	GE282143
61926099	CBYP4805.g1	GE282144
61926100	CBYP4807.b1	GE282145
61926101	CBYP4807.g1	GE282146
61926102	CBYP4808.b1	GE282147
61926103	CBYP4808.g1	GE282148
61926104	CBYP4809.b1	GE282149
61926105	CBYP4809.g1	GE282150

61926106	CBYP4810.b1	GE282151
61926107	CBYP4810.g1	GE282152
61926108	CBYP4811.b1	GE282153
61926109	CBYP4811.g1	GE282154
61926110	CBYP4812.b1	GE282155
61926111	CBYP4812.g1	GE282156
61926112	CBYP4813.b1	GE282157
61926113	CBYP4813.g1	GE282158
61926114	CBYP4814.b1	GE282159
61926115	CBYP4814.g1	GE282160
61926116	CBYP4815.b1	GE282161
61926117	CBYP4815.g1	GE282162
61926118	CBYP4816.b1	GE282163
61926119	CBYP4816.g1	GE282164
61926120	CBYP4817.b1	GE282165
61926121	CBYP4817.g1	GE282166
61926122	CBYP4818.b1	GE282167
61926123	CBYP4818.g1	GE282168
61926124	CBYP4820.b1	GE282169
61926125	CBYP4820.g1	GE282170
61926126	CBYP4821.b1	GE282171
61926127	CBYP4821.g1	GE282172
61926128	CBYP4822.b1	GE282173
61926129	CBYP4822.g1	GE282174
61926130	CBYP4823.b1	GE282175
61926131	CBYP4823.g1	GE282176
61926132	CBYP4824.b1	GE282177
61926133	CBYP4824.g1	GE282178
61926134	CBYP4825.b1	GE282179
61926135	CBYP4825.g1	GE282180
61926136	CBYP4826.b1	GE282181
61926137	CBYP4826.g1	GE282182
61926138	CBYP4827.b1	GE282183
61926139	CBYP4827.g1	GE282184
61926140	CBYP4828.b1	GE282185
61926141	CBYP4828.g1	GE282186
61926142	CBYP4829.g1	GE282187
61926143	CBYP4830.b1	GE282188
61926144	CBYP4830.g1	GE282189
61926145	CBYP4831.b1	GE282190
61926146	CBYP4831.g1	GE282191
61926147	CBYP4832.b1	GE282192
61926148	CBYP4832.g1	GE282193
61926149	CBYP4833.b1	GE282194
61926150	CBYP4833.g1	GE282195
61926151	CBYP4834.b1	GE282196
61926152	CBYP4834.g1	GE282197
61926153	CBYP4835.b1	GE282198
61926154	CBYP4835.g1	GE282199
61926155	CBYP4836.b1	GE282200
61926156	CBYP4836.g1	GE282201
61926157	CBYP4838.b1	GE282202
61926158	CBYP4838.g1	GE282203
61926159	CBYP4839.b1	GE282204
61926160	CBYP4839.g1	GE282205
61926161	CBYP4840.b1	GE282206
61926162	CBYP4840.g1	GE282207
61926163	CBYP4841.b1	GE282208
61926164	CBYP4841.g1	GE282209
61926165	CBYP4842.b1	GE282210
61926166	CBYP4842.g1	GE282211
61926167	CBYP4843.b1	GE282212
61926168	CBYP4843.g1	GE282213

61926169	CBYP4844.b1	GE282214
61926170	CBYP4844.g1	GE282215
61926171	CBYP4845.b1	GE282216
61926172	CBYP4845.g1	GE282217
61926173	CBYP4846.b1	GE282218
61926174	CBYP4846.g1	GE282219
61926175	CBYP4847.b1	GE282220
61926176	CBYP4847.g1	GE282221
61926177	CBYP4849.b1	GE282222
61926178	CBYP4849.g1	GE282223
61926179	CBYP4850.b1	GE282224
61926180	CBYP4850.g1	GE282225
61926181	CBYP4851.b1	GE282226
61926182	CBYP4851.g1	GE282227
61926183	CBYP4852.b1	GE282228
61926184	CBYP4852.g1	GE282229
61926185	CBYP4853.b1	GE282230
61926186	CBYP4853.g1	GE282231
61926187	CBYP4855.b1	GE282232
61926188	CBYP4855.g1	GE282233
61926189	CBYP4856.b1	GE282234
61926190	CBYP4856.g1	GE282235
61926191	CBYP4857.b1	GE282236
61926192	CBYP4857.g1	GE282237
61926193	CBYP4858.b1	GE282238
61926194	CBYP4858.g1	GE282239
61926195	CBYP4859.g1	GE282240
61926196	CBYP4860.b1	GE282241
61926197	CBYP4860.g1	GE282242
61926198	CBYP4861.b1	GE282243
61926199	CBYP4861.g1	GE282244
61926200	CBYP4862.b1	GE282245
61926201	CBYP4862.g1	GE282246
61926202	CBYP4863.b1	GE282247
61926203	CBYP4863.g1	GE282248
61926204	CBYP4864.b1	GE282249
61926205	CBYP4864.g1	GE282250
61926206	CBYP4865.b1	GE282251
61926207	CBYP4865.g1	GE282252
61926208	CBYP4866.b1	GE282253
61926209	CBYP4866.g1	GE282254
61926210	CBYP4867.b1	GE282255
61926211	CBYP4867.g1	GE282256
61926212	CBYP4868.b1	GE282257
61926213	CBYP4868.g1	GE282258
61926214	CBYP4869.b1	GE282259
61926215	CBYP4869.g1	GE282260
61926216	CBYP4870.b1	GE282261
61926217	CBYP4870.g1	GE282262
61926218	CBYP4871.b1	GE282263
61926219	CBYP4871.g1	GE282264
61926220	CBYP4873.b1	GE282265
61926221	CBYP4873.g1	GE282266
61926222	CBYP4874.b1	GE282267
61926223	CBYP4876.b1	GE282268
61926224	CBYP4876.g1	GE282269
61926225	CBYP4877.b1	GE282270
61926226	CBYP4877.g1	GE282271
61926227	CBYP4878.b1	GE282272
61926228	CBYP4878.g1	GE282273
61926229	CBYP4879.g1	GE282274
61926230	CBYP4880.b1	GE282275
61926231	CBYP4881.b1	GE282276

61926232	CBYP4881.g1	GE282277
61926233	CBYP4882.b1	GE282278
61926234	CBYP4882.g1	GE282279
61926235	CBYP4883.g1	GE282280
61926236	CBYP4884.b1	GE282281
61926237	CBYP4884.g1	GE282282
61926238	CBYP4885.b1	GE282283
61926239	CBYP4885.g1	GE282284
61926240	CBYP4886.b1	GE282285
61926241	CBYP4886.g1	GE282286
61926242	CBYP4887.b1	GE282287
61926243	CBYP4887.g1	GE282288
61926244	CBYP4888.b1	GE282289
61926245	CBYP4888.g1	GE282290
61926246	CBYP4889.g1	GE282291
61926247	CBYP4890.b1	GE282292
61926248	CBYP4890.g1	GE282293
61926249	CBYP4891.b1	GE282294
61926250	CBYP4891.g1	GE282295
61926251	CBYP4892.b1	GE282296
61926252	CBYP4892.g1	GE282297
61926253	CBYP4893.b1	GE282298
61926254	CBYP4893.g1	GE282299
61926255	CBYP4894.b1	GE282300
61926256	CBYP4894.g1	GE282301
61926257	CBYP4895.b1	GE282302
61926258	CBYP4895.g1	GE282303
61926259	CBYP4896.b1	GE282304
61926260	CBYP4897.b1	GE282305
61926261	CBYP4897.g1	GE282306
61926262	CBYP4898.b1	GE282307
61926263	CBYP4898.g1	GE282308
61926264	CBYP4899.b1	GE282309
61926265	CBYP4899.g1	GE282310
61926266	CBYP4900.b1	GE282311
61926267	CBYP4900.g1	GE282312
61926268	CBYP4901.b1	GE282313
61926269	CBYP4901.g1	GE282314
61926270	CBYP4902.b1	GE282315
61926271	CBYP4902.g1	GE282316
61926272	CBYP4903.b1	GE282317
61926273	CBYP4903.g1	GE282318
61926274	CBYP4904.b1	GE282319
61926275	CBYP4904.g1	GE282320
61926276	CBYP4905.b1	GE282321
61926277	CBYP4905.g1	GE282322
61926278	CBYP4906.b1	GE282323
61926279	CBYP4906.g1	GE282324
61926280	CBYP4907.b1	GE282325
61926281	CBYP4907.g1	GE282326
61926282	CBYP4908.b1	GE282327
61926283	CBYP4908.g1	GE282328
61926284	CBYP4909.b1	GE282329
61926285	CBYP4909.g1	GE282330
61926286	CBYP4910.b1	GE282331
61926287	CBYP4910.g1	GE282332
61926288	CBYP4911.b1	GE282333
61926289	CBYP4911.g1	GE282334
61926290	CBYP4912.b1	GE282335
61926291	CBYP4912.g1	GE282336
61926292	CBYP4913.b1	GE282337
61926293	CBYP4914.b1	GE282338
61926294	CBYP4914.g1	GE282339

61926295	CBYP4916.b1	GE282340
61926296	CBYP4917.b1	GE282341
61926297	CBYP4917.g1	GE282342
61926298	CBYP4918.b1	GE282343
61926299	CBYP4918.g1	GE282344
61926300	CBYP4919.b1	GE282345
61926301	CBYP4919.g1	GE282346
61926302	CBYP4920.b1	GE282347
61926303	CBYP4920.g1	GE282348
61926304	CBYP4921.b1	GE282349
61926305	CBYP4921.g1	GE282350
61926306	CBYP4922.b1	GE282351
61926307	CBYP4922.g1	GE282352
61926308	CBYP4923.b1	GE282353
61926309	CBYP4923.g1	GE282354
61926310	CBYP4924.g1	GE282355
61926311	CBYP4925.b1	GE282356
61926312	CBYP4925.g1	GE282357
61926313	CBYP4926.b1	GE282358
61926314	CBYP4926.g1	GE282359
61926315	CBYP4927.b1	GE282360
61926316	CBYP4927.g1	GE282361
61926317	CBYP4928.b1	GE282362
61926318	CBYP4928.g1	GE282363
61926319	CBYP4929.b1	GE282364
61926320	CBYP4929.g1	GE282365
61926321	CBYP4930.b1	GE282366
61926322	CBYP4930.g1	GE282367
61926323	CBYP4931.b1	GE282368
61926324	CBYP4931.g1	GE282369
61926325	CBYP4932.b1	GE282370
61926326	CBYP4932.g1	GE282371
61926327	CBYP4933.b1	GE282372
61926328	CBYP4933.g1	GE282373
61926329	CBYP4934.b1	GE282374
61926330	CBYP4934.g1	GE282375
61926331	CBYP4935.b1	GE282376
61926332	CBYP4935.g1	GE282377
61926333	CBYP4936.b1	GE282378
61926334	CBYP4938.b1	GE282379
61926335	CBYP4938.g1	GE282380
61926336	CBYP4939.b1	GE282381
61926337	CBYP4939.g1	GE282382
61926338	CBYP4940.b1	GE282383
61926339	CBYP4940.g1	GE282384
61926340	CBYP4941.g1	GE282385
61926341	CBYP4942.g1	GE282386
61926342	CBYP4943.b1	GE282387
61926343	CBYP4943.g1	GE282388
61926344	CBYP4944.b1	GE282389
61926345	CBYP4944.g1	GE282390
61926346	CBYP4946.b1	GE282391
61926347	CBYP4947.b1	GE282392
61926348	CBYP4947.g1	GE282393
61926349	CBYP4948.b1	GE282394
61926350	CBYP4948.g1	GE282395
61926351	CBYP4949.g1	GE282396
61926352	CBYP4950.b1	GE282397
61926353	CBYP4950.g1	GE282398
61926354	CBYP4951.b1	GE282399
61926355	CBYP4951.g1	GE282400
61926356	CBYP4952.b1	GE282401
61926357	CBYP4952.g1	GE282402

61926358	CBYP4953.b1	GE282403
61926359	CBYP4953.g1	GE282404
61926360	CBYP4954.b1	GE282405
61926361	CBYP4956.b1	GE282406
61926362	CBYP4956.g1	GE282407
61926363	CBYP4957.b1	GE282408
61926364	CBYP4957.g1	GE282409
61926365	CBYP4958.b1	GE282410
61926366	CBYP4958.g1	GE282411
61926367	CBYP4959.b1	GE282412
61926368	CBYP4959.g1	GE282413
61926369	CBYP4960.b1	GE282414
61926370	CBYP4960.g1	GE282415
61926371	CBYP4961.b1	GE282416
61926372	CBYP4961.g1	GE282417
61926373	CBYP4962.b1	GE282418
61926374	CBYP4962.g1	GE282419
61926375	CBYP4963.b1	GE282420
61926376	CBYP4963.g1	GE282421
61926377	CBYP4964.b1	GE282422
61926378	CBYP4964.g1	GE282423
61926379	CBYP4965.b1	GE282424
61926380	CBYP4965.g1	GE282425
61926381	CBYP4966.b1	GE282426
61926382	CBYP4966.g1	GE282427
61926383	CBYP4967.b1	GE282428
61926384	CBYP4967.g1	GE282429
61926385	CBYP4968.b1	GE282430
61926386	CBYP4968.g1	GE282431
61926387	CBYP4969.b1	GE282432
61926388	CBYP4969.g1	GE282433
61926389	CBYP4970.b1	GE282434
61926390	CBYP4970.g1	GE282435
61926391	CBYP4971.b1	GE282436
61926392	CBYP4971.g1	GE282437
61926393	CBYP4972.b1	GE282438
61926394	CBYP4972.g1	GE282439
61926395	CBYP4973.b1	GE282440
61926396	CBYP4973.g1	GE282441
61926397	CBYP4974.b1	GE282442
61926398	CBYP4975.b1	GE282443
61926399	CBYP4975.g1	GE282444
61926400	CBYP4976.b1	GE282445
61926401	CBYP4976.g1	GE282446
61926402	CBYP4977.b1	GE282447
61926403	CBYP4977.g1	GE282448
61926404	CBYP4978.b1	GE282449
61926405	CBYP4978.g1	GE282450
61926406	CBYP4979.b1	GE282451
61926407	CBYP4979.g1	GE282452
61926408	CBYP4980.b1	GE282453
61926409	CBYP4980.g1	GE282454
61926410	CBYP4981.b1	GE282455
61926411	CBYP4981.g1	GE282456
61926412	CBYP4982.b1	GE282457
61926413	CBYP4982.g1	GE282458
61926414	CBYP4983.b1	GE282459
61926415	CBYP4983.g1	GE282460
61926416	CBYP4984.g1	GE282461
61926417	CBYP4985.b1	GE282462
61926418	CBYP4985.g1	GE282463
61926419	CBYP4986.b1	GE282464
61926420	CBYP4986.g1	GE282465

61926421	CBYP4987.b1	GE282466
61926422	CBYP4987.g1	GE282467
61926423	CBYP4988.b1	GE282468
61926424	CBYP4988.g1	GE282469
61926425	CBYP4989.b1	GE282470
61926426	CBYP4989.g1	GE282471
61926427	CBYP4990.b1	GE282472
61926428	CBYP4990.g1	GE282473
61926429	CBYP4991.b1	GE282474
61926430	CBYP4991.g1	GE282475
61926431	CBYP4993.b1	GE282476
61926432	CBYP4993.g1	GE282477
61926433	CBYP4994.b1	GE282478
61926434	CBYP4994.g1	GE282479
61926435	CBYP4995.b1	GE282480
61926436	CBYP4995.g1	GE282481
61926437	CBYP4996.b1	GE282482
61926438	CBYP4996.g1	GE282483
61926439	CBYP4997.b1	GE282484
61926440	CBYP4997.g1	GE282485
61926441	CBYP4998.b1	GE282486
61926442	CBYP4998.g1	GE282487
61926443	CBYP5000.b1	GE282488
61926444	CBYP5000.g1	GE282489
61926445	CBYP5001.b1	GE282490
61926446	CBYP5001.g1	GE282491
61926447	CBYP5002.b1	GE282492
61926448	CBYP5002.g1	GE282493
61926449	CBYP5003.b1	GE282494
61926450	CBYP5003.g1	GE282495
61926451	CBYP5004.b1	GE282496
61926452	CBYP5004.g1	GE282497
61926453	CBYP5005.b1	GE282498
61926454	CBYP5005.g1	GE282499
61926455	CBYP5006.b1	GE282500
61926456	CBYP5006.g1	GE282501
61926457	CBYP5007.b1	GE282502
61926458	CBYP5007.g1	GE282503
61926459	CBYP5008.b1	GE282504
61926460	CBYP5008.g1	GE282505
61926461	CBYP5009.b1	GE282506
61926462	CBYP5009.g1	GE282507
61926463	CBYP5010.b1	GE282508
61926464	CBYP5010.g1	GE282509
61926465	CBYP5011.b1	GE282510
61926466	CBYP5011.g1	GE282511
61926467	CBYP5012.b1	GE282512
61926468	CBYP5012.g1	GE282513
61926469	CBYP5013.b1	GE282514
61926470	CBYP5013.g1	GE282515
61926471	CBYP5014.b1	GE282516
61926472	CBYP5014.g1	GE282517
61926473	CBYP5015.b1	GE282518
61926474	CBYP5015.g1	GE282519
61926475	CBYP5016.b1	GE282520
61926476	CBYP5017.b1	GE282521
61926477	CBYP5017.g1	GE282522
61926478	CBYP5018.b1	GE282523
61926479	CBYP5018.g1	GE282524
61926480	CBYP5019.b1	GE282525
61926481	CBYP5019.g1	GE282526
61926482	CBYP5020.b1	GE282527
61926483	CBYP5020.g1	GE282528

61926484	CBYP5021.b1	GE282529
61926485	CBYP5021.g1	GE282530
61926486	CBYP5022.b1	GE282531
61926487	CBYP5022.g1	GE282532
61926488	CBYP5023.b1	GE282533
61926489	CBYP5023.g1	GE282534
61926490	CBYP5024.g1	GE282535
61926491	CBYP5025.b1	GE282536
61926492	CBYP5025.g1	GE282537
61926493	CBYP5026.b1	GE282538
61926494	CBYP5026.g1	GE282539
61926495	CBYP5027.b1	GE282540
61926496	CBYP5027.g1	GE282541
61926497	CBYP5028.b1	GE282542
61926498	CBYP5029.b1	GE282543
61926499	CBYP5029.g1	GE282544
61926500	CBYP5030.b1	GE282545
61926501	CBYP5030.g1	GE282546
61926502	CBYP5031.b1	GE282547
61926503	CBYP5031.g1	GE282548
61926504	CBYP5032.b1	GE282549
61926505	CBYP5032.g1	GE282550
61926506	CBYP5033.b1	GE282551
61926507	CBYP5033.g1	GE282552
61926508	CBYP5034.b1	GE282553
61926509	CBYP5034.g1	GE282554
61926510	CBYP5035.b1	GE282555
61926511	CBYP5035.g1	GE282556
61926512	CBYP5036.b1	GE282557
61926513	CBYP5036.g1	GE282558
61926514	CBYP5037.b1	GE282559
61926515	CBYP5037.g1	GE282560
61926516	CBYP5039.b1	GE282561
61926517	CBYP5039.g1	GE282562
61926518	CBYP5040.b1	GE282563
61926519	CBYP5040.g1	GE282564
61926520	CBYP5041.b1	GE282565
61926521	CBYP5041.g1	GE282566
61926522	CBYP5042.b1	GE282567
61926523	CBYP5044.b1	GE282568
61926524	CBYP5044.g1	GE282569
61926525	CBYP5045.b1	GE282570
61926526	CBYP5045.g1	GE282571
61926527	CBYP5046.b1	GE282572
61926528	CBYP5046.g1	GE282573
61926529	CBYP5047.b1	GE282574
61926530	CBYP5047.g1	GE282575
61926531	CBYP5048.b1	GE282576
61926532	CBYP5048.g1	GE282577
61926533	CBYP5049.b1	GE282578
61926534	CBYP5049.g1	GE282579
61926535	CBYP5050.b1	GE282580
61926536	CBYP5050.g1	GE282581
61926537	CBYP5051.b1	GE282582
61926538	CBYP5051.g1	GE282583
61926539	CBYP5052.b1	GE282584
61926540	CBYP5052.g1	GE282585
61926541	CBYP5053.b1	GE282586
61926542	CBYP5053.g1	GE282587
61926543	CBYP5054.b1	GE282588
61926544	CBYP5054.g1	GE282589
61926545	CBYP5055.b1	GE282590
61926546	CBYP5055.g1	GE282591

61926547	CBYP5056.b1	GE282592
61926548	CBYP5056.g1	GE282593
61926549	CBYP5057.b1	GE282594
61926550	CBYP5057.g1	GE282595
61926551	CBYP5058.b1	GE282596
61926552	CBYP5059.b1	GE282597
61926553	CBYP5059.g1	GE282598
61926554	CBYP5060.b1	GE282599
61926555	CBYP5060.g1	GE282600
61926556	CBYP5061.b1	GE282601
61926557	CBYP5061.g1	GE282602
61926558	CBYP5062.b1	GE282603
61926559	CBYP5062.g1	GE282604
61926560	CBYP5063.b1	GE282605
61926561	CBYP5063.g1	GE282606
61926562	CBYP5064.b1	GE282607
61926563	CBYP5064.g1	GE282608
61926564	CBYP5065.b1	GE282609
61926565	CBYP5065.g1	GE282610
61926566	CBYP5066.b1	GE282611
61926567	CBYP5066.g1	GE282612
61926568	CBYP5067.b1	GE282613
61926569	CBYP5067.g1	GE282614
61926570	CBYP5068.b1	GE282615
61926571	CBYP5068.g1	GE282616
61926572	CBYP5069.b1	GE282617
61926573	CBYP5069.g1	GE282618
61926574	CBYP5070.b1	GE282619
61926575	CBYP5070.g1	GE282620
61926576	CBYP5071.b1	GE282621
61926577	CBYP5071.g1	GE282622
61926578	CBYP5072.b1	GE282623
61926579	CBYP5072.g1	GE282624
61926580	CBYP5073.b1	GE282625
61926581	CBYP5073.g1	GE282626
61926582	CBYP5074.b1	GE282627
61926583	CBYP5074.g1	GE282628
61926584	CBYP5075.b1	GE282629
61926585	CBYP5075.g1	GE282630
61926586	CBYP5076.g1	GE282631
61926587	CBYP5077.b1	GE282632
61926588	CBYP5077.g1	GE282633
61926589	CBYP5078.b1	GE282634
61926590	CBYP5078.g1	GE282635
61926591	CBYP5079.b1	GE282636
61926592	CBYP5079.g1	GE282637
61926593	CBYP5080.b1	GE282638
61926594	CBYP5080.g1	GE282639
61926595	CBYP5081.b1	GE282640
61926596	CBYP5081.g1	GE282641
61926597	CBYP5082.b1	GE282642
61926598	CBYP5082.g1	GE282643
61926599	CBYP5083.b1	GE282644
61926600	CBYP5083.g1	GE282645
61926601	CBYP5084.b1	GE282646
61926602	CBYP5085.b1	GE282647
61926603	CBYP5085.g1	GE282648
61926604	CBYP5086.b1	GE282649
61926605	CBYP5086.g1	GE282650
61926606	CBYP5087.b1	GE282651
61926607	CBYP5087.g1	GE282652
61926608	CBYP5089.b1	GE282653
61926609	CBYP5089.g1	GE282654

61926610	CBYP5090.b1	GE282655
61926611	CBYP5090.g1	GE282656
61926612	CBYP5091.b1	GE282657
61926613	CBYP5091.g1	GE282658
61926614	CBYP5092.b1	GE282659
61926615	CBYP5092.g1	GE282660
61926616	CBYP5093.b1	GE282661
61926617	CBYP5093.g1	GE282662
61926618	CBYP5094.b1	GE282663
61926619	CBYP5094.g1	GE282664
61926620	CBYP5095.b1	GE282665
61926621	CBYP5095.g1	GE282666
61926622	CBYP5096.b1	GE282667
61926623	CBYP5097.b1	GE282668
61926624	CBYP5097.g1	GE282669
61926625	CBYP5098.b1	GE282670
61926626	CBYP5099.b1	GE282671
61926627	CBYP5099.g1	GE282672
61926628	CBYP5100.b1	GE282673
61926629	CBYP5100.g1	GE282674
61926630	CBYP5101.b1	GE282675
61926631	CBYP5101.g1	GE282676
61926632	CBYP5102.b1	GE282677
61926633	CBYP5102.g1	GE282678
61926634	CBYP5104.b1	GE282679
61926635	CBYP5104.g1	GE282680
61926636	CBYP5105.b1	GE282681
61926637	CBYP5106.b1	GE282682
61926638	CBYP5106.g1	GE282683
61926639	CBYP5107.b1	GE282684
61926640	CBYP5107.g1	GE282685
61926641	CBYP5109.b1	GE282686
61926642	CBYP5110.b1	GE282687
61926643	CBYP5110.g1	GE282688
61926644	CBYP5112.b1	GE282689
61926645	CBYP5112.g1	GE282690
61926646	CBYP5113.b1	GE282691
61926647	CBYP5113.g1	GE282692
61926648	CBYP5114.b1	GE282693
61926649	CBYP5114.g1	GE282694
61926650	CBYP5115.b1	GE282695
61926651	CBYP5115.g1	GE282696
61926652	CBYP5117.b1	GE282697
61926653	CBYP5117.g1	GE282698
61926654	CBYP5118.g1	GE282699
61926655	CBYP5119.b1	GE282700
61926656	CBYP5119.g1	GE282701
61926657	CBYP5120.b1	GE282702
61926658	CBYP5120.g1	GE282703
61926659	CBYP5121.b1	GE282704
61926660	CBYP5121.g1	GE282705
61926661	CBYP5122.b1	GE282706
61926662	CBYP5122.g1	GE282707
61926663	CBYP5123.b1	GE282708
61926664	CBYP5123.g1	GE282709
61926665	CBYP5124.b1	GE282710
61926666	CBYP5126.b1	GE282711
61926667	CBYP5126.g1	GE282712
61926668	CBYP5127.b1	GE282713
61926669	CBYP5127.g1	GE282714
61926670	CBYP5128.b1	GE282715
61926671	CBYP5128.g1	GE282716
61926672	CBYP5129.b1	GE282717

61926673	CBYP5129.g1	GE282718
61926674	CBYP5130.b1	GE282719
61926675	CBYP5130.g1	GE282720
61926676	CBYP5133.b1	GE282721
61926677	CBYP5133.g1	GE282722
61926678	CBYP5134.b1	GE282723
61926679	CBYP5134.g1	GE282724
61926680	CBYP5135.b1	GE282725
61926681	CBYP5135.g1	GE282726
61926682	CBYP5136.b1	GE282727
61926683	CBYP5136.g1	GE282728
61926684	CBYP5137.b1	GE282729
61926685	CBYP5137.g1	GE282730
61926686	CBYP5138.b1	GE282731
61926687	CBYP5138.g1	GE282732
61926688	CBYP5139.b1	GE282733
61926689	CBYP5139.g1	GE282734
61926690	CBYP5140.b1	GE282735
61926691	CBYP5140.g1	GE282736
61926692	CBYP5141.b1	GE282737
61926693	CBYP5141.g1	GE282738
61926694	CBYP5142.g1	GE282739
61926695	CBYP5143.b1	GE282740
61926696	CBYP5143.g1	GE282741
61926697	CBYP5144.b1	GE282742
61926698	CBYP5144.g1	GE282743
61926699	CBYP5145.b1	GE282744
61926700	CBYP5145.g1	GE282745
61926701	CBYP5146.b1	GE282746
61926702	CBYP5146.g1	GE282747
61926703	CBYP5148.b1	GE282748
61926704	CBYP5148.g1	GE282749
61926705	CBYP5149.b1	GE282750
61926706	CBYP5149.g1	GE282751
61926707	CBYP5150.b1	GE282752
61926708	CBYP5150.g1	GE282753
61926709	CBYP5151.b1	GE282754
61926710	CBYP5151.g1	GE282755
61926711	CBYP5152.b1	GE282756
61926712	CBYP5152.g1	GE282757
61926713	CBYP5153.b1	GE282758
61926714	CBYP5153.g1	GE282759
61926715	CBYP5154.b1	GE282760
61926716	CBYP5154.g1	GE282761
61926717	CBYP5155.b1	GE282762
61926718	CBYP5155.g1	GE282763
61926719	CBYP5156.b1	GE282764
61926720	CBYP5156.g1	GE282765
61926721	CBYP5157.b1	GE282766
61926722	CBYP5157.g1	GE282767
61926723	CBYP5158.b1	GE282768
61926724	CBYP5158.g1	GE282769
61926725	CBYP5159.b1	GE282770
61926726	CBYP5159.g1	GE282771
61926727	CBYP5160.g1	GE282772
61926728	CBYP5161.b1	GE282773
61926729	CBYP5161.g1	GE282774
61926730	CBYP5162.b1	GE282775
61926731	CBYP5162.g1	GE282776
61926732	CBYP5163.b1	GE282777
61926733	CBYP5163.g1	GE282778
61926734	CBYP5164.b1	GE282779
61926735	CBYP5164.g1	GE282780

61926736	CBYP5165.b1	GE282781
61926737	CBYP5165.g1	GE282782
61926738	CBYP5166.b1	GE282783
61926739	CBYP5166.g1	GE282784
61926740	CBYP5167.b1	GE282785
61926741	CBYP5167.g1	GE282786
61926742	CBYP5168.b1	GE282787
61926743	CBYP5168.g1	GE282788
61926744	CBYP5169.b1	GE282789
61926745	CBYP5169.g1	GE282790
61926746	CBYP5170.b1	GE282791
61926747	CBYP5170.g1	GE282792
61926748	CBYP5171.b1	GE282793
61926749	CBYP5171.g1	GE282794
61926750	CBYP5172.b1	GE282795
61926751	CBYP5172.g1	GE282796
61926752	CBYP5173.b1	GE282797
61926753	CBYP5173.g1	GE282798
61926754	CBYP5174.b1	GE282799
61926755	CBYP5174.g1	GE282800
61926756	CBYP5176.b1	GE282801
61926757	CBYP5176.g1	GE282802
61926758	CBYP5177.b1	GE282803
61926759	CBYP5178.b1	GE282804
61926760	CBYP5178.g1	GE282805
61926761	CBYP5179.b1	GE282806
61926762	CBYP5179.g1	GE282807
61926763	CBYP5180.b1	GE282808
61926764	CBYP5180.g1	GE282809
61926765	CBYP5181.b1	GE282810
61926766	CBYP5181.g1	GE282811
61926767	CBYP5182.b1	GE282812
61926768	CBYP5182.g1	GE282813
61926769	CBYP5183.b1	GE282814
61926770	CBYP5183.g1	GE282815
61926771	CBYP5184.b1	GE282816
61926772	CBYP5184.g1	GE282817
61926773	CBYP5185.b1	GE282818
61926774	CBYP5185.g1	GE282819
61926775	CBYP5186.b1	GE282820
61926776	CBYP5187.b1	GE282821
61926777	CBYP5187.g1	GE282822
61926778	CBYP5188.b1	GE282823
61926779	CBYP5188.g1	GE282824
61926780	CBYP5189.b1	GE282825
61926781	CBYP5189.g1	GE282826
61926782	CBYP5190.b1	GE282827
61926783	CBYP5190.g1	GE282828
61926784	CBYP5192.b1	GE282829
61926785	CBYP5192.g1	GE282830
61926786	CBYP5193.g1	GE282831
61926787	CBYP5194.b1	GE282832
61926788	CBYP5194.g1	GE282833
61926789	CBYP5195.b1	GE282834
61926790	CBYP5195.g1	GE282835
61926791	CBYP5196.b1	GE282836
61926792	CBYP5196.g1	GE282837
61926793	CBYP5197.b1	GE282838
61926794	CBYP5197.g1	GE282839
61926795	CBYP5198.b1	GE282840
61926796	CBYP5198.g1	GE282841
61926797	CBYP5199.b1	GE282842
61926798	CBYP5199.g1	GE282843

61926799	CBYP5200.b1	GE282844
61926800	CBYP5200.g1	GE282845
61926801	CBYP5201.b1	GE282846
61926802	CBYP5201.g1	GE282847
61926803	CBYP5202.b1	GE282848
61926804	CBYP5202.g1	GE282849
61926805	CBYP5203.b1	GE282850
61926806	CBYP5203.g1	GE282851
61926807	CBYP5204.b1	GE282852
61926808	CBYP5204.g1	GE282853
61926809	CBYP5205.b1	GE282854
61926810	CBYP5205.g1	GE282855
61926811	CBYP5206.b1	GE282856
61926812	CBYP5206.g1	GE282857
61926813	CBYP5208.b1	GE282858
61926814	CBYP5208.g1	GE282859
61926815	CBYP5209.b1	GE282860
61926816	CBYP5209.g1	GE282861
61926817	CBYP5210.b1	GE282862
61926818	CBYP5210.g1	GE282863
61926819	CBYP5211.b1	GE282864
61926820	CBYP5211.g1	GE282865
61926821	CBYP5212.b1	GE282866
61926822	CBYP5212.g1	GE282867
61926823	CBYP5213.b1	GE282868
61926824	CBYP5213.g1	GE282869
61926825	CBYP5214.b1	GE282870
61926826	CBYP5214.g1	GE282871
61926827	CBYP5215.b1	GE282872
61926828	CBYP5216.b1	GE282873
61926829	CBYP5216.g1	GE282874
61926830	CBYP5217.b1	GE282875
61926831	CBYP5217.g1	GE282876
61926832	CBYP5218.b1	GE282877
61926833	CBYP5218.g1	GE282878
61926834	CBYP5219.b1	GE282879
61926835	CBYP5219.g1	GE282880
61926836	CBYP5221.b1	GE282881
61926837	CBYP5222.b1	GE282882
61926838	CBYP5222.g1	GE282883
61926839	CBYP5223.b1	GE282884
61926840	CBYP5223.g1	GE282885
61926841	CBYP5224.b1	GE282886
61926842	CBYP5224.g1	GE282887
61926843	CBYP5225.b1	GE282888
61926844	CBYP5225.g1	GE282889
61926845	CBYP5226.b1	GE282890
61926846	CBYP5226.g1	GE282891
61926847	CBYP5227.b1	GE282892
61926848	CBYP5227.g1	GE282893
61926849	CBYP5228.b1	GE282894
61926850	CBYP5228.g1	GE282895
61926851	CBYP5229.b1	GE282896
61926852	CBYP5229.g1	GE282897
61926853	CBYP5231.b1	GE282898
61926854	CBYP5231.g1	GE282899
61926855	CBYP5232.b1	GE282900
61926856	CBYP5232.g1	GE282901
61926857	CBYP5233.b1	GE282902
61926858	CBYP5233.g1	GE282903
61926859	CBYP5234.b1	GE282904
61926860	CBYP5234.g1	GE282905
61926861	CBYP5235.b1	GE282906

61926862	CBYP5235.g1	GE282907
61926863	CBYP5236.b1	GE282908
61926864	CBYP5236.g1	GE282909
61926865	CBYP5237.b1	GE282910
61926866	CBYP5237.g1	GE282911
61926867	CBYP5238.b1	GE282912
61926868	CBYP5238.g1	GE282913
61926869	CBYP5239.b1	GE282914
61926870	CBYP5239.g1	GE282915
61926871	CBYP5240.b1	GE282916
61926872	CBYP5240.g1	GE282917
61926873	CBYP5241.b1	GE282918
61926874	CBYP5241.g1	GE282919
61926875	CBYP5242.b1	GE282920
61926876	CBYP5242.g1	GE282921
61926877	CBYP5244.b1	GE282922
61926878	CBYP5244.g1	GE282923
61926879	CBYP5245.b1	GE282924
61926880	CBYP5245.g1	GE282925
61926881	CBYP5246.b1	GE282926
61926882	CBYP5246.g1	GE282927
61926883	CBYP5247.b1	GE282928
61926884	CBYP5247.g1	GE282929
61926885	CBYP5248.b1	GE282930
61926886	CBYP5248.g1	GE282931
61926887	CBYP5249.b1	GE282932
61926888	CBYP5249.g1	GE282933
61926889	CBYP5250.b1	GE282934
61926890	CBYP5250.g1	GE282935
61926891	CBYP5251.b1	GE282936
61926892	CBYP5251.g1	GE282937
61926893	CBYP5252.b1	GE282938
61926894	CBYP5252.g1	GE282939
61926895	CBYP5253.b1	GE282940
61926896	CBYP5253.g1	GE282941
61926897	CBYP5254.b1	GE282942
61926898	CBYP5254.g1	GE282943
61926899	CBYP5255.b1	GE282944
61926900	CBYP5255.g1	GE282945
61926901	CBYP5256.b1	GE282946
61926902	CBYP5256.g1	GE282947
61926903	CBYP5258.b1	GE282948
61926904	CBYP5258.g1	GE282949
61926905	CBYP5259.b1	GE282950
61926906	CBYP5259.g1	GE282951
61926907	CBYP5260.b1	GE282952
61926908	CBYP5260.g1	GE282953
61926909	CBYP5261.b1	GE282954
61926910	CBYP5261.g1	GE282955
61926911	CBYP5262.b1	GE282956
61926912	CBYP5262.g1	GE282957
61926913	CBYP5263.b1	GE282958
61926914	CBYP5263.g1	GE282959
61926915	CBYP5264.b1	GE282960
61926916	CBYP5264.g1	GE282961
61926917	CBYP5265.b1	GE282962
61926918	CBYP5265.g1	GE282963
61926919	CBYP5266.b1	GE282964
61926920	CBYP5266.g1	GE282965
61926921	CBYP5269.b1	GE282966
61926922	CBYP5269.g1	GE282967
61926923	CBYP5270.b1	GE282968
61926924	CBYP5270.g1	GE282969

61926925	CBYP5271.b1	GE282970
61926926	CBYP5271.g1	GE282971
61926927	CBYP5272.b1	GE282972
61926928	CBYP5272.g1	GE282973
61926929	CBYP5273.b1	GE282974
61926930	CBYP5273.g1	GE282975
61926931	CBYP5274.g1	GE282976
61926932	CBYP5275.b1	GE282977
61926933	CBYP5275.g1	GE282978
61926934	CBYP5276.b1	GE282979
61926935	CBYP5276.g1	GE282980
61926936	CBYP5278.g1	GE282981
61926937	CBYP5279.b1	GE282982
61926938	CBYP5279.g1	GE282983
61926939	CBYP5281.b1	GE282984
61926940	CBYP5281.g1	GE282985
61926941	CBYP5282.b1	GE282986
61926942	CBYP5282.g1	GE282987
61926943	CBYP5283.b1	GE282988
61926944	CBYP5283.g1	GE282989
61926945	CBYP5284.b1	GE282990
61926946	CBYP5284.g1	GE282991
61926947	CBYP5285.b1	GE282992
61926948	CBYP5285.g1	GE282993
61926949	CBYP5286.b1	GE282994
61926950	CBYP5286.g1	GE282995
61926951	CBYP5287.b1	GE282996
61926952	CBYP5287.g1	GE282997
61926953	CBYP5288.b1	GE282998
61926954	CBYP5289.b1	GE282999
61926955	CBYP5289.g1	GE283000
61926956	CBYP5290.b1	GE283001
61926957	CBYP5290.g1	GE283002
61926958	CBYP5291.b1	GE283003
61926959	CBYP5291.g1	GE283004
61926960	CBYP5292.b1	GE283005
61926961	CBYP5292.g1	GE283006
61926962	CBYP5293.b1	GE283007
61926963	CBYP5294.b1	GE283008
61926964	CBYP5294.g1	GE283009
61926965	CBYP5295.b1	GE283010
61926966	CBYP5295.g1	GE283011
61926967	CBYP5296.b1	GE283012
61926968	CBYP5296.g1	GE283013
61926969	CBYP5297.b1	GE283014
61926970	CBYP5298.b1	GE283015
61926971	CBYP5299.b1	GE283016
61926972	CBYP5299.g1	GE283017
61926973	CBYP5300.b1	GE283018
61926974	CBYP5300.g1	GE283019
61926975	CBYP5301.b1	GE283020
61926976	CBYP5301.g1	GE283021
61926977	CBYP5303.b1	GE283022
61926978	CBYP5303.g1	GE283023
61926979	CBYP5304.g1	GE283024
61926980	CBYP5305.b1	GE283025
61926981	CBYP5305.g1	GE283026
61926982	CBYP5306.b1	GE283027
61926983	CBYP5306.g1	GE283028
61926984	CBYP5307.b1	GE283029
61926985	CBYP5307.g1	GE283030
61926986	CBYP5308.b1	GE283031
61926987	CBYP5308.g1	GE283032

61926988	CBYP5309.b1	GE283033
61926989	CBYP5309.g1	GE283034
61926990	CBYP5311.b1	GE283035
61926991	CBYP5311.g1	GE283036
61926992	CBYP5313.b1	GE283037
61926993	CBYP5313.g1	GE283038
61926994	CBYP5314.b1	GE283039
61926995	CBYP5314.g1	GE283040
61926996	CBYP5315.g1	GE283041
61926997	CBYP5316.b1	GE283042
61926998	CBYP5316.g1	GE283043
61926999	CBYP5317.b1	GE283044
61927000	CBYP5317.g1	GE283045
61927001	CBYP5318.b1	GE283046
61927002	CBYP5318.g1	GE283047
61927003	CBYP5319.b1	GE283048
61927004	CBYP5319.g1	GE283049
61927005	CBYP5321.b1	GE283050
61927006	CBYP5321.g1	GE283051
61927007	CBYP5322.b1	GE283052
61927008	CBYP5322.g1	GE283053
61927009	CBYP5323.b1	GE283054
61927010	CBYP5323.g1	GE283055
61927011	CBYP5324.b1	GE283056
61927012	CBYP5324.g1	GE283057
61927013	CBYP5325.b1	GE283058
61927014	CBYP5325.g1	GE283059
61927015	CBYP5326.b1	GE283060
61927016	CBYP5326.g1	GE283061
61927017	CBYP5327.b1	GE283062
61927018	CBYP5327.g1	GE283063
61927019	CBYP5330.b1	GE283064
61927020	CBYP5330.g1	GE283065
61927021	CBYP5331.b1	GE283066
61927022	CBYP5331.g1	GE283067
61927023	CBYP5332.b1	GE283068
61927024	CBYP5332.g1	GE283069
61927025	CBYP5333.b1	GE283070
61927026	CBYP5333.g1	GE283071
61927027	CBYP5334.b1	GE283072
61927028	CBYP5334.g1	GE283073
61927029	CBYP5336.b1	GE283074
61927030	CBYP5336.g1	GE283075
61927031	CBYP5337.b1	GE283076
61927032	CBYP5337.g1	GE283077
61927033	CBYP5338.b1	GE283078
61927034	CBYP5338.g1	GE283079
61927035	CBYP5339.b1	GE283080
61927036	CBYP5339.g1	GE283081
61927037	CBYP5341.b1	GE283082
61927038	CBYP5341.g1	GE283083
61927039	CBYP5342.b1	GE283084
61927040	CBYP5342.g1	GE283085
61927041	CBYP5343.b1	GE283086
61927042	CBYP5343.g1	GE283087
61927043	CBYP5344.b1	GE283088
61927044	CBYP5344.g1	GE283089
61927045	CBYP5345.b1	GE283090
61927046	CBYP5345.g1	GE283091
61927047	CBYP5346.b1	GE283092
61927048	CBYP5346.g1	GE283093
61927049	CBYP5347.b1	GE283094
61927050	CBYP5347.g1	GE283095

61927051	CBYP5348.b1	GE283096
61927052	CBYP5348.g1	GE283097
61927053	CBYP5349.b1	GE283098
61927054	CBYP5349.g1	GE283099
61927055	CBYP5351.b1	GE283100
61927056	CBYP5351.g1	GE283101
61927057	CBYP5352.b1	GE283102
61927058	CBYP5352.g1	GE283103
61927059	CBYP5353.b1	GE283104
61927060	CBYP5353.g1	GE283105
61927061	CBYP5354.b1	GE283106
61927062	CBYP5354.g1	GE283107
61927063	CBYP5355.b1	GE283108
61927064	CBYP5355.g1	GE283109
61927065	CBYP5356.b1	GE283110
61927066	CBYP5356.g1	GE283111
61927067	CBYP5357.b1	GE283112
61927068	CBYP5357.g1	GE283113
61927069	CBYP5358.b1	GE283114
61927070	CBYP5358.g1	GE283115
61927071	CBYP5359.b1	GE283116
61927072	CBYP5359.g1	GE283117
61927073	CBYP5360.b1	GE283118
61927074	CBYP5360.g1	GE283119
61927075	CBYP5361.b1	GE283120
61927076	CBYP5361.g1	GE283121
61927077	CBYP5362.b1	GE283122
61927078	CBYP5362.g1	GE283123
61927079	CBYP5363.b1	GE283124
61927080	CBYP5363.g1	GE283125
61927081	CBYP5364.b1	GE283126
61927082	CBYP5364.g1	GE283127
61927083	CBYP5365.b1	GE283128
61927084	CBYP5365.g1	GE283129
61927085	CBYP5366.b1	GE283130
61927086	CBYP5366.g1	GE283131
61927087	CBYP5367.b1	GE283132
61927088	CBYP5367.g1	GE283133
61927089	CBYP5371.b1	GE283134
61927090	CBYP5371.g1	GE283135
61927091	CBYP5372.b1	GE283136
61927092	CBYP5373.b1	GE283137
61927093	CBYP5373.g1	GE283138
61927094	CBYP5374.b1	GE283139
61927095	CBYP5374.g1	GE283140
61927096	CBYP550.b1	GE283141
61927097	CBYP769.g1	GE283142
61927098	CBYP770.g1	GE283143
61927099	CBYP771.g1	GE283144
61927100	CBYP772.g1	GE283145
61927101	CBYP773.g1	GE283146
61927102	CBYP774.g1	GE283147
61927103	CBYP776.g1	GE283148
61927104	CBYP777.g1	GE283149
61927105	CBYP778.g1	GE283150
61927106	CBYP779.g1	GE283151
61927107	CBYP780.g1	GE283152
61927108	CBYP781.g1	GE283153
61927109	CBYP782.g1	GE283154
61927110	CBYP783.g1	GE283155
61927111	CBYP784.g1	GE283156
61927112	CBYP785.g1	GE283157
61927113	CBYP786.g1	GE283158

61927114	CBYP787.g1	GE283159
61927115	CBYP788.g1	GE283160
61927116	CBYP789.g1	GE283161
61927117	CBYP790.g1	GE283162
61927118	CBYP791.g1	GE283163
61927119	CBYP792.g1	GE283164
61927120	CBYP793.g1	GE283165
61927121	CBYP794.g1	GE283166
61927122	CBYP795.g1	GE283167
61927123	CBYP796.g1	GE283168
61927124	CBYP797.g1	GE283169
61927125	CBYP799.g1	GE283170
61927126	CBYP800.g1	GE283171
61927127	CBYP801.g1	GE283172
61927128	CBYP802.g1	GE283173
61927129	CBYP803.g1	GE283174
61927130	CBYP804.g1	GE283175
61927131	CBYP805.g1	GE283176
61927132	CBYP806.g1	GE283177
61927133	CBYP807.g1	GE283178
61927134	CBYP808.g1	GE283179
61927135	CBYP809.g1	GE283180
61927136	CBYP810.g1	GE283181
61927137	CBYP811.g1	GE283182
61927138	CBYP812.g1	GE283183
61927139	CBYP813.g1	GE283184
61927140	CBYP814.g1	GE283185
61927141	CBYP815.g1	GE283186
61927142	CBYP817.g1	GE283187
61927143	CBYP818.g1	GE283188
61927144	CBYP819.g1	GE283189
61927145	CBYP820.g1	GE283190
61927146	CBYP821.g1	GE283191
61927147	CBYP822.g1	GE283192
61927148	CBYP823.g1	GE283193
61927149	CBYP824.g1	GE283194
61927150	CBYP825.g1	GE283195
61927151	CBYP826.g1	GE283196
61927152	CBYP827.g1	GE283197
61927153	CBYP828.g1	GE283198
61927154	CBYP829.g1	GE283199
61927155	CBYP830.g1	GE283200
61927156	CBYP832.g1	GE283201
61927157	CBYP833.g1	GE283202
61927158	CBYP835.g1	GE283203
61927159	CBYP836.g1	GE283204
61927160	CBYP837.g1	GE283205
61927161	CBYP838.g1	GE283206
61927162	CBYP839.g1	GE283207
61927163	CBYP840.g1	GE283208
61927164	CBYP841.g1	GE283209
61927165	CBYP842.g1	GE283210
61927166	CBYP843.g1	GE283211
61927167	CBYP844.g1	GE283212
61927168	CBYP846.g1	GE283213
61927169	CBYP847.g1	GE283214
61927170	CBYP848.g1	GE283215
61927171	CBYP849.g1	GE283216
61927172	CBYP850.g1	GE283217
61927173	CBYP851.g1	GE283218
61927174	CBYP852.g1	GE283219
61927175	CBYP853.g1	GE283220
61927176	CBYP854.g1	GE283221

61927177	CBYP856.g1	GE283222
61927178	CBYP857.g1	GE283223
61927179	CBYP858.g1	GE283224
61927180	CBYP859.g1	GE283225
61927181	CBYP860.g1	GE283226
61927182	CBYP861.g1	GE283227
61927183	CBYP862.g1	GE283228
61927184	CBYP863.g1	GE283229
61927185	CBYP865.g1	GE283230
61927186	CBYP866.g1	GE283231
61927187	CBYP867.g1	GE283232
61927188	CBYP868.g1	GE283233
61927189	CBYP869.g1	GE283234
61927190	CBYP870.g1	GE283235
61927191	CBYP871.g1	GE283236
61927192	CBYP873.g1	GE283237
61927193	CBYP874.g1	GE283238
61927194	CBYP875.g1	GE283239
61927195	CBYP876.g1	GE283240
61927196	CBYP877.g1	GE283241
61927197	CBYP878.g1	GE283242
61927198	CBYP879.g1	GE283243
61927199	CBYP880.g1	GE283244
61927200	CBYP881.g1	GE283245
61927201	CBYP882.g1	GE283246
61927202	CBYP883.g1	GE283247
61927203	CBYP884.g1	GE283248
61927204	CBYP885.g1	GE283249
61927205	CBYP886.g1	GE283250
61927206	CBYP888.g1	GE283251
61927207	CBYP889.g1	GE283252
61927208	CBYP890.g1	GE283253
61927209	CBYP891.g1	GE283254
61927210	CBYP892.g1	GE283255
61927211	CBYP893.g1	GE283256
61927212	CBYP894.g1	GE283257
61927213	CBYP895.g1	GE283258
61927214	CBYP897.g1	GE283259
61927215	CBYP899.g1	GE283260
61927216	CBYP900.g1	GE283261
61927217	CBYP901.g1	GE283262
61927218	CBYP902.g1	GE283263
61927219	CBYP903.g1	GE283264
61927220	CBYP905.g1	GE283265
61927221	CBYP906.g1	GE283266
61927222	CBYP907.g1	GE283267
61927223	CBYP908.g1	GE283268
61927224	CBYP909.g1	GE283269
61927225	CBYP910.g1	GE283270
61927226	CBYP911.g1	GE283271
61927227	CBYP912.g1	GE283272
61927228	CBYP913.g1	GE283273
61927229	CBYP914.g1	GE283274
61927230	CBYP916.g1	GE283275
61927231	CBYP917.g1	GE283276
61927232	CBYP918.g1	GE283277
61927233	CBYP919.g1	GE283278
61927234	CBYP920.g1	GE283279
61927235	CBYP921.g1	GE283280
61927236	CBYP922.g1	GE283281
61927237	CBYP923.g1	GE283282
61927238	CBYP924.g1	GE283283
61927239	CBYP925.g1	GE283284

61927240	CBYP926.g1	GE283285
61927241	CBYP927.g1	GE283286
61927242	CBYP928.g1	GE283287
61927243	CBYP929.g1	GE283288
61927244	CBYP930.g1	GE283289
61927245	CBYP931.g1	GE283290
61927246	CBYP933.g1	GE283291
61927247	CBYP934.g1	GE283292
61927248	CBYP935.g1	GE283293
61927249	CBYP936.g1	GE283294
61927250	CBYP938.g1	GE283295
61927251	CBYP939.g1	GE283296
61927252	CBYP940.g1	GE283297
61927253	CBYP942.g1	GE283298
61927254	CBYP943.g1	GE283299
61927255	CBYP944.g1	GE283300
61927256	CBYP945.g1	GE283301
61927257	CBYP946.g1	GE283302
61927258	CBYP947.g1	GE283303
61927259	CBYP948.g1	GE283304
61927260	CBYP949.g1	GE283305
61927261	CBYP950.g1	GE283306
61927262	CBYP951.g1	GE283307
61927263	CBYP952.g1	GE283308
61927264	CBYP953.g1	GE283309
61927265	CBYP954.g1	GE283310
61927266	CBYP955.g1	GE283311
61927267	CBYP956.g1	GE283312
61927268	CBYP957.g1	GE283313
61927269	CBYP958.g1	GE283314
61927270	CBYP959.g1	GE283315
61927271	CBYP960.g1	GE283316
61927272	CBYP961.g1	GE283317
61927273	CBYP962.g1	GE283318
61927274	CBYP963.g1	GE283319
61927275	CBYP965.g1	GE283320
61927276	CBYP966.g1	GE283321
61927277	CBYP967.g1	GE283322
61927278	CBYP968.g1	GE283323
61927279	CBYP970.g1	GE283324
61927280	CBYP972.g1	GE283325
61927281	CBYP973.g1	GE283326
61927282	CBYP974.g1	GE283327
61927283	CBYP975.g1	GE283328
61927284	CBYP976.g1	GE283329
61927285	CBYP977.g1	GE283330
61927286	CBYP978.g1	GE283331
61927287	CBYP979.g1	GE283332
61927288	CBYP980.g1	GE283333
61927289	CBYP981.g1	GE283334
61927290	CBYP983.g1	GE283335
61927291	CBYP984.g1	GE283336
61927292	CBYP985.g1	GE283337
61927293	CBYP986.g1	GE283338
61927294	CBYP987.g1	GE283339
61927295	CBYP988.g1	GE283340
61927296	CBYP989.g1	GE283341
61927297	CBYP990.g1	GE283342
61927298	CBYP992.g1	GE283343
61927299	CBYP993.g1	GE283344
61927300	CBYP995.g1	GE283345
61927301	CBYP996.g1	GE283346
61927302	CBYP997.g1	GE283347

61927303	CBYP998.g1	GE283348
61927304	CBYP999.g1	GE283349
61927305	CBYT1000.b1	GE283350
61927306	CBYT1000.g1	GE283351
61927307	CBYT1001.b1	GE283352
61927308	CBYT1001.g1	GE283353
61927309	CBYT1002.b1	GE283354
61927310	CBYT1002.g1	GE283355
61927311	CBYT1003.b1	GE283356
61927312	CBYT1003.g1	GE283357
61927313	CBYT1004.b1	GE283358
61927314	CBYT1004.g1	GE283359
61927315	CBYT1005.b1	GE283360
61927316	CBYT1005.g1	GE283361
61927317	CBYT1006.b1	GE283362
61927318	CBYT1006.g1	GE283363
61927319	CBYT1007.b1	GE283364
61927320	CBYT1007.g1	GE283365
61927321	CBYT1008.b1	GE283366
61927322	CBYT1008.g1	GE283367
61927323	CBYT1009.b1	GE283368
61927324	CBYT1009.g1	GE283369
61927325	CBYT1010.g1	GE283370
61927326	CBYT1011.g1	GE283371
61927327	CBYT1012.b1	GE283372
61927328	CBYT1012.g1	GE283373
61927329	CBYT1013.b1	GE283374
61927330	CBYT1013.g1	GE283375
61927331	CBYT1014.b1	GE283376
61927332	CBYT1014.g1	GE283377
61927333	CBYT1015.b1	GE283378
61927334	CBYT1015.g1	GE283379
61927335	CBYT1016.b1	GE283380
61927336	CBYT1016.g1	GE283381
61927337	CBYT1017.b1	GE283382
61927338	CBYT1017.g1	GE283383
61927339	CBYT1018.g1	GE283384
61927340	CBYT1019.g1	GE283385
61927341	CBYT1020.g1	GE283386
61927342	CBYT1021.b1	GE283387
61927343	CBYT1021.g1	GE283388
61927344	CBYT1022.b1	GE283389
61927345	CBYT1022.g1	GE283390
61927346	CBYT1023.b1	GE283391
61927347	CBYT1023.g1	GE283392
61927348	CBYT1024.b1	GE283393
61927349	CBYT1024.g1	GE283394
61927350	CBYT1025.b1	GE283395
61927351	CBYT1025.g1	GE283396
61927352	CBYT1026.g1	GE283397
61927353	CBYT1028.b1	GE283398
61927354	CBYT1028.g1	GE283399
61927355	CBYT1029.b1	GE283400
61927356	CBYT1029.g1	GE283401
61927357	CBYT1030.b1	GE283402
61927358	CBYT1030.g1	GE283403
61927359	CBYT1031.b1	GE283404
61927360	CBYT1031.g1	GE283405
61927361	CBYT1032.g1	GE283406
61927362	CBYT1033.b1	GE283407
61927363	CBYT1033.g1	GE283408
61927364	CBYT1034.g1	GE283409
61927365	CBYT1035.g1	GE283410

61927366	CBYT1036.g1	GE283411
61927367	CBYT1037.b1	GE283412
61927368	CBYT1037.g1	GE283413
61927369	CBYT1038.b1	GE283414
61927370	CBYT1038.g1	GE283415
61927371	CBYT1039.b1	GE283416
61927372	CBYT1039.g1	GE283417
61927373	CBYT1040.g1	GE283418
61927374	CBYT1042.b1	GE283419
61927375	CBYT1042.g1	GE283420
61927376	CBYT1043.b1	GE283421
61927377	CBYT1043.g1	GE283422
61927378	CBYT1044.b1	GE283423
61927379	CBYT1044.g1	GE283424
61927380	CBYT1045.b1	GE283425
61927381	CBYT1045.g1	GE283426
61927382	CBYT1046.b1	GE283427
61927383	CBYT1046.g1	GE283428
61927384	CBYT1047.b1	GE283429
61927385	CBYT1047.g1	GE283430
61927386	CBYT1048.b1	GE283431
61927387	CBYT1048.g1	GE283432
61927388	CBYT1049.g1	GE283433
61927389	CBYT1050.b1	GE283434
61927390	CBYT1050.g1	GE283435
61927391	CBYT1051.b1	GE283436
61927392	CBYT1051.g1	GE283437
61927393	CBYT1052.b1	GE283438
61927394	CBYT1052.g1	GE283439
61927395	CBYT1053.b1	GE283440
61927396	CBYT1053.g1	GE283441
61927397	CBYT1054.b1	GE283442
61927398	CBYT1054.g1	GE283443
61927399	CBYT1055.b1	GE283444
61927400	CBYT1055.g1	GE283445
61927401	CBYT1057.b1	GE283446
61927402	CBYT1057.g1	GE283447
61927403	CBYT1058.g1	GE283448
61927404	CBYT1059.b1	GE283449
61927405	CBYT1060.b1	GE283450
61927406	CBYT1060.g1	GE283451
61927407	CBYT1061.b1	GE283452
61927408	CBYT1061.g1	GE283453
61927409	CBYT1062.b1	GE283454
61927410	CBYT1062.g1	GE283455
61927411	CBYT1063.b1	GE283456
61927412	CBYT1063.g1	GE283457
61927413	CBYT1065.b1	GE283458
61927414	CBYT1065.g1	GE283459
61927415	CBYT1066.b1	GE283460
61927416	CBYT1066.g1	GE283461
61927417	CBYT1067.b1	GE283462
61927418	CBYT1067.g1	GE283463
61927419	CBYT1068.b1	GE283464
61927420	CBYT1068.g1	GE283465
61927421	CBYT1069.b1	GE283466
61927422	CBYT1069.g1	GE283467
61927423	CBYT1070.b1	GE283468
61927424	CBYT1070.g1	GE283469
61927425	CBYT1072.b1	GE283470
61927426	CBYT1072.g1	GE283471
61927427	CBYT1073.b1	GE283472
61927428	CBYT1073.g1	GE283473

61927429	CBYT1074.b1	GE283474
61927430	CBYT1074.g1	GE283475
61927431	CBYT1076.b1	GE283476
61927432	CBYT1076.g1	GE283477
61927433	CBYT1077.b1	GE283478
61927434	CBYT1077.g1	GE283479
61927435	CBYT1079.g1	GE283480
61927436	CBYT1080.b1	GE283481
61927437	CBYT1080.g1	GE283482
61927438	CBYT1081.b1	GE283483
61927439	CBYT1081.g1	GE283484
61927440	CBYT1082.b1	GE283485
61927441	CBYT1082.g1	GE283486
61927442	CBYT1083.b1	GE283487
61927443	CBYT1083.g1	GE283488
61927444	CBYT1084.b1	GE283489
61927445	CBYT1084.g1	GE283490
61927446	CBYT1085.b1	GE283491
61927447	CBYT1085.g1	GE283492
61927448	CBYT1086.b1	GE283493
61927449	CBYT1086.g1	GE283494
61927450	CBYT1087.b1	GE283495
61927451	CBYT1087.g1	GE283496
61927452	CBYT1088.b1	GE283497
61927453	CBYT1089.b1	GE283498
61927454	CBYT1089.g1	GE283499
61927455	CBYT1090.g1	GE283500
61927456	CBYT1091.b1	GE283501
61927457	CBYT1091.g1	GE283502
61927458	CBYT1092.b1	GE283503
61927459	CBYT1092.g1	GE283504
61927460	CBYT1093.b1	GE283505
61927461	CBYT1094.b1	GE283506
61927462	CBYT1094.g1	GE283507
61927463	CBYT1096.g1	GE283508
61927464	CBYT1097.g1	GE283509
61927465	CBYT1098.b1	GE283510
61927466	CBYT1098.g1	GE283511
61927467	CBYT1100.b1	GE283512
61927468	CBYT1100.g1	GE283513
61927469	CBYT1101.b1	GE283514
61927470	CBYT1101.g1	GE283515
61927471	CBYT1102.b1	GE283516
61927472	CBYT1102.g1	GE283517
61927473	CBYT1103.b1	GE283518
61927474	CBYT1103.g1	GE283519
61927475	CBYT1105.g1	GE283520
61927476	CBYT1106.g1	GE283521
61927477	CBYT1107.b1	GE283522
61927478	CBYT1107.g1	GE283523
61927479	CBYT1108.b1	GE283524
61927480	CBYT1108.g1	GE283525
61927481	CBYT1109.b1	GE283526
61927482	CBYT1109.g1	GE283527
61927483	CBYT1110.b1	GE283528
61927484	CBYT1110.g1	GE283529
61927485	CBYT1111.b1	GE283530
61927486	CBYT1111.g1	GE283531
61927487	CBYT1112.b1	GE283532
61927488	CBYT1112.g1	GE283533
61927489	CBYT1114.g1	GE283534
61927490	CBYT1115.b1	GE283535
61927491	CBYT1115.g1	GE283536

61927492	CBYT1116.b1	GE283537
61927493	CBYT1116.g1	GE283538
61927494	CBYT1117.b1	GE283539
61927495	CBYT1117.g1	GE283540
61927496	CBYT1118.b1	GE283541
61927497	CBYT1118.g1	GE283542
61927498	CBYT1119.b1	GE283543
61927499	CBYT1119.g1	GE283544
61927500	CBYT1120.b1	GE283545
61927501	CBYT1120.g1	GE283546
61927502	CBYT1121.b1	GE283547
61927503	CBYT1121.g1	GE283548
61927504	CBYT1122.g1	GE283549
61927505	CBYT1123.b1	GE283550
61927506	CBYT1123.g1	GE283551
61927507	CBYT1124.g1	GE283552
61927508	CBYT1125.b1	GE283553
61927509	CBYT1125.g1	GE283554
61927510	CBYT1126.b1	GE283555
61927511	CBYT1126.g1	GE283556
61927512	CBYT1128.b1	GE283557
61927513	CBYT1128.g1	GE283558
61927514	CBYT1129.g1	GE283559
61927515	CBYT1130.b1	GE283560
61927516	CBYT1130.g1	GE283561
61927517	CBYT1131.g1	GE283562
61927518	CBYT1132.g1	GE283563
61927519	CBYT1133.b1	GE283564
61927520	CBYT1133.g1	GE283565
61927521	CBYT1134.b1	GE283566
61927522	CBYT1134.g1	GE283567
61927523	CBYT1135.b1	GE283568
61927524	CBYT1135.g1	GE283569
61927525	CBYT1136.b1	GE283570
61927526	CBYT1136.g1	GE283571
61927527	CBYT1137.b1	GE283572
61927528	CBYT1137.g1	GE283573
61927529	CBYT1138.g1	GE283574
61927530	CBYT1139.b1	GE283575
61927531	CBYT1139.g1	GE283576
61927532	CBYT1140.g1	GE283577
61927533	CBYT1141.b1	GE283578
61927534	CBYT1142.b1	GE283579
61927535	CBYT1142.g1	GE283580
61927536	CBYT1143.b1	GE283581
61927537	CBYT1143.g1	GE283582
61927538	CBYT1146.b1	GE283583
61927539	CBYT1146.g1	GE283584
61927540	CBYT1147.b1	GE283585
61927541	CBYT1147.g1	GE283586
61927542	CBYT1148.g1	GE283587
61927543	CBYT1149.b1	GE283588
61927544	CBYT1149.g1	GE283589
61927545	CBYT1153.b1	GE283590
61927546	CBYT1154.b1	GE283591
61927547	CBYT1154.g1	GE283592
61927548	CBYT1155.b1	GE283593
61927549	CBYT1155.g1	GE283594
61927550	CBYT1156.b1	GE283595
61927551	CBYT1156.g1	GE283596
61927552	CBYT1157.b1	GE283597
61927553	CBYT1157.g1	GE283598
61927554	CBYT1158.b1	GE283599

61927555	CBYT1158.g1	GE283600
61927556	CBYT1159.b1	GE283601
61927557	CBYT1159.g1	GE283602
61927558	CBYT1160.b1	GE283603
61927559	CBYT1160.g1	GE283604
61927560	CBYT1161.b1	GE283605
61927561	CBYT1161.g1	GE283606
61927562	CBYT1162.b1	GE283607
61927563	CBYT1162.g1	GE283608
61927564	CBYT1163.b1	GE283609
61927565	CBYT1163.g1	GE283610
61927566	CBYT1164.b1	GE283611
61927567	CBYT1164.g1	GE283612
61927568	CBYT1165.b1	GE283613
61927569	CBYT1165.g1	GE283614
61927570	CBYT1166.b1	GE283615
61927571	CBYT1166.g1	GE283616
61927572	CBYT1167.b1	GE283617
61927573	CBYT1167.g1	GE283618
61927574	CBYT1168.b1	GE283619
61927575	CBYT1168.g1	GE283620
61927576	CBYT1169.b1	GE283621
61927577	CBYT1170.b1	GE283622
61927578	CBYT1170.g1	GE283623
61927579	CBYT1172.b1	GE283624
61927580	CBYT1173.b1	GE283625
61927581	CBYT1174.b1	GE283626
61927582	CBYT1174.g1	GE283627
61927583	CBYT1175.b1	GE283628
61927584	CBYT1175.g1	GE283629
61927585	CBYT1176.b1	GE283630
61927586	CBYT1178.b1	GE283631
61927587	CBYT1178.g1	GE283632
61927588	CBYT1180.b1	GE283633
61927589	CBYT1180.g1	GE283634
61927590	CBYT1181.b1	GE283635
61927591	CBYT1181.g1	GE283636
61927592	CBYT1182.b1	GE283637
61927593	CBYT1182.g1	GE283638
61927594	CBYT1183.b1	GE283639
61927595	CBYT1183.g1	GE283640
61927596	CBYT1184.b1	GE283641
61927597	CBYT1184.g1	GE283642
61927598	CBYT1186.b1	GE283643
61927599	CBYT1187.b1	GE283644
61927600	CBYT1187.g1	GE283645
61927601	CBYT1188.b1	GE283646
61927602	CBYT1188.g1	GE283647
61927603	CBYT1189.b1	GE283648
61927604	CBYT1189.g1	GE283649
61927605	CBYT1191.b1	GE283650
61927606	CBYT1191.g1	GE283651
61927607	CBYT1192.b1	GE283652
61927608	CBYT1192.g1	GE283653
61927609	CBYT1193.b1	GE283654
61927610	CBYT1193.g1	GE283655
61927611	CBYT1194.b1	GE283656
61927612	CBYT1194.g1	GE283657
61927613	CBYT1195.b1	GE283658
61927614	CBYT1195.g1	GE283659
61927615	CBYT1196.b1	GE283660
61927616	CBYT1196.g1	GE283661
61927617	CBYT1197.b1	GE283662

61927618	CBYT1197.g1	GE283663
61927619	CBYT1199.b1	GE283664
61927620	CBYT1199.g1	GE283665
61927621	CBYT1200.b1	GE283666
61927622	CBYT1200.g1	GE283667
61927623	CBYT1201.b1	GE283668
61927624	CBYT1201.g1	GE283669
61927625	CBYT1202.b1	GE283670
61927626	CBYT1202.g1	GE283671
61927627	CBYT1203.b1	GE283672
61927628	CBYT1203.g1	GE283673
61927629	CBYT1204.b1	GE283674
61927630	CBYT1204.g1	GE283675
61927631	CBYT1205.b1	GE283676
61927632	CBYT1205.g1	GE283677
61927633	CBYT1206.b1	GE283678
61927634	CBYT1206.g1	GE283679
61927635	CBYT1207.b1	GE283680
61927636	CBYT1207.g1	GE283681
61927637	CBYT1208.b1	GE283682
61927638	CBYT1208.g1	GE283683
61927639	CBYT1210.b1	GE283684
61927640	CBYT1210.g1	GE283685
61927641	CBYT1211.b1	GE283686
61927642	CBYT1211.g1	GE283687
61927643	CBYT1212.b1	GE283688
61927644	CBYT1213.b1	GE283689
61927645	CBYT1214.b1	GE283690
61927646	CBYT1214.g1	GE283691
61927647	CBYT1215.b1	GE283692
61927648	CBYT1215.g1	GE283693
61927649	CBYT1216.b1	GE283694
61927650	CBYT1216.g1	GE283695
61927651	CBYT1217.b1	GE283696
61927652	CBYT1217.g1	GE283697
61927653	CBYT1218.b1	GE283698
61927654	CBYT1219.b1	GE283699
61927655	CBYT1219.g1	GE283700
61927656	CBYT1220.b1	GE283701
61927657	CBYT1220.g1	GE283702
61927658	CBYT1221.b1	GE283703
61927659	CBYT1221.g1	GE283704
61927660	CBYT1222.b1	GE283705
61927661	CBYT1222.g1	GE283706
61927662	CBYT1223.b1	GE283707
61927663	CBYT1223.g1	GE283708
61927664	CBYT1224.b1	GE283709
61927665	CBYT1224.g1	GE283710
61927666	CBYT1225.b1	GE283711
61927667	CBYT1225.g1	GE283712
61927668	CBYT1226.b1	GE283713
61927669	CBYT1226.g1	GE283714
61927670	CBYT1227.b1	GE283715
61927671	CBYT1227.g1	GE283716
61927672	CBYT1228.b1	GE283717
61927673	CBYT1228.g1	GE283718
61927674	CBYT1229.g1	GE283719
61927675	CBYT1230.b1	GE283720
61927676	CBYT1230.g1	GE283721
61927677	CBYT1231.b1	GE283722
61927678	CBYT1231.g1	GE283723
61927679	CBYT1232.b1	GE283724
61927680	CBYT1232.g1	GE283725

61927681	CBYT1234.b1	GE283726
61927682	CBYT1235.b1	GE283727
61927683	CBYT1235.g1	GE283728
61927684	CBYT1236.b1	GE283729
61927685	CBYT1236.g1	GE283730
61927686	CBYT1237.b1	GE283731
61927687	CBYT1237.g1	GE283732
61927688	CBYT1239.b1	GE283733
61927689	CBYT1239.g1	GE283734
61927690	CBYT1240.b1	GE283735
61927691	CBYT1240.g1	GE283736
61927692	CBYT1241.b1	GE283737
61927693	CBYT1241.g1	GE283738
61927694	CBYT1243.b1	GE283739
61927695	CBYT1243.g1	GE283740
61927696	CBYT1244.b1	GE283741
61927697	CBYT1244.g1	GE283742
61927698	CBYT1245.b1	GE283743
61927699	CBYT1245.g1	GE283744
61927700	CBYT1246.b1	GE283745
61927701	CBYT1246.g1	GE283746
61927702	CBYT1247.b1	GE283747
61927703	CBYT1247.g1	GE283748
61927704	CBYT1248.b1	GE283749
61927705	CBYT1249.b1	GE283750
61927706	CBYT1249.g1	GE283751
61927707	CBYT1250.b1	GE283752
61927708	CBYT1250.g1	GE283753
61927709	CBYT1252.b1	GE283754
61927710	CBYT1252.g1	GE283755
61927711	CBYT1253.b1	GE283756
61927712	CBYT1253.g1	GE283757
61927713	CBYT1254.b1	GE283758
61927714	CBYT1254.g1	GE283759
61927715	CBYT1255.b1	GE283760
61927716	CBYT1256.g1	GE283761
61927717	CBYT1257.b1	GE283762
61927718	CBYT1258.b1	GE283763
61927719	CBYT1258.g1	GE283764
61927720	CBYT1259.b1	GE283765
61927721	CBYT1259.g1	GE283766
61927722	CBYT1260.b1	GE283767
61927723	CBYT1260.g1	GE283768
61927724	CBYT1261.b1	GE283769
61927725	CBYT1261.g1	GE283770
61927726	CBYT1262.b1	GE283771
61927727	CBYT1262.g1	GE283772
61927728	CBYT1263.b1	GE283773
61927729	CBYT1264.b1	GE283774
61927730	CBYT1264.g1	GE283775
61927731	CBYT1265.b1	GE283776
61927732	CBYT1266.b1	GE283777
61927733	CBYT1267.b1	GE283778
61927734	CBYT1267.g1	GE283779
61927735	CBYT1268.b1	GE283780
61927736	CBYT1268.g1	GE283781
61927737	CBYT1269.b1	GE283782
61927738	CBYT1269.g1	GE283783
61927739	CBYT1270.b1	GE283784
61927740	CBYT1270.g1	GE283785
61927741	CBYT1271.b1	GE283786
61927742	CBYT1271.g1	GE283787
61927743	CBYT1272.b1	GE283788

61927744	CBYT1272.g1	GE283789
61927745	CBYT1273.b1	GE283790
61927746	CBYT1274.b1	GE283791
61927747	CBYT1275.b1	GE283792
61927748	CBYT1275.g1	GE283793
61927749	CBYT1276.b1	GE283794
61927750	CBYT1276.g1	GE283795
61927751	CBYT1277.b1	GE283796
61927752	CBYT1277.g1	GE283797
61927753	CBYT1278.b1	GE283798
61927754	CBYT1278.g1	GE283799
61927755	CBYT1279.b1	GE283800
61927756	CBYT1279.g1	GE283801
61927757	CBYT1280.b1	GE283802
61927758	CBYT1280.g1	GE283803
61927759	CBYT1281.b1	GE283804
61927760	CBYT1281.g1	GE283805
61927761	CBYT1282.b1	GE283806
61927762	CBYT1282.g1	GE283807
61927763	CBYT1283.b1	GE283808
61927764	CBYT1283.g1	GE283809
61927765	CBYT1284.b1	GE283810
61927766	CBYT1284.g1	GE283811
61927767	CBYT1285.b1	GE283812
61927768	CBYT1285.g1	GE283813
61927769	CBYT1286.b1	GE283814
61927770	CBYT1286.g1	GE283815
61927771	CBYT1288.b1	GE283816
61927772	CBYT1288.g1	GE283817
61927773	CBYT1289.b1	GE283818
61927774	CBYT1289.g1	GE283819
61927775	CBYT1290.b1	GE283820
61927776	CBYT1290.g1	GE283821
61927777	CBYT1291.b1	GE283822
61927778	CBYT1291.g1	GE283823
61927779	CBYT1292.b1	GE283824
61927780	CBYT1292.g1	GE283825
61927781	CBYT1293.b1	GE283826
61927782	CBYT1293.g1	GE283827
61927783	CBYT1294.b1	GE283828
61927784	CBYT1294.g1	GE283829
61927785	CBYT1295.b1	GE283830
61927786	CBYT1295.g1	GE283831
61927787	CBYT1296.b1	GE283832
61927788	CBYT1297.b1	GE283833
61927789	CBYT1297.g1	GE283834
61927790	CBYT1298.b1	GE283835
61927791	CBYT1298.g1	GE283836
61927792	CBYT1299.b1	GE283837
61927793	CBYT1299.g1	GE283838
61927794	CBYT1301.b1	GE283839
61927795	CBYT1302.b1	GE283840
61927796	CBYT1302.g1	GE283841
61927797	CBYT1303.b1	GE283842
61927798	CBYT1303.g1	GE283843
61927799	CBYT1304.b1	GE283844
61927800	CBYT1304.g1	GE283845
61927801	CBYT1305.b1	GE283846
61927802	CBYT1306.b1	GE283847
61927803	CBYT1306.g1	GE283848
61927804	CBYT1307.b1	GE283849
61927805	CBYT1307.g1	GE283850
61927806	CBYT1308.b1	GE283851

61927807	CBYT1308.g1	GE283852
61927808	CBYT1309.b1	GE283853
61927809	CBYT1309.g1	GE283854
61927810	CBYT1310.b1	GE283855
61927811	CBYT1311.b1	GE283856
61927812	CBYT1311.g1	GE283857
61927813	CBYT1312.b1	GE283858
61927814	CBYT1312.g1	GE283859
61927815	CBYT1313.b1	GE283860
61927816	CBYT1313.g1	GE283861
61927817	CBYT1314.b1	GE283862
61927818	CBYT1314.g1	GE283863
61927819	CBYT1315.b1	GE283864
61927820	CBYT1315.g1	GE283865
61927821	CBYT1316.b1	GE283866
61927822	CBYT1316.g1	GE283867
61927823	CBYT1317.b1	GE283868
61927824	CBYT1317.g1	GE283869
61927825	CBYT1318.b1	GE283870
61927826	CBYT1319.b1	GE283871
61927827	CBYT1319.g1	GE283872
61927828	CBYT1320.b1	GE283873
61927829	CBYT1320.g1	GE283874
61927830	CBYT1322.b1	GE283875
61927831	CBYT1322.g1	GE283876
61927832	CBYT1323.b1	GE283877
61927833	CBYT1323.g1	GE283878
61927834	CBYT1324.b1	GE283879
61927835	CBYT1325.b1	GE283880
61927836	CBYT1325.g1	GE283881
61927837	CBYT1326.b1	GE283882
61927838	CBYT1326.g1	GE283883
61927839	CBYT1328.b1	GE283884
61927840	CBYT1328.g1	GE283885
61927841	CBYT1329.b1	GE283886
61927842	CBYT1329.g1	GE283887
61927843	CBYT1330.b1	GE283888
61927844	CBYT1330.g1	GE283889
61927845	CBYT1331.b1	GE283890
61927846	CBYT1331.g1	GE283891
61927847	CBYT1332.b1	GE283892
61927848	CBYT1332.g1	GE283893
61927849	CBYT1333.b1	GE283894
61927850	CBYT1333.g1	GE283895
61927851	CBYT1334.b1	GE283896
61927852	CBYT1334.g1	GE283897
61927853	CBYT1335.b1	GE283898
61927854	CBYT1335.g1	GE283899
61927855	CBYT1336.b1	GE283900
61927856	CBYT1336.g1	GE283901
61927857	CBYT1338.b1	GE283902
61927858	CBYT1338.g1	GE283903
61927859	CBYT1340.b1	GE283904
61927860	CBYT1340.g1	GE283905
61927861	CBYT1341.b1	GE283906
61927862	CBYT1341.g1	GE283907
61927863	CBYT1342.b1	GE283908
61927864	CBYT1342.g1	GE283909
61927865	CBYT1343.b1	GE283910
61927866	CBYT1343.g1	GE283911
61927867	CBYT1344.b1	GE283912
61927868	CBYT1344.g1	GE283913
61927869	CBYT1345.b1	GE283914

61927870	CBYT1345.g1	GE283915
61927871	CBYT1346.b1	GE283916
61927872	CBYT1346.g1	GE283917
61927873	CBYT1347.b1	GE283918
61927874	CBYT1347.g1	GE283919
61927875	CBYT1348.b1	GE283920
61927876	CBYT1348.g1	GE283921
61927877	CBYT1349.b1	GE283922
61927878	CBYT1349.g1	GE283923
61927879	CBYT1350.b1	GE283924
61927880	CBYT1350.g1	GE283925
61927881	CBYT1351.b1	GE283926
61927882	CBYT1351.g1	GE283927
61927883	CBYT1352.b1	GE283928
61927884	CBYT1352.g1	GE283929
61927885	CBYT1354.b1	GE283930
61927886	CBYT1355.b1	GE283931
61927887	CBYT1355.g1	GE283932
61927888	CBYT1357.b1	GE283933
61927889	CBYT1357.g1	GE283934
61927890	CBYT1358.b1	GE283935
61927891	CBYT1358.g1	GE283936
61927892	CBYT1359.b1	GE283937
61927893	CBYT1359.g1	GE283938
61927894	CBYT1360.b1	GE283939
61927895	CBYT1360.g1	GE283940
61927896	CBYT1361.b1	GE283941
61927897	CBYT1361.g1	GE283942
61927898	CBYT1362.b1	GE283943
61927899	CBYT1362.g1	GE283944
61927900	CBYT1363.b1	GE283945
61927901	CBYT1363.g1	GE283946
61927902	CBYT1364.b1	GE283947
61927903	CBYT1364.g1	GE283948
61927904	CBYT1365.b1	GE283949
61927905	CBYT1365.g1	GE283950
61927906	CBYT1366.b1	GE283951
61927907	CBYT1366.g1	GE283952
61927908	CBYT1367.b1	GE283953
61927909	CBYT1368.b1	GE283954
61927910	CBYT1368.g1	GE283955
61927911	CBYT1369.b1	GE283956
61927912	CBYT1369.g1	GE283957
61927913	CBYT1370.b1	GE283958
61927914	CBYT1370.g1	GE283959
61927915	CBYT1371.b1	GE283960
61927916	CBYT1371.g1	GE283961
61927917	CBYT1372.b1	GE283962
61927918	CBYT1372.g1	GE283963
61927919	CBYT1373.b1	GE283964
61927920	CBYT1373.g1	GE283965
61927921	CBYT1374.b1	GE283966
61927922	CBYT1374.g1	GE283967
61927923	CBYT1375.b1	GE283968
61927924	CBYT1375.g1	GE283969
61927925	CBYT1376.b1	GE283970
61927926	CBYT1376.g1	GE283971
61927927	CBYT1378.b1	GE283972
61927928	CBYT1378.g1	GE283973
61927929	CBYT1380.g1	GE283974
61927930	CBYT1381.b1	GE283975
61927931	CBYT1381.g1	GE283976
61927932	CBYT1382.b1	GE283977

61927933	CBYT1383.b1	GE283978
61927934	CBYT1383.g1	GE283979
61927935	CBYT1385.b1	GE283980
61927936	CBYT1385.g1	GE283981
61927937	CBYT1386.b1	GE283982
61927938	CBYT1386.g1	GE283983
61927939	CBYT1387.b1	GE283984
61927940	CBYT1387.g1	GE283985
61927941	CBYT1389.b1	GE283986
61927942	CBYT1389.g1	GE283987
61927943	CBYT1390.b1	GE283988
61927944	CBYT1390.g1	GE283989
61927945	CBYT1391.b1	GE283990
61927946	CBYT1391.g1	GE283991
61927947	CBYT1392.b1	GE283992
61927948	CBYT1392.g1	GE283993
61927949	CBYT1393.g1	GE283994
61927950	CBYT1394.b1	GE283995
61927951	CBYT1394.g1	GE283996
61927952	CBYT1395.b1	GE283997
61927953	CBYT1395.g1	GE283998
61927954	CBYT1396.b1	GE283999
61927955	CBYT1396.g1	GE284000
61927956	CBYT1397.b1	GE284001
61927957	CBYT1397.g1	GE284002
61927958	CBYT1398.b1	GE284003
61927959	CBYT1398.g1	GE284004
61927960	CBYT1399.b1	GE284005
61927961	CBYT1400.b1	GE284006
61927962	CBYT1400.g1	GE284007
61927963	CBYT1402.b1	GE284008
61927964	CBYT1403.b1	GE284009
61927965	CBYT1403.g1	GE284010
61927966	CBYT1404.b1	GE284011
61927967	CBYT1404.g1	GE284012
61927968	CBYT1405.b1	GE284013
61927969	CBYT1405.g1	GE284014
61927970	CBYT1406.b1	GE284015
61927971	CBYT1406.g1	GE284016
61927972	CBYT1408.b1	GE284017
61927973	CBYT1408.g1	GE284018
61927974	CBYT1410.b1	GE284019
61927975	CBYT1410.g1	GE284020
61927976	CBYT1411.b1	GE284021
61927977	CBYT1411.g1	GE284022
61927978	CBYT1412.b1	GE284023
61927979	CBYT1412.g1	GE284024
61927980	CBYT1413.b1	GE284025
61927981	CBYT1413.g1	GE284026
61927982	CBYT1414.b1	GE284027
61927983	CBYT1414.g1	GE284028
61927984	CBYT1415.b1	GE284029
61927985	CBYT1415.g1	GE284030
61927986	CBYT1416.b1	GE284031
61927987	CBYT1419.b1	GE284032
61927988	CBYT1419.g1	GE284033
61927989	CBYT1420.b1	GE284034
61927990	CBYT1420.g1	GE284035
61927991	CBYT1421.b1	GE284036
61927992	CBYT1421.g1	GE284037
61927993	CBYT1423.b1	GE284038
61927994	CBYT1423.g1	GE284039
61927995	CBYT1424.b1	GE284040

61927996	CBYT1424.g1	GE284041
61927997	CBYT1426.b1	GE284042
61927998	CBYT1426.g1	GE284043
61927999	CBYT1427.b1	GE284044
61928000	CBYT1427.g1	GE284045
61928001	CBYT1428.g1	GE284046
61928002	CBYT1429.b1	GE284047
61928003	CBYT1429.g1	GE284048
61928004	CBYT1431.b1	GE284049
61928005	CBYT1431.g1	GE284050
61928006	CBYT1432.b1	GE284051
61928007	CBYT1432.g1	GE284052
61928008	CBYT1434.b1	GE284053
61928009	CBYT1434.g1	GE284054
61928010	CBYT1435.b1	GE284055
61928011	CBYT1435.g1	GE284056
61928012	CBYT1436.b1	GE284057
61928013	CBYT1436.g1	GE284058
61928014	CBYT1437.b1	GE284059
61928015	CBYT1438.g1	GE284060
61928016	CBYT1439.b1	GE284061
61928017	CBYT1439.g1	GE284062
61928018	CBYT1440.g1	GE284063
61928019	CBYT1441.b1	GE284064
61928020	CBYT1442.b1	GE284065
61928021	CBYT1442.g1	GE284066
61928022	CBYT1443.b1	GE284067
61928023	CBYT1443.g1	GE284068
61928024	CBYT1444.b1	GE284069
61928025	CBYT1444.g1	GE284070
61928026	CBYT1445.b1	GE284071
61928027	CBYT1445.g1	GE284072
61928028	CBYT1446.b1	GE284073
61928029	CBYT1446.g1	GE284074
61928030	CBYT1447.b1	GE284075
61928031	CBYT1447.g1	GE284076
61928032	CBYT1448.b1	GE284077
61928033	CBYT1448.g1	GE284078
61928034	CBYT1449.b1	GE284079
61928035	CBYT1449.g1	GE284080
61928036	CBYT1450.b1	GE284081
61928037	CBYT1450.g1	GE284082
61928038	CBYT1451.b1	GE284083
61928039	CBYT1451.g1	GE284084
61928040	CBYT1452.b1	GE284085
61928041	CBYT1452.g1	GE284086
61928042	CBYT1453.b1	GE284087
61928043	CBYT1453.g1	GE284088
61928044	CBYT1454.b1	GE284089
61928045	CBYT1455.b1	GE284090
61928046	CBYT1455.g1	GE284091
61928047	CBYT1456.b1	GE284092
61928048	CBYT1456.g1	GE284093
61928049	CBYT1457.b1	GE284094
61928050	CBYT1457.g1	GE284095
61928051	CBYT1458.b1	GE284096
61928052	CBYT1458.g1	GE284097
61928053	CBYT1459.b1	GE284098
61928054	CBYT1459.g1	GE284099
61928055	CBYT1461.b1	GE284100
61928056	CBYT1461.g1	GE284101
61928057	CBYT1462.b1	GE284102
61928058	CBYT1462.g1	GE284103

61928059	CBYT1463.g1	GE284104
61928060	CBYT1464.b1	GE284105
61928061	CBYT1464.g1	GE284106
61928062	CBYT1466.b1	GE284107
61928063	CBYT1467.b1	GE284108
61928064	CBYT1468.b1	GE284109
61928065	CBYT1468.g1	GE284110
61928066	CBYT1469.b1	GE284111
61928067	CBYT1469.g1	GE284112
61928068	CBYT1470.b1	GE284113
61928069	CBYT1470.g1	GE284114
61928070	CBYT1471.b1	GE284115
61928071	CBYT1471.g1	GE284116
61928072	CBYT1472.b1	GE284117
61928073	CBYT1473.b1	GE284118
61928074	CBYT1473.g1	GE284119
61928075	CBYT1474.b1	GE284120
61928076	CBYT1474.g1	GE284121
61928077	CBYT1475.b1	GE284122
61928078	CBYT1475.g1	GE284123
61928079	CBYT1476.b1	GE284124
61928080	CBYT1476.g1	GE284125
61928081	CBYT1477.b1	GE284126
61928082	CBYT1477.g1	GE284127
61928083	CBYT1478.b1	GE284128
61928084	CBYT1478.g1	GE284129
61928085	CBYT1480.b1	GE284130
61928086	CBYT1480.g1	GE284131
61928087	CBYT1481.b1	GE284132
61928088	CBYT1481.g1	GE284133
61928089	CBYT1482.b1	GE284134
61928090	CBYT1482.g1	GE284135
61928091	CBYT1483.b1	GE284136
61928092	CBYT1483.g1	GE284137
61928093	CBYT1484.b1	GE284138
61928094	CBYT1484.g1	GE284139
61928095	CBYT1485.b1	GE284140
61928096	CBYT1485.g1	GE284141
61928097	CBYT1486.b1	GE284142
61928098	CBYT1486.g1	GE284143
61928099	CBYT1487.b1	GE284144
61928100	CBYT1487.g1	GE284145
61928101	CBYT1488.b1	GE284146
61928102	CBYT1488.g1	GE284147
61928103	CBYT1489.b1	GE284148
61928104	CBYT1490.b1	GE284149
61928105	CBYT1491.g1	GE284150
61928106	CBYT1493.b1	GE284151
61928107	CBYT1493.g1	GE284152
61928108	CBYT1494.g1	GE284153
61928109	CBYT1495.b1	GE284154
61928110	CBYT1495.g1	GE284155
61928111	CBYT1496.b1	GE284156
61928112	CBYT1496.g1	GE284157
61928113	CBYT1497.b1	GE284158
61928114	CBYT1497.g1	GE284159
61928115	CBYT1498.b1	GE284160
61928116	CBYT1498.g1	GE284161
61928117	CBYT1499.b1	GE284162
61928118	CBYT1499.g1	GE284163
61928119	CBYT1500.b1	GE284164
61928120	CBYT1500.g1	GE284165
61928121	CBYT1501.b1	GE284166

61928122	CBYT1501.g1	GE284167
61928123	CBYT1502.b1	GE284168
61928124	CBYT1502.g1	GE284169
61928125	CBYT1503.b1	GE284170
61928126	CBYT1503.g1	GE284171
61928127	CBYT1504.b1	GE284172
61928128	CBYT1504.g1	GE284173
61928129	CBYT1505.b1	GE284174
61928130	CBYT1505.g1	GE284175
61928131	CBYT1506.b1	GE284176
61928132	CBYT1506.g1	GE284177
61928133	CBYT1507.b1	GE284178
61928134	CBYT1507.g1	GE284179
61928135	CBYT1508.b1	GE284180
61928136	CBYT1509.b1	GE284181
61928137	CBYT1509.g1	GE284182
61928138	CBYT1510.b1	GE284183
61928139	CBYT1510.g1	GE284184
61928140	CBYT1511.b1	GE284185
61928141	CBYT1511.g1	GE284186
61928142	CBYT1512.b1	GE284187
61928143	CBYT1512.g1	GE284188
61928144	CBYT1513.b1	GE284189
61928145	CBYT1513.g1	GE284190
61928146	CBYT1514.b1	GE284191
61928147	CBYT1514.g1	GE284192
61928148	CBYT1515.b1	GE284193
61928149	CBYT1515.g1	GE284194
61928150	CBYT1516.b1	GE284195
61928151	CBYT1517.b1	GE284196
61928152	CBYT1517.g1	GE284197
61928153	CBYT1518.b1	GE284198
61928154	CBYT1518.g1	GE284199
61928155	CBYT1519.b1	GE284200
61928156	CBYT1519.g1	GE284201
61928157	CBYT1520.b1	GE284202
61928158	CBYT1520.g1	GE284203
61928159	CBYT1522.b1	GE284204
61928160	CBYT1522.g1	GE284205
61928161	CBYT1523.b1	GE284206
61928162	CBYT1523.g1	GE284207
61928163	CBYT1524.b1	GE284208
61928164	CBYT1524.g1	GE284209
61928165	CBYT1525.b1	GE284210
61928166	CBYT1525.g1	GE284211
61928167	CBYT1526.b1	GE284212
61928168	CBYT1526.g1	GE284213
61928169	CBYT1527.b1	GE284214
61928170	CBYT1527.g1	GE284215
61928171	CBYT1531.b1	GE284216
61928172	CBYT1531.g1	GE284217
61928173	CBYT1533.b1	GE284218
61928174	CBYT1533.g1	GE284219
61928175	CBYT1534.b1	GE284220
61928176	CBYT1534.g1	GE284221
61928177	CBYT1537.b1	GE284222
61928178	CBYT1537.g1	GE284223
61928179	CBYT1538.b1	GE284224
61928180	CBYT1538.g1	GE284225
61928181	CBYT1539.g1	GE284226
61928182	CBYT1540.b1	GE284227
61928183	CBYT1541.b1	GE284228
61928184	CBYT1541.g1	GE284229

61928185	CBYT1542.b1	GE284230
61928186	CBYT1542.g1	GE284231
61928187	CBYT1543.b1	GE284232
61928188	CBYT1543.g1	GE284233
61928189	CBYT1544.b1	GE284234
61928190	CBYT1544.g1	GE284235
61928191	CBYT1545.b1	GE284236
61928192	CBYT1545.g1	GE284237
61928193	CBYT1546.b1	GE284238
61928194	CBYT1546.g1	GE284239
61928195	CBYT1547.b1	GE284240
61928196	CBYT1547.g1	GE284241
61928197	CBYT1548.b1	GE284242
61928198	CBYT1548.g1	GE284243
61928199	CBYT1549.b1	GE284244
61928200	CBYT1549.g1	GE284245
61928201	CBYT1551.b1	GE284246
61928202	CBYT1551.g1	GE284247
61928203	CBYT1552.b1	GE284248
61928204	CBYT1552.g1	GE284249
61928205	CBYT1553.b1	GE284250
61928206	CBYT1553.g1	GE284251
61928207	CBYT1554.b1	GE284252
61928208	CBYT1554.g1	GE284253
61928209	CBYT1555.b1	GE284254
61928210	CBYT1555.g1	GE284255
61928211	CBYT1556.b1	GE284256
61928212	CBYT1557.b1	GE284257
61928213	CBYT1557.g1	GE284258
61928214	CBYT1558.b1	GE284259
61928215	CBYT1559.b1	GE284260
61928216	CBYT1559.g1	GE284261
61928217	CBYT1560.b1	GE284262
61928218	CBYT1560.g1	GE284263
61928219	CBYT1562.b1	GE284264
61928220	CBYT1562.g1	GE284265
61928221	CBYT1563.b1	GE284266
61928222	CBYT1564.b1	GE284267
61928223	CBYT1564.g1	GE284268
61928224	CBYT1565.b1	GE284269
61928225	CBYT1565.g1	GE284270
61928226	CBYT1566.b1	GE284271
61928227	CBYT1566.g1	GE284272
61928228	CBYT1567.b1	GE284273
61928229	CBYT1567.g1	GE284274
61928230	CBYT1568.b1	GE284275
61928231	CBYT1568.g1	GE284276
61928232	CBYT1569.b1	GE284277
61928233	CBYT1569.g1	GE284278
61928234	CBYT1570.b1	GE284279
61928235	CBYT1570.g1	GE284280
61928236	CBYT1572.b1	GE284281
61928237	CBYT1572.g1	GE284282
61928238	CBYT1573.b1	GE284283
61928239	CBYT1573.g1	GE284284
61928240	CBYT1574.b1	GE284285
61928241	CBYT1574.g1	GE284286
61928242	CBYT1575.b1	GE284287
61928243	CBYT1575.g1	GE284288
61928244	CBYT1576.b1	GE284289
61928245	CBYT1576.g1	GE284290
61928246	CBYT1577.g1	GE284291
61928247	CBYT1578.b1	GE284292

61928248	CBYT1580.b1	GE284293
61928249	CBYT1580.g1	GE284294
61928250	CBYT1581.b1	GE284295
61928251	CBYT1581.g1	GE284296
61928252	CBYT1582.b1	GE284297
61928253	CBYT1582.g1	GE284298
61928254	CBYT1583.b1	GE284299
61928255	CBYT1583.g1	GE284300
61928256	CBYT1584.b1	GE284301
61928257	CBYT1584.g1	GE284302
61928258	CBYT1585.b1	GE284303
61928259	CBYT1585.g1	GE284304
61928260	CBYT1586.b1	GE284305
61928261	CBYT1587.b1	GE284306
61928262	CBYT1587.g1	GE284307
61928263	CBYT1588.b1	GE284308
61928264	CBYT1588.g1	GE284309
61928265	CBYT1589.b1	GE284310
61928266	CBYT1589.g1	GE284311
61928267	CBYT1590.b1	GE284312
61928268	CBYT1590.g1	GE284313
61928269	CBYT1591.b1	GE284314
61928270	CBYT1591.g1	GE284315
61928271	CBYT1592.b1	GE284316
61928272	CBYT1592.g1	GE284317
61928273	CBYT1594.b1	GE284318
61928274	CBYT1594.g1	GE284319
61928275	CBYT1595.b1	GE284320
61928276	CBYT1595.g1	GE284321
61928277	CBYT1596.b1	GE284322
61928278	CBYT1596.g1	GE284323
61928279	CBYT1597.g1	GE284324
61928280	CBYT1598.b1	GE284325
61928281	CBYT1598.g1	GE284326
61928282	CBYT1599.b1	GE284327
61928283	CBYT1599.g1	GE284328
61928284	CBYT1600.b1	GE284329
61928285	CBYT1601.b1	GE284330
61928286	CBYT1601.g1	GE284331
61928287	CBYT1602.b1	GE284332
61928288	CBYT1602.g1	GE284333
61928289	CBYT1603.b1	GE284334
61928290	CBYT1603.g1	GE284335
61928291	CBYT1604.b1	GE284336
61928292	CBYT1604.g1	GE284337
61928293	CBYT1605.b1	GE284338
61928294	CBYT1605.g1	GE284339
61928295	CBYT1606.b1	GE284340
61928296	CBYT1606.g1	GE284341
61928297	CBYT1608.b1	GE284342
61928298	CBYT1608.g1	GE284343
61928299	CBYT1609.b1	GE284344
61928300	CBYT1609.g1	GE284345
61928301	CBYT1610.b1	GE284346
61928302	CBYT1610.g1	GE284347
61928303	CBYT1611.b1	GE284348
61928304	CBYT1611.g1	GE284349
61928305	CBYT1612.b1	GE284350
61928306	CBYT1612.g1	GE284351
61928307	CBYT1613.b1	GE284352
61928308	CBYT1613.g1	GE284353
61928309	CBYT1614.b1	GE284354
61928310	CBYT1614.g1	GE284355

61928311	CBYT1615.b1	GE284356
61928312	CBYT1615.g1	GE284357
61928313	CBYT1616.b1	GE284358
61928314	CBYT1616.g1	GE284359
61928315	CBYT1617.b1	GE284360
61928316	CBYT1617.g1	GE284361
61928317	CBYT1618.b1	GE284362
61928318	CBYT1618.g1	GE284363
61928319	CBYT1619.b1	GE284364
61928320	CBYT1619.g1	GE284365
61928321	CBYT1620.b1	GE284366
61928322	CBYT1620.g1	GE284367
61928323	CBYT1621.b1	GE284368
61928324	CBYT1622.b1	GE284369
61928325	CBYT1622.g1	GE284370
61928326	CBYT1623.b1	GE284371
61928327	CBYT1623.g1	GE284372
61928328	CBYT1624.b1	GE284373
61928329	CBYT1624.g1	GE284374
61928330	CBYT1625.b1	GE284375
61928331	CBYT1625.g1	GE284376
61928332	CBYT1626.b1	GE284377
61928333	CBYT1626.g1	GE284378
61928334	CBYT1627.b1	GE284379
61928335	CBYT1627.g1	GE284380
61928336	CBYT1628.b1	GE284381
61928337	CBYT1628.g1	GE284382
61928338	CBYT1629.b1	GE284383
61928339	CBYT1629.g1	GE284384
61928340	CBYT1630.b1	GE284385
61928341	CBYT1630.g1	GE284386
61928342	CBYT1631.b1	GE284387
61928343	CBYT1631.g1	GE284388
61928344	CBYT1632.b1	GE284389
61928345	CBYT1632.g1	GE284390
61928346	CBYT1633.b1	GE284391
61928347	CBYT1633.g1	GE284392
61928348	CBYT1634.b1	GE284393
61928349	CBYT1634.g1	GE284394
61928350	CBYT1635.b1	GE284395
61928351	CBYT1635.g1	GE284396
61928352	CBYT1636.b1	GE284397
61928353	CBYT1636.g1	GE284398
61928354	CBYT1637.b1	GE284399
61928355	CBYT1637.g1	GE284400
61928356	CBYT1638.b1	GE284401
61928357	CBYT1638.g1	GE284402
61928358	CBYT1640.b1	GE284403
61928359	CBYT1640.g1	GE284404
61928360	CBYT1641.b1	GE284405
61928361	CBYT1641.g1	GE284406
61928362	CBYT1642.b1	GE284407
61928363	CBYT1642.g1	GE284408
61928364	CBYT1643.b1	GE284409
61928365	CBYT1643.g1	GE284410
61928366	CBYT1644.b1	GE284411
61928367	CBYT1644.g1	GE284412
61928368	CBYT1645.b1	GE284413
61928369	CBYT1646.b1	GE284414
61928370	CBYT1646.g1	GE284415
61928371	CBYT1647.b1	GE284416
61928372	CBYT1647.g1	GE284417
61928373	CBYT1648.b1	GE284418

61928374	CBYT1648.g1	GE284419
61928375	CBYT1649.b1	GE284420
61928376	CBYT1649.g1	GE284421
61928377	CBYT1650.b1	GE284422
61928378	CBYT1650.g1	GE284423
61928379	CBYT1651.b1	GE284424
61928380	CBYT1651.g1	GE284425
61928381	CBYT1652.b1	GE284426
61928382	CBYT1652.g1	GE284427
61928383	CBYT1653.b1	GE284428
61928384	CBYT1653.g1	GE284429
61928385	CBYT1654.b1	GE284430
61928386	CBYT1654.g1	GE284431
61928387	CBYT1655.b1	GE284432
61928388	CBYT1655.g1	GE284433
61928389	CBYT1656.b1	GE284434
61928390	CBYT1656.g1	GE284435
61928391	CBYT1657.b1	GE284436
61928392	CBYT1657.g1	GE284437
61928393	CBYT1658.b1	GE284438
61928394	CBYT1658.g1	GE284439
61928395	CBYT1659.b1	GE284440
61928396	CBYT1659.g1	GE284441
61928397	CBYT1660.b1	GE284442
61928398	CBYT1660.g1	GE284443
61928399	CBYT1661.b1	GE284444
61928400	CBYT1661.g1	GE284445
61928401	CBYT1662.b1	GE284446
61928402	CBYT1662.g1	GE284447
61928403	CBYT1663.g1	GE284448
61928404	CBYT1664.b1	GE284449
61928405	CBYT1664.g1	GE284450
61928406	CBYT1665.b1	GE284451
61928407	CBYT1665.g1	GE284452
61928408	CBYT1666.b1	GE284453
61928409	CBYT1666.g1	GE284454
61928410	CBYT1667.b1	GE284455
61928411	CBYT1667.g1	GE284456
61928412	CBYT1668.b1	GE284457
61928413	CBYT1668.g1	GE284458
61928414	CBYT1669.b1	GE284459
61928415	CBYT1669.g1	GE284460
61928416	CBYT1670.b1	GE284461
61928417	CBYT1670.g1	GE284462
61928418	CBYT1671.b1	GE284463
61928419	CBYT1671.g1	GE284464
61928420	CBYT1672.b1	GE284465
61928421	CBYT1672.g1	GE284466
61928422	CBYT1673.b1	GE284467
61928423	CBYT1673.g1	GE284468
61928424	CBYT1674.b1	GE284469
61928425	CBYT1674.g1	GE284470
61928426	CBYT1675.b1	GE284471
61928427	CBYT1676.b1	GE284472
61928428	CBYT1677.b1	GE284473
61928429	CBYT1677.g1	GE284474
61928430	CBYT1678.b1	GE284475
61928431	CBYT1678.g1	GE284476
61928432	CBYT1679.b1	GE284477
61928433	CBYT1679.g1	GE284478
61928434	CBYT1680.b1	GE284479
61928435	CBYT1680.g1	GE284480
61928436	CBYT1681.b1	GE284481

61928437	CBYT1681.g1	GE284482
61928438	CBYT1682.b1	GE284483
61928439	CBYT1682.g1	GE284484
61928440	CBYT1683.b1	GE284485
61928441	CBYT1684.b1	GE284486
61928442	CBYT1684.g1	GE284487
61928443	CBYT1685.b1	GE284488
61928444	CBYT1685.g1	GE284489
61928445	CBYT1686.b1	GE284490
61928446	CBYT1686.g1	GE284491
61928447	CBYT1687.b1	GE284492
61928448	CBYT1687.g1	GE284493
61928449	CBYT1688.b1	GE284494
61928450	CBYT1689.b1	GE284495
61928451	CBYT1689.g1	GE284496
61928452	CBYT1690.b1	GE284497
61928453	CBYT1690.g1	GE284498
61928454	CBYT1691.b1	GE284499
61928455	CBYT1691.g1	GE284500
61928456	CBYT1692.b1	GE284501
61928457	CBYT1692.g1	GE284502
61928458	CBYT1693.b1	GE284503
61928459	CBYT1694.b1	GE284504
61928460	CBYT1694.g1	GE284505
61928461	CBYT1695.b1	GE284506
61928462	CBYT1695.g1	GE284507
61928463	CBYT1697.b1	GE284508
61928464	CBYT1697.g1	GE284509
61928465	CBYT1698.b1	GE284510
61928466	CBYT1698.g1	GE284511
61928467	CBYT1699.b1	GE284512
61928468	CBYT1699.g1	GE284513
61928469	CBYT1700.b1	GE284514
61928470	CBYT1700.g1	GE284515
61928471	CBYT1702.b1	GE284516
61928472	CBYT1702.g1	GE284517
61928473	CBYT1703.b1	GE284518
61928474	CBYT1703.g1	GE284519
61928475	CBYT1704.b1	GE284520
61928476	CBYT1704.g1	GE284521
61928477	CBYT1705.b1	GE284522
61928478	CBYT1707.b1	GE284523
61928479	CBYT1707.g1	GE284524
61928480	CBYT1708.b1	GE284525
61928481	CBYT1708.g1	GE284526
61928482	CBYT1709.b1	GE284527
61928483	CBYT1709.g1	GE284528
61928484	CBYT1710.b1	GE284529
61928485	CBYT1710.g1	GE284530
61928486	CBYT1711.b1	GE284531
61928487	CBYT1711.g1	GE284532
61928488	CBYT1712.b1	GE284533
61928489	CBYT1712.g1	GE284534
61928490	CBYT1713.b1	GE284535
61928491	CBYT1713.g1	GE284536
61928492	CBYT1714.b1	GE284537
61928493	CBYT1714.g1	GE284538
61928494	CBYT1715.b1	GE284539
61928495	CBYT1715.g1	GE284540
61928496	CBYT1716.b1	GE284541
61928497	CBYT1716.g1	GE284542
61928498	CBYT1717.b1	GE284543
61928499	CBYT1717.g1	GE284544

61928500	CBYT1718.g1	GE284545
61928501	CBYT1719.b1	GE284546
61928502	CBYT1719.g1	GE284547
61928503	CBYT1720.b1	GE284548
61928504	CBYT1720.g1	GE284549
61928505	CBYT1722.b1	GE284550
61928506	CBYT1722.g1	GE284551
61928507	CBYT1723.b1	GE284552
61928508	CBYT1723.g1	GE284553
61928509	CBYT1724.b1	GE284554
61928510	CBYT1725.b1	GE284555
61928511	CBYT1726.b1	GE284556
61928512	CBYT1726.g1	GE284557
61928513	CBYT1727.b1	GE284558
61928514	CBYT1727.g1	GE284559
61928515	CBYT1728.b1	GE284560
61928516	CBYT1728.g1	GE284561
61928517	CBYT1729.b1	GE284562
61928518	CBYT1729.g1	GE284563
61928519	CBYT1730.b1	GE284564
61928520	CBYT1730.g1	GE284565
61928521	CBYT1731.b1	GE284566
61928522	CBYT1732.b1	GE284567
61928523	CBYT1732.g1	GE284568
61928524	CBYT1733.b1	GE284569
61928525	CBYT1733.g1	GE284570
61928526	CBYT1734.b1	GE284571
61928527	CBYT1734.g1	GE284572
61928528	CBYT1735.b1	GE284573
61928529	CBYT1735.g1	GE284574
61928530	CBYT1736.b1	GE284575
61928531	CBYT1736.g1	GE284576
61928532	CBYT1737.b1	GE284577
61928533	CBYT1737.g1	GE284578
61928534	CBYT1738.b1	GE284579
61928535	CBYT1739.b1	GE284580
61928536	CBYT1739.g1	GE284581
61928537	CBYT1740.b1	GE284582
61928538	CBYT1740.g1	GE284583
61928539	CBYT1741.b1	GE284584
61928540	CBYT1741.g1	GE284585
61928541	CBYT1742.b1	GE284586
61928542	CBYT1742.g1	GE284587
61928543	CBYT1743.b1	GE284588
61928544	CBYT1743.g1	GE284589
61928545	CBYT1744.b1	GE284590
61928546	CBYT1744.g1	GE284591
61928547	CBYT1745.b1	GE284592
61928548	CBYT1745.g1	GE284593
61928549	CBYT1746.b1	GE284594
61928550	CBYT1746.g1	GE284595
61928551	CBYT1747.b1	GE284596
61928552	CBYT1748.b1	GE284597
61928553	CBYT1748.g1	GE284598
61928554	CBYT1749.g1	GE284599
61928555	CBYT1750.b1	GE284600
61928556	CBYT1750.g1	GE284601
61928557	CBYT1751.b1	GE284602
61928558	CBYT1751.g1	GE284603
61928559	CBYT1752.b1	GE284604
61928560	CBYT1752.g1	GE284605
61928561	CBYT1753.b1	GE284606
61928562	CBYT1753.g1	GE284607

61928563	CBYT1754.b1	GE284608
61928564	CBYT1754.g1	GE284609
61928565	CBYT1755.b1	GE284610
61928566	CBYT1755.g1	GE284611
61928567	CBYT1756.b1	GE284612
61928568	CBYT1756.g1	GE284613
61928569	CBYT1757.b1	GE284614
61928570	CBYT1757.g1	GE284615
61928571	CBYT1758.b1	GE284616
61928572	CBYT1758.g1	GE284617
61928573	CBYT1759.b1	GE284618
61928574	CBYT1759.g1	GE284619
61928575	CBYT1760.b1	GE284620
61928576	CBYT1760.g1	GE284621
61928577	CBYT1762.b1	GE284622
61928578	CBYT1762.g1	GE284623
61928579	CBYT1763.b1	GE284624
61928580	CBYT1763.g1	GE284625
61928581	CBYT1764.b1	GE284626
61928582	CBYT1764.g1	GE284627
61928583	CBYT1765.g1	GE284628
61928584	CBYT1766.b1	GE284629
61928585	CBYT1766.g1	GE284630
61928586	CBYT1767.b1	GE284631
61928587	CBYT1767.g1	GE284632
61928588	CBYT1768.b1	GE284633
61928589	CBYT1768.g1	GE284634
61928590	CBYT1769.b1	GE284635
61928591	CBYT1769.g1	GE284636
61928592	CBYT1770.b1	GE284637
61928593	CBYT1770.g1	GE284638
61928594	CBYT1771.b1	GE284639
61928595	CBYT1771.g1	GE284640
61928596	CBYT1772.b1	GE284641
61928597	CBYT1772.g1	GE284642
61928598	CBYT1773.b1	GE284643
61928599	CBYT1773.g1	GE284644
61928600	CBYT1774.g1	GE284645
61928601	CBYT1775.b1	GE284646
61928602	CBYT1775.g1	GE284647
61928603	CBYT1776.b1	GE284648
61928604	CBYT1777.b1	GE284649
61928605	CBYT1777.g1	GE284650
61928606	CBYT1778.b1	GE284651
61928607	CBYT1778.g1	GE284652
61928608	CBYT1779.b1	GE284653
61928609	CBYT1779.g1	GE284654
61928610	CBYT1781.b1	GE284655
61928611	CBYT1781.g1	GE284656
61928612	CBYT1782.b1	GE284657
61928613	CBYT1782.g1	GE284658
61928614	CBYT1783.b1	GE284659
61928615	CBYT1783.g1	GE284660
61928616	CBYT1784.b1	GE284661
61928617	CBYT1784.g1	GE284662
61928618	CBYT1785.b1	GE284663
61928619	CBYT1785.g1	GE284664
61928620	CBYT1787.b1	GE284665
61928621	CBYT1788.b1	GE284666
61928622	CBYT1788.g1	GE284667
61928623	CBYT1789.b1	GE284668
61928624	CBYT1789.g1	GE284669
61928625	CBYT1790.b1	GE284670

61928626	CBYT1790.g1	GE284671
61928627	CBYT1791.b1	GE284672
61928628	CBYT1791.g1	GE284673
61928629	CBYT1792.b1	GE284674
61928630	CBYT1792.g1	GE284675
61928631	CBYT1793.b1	GE284676
61928632	CBYT1793.g1	GE284677
61928633	CBYT1794.b1	GE284678
61928634	CBYT1794.g1	GE284679
61928635	CBYT1795.b1	GE284680
61928636	CBYT1795.g1	GE284681
61928637	CBYT1796.b1	GE284682
61928638	CBYT1796.g1	GE284683
61928639	CBYT1797.b1	GE284684
61928640	CBYT1797.g1	GE284685
61928641	CBYT1798.b1	GE284686
61928642	CBYT1798.g1	GE284687
61928643	CBYT1799.b1	GE284688
61928644	CBYT1799.g1	GE284689
61928645	CBYT1800.b1	GE284690
61928646	CBYT1800.g1	GE284691
61928647	CBYT1802.b1	GE284692
61928648	CBYT1803.b1	GE284693
61928649	CBYT1803.g1	GE284694
61928650	CBYT1804.b1	GE284695
61928651	CBYT1804.g1	GE284696
61928652	CBYT1805.b1	GE284697
61928653	CBYT1805.g1	GE284698
61928654	CBYT1806.b1	GE284699
61928655	CBYT1806.g1	GE284700
61928656	CBYT1807.b1	GE284701
61928657	CBYT1807.g1	GE284702
61928658	CBYT1808.b1	GE284703
61928659	CBYT1808.g1	GE284704
61928660	CBYT1809.b1	GE284705
61928661	CBYT1809.g1	GE284706
61928662	CBYT1810.b1	GE284707
61928663	CBYT1810.g1	GE284708
61928664	CBYT1811.b1	GE284709
61928665	CBYT1811.g1	GE284710
61928666	CBYT1812.b1	GE284711
61928667	CBYT1812.g1	GE284712
61928668	CBYT1814.b1	GE284713
61928669	CBYT1814.g1	GE284714
61928670	CBYT1815.g1	GE284715
61928671	CBYT1816.b1	GE284716
61928672	CBYT1816.g1	GE284717
61928673	CBYT1817.b1	GE284718
61928674	CBYT1817.g1	GE284719
61928675	CBYT1818.b1	GE284720
61928676	CBYT1818.g1	GE284721
61928677	CBYT1819.b1	GE284722
61928678	CBYT1819.g1	GE284723
61928679	CBYT1820.b1	GE284724
61928680	CBYT1820.g1	GE284725
61928681	CBYT1821.b1	GE284726
61928682	CBYT1821.g1	GE284727
61928683	CBYT1822.b1	GE284728
61928684	CBYT1822.g1	GE284729
61928685	CBYT1823.b1	GE284730
61928686	CBYT1823.g1	GE284731
61928687	CBYT1825.b1	GE284732
61928688	CBYT1825.g1	GE284733

61928689	CBYT1826.b1	GE284734
61928690	CBYT1826.g1	GE284735
61928691	CBYT1827.b1	GE284736
61928692	CBYT1827.g1	GE284737
61928693	CBYT1828.b1	GE284738
61928694	CBYT1828.g1	GE284739
61928695	CBYT1829.b1	GE284740
61928696	CBYT1829.g1	GE284741
61928697	CBYT1830.b1	GE284742
61928698	CBYT1830.g1	GE284743
61928699	CBYT1831.b1	GE284744
61928700	CBYT1831.g1	GE284745
61928701	CBYT1832.b1	GE284746
61928702	CBYT1832.g1	GE284747
61928703	CBYT1834.b1	GE284748
61928704	CBYT1834.g1	GE284749
61928705	CBYT1835.b1	GE284750
61928706	CBYT1835.g1	GE284751
61928707	CBYT1836.g1	GE284752
61928708	CBYT1837.b1	GE284753
61928709	CBYT1837.g1	GE284754
61928710	CBYT1838.b1	GE284755
61928711	CBYT1838.g1	GE284756
61928712	CBYT1839.b1	GE284757
61928713	CBYT1839.g1	GE284758
61928714	CBYT1840.b1	GE284759
61928715	CBYT1840.g1	GE284760
61928716	CBYT1841.b1	GE284761
61928717	CBYT1841.g1	GE284762
61928718	CBYT1842.b1	GE284763
61928719	CBYT1842.g1	GE284764
61928720	CBYT1843.b1	GE284765
61928721	CBYT1843.g1	GE284766
61928722	CBYT1844.b1	GE284767
61928723	CBYT1844.g1	GE284768
61928724	CBYT1845.b1	GE284769
61928725	CBYT1845.g1	GE284770
61928726	CBYT1846.b1	GE284771
61928727	CBYT1847.b1	GE284772
61928728	CBYT1847.g1	GE284773
61928729	CBYT1848.b1	GE284774
61928730	CBYT1849.b1	GE284775
61928731	CBYT1851.b1	GE284776
61928732	CBYT1851.g1	GE284777
61928733	CBYT1852.b1	GE284778
61928734	CBYT1852.g1	GE284779
61928735	CBYT1853.b1	GE284780
61928736	CBYT1853.g1	GE284781
61928737	CBYT1854.b1	GE284782
61928738	CBYT1854.g1	GE284783
61928739	CBYT1855.b1	GE284784
61928740	CBYT1856.b1	GE284785
61928741	CBYT1856.g1	GE284786
61928742	CBYT1857.b1	GE284787
61928743	CBYT1857.g1	GE284788
61928744	CBYT1858.b1	GE284789
61928745	CBYT1858.g1	GE284790
61928746	CBYT1859.b1	GE284791
61928747	CBYT1860.b1	GE284792
61928748	CBYT1860.g1	GE284793
61928749	CBYT1861.b1	GE284794
61928750	CBYT1861.g1	GE284795
61928751	CBYT1862.g1	GE284796

61928752	CBYT1863.b1	GE284797
61928753	CBYT1863.g1	GE284798
61928754	CBYT1864.b1	GE284799
61928755	CBYT1864.g1	GE284800
61928756	CBYT1865.b1	GE284801
61928757	CBYT1865.g1	GE284802
61928758	CBYT1866.b1	GE284803
61928759	CBYT1866.g1	GE284804
61928760	CBYT1868.b1	GE284805
61928761	CBYT1868.g1	GE284806
61928762	CBYT1869.b1	GE284807
61928763	CBYT1869.g1	GE284808
61928764	CBYT1872.b1	GE284809
61928765	CBYT1872.g1	GE284810
61928766	CBYT1873.b1	GE284811
61928767	CBYT1874.b1	GE284812
61928768	CBYT1874.g1	GE284813
61928769	CBYT1875.b1	GE284814
61928770	CBYT1875.g1	GE284815
61928771	CBYT1877.b1	GE284816
61928772	CBYT1877.g1	GE284817
61928773	CBYT1878.b1	GE284818
61928774	CBYT1878.g1	GE284819
61928775	CBYT1879.b1	GE284820
61928776	CBYT1879.g1	GE284821
61928777	CBYT1880.b1	GE284822
61928778	CBYT1880.g1	GE284823
61928779	CBYT1881.b1	GE284824
61928780	CBYT1881.g1	GE284825
61928781	CBYT1882.b1	GE284826
61928782	CBYT1882.g1	GE284827
61928783	CBYT1884.b1	GE284828
61928784	CBYT1884.g1	GE284829
61928785	CBYT1887.b1	GE284830
61928786	CBYT1887.g1	GE284831
61928787	CBYT1888.b1	GE284832
61928788	CBYT1888.g1	GE284833
61928789	CBYT1889.b1	GE284834
61928790	CBYT1889.g1	GE284835
61928791	CBYT1890.b1	GE284836
61928792	CBYT1890.g1	GE284837
61928793	CBYT1891.b1	GE284838
61928794	CBYT1891.g1	GE284839
61928795	CBYT1892.b1	GE284840
61928796	CBYT1892.g1	GE284841
61928797	CBYT1893.b1	GE284842
61928798	CBYT1893.g1	GE284843
61928799	CBYT1894.b1	GE284844
61928800	CBYT1894.g1	GE284845
61928801	CBYT1895.b1	GE284846
61928802	CBYT1895.g1	GE284847
61928803	CBYT1896.b1	GE284848
61928804	CBYT1896.g1	GE284849
61928805	CBYT1897.b1	GE284850
61928806	CBYT1897.g1	GE284851
61928807	CBYT1898.b1	GE284852
61928808	CBYT1898.g1	GE284853
61928809	CBYT1899.b1	GE284854
61928810	CBYT1899.g1	GE284855
61928811	CBYT1900.b1	GE284856
61928812	CBYT1900.g1	GE284857
61928813	CBYT1901.b1	GE284858
61928814	CBYT1901.g1	GE284859

61928815	CBYT1902.b1	GE284860
61928816	CBYT1902.g1	GE284861
61928817	CBYT1903.b1	GE284862
61928818	CBYT1903.g1	GE284863
61928819	CBYT1904.b1	GE284864
61928820	CBYT1904.g1	GE284865
61928821	CBYT1905.b1	GE284866
61928822	CBYT1905.g1	GE284867
61928823	CBYT1906.g1	GE284868
61928824	CBYT1907.b1	GE284869
61928825	CBYT1907.g1	GE284870
61928826	CBYT1908.b1	GE284871
61928827	CBYT1908.g1	GE284872
61928828	CBYT1909.b1	GE284873
61928829	CBYT1909.g1	GE284874
61928830	CBYT1910.b1	GE284875
61928831	CBYT1910.g1	GE284876
61928832	CBYT1911.b1	GE284877
61928833	CBYT1911.g1	GE284878
61928834	CBYT1912.b1	GE284879
61928835	CBYT1912.g1	GE284880
61928836	CBYT1913.b1	GE284881
61928837	CBYT1913.g1	GE284882
61928838	CBYT1914.b1	GE284883
61928839	CBYT1914.g1	GE284884
61928840	CBYT1915.b1	GE284885
61928841	CBYT1915.g1	GE284886
61928842	CBYT1916.b1	GE284887
61928843	CBYT1916.g1	GE284888
61928844	CBYT1917.b1	GE284889
61928845	CBYT1917.g1	GE284890
61928846	CBYT1918.b1	GE284891
61928847	CBYT1918.g1	GE284892
61928848	CBYT1919.b1	GE284893
61928849	CBYT1919.g1	GE284894
61928850	CBYT1921.b1	GE284895
61928851	CBYT1922.b1	GE284896
61928852	CBYT1922.g1	GE284897
61928853	CBYT1923.b1	GE284898
61928854	CBYT1923.g1	GE284899
61928855	CBYT1924.b1	GE284900
61928856	CBYT1924.g1	GE284901
61928857	CBYT1925.b1	GE284902
61928858	CBYT1925.g1	GE284903
61928859	CBYT1926.b1	GE284904
61928860	CBYT1926.g1	GE284905
61928861	CBYT1927.g1	GE284906
61928862	CBYT1928.b1	GE284907
61928863	CBYT1929.b1	GE284908
61928864	CBYT1929.g1	GE284909
61928865	CBYT1930.b1	GE284910
61928866	CBYT1930.g1	GE284911
61928867	CBYT1931.b1	GE284912
61928868	CBYT1931.g1	GE284913
61928869	CBYT1932.b1	GE284914
61928870	CBYT1932.g1	GE284915
61928871	CBYT1933.b1	GE284916
61928872	CBYT1933.g1	GE284917
61928873	CBYT1935.b1	GE284918
61928874	CBYT1935.g1	GE284919
61928875	CBYT1936.b1	GE284920
61928876	CBYT1936.g1	GE284921
61928877	CBYT1937.b1	GE284922

61928878	CBYT1937.g1	GE284923
61928879	CBYT1938.b1	GE284924
61928880	CBYT1938.g1	GE284925
61928881	CBYT1939.g1	GE284926
61928882	CBYT1940.b1	GE284927
61928883	CBYT1940.g1	GE284928
61928884	CBYT1941.b1	GE284929
61928885	CBYT1941.g1	GE284930
61928886	CBYT1942.b1	GE284931
61928887	CBYT1943.b1	GE284932
61928888	CBYT1943.g1	GE284933
61928889	CBYT1944.b1	GE284934
61928890	CBYT1944.g1	GE284935
61928891	CBYT1945.b1	GE284936
61928892	CBYT1945.g1	GE284937
61928893	CBYT1947.b1	GE284938
61928894	CBYT1948.b1	GE284939
61928895	CBYT1948.g1	GE284940
61928896	CBYT1950.b1	GE284941
61928897	CBYT1950.g1	GE284942
61928898	CBYT1951.b1	GE284943
61928899	CBYT1951.g1	GE284944
61928900	CBYT1953.b1	GE284945
61928901	CBYT1953.g1	GE284946
61928902	CBYT1954.b1	GE284947
61928903	CBYT1954.g1	GE284948
61928904	CBYT1955.b1	GE284949
61928905	CBYT1955.g1	GE284950
61928906	CBYT1956.b1	GE284951
61928907	CBYT1956.g1	GE284952
61928908	CBYT1957.b1	GE284953
61928909	CBYT1957.g1	GE284954
61928910	CBYT1958.b1	GE284955
61928911	CBYT1958.g1	GE284956
61928912	CBYT1959.g1	GE284957
61928913	CBYT1960.b1	GE284958
61928914	CBYT1960.g1	GE284959
61928915	CBYT1961.b1	GE284960
61928916	CBYT1961.g1	GE284961
61928917	CBYT1964.b1	GE284962
61928918	CBYT1964.g1	GE284963
61928919	CBYT1965.b1	GE284964
61928920	CBYT1965.g1	GE284965
61928921	CBYT1966.b1	GE284966
61928922	CBYT1966.g1	GE284967
61928923	CBYT1967.b1	GE284968
61928924	CBYT1967.g1	GE284969
61928925	CBYT1968.b1	GE284970
61928926	CBYT1968.g1	GE284971
61928927	CBYT1969.b1	GE284972
61928928	CBYT1969.g1	GE284973
61928929	CBYT1970.b1	GE284974
61928930	CBYT1970.g1	GE284975
61928931	CBYT1971.b1	GE284976
61928932	CBYT1971.g1	GE284977
61928933	CBYT1972.b1	GE284978
61928934	CBYT1972.g1	GE284979
61928935	CBYT1973.b1	GE284980
61928936	CBYT1973.g1	GE284981
61928937	CBYT1974.b1	GE284982
61928938	CBYT1974.g1	GE284983
61928939	CBYT1975.b1	GE284984
61928940	CBYT1975.g1	GE284985

61928941	CBYT1976.b1	GE284986
61928942	CBYT1976.g1	GE284987
61928943	CBYT1977.b1	GE284988
61928944	CBYT1977.g1	GE284989
61928945	CBYT1978.b1	GE284990
61928946	CBYT1978.g1	GE284991
61928947	CBYT1979.b1	GE284992
61928948	CBYT1979.g1	GE284993
61928949	CBYT1980.b1	GE284994
61928950	CBYT1980.g1	GE284995
61928951	CBYT1982.g1	GE284996
61928952	CBYT1983.b1	GE284997
61928953	CBYT1983.g1	GE284998
61928954	CBYT1984.b1	GE284999
61928955	CBYT1984.g1	GE285000
61928956	CBYT1985.b1	GE285001
61928957	CBYT1986.b1	GE285002
61928958	CBYT1986.g1	GE285003
61928959	CBYT1987.b1	GE285004
61928960	CBYT1987.g1	GE285005
61928961	CBYT1988.b1	GE285006
61928962	CBYT1988.g1	GE285007
61928963	CBYT1989.b1	GE285008
61928964	CBYT1989.g1	GE285009
61928965	CBYT1990.b1	GE285010
61928966	CBYT1990.g1	GE285011
61928967	CBYT1991.b1	GE285012
61928968	CBYT1991.g1	GE285013
61928969	CBYT1992.b1	GE285014
61928970	CBYT1992.g1	GE285015
61928971	CBYT1993.b1	GE285016
61928972	CBYT1993.g1	GE285017
61928973	CBYT1994.b1	GE285018
61928974	CBYT1996.b1	GE285019
61928975	CBYT1997.b1	GE285020
61928976	CBYT1997.g1	GE285021
61928977	CBYT1998.b1	GE285022
61928978	CBYT1998.g1	GE285023
61928979	CBYT1999.b1	GE285024
61928980	CBYT1999.g1	GE285025
61928981	CBYT2000.b1	GE285026
61928982	CBYT2000.g1	GE285027
61928983	CBYT2002.b1	GE285028
61928984	CBYT2002.g1	GE285029
61928985	CBYT2003.b1	GE285030
61928986	CBYT2004.b1	GE285031
61928987	CBYT2005.b1	GE285032
61928988	CBYT2005.g1	GE285033
61928989	CBYT2008.g1	GE285034
61928990	CBYT2010.b1	GE285035
61928991	CBYT2011.b1	GE285036
61928992	CBYT2011.g1	GE285037
61928993	CBYT2012.b1	GE285038
61928994	CBYT2012.g1	GE285039
61928995	CBYT2013.b1	GE285040
61928996	CBYT2014.b1	GE285041
61928997	CBYT2014.g1	GE285042
61928998	CBYT2015.b1	GE285043
61928999	CBYT2015.g1	GE285044
61929000	CBYT2016.b1	GE285045
61929001	CBYT2016.g1	GE285046
61929002	CBYT2017.b1	GE285047
61929003	CBYT2017.g1	GE285048

61929004	CBYT2019.g1	GE285049
61929005	CBYT2020.b1	GE285050
61929006	CBYT2022.b1	GE285051
61929007	CBYT2024.b1	GE285052
61929008	CBYT2024.g1	GE285053
61929009	CBYT2026.b1	GE285054
61929010	CBYT2026.g1	GE285055
61929011	CBYT2027.b1	GE285056
61929012	CBYT2027.g1	GE285057
61929013	CBYT2028.b1	GE285058
61929014	CBYT2028.g1	GE285059
61929015	CBYT2029.b1	GE285060
61929016	CBYT2029.g1	GE285061
61929017	CBYT2031.b1	GE285062
61929018	CBYT2033.b1	GE285063
61929019	CBYT2033.g1	GE285064
61929020	CBYT2034.b1	GE285065
61929021	CBYT2034.g1	GE285066
61929022	CBYT2036.b1	GE285067
61929023	CBYT2036.g1	GE285068
61929024	CBYT2037.b1	GE285069
61929025	CBYT2037.g1	GE285070
61929026	CBYT2038.b1	GE285071
61929027	CBYT2038.g1	GE285072
61929028	CBYT2039.g1	GE285073
61929029	CBYT2040.b1	GE285074
61929030	CBYT2040.g1	GE285075
61929031	CBYT2041.b1	GE285076
61929032	CBYT2041.g1	GE285077
61929033	CBYT2043.g1	GE285078
61929034	CBYT2044.b1	GE285079
61929035	CBYT2044.g1	GE285080
61929036	CBYT2046.b1	GE285081
61929037	CBYT2046.g1	GE285082
61929038	CBYT2047.b1	GE285083
61929039	CBYT2047.g1	GE285084
61929040	CBYT2049.b1	GE285085
61929041	CBYT2049.g1	GE285086
61929042	CBYT2050.b1	GE285087
61929043	CBYT2050.g1	GE285088
61929044	CBYT2051.b1	GE285089
61929045	CBYT2051.g1	GE285090
61929046	CBYT2052.b1	GE285091
61929047	CBYT2052.g1	GE285092
61929048	CBYT2053.g1	GE285093
61929049	CBYT2054.b1	GE285094
61929050	CBYT2054.g1	GE285095
61929051	CBYT2055.b1	GE285096
61929052	CBYT2055.g1	GE285097
61929053	CBYT2056.b1	GE285098
61929054	CBYT2056.g1	GE285099
61929055	CBYT2057.b1	GE285100
61929056	CBYT2057.g1	GE285101
61929057	CBYT2058.b1	GE285102
61929058	CBYT2058.g1	GE285103
61929059	CBYT2060.g1	GE285104
61929060	CBYT2061.b1	GE285105
61929061	CBYT2061.g1	GE285106
61929062	CBYT2062.g1	GE285107
61929063	CBYT2063.b1	GE285108
61929064	CBYT2063.g1	GE285109
61929065	CBYT2064.g1	GE285110
61929066	CBYT2065.b1	GE285111

61929067	CBYT2065.g1	GE285112
61929068	CBYT2066.b1	GE285113
61929069	CBYT2066.g1	GE285114
61929070	CBYT2068.b1	GE285115
61929071	CBYT2068.g1	GE285116
61929072	CBYT2069.b1	GE285117
61929073	CBYT2069.g1	GE285118
61929074	CBYT2070.b1	GE285119
61929075	CBYT2070.g1	GE285120
61929076	CBYT2071.b1	GE285121
61929077	CBYT2071.g1	GE285122
61929078	CBYT2072.b1	GE285123
61929079	CBYT2072.g1	GE285124
61929080	CBYT2073.b1	GE285125
61929081	CBYT2073.g1	GE285126
61929082	CBYT2074.b1	GE285127
61929083	CBYT2074.g1	GE285128
61929084	CBYT2075.g1	GE285129
61929085	CBYT2076.b1	GE285130
61929086	CBYT2076.g1	GE285131
61929087	CBYT2078.b1	GE285132
61929088	CBYT2078.g1	GE285133
61929089	CBYT2079.b1	GE285134
61929090	CBYT2079.g1	GE285135
61929091	CBYT2080.b1	GE285136
61929092	CBYT2080.g1	GE285137
61929093	CBYT2081.b1	GE285138
61929094	CBYT2081.g1	GE285139
61929095	CBYT2082.b1	GE285140
61929096	CBYT2082.g1	GE285141
61929097	CBYT2083.b1	GE285142
61929098	CBYT2083.g1	GE285143
61929099	CBYT2084.b1	GE285144
61929100	CBYT2084.g1	GE285145
61929101	CBYT2085.b1	GE285146
61929102	CBYT2085.g1	GE285147
61929103	CBYT2086.b1	GE285148
61929104	CBYT2086.g1	GE285149
61929105	CBYT2087.b1	GE285150
61929106	CBYT2087.g1	GE285151
61929107	CBYT2088.b1	GE285152
61929108	CBYT2088.g1	GE285153
61929109	CBYT2089.b1	GE285154
61929110	CBYT2089.g1	GE285155
61929111	CBYT2090.b1	GE285156
61929112	CBYT2091.b1	GE285157
61929113	CBYT2092.b1	GE285158
61929114	CBYT2092.g1	GE285159
61929115	CBYT2093.b1	GE285160
61929116	CBYT2093.g1	GE285161
61929117	CBYT2094.g1	GE285162
61929118	CBYT2095.b1	GE285163
61929119	CBYT2095.g1	GE285164
61929120	CBYT2096.b1	GE285165
61929121	CBYT2096.g1	GE285166
61929122	CBYT2097.b1	GE285167
61929123	CBYT2097.g1	GE285168
61929124	CBYT2098.b1	GE285169
61929125	CBYT2098.g1	GE285170
61929126	CBYT2099.b1	GE285171
61929127	CBYT2099.g1	GE285172
61929128	CBYT2100.b1	GE285173
61929129	CBYT2100.g1	GE285174

61929130	CBYT2101.b1	GE285175
61929131	CBYT2103.b1	GE285176
61929132	CBYT2103.g1	GE285177
61929133	CBYT2104.b1	GE285178
61929134	CBYT2104.g1	GE285179
61929135	CBYT2106.b1	GE285180
61929136	CBYT2106.g1	GE285181
61929137	CBYT2107.b1	GE285182
61929138	CBYT2107.g1	GE285183
61929139	CBYT2108.b1	GE285184
61929140	CBYT2108.g1	GE285185
61929141	CBYT2109.b1	GE285186
61929142	CBYT2109.g1	GE285187
61929143	CBYT2110.b1	GE285188
61929144	CBYT2111.b1	GE285189
61929145	CBYT2111.g1	GE285190
61929146	CBYT2112.b1	GE285191
61929147	CBYT2113.b1	GE285192
61929148	CBYT2113.g1	GE285193
61929149	CBYT2114.b1	GE285194
61929150	CBYT2114.g1	GE285195
61929151	CBYT2115.b1	GE285196
61929152	CBYT2115.g1	GE285197
61929153	CBYT2116.b1	GE285198
61929154	CBYT2116.g1	GE285199
61929155	CBYT2117.b1	GE285200
61929156	CBYT2117.g1	GE285201
61929157	CBYT2118.b1	GE285202
61929158	CBYT2118.g1	GE285203
61929159	CBYT2119.b1	GE285204
61929160	CBYT2119.g1	GE285205
61929161	CBYT2120.b1	GE285206
61929162	CBYT2120.g1	GE285207
61929163	CBYT2121.b1	GE285208
61929164	CBYT2121.g1	GE285209
61929165	CBYT2122.b1	GE285210
61929166	CBYT2122.g1	GE285211
61929167	CBYT2123.b1	GE285212
61929168	CBYT2123.g1	GE285213
61929169	CBYT2124.b1	GE285214
61929170	CBYT2124.g1	GE285215
61929171	CBYT2125.b1	GE285216
61929172	CBYT2125.g1	GE285217
61929173	CBYT2126.b1	GE285218
61929174	CBYT2127.g1	GE285219
61929175	CBYT2129.b1	GE285220
61929176	CBYT2129.g1	GE285221
61929177	CBYT2130.b1	GE285222
61929178	CBYT2130.g1	GE285223
61929179	CBYT2131.b1	GE285224
61929180	CBYT2132.b1	GE285225
61929181	CBYT2132.g1	GE285226
61929182	CBYT2133.b1	GE285227
61929183	CBYT2133.g1	GE285228
61929184	CBYT2134.b1	GE285229
61929185	CBYT2135.b1	GE285230
61929186	CBYT2135.g1	GE285231
61929187	CBYT2136.b1	GE285232
61929188	CBYT2136.g1	GE285233
61929189	CBYT2137.b1	GE285234
61929190	CBYT2137.g1	GE285235
61929191	CBYT2139.g1	GE285236
61929192	CBYT2140.b1	GE285237

61929193	CBYT2140.g1	GE285238
61929194	CBYT2142.b1	GE285239
61929195	CBYT2142.g1	GE285240
61929196	CBYT2143.b1	GE285241
61929197	CBYT2143.g1	GE285242
61929198	CBYT2145.b1	GE285243
61929199	CBYT2145.g1	GE285244
61929200	CBYT2146.b1	GE285245
61929201	CBYT2146.g1	GE285246
61929202	CBYT2147.b1	GE285247
61929203	CBYT2147.g1	GE285248
61929204	CBYT2149.b1	GE285249
61929205	CBYT2149.g1	GE285250
61929206	CBYT2150.b1	GE285251
61929207	CBYT2150.g1	GE285252
61929208	CBYT2151.b1	GE285253
61929209	CBYT2151.g1	GE285254
61929210	CBYT2152.b1	GE285255
61929211	CBYT2152.g1	GE285256
61929212	CBYT2153.b1	GE285257
61929213	CBYT2153.g1	GE285258
61929214	CBYT2154.b1	GE285259
61929215	CBYT2154.g1	GE285260
61929216	CBYT2156.g1	GE285261
61929217	CBYT2157.b1	GE285262
61929218	CBYT2157.g1	GE285263
61929219	CBYT2158.b1	GE285264
61929220	CBYT2158.g1	GE285265
61929221	CBYT2159.b1	GE285266
61929222	CBYT2159.g1	GE285267
61929223	CBYT2160.b1	GE285268
61929224	CBYT2160.g1	GE285269
61929225	CBYT2161.g1	GE285270
61929226	CBYT2162.b1	GE285271
61929227	CBYT2162.g1	GE285272
61929228	CBYT2163.b1	GE285273
61929229	CBYT2163.g1	GE285274
61929230	CBYT2164.g1	GE285275
61929231	CBYT2165.b1	GE285276
61929232	CBYT2165.g1	GE285277
61929233	CBYT2166.b1	GE285278
61929234	CBYT2166.g1	GE285279
61929235	CBYT2167.b1	GE285280
61929236	CBYT2167.g1	GE285281
61929237	CBYT2168.b1	GE285282
61929238	CBYT2168.g1	GE285283
61929239	CBYT2169.b1	GE285284
61929240	CBYT2169.g1	GE285285
61929241	CBYT2170.b1	GE285286
61929242	CBYT2170.g1	GE285287
61929243	CBYT2171.b1	GE285288
61929244	CBYT2171.g1	GE285289
61929245	CBYT2172.b1	GE285290
61929246	CBYT2172.g1	GE285291
61929247	CBYT2174.b1	GE285292
61929248	CBYT2174.g1	GE285293
61929249	CBYT2175.b1	GE285294
61929250	CBYT2175.g1	GE285295
61929251	CBYT2176.b1	GE285296
61929252	CBYT2176.g1	GE285297
61929253	CBYT2177.b1	GE285298
61929254	CBYT2177.g1	GE285299
61929255	CBYT2178.b1	GE285300

61929256	CBYT2178.g1	GE285301
61929257	CBYT2179.b1	GE285302
61929258	CBYT2179.g1	GE285303
61929259	CBYT2180.b1	GE285304
61929260	CBYT2180.g1	GE285305
61929261	CBYT2181.b1	GE285306
61929262	CBYT2181.g1	GE285307
61929263	CBYT2182.b1	GE285308
61929264	CBYT2182.g1	GE285309
61929265	CBYT2183.b1	GE285310
61929266	CBYT2183.g1	GE285311
61929267	CBYT2184.b1	GE285312
61929268	CBYT2184.g1	GE285313
61929269	CBYT2186.b1	GE285314
61929270	CBYT2187.b1	GE285315
61929271	CBYT2187.g1	GE285316
61929272	CBYT2188.b1	GE285317
61929273	CBYT2188.g1	GE285318
61929274	CBYT2190.b1	GE285319
61929275	CBYT2190.g1	GE285320
61929276	CBYT2191.b1	GE285321
61929277	CBYT2191.g1	GE285322
61929278	CBYT2192.b1	GE285323
61929279	CBYT2192.g1	GE285324
61929280	CBYT2193.b1	GE285325
61929281	CBYT2194.b1	GE285326
61929282	CBYT2194.g1	GE285327
61929283	CBYT2195.b1	GE285328
61929284	CBYT2195.g1	GE285329
61929285	CBYT2196.b1	GE285330
61929286	CBYT2196.g1	GE285331
61929287	CBYT2197.g1	GE285332
61929288	CBYT2199.b1	GE285333
61929289	CBYT2199.g1	GE285334
61929290	CBYT2200.g1	GE285335
61929291	CBYT2201.b1	GE285336
61929292	CBYT2202.b1	GE285337
61929293	CBYT2202.g1	GE285338
61929294	CBYT2203.b1	GE285339
61929295	CBYT2203.g1	GE285340
61929296	CBYT2204.b1	GE285341
61929297	CBYT2204.g1	GE285342
61929298	CBYT2205.b1	GE285343
61929299	CBYT2205.g1	GE285344
61929300	CBYT2206.b1	GE285345
61929301	CBYT2208.g1	GE285346
61929302	CBYT2209.b1	GE285347
61929303	CBYT2209.g1	GE285348
61929304	CBYT2210.g1	GE285349
61929305	CBYT2211.b1	GE285350
61929306	CBYT2212.b1	GE285351
61929307	CBYT2212.g1	GE285352
61929308	CBYT2213.b1	GE285353
61929309	CBYT2214.b1	GE285354
61929310	CBYT2214.g1	GE285355
61929311	CBYT2215.b1	GE285356
61929312	CBYT2215.g1	GE285357
61929313	CBYT2216.b1	GE285358
61929314	CBYT2216.g1	GE285359
61929315	CBYT2217.b1	GE285360
61929316	CBYT2217.g1	GE285361
61929317	CBYT2218.b1	GE285362
61929318	CBYT2219.b1	GE285363

61929319	CBYT2219.g1	GE285364
61929320	CBYT2220.b1	GE285365
61929321	CBYT2220.g1	GE285366
61929322	CBYT2221.b1	GE285367
61929323	CBYT2222.b1	GE285368
61929324	CBYT2223.b1	GE285369
61929325	CBYT2223.g1	GE285370
61929326	CBYT2224.b1	GE285371
61929327	CBYT2224.g1	GE285372
61929328	CBYT2225.b1	GE285373
61929329	CBYT2225.g1	GE285374
61929330	CBYT2226.b1	GE285375
61929331	CBYT2226.g1	GE285376
61929332	CBYT2228.b1	GE285377
61929333	CBYT2228.g1	GE285378
61929334	CBYT2229.b1	GE285379
61929335	CBYT2230.b1	GE285380
61929336	CBYT2230.g1	GE285381
61929337	CBYT2231.b1	GE285382
61929338	CBYT2231.g1	GE285383
61929339	CBYT2232.b1	GE285384
61929340	CBYT2233.g1	GE285385
61929341	CBYT2234.b1	GE285386
61929342	CBYT2235.b1	GE285387
61929343	CBYT2236.b1	GE285388
61929344	CBYT2237.g1	GE285389
61929345	CBYT2239.b1	GE285390
61929346	CBYT2239.g1	GE285391
61929347	CBYT2241.b1	GE285392
61929348	CBYT2241.g1	GE285393
61929349	CBYT2242.b1	GE285394
61929350	CBYT2242.g1	GE285395
61929351	CBYT2243.b1	GE285396
61929352	CBYT2243.g1	GE285397
61929353	CBYT2244.b1	GE285398
61929354	CBYT2244.g1	GE285399
61929355	CBYT2245.b1	GE285400
61929356	CBYT2245.g1	GE285401
61929357	CBYT2246.b1	GE285402
61929358	CBYT2246.g1	GE285403
61929359	CBYT2247.g1	GE285404
61929360	CBYT2248.b1	GE285405
61929361	CBYT2248.g1	GE285406
61929362	CBYT2249.b1	GE285407
61929363	CBYT2249.g1	GE285408
61929364	CBYT2250.b1	GE285409
61929365	CBYT2250.g1	GE285410
61929366	CBYT2253.b1	GE285411
61929367	CBYT2253.g1	GE285412
61929368	CBYT2254.b1	GE285413
61929369	CBYT2254.g1	GE285414
61929370	CBYT2255.b1	GE285415
61929371	CBYT2255.g1	GE285416
61929372	CBYT2256.b1	GE285417
61929373	CBYT2256.g1	GE285418
61929374	CBYT2257.b1	GE285419
61929375	CBYT2257.g1	GE285420
61929376	CBYT2258.b1	GE285421
61929377	CBYT2258.g1	GE285422
61929378	CBYT2259.b1	GE285423
61929379	CBYT2259.g1	GE285424
61929380	CBYT2260.b1	GE285425
61929381	CBYT2260.g1	GE285426

61929382	CBYT2261.b1	GE285427
61929383	CBYT2261.g1	GE285428
61929384	CBYT2262.b1	GE285429
61929385	CBYT2263.b1	GE285430
61929386	CBYT2263.g1	GE285431
61929387	CBYT2264.b1	GE285432
61929388	CBYT2264.g1	GE285433
61929389	CBYT2265.b1	GE285434
61929390	CBYT2265.g1	GE285435
61929391	CBYT2266.b1	GE285436
61929392	CBYT2266.g1	GE285437
61929393	CBYT2267.g1	GE285438
61929394	CBYT2268.b1	GE285439
61929395	CBYT2268.g1	GE285440
61929396	CBYT2270.b1	GE285441
61929397	CBYT2270.g1	GE285442
61929398	CBYT2271.b1	GE285443
61929399	CBYT2271.g1	GE285444
61929400	CBYT2272.b1	GE285445
61929401	CBYT2272.g1	GE285446
61929402	CBYT2273.b1	GE285447
61929403	CBYT2273.g1	GE285448
61929404	CBYT2274.b1	GE285449
61929405	CBYT2275.b1	GE285450
61929406	CBYT2275.g1	GE285451
61929407	CBYT2276.b1	GE285452
61929408	CBYT2276.g1	GE285453
61929409	CBYT2277.b1	GE285454
61929410	CBYT2277.g1	GE285455
61929411	CBYT2279.b1	GE285456
61929412	CBYT2279.g1	GE285457
61929413	CBYT2280.b1	GE285458
61929414	CBYT2280.g1	GE285459
61929415	CBYT2281.b1	GE285460
61929416	CBYT2281.g1	GE285461
61929417	CBYT2282.b1	GE285462
61929418	CBYT2283.b1	GE285463
61929419	CBYT2283.g1	GE285464
61929420	CBYT2284.b1	GE285465
61929421	CBYT2284.g1	GE285466
61929422	CBYT2285.b1	GE285467
61929423	CBYT2285.g1	GE285468
61929424	CBYT2286.b1	GE285469
61929425	CBYT2286.g1	GE285470
61929426	CBYT2287.b1	GE285471
61929427	CBYT2287.g1	GE285472
61929428	CBYT2288.b1	GE285473
61929429	CBYT2288.g1	GE285474
61929430	CBYT2289.b1	GE285475
61929431	CBYT2289.g1	GE285476
61929432	CBYT2290.b1	GE285477
61929433	CBYT2290.g1	GE285478
61929434	CBYT2291.b1	GE285479
61929435	CBYT2291.g1	GE285480
61929436	CBYT2292.b1	GE285481
61929437	CBYT2292.g1	GE285482
61929438	CBYT2295.b1	GE285483
61929439	CBYT2295.g1	GE285484
61929440	CBYT2298.b1	GE285485
61929441	CBYT2298.g1	GE285486
61929442	CBYT2300.b1	GE285487
61929443	CBYT2300.g1	GE285488
61929444	CBYT2301.b1	GE285489

61929445	CBYT2301.g1	GE285490
61929446	CBYT2305.b1	GE285491
61929447	CBYT2306.b1	GE285492
61929448	CBYT2306.g1	GE285493
61929449	CBYT2307.b1	GE285494
61929450	CBYT2307.g1	GE285495
61929451	CBYT2308.b1	GE285496
61929452	CBYT2308.g1	GE285497
61929453	CBYT2309.b1	GE285498
61929454	CBYT2309.g1	GE285499
61929455	CBYT2310.b1	GE285500
61929456	CBYT2311.b1	GE285501
61929457	CBYT2313.b1	GE285502
61929458	CBYT2313.g1	GE285503
61929459	CBYT2314.b1	GE285504
61929460	CBYT2314.g1	GE285505
61929461	CBYT2315.b1	GE285506
61929462	CBYT2315.g1	GE285507
61929463	CBYT2316.b1	GE285508
61929464	CBYT2316.g1	GE285509
61929465	CBYT2317.b1	GE285510
61929466	CBYT2317.g1	GE285511
61929467	CBYT2318.b1	GE285512
61929468	CBYT2318.g1	GE285513
61929469	CBYT2319.b1	GE285514
61929470	CBYT2319.g1	GE285515
61929471	CBYT2320.b1	GE285516
61929472	CBYT2320.g1	GE285517
61929473	CBYT2321.g1	GE285518
61929474	CBYT2323.b1	GE285519
61929475	CBYT2323.g1	GE285520
61929476	CBYT2324.b1	GE285521
61929477	CBYT2324.g1	GE285522
61929478	CBYT2325.b1	GE285523
61929479	CBYT2325.g1	GE285524
61929480	CBYT2326.g1	GE285525
61929481	CBYT2327.b1	GE285526
61929482	CBYT2327.g1	GE285527
61929483	CBYT2328.b1	GE285528
61929484	CBYT2328.g1	GE285529
61929485	CBYT2329.b1	GE285530
61929486	CBYT2329.g1	GE285531
61929487	CBYT2330.b1	GE285532
61929488	CBYT2330.g1	GE285533
61929489	CBYT2332.g1	GE285534
61929490	CBYT2333.b1	GE285535
61929491	CBYT2333.g1	GE285536
61929492	CBYT2334.b1	GE285537
61929493	CBYT2334.g1	GE285538
61929494	CBYT2336.g1	GE285539
61929495	CBYT2337.b1	GE285540
61929496	CBYT2337.g1	GE285541
61929497	CBYT2338.b1	GE285542
61929498	CBYT2338.g1	GE285543
61929499	CBYT2339.b1	GE285544
61929500	CBYT2339.g1	GE285545
61929501	CBYT2340.b1	GE285546
61929502	CBYT2340.g1	GE285547
61929503	CBYT2341.g1	GE285548
61929504	CBYT2342.b1	GE285549
61929505	CBYT2343.b1	GE285550
61929506	CBYT2343.g1	GE285551
61929507	CBYT2344.b1	GE285552

61929508	CBYT2344.g1	GE285553
61929509	CBYT2345.g1	GE285554
61929510	CBYT2346.b1	GE285555
61929511	CBYT2346.g1	GE285556
61929512	CBYT2347.b1	GE285557
61929513	CBYT2347.g1	GE285558
61929514	CBYT2348.b1	GE285559
61929515	CBYT2348.g1	GE285560
61929516	CBYT2349.b1	GE285561
61929517	CBYT2349.g1	GE285562
61929518	CBYT2350.b1	GE285563
61929519	CBYT2350.g1	GE285564
61929520	CBYT2351.b1	GE285565
61929521	CBYT2351.g1	GE285566
61929522	CBYT2352.b1	GE285567
61929523	CBYT2352.g1	GE285568
61929524	CBYT2353.b1	GE285569
61929525	CBYT2353.g1	GE285570
61929526	CBYT2355.g1	GE285571
61929527	CBYT2356.g1	GE285572
61929528	CBYT2357.b1	GE285573
61929529	CBYT2357.g1	GE285574
61929530	CBYT2358.b1	GE285575
61929531	CBYT2358.g1	GE285576
61929532	CBYT2361.b1	GE285577
61929533	CBYT2361.g1	GE285578
61929534	CBYT2362.b1	GE285579
61929535	CBYT2362.g1	GE285580
61929536	CBYT2363.b1	GE285581
61929537	CBYT2363.g1	GE285582
61929538	CBYT2364.b1	GE285583
61929539	CBYT2364.g1	GE285584
61929540	CBYT2366.b1	GE285585
61929541	CBYT2366.g1	GE285586
61929542	CBYT2367.b1	GE285587
61929543	CBYT2367.g1	GE285588
61929544	CBYT2368.b1	GE285589
61929545	CBYT2368.g1	GE285590
61929546	CBYT2369.b1	GE285591
61929547	CBYT2369.g1	GE285592
61929548	CBYT2370.b1	GE285593
61929549	CBYT2370.g1	GE285594
61929550	CBYT2371.b1	GE285595
61929551	CBYT2371.g1	GE285596
61929552	CBYT2372.g1	GE285597
61929553	CBYT2373.b1	GE285598
61929554	CBYT2373.g1	GE285599
61929555	CBYT2374.b1	GE285600
61929556	CBYT2374.g1	GE285601
61929557	CBYT2375.b1	GE285602
61929558	CBYT2375.g1	GE285603
61929559	CBYT2376.g1	GE285604
61929560	CBYT2377.b1	GE285605
61929561	CBYT2377.g1	GE285606
61929562	CBYT2378.b1	GE285607
61929563	CBYT2378.g1	GE285608
61929564	CBYT2379.b1	GE285609
61929565	CBYT2379.g1	GE285610
61929566	CBYT2380.b1	GE285611
61929567	CBYT2380.g1	GE285612
61929568	CBYT2381.b1	GE285613
61929569	CBYT2381.g1	GE285614
61929570	CBYT2382.b1	GE285615

61929571	CBYT2382.g1	GE285616
61929572	CBYT2383.b1	GE285617
61929573	CBYT2383.g1	GE285618
61929574	CBYT2384.b1	GE285619
61929575	CBYT2384.g1	GE285620
61929576	CBYT2385.b1	GE285621
61929577	CBYT2385.g1	GE285622
61929578	CBYT2386.b1	GE285623
61929579	CBYT2386.g1	GE285624
61929580	CBYT2387.b1	GE285625
61929581	CBYT2387.g1	GE285626
61929582	CBYT2388.b1	GE285627
61929583	CBYT2388.g1	GE285628
61929584	CBYT2389.b1	GE285629
61929585	CBYT2389.g1	GE285630
61929586	CBYT2390.b1	GE285631
61929587	CBYT2390.g1	GE285632
61929588	CBYT2391.b1	GE285633
61929589	CBYT2391.g1	GE285634
61929590	CBYT2392.b1	GE285635
61929591	CBYT2392.g1	GE285636
61929592	CBYT2393.b1	GE285637
61929593	CBYT2393.g1	GE285638
61929594	CBYT2394.b1	GE285639
61929595	CBYT2394.g1	GE285640
61929596	CBYT2395.g1	GE285641
61929597	CBYT2396.b1	GE285642
61929598	CBYT2396.g1	GE285643
61929599	CBYT2397.b1	GE285644
61929600	CBYT2397.g1	GE285645
61929601	CBYT2399.g1	GE285646
61929602	CBYT2400.b1	GE285647
61929603	CBYT2400.g1	GE285648
61929604	CBYT2401.b1	GE285649
61929605	CBYT2401.g1	GE285650
61929606	CBYT2402.b1	GE285651
61929607	CBYT2402.g1	GE285652
61929608	CBYT2403.b1	GE285653
61929609	CBYT2404.b1	GE285654
61929610	CBYT2404.g1	GE285655
61929611	CBYT2405.b1	GE285656
61929612	CBYT2405.g1	GE285657
61929613	CBYT2406.b1	GE285658
61929614	CBYT2407.b1	GE285659
61929615	CBYT2407.g1	GE285660
61929616	CBYT2408.b1	GE285661
61929617	CBYT2408.g1	GE285662
61929618	CBYT2410.b1	GE285663
61929619	CBYT2410.g1	GE285664
61929620	CBYT2411.b1	GE285665
61929621	CBYT2411.g1	GE285666
61929622	CBYT2412.b1	GE285667
61929623	CBYT2412.g1	GE285668
61929624	CBYT2413.b1	GE285669
61929625	CBYT2413.g1	GE285670
61929626	CBYT2414.g1	GE285671
61929627	CBYT2416.b1	GE285672
61929628	CBYT2416.g1	GE285673
61929629	CBYT2417.b1	GE285674
61929630	CBYT2418.b1	GE285675
61929631	CBYT2418.g1	GE285676
61929632	CBYT2420.b1	GE285677
61929633	CBYT2421.b1	GE285678

61929634	CBYT2423.b1	GE285679
61929635	CBYT2423.g1	GE285680
61929636	CBYT2424.b1	GE285681
61929637	CBYT2424.g1	GE285682
61929638	CBYT2426.b1	GE285683
61929639	CBYT2426.g1	GE285684
61929640	CBYT2427.b1	GE285685
61929641	CBYT2427.g1	GE285686
61929642	CBYT2429.b1	GE285687
61929643	CBYT2429.g1	GE285688
61929644	CBYT2430.b1	GE285689
61929645	CBYT2430.g1	GE285690
61929646	CBYT2431.b1	GE285691
61929647	CBYT2431.g1	GE285692
61929648	CBYT2432.g1	GE285693
61929649	CBYT2433.b1	GE285694
61929650	CBYT2433.g1	GE285695
61929651	CBYT2434.b1	GE285696
61929652	CBYT2434.g1	GE285697
61929653	CBYT2435.b1	GE285698
61929654	CBYT2435.g1	GE285699
61929655	CBYT2436.b1	GE285700
61929656	CBYT2436.g1	GE285701
61929657	CBYT2437.g1	GE285702
61929658	CBYT2438.b1	GE285703
61929659	CBYT2438.g1	GE285704
61929660	CBYT2439.b1	GE285705
61929661	CBYT2439.g1	GE285706
61929662	CBYT2440.b1	GE285707
61929663	CBYT2440.g1	GE285708
61929664	CBYT2441.b1	GE285709
61929665	CBYT2441.g1	GE285710
61929666	CBYT2442.b1	GE285711
61929667	CBYT2442.g1	GE285712
61929668	CBYT2443.b1	GE285713
61929669	CBYT2443.g1	GE285714
61929670	CBYT2444.b1	GE285715
61929671	CBYT2444.g1	GE285716
61929672	CBYT2445.b1	GE285717
61929673	CBYT2445.g1	GE285718
61929674	CBYT2446.b1	GE285719
61929675	CBYT2446.g1	GE285720
61929676	CBYT2447.b1	GE285721
61929677	CBYT2447.g1	GE285722
61929678	CBYT2448.b1	GE285723
61929679	CBYT2448.g1	GE285724
61929680	CBYT2449.b1	GE285725
61929681	CBYT2449.g1	GE285726
61929682	CBYT2450.b1	GE285727
61929683	CBYT2451.b1	GE285728
61929684	CBYT2452.b1	GE285729
61929685	CBYT2452.g1	GE285730
61929686	CBYT2453.b1	GE285731
61929687	CBYT2454.g1	GE285732
61929688	CBYT2455.b1	GE285733
61929689	CBYT2455.g1	GE285734
61929690	CBYT2456.b1	GE285735
61929691	CBYT2456.g1	GE285736
61929692	CBYT2457.b1	GE285737
61929693	CBYT2457.g1	GE285738
61929694	CBYT2458.b1	GE285739
61929695	CBYT2458.g1	GE285740
61929696	CBYT2459.b1	GE285741

61929697	CBYT2459.g1	GE285742
61929698	CBYT2460.b1	GE285743
61929699	CBYT2460.g1	GE285744
61929700	CBYT2461.b1	GE285745
61929701	CBYT2461.g1	GE285746
61929702	CBYT2462.b1	GE285747
61929703	CBYT2462.g1	GE285748
61929704	CBYT2463.g1	GE285749
61929705	CBYT2464.b1	GE285750
61929706	CBYT2464.g1	GE285751
61929707	CBYT2465.b1	GE285752
61929708	CBYT2465.g1	GE285753
61929709	CBYT2466.b1	GE285754
61929710	CBYT2466.g1	GE285755
61929711	CBYT2467.b1	GE285756
61929712	CBYT2467.g1	GE285757
61929713	CBYT2468.b1	GE285758
61929714	CBYT2468.g1	GE285759
61929715	CBYT2469.b1	GE285760
61929716	CBYT2469.g1	GE285761
61929717	CBYT2471.b1	GE285762
61929718	CBYT2471.g1	GE285763
61929719	CBYT2472.g1	GE285764
61929720	CBYT2473.b1	GE285765
61929721	CBYT2473.g1	GE285766
61929722	CBYT2474.b1	GE285767
61929723	CBYT2474.g1	GE285768
61929724	CBYT2475.b1	GE285769
61929725	CBYT2475.g1	GE285770
61929726	CBYT2476.b1	GE285771
61929727	CBYT2476.g1	GE285772
61929728	CBYT2477.b1	GE285773
61929729	CBYT2477.g1	GE285774
61929730	CBYT2478.b1	GE285775
61929731	CBYT2478.g1	GE285776
61929732	CBYT2479.b1	GE285777
61929733	CBYT2479.g1	GE285778
61929734	CBYT2480.b1	GE285779
61929735	CBYT2480.g1	GE285780
61929736	CBYT2481.b1	GE285781
61929737	CBYT2482.b1	GE285782
61929738	CBYT2482.g1	GE285783
61929739	CBYT2483.b1	GE285784
61929740	CBYT2483.g1	GE285785
61929741	CBYT2484.b1	GE285786
61929742	CBYT2484.g1	GE285787
61929743	CBYT2485.b1	GE285788
61929744	CBYT2485.g1	GE285789
61929745	CBYT2486.b1	GE285790
61929746	CBYT2486.g1	GE285791
61929747	CBYT2487.b1	GE285792
61929748	CBYT2487.g1	GE285793
61929749	CBYT2488.b1	GE285794
61929750	CBYT2488.g1	GE285795
61929751	CBYT2489.b1	GE285796
61929752	CBYT2489.g1	GE285797
61929753	CBYT2491.b1	GE285798
61929754	CBYT2491.g1	GE285799
61929755	CBYT2492.b1	GE285800
61929756	CBYT2492.g1	GE285801
61929757	CBYT2494.b1	GE285802
61929758	CBYT2495.b1	GE285803
61929759	CBYT2495.g1	GE285804

61929760	CBYT2496.b1	GE285805
61929761	CBYT2496.g1	GE285806
61929762	CBYT2497.b1	GE285807
61929763	CBYT2497.g1	GE285808
61929764	CBYT2498.b1	GE285809
61929765	CBYT2498.g1	GE285810
61929766	CBYT2499.b1	GE285811
61929767	CBYT2499.g1	GE285812
61929768	CBYT2500.b1	GE285813
61929769	CBYT2501.b1	GE285814
61929770	CBYT2501.g1	GE285815
61929771	CBYT2502.b1	GE285816
61929772	CBYT2502.g1	GE285817
61929773	CBYT2503.b1	GE285818
61929774	CBYT2503.g1	GE285819
61929775	CBYT2504.b1	GE285820
61929776	CBYT2504.g1	GE285821
61929777	CBYT2506.b1	GE285822
61929778	CBYT2507.b1	GE285823
61929779	CBYT2507.g1	GE285824
61929780	CBYT2508.b1	GE285825
61929781	CBYT2508.g1	GE285826
61929782	CBYT2511.b1	GE285827
61929783	CBYT2511.g1	GE285828
61929784	CBYT2512.b1	GE285829
61929785	CBYT2512.g1	GE285830
61929786	CBYT2513.b1	GE285831
61929787	CBYT2513.g1	GE285832
61929788	CBYT2514.b1	GE285833
61929789	CBYT2514.g1	GE285834
61929790	CBYT2515.b1	GE285835
61929791	CBYT2515.g1	GE285836
61929792	CBYT2516.b1	GE285837
61929793	CBYT2517.b1	GE285838
61929794	CBYT2519.b1	GE285839
61929795	CBYT2519.g1	GE285840
61929796	CBYT2520.b1	GE285841
61929797	CBYT2520.g1	GE285842
61929798	CBYT2521.b1	GE285843
61929799	CBYT2521.g1	GE285844
61929800	CBYT2522.b1	GE285845
61929801	CBYT2522.g1	GE285846
61929802	CBYT2523.b1	GE285847
61929803	CBYT2524.g1	GE285848
61929804	CBYT2525.b1	GE285849
61929805	CBYT2525.g1	GE285850
61929806	CBYT2526.b1	GE285851
61929807	CBYT2526.g1	GE285852
61929808	CBYT2527.b1	GE285853
61929809	CBYT2527.g1	GE285854
61929810	CBYT2530.b1	GE285855
61929811	CBYT2530.g1	GE285856
61929812	CBYT2531.b1	GE285857
61929813	CBYT2531.g1	GE285858
61929814	CBYT2532.b1	GE285859
61929815	CBYT2532.g1	GE285860
61929816	CBYT2534.b1	GE285861
61929817	CBYT2534.g1	GE285862
61929818	CBYT2535.b1	GE285863
61929819	CBYT2535.g1	GE285864
61929820	CBYT2537.b1	GE285865
61929821	CBYT2537.g1	GE285866
61929822	CBYT2538.b1	GE285867

61929823	CBYT2538.g1	GE285868
61929824	CBYT2539.b1	GE285869
61929825	CBYT2539.g1	GE285870
61929826	CBYT2540.b1	GE285871
61929827	CBYT2540.g1	GE285872
61929828	CBYT2541.b1	GE285873
61929829	CBYT2541.g1	GE285874
61929830	CBYT2542.b1	GE285875
61929831	CBYT2542.g1	GE285876
61929832	CBYT2543.b1	GE285877
61929833	CBYT2543.g1	GE285878
61929834	CBYT2544.g1	GE285879
61929835	CBYT2545.b1	GE285880
61929836	CBYT2545.g1	GE285881
61929837	CBYT2546.b1	GE285882
61929838	CBYT2547.b1	GE285883
61929839	CBYT2548.b1	GE285884
61929840	CBYT2548.g1	GE285885
61929841	CBYT2549.b1	GE285886
61929842	CBYT2549.g1	GE285887
61929843	CBYT2550.b1	GE285888
61929844	CBYT2550.g1	GE285889
61929845	CBYT2551.b1	GE285890
61929846	CBYT2551.g1	GE285891
61929847	CBYT2552.b1	GE285892
61929848	CBYT2552.g1	GE285893
61929849	CBYT2553.b1	GE285894
61929850	CBYT2553.g1	GE285895
61929851	CBYT2554.b1	GE285896
61929852	CBYT2554.g1	GE285897
61929853	CBYT2555.b1	GE285898
61929854	CBYT2555.g1	GE285899
61929855	CBYT2556.b1	GE285900
61929856	CBYT2556.g1	GE285901
61929857	CBYT2558.b1	GE285902
61929858	CBYT2560.b1	GE285903
61929859	CBYT2560.g1	GE285904
61929860	CBYT2561.b1	GE285905
61929861	CBYT2561.g1	GE285906
61929862	CBYT2562.b1	GE285907
61929863	CBYT2563.g1	GE285908
61929864	CBYT2564.b1	GE285909
61929865	CBYT2564.g1	GE285910
61929866	CBYT2566.b1	GE285911
61929867	CBYT2566.g1	GE285912
61929868	CBYT2567.b1	GE285913
61929869	CBYT2567.g1	GE285914
61929870	CBYT2568.b1	GE285915
61929871	CBYT2568.g1	GE285916
61929872	CBYT2570.b1	GE285917
61929873	CBYT2570.g1	GE285918
61929874	CBYT2571.b1	GE285919
61929875	CBYT2571.g1	GE285920
61929876	CBYT2572.b1	GE285921
61929877	CBYT2572.g1	GE285922
61929878	CBYT2573.b1	GE285923
61929879	CBYT2573.g1	GE285924
61929880	CBYT2574.b1	GE285925
61929881	CBYT2574.g1	GE285926
61929882	CBYT2575.b1	GE285927
61929883	CBYT2575.g1	GE285928
61929884	CBYT2576.b1	GE285929
61929885	CBYT2576.g1	GE285930

61929886	CBYT2577.b1	GE285931
61929887	CBYT2577.g1	GE285932
61929888	CBYT2578.b1	GE285933
61929889	CBYT2578.g1	GE285934
61929890	CBYT2579.b1	GE285935
61929891	CBYT2579.g1	GE285936
61929892	CBYT2580.b1	GE285937
61929893	CBYT2581.b1	GE285938
61929894	CBYT2581.g1	GE285939
61929895	CBYT2582.b1	GE285940
61929896	CBYT2583.b1	GE285941
61929897	CBYT2583.g1	GE285942
61929898	CBYT2584.b1	GE285943
61929899	CBYT2584.g1	GE285944
61929900	CBYT2585.b1	GE285945
61929901	CBYT2585.g1	GE285946
61929902	CBYT2587.b1	GE285947
61929903	CBYT2587.g1	GE285948
61929904	CBYT2588.b1	GE285949
61929905	CBYT2588.g1	GE285950
61929906	CBYT2589.b1	GE285951
61929907	CBYT2590.b1	GE285952
61929908	CBYT2590.g1	GE285953
61929909	CBYT2591.b1	GE285954
61929910	CBYT2591.g1	GE285955
61929911	CBYT2593.b1	GE285956
61929912	CBYT2593.g1	GE285957
61929913	CBYT2594.b1	GE285958
61929914	CBYT2594.g1	GE285959
61929915	CBYT2595.b1	GE285960
61929916	CBYT2595.g1	GE285961
61929917	CBYT2596.g1	GE285962
61929918	CBYT2597.b1	GE285963
61929919	CBYT2597.g1	GE285964
61929920	CBYT2598.b1	GE285965
61929921	CBYT2598.g1	GE285966
61929922	CBYT2600.g1	GE285967
61929923	CBYT2601.b1	GE285968
61929924	CBYT2601.g1	GE285969
61929925	CBYT2602.b1	GE285970
61929926	CBYT2602.g1	GE285971
61929927	CBYT2603.b1	GE285972
61929928	CBYT2603.g1	GE285973
61929929	CBYT2604.b1	GE285974
61929930	CBYT2604.g1	GE285975
61929931	CBYT2605.b1	GE285976
61929932	CBYT2605.g1	GE285977
61929933	CBYT2606.g1	GE285978
61929934	CBYT2607.b1	GE285979
61929935	CBYT2607.g1	GE285980
61929936	CBYT2608.b1	GE285981
61929937	CBYT2608.g1	GE285982
61929938	CBYT2609.b1	GE285983
61929939	CBYT2609.g1	GE285984
61929940	CBYT2610.g1	GE285985
61929941	CBYT2611.b1	GE285986
61929942	CBYT2612.b1	GE285987
61929943	CBYT2612.g1	GE285988
61929944	CBYT2613.b1	GE285989
61929945	CBYT2613.g1	GE285990
61929946	CBYT2616.b1	GE285991
61929947	CBYT2616.g1	GE285992
61929948	CBYT2617.b1	GE285993

61929949	CBYT2617.g1	GE285994
61929950	CBYT2618.b1	GE285995
61929951	CBYT2618.g1	GE285996
61929952	CBYT2621.b1	GE285997
61929953	CBYT2622.b1	GE285998
61929954	CBYT2622.g1	GE285999
61929955	CBYT2623.g1	GE286000
61929956	CBYT2625.b1	GE286001
61929957	CBYT2625.g1	GE286002
61929958	CBYT2626.b1	GE286003
61929959	CBYT2626.g1	GE286004
61929960	CBYT2627.b1	GE286005
61929961	CBYT2627.g1	GE286006
61929962	CBYT2628.b1	GE286007
61929963	CBYT2628.g1	GE286008
61929964	CBYT2631.b1	GE286009
61929965	CBYT2631.g1	GE286010
61929966	CBYT2632.b1	GE286011
61929967	CBYT2632.g1	GE286012
61929968	CBYT2633.b1	GE286013
61929969	CBYT2633.g1	GE286014
61929970	CBYT2634.g1	GE286015
61929971	CBYT2635.b1	GE286016
61929972	CBYT2635.g1	GE286017
61929973	CBYT2636.b1	GE286018
61929974	CBYT2636.g1	GE286019
61929975	CBYT2637.b1	GE286020
61929976	CBYT2637.g1	GE286021
61929977	CBYT2638.b1	GE286022
61929978	CBYT2638.g1	GE286023
61929979	CBYT2639.b1	GE286024
61929980	CBYT2639.g1	GE286025
61929981	CBYT2640.b1	GE286026
61929982	CBYT2640.g1	GE286027
61929983	CBYT2641.b1	GE286028
61929984	CBYT2642.b1	GE286029
61929985	CBYT2643.b1	GE286030
61929986	CBYT2643.g1	GE286031
61929987	CBYT2645.b1	GE286032
61929988	CBYT2645.g1	GE286033
61929989	CBYT2646.b1	GE286034
61929990	CBYT2648.b1	GE286035
61929991	CBYT2648.g1	GE286036
61929992	CBYT2649.b1	GE286037
61929993	CBYT2649.g1	GE286038
61929994	CBYT2650.b1	GE286039
61929995	CBYT2650.g1	GE286040
61929996	CBYT2651.b1	GE286041
61929997	CBYT2651.g1	GE286042
61929998	CBYT2652.b1	GE286043
61929999	CBYT2652.g1	GE286044
61930000	CBYT2653.b1	GE286045
61930001	CBYT2654.b1	GE286046
61930002	CBYT2654.g1	GE286047
61930003	CBYT2656.b1	GE286048
61930004	CBYT2656.g1	GE286049
61930005	CBYT2657.b1	GE286050
61930006	CBYT2657.g1	GE286051
61930007	CBYT2658.b1	GE286052
61930008	CBYT2658.g1	GE286053
61930009	CBYT2659.b1	GE286054
61930010	CBYT2659.g1	GE286055
61930011	CBYT2660.b1	GE286056

61930012	CBYT2660.g1	GE286057
61930013	CBYT2661.b1	GE286058
61930014	CBYT2661.g1	GE286059
61930015	CBYT2662.b1	GE286060
61930016	CBYT2662.g1	GE286061
61930017	CBYT2663.g1	GE286062
61930018	CBYT2664.b1	GE286063
61930019	CBYT2664.g1	GE286064
61930020	CBYT2665.b1	GE286065
61930021	CBYT2665.g1	GE286066
61930022	CBYT2666.b1	GE286067
61930023	CBYT2666.g1	GE286068
61930024	CBYT2667.b1	GE286069
61930025	CBYT2667.g1	GE286070
61930026	CBYT2668.b1	GE286071
61930027	CBYT2668.g1	GE286072
61930028	CBYT2669.b1	GE286073
61930029	CBYT2670.b1	GE286074
61930030	CBYT2670.g1	GE286075
61930031	CBYT2671.b1	GE286076
61930032	CBYT2671.g1	GE286077
61930033	CBYT2672.b1	GE286078
61930034	CBYT2672.g1	GE286079
61930035	CBYT2673.b1	GE286080
61930036	CBYT2674.b1	GE286081
61930037	CBYT2676.b1	GE286082
61930038	CBYT2676.g1	GE286083
61930039	CBYT2677.b1	GE286084
61930040	CBYT2677.g1	GE286085
61930041	CBYT2678.b1	GE286086
61930042	CBYT2678.g1	GE286087
61930043	CBYT2679.b1	GE286088
61930044	CBYT2679.g1	GE286089
61930045	CBYT2680.b1	GE286090
61930046	CBYT2680.g1	GE286091
61930047	CBYT2681.b1	GE286092
61930048	CBYT2681.g1	GE286093
61930049	CBYT2682.b1	GE286094
61930050	CBYT2682.g1	GE286095
61930051	CBYT2683.b1	GE286096
61930052	CBYT2683.g1	GE286097
61930053	CBYT2684.b1	GE286098
61930054	CBYT2684.g1	GE286099
61930055	CBYT2686.b1	GE286100
61930056	CBYT2686.g1	GE286101
61930057	CBYT2687.g1	GE286102
61930058	CBYT2688.b1	GE286103
61930059	CBYT2688.g1	GE286104
61930060	CBYT2689.b1	GE286105
61930061	CBYT2690.b1	GE286106
61930062	CBYT2690.g1	GE286107
61930063	CBYT2691.b1	GE286108
61930064	CBYT2691.g1	GE286109
61930065	CBYT2692.b1	GE286110
61930066	CBYT2692.g1	GE286111
61930067	CBYT2693.b1	GE286112
61930068	CBYT2693.g1	GE286113
61930069	CBYT2694.b1	GE286114
61930070	CBYT2695.b1	GE286115
61930071	CBYT2695.g1	GE286116
61930072	CBYT2697.b1	GE286117
61930073	CBYT2697.g1	GE286118
61930074	CBYT2698.b1	GE286119

61930075	CBYT2698.g1	GE286120
61930076	CBYT2699.b1	GE286121
61930077	CBYT2699.g1	GE286122
61930078	CBYT2700.b1	GE286123
61930079	CBYT2700.g1	GE286124
61930080	CBYT2701.b1	GE286125
61930081	CBYT2701.g1	GE286126
61930082	CBYT2702.b1	GE286127
61930083	CBYT2702.g1	GE286128
61930084	CBYT2703.b1	GE286129
61930085	CBYT2703.g1	GE286130
61930086	CBYT2704.b1	GE286131
61930087	CBYT2704.g1	GE286132
61930088	CBYT2705.b1	GE286133
61930089	CBYT2705.g1	GE286134
61930090	CBYT2706.b1	GE286135
61930091	CBYT2706.g1	GE286136
61930092	CBYT2707.b1	GE286137
61930093	CBYT2707.g1	GE286138
61930094	CBYT2708.b1	GE286139
61930095	CBYT2708.g1	GE286140
61930096	CBYT2709.b1	GE286141
61930097	CBYT2709.g1	GE286142
61930098	CBYT2710.b1	GE286143
61930099	CBYT2710.g1	GE286144
61930100	CBYT2711.b1	GE286145
61930101	CBYT2715.b1	GE286146
61930102	CBYT2715.g1	GE286147
61930103	CBYT2716.b1	GE286148
61930104	CBYT2716.g1	GE286149
61930105	CBYT2718.b1	GE286150
61930106	CBYT2718.g1	GE286151
61930107	CBYT2719.b1	GE286152
61930108	CBYT2719.g1	GE286153
61930109	CBYT2720.b1	GE286154
61930110	CBYT2721.b1	GE286155
61930111	CBYT2722.b1	GE286156
61930112	CBYT2722.g1	GE286157
61930113	CBYT2723.b1	GE286158
61930114	CBYT2723.g1	GE286159
61930115	CBYT2724.b1	GE286160
61930116	CBYT2724.g1	GE286161
61930117	CBYT2725.b1	GE286162
61930118	CBYT2725.g1	GE286163
61930119	CBYT2726.b1	GE286164
61930120	CBYT2726.g1	GE286165
61930121	CBYT2727.b1	GE286166
61930122	CBYT2727.g1	GE286167
61930123	CBYT2728.b1	GE286168
61930124	CBYT2728.g1	GE286169
61930125	CBYT2729.b1	GE286170
61930126	CBYT2730.b1	GE286171
61930127	CBYT2730.g1	GE286172
61930128	CBYT2731.b1	GE286173
61930129	CBYT2731.g1	GE286174
61930130	CBYT2733.b1	GE286175
61930131	CBYT2733.g1	GE286176
61930132	CBYT2734.b1	GE286177
61930133	CBYT2734.g1	GE286178
61930134	CBYT2735.b1	GE286179
61930135	CBYT2736.b1	GE286180
61930136	CBYT2737.b1	GE286181
61930137	CBYT2737.g1	GE286182

61930138	CBYT2738.b1	GE286183
61930139	CBYT2738.g1	GE286184
61930140	CBYT2739.b1	GE286185
61930141	CBYT2739.g1	GE286186
61930142	CBYT2740.b1	GE286187
61930143	CBYT2740.g1	GE286188
61930144	CBYT2741.b1	GE286189
61930145	CBYT2742.b1	GE286190
61930146	CBYT2742.g1	GE286191
61930147	CBYT2743.b1	GE286192
61930148	CBYT2744.b1	GE286193
61930149	CBYT2745.b1	GE286194
61930150	CBYT2745.g1	GE286195
61930151	CBYT2746.b1	GE286196
61930152	CBYT2746.g1	GE286197
61930153	CBYT2748.b1	GE286198
61930154	CBYT2749.b1	GE286199
61930155	CBYT2749.g1	GE286200
61930156	CBYT2750.b1	GE286201
61930157	CBYT2750.g1	GE286202
61930158	CBYT2751.b1	GE286203
61930159	CBYT2751.g1	GE286204
61930160	CBYT2752.b1	GE286205
61930161	CBYT2752.g1	GE286206
61930162	CBYT2753.b1	GE286207
61930163	CBYT2753.g1	GE286208
61930164	CBYT2754.b1	GE286209
61930165	CBYT2754.g1	GE286210
61930166	CBYT2755.b1	GE286211
61930167	CBYT2755.g1	GE286212
61930168	CBYT2756.b1	GE286213
61930169	CBYT2756.g1	GE286214
61930170	CBYT2757.b1	GE286215
61930171	CBYT2757.g1	GE286216
61930172	CBYT2758.b1	GE286217
61930173	CBYT2758.g1	GE286218
61930174	CBYT2759.b1	GE286219
61930175	CBYT2759.g1	GE286220
61930176	CBYT2760.b1	GE286221
61930177	CBYT2760.g1	GE286222
61930178	CBYT2761.b1	GE286223
61930179	CBYT2761.g1	GE286224
61930180	CBYT2762.b1	GE286225
61930181	CBYT2762.g1	GE286226
61930182	CBYT2764.b1	GE286227
61930183	CBYT2764.g1	GE286228
61930184	CBYT2765.b1	GE286229
61930185	CBYT2765.g1	GE286230
61930186	CBYT2766.b1	GE286231
61930187	CBYT2766.g1	GE286232
61930188	CBYT2768.b1	GE286233
61930189	CBYT2770.b1	GE286234
61930190	CBYT2770.g1	GE286235
61930191	CBYT2771.b1	GE286236
61930192	CBYT2771.g1	GE286237
61930193	CBYT2772.b1	GE286238
61930194	CBYT2772.g1	GE286239
61930195	CBYT2773.b1	GE286240
61930196	CBYT2773.g1	GE286241
61930197	CBYT2774.b1	GE286242
61930198	CBYT2774.g1	GE286243
61930199	CBYT2775.b1	GE286244
61930200	CBYT2775.g1	GE286245

61930201	CBYT2776.b1	GE286246
61930202	CBYT2776.g1	GE286247
61930203	CBYT2777.b1	GE286248
61930204	CBYT2777.g1	GE286249
61930205	CBYT2778.b1	GE286250
61930206	CBYT2779.b1	GE286251
61930207	CBYT2779.g1	GE286252
61930208	CBYT2780.b1	GE286253
61930209	CBYT2783.b1	GE286254
61930210	CBYT2783.g1	GE286255
61930211	CBYT2785.b1	GE286256
61930212	CBYT2785.g1	GE286257
61930213	CBYT2786.b1	GE286258
61930214	CBYT2786.g1	GE286259
61930215	CBYT2787.b1	GE286260
61930216	CBYT2789.b1	GE286261
61930217	CBYT2790.b1	GE286262
61930218	CBYT2790.g1	GE286263
61930219	CBYT2791.b1	GE286264
61930220	CBYT2791.g1	GE286265
61930221	CBYT2794.b1	GE286266
61930222	CBYT2794.g1	GE286267
61930223	CBYT2795.b1	GE286268
61930224	CBYT2795.g1	GE286269
61930225	CBYT2796.b1	GE286270
61930226	CBYT2796.g1	GE286271
61930227	CBYT2797.b1	GE286272
61930228	CBYT2797.g1	GE286273
61930229	CBYT2798.b1	GE286274
61930230	CBYT2801.b1	GE286275
61930231	CBYT2801.g1	GE286276
61930232	CBYT2802.b1	GE286277
61930233	CBYT2802.g1	GE286278
61930234	CBYT2803.b1	GE286279
61930235	CBYT2803.g1	GE286280
61930236	CBYT2804.b1	GE286281
61930237	CBYT2804.g1	GE286282
61930238	CBYT2805.b1	GE286283
61930239	CBYT2805.g1	GE286284
61930240	CBYT2806.b1	GE286285
61930241	CBYT2806.g1	GE286286
61930242	CBYT2807.b1	GE286287
61930243	CBYT2807.g1	GE286288
61930244	CBYT2808.g1	GE286289
61930245	CBYT2809.b1	GE286290
61930246	CBYT2809.g1	GE286291
61930247	CBYT2810.b1	GE286292
61930248	CBYT2810.g1	GE286293
61930249	CBYT2811.b1	GE286294
61930250	CBYT2811.g1	GE286295
61930251	CBYT2812.b1	GE286296
61930252	CBYT2812.g1	GE286297
61930253	CBYT2813.b1	GE286298
61930254	CBYT2813.g1	GE286299
61930255	CBYT2814.b1	GE286300
61930256	CBYT2814.g1	GE286301
61930257	CBYT2815.b1	GE286302
61930258	CBYT2816.b1	GE286303
61930259	CBYT2817.b1	GE286304
61930260	CBYT2817.g1	GE286305
61930261	CBYT2818.b1	GE286306
61930262	CBYT2818.g1	GE286307
61930263	CBYT2819.b1	GE286308

61930264	CBYT2819.g1	GE286309
61930265	CBYT2820.b1	GE286310
61930266	CBYT2820.g1	GE286311
61930267	CBYT2821.b1	GE286312
61930268	CBYT2821.g1	GE286313
61930269	CBYT2822.b1	GE286314
61930270	CBYT2822.g1	GE286315
61930271	CBYT2823.b1	GE286316
61930272	CBYT2823.g1	GE286317
61930273	CBYT2824.b1	GE286318
61930274	CBYT2824.g1	GE286319
61930275	CBYT2825.b1	GE286320
61930276	CBYT2825.g1	GE286321
61930277	CBYT2826.b1	GE286322
61930278	CBYT2826.g1	GE286323
61930279	CBYT2827.b1	GE286324
61930280	CBYT2827.g1	GE286325
61930281	CBYT2828.b1	GE286326
61930282	CBYT2828.g1	GE286327
61930283	CBYT2830.g1	GE286328
61930284	CBYT2831.b1	GE286329
61930285	CBYT2831.g1	GE286330
61930286	CBYT2832.b1	GE286331
61930287	CBYT2832.g1	GE286332
61930288	CBYT2833.b1	GE286333
61930289	CBYT2833.g1	GE286334
61930290	CBYT2834.b1	GE286335
61930291	CBYT2834.g1	GE286336
61930292	CBYT2835.b1	GE286337
61930293	CBYT2835.g1	GE286338
61930294	CBYT2836.b1	GE286339
61930295	CBYT2836.g1	GE286340
61930296	CBYT2837.b1	GE286341
61930297	CBYT2837.g1	GE286342
61930298	CBYT2838.b1	GE286343
61930299	CBYT2842.b1	GE286344
61930300	CBYT2842.g1	GE286345
61930301	CBYT2843.b1	GE286346
61930302	CBYT2843.g1	GE286347
61930303	CBYT2844.b1	GE286348
61930304	CBYT2844.g1	GE286349
61930305	CBYT2845.b1	GE286350
61930306	CBYT2846.b1	GE286351
61930307	CBYT2846.g1	GE286352
61930308	CBYT2847.b1	GE286353
61930309	CBYT2847.g1	GE286354
61930310	CBYT2848.b1	GE286355
61930311	CBYT2848.g1	GE286356
61930312	CBYT2849.b1	GE286357
61930313	CBYT2849.g1	GE286358
61930314	CBYT2852.b1	GE286359
61930315	CBYT2853.b1	GE286360
61930316	CBYT2853.g1	GE286361
61930317	CBYT2854.b1	GE286362
61930318	CBYT2854.g1	GE286363
61930319	CBYT2855.b1	GE286364
61930320	CBYT2855.g1	GE286365
61930321	CBYT2856.b1	GE286366
61930322	CBYT2859.b1	GE286367
61930323	CBYT2860.b1	GE286368
61930324	CBYT2860.g1	GE286369
61930325	CBYT2861.b1	GE286370
61930326	CBYT2861.g1	GE286371

61930327	CBYT2862.b1	GE286372
61930328	CBYT2862.g1	GE286373
61930329	CBYT2864.b1	GE286374
61930330	CBYT2864.g1	GE286375
61930331	CBYT2865.b1	GE286376
61930332	CBYT2865.g1	GE286377
61930333	CBYT2866.b1	GE286378
61930334	CBYT2866.g1	GE286379
61930335	CBYT2867.b1	GE286380
61930336	CBYT2868.b1	GE286381
61930337	CBYT2868.g1	GE286382
61930338	CBYT2869.b1	GE286383
61930339	CBYT2869.g1	GE286384
61930340	CBYT2870.b1	GE286385
61930341	CBYT2870.g1	GE286386
61930342	CBYT2871.b1	GE286387
61930343	CBYT2872.b1	GE286388
61930344	CBYT2872.g1	GE286389
61930345	CBYT2873.b1	GE286390
61930346	CBYT2874.b1	GE286391
61930347	CBYT2874.g1	GE286392
61930348	CBYT2875.b1	GE286393
61930349	CBYT2876.b1	GE286394
61930350	CBYT2876.g1	GE286395
61930351	CBYT2878.b1	GE286396
61930352	CBYT2878.g1	GE286397
61930353	CBYT2879.b1	GE286398
61930354	CBYT2879.g1	GE286399
61930355	CBYT2880.b1	GE286400
61930356	CBYT2880.g1	GE286401
61930357	CBYT2881.b1	GE286402
61930358	CBYT2881.g1	GE286403
61930359	CBYT2882.b1	GE286404
61930360	CBYT2883.b1	GE286405
61930361	CBYT2883.g1	GE286406
61930362	CBYT2884.g1	GE286407
61930363	CBYT2885.b1	GE286408
61930364	CBYT2886.b1	GE286409
61930365	CBYT2886.g1	GE286410
61930366	CBYT2887.b1	GE286411
61930367	CBYT2887.g1	GE286412
61930368	CBYT2890.b1	GE286413
61930369	CBYT2890.g1	GE286414
61930370	CBYT2891.b1	GE286415
61930371	CBYT2891.g1	GE286416
61930372	CBYT2892.b1	GE286417
61930373	CBYT2892.g1	GE286418
61930374	CBYT2893.b1	GE286419
61930375	CBYT2893.g1	GE286420
61930376	CBYT2894.b1	GE286421
61930377	CBYT2894.g1	GE286422
61930378	CBYT2895.b1	GE286423
61930379	CBYT2895.g1	GE286424
61930380	CBYT2896.b1	GE286425
61930381	CBYT2896.g1	GE286426
61930382	CBYT2897.b1	GE286427
61930383	CBYT2897.g1	GE286428
61930384	CBYT2899.b1	GE286429
61930385	CBYT2899.g1	GE286430
61930386	CBYT2900.b1	GE286431
61930387	CBYT2900.g1	GE286432
61930388	CBYT2901.b1	GE286433
61930389	CBYT2901.g1	GE286434

61930390	CBYT2902.g1	GE286435
61930391	CBYT2903.b1	GE286436
61930392	CBYT2903.g1	GE286437
61930393	CBYT2904.b1	GE286438
61930394	CBYT2904.g1	GE286439
61930395	CBYT2905.b1	GE286440
61930396	CBYT2905.g1	GE286441
61930397	CBYT2906.b1	GE286442
61930398	CBYT2907.b1	GE286443
61930399	CBYT2907.g1	GE286444
61930400	CBYT2908.b1	GE286445
61930401	CBYT2908.g1	GE286446
61930402	CBYT2909.b1	GE286447
61930403	CBYT2909.g1	GE286448
61930404	CBYT2910.b1	GE286449
61930405	CBYT2910.g1	GE286450
61930406	CBYT2911.b1	GE286451
61930407	CBYT2911.g1	GE286452
61930408	CBYT2912.b1	GE286453
61930409	CBYT2912.g1	GE286454
61930410	CBYT2914.g1	GE286455
61930411	CBYT2915.b1	GE286456
61930412	CBYT2915.g1	GE286457
61930413	CBYT2917.b1	GE286458
61930414	CBYT2918.b1	GE286459
61930415	CBYT2918.g1	GE286460
61930416	CBYT2919.b1	GE286461
61930417	CBYT2919.g1	GE286462
61930418	CBYT2921.b1	GE286463
61930419	CBYT2921.g1	GE286464
61930420	CBYT2922.b1	GE286465
61930421	CBYT2922.g1	GE286466
61930422	CBYT2923.b1	GE286467
61930423	CBYT2923.g1	GE286468
61930424	CBYT2924.g1	GE286469
61930425	CBYT2925.b1	GE286470
61930426	CBYT2925.g1	GE286471
61930427	CBYT2926.b1	GE286472
61930428	CBYT2926.g1	GE286473
61930429	CBYT2927.b1	GE286474
61930430	CBYT2927.g1	GE286475
61930431	CBYT2928.b1	GE286476
61930432	CBYT2928.g1	GE286477
61930433	CBYT2929.b1	GE286478
61930434	CBYT2930.b1	GE286479
61930435	CBYT2930.g1	GE286480
61930436	CBYT2931.b1	GE286481
61930437	CBYT2931.g1	GE286482
61930438	CBYT2933.b1	GE286483
61930439	CBYT2933.g1	GE286484
61930440	CBYT2934.b1	GE286485
61930441	CBYT2934.g1	GE286486
61930442	CBYT2935.b1	GE286487
61930443	CBYT2935.g1	GE286488
61930444	CBYT2936.b1	GE286489
61930445	CBYT2936.g1	GE286490
61930446	CBYT2937.b1	GE286491
61930447	CBYT2937.g1	GE286492
61930448	CBYT2938.b1	GE286493
61930449	CBYT2938.g1	GE286494
61930450	CBYT2939.b1	GE286495
61930451	CBYT2939.g1	GE286496
61930452	CBYT2940.b1	GE286497

61930453	CBYT2940.g1	GE286498
61930454	CBYT2941.g1	GE286499
61930455	CBYT2942.b1	GE286500
61930456	CBYT2942.g1	GE286501
61930457	CBYT2943.b1	GE286502
61930458	CBYT2943.g1	GE286503
61930459	CBYT2944.b1	GE286504
61930460	CBYT2944.g1	GE286505
61930461	CBYT2945.b1	GE286506
61930462	CBYT2946.b1	GE286507
61930463	CBYT2946.g1	GE286508
61930464	CBYT2947.b1	GE286509
61930465	CBYT2947.g1	GE286510
61930466	CBYT2948.b1	GE286511
61930467	CBYT2948.g1	GE286512
61930468	CBYT2949.b1	GE286513
61930469	CBYT2949.g1	GE286514
61930470	CBYT2950.b1	GE286515
61930471	CBYT2950.g1	GE286516
61930472	CBYT2951.g1	GE286517
61930473	CBYT2952.b1	GE286518
61930474	CBYT2952.g1	GE286519
61930475	CBYT2955.g1	GE286520
61930476	CBYT2956.b1	GE286521
61930477	CBYT2957.b1	GE286522
61930478	CBYT2957.g1	GE286523
61930479	CBYT2958.b1	GE286524
61930480	CBYT2958.g1	GE286525
61930481	CBYT2959.b1	GE286526
61930482	CBYT2959.g1	GE286527
61930483	CBYT2960.b1	GE286528
61930484	CBYT2960.g1	GE286529
61930485	CBYT2962.b1	GE286530
61930486	CBYT2962.g1	GE286531
61930487	CBYT2963.b1	GE286532
61930488	CBYT2963.g1	GE286533
61930489	CBYT2964.b1	GE286534
61930490	CBYT2964.g1	GE286535
61930491	CBYT2965.b1	GE286536
61930492	CBYT2965.g1	GE286537
61930493	CBYT2966.b1	GE286538
61930494	CBYT2967.b1	GE286539
61930495	CBYT2967.g1	GE286540
61930496	CBYT2969.b1	GE286541
61930497	CBYT2969.g1	GE286542
61930498	CBYT2970.b1	GE286543
61930499	CBYT2970.g1	GE286544
61930500	CBYT2971.b1	GE286545
61930501	CBYT2971.g1	GE286546
61930502	CBYT2972.b1	GE286547
61930503	CBYT2972.g1	GE286548
61930504	CBYT2973.b1	GE286549
61930505	CBYT2973.g1	GE286550
61930506	CBYT2975.b1	GE286551
61930507	CBYT2975.g1	GE286552
61930508	CBYT2977.b1	GE286553
61930509	CBYT2977.g1	GE286554
61930510	CBYT2978.b1	GE286555
61930511	CBYT2978.g1	GE286556
61930512	CBYT2979.b1	GE286557
61930513	CBYT2979.g1	GE286558
61930514	CBYT2980.b1	GE286559
61930515	CBYT2980.g1	GE286560

61930516	CBYT2981.b1	GE286561
61930517	CBYT2981.g1	GE286562
61930518	CBYT2982.g1	GE286563
61930519	CBYT2983.b1	GE286564
61930520	CBYT2983.g1	GE286565
61930521	CBYT2985.b1	GE286566
61930522	CBYT2985.g1	GE286567
61930523	CBYT2986.b1	GE286568
61930524	CBYT2986.g1	GE286569
61930525	CBYT2987.b1	GE286570
61930526	CBYT2988.b1	GE286571
61930527	CBYT2988.g1	GE286572
61930528	CBYT2989.b1	GE286573
61930529	CBYT2989.g1	GE286574
61930530	CBYT2990.b1	GE286575
61930531	CBYT2990.g1	GE286576
61930532	CBYT2991.b1	GE286577
61930533	CBYT2991.g1	GE286578
61930534	CBYT2992.b1	GE286579
61930535	CBYT2992.g1	GE286580
61930536	CBYT2993.b1	GE286581
61930537	CBYT2994.b1	GE286582
61930538	CBYT2994.g1	GE286583
61930539	CBYT2995.b1	GE286584
61930540	CBYT2995.g1	GE286585
61930541	CBYT2996.g1	GE286586
61930542	CBYT2997.b1	GE286587
61930543	CBYT2997.g1	GE286588
61930544	CBYT2998.g1	GE286589
61930545	CBYT2999.b1	GE286590
61930546	CBYT2999.g1	GE286591
61930547	CBYT3000.b1	GE286592
61930548	CBYT3001.b1	GE286593
61930549	CBYT3002.b1	GE286594
61930550	CBYT3002.g1	GE286595
61930551	CBYT3003.b1	GE286596
61930552	CBYT3003.g1	GE286597
61930553	CBYT3004.b1	GE286598
61930554	CBYT3004.g1	GE286599
61930555	CBYT3005.b1	GE286600
61930556	CBYT3005.g1	GE286601
61930557	CBYT3006.b1	GE286602
61930558	CBYT3006.g1	GE286603
61930559	CBYT3007.b1	GE286604
61930560	CBYT3007.g1	GE286605
61930561	CBYT3009.b1	GE286606
61930562	CBYT3009.g1	GE286607
61930563	CBYT3010.b1	GE286608
61930564	CBYT3010.g1	GE286609
61930565	CBYT3011.g1	GE286610
61930566	CBYT3012.b1	GE286611
61930567	CBYT3012.g1	GE286612
61930568	CBYT3013.b1	GE286613
61930569	CBYT3013.g1	GE286614
61930570	CBYT3014.b1	GE286615
61930571	CBYT3015.b1	GE286616
61930572	CBYT3015.g1	GE286617
61930573	CBYT3016.b1	GE286618
61930574	CBYT3016.g1	GE286619
61930575	CBYT3017.b1	GE286620
61930576	CBYT3017.g1	GE286621
61930577	CBYT3018.b1	GE286622
61930578	CBYT3018.g1	GE286623

61930579	CBYT3019.b1	GE286624
61930580	CBYT3019.g1	GE286625
61930581	CBYT3020.b1	GE286626
61930582	CBYT3020.g1	GE286627
61930583	CBYT3021.b1	GE286628
61930584	CBYT3021.g1	GE286629
61930585	CBYT3022.b1	GE286630
61930586	CBYT3022.g1	GE286631
61930587	CBYT3023.b1	GE286632
61930588	CBYT3023.g1	GE286633
61930589	CBYT3024.b1	GE286634
61930590	CBYT3024.g1	GE286635
61930591	CBYT3026.b1	GE286636
61930592	CBYT3026.g1	GE286637
61930593	CBYT3027.b1	GE286638
61930594	CBYT3027.g1	GE286639
61930595	CBYT3028.b1	GE286640
61930596	CBYT3029.b1	GE286641
61930597	CBYT3029.g1	GE286642
61930598	CBYT3030.b1	GE286643
61930599	CBYT3030.g1	GE286644
61930600	CBYT3031.b1	GE286645
61930601	CBYT3031.g1	GE286646
61930602	CBYT3032.b1	GE286647
61930603	CBYT3033.b1	GE286648
61930604	CBYT3033.g1	GE286649
61930605	CBYT3034.b1	GE286650
61930606	CBYT3034.g1	GE286651
61930607	CBYT3035.b1	GE286652
61930608	CBYT3035.g1	GE286653
61930609	CBYT3036.b1	GE286654
61930610	CBYT3037.b1	GE286655
61930611	CBYT3037.g1	GE286656
61930612	CBYT3038.b1	GE286657
61930613	CBYT3038.g1	GE286658
61930614	CBYT3039.b1	GE286659
61930615	CBYT3039.g1	GE286660
61930616	CBYT3040.b1	GE286661
61930617	CBYT3040.g1	GE286662
61930618	CBYT3041.b1	GE286663
61930619	CBYT3042.b1	GE286664
61930620	CBYT3042.g1	GE286665
61930621	CBYT3043.b1	GE286666
61930622	CBYT3043.g1	GE286667
61930623	CBYT3044.b1	GE286668
61930624	CBYT3044.g1	GE286669
61930625	CBYT3045.b1	GE286670
61930626	CBYT3045.g1	GE286671
61930627	CBYT3046.b1	GE286672
61930628	CBYT3046.g1	GE286673
61930629	CBYT3047.b1	GE286674
61930630	CBYT3047.g1	GE286675
61930631	CBYT3049.g1	GE286676
61930632	CBYT3051.b1	GE286677
61930633	CBYT3051.g1	GE286678
61930634	CBYT3052.b1	GE286679
61930635	CBYT3052.g1	GE286680
61930636	CBYT3053.b1	GE286681
61930637	CBYT3053.g1	GE286682
61930638	CBYT3054.b1	GE286683
61930639	CBYT3054.g1	GE286684
61930640	CBYT3056.b1	GE286685
61930641	CBYT3056.g1	GE286686

61930642	CBYT3058.b1	GE286687
61930643	CBYT3058.g1	GE286688
61930644	CBYT3059.b1	GE286689
61930645	CBYT3060.b1	GE286690
61930646	CBYT3060.g1	GE286691
61930647	CBYT3062.b1	GE286692
61930648	CBYT3062.g1	GE286693
61930649	CBYT3063.b1	GE286694
61930650	CBYT3063.g1	GE286695
61930651	CBYT3065.b1	GE286696
61930652	CBYT3065.g1	GE286697
61930653	CBYT3066.g1	GE286698
61930654	CBYT3068.b1	GE286699
61930655	CBYT3068.g1	GE286700
61930656	CBYT3069.b1	GE286701
61930657	CBYT3069.g1	GE286702
61930658	CBYT3070.b1	GE286703
61930659	CBYT3070.g1	GE286704
61930660	CBYT3073.b1	GE286705
61930661	CBYT3073.g1	GE286706
61930662	CBYT3074.b1	GE286707
61930663	CBYT3074.g1	GE286708
61930664	CBYT3075.b1	GE286709
61930665	CBYT3075.g1	GE286710
61930666	CBYT3076.b1	GE286711
61930667	CBYT3076.g1	GE286712
61930668	CBYT3077.b1	GE286713
61930669	CBYT3077.g1	GE286714
61930670	CBYT3078.b1	GE286715
61930671	CBYT3078.g1	GE286716
61930672	CBYT3079.b1	GE286717
61930673	CBYT3079.g1	GE286718
61930674	CBYT3080.b1	GE286719
61930675	CBYT3080.g1	GE286720
61930676	CBYT3081.b1	GE286721
61930677	CBYT3082.b1	GE286722
61930678	CBYT3082.g1	GE286723
61930679	CBYT3083.b1	GE286724
61930680	CBYT3083.g1	GE286725
61930681	CBYT3084.b1	GE286726
61930682	CBYT3084.g1	GE286727
61930683	CBYT3085.b1	GE286728
61930684	CBYT3085.g1	GE286729
61930685	CBYT3086.b1	GE286730
61930686	CBYT3086.g1	GE286731
61930687	CBYT3087.b1	GE286732
61930688	CBYT3087.g1	GE286733
61930689	CBYT3088.b1	GE286734
61930690	CBYT3088.g1	GE286735
61930691	CBYT3089.b1	GE286736
61930692	CBYT3089.g1	GE286737
61930693	CBYT3090.b1	GE286738
61930694	CBYT3090.g1	GE286739
61930695	CBYT3091.b1	GE286740
61930696	CBYT3091.g1	GE286741
61930697	CBYT3092.b1	GE286742
61930698	CBYT3092.g1	GE286743
61930699	CBYT3093.b1	GE286744
61930700	CBYT3093.g1	GE286745
61930701	CBYT3094.b1	GE286746
61930702	CBYT3094.g1	GE286747
61930703	CBYT3095.b1	GE286748
61930704	CBYT3095.g1	GE286749

61930705	CBYT3096.b1	GE286750
61930706	CBYT3096.g1	GE286751
61930707	CBYT3097.b1	GE286752
61930708	CBYT3097.g1	GE286753
61930709	CBYT3099.b1	GE286754
61930710	CBYT3099.g1	GE286755
61930711	CBYT3101.g1	GE286756
61930712	CBYT3102.b1	GE286757
61930713	CBYT3102.g1	GE286758
61930714	CBYT3104.b1	GE286759
61930715	CBYT3104.g1	GE286760
61930716	CBYT3105.b1	GE286761
61930717	CBYT3105.g1	GE286762
61930718	CBYT3106.b1	GE286763
61930719	CBYT3106.g1	GE286764
61930720	CBYT3107.b1	GE286765
61930721	CBYT3107.g1	GE286766
61930722	CBYT3108.b1	GE286767
61930723	CBYT3108.g1	GE286768
61930724	CBYT3109.b1	GE286769
61930725	CBYT3109.g1	GE286770
61930726	CBYT3110.b1	GE286771
61930727	CBYT3110.g1	GE286772
61930728	CBYT3111.b1	GE286773
61930729	CBYT3111.g1	GE286774
61930730	CBYT3112.b1	GE286775
61930731	CBYT3112.g1	GE286776
61930732	CBYT3113.b1	GE286777
61930733	CBYT3113.g1	GE286778
61930734	CBYT3115.b1	GE286779
61930735	CBYT3115.g1	GE286780
61930736	CBYT3117.b1	GE286781
61930737	CBYT3117.g1	GE286782
61930738	CBYT3119.b1	GE286783
61930739	CBYT3119.g1	GE286784
61930740	CBYT3121.b1	GE286785
61930741	CBYT3122.g1	GE286786
61930742	CBYT3123.b1	GE286787
61930743	CBYT3124.g1	GE286788
61930744	CBYT3125.b1	GE286789
61930745	CBYT3125.g1	GE286790
61930746	CBYT3126.b1	GE286791
61930747	CBYT3127.b1	GE286792
61930748	CBYT3127.g1	GE286793
61930749	CBYT3128.b1	GE286794
61930750	CBYT3128.g1	GE286795
61930751	CBYT3129.b1	GE286796
61930752	CBYT3129.g1	GE286797
61930753	CBYT3130.b1	GE286798
61930754	CBYT3130.g1	GE286799
61930755	CBYT3131.b1	GE286800
61930756	CBYT3131.g1	GE286801
61930757	CBYT3132.b1	GE286802
61930758	CBYT3132.g1	GE286803
61930759	CBYT3133.b1	GE286804
61930760	CBYT3133.g1	GE286805
61930761	CBYT3134.b1	GE286806
61930762	CBYT3134.g1	GE286807
61930763	CBYT3135.b1	GE286808
61930764	CBYT3135.g1	GE286809
61930765	CBYT3136.b1	GE286810
61930766	CBYT3136.g1	GE286811
61930767	CBYT3137.b1	GE286812

61930768	CBYT3137.g1	GE286813
61930769	CBYT3138.b1	GE286814
61930770	CBYT3138.g1	GE286815
61930771	CBYT3139.b1	GE286816
61930772	CBYT3139.g1	GE286817
61930773	CBYT3140.b1	GE286818
61930774	CBYT3140.g1	GE286819
61930775	CBYT3141.b1	GE286820
61930776	CBYT3141.g1	GE286821
61930777	CBYT3142.b1	GE286822
61930778	CBYT3142.g1	GE286823
61930779	CBYT3143.b1	GE286824
61930780	CBYT3143.g1	GE286825
61930781	CBYT3145.b1	GE286826
61930782	CBYT3145.g1	GE286827
61930783	CBYT3146.b1	GE286828
61930784	CBYT3146.g1	GE286829
61930785	CBYT3147.b1	GE286830
61930786	CBYT3149.b1	GE286831
61930787	CBYT3149.g1	GE286832
61930788	CBYT3150.b1	GE286833
61930789	CBYT3150.g1	GE286834
61930790	CBYT3152.g1	GE286835
61930791	CBYT3153.b1	GE286836
61930792	CBYT3153.g1	GE286837
61930793	CBYT3154.b1	GE286838
61930794	CBYT3154.g1	GE286839
61930795	CBYT3156.b1	GE286840
61930796	CBYT3156.g1	GE286841
61930797	CBYT3157.b1	GE286842
61930798	CBYT3157.g1	GE286843
61930799	CBYT3159.b1	GE286844
61930800	CBYT3159.g1	GE286845
61930801	CBYT3160.b1	GE286846
61930802	CBYT3160.g1	GE286847
61930803	CBYT3161.b1	GE286848
61930804	CBYT3161.g1	GE286849
61930805	CBYT3162.b1	GE286850
61930806	CBYT3162.g1	GE286851
61930807	CBYT3163.b1	GE286852
61930808	CBYT3163.g1	GE286853
61930809	CBYT3165.b1	GE286854
61930810	CBYT3165.g1	GE286855
61930811	CBYT3166.b1	GE286856
61930812	CBYT3166.g1	GE286857
61930813	CBYT3167.b1	GE286858
61930814	CBYT3167.g1	GE286859
61930815	CBYT3168.b1	GE286860
61930816	CBYT3168.g1	GE286861
61930817	CBYT3169.g1	GE286862
61930818	CBYT3171.b1	GE286863
61930819	CBYT3171.g1	GE286864
61930820	CBYT3172.b1	GE286865
61930821	CBYT3172.g1	GE286866
61930822	CBYT3173.b1	GE286867
61930823	CBYT3174.g1	GE286868
61930824	CBYT3175.b1	GE286869
61930825	CBYT3175.g1	GE286870
61930826	CBYT3176.b1	GE286871
61930827	CBYT3176.g1	GE286872
61930828	CBYT3177.b1	GE286873
61930829	CBYT3177.g1	GE286874
61930830	CBYT3178.b1	GE286875

61930831	CBYT3178.g1	GE286876
61930832	CBYT3179.b1	GE286877
61930833	CBYT3179.g1	GE286878
61930834	CBYT3180.b1	GE286879
61930835	CBYT3180.g1	GE286880
61930836	CBYT3181.b1	GE286881
61930837	CBYT3182.b1	GE286882
61930838	CBYT3182.g1	GE286883
61930839	CBYT3183.b1	GE286884
61930840	CBYT3184.b1	GE286885
61930841	CBYT3184.g1	GE286886
61930842	CBYT3185.b1	GE286887
61930843	CBYT3185.g1	GE286888
61930844	CBYT3186.b1	GE286889
61930845	CBYT3186.g1	GE286890
61930846	CBYT3187.b1	GE286891
61930847	CBYT3187.g1	GE286892
61930848	CBYT3188.b1	GE286893
61930849	CBYT3188.g1	GE286894
61930850	CBYT3189.b1	GE286895
61930851	CBYT3190.b1	GE286896
61930852	CBYT3190.g1	GE286897
61930853	CBYT3191.b1	GE286898
61930854	CBYT3191.g1	GE286899
61930855	CBYT3192.b1	GE286900
61930856	CBYT3192.g1	GE286901
61930857	CBYT3193.b1	GE286902
61930858	CBYT3193.g1	GE286903
61930859	CBYT3194.b1	GE286904
61930860	CBYT3194.g1	GE286905
61930861	CBYT3195.b1	GE286906
61930862	CBYT3197.b1	GE286907
61930863	CBYT3198.g1	GE286908
61930864	CBYT3199.b1	GE286909
61930865	CBYT3199.g1	GE286910
61930866	CBYT3200.b1	GE286911
61930867	CBYT3200.g1	GE286912
61930868	CBYT3201.b1	GE286913
61930869	CBYT3201.g1	GE286914
61930870	CBYT3202.b1	GE286915
61930871	CBYT3202.g1	GE286916
61930872	CBYT3203.b1	GE286917
61930873	CBYT3203.g1	GE286918
61930874	CBYT3204.b1	GE286919
61930875	CBYT3204.g1	GE286920
61930876	CBYT3205.b1	GE286921
61930877	CBYT3205.g1	GE286922
61930878	CBYT3206.b1	GE286923
61930879	CBYT3206.g1	GE286924
61930880	CBYT3207.b1	GE286925
61930881	CBYT3207.g1	GE286926
61930882	CBYT3208.b1	GE286927
61930883	CBYT3208.g1	GE286928
61930884	CBYT3209.b1	GE286929
61930885	CBYT3209.g1	GE286930
61930886	CBYT3211.b1	GE286931
61930887	CBYT3211.g1	GE286932
61930888	CBYT3213.b1	GE286933
61930889	CBYT3213.g1	GE286934
61930890	CBYT3214.b1	GE286935
61930891	CBYT3214.g1	GE286936
61930892	CBYT3215.b1	GE286937
61930893	CBYT3216.g1	GE286938

61930894	CBYT3217.b1	GE286939
61930895	CBYT3217.g1	GE286940
61930896	CBYT3218.g1	GE286941
61930897	CBYT3219.b1	GE286942
61930898	CBYT3219.g1	GE286943
61930899	CBYT3220.b1	GE286944
61930900	CBYT3220.g1	GE286945
61930901	CBYT3221.b1	GE286946
61930902	CBYT3221.g1	GE286947
61930903	CBYT3222.b1	GE286948
61930904	CBYT3222.g1	GE286949
61930905	CBYT3223.b1	GE286950
61930906	CBYT3223.g1	GE286951
61930907	CBYT3224.b1	GE286952
61930908	CBYT3226.g1	GE286953
61930909	CBYT3227.b1	GE286954
61930910	CBYT3227.g1	GE286955
61930911	CBYT3228.b1	GE286956
61930912	CBYT3228.g1	GE286957
61930913	CBYT3229.b1	GE286958
61930914	CBYT3229.g1	GE286959
61930915	CBYT3230.b1	GE286960
61930916	CBYT3230.g1	GE286961
61930917	CBYT3231.b1	GE286962
61930918	CBYT3231.g1	GE286963
61930919	CBYT3232.b1	GE286964
61930920	CBYT3233.b1	GE286965
61930921	CBYT3233.g1	GE286966
61930922	CBYT3234.b1	GE286967
61930923	CBYT3234.g1	GE286968
61930924	CBYT3235.b1	GE286969
61930925	CBYT3235.g1	GE286970
61930926	CBYT3236.b1	GE286971
61930927	CBYT3236.g1	GE286972
61930928	CBYT3237.b1	GE286973
61930929	CBYT3237.g1	GE286974
61930930	CBYT3238.b1	GE286975
61930931	CBYT3238.g1	GE286976
61930932	CBYT3239.b1	GE286977
61930933	CBYT3239.g1	GE286978
61930934	CBYT3240.b1	GE286979
61930935	CBYT3240.g1	GE286980
61930936	CBYT3241.b1	GE286981
61930937	CBYT3241.g1	GE286982
61930938	CBYT3242.b1	GE286983
61930939	CBYT3242.g1	GE286984
61930940	CBYT3243.b1	GE286985
61930941	CBYT3243.g1	GE286986
61930942	CBYT3245.b1	GE286987
61930943	CBYT3245.g1	GE286988
61930944	CBYT3246.b1	GE286989
61930945	CBYT3246.g1	GE286990
61930946	CBYT3248.b1	GE286991
61930947	CBYT3248.g1	GE286992
61930948	CBYT3249.b1	GE286993
61930949	CBYT3251.b1	GE286994
61930950	CBYT3251.g1	GE286995
61930951	CBYT3252.b1	GE286996
61930952	CBYT3253.b1	GE286997
61930953	CBYT3253.g1	GE286998
61930954	CBYT3254.b1	GE286999
61930955	CBYT3254.g1	GE287000
61930956	CBYT3255.b1	GE287001

61930957	CBYT3255.g1	GE287002
61930958	CBYT3256.b1	GE287003
61930959	CBYT3256.g1	GE287004
61930960	CBYT3257.b1	GE287005
61930961	CBYT3257.g1	GE287006
61930962	CBYT3258.b1	GE287007
61930963	CBYT3258.g1	GE287008
61930964	CBYT3259.b1	GE287009
61930965	CBYT3259.g1	GE287010
61930966	CBYT3260.g1	GE287011
61930967	CBYT3261.b1	GE287012
61930968	CBYT3261.g1	GE287013
61930969	CBYT3262.b1	GE287014
61930970	CBYT3262.g1	GE287015
61930971	CBYT3263.b1	GE287016
61930972	CBYT3263.g1	GE287017
61930973	CBYT3266.b1	GE287018
61930974	CBYT3266.g1	GE287019
61930975	CBYT3267.b1	GE287020
61930976	CBYT3268.b1	GE287021
61930977	CBYT3268.g1	GE287022
61930978	CBYT3269.b1	GE287023
61930979	CBYT3269.g1	GE287024
61930980	CBYT3270.b1	GE287025
61930981	CBYT3270.g1	GE287026
61930982	CBYT3271.b1	GE287027
61930983	CBYT3271.g1	GE287028
61930984	CBYT3272.b1	GE287029
61930985	CBYT3272.g1	GE287030
61930986	CBYT3273.b1	GE287031
61930987	CBYT3273.g1	GE287032
61930988	CBYT3274.b1	GE287033
61930989	CBYT3274.g1	GE287034
61930990	CBYT3275.b1	GE287035
61930991	CBYT3275.g1	GE287036
61930992	CBYT3276.b1	GE287037
61930993	CBYT3276.g1	GE287038
61930994	CBYT3277.b1	GE287039
61930995	CBYT3277.g1	GE287040
61930996	CBYT3278.b1	GE287041
61930997	CBYT3278.g1	GE287042
61930998	CBYT3279.b1	GE287043
61930999	CBYT3279.g1	GE287044
61931000	CBYT3280.b1	GE287045
61931001	CBYT3280.g1	GE287046
61931002	CBYT3281.b1	GE287047
61931003	CBYT3281.g1	GE287048
61931004	CBYT3282.b1	GE287049
61931005	CBYT3282.g1	GE287050
61931006	CBYT3283.b1	GE287051
61931007	CBYT3283.g1	GE287052
61931008	CBYT3284.b1	GE287053
61931009	CBYT3284.g1	GE287054
61931010	CBYT3285.b1	GE287055
61931011	CBYT3285.g1	GE287056
61931012	CBYT3286.b1	GE287057
61931013	CBYT3286.g1	GE287058
61931014	CBYT3287.b1	GE287059
61931015	CBYT3287.g1	GE287060
61931016	CBYT3288.b1	GE287061
61931017	CBYT3288.g1	GE287062
61931018	CBYT3289.b1	GE287063
61931019	CBYT3289.g1	GE287064

61931020	CBYT3291.b1	GE287065
61931021	CBYT3291.g1	GE287066
61931022	CBYT3294.b1	GE287067
61931023	CBYT3294.g1	GE287068
61931024	CBYT3295.b1	GE287069
61931025	CBYT3295.g1	GE287070
61931026	CBYT3296.b1	GE287071
61931027	CBYT3297.b1	GE287072
61931028	CBYT3297.g1	GE287073
61931029	CBYT3298.b1	GE287074
61931030	CBYT3298.g1	GE287075
61931031	CBYT3300.b1	GE287076
61931032	CBYT3300.g1	GE287077
61931033	CBYT3301.b1	GE287078
61931034	CBYT3301.g1	GE287079
61931035	CBYT3302.b1	GE287080
61931036	CBYT3302.g1	GE287081
61931037	CBYT3303.b1	GE287082
61931038	CBYT3303.g1	GE287083
61931039	CBYT3304.b1	GE287084
61931040	CBYT3304.g1	GE287085
61931041	CBYT3305.b1	GE287086
61931042	CBYT3305.g1	GE287087
61931043	CBYT3306.g1	GE287088
61931044	CBYT3307.b1	GE287089
61931045	CBYT3307.g1	GE287090
61931046	CBYT3308.b1	GE287091
61931047	CBYT3309.b1	GE287092
61931048	CBYT3310.b1	GE287093
61931049	CBYT3310.g1	GE287094
61931050	CBYT3311.b1	GE287095
61931051	CBYT3311.g1	GE287096
61931052	CBYT3312.b1	GE287097
61931053	CBYT3312.g1	GE287098
61931054	CBYT3315.b1	GE287099
61931055	CBYT3315.g1	GE287100
61931056	CBYT3316.b1	GE287101
61931057	CBYT3317.b1	GE287102
61931058	CBYT3317.g1	GE287103
61931059	CBYT3318.b1	GE287104
61931060	CBYT3318.g1	GE287105
61931061	CBYT3319.b1	GE287106
61931062	CBYT3319.g1	GE287107
61931063	CBYT3321.b1	GE287108
61931064	CBYT3321.g1	GE287109
61931065	CBYT3322.b1	GE287110
61931066	CBYT3322.g1	GE287111
61931067	CBYT3323.b1	GE287112
61931068	CBYT3323.g1	GE287113
61931069	CBYT3324.b1	GE287114
61931070	CBYT3324.g1	GE287115
61931071	CBYT3325.b1	GE287116
61931072	CBYT3325.g1	GE287117
61931073	CBYT3326.b1	GE287118
61931074	CBYT3327.b1	GE287119
61931075	CBYT3327.g1	GE287120
61931076	CBYT3329.b1	GE287121
61931077	CBYT3329.g1	GE287122
61931078	CBYT3330.b1	GE287123
61931079	CBYT3330.g1	GE287124
61931080	CBYT3331.b1	GE287125
61931081	CBYT3331.g1	GE287126
61931082	CBYT3332.b1	GE287127

61931083	CBYT3332.g1	GE287128
61931084	CBYT3333.b1	GE287129
61931085	CBYT3333.g1	GE287130
61931086	CBYT3334.b1	GE287131
61931087	CBYT3335.b1	GE287132
61931088	CBYT3335.g1	GE287133
61931089	CBYT3336.b1	GE287134
61931090	CBYT3336.g1	GE287135
61931091	CBYT3337.b1	GE287136
61931092	CBYT3337.g1	GE287137
61931093	CBYT3338.b1	GE287138
61931094	CBYT3338.g1	GE287139
61931095	CBYT3339.b1	GE287140
61931096	CBYT3339.g1	GE287141
61931097	CBYT3341.b1	GE287142
61931098	CBYT3341.g1	GE287143
61931099	CBYT3342.b1	GE287144
61931100	CBYT3342.g1	GE287145
61931101	CBYT3344.b1	GE287146
61931102	CBYT3344.g1	GE287147
61931103	CBYT3345.b1	GE287148
61931104	CBYT3345.g1	GE287149
61931105	CBYT3346.b1	GE287150
61931106	CBYT3346.g1	GE287151
61931107	CBYT3347.b1	GE287152
61931108	CBYT3347.g1	GE287153
61931109	CBYT3348.b1	GE287154
61931110	CBYT3348.g1	GE287155
61931111	CBYT3349.b1	GE287156
61931112	CBYT3350.b1	GE287157
61931113	CBYT3350.g1	GE287158
61931114	CBYT3351.b1	GE287159
61931115	CBYT3351.g1	GE287160
61931116	CBYT3352.b1	GE287161
61931117	CBYT3352.g1	GE287162
61931118	CBYT3353.b1	GE287163
61931119	CBYT3353.g1	GE287164
61931120	CBYT3354.b1	GE287165
61931121	CBYT3354.g1	GE287166
61931122	CBYT3355.b1	GE287167
61931123	CBYT3355.g1	GE287168
61931124	CBYT3356.b1	GE287169
61931125	CBYT3357.b1	GE287170
61931126	CBYT3357.g1	GE287171
61931127	CBYT3358.b1	GE287172
61931128	CBYT3358.g1	GE287173
61931129	CBYT3359.b1	GE287174
61931130	CBYT3359.g1	GE287175
61931131	CBYT3361.b1	GE287176
61931132	CBYT3361.g1	GE287177
61931133	CBYT3363.b1	GE287178
61931134	CBYT3363.g1	GE287179
61931135	CBYT3364.b1	GE287180
61931136	CBYT3364.g1	GE287181
61931137	CBYT3365.b1	GE287182
61931138	CBYT3365.g1	GE287183
61931139	CBYT3366.b1	GE287184
61931140	CBYT3366.g1	GE287185
61931141	CBYT3367.b1	GE287186
61931142	CBYT3367.g1	GE287187
61931143	CBYT3368.b1	GE287188
61931144	CBYT3369.b1	GE287189
61931145	CBYT3369.g1	GE287190

61931146	CBYT3370.b1	GE287191
61931147	CBYT3370.g1	GE287192
61931148	CBYT3372.b1	GE287193
61931149	CBYT3372.g1	GE287194
61931150	CBYT3373.b1	GE287195
61931151	CBYT3373.g1	GE287196
61931152	CBYT3374.b1	GE287197
61931153	CBYT3375.b1	GE287198
61931154	CBYT3375.g1	GE287199
61931155	CBYT3376.b1	GE287200
61931156	CBYT3376.g1	GE287201
61931157	CBYT3378.b1	GE287202
61931158	CBYT3378.g1	GE287203
61931159	CBYT3379.b1	GE287204
61931160	CBYT3379.g1	GE287205
61931161	CBYT3380.b1	GE287206
61931162	CBYT3380.g1	GE287207
61931163	CBYT3381.g1	GE287208
61931164	CBYT3382.b1	GE287209
61931165	CBYT3382.g1	GE287210
61931166	CBYT3383.b1	GE287211
61931167	CBYT3383.g1	GE287212
61931168	CBYT3384.b1	GE287213
61931169	CBYT3384.g1	GE287214
61931170	CBYT3385.b1	GE287215
61931171	CBYT3385.g1	GE287216
61931172	CBYT3386.b1	GE287217
61931173	CBYT3386.g1	GE287218
61931174	CBYT3387.b1	GE287219
61931175	CBYT3387.g1	GE287220
61931176	CBYT3390.b1	GE287221
61931177	CBYT3390.g1	GE287222
61931178	CBYT3391.b1	GE287223
61931179	CBYT3391.g1	GE287224
61931180	CBYT3392.b1	GE287225
61931181	CBYT3392.g1	GE287226
61931182	CBYT3393.b1	GE287227
61931183	CBYT3393.g1	GE287228
61931184	CBYT3394.b1	GE287229
61931185	CBYT3395.b1	GE287230
61931186	CBYT3395.g1	GE287231
61931187	CBYT3396.b1	GE287232
61931188	CBYT3396.g1	GE287233
61931189	CBYT3397.b1	GE287234
61931190	CBYT3397.g1	GE287235
61931191	CBYT3398.b1	GE287236
61931192	CBYT3398.g1	GE287237
61931193	CBYT3400.b1	GE287238
61931194	CBYT3400.g1	GE287239
61931195	CBYT3401.g1	GE287240
61931196	CBYT3402.b1	GE287241
61931197	CBYT3403.b1	GE287242
61931198	CBYT3403.g1	GE287243
61931199	CBYT3404.g1	GE287244
61931200	CBYT3405.b1	GE287245
61931201	CBYT3406.b1	GE287246
61931202	CBYT3406.g1	GE287247
61931203	CBYT3407.b1	GE287248
61931204	CBYT3407.g1	GE287249
61931205	CBYT3409.b1	GE287250
61931206	CBYT3409.g1	GE287251
61931207	CBYT3411.b1	GE287252
61931208	CBYT3411.g1	GE287253

61931209	CBYT3412.b1	GE287254
61931210	CBYT3412.g1	GE287255
61931211	CBYT3413.b1	GE287256
61931212	CBYT3413.g1	GE287257
61931213	CBYT3414.b1	GE287258
61931214	CBYT3415.b1	GE287259
61931215	CBYT3415.g1	GE287260
61931216	CBYT3416.b1	GE287261
61931217	CBYT3416.g1	GE287262
61931218	CBYT3418.b1	GE287263
61931219	CBYT3418.g1	GE287264
61931220	CBYT3419.b1	GE287265
61931221	CBYT3419.g1	GE287266
61931222	CBYT3420.b1	GE287267
61931223	CBYT3420.g1	GE287268
61931224	CBYT3421.b1	GE287269
61931225	CBYT3421.g1	GE287270
61931226	CBYT3422.b1	GE287271
61931227	CBYT3422.g1	GE287272
61931228	CBYT3423.b1	GE287273
61931229	CBYT3423.g1	GE287274
61931230	CBYT3424.b1	GE287275
61931231	CBYT3424.g1	GE287276
61931232	CBYT3425.g1	GE287277
61931233	CBYT3426.b1	GE287278
61931234	CBYT3426.g1	GE287279
61931235	CBYT3427.b1	GE287280
61931236	CBYT3427.g1	GE287281
61931237	CBYT3428.b1	GE287282
61931238	CBYT3428.g1	GE287283
61931239	CBYT3429.b1	GE287284
61931240	CBYT3429.g1	GE287285
61931241	CBYT3430.b1	GE287286
61931242	CBYT3430.g1	GE287287
61931243	CBYT3431.b1	GE287288
61931244	CBYT3431.g1	GE287289
61931245	CBYT3432.b1	GE287290
61931246	CBYT3432.g1	GE287291
61931247	CBYT3433.b1	GE287292
61931248	CBYT3433.g1	GE287293
61931249	CBYT3434.b1	GE287294
61931250	CBYT3434.g1	GE287295
61931251	CBYT3435.b1	GE287296
61931252	CBYT3435.g1	GE287297
61931253	CBYT3437.b1	GE287298
61931254	CBYT3437.g1	GE287299
61931255	CBYT3438.b1	GE287300
61931256	CBYT3438.g1	GE287301
61931257	CBYT3440.b1	GE287302
61931258	CBYT3440.g1	GE287303
61931259	CBYT3442.b1	GE287304
61931260	CBYT3442.g1	GE287305
61931261	CBYT3443.b1	GE287306
61931262	CBYT3443.g1	GE287307
61931263	CBYT3444.g1	GE287308
61931264	CBYT3445.b1	GE287309
61931265	CBYT3445.g1	GE287310
61931266	CBYT3446.b1	GE287311
61931267	CBYT3446.g1	GE287312
61931268	CBYT3447.b1	GE287313
61931269	CBYT3447.g1	GE287314
61931270	CBYT3448.b1	GE287315
61931271	CBYT3448.g1	GE287316

61931272	CBYT3449.b1	GE287317
61931273	CBYT3449.g1	GE287318
61931274	CBYT3450.b1	GE287319
61931275	CBYT3450.g1	GE287320
61931276	CBYT3451.b1	GE287321
61931277	CBYT3452.b1	GE287322
61931278	CBYT3452.g1	GE287323
61931279	CBYT3453.b1	GE287324
61931280	CBYT3453.g1	GE287325
61931281	CBYT3454.b1	GE287326
61931282	CBYT3455.b1	GE287327
61931283	CBYT3455.g1	GE287328
61931284	CBYT3456.b1	GE287329
61931285	CBYT3456.g1	GE287330
61931286	CBYT3457.b1	GE287331
61931287	CBYT3457.g1	GE287332
61931288	CBYT3458.b1	GE287333
61931289	CBYT3458.g1	GE287334
61931290	CBYT3459.b1	GE287335
61931291	CBYT3459.g1	GE287336
61931292	CBYT3460.b1	GE287337
61931293	CBYT3460.g1	GE287338
61931294	CBYT3461.g1	GE287339
61931295	CBYT3462.b1	GE287340
61931296	CBYT3462.g1	GE287341
61931297	CBYT3463.b1	GE287342
61931298	CBYT3463.g1	GE287343
61931299	CBYT3464.b1	GE287344
61931300	CBYT3464.g1	GE287345
61931301	CBYT3465.b1	GE287346
61931302	CBYT3465.g1	GE287347
61931303	CBYT3466.b1	GE287348
61931304	CBYT3466.g1	GE287349
61931305	CBYT3467.b1	GE287350
61931306	CBYT3467.g1	GE287351
61931307	CBYT3468.b1	GE287352
61931308	CBYT3468.g1	GE287353
61931309	CBYT3469.b1	GE287354
61931310	CBYT3469.g1	GE287355
61931311	CBYT3470.b1	GE287356
61931312	CBYT3470.g1	GE287357
61931313	CBYT3471.b1	GE287358
61931314	CBYT3471.g1	GE287359
61931315	CBYT3472.b1	GE287360
61931316	CBYT3472.g1	GE287361
61931317	CBYT3473.b1	GE287362
61931318	CBYT3473.g1	GE287363
61931319	CBYT3474.b1	GE287364
61931320	CBYT3474.g1	GE287365
61931321	CBYT3475.b1	GE287366
61931322	CBYT3475.g1	GE287367
61931323	CBYT3478.b1	GE287368
61931324	CBYT3478.g1	GE287369
61931325	CBYT3479.b1	GE287370
61931326	CBYT3479.g1	GE287371
61931327	CBYT3480.b1	GE287372
61931328	CBYT3480.g1	GE287373
61931329	CBYT3481.b1	GE287374
61931330	CBYT3481.g1	GE287375
61931331	CBYT3482.b1	GE287376
61931332	CBYT3483.b1	GE287377
61931333	CBYT3483.g1	GE287378
61931334	CBYT3484.g1	GE287379

61931335	CBYT3485.b1	GE287380
61931336	CBYT3485.g1	GE287381
61931337	CBYT3486.b1	GE287382
61931338	CBYT3486.g1	GE287383
61931339	CBYT3487.b1	GE287384
61931340	CBYT3487.g1	GE287385
61931341	CBYT3488.b1	GE287386
61931342	CBYT3488.g1	GE287387
61931343	CBYT3489.b1	GE287388
61931344	CBYT3489.g1	GE287389
61931345	CBYT3490.b1	GE287390
61931346	CBYT3491.b1	GE287391
61931347	CBYT3491.g1	GE287392
61931348	CBYT3492.b1	GE287393
61931349	CBYT3492.g1	GE287394
61931350	CBYT3493.b1	GE287395
61931351	CBYT3493.g1	GE287396
61931352	CBYT3494.b1	GE287397
61931353	CBYT3494.g1	GE287398
61931354	CBYT3495.b1	GE287399
61931355	CBYT3495.g1	GE287400
61931356	CBYT3496.g1	GE287401
61931357	CBYT3497.b1	GE287402
61931358	CBYT3497.g1	GE287403
61931359	CBYT3498.b1	GE287404
61931360	CBYT3498.g1	GE287405
61931361	CBYT3499.b1	GE287406
61931362	CBYT3499.g1	GE287407
61931363	CBYT3500.b1	GE287408
61931364	CBYT3500.g1	GE287409
61931365	CBYT3501.b1	GE287410
61931366	CBYT3501.g1	GE287411
61931367	CBYT3502.b1	GE287412
61931368	CBYT3502.g1	GE287413
61931369	CBYT3503.b1	GE287414
61931370	CBYT3503.g1	GE287415
61931371	CBYT3504.b1	GE287416
61931372	CBYT3504.g1	GE287417
61931373	CBYT3505.b1	GE287418
61931374	CBYT3505.g1	GE287419
61931375	CBYT3507.b1	GE287420
61931376	CBYT3507.g1	GE287421
61931377	CBYT3508.b1	GE287422
61931378	CBYT3508.g1	GE287423
61931379	CBYT3509.b1	GE287424
61931380	CBYT3509.g1	GE287425
61931381	CBYT3510.g1	GE287426
61931382	CBYT3511.b1	GE287427
61931383	CBYT3511.g1	GE287428
61931384	CBYT3512.b1	GE287429
61931385	CBYT3512.g1	GE287430
61931386	CBYT3513.b1	GE287431
61931387	CBYT3513.g1	GE287432
61931388	CBYT3514.b1	GE287433
61931389	CBYT3515.b1	GE287434
61931390	CBYT3517.b1	GE287435
61931391	CBYT3517.g1	GE287436
61931392	CBYT3519.b1	GE287437
61931393	CBYT3519.g1	GE287438
61931394	CBYT3521.b1	GE287439
61931395	CBYT3521.g1	GE287440
61931396	CBYT3522.b1	GE287441
61931397	CBYT3522.g1	GE287442

61931398	CBYT3523.b1	GE287443
61931399	CBYT3523.g1	GE287444
61931400	CBYT3524.b1	GE287445
61931401	CBYT3524.g1	GE287446
61931402	CBYT3525.b1	GE287447
61931403	CBYT3526.b1	GE287448
61931404	CBYT3526.g1	GE287449
61931405	CBYT3527.b1	GE287450
61931406	CBYT3527.g1	GE287451
61931407	CBYT3528.g1	GE287452
61931408	CBYT3529.b1	GE287453
61931409	CBYT3529.g1	GE287454
61931410	CBYT3530.g1	GE287455
61931411	CBYT3531.b1	GE287456
61931412	CBYT3531.g1	GE287457
61931413	CBYT3532.b1	GE287458
61931414	CBYT3532.g1	GE287459
61931415	CBYT3533.b1	GE287460
61931416	CBYT3533.g1	GE287461
61931417	CBYT3534.b1	GE287462
61931418	CBYT3534.g1	GE287463
61931419	CBYT3535.b1	GE287464
61931420	CBYT3535.g1	GE287465
61931421	CBYT3537.g1	GE287466
61931422	CBYT3538.b1	GE287467
61931423	CBYT3538.g1	GE287468
61931424	CBYT3539.b1	GE287469
61931425	CBYT3539.g1	GE287470
61931426	CBYT3540.b1	GE287471
61931427	CBYT3540.g1	GE287472
61931428	CBYT3541.b1	GE287473
61931429	CBYT3541.g1	GE287474
61931430	CBYT3542.b1	GE287475
61931431	CBYT3542.g1	GE287476
61931432	CBYT3543.b1	GE287477
61931433	CBYT3546.b1	GE287478
61931434	CBYT3546.g1	GE287479
61931435	CBYT3547.b1	GE287480
61931436	CBYT3548.b1	GE287481
61931437	CBYT3548.g1	GE287482
61931438	CBYT3549.b1	GE287483
61931439	CBYT3549.g1	GE287484
61931440	CBYT3550.g1	GE287485
61931441	CBYT3553.b1	GE287486
61931442	CBYT3553.g1	GE287487
61931443	CBYT3554.b1	GE287488
61931444	CBYT3554.g1	GE287489
61931445	CBYT3555.b1	GE287490
61931446	CBYT3556.b1	GE287491
61931447	CBYT3556.g1	GE287492
61931448	CBYT3557.b1	GE287493
61931449	CBYT3558.b1	GE287494
61931450	CBYT3558.g1	GE287495
61931451	CBYT3559.b1	GE287496
61931452	CBYT3559.g1	GE287497
61931453	CBYT3560.b1	GE287498
61931454	CBYT3560.g1	GE287499
61931455	CBYT3561.b1	GE287500
61931456	CBYT3561.g1	GE287501
61931457	CBYT3562.b1	GE287502
61931458	CBYT3562.g1	GE287503
61931459	CBYT3563.b1	GE287504
61931460	CBYT3563.g1	GE287505

61931461	CBYT3564.b1	GE287506
61931462	CBYT3564.g1	GE287507
61931463	CBYT3565.b1	GE287508
61931464	CBYT3565.g1	GE287509
61931465	CBYT3567.b1	GE287510
61931466	CBYT3567.g1	GE287511
61931467	CBYT3568.b1	GE287512
61931468	CBYT3568.g1	GE287513
61931469	CBYT3569.b1	GE287514
61931470	CBYT3569.g1	GE287515
61931471	CBYT3570.b1	GE287516
61931472	CBYT3570.g1	GE287517
61931473	CBYT3571.b1	GE287518
61931474	CBYT3572.b1	GE287519
61931475	CBYT3572.g1	GE287520
61931476	CBYT3573.g1	GE287521
61931477	CBYT3574.b1	GE287522
61931478	CBYT3574.g1	GE287523
61931479	CBYT3575.b1	GE287524
61931480	CBYT3575.g1	GE287525
61931481	CBYT3576.b1	GE287526
61931482	CBYT3576.g1	GE287527
61931483	CBYT3577.b1	GE287528
61931484	CBYT3578.b1	GE287529
61931485	CBYT3578.g1	GE287530
61931486	CBYT3579.b1	GE287531
61931487	CBYT3579.g1	GE287532
61931488	CBYT3580.b1	GE287533
61931489	CBYT3580.g1	GE287534
61931490	CBYT3581.b1	GE287535
61931491	CBYT3581.g1	GE287536
61931492	CBYT3582.b1	GE287537
61931493	CBYT3582.g1	GE287538
61931494	CBYT3583.b1	GE287539
61931495	CBYT3583.g1	GE287540
61931496	CBYT3584.b1	GE287541
61931497	CBYT3585.b1	GE287542
61931498	CBYT3585.g1	GE287543
61931499	CBYT3586.b1	GE287544
61931500	CBYT3586.g1	GE287545
61931501	CBYT3587.b1	GE287546
61931502	CBYT3587.g1	GE287547
61931503	CBYT3588.b1	GE287548
61931504	CBYT3588.g1	GE287549
61931505	CBYT3589.b1	GE287550
61931506	CBYT3589.g1	GE287551
61931507	CBYT3590.b1	GE287552
61931508	CBYT3590.g1	GE287553
61931509	CBYT3591.b1	GE287554
61931510	CBYT3591.g1	GE287555
61931511	CBYT3592.b1	GE287556
61931512	CBYT3592.g1	GE287557
61931513	CBYT3593.b1	GE287558
61931514	CBYT3594.b1	GE287559
61931515	CBYT3595.b1	GE287560
61931516	CBYT3595.g1	GE287561
61931517	CBYT3596.b1	GE287562
61931518	CBYT3596.g1	GE287563
61931519	CBYT3597.b1	GE287564
61931520	CBYT3597.g1	GE287565
61931521	CBYT3598.b1	GE287566
61931522	CBYT3598.g1	GE287567
61931523	CBYT3599.b1	GE287568

61931524	CBYT3599.g1	GE287569
61931525	CBYT3600.b1	GE287570
61931526	CBYT3600.g1	GE287571
61931527	CBYT3601.b1	GE287572
61931528	CBYT3601.g1	GE287573
61931529	CBYT3602.g1	GE287574
61931530	CBYT3603.b1	GE287575
61931531	CBYT3603.g1	GE287576
61931532	CBYT3604.b1	GE287577
61931533	CBYT3604.g1	GE287578
61931534	CBYT3605.b1	GE287579
61931535	CBYT3605.g1	GE287580
61931536	CBYT3606.g1	GE287581
61931537	CBYT3607.b1	GE287582
61931538	CBYT3608.b1	GE287583
61931539	CBYT3608.g1	GE287584
61931540	CBYT3609.b1	GE287585
61931541	CBYT3609.g1	GE287586
61931542	CBYT3610.b1	GE287587
61931543	CBYT3611.b1	GE287588
61931544	CBYT3611.g1	GE287589
61931545	CBYT3612.b1	GE287590
61931546	CBYT3612.g1	GE287591
61931547	CBYT3613.b1	GE287592
61931548	CBYT3613.g1	GE287593
61931549	CBYT3614.b1	GE287594
61931550	CBYT3614.g1	GE287595
61931551	CBYT3615.b1	GE287596
61931552	CBYT3615.g1	GE287597
61931553	CBYT3616.b1	GE287598
61931554	CBYT3616.g1	GE287599
61931555	CBYT3617.g1	GE287600
61931556	CBYT3618.b1	GE287601
61931557	CBYT3619.b1	GE287602
61931558	CBYT3619.g1	GE287603
61931559	CBYT3621.b1	GE287604
61931560	CBYT3621.g1	GE287605
61931561	CBYT3622.g1	GE287606
61931562	CBYT3623.b1	GE287607
61931563	CBYT3623.g1	GE287608
61931564	CBYT3624.g1	GE287609
61931565	CBYT3625.b1	GE287610
61931566	CBYT3625.g1	GE287611
61931567	CBYT3626.g1	GE287612
61931568	CBYT3627.b1	GE287613
61931569	CBYT3627.g1	GE287614
61931570	CBYT3628.b1	GE287615
61931571	CBYT3628.g1	GE287616
61931572	CBYT3629.b1	GE287617
61931573	CBYT3629.g1	GE287618
61931574	CBYT3630.b1	GE287619
61931575	CBYT3630.g1	GE287620
61931576	CBYT3631.b1	GE287621
61931577	CBYT3631.g1	GE287622
61931578	CBYT3632.b1	GE287623
61931579	CBYT3632.g1	GE287624
61931580	CBYT3633.b1	GE287625
61931581	CBYT3633.g1	GE287626
61931582	CBYT3634.b1	GE287627
61931583	CBYT3634.g1	GE287628
61931584	CBYT3636.b1	GE287629
61931585	CBYT3636.g1	GE287630
61931586	CBYT3637.b1	GE287631

61931587	CBYT3637.g1	GE287632
61931588	CBYT3638.g1	GE287633
61931589	CBYT3639.b1	GE287634
61931590	CBYT3639.g1	GE287635
61931591	CBYT3640.g1	GE287636
61931592	CBYT3641.g1	GE287637
61931593	CBYT3642.b1	GE287638
61931594	CBYT3642.g1	GE287639
61931595	CBYT3643.b1	GE287640
61931596	CBYT3643.g1	GE287641
61931597	CBYT3644.b1	GE287642
61931598	CBYT3644.g1	GE287643
61931599	CBYT3645.b1	GE287644
61931600	CBYT3645.g1	GE287645
61931601	CBYT3646.b1	GE287646
61931602	CBYT3646.g1	GE287647
61931603	CBYT3647.b1	GE287648
61931604	CBYT3647.g1	GE287649
61931605	CBYT3648.b1	GE287650
61931606	CBYT3648.g1	GE287651
61931607	CBYT3649.b1	GE287652
61931608	CBYT3649.g1	GE287653
61931609	CBYT3650.b1	GE287654
61931610	CBYT3650.g1	GE287655
61931611	CBYT3651.b1	GE287656
61931612	CBYT3651.g1	GE287657
61931613	CBYT3652.b1	GE287658
61931614	CBYT3652.g1	GE287659
61931615	CBYT3653.b1	GE287660
61931616	CBYT3653.g1	GE287661
61931617	CBYT3654.g1	GE287662
61931618	CBYT3655.b1	GE287663
61931619	CBYT3655.g1	GE287664
61931620	CBYT3656.b1	GE287665
61931621	CBYT3656.g1	GE287666
61931622	CBYT3657.b1	GE287667
61931623	CBYT3657.g1	GE287668
61931624	CBYT3659.b1	GE287669
61931625	CBYT3661.b1	GE287670
61931626	CBYT3661.g1	GE287671
61931627	CBYT3662.b1	GE287672
61931628	CBYT3662.g1	GE287673
61931629	CBYT3663.b1	GE287674
61931630	CBYT3663.g1	GE287675
61931631	CBYT3664.b1	GE287676
61931632	CBYT3664.g1	GE287677
61931633	CBYT3665.b1	GE287678
61931634	CBYT3665.g1	GE287679
61931635	CBYT3666.b1	GE287680
61931636	CBYT3666.g1	GE287681
61931637	CBYT3667.b1	GE287682
61931638	CBYT3667.g1	GE287683
61931639	CBYT3668.b1	GE287684
61931640	CBYT3668.g1	GE287685
61931641	CBYT3669.g1	GE287686
61931642	CBYT3670.b1	GE287687
61931643	CBYT3670.g1	GE287688
61931644	CBYT3671.b1	GE287689
61931645	CBYT3671.g1	GE287690
61931646	CBYT3672.b1	GE287691
61931647	CBYT3672.g1	GE287692
61931648	CBYT3673.b1	GE287693
61931649	CBYT3673.g1	GE287694

61931650	CBYT3674.b1	GE287695
61931651	CBYT3674.g1	GE287696
61931652	CBYT3675.b1	GE287697
61931653	CBYT3676.b1	GE287698
61931654	CBYT3676.g1	GE287699
61931655	CBYT3677.b1	GE287700
61931656	CBYT3677.g1	GE287701
61931657	CBYT3678.g1	GE287702
61931658	CBYT3679.b1	GE287703
61931659	CBYT3679.g1	GE287704
61931660	CBYT3680.b1	GE287705
61931661	CBYT3680.g1	GE287706
61931662	CBYT3681.b1	GE287707
61931663	CBYT3681.g1	GE287708
61931664	CBYT3682.b1	GE287709
61931665	CBYT3682.g1	GE287710
61931666	CBYT3685.b1	GE287711
61931667	CBYT3686.b1	GE287712
61931668	CBYT3686.g1	GE287713
61931669	CBYT3687.b1	GE287714
61931670	CBYT3688.b1	GE287715
61931671	CBYT3689.b1	GE287716
61931672	CBYT3689.g1	GE287717
61931673	CBYT3690.b1	GE287718
61931674	CBYT3690.g1	GE287719
61931675	CBYT3691.g1	GE287720
61931676	CBYT3693.b1	GE287721
61931677	CBYT3693.g1	GE287722
61931678	CBYT3694.b1	GE287723
61931679	CBYT3694.g1	GE287724
61931680	CBYT3695.b1	GE287725
61931681	CBYT3695.g1	GE287726
61931682	CBYT3696.b1	GE287727
61931683	CBYT3696.g1	GE287728
61931684	CBYT3697.b1	GE287729
61931685	CBYT3698.g1	GE287730
61931686	CBYT3699.b1	GE287731
61931687	CBYT3699.g1	GE287732
61931688	CBYT3700.b1	GE287733
61931689	CBYT3700.g1	GE287734
61931690	CBYT3701.b1	GE287735
61931691	CBYT3701.g1	GE287736
61931692	CBYT3702.g1	GE287737
61931693	CBYT3703.b1	GE287738
61931694	CBYT3703.g1	GE287739
61931695	CBYT3704.b1	GE287740
61931696	CBYT3704.g1	GE287741
61931697	CBYT3705.b1	GE287742
61931698	CBYT3705.g1	GE287743
61931699	CBYT3708.b1	GE287744
61931700	CBYT3708.g1	GE287745
61931701	CBYT3709.b1	GE287746
61931702	CBYT3709.g1	GE287747
61931703	CBYT3710.b1	GE287748
61931704	CBYT3710.g1	GE287749
61931705	CBYT3711.b1	GE287750
61931706	CBYT3711.g1	GE287751
61931707	CBYT3712.b1	GE287752
61931708	CBYT3712.g1	GE287753
61931709	CBYT3714.b1	GE287754
61931710	CBYT3714.g1	GE287755
61931711	CBYT3715.b1	GE287756
61931712	CBYT3715.g1	GE287757

61931713	CBYT3716.b1	GE287758
61931714	CBYT3716.g1	GE287759
61931715	CBYT3717.b1	GE287760
61931716	CBYT3717.g1	GE287761
61931717	CBYT3718.b1	GE287762
61931718	CBYT3718.g1	GE287763
61931719	CBYT3719.b1	GE287764
61931720	CBYT3719.g1	GE287765
61931721	CBYT3720.g1	GE287766
61931722	CBYT3721.g1	GE287767
61931723	CBYT3723.b1	GE287768
61931724	CBYT3723.g1	GE287769
61931725	CBYT3724.g1	GE287770
61931726	CBYT3725.b1	GE287771
61931727	CBYT3725.g1	GE287772
61931728	CBYT3726.b1	GE287773
61931729	CBYT3726.g1	GE287774
61931730	CBYT3727.b1	GE287775
61931731	CBYT3727.g1	GE287776
61931732	CBYT3728.g1	GE287777
61931733	CBYT3729.b1	GE287778
61931734	CBYT3729.g1	GE287779
61931735	CBYT3730.b1	GE287780
61931736	CBYT3730.g1	GE287781
61931737	CBYT3731.b1	GE287782
61931738	CBYT3731.g1	GE287783
61931739	CBYT3732.b1	GE287784
61931740	CBYT3732.g1	GE287785
61931741	CBYT3733.b1	GE287786
61931742	CBYT3733.g1	GE287787
61931743	CBYT3734.b1	GE287788
61931744	CBYT3734.g1	GE287789
61931745	CBYT3735.g1	GE287790
61931746	CBYT3736.g1	GE287791
61931747	CBYT3737.g1	GE287792
61931748	CBYT3738.g1	GE287793
61931749	CBYT3739.b1	GE287794
61931750	CBYT3739.g1	GE287795
61931751	CBYT3742.b1	GE287796
61931752	CBYT3742.g1	GE287797
61931753	CBYT3745.b1	GE287798
61931754	CBYT3745.g1	GE287799
61931755	CBYT3746.b1	GE287800
61931756	CBYT3746.g1	GE287801
61931757	CBYT3747.b1	GE287802
61931758	CBYT3747.g1	GE287803
61931759	CBYT3748.b1	GE287804
61931760	CBYT3748.g1	GE287805
61931761	CBYT3749.b1	GE287806
61931762	CBYT3749.g1	GE287807
61931763	CBYT3750.b1	GE287808
61931764	CBYT3750.g1	GE287809
61931765	CBYT3751.b1	GE287810
61931766	CBYT3751.g1	GE287811
61931767	CBYT3752.b1	GE287812
61931768	CBYT3752.g1	GE287813
61931769	CBYT3753.b1	GE287814
61931770	CBYT3754.b1	GE287815
61931771	CBYT3754.g1	GE287816
61931772	CBYT3755.b1	GE287817
61931773	CBYT3755.g1	GE287818
61931774	CBYT3756.b1	GE287819
61931775	CBYT3756.g1	GE287820

61931776	CBYT3757.b1	GE287821
61931777	CBYT3757.g1	GE287822
61931778	CBYT3758.b1	GE287823
61931779	CBYT3758.g1	GE287824
61931780	CBYT3759.b1	GE287825
61931781	CBYT3759.g1	GE287826
61931782	CBYT3760.b1	GE287827
61931783	CBYT3760.g1	GE287828
61931784	CBYT3762.b1	GE287829
61931785	CBYT3762.g1	GE287830
61931786	CBYT3764.b1	GE287831
61931787	CBYT3764.g1	GE287832
61931788	CBYT3765.g1	GE287833
61931789	CBYT3766.b1	GE287834
61931790	CBYT3766.g1	GE287835
61931791	CBYT3767.b1	GE287836
61931792	CBYT3767.g1	GE287837
61931793	CBYT3768.b1	GE287838
61931794	CBYT3768.g1	GE287839
61931795	CBYT3769.b1	GE287840
61931796	CBYT3769.g1	GE287841
61931797	CBYT3770.b1	GE287842
61931798	CBYT3770.g1	GE287843
61931799	CBYT3771.b1	GE287844
61931800	CBYT3771.g1	GE287845
61931801	CBYT3772.b1	GE287846
61931802	CBYT3772.g1	GE287847
61931803	CBYT3773.b1	GE287848
61931804	CBYT3773.g1	GE287849
61931805	CBYT3774.b1	GE287850
61931806	CBYT3774.g1	GE287851
61931807	CBYT3775.b1	GE287852
61931808	CBYT3775.g1	GE287853
61931809	CBYT3776.b1	GE287854
61931810	CBYT3776.g1	GE287855
61931811	CBYT3777.b1	GE287856
61931812	CBYT3777.g1	GE287857
61931813	CBYT3779.b1	GE287858
61931814	CBYT3779.g1	GE287859
61931815	CBYT3780.b1	GE287860
61931816	CBYT3780.g1	GE287861
61931817	CBYT3782.b1	GE287862
61931818	CBYT3782.g1	GE287863
61931819	CBYT3783.b1	GE287864
61931820	CBYT3783.g1	GE287865
61931821	CBYT3785.b1	GE287866
61931822	CBYT3785.g1	GE287867
61931823	CBYT3786.b1	GE287868
61931824	CBYT3786.g1	GE287869
61931825	CBYT3787.b1	GE287870
61931826	CBYT3787.g1	GE287871
61931827	CBYT3788.b1	GE287872
61931828	CBYT3790.b1	GE287873
61931829	CBYT3790.g1	GE287874
61931830	CBYT3791.b1	GE287875
61931831	CBYT3791.g1	GE287876
61931832	CBYT3792.b1	GE287877
61931833	CBYT3792.g1	GE287878
61931834	CBYT3793.g1	GE287879
61931835	CBYT3794.b1	GE287880
61931836	CBYT3795.b1	GE287881
61931837	CBYT3795.g1	GE287882
61931838	CBYT3796.b1	GE287883

61931839	CBYT3797.b1	GE287884
61931840	CBYT3797.g1	GE287885
61931841	CBYT3798.b1	GE287886
61931842	CBYT3798.g1	GE287887
61931843	CBYT3799.g1	GE287888
61931844	CBYT3800.b1	GE287889
61931845	CBYT3800.g1	GE287890
61931846	CBYT3801.b1	GE287891
61931847	CBYT3801.g1	GE287892
61931848	CBYT3803.b1	GE287893
61931849	CBYT3804.b1	GE287894
61931850	CBYT3804.g1	GE287895
61931851	CBYT3805.b1	GE287896
61931852	CBYT3805.g1	GE287897
61931853	CBYT3806.b1	GE287898
61931854	CBYT3806.g1	GE287899
61931855	CBYT3807.b1	GE287900
61931856	CBYT3807.g1	GE287901
61931857	CBYT3808.b1	GE287902
61931858	CBYT3808.g1	GE287903
61931859	CBYT3809.b1	GE287904
61931860	CBYT3809.g1	GE287905
61931861	CBYT3810.b1	GE287906
61931862	CBYT3810.g1	GE287907
61931863	CBYT3811.b1	GE287908
61931864	CBYT3812.b1	GE287909
61931865	CBYT3812.g1	GE287910
61931866	CBYT3813.b1	GE287911
61931867	CBYT3813.g1	GE287912
61931868	CBYT3814.b1	GE287913
61931869	CBYT3814.g1	GE287914
61931870	CBYT3815.b1	GE287915
61931871	CBYT3815.g1	GE287916
61931872	CBYT3816.b1	GE287917
61931873	CBYT3816.g1	GE287918
61931874	CBYT3817.b1	GE287919
61931875	CBYT3817.g1	GE287920
61931876	CBYT3818.b1	GE287921
61931877	CBYT3818.g1	GE287922
61931878	CBYT3819.b1	GE287923
61931879	CBYT3819.g1	GE287924
61931880	CBYT3820.b1	GE287925
61931881	CBYT3821.b1	GE287926
61931882	CBYT3822.b1	GE287927
61931883	CBYT3822.g1	GE287928
61931884	CBYT3823.b1	GE287929
61931885	CBYT3823.g1	GE287930
61931886	CBYT3824.b1	GE287931
61931887	CBYT3825.b1	GE287932
61931888	CBYT3825.g1	GE287933
61931889	CBYT3826.b1	GE287934
61931890	CBYT3826.g1	GE287935
61931891	CBYT3827.b1	GE287936
61931892	CBYT3827.g1	GE287937
61931893	CBYT3828.b1	GE287938
61931894	CBYT3828.g1	GE287939
61931895	CBYT3829.b1	GE287940
61931896	CBYT3829.g1	GE287941
61931897	CBYT3830.b1	GE287942
61931898	CBYT3830.g1	GE287943
61931899	CBYT3831.b1	GE287944
61931900	CBYT3831.g1	GE287945
61931901	CBYT3833.g1	GE287946

61931902	CBYT3834.b1	GE287947
61931903	CBYT3834.g1	GE287948
61931904	CBYT3835.b1	GE287949
61931905	CBYT3835.g1	GE287950
61931906	CBYT3836.b1	GE287951
61931907	CBYT3836.g1	GE287952
61931908	CBYT3837.b1	GE287953
61931909	CBYT3837.g1	GE287954
61931910	CBYT3838.b1	GE287955
61931911	CBYT3838.g1	GE287956
61931912	CBYT3839.b1	GE287957
61931913	CBYT3842.b1	GE287958
61931914	CBYT3842.g1	GE287959
61931915	CBYT3843.b1	GE287960
61931916	CBYT3844.b1	GE287961
61931917	CBYT3844.g1	GE287962
61931918	CBYT3845.b1	GE287963
61931919	CBYT3845.g1	GE287964
61931920	CBYT3846.b1	GE287965
61931921	CBYT3846.g1	GE287966
61931922	CBYT3847.b1	GE287967
61931923	CBYT3847.g1	GE287968
61931924	CBYT3848.b1	GE287969
61931925	CBYT3848.g1	GE287970
61931926	CBYT3849.b1	GE287971
61931927	CBYT3849.g1	GE287972
61931928	CBYT385.b1	GE287973
61931929	CBYT385.g2	GE287974
61931930	CBYT3850.b1	GE287975
61931931	CBYT3850.g1	GE287976
61931932	CBYT3851.b1	GE287977
61931933	CBYT3851.g1	GE287978
61931934	CBYT3852.b1	GE287979
61931935	CBYT3852.g1	GE287980
61931936	CBYT3853.b1	GE287981
61931937	CBYT3853.g1	GE287982
61931938	CBYT3854.b1	GE287983
61931939	CBYT3855.b1	GE287984
61931940	CBYT3855.g1	GE287985
61931941	CBYT3856.b1	GE287986
61931942	CBYT3856.g1	GE287987
61931943	CBYT3857.b1	GE287988
61931944	CBYT3857.g1	GE287989
61931945	CBYT3858.b1	GE287990
61931946	CBYT3858.g1	GE287991
61931947	CBYT3859.b1	GE287992
61931948	CBYT3859.g1	GE287993
61931949	CBYT386.b1	GE287994
61931950	CBYT386.g2	GE287995
61931951	CBYT3860.b1	GE287996
61931952	CBYT3860.g1	GE287997
61931953	CBYT3862.b1	GE287998
61931954	CBYT3862.g1	GE287999
61931955	CBYT3863.b1	GE288000
61931956	CBYT3863.g1	GE288001
61931957	CBYT3864.b1	GE288002
61931958	CBYT3864.g1	GE288003
61931959	CBYT3865.b1	GE288004
61931960	CBYT3865.g1	GE288005
61931961	CBYT3866.b1	GE288006
61931962	CBYT3866.g1	GE288007
61931963	CBYT3867.b1	GE288008
61931964	CBYT3868.b1	GE288009

61931965	CBYT3868.g1	GE288010
61931966	CBYT3869.b1	GE288011
61931967	CBYT3869.g1	GE288012
61931968	CBYT387.b1	GE288013
61931969	CBYT387.g2	GE288014
61931970	CBYT3870.b1	GE288015
61931971	CBYT3870.g1	GE288016
61931972	CBYT3871.b1	GE288017
61931973	CBYT3871.g1	GE288018
61931974	CBYT3872.b1	GE288019
61931975	CBYT3872.g1	GE288020
61931976	CBYT3873.b1	GE288021
61931977	CBYT3873.g1	GE288022
61931978	CBYT3874.b1	GE288023
61931979	CBYT3874.g1	GE288024
61931980	CBYT3875.b1	GE288025
61931981	CBYT3875.g1	GE288026
61931982	CBYT3876.b1	GE288027
61931983	CBYT3876.g1	GE288028
61931984	CBYT3878.b1	GE288029
61931985	CBYT3878.g1	GE288030
61931986	CBYT3879.b1	GE288031
61931987	CBYT3879.g1	GE288032
61931988	CBYT388.b1	GE288033
61931989	CBYT388.g2	GE288034
61931990	CBYT3880.b1	GE288035
61931991	CBYT3880.g1	GE288036
61931992	CBYT3881.b1	GE288037
61931993	CBYT3881.g1	GE288038
61931994	CBYT3882.b1	GE288039
61931995	CBYT3882.g1	GE288040
61931996	CBYT3883.b1	GE288041
61931997	CBYT3883.g1	GE288042
61931998	CBYT3884.b1	GE288043
61931999	CBYT3884.g1	GE288044
61932000	CBYT3885.b1	GE288045
61932001	CBYT3885.g1	GE288046
61932002	CBYT3886.b1	GE288047
61932003	CBYT3886.g1	GE288048
61932004	CBYT3887.b1	GE288049
61932005	CBYT3887.g1	GE288050
61932006	CBYT3888.b1	GE288051
61932007	CBYT3888.g1	GE288052
61932008	CBYT3889.b1	GE288053
61932009	CBYT3889.g1	GE288054
61932010	CBYT389.b1	GE288055
61932011	CBYT389.g2	GE288056
61932012	CBYT3890.b1	GE288057
61932013	CBYT3890.g1	GE288058
61932014	CBYT3891.b1	GE288059
61932015	CBYT3892.b1	GE288060
61932016	CBYT3892.g1	GE288061
61932017	CBYT3893.b1	GE288062
61932018	CBYT3893.g1	GE288063
61932019	CBYT3894.b1	GE288064
61932020	CBYT3894.g1	GE288065
61932021	CBYT3895.b1	GE288066
61932022	CBYT3895.g1	GE288067
61932023	CBYT3896.b1	GE288068
61932024	CBYT3896.g1	GE288069
61932025	CBYT3897.b1	GE288070
61932026	CBYT3897.g1	GE288071
61932027	CBYT3898.b1	GE288072

61932028	CBYT3898.g1	GE288073
61932029	CBYT3899.g1	GE288074
61932030	CBYT390.b1	GE288075
61932031	CBYT390.g2	GE288076
61932032	CBYT3900.b1	GE288077
61932033	CBYT3900.g1	GE288078
61932034	CBYT3901.b1	GE288079
61932035	CBYT3901.g1	GE288080
61932036	CBYT3902.b1	GE288081
61932037	CBYT3902.g1	GE288082
61932038	CBYT3903.b1	GE288083
61932039	CBYT3903.g1	GE288084
61932040	CBYT3904.b1	GE288085
61932041	CBYT3904.g1	GE288086
61932042	CBYT3905.b1	GE288087
61932043	CBYT3905.g1	GE288088
61932044	CBYT3906.b1	GE288089
61932045	CBYT3906.g1	GE288090
61932046	CBYT3907.b1	GE288091
61932047	CBYT3907.g1	GE288092
61932048	CBYT3908.b1	GE288093
61932049	CBYT3908.g1	GE288094
61932050	CBYT3909.b1	GE288095
61932051	CBYT3909.g1	GE288096
61932052	CBYT391.b1	GE288097
61932053	CBYT391.g2	GE288098
61932054	CBYT3910.b1	GE288099
61932055	CBYT3910.g1	GE288100
61932056	CBYT3911.b1	GE288101
61932057	CBYT3911.g1	GE288102
61932058	CBYT3912.b1	GE288103
61932059	CBYT3912.g1	GE288104
61932060	CBYT3913.b1	GE288105
61932061	CBYT3913.g1	GE288106
61932062	CBYT3914.g1	GE288107
61932063	CBYT3915.b1	GE288108
61932064	CBYT3915.g1	GE288109
61932065	CBYT3916.b1	GE288110
61932066	CBYT3916.g1	GE288111
61932067	CBYT3917.b1	GE288112
61932068	CBYT3917.g1	GE288113
61932069	CBYT3918.b1	GE288114
61932070	CBYT3919.b1	GE288115
61932071	CBYT3919.g1	GE288116
61932072	CBYT392.b1	GE288117
61932073	CBYT392.g2	GE288118
61932074	CBYT3920.b1	GE288119
61932075	CBYT3920.g1	GE288120
61932076	CBYT3921.b1	GE288121
61932077	CBYT3921.g1	GE288122
61932078	CBYT3922.b1	GE288123
61932079	CBYT3922.g1	GE288124
61932080	CBYT3923.b1	GE288125
61932081	CBYT3923.g1	GE288126
61932082	CBYT3924.b1	GE288127
61932083	CBYT3924.g1	GE288128
61932084	CBYT3925.b1	GE288129
61932085	CBYT3925.g1	GE288130
61932086	CBYT3926.b1	GE288131
61932087	CBYT3926.g1	GE288132
61932088	CBYT3927.b1	GE288133
61932089	CBYT3927.g1	GE288134
61932090	CBYT3928.b1	GE288135

61932091	CBYT3928.g1	GE288136
61932092	CBYT3929.g1	GE288137
61932093	CBYT393.b1	GE288138
61932094	CBYT393.g2	GE288139
61932095	CBYT3930.b1	GE288140
61932096	CBYT3930.g1	GE288141
61932097	CBYT3931.b1	GE288142
61932098	CBYT3931.g1	GE288143
61932099	CBYT3933.b1	GE288144
61932100	CBYT3933.g1	GE288145
61932101	CBYT3934.b1	GE288146
61932102	CBYT3934.g1	GE288147
61932103	CBYT3935.b1	GE288148
61932104	CBYT3935.g1	GE288149
61932105	CBYT3936.g1	GE288150
61932106	CBYT3937.g1	GE288151
61932107	CBYT3938.b1	GE288152
61932108	CBYT3938.g1	GE288153
61932109	CBYT3939.b1	GE288154
61932110	CBYT3939.g1	GE288155
61932111	CBYT394.b1	GE288156
61932112	CBYT394.g2	GE288157
61932113	CBYT3940.b1	GE288158
61932114	CBYT3940.g1	GE288159
61932115	CBYT3941.b1	GE288160
61932116	CBYT3941.g1	GE288161
61932117	CBYT3942.b1	GE288162
61932118	CBYT3942.g1	GE288163
61932119	CBYT3943.b1	GE288164
61932120	CBYT3944.b1	GE288165
61932121	CBYT3944.g1	GE288166
61932122	CBYT3945.b1	GE288167
61932123	CBYT3945.g1	GE288168
61932124	CBYT3946.b1	GE288169
61932125	CBYT3946.g1	GE288170
61932126	CBYT3947.b1	GE288171
61932127	CBYT3947.g1	GE288172
61932128	CBYT3948.b1	GE288173
61932129	CBYT3948.g1	GE288174
61932130	CBYT3949.b1	GE288175
61932131	CBYT3949.g1	GE288176
61932132	CBYT395.b1	GE288177
61932133	CBYT395.g2	GE288178
61932134	CBYT3950.b1	GE288179
61932135	CBYT3950.g1	GE288180
61932136	CBYT3951.b1	GE288181
61932137	CBYT3951.g1	GE288182
61932138	CBYT3952.b1	GE288183
61932139	CBYT3952.g1	GE288184
61932140	CBYT3953.b1	GE288185
61932141	CBYT3953.g1	GE288186
61932142	CBYT3954.b1	GE288187
61932143	CBYT3954.g1	GE288188
61932144	CBYT3955.b1	GE288189
61932145	CBYT3955.g1	GE288190
61932146	CBYT3956.b1	GE288191
61932147	CBYT3956.g1	GE288192
61932148	CBYT3957.b1	GE288193
61932149	CBYT3957.g1	GE288194
61932150	CBYT3958.b1	GE288195
61932151	CBYT3958.g1	GE288196
61932152	CBYT3959.b1	GE288197
61932153	CBYT3959.g1	GE288198

61932154	CBYT396.g2	GE288199
61932155	CBYT3960.b1	GE288200
61932156	CBYT3960.g1	GE288201
61932157	CBYT3961.b1	GE288202
61932158	CBYT3961.g1	GE288203
61932159	CBYT3962.b1	GE288204
61932160	CBYT3962.g1	GE288205
61932161	CBYT3963.b1	GE288206
61932162	CBYT3963.g1	GE288207
61932163	CBYT3964.b1	GE288208
61932164	CBYT3965.b1	GE288209
61932165	CBYT3965.g1	GE288210
61932166	CBYT3966.b1	GE288211
61932167	CBYT3966.g1	GE288212
61932168	CBYT3967.b1	GE288213
61932169	CBYT3967.g1	GE288214
61932170	CBYT3968.g1	GE288215
61932171	CBYT3969.b1	GE288216
61932172	CBYT3969.g1	GE288217
61932173	CBYT397.g2	GE288218
61932174	CBYT3970.b1	GE288219
61932175	CBYT3970.g1	GE288220
61932176	CBYT3971.b1	GE288221
61932177	CBYT3971.g1	GE288222
61932178	CBYT3972.b1	GE288223
61932179	CBYT3972.g1	GE288224
61932180	CBYT3974.b1	GE288225
61932181	CBYT3974.g1	GE288226
61932182	CBYT3976.b1	GE288227
61932183	CBYT3976.g1	GE288228
61932184	CBYT3977.b1	GE288229
61932185	CBYT3977.g1	GE288230
61932186	CBYT3978.b1	GE288231
61932187	CBYT3979.g1	GE288232
61932188	CBYT398.b1	GE288233
61932189	CBYT398.g2	GE288234
61932190	CBYT3980.b1	GE288235
61932191	CBYT3980.g1	GE288236
61932192	CBYT3981.g1	GE288237
61932193	CBYT3982.b1	GE288238
61932194	CBYT3983.b1	GE288239
61932195	CBYT3984.b1	GE288240
61932196	CBYT3985.b1	GE288241
61932197	CBYT3985.g1	GE288242
61932198	CBYT3986.b1	GE288243
61932199	CBYT3987.b1	GE288244
61932200	CBYT3987.g1	GE288245
61932201	CBYT3988.b1	GE288246
61932202	CBYT3988.g1	GE288247
61932203	CBYT3989.b1	GE288248
61932204	CBYT3989.g1	GE288249
61932205	CBYT399.b1	GE288250
61932206	CBYT399.g2	GE288251
61932207	CBYT3990.b1	GE288252
61932208	CBYT3990.g1	GE288253
61932209	CBYT3991.b1	GE288254
61932210	CBYT3992.b1	GE288255
61932211	CBYT3992.g1	GE288256
61932212	CBYT3993.g1	GE288257
61932213	CBYT3994.b1	GE288258
61932214	CBYT3994.g1	GE288259
61932215	CBYT3995.b1	GE288260
61932216	CBYT3996.b1	GE288261

61932217	CBYT3996.g1	GE288262
61932218	CBYT3997.b1	GE288263
61932219	CBYT3997.g1	GE288264
61932220	CBYT3998.b1	GE288265
61932221	CBYT3998.g1	GE288266
61932222	CBYT3999.b1	GE288267
61932223	CBYT3999.g1	GE288268
61932224	CBYT400.b1	GE288269
61932225	CBYT400.g2	GE288270
61932226	CBYT4000.b1	GE288271
61932227	CBYT4000.g1	GE288272
61932228	CBYT4001.b1	GE288273
61932229	CBYT4001.g1	GE288274
61932230	CBYT4002.b1	GE288275
61932231	CBYT4002.g1	GE288276
61932232	CBYT4003.b1	GE288277
61932233	CBYT4003.g1	GE288278
61932234	CBYT4004.b1	GE288279
61932235	CBYT4004.g1	GE288280
61932236	CBYT4005.g1	GE288281
61932237	CBYT4006.b1	GE288282
61932238	CBYT4007.b1	GE288283
61932239	CBYT4007.g1	GE288284
61932240	CBYT4008.b1	GE288285
61932241	CBYT4008.g1	GE288286
61932242	CBYT4009.b1	GE288287
61932243	CBYT4009.g1	GE288288
61932244	CBYT401.b1	GE288289
61932245	CBYT401.g2	GE288290
61932246	CBYT4010.b1	GE288291
61932247	CBYT4010.g1	GE288292
61932248	CBYT4011.b1	GE288293
61932249	CBYT4011.g1	GE288294
61932250	CBYT4012.b1	GE288295
61932251	CBYT4012.g1	GE288296
61932252	CBYT4013.b1	GE288297
61932253	CBYT4013.g1	GE288298
61932254	CBYT4014.b1	GE288299
61932255	CBYT4014.g1	GE288300
61932256	CBYT4015.b1	GE288301
61932257	CBYT4015.g1	GE288302
61932258	CBYT4016.b1	GE288303
61932259	CBYT4016.g1	GE288304
61932260	CBYT4017.g1	GE288305
61932261	CBYT4018.b1	GE288306
61932262	CBYT4018.g1	GE288307
61932263	CBYT4019.b1	GE288308
61932264	CBYT4019.g1	GE288309
61932265	CBYT402.b1	GE288310
61932266	CBYT402.g2	GE288311
61932267	CBYT4020.b1	GE288312
61932268	CBYT4020.g1	GE288313
61932269	CBYT4021.b1	GE288314
61932270	CBYT4021.g1	GE288315
61932271	CBYT4022.b1	GE288316
61932272	CBYT4022.g1	GE288317
61932273	CBYT4023.b1	GE288318
61932274	CBYT4023.g1	GE288319
61932275	CBYT4024.b1	GE288320
61932276	CBYT4024.g1	GE288321
61932277	CBYT4025.b1	GE288322
61932278	CBYT4026.b1	GE288323
61932279	CBYT4027.b1	GE288324

61932280	CBYT4027.g1	GE288325
61932281	CBYT4028.b1	GE288326
61932282	CBYT4028.g1	GE288327
61932283	CBYT4029.b1	GE288328
61932284	CBYT4029.g1	GE288329
61932285	CBYT4030.b1	GE288330
61932286	CBYT4030.g1	GE288331
61932287	CBYT4031.b1	GE288332
61932288	CBYT4031.g1	GE288333
61932289	CBYT4032.b1	GE288334
61932290	CBYT4032.g1	GE288335
61932291	CBYT4033.b1	GE288336
61932292	CBYT4033.g1	GE288337
61932293	CBYT4034.b1	GE288338
61932294	CBYT4034.g1	GE288339
61932295	CBYT4035.b1	GE288340
61932296	CBYT4035.g1	GE288341
61932297	CBYT4036.b1	GE288342
61932298	CBYT4036.g1	GE288343
61932299	CBYT4037.b1	GE288344
61932300	CBYT4037.g1	GE288345
61932301	CBYT4038.b1	GE288346
61932302	CBYT4039.b1	GE288347
61932303	CBYT4039.g1	GE288348
61932304	CBYT404.b1	GE288349
61932305	CBYT404.g2	GE288350
61932306	CBYT4040.b1	GE288351
61932307	CBYT4040.g1	GE288352
61932308	CBYT4041.b1	GE288353
61932309	CBYT4041.g1	GE288354
61932310	CBYT4042.b1	GE288355
61932311	CBYT4042.g1	GE288356
61932312	CBYT4044.b1	GE288357
61932313	CBYT4044.g1	GE288358
61932314	CBYT4045.b1	GE288359
61932315	CBYT4045.g1	GE288360
61932316	CBYT4046.b1	GE288361
61932317	CBYT4046.g1	GE288362
61932318	CBYT4047.b1	GE288363
61932319	CBYT4048.b1	GE288364
61932320	CBYT4048.g1	GE288365
61932321	CBYT4049.b1	GE288366
61932322	CBYT4049.g1	GE288367
61932323	CBYT405.b1	GE288368
61932324	CBYT405.g2	GE288369
61932325	CBYT4050.b1	GE288370
61932326	CBYT4050.g1	GE288371
61932327	CBYT4051.b1	GE288372
61932328	CBYT4051.g1	GE288373
61932329	CBYT4052.b1	GE288374
61932330	CBYT4052.g1	GE288375
61932331	CBYT4053.b1	GE288376
61932332	CBYT4053.g1	GE288377
61932333	CBYT4054.b1	GE288378
61932334	CBYT4054.g1	GE288379
61932335	CBYT4055.b1	GE288380
61932336	CBYT4055.g1	GE288381
61932337	CBYT4056.b1	GE288382
61932338	CBYT4056.g1	GE288383
61932339	CBYT4057.b1	GE288384
61932340	CBYT4057.g1	GE288385
61932341	CBYT4058.b1	GE288386
61932342	CBYT4058.g1	GE288387

61932343	CBYT4059.b1	GE288388
61932344	CBYT4059.g1	GE288389
61932345	CBYT406.b1	GE288390
61932346	CBYT4060.b1	GE288391
61932347	CBYT4060.g1	GE288392
61932348	CBYT4061.b1	GE288393
61932349	CBYT4061.g1	GE288394
61932350	CBYT4062.b1	GE288395
61932351	CBYT4062.g1	GE288396
61932352	CBYT4064.b1	GE288397
61932353	CBYT4064.g1	GE288398
61932354	CBYT4065.b1	GE288399
61932355	CBYT4065.g1	GE288400
61932356	CBYT4066.b1	GE288401
61932357	CBYT4066.g1	GE288402
61932358	CBYT4067.b1	GE288403
61932359	CBYT4067.g1	GE288404
61932360	CBYT4068.b1	GE288405
61932361	CBYT4068.g1	GE288406
61932362	CBYT4069.g1	GE288407
61932363	CBYT407.b1	GE288408
61932364	CBYT407.g2	GE288409
61932365	CBYT4070.b1	GE288410
61932366	CBYT4070.g1	GE288411
61932367	CBYT4071.b1	GE288412
61932368	CBYT4072.b1	GE288413
61932369	CBYT4073.b1	GE288414
61932370	CBYT4073.g1	GE288415
61932371	CBYT4074.b1	GE288416
61932372	CBYT4074.g1	GE288417
61932373	CBYT4075.b1	GE288418
61932374	CBYT4075.g1	GE288419
61932375	CBYT4076.b1	GE288420
61932376	CBYT4076.g1	GE288421
61932377	CBYT4077.b1	GE288422
61932378	CBYT4077.g1	GE288423
61932379	CBYT4078.b1	GE288424
61932380	CBYT4078.g1	GE288425
61932381	CBYT4079.b1	GE288426
61932382	CBYT4079.g1	GE288427
61932383	CBYT408.g2	GE288428
61932384	CBYT4080.b1	GE288429
61932385	CBYT4080.g1	GE288430
61932386	CBYT4081.b1	GE288431
61932387	CBYT4081.g1	GE288432
61932388	CBYT4082.b1	GE288433
61932389	CBYT4082.g1	GE288434
61932390	CBYT4083.g1	GE288435
61932391	CBYT4084.b1	GE288436
61932392	CBYT4084.g1	GE288437
61932393	CBYT4085.b1	GE288438
61932394	CBYT4085.g1	GE288439
61932395	CBYT4086.b1	GE288440
61932396	CBYT4086.g1	GE288441
61932397	CBYT4087.b1	GE288442
61932398	CBYT4087.g1	GE288443
61932399	CBYT4088.b1	GE288444
61932400	CBYT4088.g1	GE288445
61932401	CBYT4089.b1	GE288446
61932402	CBYT4089.g1	GE288447
61932403	CBYT409.b1	GE288448
61932404	CBYT409.g2	GE288449
61932405	CBYT4090.b1	GE288450

61932406	CBYT4090.g1	GE288451
61932407	CBYT4091.b1	GE288452
61932408	CBYT4091.g1	GE288453
61932409	CBYT4092.b1	GE288454
61932410	CBYT4092.g1	GE288455
61932411	CBYT4093.b1	GE288456
61932412	CBYT4093.g1	GE288457
61932413	CBYT4095.b1	GE288458
61932414	CBYT4095.g1	GE288459
61932415	CBYT4096.b1	GE288460
61932416	CBYT4096.g1	GE288461
61932417	CBYT4097.g1	GE288462
61932418	CBYT4098.b1	GE288463
61932419	CBYT4098.g1	GE288464
61932420	CBYT410.b1	GE288465
61932421	CBYT410.g2	GE288466
61932422	CBYT4100.b1	GE288467
61932423	CBYT4100.g1	GE288468
61932424	CBYT4101.b1	GE288469
61932425	CBYT4101.g1	GE288470
61932426	CBYT4102.b1	GE288471
61932427	CBYT4102.g1	GE288472
61932428	CBYT4103.g1	GE288473
61932429	CBYT4104.b1	GE288474
61932430	CBYT4104.g1	GE288475
61932431	CBYT4105.b1	GE288476
61932432	CBYT4105.g1	GE288477
61932433	CBYT4106.b1	GE288478
61932434	CBYT4106.g1	GE288479
61932435	CBYT4107.b1	GE288480
61932436	CBYT4107.g1	GE288481
61932437	CBYT4109.b1	GE288482
61932438	CBYT4109.g1	GE288483
61932439	CBYT411.b1	GE288484
61932440	CBYT411.g2	GE288485
61932441	CBYT4110.b1	GE288486
61932442	CBYT4110.g1	GE288487
61932443	CBYT4111.b1	GE288488
61932444	CBYT4111.g1	GE288489
61932445	CBYT4112.b1	GE288490
61932446	CBYT4112.g1	GE288491
61932447	CBYT4113.b1	GE288492
61932448	CBYT4113.g1	GE288493
61932449	CBYT4114.b1	GE288494
61932450	CBYT4114.g1	GE288495
61932451	CBYT4115.b1	GE288496
61932452	CBYT4115.g1	GE288497
61932453	CBYT4117.b1	GE288498
61932454	CBYT4117.g1	GE288499
61932455	CBYT4118.b1	GE288500
61932456	CBYT4118.g1	GE288501
61932457	CBYT4119.b1	GE288502
61932458	CBYT4119.g1	GE288503
61932459	CBYT412.b1	GE288504
61932460	CBYT412.g2	GE288505
61932461	CBYT4120.b1	GE288506
61932462	CBYT4120.g1	GE288507
61932463	CBYT4121.b1	GE288508
61932464	CBYT4121.g1	GE288509
61932465	CBYT4122.b1	GE288510
61932466	CBYT4122.g1	GE288511
61932467	CBYT4123.b1	GE288512
61932468	CBYT4123.g1	GE288513

61932469	CBYT4124.b1	GE288514
61932470	CBYT4124.g1	GE288515
61932471	CBYT4125.b1	GE288516
61932472	CBYT4125.g1	GE288517
61932473	CBYT4126.b1	GE288518
61932474	CBYT4126.g1	GE288519
61932475	CBYT4127.b1	GE288520
61932476	CBYT4127.g1	GE288521
61932477	CBYT4129.b1	GE288522
61932478	CBYT4129.g1	GE288523
61932479	CBYT413.b1	GE288524
61932480	CBYT413.g2	GE288525
61932481	CBYT4130.b1	GE288526
61932482	CBYT4130.g1	GE288527
61932483	CBYT4131.b1	GE288528
61932484	CBYT4131.g1	GE288529
61932485	CBYT4132.b1	GE288530
61932486	CBYT4132.g1	GE288531
61932487	CBYT4133.b1	GE288532
61932488	CBYT4133.g1	GE288533
61932489	CBYT4134.b1	GE288534
61932490	CBYT4134.g1	GE288535
61932491	CBYT4135.b1	GE288536
61932492	CBYT4135.g1	GE288537
61932493	CBYT4136.b1	GE288538
61932494	CBYT4136.g1	GE288539
61932495	CBYT4137.b1	GE288540
61932496	CBYT4137.g1	GE288541
61932497	CBYT4138.b1	GE288542
61932498	CBYT4138.g1	GE288543
61932499	CBYT4139.b1	GE288544
61932500	CBYT414.b1	GE288545
61932501	CBYT414.g2	GE288546
61932502	CBYT4140.b1	GE288547
61932503	CBYT4140.g1	GE288548
61932504	CBYT4141.b1	GE288549
61932505	CBYT4141.g1	GE288550
61932506	CBYT4142.g1	GE288551
61932507	CBYT4143.b1	GE288552
61932508	CBYT4143.g1	GE288553
61932509	CBYT4144.b1	GE288554
61932510	CBYT4144.g1	GE288555
61932511	CBYT4145.b1	GE288556
61932512	CBYT4145.g1	GE288557
61932513	CBYT4146.b1	GE288558
61932514	CBYT4146.g1	GE288559
61932515	CBYT4147.b1	GE288560
61932516	CBYT4147.g1	GE288561
61932517	CBYT4148.b1	GE288562
61932518	CBYT4148.g1	GE288563
61932519	CBYT4149.b1	GE288564
61932520	CBYT4149.g1	GE288565
61932521	CBYT415.b1	GE288566
61932522	CBYT415.g2	GE288567
61932523	CBYT4150.b1	GE288568
61932524	CBYT4150.g1	GE288569
61932525	CBYT4151.b1	GE288570
61932526	CBYT4151.g1	GE288571
61932527	CBYT4152.b1	GE288572
61932528	CBYT4152.g1	GE288573
61932529	CBYT4153.b1	GE288574
61932530	CBYT4153.g1	GE288575
61932531	CBYT4155.g1	GE288576

61932532	CBYT4156.b1	GE288577
61932533	CBYT4157.b1	GE288578
61932534	CBYT4157.g1	GE288579
61932535	CBYT4158.b1	GE288580
61932536	CBYT4158.g1	GE288581
61932537	CBYT4159.b1	GE288582
61932538	CBYT4159.g1	GE288583
61932539	CBYT4160.b1	GE288584
61932540	CBYT4160.g1	GE288585
61932541	CBYT4161.b1	GE288586
61932542	CBYT4161.g1	GE288587
61932543	CBYT4163.b1	GE288588
61932544	CBYT4163.g1	GE288589
61932545	CBYT4164.b1	GE288590
61932546	CBYT4164.g1	GE288591
61932547	CBYT4165.b1	GE288592
61932548	CBYT4165.g1	GE288593
61932549	CBYT4166.b1	GE288594
61932550	CBYT4166.g1	GE288595
61932551	CBYT4167.b1	GE288596
61932552	CBYT4167.g1	GE288597
61932553	CBYT4168.b1	GE288598
61932554	CBYT4168.g1	GE288599
61932555	CBYT4169.b1	GE288600
61932556	CBYT4169.g1	GE288601
61932557	CBYT417.b1	GE288602
61932558	CBYT4170.b1	GE288603
61932559	CBYT4170.g1	GE288604
61932560	CBYT4171.b1	GE288605
61932561	CBYT4171.g1	GE288606
61932562	CBYT4172.b1	GE288607
61932563	CBYT4172.g1	GE288608
61932564	CBYT4173.b1	GE288609
61932565	CBYT4173.g1	GE288610
61932566	CBYT4174.b1	GE288611
61932567	CBYT4174.g1	GE288612
61932568	CBYT4175.b1	GE288613
61932569	CBYT4175.g1	GE288614
61932570	CBYT4176.g1	GE288615
61932571	CBYT4177.b1	GE288616
61932572	CBYT4177.g1	GE288617
61932573	CBYT4178.b1	GE288618
61932574	CBYT4178.g1	GE288619
61932575	CBYT4179.g1	GE288620
61932576	CBYT418.b1	GE288621
61932577	CBYT418.g2	GE288622
61932578	CBYT4180.g1	GE288623
61932579	CBYT4181.b1	GE288624
61932580	CBYT4181.g1	GE288625
61932581	CBYT4183.b1	GE288626
61932582	CBYT4184.b1	GE288627
61932583	CBYT4184.g1	GE288628
61932584	CBYT4185.b1	GE288629
61932585	CBYT4185.g1	GE288630
61932586	CBYT4186.b1	GE288631
61932587	CBYT4186.g1	GE288632
61932588	CBYT4187.b1	GE288633
61932589	CBYT4187.g1	GE288634
61932590	CBYT4188.b1	GE288635
61932591	CBYT4189.b1	GE288636
61932592	CBYT4189.g1	GE288637
61932593	CBYT4190.b1	GE288638
61932594	CBYT4190.g1	GE288639

61932595	CBYT4191.b1	GE288640
61932596	CBYT4191.g1	GE288641
61932597	CBYT4192.b1	GE288642
61932598	CBYT4193.b1	GE288643
61932599	CBYT4193.g1	GE288644
61932600	CBYT4194.b1	GE288645
61932601	CBYT4194.g1	GE288646
61932602	CBYT4195.b1	GE288647
61932603	CBYT4195.g1	GE288648
61932604	CBYT4196.g1	GE288649
61932605	CBYT4198.b1	GE288650
61932606	CBYT4199.b1	GE288651
61932607	CBYT4199.g1	GE288652
61932608	CBYT420.g2	GE288653
61932609	CBYT4200.b1	GE288654
61932610	CBYT4200.g1	GE288655
61932611	CBYT4201.b1	GE288656
61932612	CBYT4201.g1	GE288657
61932613	CBYT4202.b1	GE288658
61932614	CBYT4202.g1	GE288659
61932615	CBYT4203.b1	GE288660
61932616	CBYT4203.g1	GE288661
61932617	CBYT4204.b1	GE288662
61932618	CBYT4204.g1	GE288663
61932619	CBYT4205.b1	GE288664
61932620	CBYT4205.g1	GE288665
61932621	CBYT4206.b1	GE288666
61932622	CBYT4206.g1	GE288667
61932623	CBYT4207.b1	GE288668
61932624	CBYT4207.g1	GE288669
61932625	CBYT4208.b1	GE288670
61932626	CBYT4208.g1	GE288671
61932627	CBYT4209.b1	GE288672
61932628	CBYT4209.g1	GE288673
61932629	CBYT421.b1	GE288674
61932630	CBYT421.g2	GE288675
61932631	CBYT4210.b1	GE288676
61932632	CBYT4210.g1	GE288677
61932633	CBYT4211.b1	GE288678
61932634	CBYT4212.b1	GE288679
61932635	CBYT4212.g1	GE288680
61932636	CBYT4213.b1	GE288681
61932637	CBYT4214.b1	GE288682
61932638	CBYT4214.g1	GE288683
61932639	CBYT4215.b1	GE288684
61932640	CBYT4215.g1	GE288685
61932641	CBYT4217.b1	GE288686
61932642	CBYT4218.b1	GE288687
61932643	CBYT4218.g1	GE288688
61932644	CBYT4219.b1	GE288689
61932645	CBYT4219.g1	GE288690
61932646	CBYT422.g2	GE288691
61932647	CBYT4221.b1	GE288692
61932648	CBYT4221.g1	GE288693
61932649	CBYT4222.b1	GE288694
61932650	CBYT4222.g1	GE288695
61932651	CBYT4224.b1	GE288696
61932652	CBYT4224.g1	GE288697
61932653	CBYT4225.b1	GE288698
61932654	CBYT4225.g1	GE288699
61932655	CBYT4226.b1	GE288700
61932656	CBYT4226.g1	GE288701
61932657	CBYT4227.b1	GE288702

61932658	CBYT4227.g1	GE288703
61932659	CBYT4228.b1	GE288704
61932660	CBYT4228.g1	GE288705
61932661	CBYT4229.b1	GE288706
61932662	CBYT4229.g1	GE288707
61932663	CBYT423.b1	GE288708
61932664	CBYT423.g2	GE288709
61932665	CBYT4230.b1	GE288710
61932666	CBYT4230.g1	GE288711
61932667	CBYT4231.b1	GE288712
61932668	CBYT4231.g1	GE288713
61932669	CBYT4233.b1	GE288714
61932670	CBYT4233.g1	GE288715
61932671	CBYT4234.b1	GE288716
61932672	CBYT4234.g1	GE288717
61932673	CBYT4235.b1	GE288718
61932674	CBYT4236.b1	GE288719
61932675	CBYT4236.g1	GE288720
61932676	CBYT4237.b1	GE288721
61932677	CBYT4237.g1	GE288722
61932678	CBYT4238.b1	GE288723
61932679	CBYT4238.g1	GE288724
61932680	CBYT4239.b1	GE288725
61932681	CBYT4239.g1	GE288726
61932682	CBYT424.b1	GE288727
61932683	CBYT424.g2	GE288728
61932684	CBYT4240.b1	GE288729
61932685	CBYT4240.g1	GE288730
61932686	CBYT4241.b1	GE288731
61932687	CBYT4241.g1	GE288732
61932688	CBYT4242.b1	GE288733
61932689	CBYT4242.g1	GE288734
61932690	CBYT4243.b1	GE288735
61932691	CBYT4243.g1	GE288736
61932692	CBYT4245.b1	GE288737
61932693	CBYT4245.g1	GE288738
61932694	CBYT4246.b1	GE288739
61932695	CBYT4246.g1	GE288740
61932696	CBYT4247.b1	GE288741
61932697	CBYT4247.g1	GE288742
61932698	CBYT4248.g1	GE288743
61932699	CBYT4249.g1	GE288744
61932700	CBYT425.g2	GE288745
61932701	CBYT4250.b1	GE288746
61932702	CBYT4250.g1	GE288747
61932703	CBYT4251.b1	GE288748
61932704	CBYT4251.g1	GE288749
61932705	CBYT4252.b1	GE288750
61932706	CBYT4252.g1	GE288751
61932707	CBYT4253.b1	GE288752
61932708	CBYT4253.g1	GE288753
61932709	CBYT4254.b1	GE288754
61932710	CBYT4254.g1	GE288755
61932711	CBYT4256.b1	GE288756
61932712	CBYT4256.g1	GE288757
61932713	CBYT4257.b1	GE288758
61932714	CBYT4257.g1	GE288759
61932715	CBYT4258.b1	GE288760
61932716	CBYT4258.g1	GE288761
61932717	CBYT4259.b1	GE288762
61932718	CBYT4259.g1	GE288763
61932719	CBYT426.b1	GE288764
61932720	CBYT426.g2	GE288765

61932721	CBYT4260.b1	GE288766
61932722	CBYT4260.g1	GE288767
61932723	CBYT4261.b1	GE288768
61932724	CBYT4261.g1	GE288769
61932725	CBYT4262.b1	GE288770
61932726	CBYT4262.g1	GE288771
61932727	CBYT4263.b1	GE288772
61932728	CBYT4263.g1	GE288773
61932729	CBYT4264.b1	GE288774
61932730	CBYT4264.g1	GE288775
61932731	CBYT4265.b1	GE288776
61932732	CBYT4265.g1	GE288777
61932733	CBYT4266.b1	GE288778
61932734	CBYT4267.b1	GE288779
61932735	CBYT4267.g1	GE288780
61932736	CBYT4268.b1	GE288781
61932737	CBYT4268.g1	GE288782
61932738	CBYT4269.b1	GE288783
61932739	CBYT4269.g1	GE288784
61932740	CBYT427.b1	GE288785
61932741	CBYT427.g2	GE288786
61932742	CBYT4270.b1	GE288787
61932743	CBYT4270.g1	GE288788
61932744	CBYT4271.b1	GE288789
61932745	CBYT4271.g1	GE288790
61932746	CBYT4272.b1	GE288791
61932747	CBYT4272.g1	GE288792
61932748	CBYT4273.b1	GE288793
61932749	CBYT4273.g1	GE288794
61932750	CBYT4274.b1	GE288795
61932751	CBYT4274.g1	GE288796
61932752	CBYT4275.g1	GE288797
61932753	CBYT4276.b1	GE288798
61932754	CBYT4276.g1	GE288799
61932755	CBYT4277.b1	GE288800
61932756	CBYT4277.g1	GE288801
61932757	CBYT4278.b1	GE288802
61932758	CBYT4278.g1	GE288803
61932759	CBYT428.b1	GE288804
61932760	CBYT428.g2	GE288805
61932761	CBYT4280.b1	GE288806
61932762	CBYT4280.g1	GE288807
61932763	CBYT4281.b1	GE288808
61932764	CBYT4281.g1	GE288809
61932765	CBYT4282.b1	GE288810
61932766	CBYT4282.g1	GE288811
61932767	CBYT4283.b1	GE288812
61932768	CBYT4283.g1	GE288813
61932769	CBYT4284.b1	GE288814
61932770	CBYT4284.g1	GE288815
61932771	CBYT4285.b1	GE288816
61932772	CBYT4285.g1	GE288817
61932773	CBYT4286.b1	GE288818
61932774	CBYT4286.g1	GE288819
61932775	CBYT4287.b1	GE288820
61932776	CBYT4287.g1	GE288821
61932777	CBYT4288.b1	GE288822
61932778	CBYT4288.g1	GE288823
61932779	CBYT4289.b1	GE288824
61932780	CBYT4289.g1	GE288825
61932781	CBYT429.b1	GE288826
61932782	CBYT429.g2	GE288827
61932783	CBYT4290.b1	GE288828

61932784	CBYT4290.g1	GE288829
61932785	CBYT4291.b1	GE288830
61932786	CBYT4291.g1	GE288831
61932787	CBYT4292.b1	GE288832
61932788	CBYT4292.g1	GE288833
61932789	CBYT4293.b1	GE288834
61932790	CBYT4293.g1	GE288835
61932791	CBYT4294.b1	GE288836
61932792	CBYT4294.g1	GE288837
61932793	CBYT4295.b1	GE288838
61932794	CBYT4295.g1	GE288839
61932795	CBYT4296.b1	GE288840
61932796	CBYT4296.g1	GE288841
61932797	CBYT4297.b1	GE288842
61932798	CBYT4297.g1	GE288843
61932799	CBYT4298.b1	GE288844
61932800	CBYT4298.g1	GE288845
61932801	CBYT4299.b1	GE288846
61932802	CBYT4299.g1	GE288847
61932803	CBYT430.b1	GE288848
61932804	CBYT430.g2	GE288849
61932805	CBYT4300.b1	GE288850
61932806	CBYT4300.g1	GE288851
61932807	CBYT4301.b1	GE288852
61932808	CBYT4301.g1	GE288853
61932809	CBYT4302.b1	GE288854
61932810	CBYT4302.g1	GE288855
61932811	CBYT4303.b1	GE288856
61932812	CBYT4303.g1	GE288857
61932813	CBYT4304.b1	GE288858
61932814	CBYT4304.g1	GE288859
61932815	CBYT4305.b1	GE288860
61932816	CBYT4305.g1	GE288861
61932817	CBYT4306.b1	GE288862
61932818	CBYT4306.g1	GE288863
61932819	CBYT4307.b1	GE288864
61932820	CBYT4307.g1	GE288865
61932821	CBYT4308.b1	GE288866
61932822	CBYT4308.g1	GE288867
61932823	CBYT4309.b1	GE288868
61932824	CBYT4309.g1	GE288869
61932825	CBYT431.b1	GE288870
61932826	CBYT431.g2	GE288871
61932827	CBYT4310.b1	GE288872
61932828	CBYT4310.g1	GE288873
61932829	CBYT4311.b1	GE288874
61932830	CBYT4311.g1	GE288875
61932831	CBYT4312.b1	GE288876
61932832	CBYT4312.g1	GE288877
61932833	CBYT4313.b1	GE288878
61932834	CBYT4313.g1	GE288879
61932835	CBYT4314.b1	GE288880
61932836	CBYT4314.g1	GE288881
61932837	CBYT4316.b1	GE288882
61932838	CBYT4316.g1	GE288883
61932839	CBYT4317.b1	GE288884
61932840	CBYT4317.g1	GE288885
61932841	CBYT4318.b1	GE288886
61932842	CBYT4318.g1	GE288887
61932843	CBYT4319.b1	GE288888
61932844	CBYT4319.g1	GE288889
61932845	CBYT432.b1	GE288890
61932846	CBYT432.g2	GE288891

61932847	CBYT4320.b1	GE288892
61932848	CBYT4320.g1	GE288893
61932849	CBYT4321.b1	GE288894
61932850	CBYT4321.g1	GE288895
61932851	CBYT4322.g1	GE288896
61932852	CBYT4323.g1	GE288897
61932853	CBYT4324.b1	GE288898
61932854	CBYT4324.g1	GE288899
61932855	CBYT4325.b1	GE288900
61932856	CBYT4325.g1	GE288901
61932857	CBYT4326.b1	GE288902
61932858	CBYT4326.g1	GE288903
61932859	CBYT4327.b1	GE288904
61932860	CBYT4327.g1	GE288905
61932861	CBYT4328.b1	GE288906
61932862	CBYT4328.g1	GE288907
61932863	CBYT433.b1	GE288908
61932864	CBYT433.g2	GE288909
61932865	CBYT4330.b1	GE288910
61932866	CBYT4330.g1	GE288911
61932867	CBYT4331.b1	GE288912
61932868	CBYT4331.g1	GE288913
61932869	CBYT4332.b1	GE288914
61932870	CBYT4333.b1	GE288915
61932871	CBYT4333.g1	GE288916
61932872	CBYT4334.b1	GE288917
61932873	CBYT4334.g1	GE288918
61932874	CBYT4335.b1	GE288919
61932875	CBYT4335.g1	GE288920
61932876	CBYT4336.b1	GE288921
61932877	CBYT4336.g1	GE288922
61932878	CBYT4337.b1	GE288923
61932879	CBYT4337.g1	GE288924
61932880	CBYT4338.b1	GE288925
61932881	CBYT4338.g1	GE288926
61932882	CBYT434.b1	GE288927
61932883	CBYT434.g2	GE288928
61932884	CBYT4340.b1	GE288929
61932885	CBYT4340.g1	GE288930
61932886	CBYT4341.b1	GE288931
61932887	CBYT4341.g1	GE288932
61932888	CBYT4344.b1	GE288933
61932889	CBYT4344.g1	GE288934
61932890	CBYT4345.b1	GE288935
61932891	CBYT4346.b1	GE288936
61932892	CBYT4346.g1	GE288937
61932893	CBYT4347.b1	GE288938
61932894	CBYT4347.g1	GE288939
61932895	CBYT4348.b1	GE288940
61932896	CBYT4348.g1	GE288941
61932897	CBYT4350.b1	GE288942
61932898	CBYT4350.g1	GE288943
61932899	CBYT4351.b1	GE288944
61932900	CBYT4351.g1	GE288945
61932901	CBYT4352.b1	GE288946
61932902	CBYT4352.g1	GE288947
61932903	CBYT4353.b1	GE288948
61932904	CBYT4353.g1	GE288949
61932905	CBYT4354.g1	GE288950
61932906	CBYT4355.b1	GE288951
61932907	CBYT4355.g1	GE288952
61932908	CBYT4356.g1	GE288953
61932909	CBYT4357.b1	GE288954

61932910	CBYT4358.g1	GE288955
61932911	CBYT4359.b1	GE288956
61932912	CBYT4359.g1	GE288957
61932913	CBYT4360.b1	GE288958
61932914	CBYT4360.g1	GE288959
61932915	CBYT4361.b1	GE288960
61932916	CBYT4361.g1	GE288961
61932917	CBYT4362.b1	GE288962
61932918	CBYT4362.g1	GE288963
61932919	CBYT4363.b1	GE288964
61932920	CBYT4363.g1	GE288965
61932921	CBYT4364.b1	GE288966
61932922	CBYT4364.g1	GE288967
61932923	CBYT4365.b1	GE288968
61932924	CBYT4365.g1	GE288969
61932925	CBYT4366.b1	GE288970
61932926	CBYT4366.g1	GE288971
61932927	CBYT4367.b1	GE288972
61932928	CBYT4367.g1	GE288973
61932929	CBYT4368.b1	GE288974
61932930	CBYT4368.g1	GE288975
61932931	CBYT4369.b1	GE288976
61932932	CBYT4369.g1	GE288977
61932933	CBYT437.b1	GE288978
61932934	CBYT437.g2	GE288979
61932935	CBYT4370.b1	GE288980
61932936	CBYT4370.g1	GE288981
61932937	CBYT4372.b1	GE288982
61932938	CBYT4372.g1	GE288983
61932939	CBYT4373.b1	GE288984
61932940	CBYT4373.g1	GE288985
61932941	CBYT4374.b1	GE288986
61932942	CBYT4374.g1	GE288987
61932943	CBYT4376.g1	GE288988
61932944	CBYT4377.b1	GE288989
61932945	CBYT4378.b1	GE288990
61932946	CBYT4378.g1	GE288991
61932947	CBYT4379.b1	GE288992
61932948	CBYT4379.g1	GE288993
61932949	CBYT438.b1	GE288994
61932950	CBYT438.g2	GE288995
61932951	CBYT4380.b1	GE288996
61932952	CBYT4380.g1	GE288997
61932953	CBYT4381.b1	GE288998
61932954	CBYT4382.b1	GE288999
61932955	CBYT4382.g1	GE289000
61932956	CBYT4383.b1	GE289001
61932957	CBYT4383.g1	GE289002
61932958	CBYT4385.b1	GE289003
61932959	CBYT4385.g1	GE289004
61932960	CBYT4386.b1	GE289005
61932961	CBYT4387.b1	GE289006
61932962	CBYT4387.g1	GE289007
61932963	CBYT4388.b1	GE289008
61932964	CBYT4388.g1	GE289009
61932965	CBYT4389.b1	GE289010
61932966	CBYT4389.g1	GE289011
61932967	CBYT439.b1	GE289012
61932968	CBYT439.g2	GE289013
61932969	CBYT4390.b1	GE289014
61932970	CBYT4390.g1	GE289015
61932971	CBYT4391.b1	GE289016
61932972	CBYT4391.g1	GE289017

61932973	CBYT4393.b1	GE289018
61932974	CBYT4393.g1	GE289019
61932975	CBYT4394.b1	GE289020
61932976	CBYT4395.b1	GE289021
61932977	CBYT4395.g1	GE289022
61932978	CBYT4396.g1	GE289023
61932979	CBYT4397.b1	GE289024
61932980	CBYT4397.g1	GE289025
61932981	CBYT4398.b1	GE289026
61932982	CBYT4398.g1	GE289027
61932983	CBYT4399.b1	GE289028
61932984	CBYT4399.g1	GE289029
61932985	CBYT440.b1	GE289030
61932986	CBYT440.g2	GE289031
61932987	CBYT4400.b1	GE289032
61932988	CBYT4400.g1	GE289033
61932989	CBYT4402.b1	GE289034
61932990	CBYT4402.g1	GE289035
61932991	CBYT4403.b1	GE289036
61932992	CBYT4404.b1	GE289037
61932993	CBYT4404.g1	GE289038
61932994	CBYT4405.b1	GE289039
61932995	CBYT4405.g1	GE289040
61932996	CBYT4406.b1	GE289041
61932997	CBYT4406.g1	GE289042
61932998	CBYT4407.b1	GE289043
61932999	CBYT4407.g1	GE289044
61933000	CBYT4408.b1	GE289045
61933001	CBYT4408.g1	GE289046
61933002	CBYT4409.b1	GE289047
61933003	CBYT4409.g1	GE289048
61933004	CBYT4410.b1	GE289049
61933005	CBYT4410.g1	GE289050
61933006	CBYT4411.b1	GE289051
61933007	CBYT4411.g1	GE289052
61933008	CBYT4413.b1	GE289053
61933009	CBYT4413.g1	GE289054
61933010	CBYT4414.b1	GE289055
61933011	CBYT4414.g1	GE289056
61933012	CBYT4415.b1	GE289057
61933013	CBYT4415.g1	GE289058
61933014	CBYT4416.b1	GE289059
61933015	CBYT4416.g1	GE289060
61933016	CBYT4417.b1	GE289061
61933017	CBYT4417.g1	GE289062
61933018	CBYT4419.b1	GE289063
61933019	CBYT4419.g1	GE289064
61933020	CBYT4422.b1	GE289065
61933021	CBYT4422.g1	GE289066
61933022	CBYT4423.b1	GE289067
61933023	CBYT4423.g1	GE289068
61933024	CBYT4424.b1	GE289069
61933025	CBYT4424.g1	GE289070
61933026	CBYT4425.b1	GE289071
61933027	CBYT4425.g1	GE289072
61933028	CBYT4426.b1	GE289073
61933029	CBYT4426.g1	GE289074
61933030	CBYT4427.b1	GE289075
61933031	CBYT4427.g1	GE289076
61933032	CBYT4428.b1	GE289077
61933033	CBYT4428.g1	GE289078
61933034	CBYT443.b1	GE289079
61933035	CBYT4430.b1	GE289080

61933036	CBYT4430.g1	GE289081
61933037	CBYT4431.b1	GE289082
61933038	CBYT4431.g1	GE289083
61933039	CBYT4432.b1	GE289084
61933040	CBYT4432.g1	GE289085
61933041	CBYT4433.b1	GE289086
61933042	CBYT4433.g1	GE289087
61933043	CBYT4434.b1	GE289088
61933044	CBYT4434.g1	GE289089
61933045	CBYT4435.b1	GE289090
61933046	CBYT4435.g1	GE289091
61933047	CBYT4436.b1	GE289092
61933048	CBYT4436.g1	GE289093
61933049	CBYT4437.b1	GE289094
61933050	CBYT4437.g1	GE289095
61933051	CBYT4438.b1	GE289096
61933052	CBYT4439.b1	GE289097
61933053	CBYT4439.g1	GE289098
61933054	CBYT444.b1	GE289099
61933055	CBYT444.g2	GE289100
61933056	CBYT4440.b1	GE289101
61933057	CBYT4440.g1	GE289102
61933058	CBYT4441.b1	GE289103
61933059	CBYT4441.g1	GE289104
61933060	CBYT4442.b1	GE289105
61933061	CBYT4442.g1	GE289106
61933062	CBYT4443.b1	GE289107
61933063	CBYT4444.b1	GE289108
61933064	CBYT4446.b1	GE289109
61933065	CBYT4446.g1	GE289110
61933066	CBYT4447.b1	GE289111
61933067	CBYT4447.g1	GE289112
61933068	CBYT4448.b1	GE289113
61933069	CBYT4448.g1	GE289114
61933070	CBYT4449.b1	GE289115
61933071	CBYT4449.g1	GE289116
61933072	CBYT445.b1	GE289117
61933073	CBYT445.g2	GE289118
61933074	CBYT4450.b1	GE289119
61933075	CBYT4450.g1	GE289120
61933076	CBYT4451.b1	GE289121
61933077	CBYT4451.g1	GE289122
61933078	CBYT4452.b1	GE289123
61933079	CBYT4452.g1	GE289124
61933080	CBYT4453.b1	GE289125
61933081	CBYT4453.g1	GE289126
61933082	CBYT4455.g1	GE289127
61933083	CBYT4456.b1	GE289128
61933084	CBYT4456.g1	GE289129
61933085	CBYT4457.b1	GE289130
61933086	CBYT4457.g1	GE289131
61933087	CBYT4458.b1	GE289132
61933088	CBYT4459.b1	GE289133
61933089	CBYT4459.g1	GE289134
61933090	CBYT446.b1	GE289135
61933091	CBYT446.g2	GE289136
61933092	CBYT4460.b1	GE289137
61933093	CBYT4460.g1	GE289138
61933094	CBYT4461.b1	GE289139
61933095	CBYT4461.g1	GE289140
61933096	CBYT4462.b1	GE289141
61933097	CBYT4462.g1	GE289142
61933098	CBYT4463.b1	GE289143

61933099	CBYT4463.g1	GE289144
61933100	CBYT4464.b1	GE289145
61933101	CBYT4464.g1	GE289146
61933102	CBYT4466.b1	GE289147
61933103	CBYT4466.g1	GE289148
61933104	CBYT4467.b1	GE289149
61933105	CBYT4467.g1	GE289150
61933106	CBYT4468.b1	GE289151
61933107	CBYT4468.g1	GE289152
61933108	CBYT4469.b1	GE289153
61933109	CBYT4469.g1	GE289154
61933110	CBYT447.b1	GE289155
61933111	CBYT447.g2	GE289156
61933112	CBYT4470.b1	GE289157
61933113	CBYT4470.g1	GE289158
61933114	CBYT4471.b1	GE289159
61933115	CBYT4471.g1	GE289160
61933116	CBYT4472.b1	GE289161
61933117	CBYT4472.g1	GE289162
61933118	CBYT4473.b1	GE289163
61933119	CBYT4473.g1	GE289164
61933120	CBYT4474.b1	GE289165
61933121	CBYT4474.g1	GE289166
61933122	CBYT4475.b1	GE289167
61933123	CBYT4475.g1	GE289168
61933124	CBYT4476.b1	GE289169
61933125	CBYT4476.g1	GE289170
61933126	CBYT4477.b1	GE289171
61933127	CBYT4477.g1	GE289172
61933128	CBYT4478.b1	GE289173
61933129	CBYT4478.g1	GE289174
61933130	CBYT4479.b1	GE289175
61933131	CBYT4479.g1	GE289176
61933132	CBYT448.b1	GE289177
61933133	CBYT448.g2	GE289178
61933134	CBYT4480.b1	GE289179
61933135	CBYT4480.g1	GE289180
61933136	CBYT4481.b1	GE289181
61933137	CBYT4481.g1	GE289182
61933138	CBYT4482.b1	GE289183
61933139	CBYT4482.g1	GE289184
61933140	CBYT4483.b1	GE289185
61933141	CBYT4483.g1	GE289186
61933142	CBYT4484.b1	GE289187
61933143	CBYT4484.g1	GE289188
61933144	CBYT4485.b1	GE289189
61933145	CBYT4485.g1	GE289190
61933146	CBYT4486.b1	GE289191
61933147	CBYT4486.g1	GE289192
61933148	CBYT4487.g1	GE289193
61933149	CBYT4489.b1	GE289194
61933150	CBYT4489.g1	GE289195
61933151	CBYT4490.b1	GE289196
61933152	CBYT4490.g1	GE289197
61933153	CBYT4491.b1	GE289198
61933154	CBYT4491.g1	GE289199
61933155	CBYT4492.b1	GE289200
61933156	CBYT4492.g1	GE289201
61933157	CBYT4493.b1	GE289202
61933158	CBYT4493.g1	GE289203
61933159	CBYT4494.b1	GE289204
61933160	CBYT4494.g1	GE289205
61933161	CBYT4495.b1	GE289206

61933162	CBYT4495.g1	GE289207
61933163	CBYT4496.b1	GE289208
61933164	CBYT4496.g1	GE289209
61933165	CBYT4498.b1	GE289210
61933166	CBYT4498.g1	GE289211
61933167	CBYT4499.g1	GE289212
61933168	CBYT450.b1	GE289213
61933169	CBYT450.g2	GE289214
61933170	CBYT4500.b1	GE289215
61933171	CBYT4500.g1	GE289216
61933172	CBYT4501.b1	GE289217
61933173	CBYT4501.g1	GE289218
61933174	CBYT4502.b1	GE289219
61933175	CBYT4502.g1	GE289220
61933176	CBYT4503.b1	GE289221
61933177	CBYT4503.g1	GE289222
61933178	CBYT4504.b1	GE289223
61933179	CBYT4504.g1	GE289224
61933180	CBYT4506.b1	GE289225
61933181	CBYT4506.g1	GE289226
61933182	CBYT4507.b1	GE289227
61933183	CBYT4507.g1	GE289228
61933184	CBYT4508.g1	GE289229
61933185	CBYT4509.b1	GE289230
61933186	CBYT4509.g1	GE289231
61933187	CBYT451.b1	GE289232
61933188	CBYT451.g2	GE289233
61933189	CBYT4510.g1	GE289234
61933190	CBYT4511.b1	GE289235
61933191	CBYT4511.g1	GE289236
61933192	CBYT4513.b1	GE289237
61933193	CBYT4513.g1	GE289238
61933194	CBYT4514.b1	GE289239
61933195	CBYT4514.g1	GE289240
61933196	CBYT4515.b1	GE289241
61933197	CBYT4515.g1	GE289242
61933198	CBYT4516.b1	GE289243
61933199	CBYT4516.g1	GE289244
61933200	CBYT4517.b1	GE289245
61933201	CBYT4517.g1	GE289246
61933202	CBYT4518.b1	GE289247
61933203	CBYT4519.b1	GE289248
61933204	CBYT4519.g1	GE289249
61933205	CBYT452.b1	GE289250
61933206	CBYT452.g2	GE289251
61933207	CBYT4520.b1	GE289252
61933208	CBYT4520.g1	GE289253
61933209	CBYT4521.b1	GE289254
61933210	CBYT4521.g1	GE289255
61933211	CBYT4522.b1	GE289256
61933212	CBYT4522.g1	GE289257
61933213	CBYT4523.b1	GE289258
61933214	CBYT4523.g1	GE289259
61933215	CBYT4524.b1	GE289260
61933216	CBYT4524.g1	GE289261
61933217	CBYT4525.b1	GE289262
61933218	CBYT4525.g1	GE289263
61933219	CBYT4526.b1	GE289264
61933220	CBYT4526.g1	GE289265
61933221	CBYT4527.b1	GE289266
61933222	CBYT4527.g1	GE289267
61933223	CBYT4528.b1	GE289268
61933224	CBYT4528.g1	GE289269

61933225	CBYT4529.b1	GE289270
61933226	CBYT4529.g1	GE289271
61933227	CBYT453.b1	GE289272
61933228	CBYT4530.b1	GE289273
61933229	CBYT4530.g1	GE289274
61933230	CBYT4531.b1	GE289275
61933231	CBYT4531.g1	GE289276
61933232	CBYT4532.b1	GE289277
61933233	CBYT4532.g1	GE289278
61933234	CBYT4533.b1	GE289279
61933235	CBYT4533.g1	GE289280
61933236	CBYT4534.b1	GE289281
61933237	CBYT4534.g1	GE289282
61933238	CBYT4535.b1	GE289283
61933239	CBYT4535.g1	GE289284
61933240	CBYT4536.b1	GE289285
61933241	CBYT4536.g1	GE289286
61933242	CBYT4537.b1	GE289287
61933243	CBYT4537.g1	GE289288
61933244	CBYT4538.b1	GE289289
61933245	CBYT4538.g1	GE289290
61933246	CBYT4539.b1	GE289291
61933247	CBYT4539.g1	GE289292
61933248	CBYT454.b1	GE289293
61933249	CBYT454.g2	GE289294
61933250	CBYT4540.b1	GE289295
61933251	CBYT4540.g1	GE289296
61933252	CBYT4542.b1	GE289297
61933253	CBYT4542.g1	GE289298
61933254	CBYT4543.b1	GE289299
61933255	CBYT4543.g1	GE289300
61933256	CBYT4544.b1	GE289301
61933257	CBYT4544.g1	GE289302
61933258	CBYT4545.b1	GE289303
61933259	CBYT4545.g1	GE289304
61933260	CBYT4546.b1	GE289305
61933261	CBYT4546.g1	GE289306
61933262	CBYT4547.b1	GE289307
61933263	CBYT4547.g1	GE289308
61933264	CBYT4548.b1	GE289309
61933265	CBYT4548.g1	GE289310
61933266	CBYT4549.b1	GE289311
61933267	CBYT4549.g1	GE289312
61933268	CBYT455.b1	GE289313
61933269	CBYT455.g2	GE289314
61933270	CBYT4550.b1	GE289315
61933271	CBYT4550.g1	GE289316
61933272	CBYT4551.b1	GE289317
61933273	CBYT4551.g1	GE289318
61933274	CBYT4552.b1	GE289319
61933275	CBYT4552.g1	GE289320
61933276	CBYT4553.b1	GE289321
61933277	CBYT4553.g1	GE289322
61933278	CBYT4554.b1	GE289323
61933279	CBYT4554.g1	GE289324
61933280	CBYT4555.b1	GE289325
61933281	CBYT4555.g1	GE289326
61933282	CBYT4556.b1	GE289327
61933283	CBYT4556.g1	GE289328
61933284	CBYT4557.b1	GE289329
61933285	CBYT4557.g1	GE289330
61933286	CBYT4558.b1	GE289331
61933287	CBYT4558.g1	GE289332

61933288	CBYT4559.b1	GE289333
61933289	CBYT4559.g1	GE289334
61933290	CBYT456.b1	GE289335
61933291	CBYT456.g2	GE289336
61933292	CBYT4560.b1	GE289337
61933293	CBYT4560.g1	GE289338
61933294	CBYT4561.b1	GE289339
61933295	CBYT4561.g1	GE289340
61933296	CBYT4562.b1	GE289341
61933297	CBYT4562.g1	GE289342
61933298	CBYT4563.b1	GE289343
61933299	CBYT4563.g1	GE289344
61933300	CBYT4564.b1	GE289345
61933301	CBYT4564.g1	GE289346
61933302	CBYT4565.b1	GE289347
61933303	CBYT4565.g1	GE289348
61933304	CBYT4566.b1	GE289349
61933305	CBYT4566.g1	GE289350
61933306	CBYT4567.b1	GE289351
61933307	CBYT4567.g1	GE289352
61933308	CBYT4569.b1	GE289353
61933309	CBYT4569.g1	GE289354
61933310	CBYT457.b1	GE289355
61933311	CBYT457.g2	GE289356
61933312	CBYT4570.b1	GE289357
61933313	CBYT4570.g1	GE289358
61933314	CBYT4571.b1	GE289359
61933315	CBYT4571.g1	GE289360
61933316	CBYT4572.b1	GE289361
61933317	CBYT4572.g1	GE289362
61933318	CBYT4573.b1	GE289363
61933319	CBYT4573.g1	GE289364
61933320	CBYT4574.b1	GE289365
61933321	CBYT4574.g1	GE289366
61933322	CBYT4575.b1	GE289367
61933323	CBYT4575.g1	GE289368
61933324	CBYT4576.b1	GE289369
61933325	CBYT4576.g1	GE289370
61933326	CBYT4577.b1	GE289371
61933327	CBYT4577.g1	GE289372
61933328	CBYT4578.b1	GE289373
61933329	CBYT4578.g1	GE289374
61933330	CBYT4579.b1	GE289375
61933331	CBYT4579.g1	GE289376
61933332	CBYT458.b1	GE289377
61933333	CBYT458.g2	GE289378
61933334	CBYT4580.b1	GE289379
61933335	CBYT4580.g1	GE289380
61933336	CBYT4581.b1	GE289381
61933337	CBYT4582.b1	GE289382
61933338	CBYT4582.g1	GE289383
61933339	CBYT4583.b1	GE289384
61933340	CBYT4583.g1	GE289385
61933341	CBYT4584.b1	GE289386
61933342	CBYT4584.g1	GE289387
61933343	CBYT4585.b1	GE289388
61933344	CBYT4585.g1	GE289389
61933345	CBYT4586.b1	GE289390
61933346	CBYT4586.g1	GE289391
61933347	CBYT4587.b1	GE289392
61933348	CBYT4587.g1	GE289393
61933349	CBYT4589.b1	GE289394
61933350	CBYT4589.g1	GE289395

61933351	CBYT459.g2	GE289396
61933352	CBYT4590.b1	GE289397
61933353	CBYT4590.g1	GE289398
61933354	CBYT4591.b1	GE289399
61933355	CBYT4591.g1	GE289400
61933356	CBYT4593.b1	GE289401
61933357	CBYT4593.g1	GE289402
61933358	CBYT4594.b1	GE289403
61933359	CBYT4594.g1	GE289404
61933360	CBYT4595.b1	GE289405
61933361	CBYT4595.g1	GE289406
61933362	CBYT4596.g1	GE289407
61933363	CBYT4597.b1	GE289408
61933364	CBYT4597.g1	GE289409
61933365	CBYT4598.b1	GE289410
61933366	CBYT4598.g1	GE289411
61933367	CBYT4599.b1	GE289412
61933368	CBYT4599.g1	GE289413
61933369	CBYT460.b1	GE289414
61933370	CBYT460.g2	GE289415
61933371	CBYT4600.b1	GE289416
61933372	CBYT4600.g1	GE289417
61933373	CBYT4601.b1	GE289418
61933374	CBYT4601.g1	GE289419
61933375	CBYT4602.b1	GE289420
61933376	CBYT4602.g1	GE289421
61933377	CBYT4603.b1	GE289422
61933378	CBYT4603.g1	GE289423
61933379	CBYT4604.b1	GE289424
61933380	CBYT4604.g1	GE289425
61933381	CBYT4605.b1	GE289426
61933382	CBYT4606.b1	GE289427
61933383	CBYT4607.b1	GE289428
61933384	CBYT4607.g1	GE289429
61933385	CBYT4608.b1	GE289430
61933386	CBYT4608.g1	GE289431
61933387	CBYT4609.b1	GE289432
61933388	CBYT4609.g1	GE289433
61933389	CBYT461.b1	GE289434
61933390	CBYT461.g2	GE289435
61933391	CBYT4610.b1	GE289436
61933392	CBYT4610.g1	GE289437
61933393	CBYT4611.b1	GE289438
61933394	CBYT4611.g1	GE289439
61933395	CBYT4612.b1	GE289440
61933396	CBYT4612.g1	GE289441
61933397	CBYT4613.b1	GE289442
61933398	CBYT4613.g1	GE289443
61933399	CBYT4614.b1	GE289444
61933400	CBYT4614.g1	GE289445
61933401	CBYT4615.b1	GE289446
61933402	CBYT4615.g1	GE289447
61933403	CBYT4617.g1	GE289448
61933404	CBYT4618.b1	GE289449
61933405	CBYT4618.g1	GE289450
61933406	CBYT4619.b1	GE289451
61933407	CBYT4619.g1	GE289452
61933408	CBYT462.b1	GE289453
61933409	CBYT462.g2	GE289454
61933410	CBYT4620.b1	GE289455
61933411	CBYT4621.b1	GE289456
61933412	CBYT4621.g1	GE289457
61933413	CBYT4622.b1	GE289458

61933414	CBYT4622.g1	GE289459
61933415	CBYT4623.b1	GE289460
61933416	CBYT4623.g1	GE289461
61933417	CBYT4624.b1	GE289462
61933418	CBYT4624.g1	GE289463
61933419	CBYT4625.b1	GE289464
61933420	CBYT4625.g1	GE289465
61933421	CBYT4626.b1	GE289466
61933422	CBYT4626.g1	GE289467
61933423	CBYT4628.b1	GE289468
61933424	CBYT4628.g1	GE289469
61933425	CBYT4629.b1	GE289470
61933426	CBYT4629.g1	GE289471
61933427	CBYT463.b1	GE289472
61933428	CBYT463.g2	GE289473
61933429	CBYT4630.b1	GE289474
61933430	CBYT4630.g1	GE289475
61933431	CBYT4631.b1	GE289476
61933432	CBYT4631.g1	GE289477
61933433	CBYT4632.b1	GE289478
61933434	CBYT4632.g1	GE289479
61933435	CBYT4633.b1	GE289480
61933436	CBYT4633.g1	GE289481
61933437	CBYT4634.b1	GE289482
61933438	CBYT4634.g1	GE289483
61933439	CBYT4635.b1	GE289484
61933440	CBYT4635.g1	GE289485
61933441	CBYT4636.b1	GE289486
61933442	CBYT4636.g1	GE289487
61933443	CBYT4637.b1	GE289488
61933444	CBYT4637.g1	GE289489
61933445	CBYT4638.b1	GE289490
61933446	CBYT4638.g1	GE289491
61933447	CBYT4639.b1	GE289492
61933448	CBYT4639.g1	GE289493
61933449	CBYT464.b1	GE289494
61933450	CBYT464.g2	GE289495
61933451	CBYT4640.b1	GE289496
61933452	CBYT4640.g1	GE289497
61933453	CBYT4641.b1	GE289498
61933454	CBYT4641.g1	GE289499
61933455	CBYT4642.b1	GE289500
61933456	CBYT4642.g1	GE289501
61933457	CBYT4643.b1	GE289502
61933458	CBYT4643.g1	GE289503
61933459	CBYT4644.b1	GE289504
61933460	CBYT4644.g1	GE289505
61933461	CBYT4645.b1	GE289506
61933462	CBYT4645.g1	GE289507
61933463	CBYT4646.b1	GE289508
61933464	CBYT4646.g1	GE289509
61933465	CBYT4647.b1	GE289510
61933466	CBYT4647.g1	GE289511
61933467	CBYT4648.b1	GE289512
61933468	CBYT4649.b1	GE289513
61933469	CBYT465.b1	GE289514
61933470	CBYT465.g2	GE289515
61933471	CBYT4651.b1	GE289516
61933472	CBYT4651.g1	GE289517
61933473	CBYT4652.b1	GE289518
61933474	CBYT4652.g1	GE289519
61933475	CBYT4653.g1	GE289520
61933476	CBYT4654.g1	GE289521

61933477	CBYT4655.b1	GE289522
61933478	CBYT4656.b1	GE289523
61933479	CBYT4656.g1	GE289524
61933480	CBYT4657.b1	GE289525
61933481	CBYT4657.g1	GE289526
61933482	CBYT4658.b1	GE289527
61933483	CBYT4658.g1	GE289528
61933484	CBYT4659.b1	GE289529
61933485	CBYT4659.g1	GE289530
61933486	CBYT466.b1	GE289531
61933487	CBYT4660.b1	GE289532
61933488	CBYT4660.g1	GE289533
61933489	CBYT4661.b1	GE289534
61933490	CBYT4661.g1	GE289535
61933491	CBYT4663.b1	GE289536
61933492	CBYT4664.b1	GE289537
61933493	CBYT4664.g1	GE289538
61933494	CBYT4665.b1	GE289539
61933495	CBYT4665.g1	GE289540
61933496	CBYT4666.b1	GE289541
61933497	CBYT4666.g1	GE289542
61933498	CBYT4667.b1	GE289543
61933499	CBYT4667.g1	GE289544
61933500	CBYT4668.b1	GE289545
61933501	CBYT4668.g1	GE289546
61933502	CBYT4669.b1	GE289547
61933503	CBYT4669.g1	GE289548
61933504	CBYT4670.b1	GE289549
61933505	CBYT4670.g1	GE289550
61933506	CBYT4671.b1	GE289551
61933507	CBYT4671.g1	GE289552
61933508	CBYT4672.b1	GE289553
61933509	CBYT4672.g1	GE289554
61933510	CBYT4673.b1	GE289555
61933511	CBYT4674.b1	GE289556
61933512	CBYT4674.g1	GE289557
61933513	CBYT4675.b1	GE289558
61933514	CBYT4675.g1	GE289559
61933515	CBYT4676.b1	GE289560
61933516	CBYT4676.g1	GE289561
61933517	CBYT4677.b1	GE289562
61933518	CBYT4677.g1	GE289563
61933519	CBYT4678.b1	GE289564
61933520	CBYT4678.g1	GE289565
61933521	CBYT468.b1	GE289566
61933522	CBYT468.g2	GE289567
61933523	CBYT4680.b1	GE289568
61933524	CBYT4680.g1	GE289569
61933525	CBYT4681.b1	GE289570
61933526	CBYT4681.g1	GE289571
61933527	CBYT4682.b1	GE289572
61933528	CBYT4683.g1	GE289573
61933529	CBYT4684.b1	GE289574
61933530	CBYT4684.g1	GE289575
61933531	CBYT4685.b1	GE289576
61933532	CBYT4685.g1	GE289577
61933533	CBYT4686.b1	GE289578
61933534	CBYT4686.g1	GE289579
61933535	CBYT4687.b1	GE289580
61933536	CBYT4687.g1	GE289581
61933537	CBYT4688.b1	GE289582
61933538	CBYT4688.g1	GE289583
61933539	CBYT4689.b1	GE289584

61933540	CBYT4689.g1	GE289585
61933541	CBYT469.b1	GE289586
61933542	CBYT469.g2	GE289587
61933543	CBYT4691.b1	GE289588
61933544	CBYT4691.g1	GE289589
61933545	CBYT4693.b1	GE289590
61933546	CBYT4693.g1	GE289591
61933547	CBYT4694.b1	GE289592
61933548	CBYT4694.g1	GE289593
61933549	CBYT4695.g1	GE289594
61933550	CBYT4696.b1	GE289595
61933551	CBYT4696.g1	GE289596
61933552	CBYT4697.b1	GE289597
61933553	CBYT4697.g1	GE289598
61933554	CBYT4698.b1	GE289599
61933555	CBYT4698.g1	GE289600
61933556	CBYT4699.b1	GE289601
61933557	CBYT4699.g1	GE289602
61933558	CBYT470.b1	GE289603
61933559	CBYT4700.b1	GE289604
61933560	CBYT4700.g1	GE289605
61933561	CBYT4701.b1	GE289606
61933562	CBYT4701.g1	GE289607
61933563	CBYT4702.b1	GE289608
61933564	CBYT4702.g1	GE289609
61933565	CBYT4703.b1	GE289610
61933566	CBYT4703.g1	GE289611
61933567	CBYT4704.b1	GE289612
61933568	CBYT4704.g1	GE289613
61933569	CBYT4705.b1	GE289614
61933570	CBYT4705.g1	GE289615
61933571	CBYT4706.b1	GE289616
61933572	CBYT4706.g1	GE289617
61933573	CBYT4707.b1	GE289618
61933574	CBYT4707.g1	GE289619
61933575	CBYT4708.b1	GE289620
61933576	CBYT4708.g1	GE289621
61933577	CBYT4709.b1	GE289622
61933578	CBYT4709.g1	GE289623
61933579	CBYT471.b1	GE289624
61933580	CBYT471.g2	GE289625
61933581	CBYT4710.b1	GE289626
61933582	CBYT4710.g1	GE289627
61933583	CBYT4711.b1	GE289628
61933584	CBYT4711.g1	GE289629
61933585	CBYT4712.b1	GE289630
61933586	CBYT4712.g1	GE289631
61933587	CBYT4713.b1	GE289632
61933588	CBYT4713.g1	GE289633
61933589	CBYT4714.b1	GE289634
61933590	CBYT4714.g1	GE289635
61933591	CBYT4716.b1	GE289636
61933592	CBYT4716.g1	GE289637
61933593	CBYT4717.b1	GE289638
61933594	CBYT4718.b1	GE289639
61933595	CBYT4718.g1	GE289640
61933596	CBYT4719.g1	GE289641
61933597	CBYT472.b1	GE289642
61933598	CBYT472.g2	GE289643
61933599	CBYT4720.b1	GE289644
61933600	CBYT4722.b1	GE289645
61933601	CBYT4722.g1	GE289646
61933602	CBYT4723.b1	GE289647

61933603	CBYT4723.g1	GE289648
61933604	CBYT4724.b1	GE289649
61933605	CBYT4724.g1	GE289650
61933606	CBYT4726.b1	GE289651
61933607	CBYT4726.g1	GE289652
61933608	CBYT4727.b1	GE289653
61933609	CBYT4727.g1	GE289654
61933610	CBYT4728.b1	GE289655
61933611	CBYT4728.g1	GE289656
61933612	CBYT4729.g1	GE289657
61933613	CBYT473.b1	GE289658
61933614	CBYT473.g2	GE289659
61933615	CBYT4730.b1	GE289660
61933616	CBYT4730.g1	GE289661
61933617	CBYT4731.b1	GE289662
61933618	CBYT4731.g1	GE289663
61933619	CBYT4732.b1	GE289664
61933620	CBYT4732.g1	GE289665
61933621	CBYT4733.b1	GE289666
61933622	CBYT4733.g1	GE289667
61933623	CBYT4734.g1	GE289668
61933624	CBYT4735.g1	GE289669
61933625	CBYT4737.b1	GE289670
61933626	CBYT4738.b1	GE289671
61933627	CBYT4738.g1	GE289672
61933628	CBYT4739.b1	GE289673
61933629	CBYT4739.g1	GE289674
61933630	CBYT474.b1	GE289675
61933631	CBYT474.g2	GE289676
61933632	CBYT4740.g1	GE289677
61933633	CBYT4741.b1	GE289678
61933634	CBYT4741.g1	GE289679
61933635	CBYT4742.b1	GE289680
61933636	CBYT4742.g1	GE289681
61933637	CBYT4743.b1	GE289682
61933638	CBYT4743.g1	GE289683
61933639	CBYT4744.b1	GE289684
61933640	CBYT4744.g1	GE289685
61933641	CBYT4745.g1	GE289686
61933642	CBYT4746.b1	GE289687
61933643	CBYT4746.g1	GE289688
61933644	CBYT4747.b1	GE289689
61933645	CBYT4747.g1	GE289690
61933646	CBYT4748.g1	GE289691
61933647	CBYT4749.b1	GE289692
61933648	CBYT4749.g1	GE289693
61933649	CBYT475.b1	GE289694
61933650	CBYT475.g2	GE289695
61933651	CBYT4750.b1	GE289696
61933652	CBYT4750.g1	GE289697
61933653	CBYT4751.b1	GE289698
61933654	CBYT4751.g1	GE289699
61933655	CBYT4752.b1	GE289700
61933656	CBYT4752.g1	GE289701
61933657	CBYT4753.b1	GE289702
61933658	CBYT4753.g1	GE289703
61933659	CBYT4754.b1	GE289704
61933660	CBYT4754.g1	GE289705
61933661	CBYT4757.b1	GE289706
61933662	CBYT4757.g1	GE289707
61933663	CBYT4758.b1	GE289708
61933664	CBYT4758.g1	GE289709
61933665	CBYT4759.b1	GE289710

61933666	CBYT4759.g1	GE289711
61933667	CBYT476.b1	GE289712
61933668	CBYT476.g2	GE289713
61933669	CBYT4760.b1	GE289714
61933670	CBYT4760.g1	GE289715
61933671	CBYT4761.b1	GE289716
61933672	CBYT4761.g1	GE289717
61933673	CBYT4762.g1	GE289718
61933674	CBYT4763.b1	GE289719
61933675	CBYT4763.g1	GE289720
61933676	CBYT4765.b1	GE289721
61933677	CBYT4765.g1	GE289722
61933678	CBYT4766.b1	GE289723
61933679	CBYT4766.g1	GE289724
61933680	CBYT4767.b1	GE289725
61933681	CBYT4767.g1	GE289726
61933682	CBYT4768.b1	GE289727
61933683	CBYT4768.g1	GE289728
61933684	CBYT4769.b1	GE289729
61933685	CBYT477.b1	GE289730
61933686	CBYT477.g2	GE289731
61933687	CBYT4770.b1	GE289732
61933688	CBYT4770.g1	GE289733
61933689	CBYT4771.b1	GE289734
61933690	CBYT4772.b1	GE289735
61933691	CBYT4772.g1	GE289736
61933692	CBYT4773.b1	GE289737
61933693	CBYT4773.g1	GE289738
61933694	CBYT4774.b1	GE289739
61933695	CBYT4774.g1	GE289740
61933696	CBYT4775.b1	GE289741
61933697	CBYT4775.g1	GE289742
61933698	CBYT4776.b1	GE289743
61933699	CBYT4776.g1	GE289744
61933700	CBYT4777.b1	GE289745
61933701	CBYT4777.g1	GE289746
61933702	CBYT4778.b1	GE289747
61933703	CBYT4778.g1	GE289748
61933704	CBYT4779.b1	GE289749
61933705	CBYT4779.g1	GE289750
61933706	CBYT478.b1	GE289751
61933707	CBYT4782.b1	GE289752
61933708	CBYT4782.g1	GE289753
61933709	CBYT4783.b1	GE289754
61933710	CBYT4783.g1	GE289755
61933711	CBYT4784.b1	GE289756
61933712	CBYT4784.g1	GE289757
61933713	CBYT4785.b1	GE289758
61933714	CBYT4785.g1	GE289759
61933715	CBYT4786.b1	GE289760
61933716	CBYT4786.g1	GE289761
61933717	CBYT4787.b1	GE289762
61933718	CBYT4788.b1	GE289763
61933719	CBYT4788.g1	GE289764
61933720	CBYT4789.b1	GE289765
61933721	CBYT4789.g1	GE289766
61933722	CBYT479.b1	GE289767
61933723	CBYT479.g2	GE289768
61933724	CBYT4790.b1	GE289769
61933725	CBYT4790.g1	GE289770
61933726	CBYT4791.b1	GE289771
61933727	CBYT4791.g1	GE289772
61933728	CBYT4792.b1	GE289773

61933729	CBYT4792.g1	GE289774
61933730	CBYT4793.b1	GE289775
61933731	CBYT4794.b1	GE289776
61933732	CBYT4794.g1	GE289777
61933733	CBYT4795.b1	GE289778
61933734	CBYT4795.g1	GE289779
61933735	CBYT4796.b1	GE289780
61933736	CBYT4796.g1	GE289781
61933737	CBYT4797.b1	GE289782
61933738	CBYT4797.g1	GE289783
61933739	CBYT4798.b1	GE289784
61933740	CBYT4798.g1	GE289785
61933741	CBYT4799.b1	GE289786
61933742	CBYT4799.g1	GE289787
61933743	CBYT480.b1	GE289788
61933744	CBYT480.g2	GE289789
61933745	CBYT4800.b1	GE289790
61933746	CBYT4800.g1	GE289791
61933747	CBYT4801.b1	GE289792
61933748	CBYT4802.g1	GE289793
61933749	CBYT4803.b1	GE289794
61933750	CBYT4803.g1	GE289795
61933751	CBYT4804.b1	GE289796
61933752	CBYT4804.g1	GE289797
61933753	CBYT4805.b1	GE289798
61933754	CBYT4805.g1	GE289799
61933755	CBYT4806.b1	GE289800
61933756	CBYT4806.g1	GE289801
61933757	CBYT4807.b1	GE289802
61933758	CBYT4807.g1	GE289803
61933759	CBYT4808.b1	GE289804
61933760	CBYT4808.g1	GE289805
61933761	CBYT4809.b1	GE289806
61933762	CBYT4809.g1	GE289807
61933763	CBYT481.b1	GE289808
61933764	CBYT481.g2	GE289809
61933765	CBYT4810.b1	GE289810
61933766	CBYT4811.b1	GE289811
61933767	CBYT4811.g1	GE289812
61933768	CBYT4812.b1	GE289813
61933769	CBYT4812.g1	GE289814
61933770	CBYT4813.b1	GE289815
61933771	CBYT4813.g1	GE289816
61933772	CBYT4814.b1	GE289817
61933773	CBYT4814.g1	GE289818
61933774	CBYT4815.b1	GE289819
61933775	CBYT4815.g1	GE289820
61933776	CBYT4816.b1	GE289821
61933777	CBYT4816.g1	GE289822
61933778	CBYT4817.b1	GE289823
61933779	CBYT4817.g1	GE289824
61933780	CBYT4819.b1	GE289825
61933781	CBYT4819.g1	GE289826
61933782	CBYT482.b1	GE289827
61933783	CBYT482.g2	GE289828
61933784	CBYT4820.b1	GE289829
61933785	CBYT4820.g1	GE289830
61933786	CBYT4821.b1	GE289831
61933787	CBYT4821.g1	GE289832
61933788	CBYT4822.b1	GE289833
61933789	CBYT4822.g1	GE289834
61933790	CBYT4823.b1	GE289835
61933791	CBYT4823.g1	GE289836

61933792	CBYT4824.b1	GE289837
61933793	CBYT4824.g1	GE289838
61933794	CBYT4825.b1	GE289839
61933795	CBYT4825.g1	GE289840
61933796	CBYT4828.b1	GE289841
61933797	CBYT4828.g1	GE289842
61933798	CBYT4829.b1	GE289843
61933799	CBYT4829.g1	GE289844
61933800	CBYT483.b1	GE289845
61933801	CBYT4830.b1	GE289846
61933802	CBYT4830.g1	GE289847
61933803	CBYT4831.b1	GE289848
61933804	CBYT4831.g1	GE289849
61933805	CBYT4832.b1	GE289850
61933806	CBYT4832.g1	GE289851
61933807	CBYT4833.b1	GE289852
61933808	CBYT4833.g1	GE289853
61933809	CBYT4834.b1	GE289854
61933810	CBYT4834.g1	GE289855
61933811	CBYT4835.b1	GE289856
61933812	CBYT4835.g1	GE289857
61933813	CBYT4836.b1	GE289858
61933814	CBYT4836.g1	GE289859
61933815	CBYT4837.b1	GE289860
61933816	CBYT4837.g1	GE289861
61933817	CBYT4838.g1	GE289862
61933818	CBYT4839.b1	GE289863
61933819	CBYT4839.g1	GE289864
61933820	CBYT484.b1	GE289865
61933821	CBYT484.g2	GE289866
61933822	CBYT4840.b1	GE289867
61933823	CBYT4840.g1	GE289868
61933824	CBYT4841.b1	GE289869
61933825	CBYT4841.g1	GE289870
61933826	CBYT4842.b1	GE289871
61933827	CBYT4843.b1	GE289872
61933828	CBYT4843.g1	GE289873
61933829	CBYT4844.b1	GE289874
61933830	CBYT4844.g1	GE289875
61933831	CBYT4845.b1	GE289876
61933832	CBYT4845.g1	GE289877
61933833	CBYT4846.b1	GE289878
61933834	CBYT4847.b1	GE289879
61933835	CBYT4847.g1	GE289880
61933836	CBYT4848.b1	GE289881
61933837	CBYT4848.g1	GE289882
61933838	CBYT4849.b1	GE289883
61933839	CBYT4849.g1	GE289884
61933840	CBYT485.b1	GE289885
61933841	CBYT485.g2	GE289886
61933842	CBYT4850.b1	GE289887
61933843	CBYT4850.g1	GE289888
61933844	CBYT4851.b1	GE289889
61933845	CBYT4851.g1	GE289890
61933846	CBYT4852.b1	GE289891
61933847	CBYT4852.g1	GE289892
61933848	CBYT4853.b1	GE289893
61933849	CBYT4853.g1	GE289894
61933850	CBYT4854.b1	GE289895
61933851	CBYT4854.g1	GE289896
61933852	CBYT4855.b1	GE289897
61933853	CBYT4855.g1	GE289898
61933854	CBYT4856.b1	GE289899

61933855	CBYT4856.g1	GE289900
61933856	CBYT4857.g1	GE289901
61933857	CBYT4858.g1	GE289902
61933858	CBYT4859.b1	GE289903
61933859	CBYT4859.g1	GE289904
61933860	CBYT486.b1	GE289905
61933861	CBYT486.g2	GE289906
61933862	CBYT4860.b1	GE289907
61933863	CBYT4860.g1	GE289908
61933864	CBYT4861.b1	GE289909
61933865	CBYT4861.g1	GE289910
61933866	CBYT4862.b1	GE289911
61933867	CBYT4862.g1	GE289912
61933868	CBYT4863.b1	GE289913
61933869	CBYT4863.g1	GE289914
61933870	CBYT4864.b1	GE289915
61933871	CBYT4864.g1	GE289916
61933872	CBYT4865.b1	GE289917
61933873	CBYT4865.g1	GE289918
61933874	CBYT4866.b1	GE289919
61933875	CBYT4866.g1	GE289920
61933876	CBYT4867.b1	GE289921
61933877	CBYT4867.g1	GE289922
61933878	CBYT4868.b1	GE289923
61933879	CBYT4868.g1	GE289924
61933880	CBYT4869.b1	GE289925
61933881	CBYT4869.g1	GE289926
61933882	CBYT4870.b1	GE289927
61933883	CBYT4870.g1	GE289928
61933884	CBYT4871.b1	GE289929
61933885	CBYT4871.g1	GE289930
61933886	CBYT4872.b1	GE289931
61933887	CBYT4872.g1	GE289932
61933888	CBYT4873.b1	GE289933
61933889	CBYT4873.g1	GE289934
61933890	CBYT4874.b1	GE289935
61933891	CBYT4875.b1	GE289936
61933892	CBYT4875.g1	GE289937
61933893	CBYT4876.b1	GE289938
61933894	CBYT4876.g1	GE289939
61933895	CBYT4877.g1	GE289940
61933896	CBYT4878.b1	GE289941
61933897	CBYT4878.g1	GE289942
61933898	CBYT4879.b1	GE289943
61933899	CBYT4879.g1	GE289944
61933900	CBYT488.b1	GE289945
61933901	CBYT488.g2	GE289946
61933902	CBYT4880.b1	GE289947
61933903	CBYT4880.g1	GE289948
61933904	CBYT4881.b1	GE289949
61933905	CBYT4881.g1	GE289950
61933906	CBYT4882.b1	GE289951
61933907	CBYT4882.g1	GE289952
61933908	CBYT4884.b1	GE289953
61933909	CBYT4884.g1	GE289954
61933910	CBYT4885.g1	GE289955
61933911	CBYT4886.b1	GE289956
61933912	CBYT4886.g1	GE289957
61933913	CBYT4887.b1	GE289958
61933914	CBYT4887.g1	GE289959
61933915	CBYT4888.b1	GE289960
61933916	CBYT4888.g1	GE289961
61933917	CBYT4889.b1	GE289962

61933918	CBYT489.b1	GE289963
61933919	CBYT489.g2	GE289964
61933920	CBYT4890.b1	GE289965
61933921	CBYT4890.g1	GE289966
61933922	CBYT4891.b1	GE289967
61933923	CBYT4891.g1	GE289968
61933924	CBYT4892.b1	GE289969
61933925	CBYT4892.g1	GE289970
61933926	CBYT4893.b1	GE289971
61933927	CBYT4893.g1	GE289972
61933928	CBYT4894.b1	GE289973
61933929	CBYT4894.g1	GE289974
61933930	CBYT4895.b1	GE289975
61933931	CBYT4895.g1	GE289976
61933932	CBYT4896.b1	GE289977
61933933	CBYT4896.g1	GE289978
61933934	CBYT4897.b1	GE289979
61933935	CBYT4897.g1	GE289980
61933936	CBYT4898.b1	GE289981
61933937	CBYT4898.g1	GE289982
61933938	CBYT4899.b1	GE289983
61933939	CBYT4899.g1	GE289984
61933940	CBYT490.b1	GE289985
61933941	CBYT490.g2	GE289986
61933942	CBYT4900.b1	GE289987
61933943	CBYT4900.g1	GE289988
61933944	CBYT4901.b1	GE289989
61933945	CBYT4901.g1	GE289990
61933946	CBYT4902.b1	GE289991
61933947	CBYT4902.g1	GE289992
61933948	CBYT4903.b1	GE289993
61933949	CBYT4903.g1	GE289994
61933950	CBYT4905.b1	GE289995
61933951	CBYT4905.g1	GE289996
61933952	CBYT4906.g1	GE289997
61933953	CBYT4907.b1	GE289998
61933954	CBYT4907.g1	GE289999
61933955	CBYT4908.b1	GE290000
61933956	CBYT4909.b1	GE290001
61933957	CBYT4909.g1	GE290002
61933958	CBYT491.b1	GE290003
61933959	CBYT491.g2	GE290004
61933960	CBYT4910.b1	GE290005
61933961	CBYT4910.g1	GE290006
61933962	CBYT4911.b1	GE290007
61933963	CBYT4911.g1	GE290008
61933964	CBYT4912.b1	GE290009
61933965	CBYT4912.g1	GE290010
61933966	CBYT4913.b1	GE290011
61933967	CBYT4913.g1	GE290012
61933968	CBYT4914.b1	GE290013
61933969	CBYT4915.b1	GE290014
61933970	CBYT4915.g1	GE290015
61933971	CBYT4916.b1	GE290016
61933972	CBYT4916.g1	GE290017
61933973	CBYT4917.b1	GE290018
61933974	CBYT4917.g1	GE290019
61933975	CBYT4918.b1	GE290020
61933976	CBYT4918.g1	GE290021
61933977	CBYT4919.b1	GE290022
61933978	CBYT4919.g1	GE290023
61933979	CBYT492.b1	GE290024
61933980	CBYT492.g2	GE290025

61933981	CBYT4920.g1	GE290026
61933982	CBYT4921.b1	GE290027
61933983	CBYT4922.b1	GE290028
61933984	CBYT4922.g1	GE290029
61933985	CBYT4923.b1	GE290030
61933986	CBYT4923.g1	GE290031
61933987	CBYT4924.b1	GE290032
61933988	CBYT4924.g1	GE290033
61933989	CBYT4925.b1	GE290034
61933990	CBYT4925.g1	GE290035
61933991	CBYT4926.b1	GE290036
61933992	CBYT4926.g1	GE290037
61933993	CBYT4927.b1	GE290038
61933994	CBYT4927.g1	GE290039
61933995	CBYT4928.b1	GE290040
61933996	CBYT4928.g1	GE290041
61933997	CBYT4929.b1	GE290042
61933998	CBYT4929.g1	GE290043
61933999	CBYT493.b1	GE290044
61934000	CBYT493.g2	GE290045
61934001	CBYT4931.b1	GE290046
61934002	CBYT4932.b1	GE290047
61934003	CBYT4932.g1	GE290048
61934004	CBYT4933.b1	GE290049
61934005	CBYT4933.g1	GE290050
61934006	CBYT4934.b1	GE290051
61934007	CBYT4934.g1	GE290052
61934008	CBYT4935.g1	GE290053
61934009	CBYT4936.b1	GE290054
61934010	CBYT4936.g1	GE290055
61934011	CBYT4937.b1	GE290056
61934012	CBYT4937.g1	GE290057
61934013	CBYT4938.b1	GE290058
61934014	CBYT4938.g1	GE290059
61934015	CBYT4939.b1	GE290060
61934016	CBYT4939.g1	GE290061
61934017	CBYT494.b1	GE290062
61934018	CBYT494.g2	GE290063
61934019	CBYT4940.b1	GE290064
61934020	CBYT4941.b1	GE290065
61934021	CBYT4941.g1	GE290066
61934022	CBYT4942.b1	GE290067
61934023	CBYT4942.g1	GE290068
61934024	CBYT4943.b1	GE290069
61934025	CBYT4944.b1	GE290070
61934026	CBYT4944.g1	GE290071
61934027	CBYT4945.b1	GE290072
61934028	CBYT4945.g1	GE290073
61934029	CBYT4946.b1	GE290074
61934030	CBYT4947.b1	GE290075
61934031	CBYT4947.g1	GE290076
61934032	CBYT4948.b1	GE290077
61934033	CBYT4948.g1	GE290078
61934034	CBYT4949.b1	GE290079
61934035	CBYT4949.g1	GE290080
61934036	CBYT495.b1	GE290081
61934037	CBYT495.g2	GE290082
61934038	CBYT4950.g1	GE290083
61934039	CBYT4951.b1	GE290084
61934040	CBYT4952.b1	GE290085
61934041	CBYT4952.g1	GE290086
61934042	CBYT4953.b1	GE290087
61934043	CBYT4953.g1	GE290088

61934044	CBYT4954.b1	GE290089
61934045	CBYT4954.g1	GE290090
61934046	CBYT4955.b1	GE290091
61934047	CBYT4956.b1	GE290092
61934048	CBYT4957.b1	GE290093
61934049	CBYT4958.g1	GE290094
61934050	CBYT4959.b1	GE290095
61934051	CBYT4959.g1	GE290096
61934052	CBYT496.b1	GE290097
61934053	CBYT4960.b1	GE290098
61934054	CBYT4960.g1	GE290099
61934055	CBYT4961.b1	GE290100
61934056	CBYT4962.b1	GE290101
61934057	CBYT4962.g1	GE290102
61934058	CBYT4963.b1	GE290103
61934059	CBYT4963.g1	GE290104
61934060	CBYT4964.b1	GE290105
61934061	CBYT4964.g1	GE290106
61934062	CBYT4965.b1	GE290107
61934063	CBYT4965.g1	GE290108
61934064	CBYT4966.b1	GE290109
61934065	CBYT4967.b1	GE290110
61934066	CBYT4967.g1	GE290111
61934067	CBYT4968.b1	GE290112
61934068	CBYT4968.g1	GE290113
61934069	CBYT4969.b1	GE290114
61934070	CBYT4969.g1	GE290115
61934071	CBYT497.b1	GE290116
61934072	CBYT497.g2	GE290117
61934073	CBYT4970.b1	GE290118
61934074	CBYT4970.g1	GE290119
61934075	CBYT4971.b1	GE290120
61934076	CBYT4972.b1	GE290121
61934077	CBYT4972.g1	GE290122
61934078	CBYT4973.b1	GE290123
61934079	CBYT4973.g1	GE290124
61934080	CBYT4974.b1	GE290125
61934081	CBYT4974.g1	GE290126
61934082	CBYT4975.b1	GE290127
61934083	CBYT4975.g1	GE290128
61934084	CBYT4976.b1	GE290129
61934085	CBYT4976.g1	GE290130
61934086	CBYT4977.b1	GE290131
61934087	CBYT4977.g1	GE290132
61934088	CBYT4978.b1	GE290133
61934089	CBYT4979.b1	GE290134
61934090	CBYT4979.g1	GE290135
61934091	CBYT498.b1	GE290136
61934092	CBYT498.g2	GE290137
61934093	CBYT4980.b1	GE290138
61934094	CBYT4980.g1	GE290139
61934095	CBYT4981.b1	GE290140
61934096	CBYT4981.g1	GE290141
61934097	CBYT4982.b1	GE290142
61934098	CBYT4982.g1	GE290143
61934099	CBYT4984.b1	GE290144
61934100	CBYT4984.g1	GE290145
61934101	CBYT4985.b1	GE290146
61934102	CBYT4986.b1	GE290147
61934103	CBYT4986.g1	GE290148
61934104	CBYT4988.b1	GE290149
61934105	CBYT4988.g1	GE290150
61934106	CBYT4989.b1	GE290151

61934107	CBYT4989.g1	GE290152
61934108	CBYT499.b1	GE290153
61934109	CBYT499.g2	GE290154
61934110	CBYT4990.b1	GE290155
61934111	CBYT4990.g1	GE290156
61934112	CBYT4991.b1	GE290157
61934113	CBYT4991.g1	GE290158
61934114	CBYT4992.g1	GE290159
61934115	CBYT500.b1	GE290160
61934116	CBYT500.g2	GE290161
61934117	CBYT502.b1	GE290162
61934118	CBYT502.g2	GE290163
61934119	CBYT503.b1	GE290164
61934120	CBYT503.g2	GE290165
61934121	CBYT504.b1	GE290166
61934122	CBYT504.g2	GE290167
61934123	CBYT505.b1	GE290168
61934124	CBYT505.g2	GE290169
61934125	CBYT506.b1	GE290170
61934126	CBYT506.g2	GE290171
61934127	CBYT507.b1	GE290172
61934128	CBYT507.g2	GE290173
61934129	CBYT508.b1	GE290174
61934130	CBYT508.g2	GE290175
61934131	CBYT509.b1	GE290176
61934132	CBYT509.g2	GE290177
61934133	CBYT511.b1	GE290178
61934134	CBYT511.g2	GE290179
61934135	CBYT512.b1	GE290180
61934136	CBYT512.g2	GE290181
61934137	CBYT513.b1	GE290182
61934138	CBYT513.g2	GE290183
61934139	CBYT515.b1	GE290184
61934140	CBYT515.g2	GE290185
61934141	CBYT516.b1	GE290186
61934142	CBYT516.g2	GE290187
61934143	CBYT517.b1	GE290188
61934144	CBYT517.g2	GE290189
61934145	CBYT518.b1	GE290190
61934146	CBYT518.g2	GE290191
61934147	CBYT519.b1	GE290192
61934148	CBYT519.g2	GE290193
61934149	CBYT521.b1	GE290194
61934150	CBYT521.g2	GE290195
61934151	CBYT522.b1	GE290196
61934152	CBYT522.g2	GE290197
61934153	CBYT524.b1	GE290198
61934154	CBYT524.g2	GE290199
61934155	CBYT525.b1	GE290200
61934156	CBYT525.g2	GE290201
61934157	CBYT526.b1	GE290202
61934158	CBYT526.g2	GE290203
61934159	CBYT527.b1	GE290204
61934160	CBYT527.g2	GE290205
61934161	CBYT528.b1	GE290206
61934162	CBYT528.g2	GE290207
61934163	CBYT529.b1	GE290208
61934164	CBYT530.b1	GE290209
61934165	CBYT530.g2	GE290210
61934166	CBYT531.b1	GE290211
61934167	CBYT531.g2	GE290212
61934168	CBYT532.b1	GE290213
61934169	CBYT533.b1	GE290214

61934170	CBYT533.g2	GE290215
61934171	CBYT534.b1	GE290216
61934172	CBYT534.g2	GE290217
61934173	CBYT535.b1	GE290218
61934174	CBYT535.g2	GE290219
61934175	CBYT536.b1	GE290220
61934176	CBYT536.g2	GE290221
61934177	CBYT537.b1	GE290222
61934178	CBYT537.g2	GE290223
61934179	CBYT539.b1	GE290224
61934180	CBYT539.g2	GE290225
61934181	CBYT540.b1	GE290226
61934182	CBYT540.g2	GE290227
61934183	CBYT541.b1	GE290228
61934184	CBYT541.g2	GE290229
61934185	CBYT542.b1	GE290230
61934186	CBYT542.g2	GE290231
61934187	CBYT543.b1	GE290232
61934188	CBYT543.g2	GE290233
61934189	CBYT544.b1	GE290234
61934190	CBYT544.g2	GE290235
61934191	CBYT545.b1	GE290236
61934192	CBYT545.g2	GE290237
61934193	CBYT546.b1	GE290238
61934194	CBYT546.g2	GE290239
61934195	CBYT547.b1	GE290240
61934196	CBYT547.g2	GE290241
61934197	CBYT548.b1	GE290242
61934198	CBYT549.b1	GE290243
61934199	CBYT549.g2	GE290244
61934200	CBYT551.b1	GE290245
61934201	CBYT551.g2	GE290246
61934202	CBYT552.b1	GE290247
61934203	CBYT552.g2	GE290248
61934204	CBYT553.b1	GE290249
61934205	CBYT553.g2	GE290250
61934206	CBYT554.b1	GE290251
61934207	CBYT554.g2	GE290252
61934208	CBYT555.b1	GE290253
61934209	CBYT555.g2	GE290254
61934210	CBYT557.b1	GE290255
61934211	CBYT557.g2	GE290256
61934212	CBYT558.b1	GE290257
61934213	CBYT558.g2	GE290258
61934214	CBYT559.b1	GE290259
61934215	CBYT559.g2	GE290260
61934216	CBYT560.b1	GE290261
61934217	CBYT560.g2	GE290262
61934218	CBYT561.b1	GE290263
61934219	CBYT561.g2	GE290264
61934220	CBYT562.b1	GE290265
61934221	CBYT562.g2	GE290266
61934222	CBYT563.b1	GE290267
61934223	CBYT564.b1	GE290268
61934224	CBYT565.b1	GE290269
61934225	CBYT565.g2	GE290270
61934226	CBYT566.b1	GE290271
61934227	CBYT566.g2	GE290272
61934228	CBYT567.b1	GE290273
61934229	CBYT567.g2	GE290274
61934230	CBYT568.b1	GE290275
61934231	CBYT568.g2	GE290276
61934232	CBYT569.b1	GE290277

61934233	CBYT569.g2	GE290278
61934234	CBYT570.b1	GE290279
61934235	CBYT570.g2	GE290280
61934236	CBYT571.b1	GE290281
61934237	CBYT571.g2	GE290282
61934238	CBYT572.b1	GE290283
61934239	CBYT572.g2	GE290284
61934240	CBYT573.b1	GE290285
61934241	CBYT573.g2	GE290286
61934242	CBYT574.b1	GE290287
61934243	CBYT574.g2	GE290288
61934244	CBYT575.b1	GE290289
61934245	CBYT575.g2	GE290290
61934246	CBYT576.b1	GE290291
61934247	CBYT576.g2	GE290292
61934248	CBYT577.b1	GE290293
61934249	CBYT577.g2	GE290294
61934250	CBYT578.b1	GE290295
61934251	CBYT578.g2	GE290296
61934252	CBYT579.b1	GE290297
61934253	CBYT579.g2	GE290298
61934254	CBYT580.b1	GE290299
61934255	CBYT580.g2	GE290300
61934256	CBYT581.b1	GE290301
61934257	CBYT581.g2	GE290302
61934258	CBYT582.b1	GE290303
61934259	CBYT582.g2	GE290304
61934260	CBYT583.b1	GE290305
61934261	CBYT583.g2	GE290306
61934262	CBYT584.b1	GE290307
61934263	CBYT584.g2	GE290308
61934264	CBYT585.b1	GE290309
61934265	CBYT585.g2	GE290310
61934266	CBYT586.b1	GE290311
61934267	CBYT586.g2	GE290312
61934268	CBYT587.b1	GE290313
61934269	CBYT587.g2	GE290314
61934270	CBYT588.b1	GE290315
61934271	CBYT588.g2	GE290316
61934272	CBYT589.b1	GE290317
61934273	CBYT589.g2	GE290318
61934274	CBYT590.b1	GE290319
61934275	CBYT590.g2	GE290320
61934276	CBYT591.b1	GE290321
61934277	CBYT591.g2	GE290322
61934278	CBYT593.b1	GE290323
61934279	CBYT593.g2	GE290324
61934280	CBYT594.b1	GE290325
61934281	CBYT594.g2	GE290326
61934282	CBYT595.g2	GE290327
61934283	CBYT596.b1	GE290328
61934284	CBYT597.b1	GE290329
61934285	CBYT597.g2	GE290330
61934286	CBYT598.b1	GE290331
61934287	CBYT598.g2	GE290332
61934288	CBYT599.b1	GE290333
61934289	CBYT599.g2	GE290334
61934290	CBYT600.b1	GE290335
61934291	CBYT600.g2	GE290336
61934292	CBYT601.g2	GE290337
61934293	CBYT602.b1	GE290338
61934294	CBYT602.g2	GE290339
61934295	CBYT604.b1	GE290340

61934296	CBYT604.g2	GE290341
61934297	CBYT605.b1	GE290342
61934298	CBYT605.g2	GE290343
61934299	CBYT606.b1	GE290344
61934300	CBYT606.g2	GE290345
61934301	CBYT607.b1	GE290346
61934302	CBYT607.g2	GE290347
61934303	CBYT608.b1	GE290348
61934304	CBYT608.g2	GE290349
61934305	CBYT609.b1	GE290350
61934306	CBYT609.g2	GE290351
61934307	CBYT610.b1	GE290352
61934308	CBYT610.g2	GE290353
61934309	CBYT611.b1	GE290354
61934310	CBYT611.g2	GE290355
61934311	CBYT613.b1	GE290356
61934312	CBYT613.g2	GE290357
61934313	CBYT614.b1	GE290358
61934314	CBYT614.g2	GE290359
61934315	CBYT615.b1	GE290360
61934316	CBYT615.g2	GE290361
61934317	CBYT616.b1	GE290362
61934318	CBYT616.g2	GE290363
61934319	CBYT617.b1	GE290364
61934320	CBYT617.g2	GE290365
61934321	CBYT618.b1	GE290366
61934322	CBYT618.g2	GE290367
61934323	CBYT619.b1	GE290368
61934324	CBYT620.b1	GE290369
61934325	CBYT620.g2	GE290370
61934326	CBYT621.b1	GE290371
61934327	CBYT621.g2	GE290372
61934328	CBYT622.b1	GE290373
61934329	CBYT622.g2	GE290374
61934330	CBYT624.g2	GE290375
61934331	CBYT625.b1	GE290376
61934332	CBYT625.g2	GE290377
61934333	CBYT626.b1	GE290378
61934334	CBYT626.g2	GE290379
61934335	CBYT627.b1	GE290380
61934336	CBYT627.g2	GE290381
61934337	CBYT628.b1	GE290382
61934338	CBYT628.g2	GE290383
61934339	CBYT629.b1	GE290384
61934340	CBYT630.b1	GE290385
61934341	CBYT630.g2	GE290386
61934342	CBYT631.b1	GE290387
61934343	CBYT631.g2	GE290388
61934344	CBYT632.b1	GE290389
61934345	CBYT632.g2	GE290390
61934346	CBYT634.b1	GE290391
61934347	CBYT634.g2	GE290392
61934348	CBYT635.b1	GE290393
61934349	CBYT635.g2	GE290394
61934350	CBYT637.b1	GE290395
61934351	CBYT637.g2	GE290396
61934352	CBYT638.b1	GE290397
61934353	CBYT638.g2	GE290398
61934354	CBYT639.b1	GE290399
61934355	CBYT639.g2	GE290400
61934356	CBYT640.b1	GE290401
61934357	CBYT640.g2	GE290402
61934358	CBYT641.b1	GE290403

61934359	CBYT641.g2	GE290404
61934360	CBYT642.b1	GE290405
61934361	CBYT642.g2	GE290406
61934362	CBYT643.b1	GE290407
61934363	CBYT643.g2	GE290408
61934364	CBYT644.g2	GE290409
61934365	CBYT645.b1	GE290410
61934366	CBYT645.g2	GE290411
61934367	CBYT646.b1	GE290412
61934368	CBYT646.g2	GE290413
61934369	CBYT648.b1	GE290414
61934370	CBYT648.g2	GE290415
61934371	CBYT649.b1	GE290416
61934372	CBYT649.g2	GE290417
61934373	CBYT650.b1	GE290418
61934374	CBYT650.g2	GE290419
61934375	CBYT651.b1	GE290420
61934376	CBYT651.g2	GE290421
61934377	CBYT652.b1	GE290422
61934378	CBYT652.g2	GE290423
61934379	CBYT653.b1	GE290424
61934380	CBYT654.b1	GE290425
61934381	CBYT654.g2	GE290426
61934382	CBYT655.b1	GE290427
61934383	CBYT655.g2	GE290428
61934384	CBYT656.b1	GE290429
61934385	CBYT656.g2	GE290430
61934386	CBYT657.b1	GE290431
61934387	CBYT657.g2	GE290432
61934388	CBYT658.b1	GE290433
61934389	CBYT658.g2	GE290434
61934390	CBYT659.b1	GE290435
61934391	CBYT659.g2	GE290436
61934392	CBYT660.b1	GE290437
61934393	CBYT660.g2	GE290438
61934394	CBYT661.b1	GE290439
61934395	CBYT661.g2	GE290440
61934396	CBYT663.b1	GE290441
61934397	CBYT663.g2	GE290442
61934398	CBYT664.b1	GE290443
61934399	CBYT664.g2	GE290444
61934400	CBYT666.b1	GE290445
61934401	CBYT666.g2	GE290446
61934402	CBYT667.b1	GE290447
61934403	CBYT667.g2	GE290448
61934404	CBYT668.b1	GE290449
61934405	CBYT668.g2	GE290450
61934406	CBYT669.b1	GE290451
61934407	CBYT669.g2	GE290452
61934408	CBYT670.b1	GE290453
61934409	CBYT670.g2	GE290454
61934410	CBYT671.b1	GE290455
61934411	CBYT671.g2	GE290456
61934412	CBYT672.b1	GE290457
61934413	CBYT672.g2	GE290458
61934414	CBYT673.b1	GE290459
61934415	CBYT673.g2	GE290460
61934416	CBYT674.b1	GE290461
61934417	CBYT674.g2	GE290462
61934418	CBYT675.b1	GE290463
61934419	CBYT675.g2	GE290464
61934420	CBYT676.b1	GE290465
61934421	CBYT676.g2	GE290466

61934422	CBYT677.b1	GE290467
61934423	CBYT677.g2	GE290468
61934424	CBYT678.b1	GE290469
61934425	CBYT679.b1	GE290470
61934426	CBYT679.g2	GE290471
61934427	CBYT680.b1	GE290472
61934428	CBYT680.g2	GE290473
61934429	CBYT681.b1	GE290474
61934430	CBYT681.g2	GE290475
61934431	CBYT682.b1	GE290476
61934432	CBYT682.g2	GE290477
61934433	CBYT683.b1	GE290478
61934434	CBYT683.g2	GE290479
61934435	CBYT684.b1	GE290480
61934436	CBYT684.g2	GE290481
61934437	CBYT685.b1	GE290482
61934438	CBYT685.g2	GE290483
61934439	CBYT686.b1	GE290484
61934440	CBYT686.g2	GE290485
61934441	CBYT687.b1	GE290486
61934442	CBYT687.g2	GE290487
61934443	CBYT688.b1	GE290488
61934444	CBYT688.g2	GE290489
61934445	CBYT689.b1	GE290490
61934446	CBYT689.g2	GE290491
61934447	CBYT690.b1	GE290492
61934448	CBYT690.g2	GE290493
61934449	CBYT692.b1	GE290494
61934450	CBYT692.g2	GE290495
61934451	CBYT693.b1	GE290496
61934452	CBYT693.g2	GE290497
61934453	CBYT694.b1	GE290498
61934454	CBYT694.g2	GE290499
61934455	CBYT695.b1	GE290500
61934456	CBYT695.g2	GE290501
61934457	CBYT696.b1	GE290502
61934458	CBYT696.g2	GE290503
61934459	CBYT697.b1	GE290504
61934460	CBYT697.g2	GE290505
61934461	CBYT698.b1	GE290506
61934462	CBYT698.g2	GE290507
61934463	CBYT699.b1	GE290508
61934464	CBYT699.g2	GE290509
61934465	CBYT700.b1	GE290510
61934466	CBYT700.g2	GE290511
61934467	CBYT701.b1	GE290512
61934468	CBYT701.g2	GE290513
61934469	CBYT702.b1	GE290514
61934470	CBYT702.g2	GE290515
61934471	CBYT703.b1	GE290516
61934472	CBYT703.g2	GE290517
61934473	CBYT704.b1	GE290518
61934474	CBYT704.g2	GE290519
61934475	CBYT705.b1	GE290520
61934476	CBYT705.g2	GE290521
61934477	CBYT706.b1	GE290522
61934478	CBYT706.g2	GE290523
61934479	CBYT707.b1	GE290524
61934480	CBYT707.g2	GE290525
61934481	CBYT708.b1	GE290526
61934482	CBYT708.g2	GE290527
61934483	CBYT709.b1	GE290528
61934484	CBYT709.g2	GE290529

61934485	CBYT710.b1	GE290530
61934486	CBYT710.g2	GE290531
61934487	CBYT711.b1	GE290532
61934488	CBYT711.g2	GE290533
61934489	CBYT712.b1	GE290534
61934490	CBYT712.g2	GE290535
61934491	CBYT713.b1	GE290536
61934492	CBYT713.g2	GE290537
61934493	CBYT714.b1	GE290538
61934494	CBYT714.g2	GE290539
61934495	CBYT715.b1	GE290540
61934496	CBYT715.g2	GE290541
61934497	CBYT716.b1	GE290542
61934498	CBYT716.g2	GE290543
61934499	CBYT717.b1	GE290544
61934500	CBYT717.g2	GE290545
61934501	CBYT718.b1	GE290546
61934502	CBYT718.g2	GE290547
61934503	CBYT719.b1	GE290548
61934504	CBYT719.g2	GE290549
61934505	CBYT720.b1	GE290550
61934506	CBYT720.g2	GE290551
61934507	CBYT721.b1	GE290552
61934508	CBYT721.g2	GE290553
61934509	CBYT722.b1	GE290554
61934510	CBYT722.g2	GE290555
61934511	CBYT723.b1	GE290556
61934512	CBYT723.g2	GE290557
61934513	CBYT724.b1	GE290558
61934514	CBYT724.g2	GE290559
61934515	CBYT725.b1	GE290560
61934516	CBYT725.g2	GE290561
61934517	CBYT726.b1	GE290562
61934518	CBYT726.g2	GE290563
61934519	CBYT727.b1	GE290564
61934520	CBYT727.g2	GE290565
61934521	CBYT728.b1	GE290566
61934522	CBYT728.g2	GE290567
61934523	CBYT729.b1	GE290568
61934524	CBYT730.b1	GE290569
61934525	CBYT730.g2	GE290570
61934526	CBYT731.b1	GE290571
61934527	CBYT731.g2	GE290572
61934528	CBYT732.b1	GE290573
61934529	CBYT732.g2	GE290574
61934530	CBYT733.b1	GE290575
61934531	CBYT733.g2	GE290576
61934532	CBYT734.b1	GE290577
61934533	CBYT734.g2	GE290578
61934534	CBYT735.b1	GE290579
61934535	CBYT735.g2	GE290580
61934536	CBYT736.b1	GE290581
61934537	CBYT736.g2	GE290582
61934538	CBYT737.b1	GE290583
61934539	CBYT737.g2	GE290584
61934540	CBYT738.b1	GE290585
61934541	CBYT738.g2	GE290586
61934542	CBYT739.b1	GE290587
61934543	CBYT739.g2	GE290588
61934544	CBYT740.g2	GE290589
61934545	CBYT741.b1	GE290590
61934546	CBYT741.g2	GE290591
61934547	CBYT742.b1	GE290592

61934548	CBYT742.g2	GE290593
61934549	CBYT743.b1	GE290594
61934550	CBYT743.g2	GE290595
61934551	CBYT744.b1	GE290596
61934552	CBYT744.g2	GE290597
61934553	CBYT745.b1	GE290598
61934554	CBYT745.g2	GE290599
61934555	CBYT746.b1	GE290600
61934556	CBYT746.g2	GE290601
61934557	CBYT747.b1	GE290602
61934558	CBYT747.g2	GE290603
61934559	CBYT748.b1	GE290604
61934560	CBYT748.g2	GE290605
61934561	CBYT749.b1	GE290606
61934562	CBYT749.g2	GE290607
61934563	CBYT750.b1	GE290608
61934564	CBYT750.g2	GE290609
61934565	CBYT751.b1	GE290610
61934566	CBYT751.g2	GE290611
61934567	CBYT752.b1	GE290612
61934568	CBYT752.g2	GE290613
61934569	CBYT753.b1	GE290614
61934570	CBYT753.g2	GE290615
61934571	CBYT755.b1	GE290616
61934572	CBYT755.g2	GE290617
61934573	CBYT756.b1	GE290618
61934574	CBYT756.g2	GE290619
61934575	CBYT757.b1	GE290620
61934576	CBYT757.g2	GE290621
61934577	CBYT758.b1	GE290622
61934578	CBYT758.g2	GE290623
61934579	CBYT759.b1	GE290624
61934580	CBYT759.g2	GE290625
61934581	CBYT760.b1	GE290626
61934582	CBYT760.g2	GE290627
61934583	CBYT761.b1	GE290628
61934584	CBYT761.g2	GE290629
61934585	CBYT762.b1	GE290630
61934586	CBYT762.g2	GE290631
61934587	CBYT763.b1	GE290632
61934588	CBYT763.g2	GE290633
61934589	CBYT764.b1	GE290634
61934590	CBYT764.g2	GE290635
61934591	CBYT765.b1	GE290636
61934592	CBYT765.g2	GE290637
61934593	CBYT766.b1	GE290638
61934594	CBYT766.g2	GE290639
61934595	CBYT767.b1	GE290640
61934596	CBYT768.b1	GE290641
61934597	CBYT768.g2	GE290642
61934598	CBYT769.b1	GE290643
61934599	CBYT769.g1	GE290644
61934600	CBYT770.b1	GE290645
61934601	CBYT770.g1	GE290646
61934602	CBYT771.b1	GE290647
61934603	CBYT771.g1	GE290648
61934604	CBYT772.b1	GE290649
61934605	CBYT772.g1	GE290650
61934606	CBYT773.g1	GE290651
61934607	CBYT774.b1	GE290652
61934608	CBYT774.g1	GE290653
61934609	CBYT775.g1	GE290654
61934610	CBYT776.b1	GE290655

61934611	CBYT776.g1	GE290656
61934612	CBYT777.g1	GE290657
61934613	CBYT779.b1	GE290658
61934614	CBYT779.g1	GE290659
61934615	CBYT780.b1	GE290660
61934616	CBYT781.b1	GE290661
61934617	CBYT781.g1	GE290662
61934618	CBYT782.b1	GE290663
61934619	CBYT782.g1	GE290664
61934620	CBYT783.b1	GE290665
61934621	CBYT783.g1	GE290666
61934622	CBYT784.b1	GE290667
61934623	CBYT784.g1	GE290668
61934624	CBYT785.b1	GE290669
61934625	CBYT785.g1	GE290670
61934626	CBYT786.b1	GE290671
61934627	CBYT786.g1	GE290672
61934628	CBYT787.b1	GE290673
61934629	CBYT787.g1	GE290674
61934630	CBYT788.b1	GE290675
61934631	CBYT788.g1	GE290676
61934632	CBYT789.g1	GE290677
61934633	CBYT790.b1	GE290678
61934634	CBYT790.g1	GE290679
61934635	CBYT791.g1	GE290680
61934636	CBYT792.b1	GE290681
61934637	CBYT792.g1	GE290682
61934638	CBYT793.b1	GE290683
61934639	CBYT793.g1	GE290684
61934640	CBYT794.g1	GE290685
61934641	CBYT795.g1	GE290686
61934642	CBYT797.b1	GE290687
61934643	CBYT797.g1	GE290688
61934644	CBYT798.b1	GE290689
61934645	CBYT798.g1	GE290690
61934646	CBYT799.b1	GE290691
61934647	CBYT799.g1	GE290692
61934648	CBYT800.g1	GE290693
61934649	CBYT801.b1	GE290694
61934650	CBYT801.g1	GE290695
61934651	CBYT802.g1	GE290696
61934652	CBYT805.g1	GE290697
61934653	CBYT806.b1	GE290698
61934654	CBYT806.g1	GE290699
61934655	CBYT807.b1	GE290700
61934656	CBYT807.g1	GE290701
61934657	CBYT808.b1	GE290702
61934658	CBYT808.g1	GE290703
61934659	CBYT809.b1	GE290704
61934660	CBYT809.g1	GE290705
61934661	CBYT811.g1	GE290706
61934662	CBYT812.b1	GE290707
61934663	CBYT812.g1	GE290708
61934664	CBYT813.b1	GE290709
61934665	CBYT813.g1	GE290710
61934666	CBYT814.b1	GE290711
61934667	CBYT814.g1	GE290712
61934668	CBYT815.b1	GE290713
61934669	CBYT815.g1	GE290714
61934670	CBYT816.b1	GE290715
61934671	CBYT816.g1	GE290716
61934672	CBYT817.b1	GE290717
61934673	CBYT817.g1	GE290718

61934674	CBYT818.g1	GE290719
61934675	CBYT819.b1	GE290720
61934676	CBYT819.g1	GE290721
61934677	CBYT820.b1	GE290722
61934678	CBYT820.g1	GE290723
61934679	CBYT821.b1	GE290724
61934680	CBYT822.b1	GE290725
61934681	CBYT822.g1	GE290726
61934682	CBYT823.b1	GE290727
61934683	CBYT823.g1	GE290728
61934684	CBYT824.b1	GE290729
61934685	CBYT824.g1	GE290730
61934686	CBYT825.b1	GE290731
61934687	CBYT825.g1	GE290732
61934688	CBYT826.g1	GE290733
61934689	CBYT827.b1	GE290734
61934690	CBYT827.g1	GE290735
61934691	CBYT828.b1	GE290736
61934692	CBYT828.g1	GE290737
61934693	CBYT829.b1	GE290738
61934694	CBYT829.g1	GE290739
61934695	CBYT830.b1	GE290740
61934696	CBYT830.g1	GE290741
61934697	CBYT831.b1	GE290742
61934698	CBYT831.g1	GE290743
61934699	CBYT832.b1	GE290744
61934700	CBYT832.g1	GE290745
61934701	CBYT833.b1	GE290746
61934702	CBYT833.g1	GE290747
61934703	CBYT834.g1	GE290748
61934704	CBYT836.g1	GE290749
61934705	CBYT837.b1	GE290750
61934706	CBYT837.g1	GE290751
61934707	CBYT838.b1	GE290752
61934708	CBYT838.g1	GE290753
61934709	CBYT839.b1	GE290754
61934710	CBYT839.g1	GE290755
61934711	CBYT840.b1	GE290756
61934712	CBYT840.g1	GE290757
61934713	CBYT841.b1	GE290758
61934714	CBYT841.g1	GE290759
61934715	CBYT842.g1	GE290760
61934716	CBYT843.b1	GE290761
61934717	CBYT843.g1	GE290762
61934718	CBYT844.b1	GE290763
61934719	CBYT844.g1	GE290764
61934720	CBYT845.b1	GE290765
61934721	CBYT845.g1	GE290766
61934722	CBYT846.b1	GE290767
61934723	CBYT846.g1	GE290768
61934724	CBYT847.b1	GE290769
61934725	CBYT847.g1	GE290770
61934726	CBYT849.g1	GE290771
61934727	CBYT850.g1	GE290772
61934728	CBYT851.g1	GE290773
61934729	CBYT852.b1	GE290774
61934730	CBYT852.g1	GE290775
61934731	CBYT853.b1	GE290776
61934732	CBYT853.g1	GE290777
61934733	CBYT854.b1	GE290778
61934734	CBYT854.g1	GE290779
61934735	CBYT855.b1	GE290780
61934736	CBYT855.g1	GE290781

61934737	CBYT856.b1	GE290782
61934738	CBYT856.g1	GE290783
61934739	CBYT857.g1	GE290784
61934740	CBYT858.g1	GE290785
61934741	CBYT859.b1	GE290786
61934742	CBYT859.g1	GE290787
61934743	CBYT860.b1	GE290788
61934744	CBYT860.g1	GE290789
61934745	CBYT861.b1	GE290790
61934746	CBYT861.g1	GE290791
61934747	CBYT862.b1	GE290792
61934748	CBYT862.g1	GE290793
61934749	CBYT863.b1	GE290794
61934750	CBYT863.g1	GE290795
61934751	CBYT865.b1	GE290796
61934752	CBYT865.g1	GE290797
61934753	CBYT866.b1	GE290798
61934754	CBYT866.g1	GE290799
61934755	CBYT867.b1	GE290800
61934756	CBYT867.g1	GE290801
61934757	CBYT868.b1	GE290802
61934758	CBYT868.g1	GE290803
61934759	CBYT870.b1	GE290804
61934760	CBYT870.g1	GE290805
61934761	CBYT871.b1	GE290806
61934762	CBYT871.g1	GE290807
61934763	CBYT872.b1	GE290808
61934764	CBYT872.g1	GE290809
61934765	CBYT873.b1	GE290810
61934766	CBYT873.g1	GE290811
61934767	CBYT874.b1	GE290812
61934768	CBYT874.g1	GE290813
61934769	CBYT875.b1	GE290814
61934770	CBYT875.g1	GE290815
61934771	CBYT876.b1	GE290816
61934772	CBYT876.g1	GE290817
61934773	CBYT877.b1	GE290818
61934774	CBYT877.g1	GE290819
61934775	CBYT878.b1	GE290820
61934776	CBYT878.g1	GE290821
61934777	CBYT879.b1	GE290822
61934778	CBYT879.g1	GE290823
61934779	CBYT880.b1	GE290824
61934780	CBYT881.b1	GE290825
61934781	CBYT881.g1	GE290826
61934782	CBYT882.b1	GE290827
61934783	CBYT882.g1	GE290828
61934784	CBYT884.b1	GE290829
61934785	CBYT884.g1	GE290830
61934786	CBYT885.b1	GE290831
61934787	CBYT885.g1	GE290832
61934788	CBYT886.b1	GE290833
61934789	CBYT886.g1	GE290834
61934790	CBYT887.g1	GE290835
61934791	CBYT888.g1	GE290836
61934792	CBYT889.b1	GE290837
61934793	CBYT889.g1	GE290838
61934794	CBYT890.b1	GE290839
61934795	CBYT890.g1	GE290840
61934796	CBYT891.b1	GE290841
61934797	CBYT891.g1	GE290842
61934798	CBYT892.b1	GE290843
61934799	CBYT892.g1	GE290844

61934800	CBYT893.b1	GE290845
61934801	CBYT893.g1	GE290846
61934802	CBYT894.b1	GE290847
61934803	CBYT894.g1	GE290848
61934804	CBYT895.g1	GE290849
61934805	CBYT896.b1	GE290850
61934806	CBYT896.g1	GE290851
61934807	CBYT897.b1	GE290852
61934808	CBYT897.g1	GE290853
61934809	CBYT898.g1	GE290854
61934810	CBYT899.b1	GE290855
61934811	CBYT899.g1	GE290856
61934812	CBYT900.b1	GE290857
61934813	CBYT900.g1	GE290858
61934814	CBYT901.g1	GE290859
61934815	CBYT902.b1	GE290860
61934816	CBYT902.g1	GE290861
61934817	CBYT903.b1	GE290862
61934818	CBYT903.g1	GE290863
61934819	CBYT904.g1	GE290864
61934820	CBYT905.b1	GE290865
61934821	CBYT905.g1	GE290866
61934822	CBYT906.g1	GE290867
61934823	CBYT907.b1	GE290868
61934824	CBYT907.g1	GE290869
61934825	CBYT908.b1	GE290870
61934826	CBYT908.g1	GE290871
61934827	CBYT909.b1	GE290872
61934828	CBYT909.g1	GE290873
61934829	CBYT910.g1	GE290874
61934830	CBYT911.b1	GE290875
61934831	CBYT911.g1	GE290876
61934832	CBYT912.b1	GE290877
61934833	CBYT912.g1	GE290878
61934834	CBYT913.g1	GE290879
61934835	CBYT915.b1	GE290880
61934836	CBYT915.g1	GE290881
61934837	CBYT916.b1	GE290882
61934838	CBYT916.g1	GE290883
61934839	CBYT917.b1	GE290884
61934840	CBYT917.g1	GE290885
61934841	CBYT918.b1	GE290886
61934842	CBYT918.g1	GE290887
61934843	CBYT919.b1	GE290888
61934844	CBYT919.g1	GE290889
61934845	CBYT920.b1	GE290890
61934846	CBYT920.g1	GE290891
61934847	CBYT921.g1	GE290892
61934848	CBYT922.g1	GE290893
61934849	CBYT923.b1	GE290894
61934850	CBYT923.g1	GE290895
61934851	CBYT924.b1	GE290896
61934852	CBYT924.g1	GE290897
61934853	CBYT925.b1	GE290898
61934854	CBYT925.g1	GE290899
61934855	CBYT926.b1	GE290900
61934856	CBYT926.g1	GE290901
61934857	CBYT927.b1	GE290902
61934858	CBYT927.g1	GE290903
61934859	CBYT928.b1	GE290904
61934860	CBYT928.g1	GE290905
61934861	CBYT929.g1	GE290906
61934862	CBYT930.g1	GE290907

61934863	CBYT931.b1	GE290908
61934864	CBYT931.g1	GE290909
61934865	CBYT932.b1	GE290910
61934866	CBYT932.g1	GE290911
61934867	CBYT933.b1	GE290912
61934868	CBYT933.g1	GE290913
61934869	CBYT934.b1	GE290914
61934870	CBYT934.g1	GE290915
61934871	CBYT936.b1	GE290916
61934872	CBYT936.g1	GE290917
61934873	CBYT937.g1	GE290918
61934874	CBYT939.g1	GE290919
61934875	CBYT940.b1	GE290920
61934876	CBYT940.g1	GE290921
61934877	CBYT941.b1	GE290922
61934878	CBYT941.g1	GE290923
61934879	CBYT942.b1	GE290924
61934880	CBYT942.g1	GE290925
61934881	CBYT943.g1	GE290926
61934882	CBYT945.b1	GE290927
61934883	CBYT945.g1	GE290928
61934884	CBYT946.g1	GE290929
61934885	CBYT947.b1	GE290930
61934886	CBYT947.g1	GE290931
61934887	CBYT948.g1	GE290932
61934888	CBYT949.g1	GE290933
61934889	CBYT950.g1	GE290934
61934890	CBYT951.b1	GE290935
61934891	CBYT951.g1	GE290936
61934892	CBYT952.g1	GE290937
61934893	CBYT954.g1	GE290938
61934894	CBYT955.b1	GE290939
61934895	CBYT955.g1	GE290940
61934896	CBYT957.b1	GE290941
61934897	CBYT957.g1	GE290942
61934898	CBYT958.b1	GE290943
61934899	CBYT958.g1	GE290944
61934900	CBYT959.b1	GE290945
61934901	CBYT959.g1	GE290946
61934902	CBYT961.b1	GE290947
61934903	CBYT961.g1	GE290948
61934904	CBYT962.b1	GE290949
61934905	CBYT962.g1	GE290950
61934906	CBYT963.b1	GE290951
61934907	CBYT963.g1	GE290952
61934908	CBYT964.b1	GE290953
61934909	CBYT964.g1	GE290954
61934910	CBYT965.g1	GE290955
61934911	CBYT966.b1	GE290956
61934912	CBYT966.g1	GE290957
61934913	CBYT967.b1	GE290958
61934914	CBYT967.g1	GE290959
61934915	CBYT968.b1	GE290960
61934916	CBYT968.g1	GE290961
61934917	CBYT969.b1	GE290962
61934918	CBYT970.b1	GE290963
61934919	CBYT970.g1	GE290964
61934920	CBYT971.b1	GE290965
61934921	CBYT972.g1	GE290966
61934922	CBYT973.b1	GE290967
61934923	CBYT973.g1	GE290968
61934924	CBYT974.b1	GE290969
61934925	CBYT974.g1	GE290970

61934926	CBYT975.b1	GE290971
61934927	CBYT976.b1	GE290972
61934928	CBYT976.g1	GE290973
61934929	CBYT977.b1	GE290974
61934930	CBYT977.g1	GE290975
61934931	CBYT978.b1	GE290976
61934932	CBYT978.g1	GE290977
61934933	CBYT979.g1	GE290978
61934934	CBYT980.b1	GE290979
61934935	CBYT980.g1	GE290980
61934936	CBYT981.b1	GE290981
61934937	CBYT981.g1	GE290982
61934938	CBYT982.b1	GE290983
61934939	CBYT982.g1	GE290984
61934940	CBYT983.g1	GE290985
61934941	CBYT984.g1	GE290986
61934942	CBYT985.b1	GE290987
61934943	CBYT985.g1	GE290988
61934944	CBYT987.g1	GE290989
61934945	CBYT988.b1	GE290990
61934946	CBYT988.g1	GE290991
61934947	CBYT989.g1	GE290992
61934948	CBYT990.b1	GE290993
61934949	CBYT990.g1	GE290994
61934950	CBYT991.b1	GE290995
61934951	CBYT991.g1	GE290996
61934952	CBYT992.b1	GE290997
61934953	CBYT992.g1	GE290998
61934954	CBYT993.b1	GE290999
61934955	CBYT994.g1	GE291000
61934956	CBYT996.b1	GE291001
61934957	CBYT996.g1	GE291002
61934958	CBYT997.b1	GE291003
61934959	CBYT997.g1	GE291004
61934960	CBYT998.b1	GE291005
61934961	CBYT998.g1	GE291006
61934962	CBYT999.b1	GE291007
61934963	CCAH1000.b1	GE291008
61934964	CCAH1000.g1	GE291009
61934965	CCAH1001.b1	GE291010
61934966	CCAH1001.g1	GE291011
61934967	CCAH1002.b1	GE291012
61934968	CCAH1002.g1	GE291013
61934969	CCAH1003.b1	GE291014
61934970	CCAH1004.b1	GE291015
61934971	CCAH1004.g1	GE291016
61934972	CCAH1005.b1	GE291017
61934973	CCAH1005.g1	GE291018
61934974	CCAH1006.b1	GE291019
61934975	CCAH1006.g1	GE291020
61934976	CCAH1007.b1	GE291021
61934977	CCAH1007.g1	GE291022
61934978	CCAH1008.b1	GE291023
61934979	CCAH1008.g1	GE291024
61934980	CCAH1009.b1	GE291025
61934981	CCAH1009.g1	GE291026
61934982	CCAH1010.b1	GE291027
61934983	CCAH1010.g1	GE291028
61934984	CCAH1011.g1	GE291029
61934985	CCAH1012.b1	GE291030
61934986	CCAH1012.g1	GE291031
61934987	CCAH1013.b1	GE291032
61934988	CCAH1013.g1	GE291033

61934989	CCAH1014.b1	GE291034
61934990	CCAH1015.b1	GE291035
61934991	CCAH1015.g1	GE291036
61934992	CCAH1016.b1	GE291037
61934993	CCAH1016.g1	GE291038
61934994	CCAH1017.b1	GE291039
61934995	CCAH1017.g1	GE291040
61934996	CCAH1018.b1	GE291041
61934997	CCAH1018.g1	GE291042
61934998	CCAH1019.b1	GE291043
61934999	CCAH1019.g1	GE291044
61935000	CCAH1020.b1	GE291045
61935001	CCAH1020.g1	GE291046
61935002	CCAH1021.b1	GE291047
61935003	CCAH1021.g1	GE291048
61935004	CCAH1022.b1	GE291049
61935005	CCAH1022.g1	GE291050
61935006	CCAH1023.b1	GE291051
61935007	CCAH1023.g1	GE291052
61935008	CCAH1024.b1	GE291053
61935009	CCAH1024.g1	GE291054
61935010	CCAH1025.b1	GE291055
61935011	CCAH1025.g1	GE291056
61935012	CCAH1026.b1	GE291057
61935013	CCAH1026.g1	GE291058
61935014	CCAH1028.b1	GE291059
61935015	CCAH1029.b1	GE291060
61935016	CCAH1029.g1	GE291061
61935017	CCAH1031.b1	GE291062
61935018	CCAH1031.g1	GE291063
61935019	CCAH1032.b1	GE291064
61935020	CCAH1032.g1	GE291065
61935021	CCAH1033.b1	GE291066
61935022	CCAH1033.g1	GE291067
61935023	CCAH1034.b1	GE291068
61935024	CCAH1034.g1	GE291069
61935025	CCAH1035.b1	GE291070
61935026	CCAH1035.g1	GE291071
61935027	CCAH1036.b1	GE291072
61935028	CCAH1036.g1	GE291073
61935029	CCAH1037.b1	GE291074
61935030	CCAH1037.g1	GE291075
61935031	CCAH1038.b1	GE291076
61935032	CCAH1038.g1	GE291077
61935033	CCAH1039.b1	GE291078
61935034	CCAH1039.g1	GE291079
61935035	CCAH1040.b1	GE291080
61935036	CCAH1040.g1	GE291081
61935037	CCAH1041.b1	GE291082
61935038	CCAH1041.g1	GE291083
61935039	CCAH1042.b1	GE291084
61935040	CCAH1042.g1	GE291085
61935041	CCAH1043.b1	GE291086
61935042	CCAH1043.g1	GE291087
61935043	CCAH1044.b1	GE291088
61935044	CCAH1044.g1	GE291089
61935045	CCAH1046.b1	GE291090
61935046	CCAH1046.g1	GE291091
61935047	CCAH1047.b1	GE291092
61935048	CCAH1047.g1	GE291093
61935049	CCAH1048.b1	GE291094
61935050	CCAH1048.g1	GE291095
61935051	CCAH1049.b1	GE291096

61935052	CCAH1049.g1	GE291097
61935053	CCAH1051.b1	GE291098
61935054	CCAH1051.g1	GE291099
61935055	CCAH1052.b1	GE291100
61935056	CCAH1052.g1	GE291101
61935057	CCAH1053.b1	GE291102
61935058	CCAH1053.g1	GE291103
61935059	CCAH1054.b1	GE291104
61935060	CCAH1054.g1	GE291105
61935061	CCAH1058.b1	GE291106
61935062	CCAH1058.g1	GE291107
61935063	CCAH1059.b1	GE291108
61935064	CCAH1059.g1	GE291109
61935065	CCAH1062.b1	GE291110
61935066	CCAH1062.g1	GE291111
61935067	CCAH1063.b1	GE291112
61935068	CCAH1063.g1	GE291113
61935069	CCAH1064.b1	GE291114
61935070	CCAH1065.b1	GE291115
61935071	CCAH1065.g1	GE291116
61935072	CCAH1066.b1	GE291117
61935073	CCAH1066.g1	GE291118
61935074	CCAH1067.b1	GE291119
61935075	CCAH1068.b1	GE291120
61935076	CCAH1068.g1	GE291121
61935077	CCAH1069.b1	GE291122
61935078	CCAH1069.g1	GE291123
61935079	CCAH1070.b1	GE291124
61935080	CCAH1070.g1	GE291125
61935081	CCAH1071.b1	GE291126
61935082	CCAH1071.g1	GE291127
61935083	CCAH1072.b1	GE291128
61935084	CCAH1074.b1	GE291129
61935085	CCAH1074.g1	GE291130
61935086	CCAH1075.b1	GE291131
61935087	CCAH1075.g1	GE291132
61935088	CCAH1076.g1	GE291133
61935089	CCAH1077.b1	GE291134
61935090	CCAH1077.g1	GE291135
61935091	CCAH1080.b1	GE291136
61935092	CCAH1080.g1	GE291137
61935093	CCAH1081.b1	GE291138
61935094	CCAH1081.g1	GE291139
61935095	CCAH1083.b1	GE291140
61935096	CCAH1083.g1	GE291141
61935097	CCAH1084.b1	GE291142
61935098	CCAH1085.b1	GE291143
61935099	CCAH1086.b1	GE291144
61935100	CCAH1086.g1	GE291145
61935101	CCAH1087.b1	GE291146
61935102	CCAH1087.g1	GE291147
61935103	CCAH1088.b1	GE291148
61935104	CCAH1088.g1	GE291149
61935105	CCAH1089.b1	GE291150
61935106	CCAH1089.g1	GE291151
61935107	CCAH1090.b1	GE291152
61935108	CCAH1090.g1	GE291153
61935109	CCAH1091.b1	GE291154
61935110	CCAH1091.g1	GE291155
61935111	CCAH1093.b1	GE291156
61935112	CCAH1094.b1	GE291157
61935113	CCAH1094.g1	GE291158
61935114	CCAH1095.b1	GE291159

61935115	CCAH1095.g1	GE291160
61935116	CCAH1096.b1	GE291161
61935117	CCAH1096.g1	GE291162
61935118	CCAH1097.b1	GE291163
61935119	CCAH1097.g1	GE291164
61935120	CCAH1098.b1	GE291165
61935121	CCAH1098.g1	GE291166
61935122	CCAH1099.b1	GE291167
61935123	CCAH1099.g1	GE291168
61935124	CCAH1100.b1	GE291169
61935125	CCAH1100.g1	GE291170
61935126	CCAH1101.b1	GE291171
61935127	CCAH1102.b1	GE291172
61935128	CCAH1102.g1	GE291173
61935129	CCAH1103.b1	GE291174
61935130	CCAH1103.g1	GE291175
61935131	CCAH1104.b1	GE291176
61935132	CCAH1104.g1	GE291177
61935133	CCAH1105.b1	GE291178
61935134	CCAH1105.g1	GE291179
61935135	CCAH1106.b1	GE291180
61935136	CCAH1106.g1	GE291181
61935137	CCAH1107.b1	GE291182
61935138	CCAH1107.g1	GE291183
61935139	CCAH1108.g1	GE291184
61935140	CCAH1109.b1	GE291185
61935141	CCAH1109.g1	GE291186
61935142	CCAH1110.b1	GE291187
61935143	CCAH1110.g1	GE291188
61935144	CCAH1111.b1	GE291189
61935145	CCAH1111.g1	GE291190
61935146	CCAH1112.b1	GE291191
61935147	CCAH1112.g1	GE291192
61935148	CCAH1113.b1	GE291193
61935149	CCAH1113.g1	GE291194
61935150	CCAH1114.b1	GE291195
61935151	CCAH1114.g1	GE291196
61935152	CCAH1115.b1	GE291197
61935153	CCAH1115.g1	GE291198
61935154	CCAH1116.b1	GE291199
61935155	CCAH1116.g1	GE291200
61935156	CCAH1117.b1	GE291201
61935157	CCAH1117.g1	GE291202
61935158	CCAH1118.b1	GE291203
61935159	CCAH1118.g1	GE291204
61935160	CCAH1120.b1	GE291205
61935161	CCAH1120.g1	GE291206
61935162	CCAH1121.b1	GE291207
61935163	CCAH1121.g1	GE291208
61935164	CCAH1122.b1	GE291209
61935165	CCAH1122.g1	GE291210
61935166	CCAH1123.b1	GE291211
61935167	CCAH1123.g1	GE291212
61935168	CCAH1124.b1	GE291213
61935169	CCAH1124.g1	GE291214
61935170	CCAH1128.b1	GE291215
61935171	CCAH1130.b1	GE291216
61935172	CCAH1130.g1	GE291217
61935173	CCAH1131.b1	GE291218
61935174	CCAH1131.g1	GE291219
61935175	CCAH1132.b1	GE291220
61935176	CCAH1132.g1	GE291221
61935177	CCAH1133.g1	GE291222

61935178	CCAH1134.b1	GE291223
61935179	CCAH1134.g1	GE291224
61935180	CCAH1136.b1	GE291225
61935181	CCAH1136.g1	GE291226
61935182	CCAH1137.b1	GE291227
61935183	CCAH1137.g1	GE291228
61935184	CCAH1138.g1	GE291229
61935185	CCAH1139.b1	GE291230
61935186	CCAH1139.g1	GE291231
61935187	CCAH1140.b1	GE291232
61935188	CCAH1140.g1	GE291233
61935189	CCAH1141.b1	GE291234
61935190	CCAH1141.g1	GE291235
61935191	CCAH1142.b1	GE291236
61935192	CCAH1142.g1	GE291237
61935193	CCAH1143.b1	GE291238
61935194	CCAH1143.g1	GE291239
61935195	CCAH1144.b1	GE291240
61935196	CCAH1145.b1	GE291241
61935197	CCAH1145.g1	GE291242
61935198	CCAH1146.b1	GE291243
61935199	CCAH1146.g1	GE291244
61935200	CCAH1147.g1	GE291245
61935201	CCAH1148.b1	GE291246
61935202	CCAH1148.g1	GE291247
61935203	CCAH1149.b1	GE291248
61935204	CCAH1149.g1	GE291249
61935205	CCAH1150.b1	GE291250
61935206	CCAH1150.g1	GE291251
61935207	CCAH1153.b1	GE291252
61935208	CCAH1153.g1	GE291253
61935209	CCAH1155.b1	GE291254
61935210	CCAH1155.g1	GE291255
61935211	CCAH1156.b1	GE291256
61935212	CCAH1156.g1	GE291257
61935213	CCAH1157.b1	GE291258
61935214	CCAH1157.g1	GE291259
61935215	CCAH1158.b1	GE291260
61935216	CCAH1158.g1	GE291261
61935217	CCAH1159.b1	GE291262
61935218	CCAH1159.g1	GE291263
61935219	CCAH1160.b1	GE291264
61935220	CCAH1160.g1	GE291265
61935221	CCAH1161.b1	GE291266
61935222	CCAH1161.g1	GE291267
61935223	CCAH1162.b1	GE291268
61935224	CCAH1162.g1	GE291269
61935225	CCAH1163.b1	GE291270
61935226	CCAH1163.g1	GE291271
61935227	CCAH1164.b1	GE291272
61935228	CCAH1166.b1	GE291273
61935229	CCAH1166.g1	GE291274
61935230	CCAH1168.b1	GE291275
61935231	CCAH1168.g1	GE291276
61935232	CCAH1169.b1	GE291277
61935233	CCAH1169.g1	GE291278
61935234	CCAH1170.b1	GE291279
61935235	CCAH1170.g1	GE291280
61935236	CCAH1171.b1	GE291281
61935237	CCAH1171.g1	GE291282
61935238	CCAH1172.b1	GE291283
61935239	CCAH1172.g1	GE291284
61935240	CCAH1173.b1	GE291285

61935241	CCAH1173.g1	GE291286
61935242	CCAH1174.b1	GE291287
61935243	CCAH1176.b1	GE291288
61935244	CCAH1176.g1	GE291289
61935245	CCAH1177.b1	GE291290
61935246	CCAH1178.b1	GE291291
61935247	CCAH1179.b1	GE291292
61935248	CCAH1179.g1	GE291293
61935249	CCAH1180.b1	GE291294
61935250	CCAH1180.g1	GE291295
61935251	CCAH1181.b1	GE291296
61935252	CCAH1181.g1	GE291297
61935253	CCAH1182.b1	GE291298
61935254	CCAH1182.g1	GE291299
61935255	CCAH1183.b1	GE291300
61935256	CCAH1183.g1	GE291301
61935257	CCAH1184.b1	GE291302
61935258	CCAH1184.g1	GE291303
61935259	CCAH1185.b1	GE291304
61935260	CCAH1185.g1	GE291305
61935261	CCAH1186.b1	GE291306
61935262	CCAH1186.g1	GE291307
61935263	CCAH1187.b1	GE291308
61935264	CCAH1187.g1	GE291309
61935265	CCAH1188.b1	GE291310
61935266	CCAH1188.g1	GE291311
61935267	CCAH1189.b1	GE291312
61935268	CCAH1189.g1	GE291313
61935269	CCAH1190.b1	GE291314
61935270	CCAH1190.g1	GE291315
61935271	CCAH1191.b1	GE291316
61935272	CCAH1191.g1	GE291317
61935273	CCAH1192.b1	GE291318
61935274	CCAH1193.b1	GE291319
61935275	CCAH1194.b1	GE291320
61935276	CCAH1194.g1	GE291321
61935277	CCAH1195.b1	GE291322
61935278	CCAH1195.g1	GE291323
61935279	CCAH1196.b1	GE291324
61935280	CCAH1197.b1	GE291325
61935281	CCAH1197.g1	GE291326
61935282	CCAH1198.b1	GE291327
61935283	CCAH1198.g1	GE291328
61935284	CCAH1199.b1	GE291329
61935285	CCAH1199.g1	GE291330
61935286	CCAH1200.b1	GE291331
61935287	CCAH1200.g1	GE291332
61935288	CCAH1202.b1	GE291333
61935289	CCAH1202.g1	GE291334
61935290	CCAH1203.b1	GE291335
61935291	CCAH1203.g1	GE291336
61935292	CCAH1204.b1	GE291337
61935293	CCAH1205.b1	GE291338
61935294	CCAH1205.g1	GE291339
61935295	CCAH1206.b1	GE291340
61935296	CCAH1206.g1	GE291341
61935297	CCAH1207.b1	GE291342
61935298	CCAH1207.g1	GE291343
61935299	CCAH1208.b1	GE291344
61935300	CCAH1208.g1	GE291345
61935301	CCAH1209.b1	GE291346
61935302	CCAH1209.g1	GE291347
61935303	CCAH1210.b1	GE291348

61935304	CCAH1210.g1	GE291349
61935305	CCAH1211.b1	GE291350
61935306	CCAH1212.b1	GE291351
61935307	CCAH1213.b1	GE291352
61935308	CCAH1213.g1	GE291353
61935309	CCAH1214.b1	GE291354
61935310	CCAH1214.g1	GE291355
61935311	CCAH1215.b1	GE291356
61935312	CCAH1215.g1	GE291357
61935313	CCAH1216.g1	GE291358
61935314	CCAH1218.g1	GE291359
61935315	CCAH1219.b1	GE291360
61935316	CCAH1219.g1	GE291361
61935317	CCAH1220.b1	GE291362
61935318	CCAH1220.g1	GE291363
61935319	CCAH1222.b1	GE291364
61935320	CCAH1223.b1	GE291365
61935321	CCAH1223.g1	GE291366
61935322	CCAH1224.b1	GE291367
61935323	CCAH1224.g1	GE291368
61935324	CCAH1225.b1	GE291369
61935325	CCAH1225.g1	GE291370
61935326	CCAH1226.b1	GE291371
61935327	CCAH1226.g1	GE291372
61935328	CCAH1227.b1	GE291373
61935329	CCAH1227.g1	GE291374
61935330	CCAH1228.b1	GE291375
61935331	CCAH1228.g1	GE291376
61935332	CCAH1230.b1	GE291377
61935333	CCAH1230.g1	GE291378
61935334	CCAH1231.b1	GE291379
61935335	CCAH1231.g1	GE291380
61935336	CCAH1232.g1	GE291381
61935337	CCAH1233.b1	GE291382
61935338	CCAH1233.g1	GE291383
61935339	CCAH1234.b1	GE291384
61935340	CCAH1234.g1	GE291385
61935341	CCAH1235.b1	GE291386
61935342	CCAH1235.g1	GE291387
61935343	CCAH1237.b1	GE291388
61935344	CCAH1237.g1	GE291389
61935345	CCAH1238.b1	GE291390
61935346	CCAH1238.g1	GE291391
61935347	CCAH1239.b1	GE291392
61935348	CCAH1239.g1	GE291393
61935349	CCAH1240.b1	GE291394
61935350	CCAH1240.g1	GE291395
61935351	CCAH1242.b1	GE291396
61935352	CCAH1242.g1	GE291397
61935353	CCAH1243.b1	GE291398
61935354	CCAH1243.g1	GE291399
61935355	CCAH1244.b1	GE291400
61935356	CCAH1244.g1	GE291401
61935357	CCAH1245.b1	GE291402
61935358	CCAH1247.g1	GE291403
61935359	CCAH1248.b1	GE291404
61935360	CCAH1248.g1	GE291405
61935361	CCAH1249.b1	GE291406
61935362	CCAH1249.g1	GE291407
61935363	CCAH1250.b1	GE291408
61935364	CCAH1250.g1	GE291409
61935365	CCAH1251.b1	GE291410
61935366	CCAH1251.g1	GE291411

61935367	CCAH1252.b1	GE291412
61935368	CCAH1252.g1	GE291413
61935369	CCAH1253.b1	GE291414
61935370	CCAH1253.g1	GE291415
61935371	CCAH1254.b1	GE291416
61935372	CCAH1254.g1	GE291417
61935373	CCAH1255.b1	GE291418
61935374	CCAH1255.g1	GE291419
61935375	CCAH1256.b1	GE291420
61935376	CCAH1256.g1	GE291421
61935377	CCAH1257.b1	GE291422
61935378	CCAH1257.g1	GE291423
61935379	CCAH1258.b1	GE291424
61935380	CCAH1259.b1	GE291425
61935381	CCAH1259.g1	GE291426
61935382	CCAH1260.b1	GE291427
61935383	CCAH1260.g1	GE291428
61935384	CCAH1261.b1	GE291429
61935385	CCAH1261.g1	GE291430
61935386	CCAH1262.b1	GE291431
61935387	CCAH1262.g1	GE291432
61935388	CCAH1264.b1	GE291433
61935389	CCAH1265.b1	GE291434
61935390	CCAH1265.g1	GE291435
61935391	CCAH1266.b1	GE291436
61935392	CCAH1266.g1	GE291437
61935393	CCAH1267.b1	GE291438
61935394	CCAH1267.g1	GE291439
61935395	CCAH1269.g1	GE291440
61935396	CCAH1270.b1	GE291441
61935397	CCAH1270.g1	GE291442
61935398	CCAH1271.b1	GE291443
61935399	CCAH1271.g1	GE291444
61935400	CCAH1272.b1	GE291445
61935401	CCAH1272.g1	GE291446
61935402	CCAH1273.b1	GE291447
61935403	CCAH1273.g1	GE291448
61935404	CCAH1274.b1	GE291449
61935405	CCAH1275.b1	GE291450
61935406	CCAH1275.g1	GE291451
61935407	CCAH1276.b1	GE291452
61935408	CCAH1276.g1	GE291453
61935409	CCAH1277.g1	GE291454
61935410	CCAH1278.b1	GE291455
61935411	CCAH1278.g1	GE291456
61935412	CCAH1279.b1	GE291457
61935413	CCAH1279.g1	GE291458
61935414	CCAH1280.b1	GE291459
61935415	CCAH1280.g1	GE291460
61935416	CCAH1281.b1	GE291461
61935417	CCAH1281.g1	GE291462
61935418	CCAH1282.b1	GE291463
61935419	CCAH1282.g1	GE291464
61935420	CCAH1283.b1	GE291465
61935421	CCAH1284.b1	GE291466
61935422	CCAH1284.g1	GE291467
61935423	CCAH1285.b1	GE291468
61935424	CCAH1285.g1	GE291469
61935425	CCAH1286.b1	GE291470
61935426	CCAH1286.g1	GE291471
61935427	CCAH1287.b1	GE291472
61935428	CCAH1287.g1	GE291473
61935429	CCAH1288.b1	GE291474

61935430	CCAH1288.g1	GE291475
61935431	CCAH1289.b1	GE291476
61935432	CCAH1289.g1	GE291477
61935433	CCAH1290.b1	GE291478
61935434	CCAH1290.g1	GE291479
61935435	CCAH1291.b1	GE291480
61935436	CCAH1291.g1	GE291481
61935437	CCAH1292.b1	GE291482
61935438	CCAH1292.g1	GE291483
61935439	CCAH1293.b1	GE291484
61935440	CCAH1293.g1	GE291485
61935441	CCAH1295.b1	GE291486
61935442	CCAH1296.b1	GE291487
61935443	CCAH1296.g1	GE291488
61935444	CCAH1297.g1	GE291489
61935445	CCAH1298.b1	GE291490
61935446	CCAH1298.g1	GE291491
61935447	CCAH1299.b1	GE291492
61935448	CCAH1299.g1	GE291493
61935449	CCAH1300.b1	GE291494
61935450	CCAH1301.b1	GE291495
61935451	CCAH1302.b1	GE291496
61935452	CCAH1302.g1	GE291497
61935453	CCAH1303.b1	GE291498
61935454	CCAH1303.g1	GE291499
61935455	CCAH1304.b1	GE291500
61935456	CCAH1304.g1	GE291501
61935457	CCAH1305.b1	GE291502
61935458	CCAH1305.g1	GE291503
61935459	CCAH1306.b1	GE291504
61935460	CCAH1306.g1	GE291505
61935461	CCAH1307.b1	GE291506
61935462	CCAH1308.b1	GE291507
61935463	CCAH1308.g1	GE291508
61935464	CCAH1309.b1	GE291509
61935465	CCAH1309.g1	GE291510
61935466	CCAH1310.b1	GE291511
61935467	CCAH1310.g1	GE291512
61935468	CCAH1311.b1	GE291513
61935469	CCAH1312.b1	GE291514
61935470	CCAH1312.g1	GE291515
61935471	CCAH1313.b1	GE291516
61935472	CCAH1313.g1	GE291517
61935473	CCAH1315.b1	GE291518
61935474	CCAH1315.g1	GE291519
61935475	CCAH1316.b1	GE291520
61935476	CCAH1316.g1	GE291521
61935477	CCAH1317.b1	GE291522
61935478	CCAH1317.g1	GE291523
61935479	CCAH1318.b1	GE291524
61935480	CCAH1318.g1	GE291525
61935481	CCAH1319.b1	GE291526
61935482	CCAH1319.g1	GE291527
61935483	CCAH1321.b1	GE291528
61935484	CCAH1321.g1	GE291529
61935485	CCAH1323.b1	GE291530
61935486	CCAH1323.g1	GE291531
61935487	CCAH1324.b1	GE291532
61935488	CCAH1324.g1	GE291533
61935489	CCAH1327.b1	GE291534
61935490	CCAH1327.g1	GE291535
61935491	CCAH1328.b1	GE291536
61935492	CCAH1328.g1	GE291537

61935493	CCAH1329.b1	GE291538
61935494	CCAH1329.g1	GE291539
61935495	CCAH1331.b1	GE291540
61935496	CCAH1331.g1	GE291541
61935497	CCAH1332.b1	GE291542
61935498	CCAH1332.g1	GE291543
61935499	CCAH1333.b1	GE291544
61935500	CCAH1334.b1	GE291545
61935501	CCAH1335.b1	GE291546
61935502	CCAH1335.g1	GE291547
61935503	CCAH1336.b1	GE291548
61935504	CCAH1336.g1	GE291549
61935505	CCAH1337.b1	GE291550
61935506	CCAH1337.g1	GE291551
61935507	CCAH1339.b1	GE291552
61935508	CCAH1339.g1	GE291553
61935509	CCAH1340.b1	GE291554
61935510	CCAH1340.g1	GE291555
61935511	CCAH1341.b1	GE291556
61935512	CCAH1341.g1	GE291557
61935513	CCAH1342.b1	GE291558
61935514	CCAH1342.g1	GE291559
61935515	CCAH1343.b1	GE291560
61935516	CCAH1343.g1	GE291561
61935517	CCAH1344.b1	GE291562
61935518	CCAH1344.g1	GE291563
61935519	CCAH1345.b1	GE291564
61935520	CCAH1345.g1	GE291565
61935521	CCAH1346.b1	GE291566
61935522	CCAH1346.g1	GE291567
61935523	CCAH1347.b1	GE291568
61935524	CCAH1348.g1	GE291569
61935525	CCAH1349.b1	GE291570
61935526	CCAH1349.g1	GE291571
61935527	CCAH1350.b1	GE291572
61935528	CCAH1351.b1	GE291573
61935529	CCAH1352.b1	GE291574
61935530	CCAH1352.g1	GE291575
61935531	CCAH1353.b1	GE291576
61935532	CCAH1353.g1	GE291577
61935533	CCAH1354.b1	GE291578
61935534	CCAH1354.g1	GE291579
61935535	CCAH1355.b1	GE291580
61935536	CCAH1355.g1	GE291581
61935537	CCAH1356.b1	GE291582
61935538	CCAH1356.g1	GE291583
61935539	CCAH1357.b1	GE291584
61935540	CCAH1358.b1	GE291585
61935541	CCAH1358.g1	GE291586
61935542	CCAH1359.b1	GE291587
61935543	CCAH1360.b1	GE291588
61935544	CCAH1360.g1	GE291589
61935545	CCAH1361.b1	GE291590
61935546	CCAH1363.b1	GE291591
61935547	CCAH1364.b1	GE291592
61935548	CCAH1364.g1	GE291593
61935549	CCAH1366.b1	GE291594
61935550	CCAH1366.g1	GE291595
61935551	CCAH1367.b1	GE291596
61935552	CCAH1367.g1	GE291597
61935553	CCAH1368.b1	GE291598
61935554	CCAH1368.g1	GE291599
61935555	CCAH1369.b1	GE291600

61935556	CCAH1369.g1	GE291601
61935557	CCAH1370.b1	GE291602
61935558	CCAH1370.g1	GE291603
61935559	CCAH1371.b1	GE291604
61935560	CCAH1371.g1	GE291605
61935561	CCAH1372.b1	GE291606
61935562	CCAH1372.g1	GE291607
61935563	CCAH1373.b1	GE291608
61935564	CCAH1373.g1	GE291609
61935565	CCAH1374.b1	GE291610
61935566	CCAH1376.b1	GE291611
61935567	CCAH1376.g1	GE291612
61935568	CCAH1377.b1	GE291613
61935569	CCAH1377.g1	GE291614
61935570	CCAH1378.b1	GE291615
61935571	CCAH1378.g1	GE291616
61935572	CCAH1379.b1	GE291617
61935573	CCAH1379.g1	GE291618
61935574	CCAH1380.b1	GE291619
61935575	CCAH1380.g1	GE291620
61935576	CCAH1381.b1	GE291621
61935577	CCAH1381.g1	GE291622
61935578	CCAH1382.b1	GE291623
61935579	CCAH1382.g1	GE291624
61935580	CCAH1383.b1	GE291625
61935581	CCAH1383.g1	GE291626
61935582	CCAH1384.b1	GE291627
61935583	CCAH1384.g1	GE291628
61935584	CCAH1385.b1	GE291629
61935585	CCAH1385.g1	GE291630
61935586	CCAH1386.b1	GE291631
61935587	CCAH1386.g1	GE291632
61935588	CCAH1387.b1	GE291633
61935589	CCAH1387.g1	GE291634
61935590	CCAH1388.b1	GE291635
61935591	CCAH1389.b1	GE291636
61935592	CCAH1389.g1	GE291637
61935593	CCAH1390.b1	GE291638
61935594	CCAH1391.b1	GE291639
61935595	CCAH1391.g1	GE291640
61935596	CCAH1392.b1	GE291641
61935597	CCAH1392.g1	GE291642
61935598	CCAH1393.b1	GE291643
61935599	CCAH1393.g1	GE291644
61935600	CCAH1395.b1	GE291645
61935601	CCAH1395.g1	GE291646
61935602	CCAH1396.b1	GE291647
61935603	CCAH1396.g1	GE291648
61935604	CCAH1397.b1	GE291649
61935605	CCAH1397.g1	GE291650
61935606	CCAH1398.b1	GE291651
61935607	CCAH1398.g1	GE291652
61935608	CCAH1399.b1	GE291653
61935609	CCAH1399.g1	GE291654
61935610	CCAH1400.b1	GE291655
61935611	CCAH1400.g1	GE291656
61935612	CCAH1401.b1	GE291657
61935613	CCAH1401.g1	GE291658
61935614	CCAH1402.b1	GE291659
61935615	CCAH1403.b1	GE291660
61935616	CCAH1403.g1	GE291661
61935617	CCAH1404.b1	GE291662
61935618	CCAH1404.g1	GE291663

61935619	CCAH1405.b1	GE291664
61935620	CCAH1405.g1	GE291665
61935621	CCAH1406.b1	GE291666
61935622	CCAH1406.g1	GE291667
61935623	CCAH1407.b1	GE291668
61935624	CCAH1407.g1	GE291669
61935625	CCAH1408.b1	GE291670
61935626	CCAH1408.g1	GE291671
61935627	CCAH1409.b1	GE291672
61935628	CCAH1409.g1	GE291673
61935629	CCAH1410.b1	GE291674
61935630	CCAH1410.g1	GE291675
61935631	CCAH1412.b1	GE291676
61935632	CCAH1412.g1	GE291677
61935633	CCAH1413.b1	GE291678
61935634	CCAH1414.b1	GE291679
61935635	CCAH1414.g1	GE291680
61935636	CCAH1415.b1	GE291681
61935637	CCAH1415.g1	GE291682
61935638	CCAH1416.b1	GE291683
61935639	CCAH1416.g1	GE291684
61935640	CCAH1417.g1	GE291685
61935641	CCAH1418.b1	GE291686
61935642	CCAH1418.g1	GE291687
61935643	CCAH1419.b1	GE291688
61935644	CCAH1419.g1	GE291689
61935645	CCAH1420.b1	GE291690
61935646	CCAH1420.g1	GE291691
61935647	CCAH1421.b1	GE291692
61935648	CCAH1421.g1	GE291693
61935649	CCAH1422.b1	GE291694
61935650	CCAH1422.g1	GE291695
61935651	CCAH1423.b1	GE291696
61935652	CCAH1423.g1	GE291697
61935653	CCAH1424.b1	GE291698
61935654	CCAH1425.b1	GE291699
61935655	CCAH1425.g1	GE291700
61935656	CCAH1426.b1	GE291701
61935657	CCAH1426.g1	GE291702
61935658	CCAH1428.b1	GE291703
61935659	CCAH1428.g1	GE291704
61935660	CCAH1429.b1	GE291705
61935661	CCAH1430.b1	GE291706
61935662	CCAH1430.g1	GE291707
61935663	CCAH1431.b1	GE291708
61935664	CCAH1432.b1	GE291709
61935665	CCAH1432.g1	GE291710
61935666	CCAH1433.b1	GE291711
61935667	CCAH1433.g1	GE291712
61935668	CCAH1434.b1	GE291713
61935669	CCAH1434.g1	GE291714
61935670	CCAH1435.b1	GE291715
61935671	CCAH1436.b1	GE291716
61935672	CCAH1436.g1	GE291717
61935673	CCAH1438.b1	GE291718
61935674	CCAH1438.g1	GE291719
61935675	CCAH1440.b1	GE291720
61935676	CCAH1440.g1	GE291721
61935677	CCAH1441.b1	GE291722
61935678	CCAH1441.g1	GE291723
61935679	CCAH1443.b1	GE291724
61935680	CCAH1443.g1	GE291725
61935681	CCAH1444.b1	GE291726

61935682	CCAH1444.g1	GE291727
61935683	CCAH1445.b1	GE291728
61935684	CCAH1445.g1	GE291729
61935685	CCAH1446.b1	GE291730
61935686	CCAH1448.b1	GE291731
61935687	CCAH1449.b1	GE291732
61935688	CCAH1449.g1	GE291733
61935689	CCAH1450.b1	GE291734
61935690	CCAH1451.b1	GE291735
61935691	CCAH1451.g1	GE291736
61935692	CCAH1452.g1	GE291737
61935693	CCAH1453.b1	GE291738
61935694	CCAH1453.g1	GE291739
61935695	CCAH1454.b1	GE291740
61935696	CCAH1454.g1	GE291741
61935697	CCAH1455.b1	GE291742
61935698	CCAH1455.g1	GE291743
61935699	CCAH1456.b1	GE291744
61935700	CCAH1456.g1	GE291745
61935701	CCAH1457.b1	GE291746
61935702	CCAH1457.g1	GE291747
61935703	CCAH1458.b1	GE291748
61935704	CCAH1458.g1	GE291749
61935705	CCAH1459.b1	GE291750
61935706	CCAH1459.g1	GE291751
61935707	CCAH1460.b1	GE291752
61935708	CCAH1460.g1	GE291753
61935709	CCAH1461.b1	GE291754
61935710	CCAH1462.b1	GE291755
61935711	CCAH1462.g1	GE291756
61935712	CCAH1463.b1	GE291757
61935713	CCAH1463.g1	GE291758
61935714	CCAH1464.b1	GE291759
61935715	CCAH1464.g1	GE291760
61935716	CCAH1465.b1	GE291761
61935717	CCAH1466.b1	GE291762
61935718	CCAH1466.g1	GE291763
61935719	CCAH1467.b1	GE291764
61935720	CCAH1468.b1	GE291765
61935721	CCAH1468.g1	GE291766
61935722	CCAH1469.g1	GE291767
61935723	CCAH1470.b1	GE291768
61935724	CCAH1470.g1	GE291769
61935725	CCAH1471.b1	GE291770
61935726	CCAH1471.g1	GE291771
61935727	CCAH1472.b1	GE291772
61935728	CCAH1472.g1	GE291773
61935729	CCAH1473.b1	GE291774
61935730	CCAH1473.g1	GE291775
61935731	CCAH1474.b1	GE291776
61935732	CCAH1474.g1	GE291777
61935733	CCAH1475.b1	GE291778
61935734	CCAH1475.g1	GE291779
61935735	CCAH1476.b1	GE291780
61935736	CCAH1477.b1	GE291781
61935737	CCAH1477.g1	GE291782
61935738	CCAH1478.b1	GE291783
61935739	CCAH1478.g1	GE291784
61935740	CCAH1479.b1	GE291785
61935741	CCAH1479.g1	GE291786
61935742	CCAH1480.b1	GE291787
61935743	CCAH1480.g1	GE291788
61935744	CCAH1481.b1	GE291789

61935745	CCAH1482.b1	GE291790
61935746	CCAH1482.g1	GE291791
61935747	CCAH1483.b1	GE291792
61935748	CCAH1483.g1	GE291793
61935749	CCAH1485.b1	GE291794
61935750	CCAH1485.g1	GE291795
61935751	CCAH1486.b1	GE291796
61935752	CCAH1486.g1	GE291797
61935753	CCAH1487.b1	GE291798
61935754	CCAH1487.g1	GE291799
61935755	CCAH1488.b1	GE291800
61935756	CCAH1488.g1	GE291801
61935757	CCAH1489.b1	GE291802
61935758	CCAH1489.g1	GE291803
61935759	CCAH1490.b1	GE291804
61935760	CCAH1490.g1	GE291805
61935761	CCAH1491.b1	GE291806
61935762	CCAH1491.g1	GE291807
61935763	CCAH1492.b1	GE291808
61935764	CCAH1493.b1	GE291809
61935765	CCAH1493.g1	GE291810
61935766	CCAH1494.b1	GE291811
61935767	CCAH1494.g1	GE291812
61935768	CCAH1495.b1	GE291813
61935769	CCAH1495.g1	GE291814
61935770	CCAH1496.b1	GE291815
61935771	CCAH1497.b1	GE291816
61935772	CCAH1497.g1	GE291817
61935773	CCAH1498.b1	GE291818
61935774	CCAH1498.g1	GE291819
61935775	CCAH1499.b1	GE291820
61935776	CCAH1500.b1	GE291821
61935777	CCAH1500.g1	GE291822
61935778	CCAH1501.b1	GE291823
61935779	CCAH1501.g1	GE291824
61935780	CCAH1502.b1	GE291825
61935781	CCAH1503.b1	GE291826
61935782	CCAH1503.g1	GE291827
61935783	CCAH1504.b1	GE291828
61935784	CCAH1504.g1	GE291829
61935785	CCAH1505.b1	GE291830
61935786	CCAH1505.g1	GE291831
61935787	CCAH1506.b1	GE291832
61935788	CCAH1506.g1	GE291833
61935789	CCAH1507.b1	GE291834
61935790	CCAH1507.g1	GE291835
61935791	CCAH1508.b1	GE291836
61935792	CCAH1508.g1	GE291837
61935793	CCAH1509.g1	GE291838
61935794	CCAH1510.b1	GE291839
61935795	CCAH1510.g1	GE291840
61935796	CCAH1511.b1	GE291841
61935797	CCAH1511.g1	GE291842
61935798	CCAH1512.b1	GE291843
61935799	CCAH1512.g1	GE291844
61935800	CCAH1514.b1	GE291845
61935801	CCAH1515.b1	GE291846
61935802	CCAH1516.b1	GE291847
61935803	CCAH1516.g1	GE291848
61935804	CCAH1517.b1	GE291849
61935805	CCAH1517.g1	GE291850
61935806	CCAH1518.b1	GE291851
61935807	CCAH1518.g1	GE291852

61935808	CCAH1519.b1	GE291853
61935809	CCAH1519.g1	GE291854
61935810	CCAH1520.b1	GE291855
61935811	CCAH1521.b1	GE291856
61935812	CCAH1521.g1	GE291857
61935813	CCAH1522.b1	GE291858
61935814	CCAH1522.g1	GE291859
61935815	CCAH1523.b1	GE291860
61935816	CCAH1523.g1	GE291861
61935817	CCAH1524.b1	GE291862
61935818	CCAH1524.g1	GE291863
61935819	CCAH1525.b1	GE291864
61935820	CCAH1525.g1	GE291865
61935821	CCAH1526.b1	GE291866
61935822	CCAH1526.g1	GE291867
61935823	CCAH1527.b1	GE291868
61935824	CCAH1527.g1	GE291869
61935825	CCAH1528.b1	GE291870
61935826	CCAH1528.g1	GE291871
61935827	CCAH1529.b1	GE291872
61935828	CCAH1529.g1	GE291873
61935829	CCAH1531.b1	GE291874
61935830	CCAH1531.g1	GE291875
61935831	CCAH1532.b1	GE291876
61935832	CCAH1532.g1	GE291877
61935833	CCAH1533.b1	GE291878
61935834	CCAH1533.g1	GE291879
61935835	CCAH1534.b1	GE291880
61935836	CCAH1535.b1	GE291881
61935837	CCAH1535.g1	GE291882
61935838	CCAH1536.b1	GE291883
61935839	CCAH1536.g1	GE291884
61935840	CCAH1537.b1	GE291885
61935841	CCAH1537.g1	GE291886
61935842	CCAH1538.b1	GE291887
61935843	CCAH1538.g1	GE291888
61935844	CCAH1539.b1	GE291889
61935845	CCAH1539.g1	GE291890
61935846	CCAH1540.b1	GE291891
61935847	CCAH1540.g1	GE291892
61935848	CCAH1541.b1	GE291893
61935849	CCAH1542.b1	GE291894
61935850	CCAH1542.g1	GE291895
61935851	CCAH1543.b1	GE291896
61935852	CCAH1543.g1	GE291897
61935853	CCAH1544.b1	GE291898
61935854	CCAH1544.g1	GE291899
61935855	CCAH1545.b1	GE291900
61935856	CCAH1545.g1	GE291901
61935857	CCAH1546.b1	GE291902
61935858	CCAH1546.g1	GE291903
61935859	CCAH1547.b1	GE291904
61935860	CCAH1548.b1	GE291905
61935861	CCAH1548.g1	GE291906
61935862	CCAH1549.b1	GE291907
61935863	CCAH1549.g1	GE291908
61935864	CCAH1551.b1	GE291909
61935865	CCAH1551.g1	GE291910
61935866	CCAH1552.g1	GE291911
61935867	CCAH1553.b1	GE291912
61935868	CCAH1553.g1	GE291913
61935869	CCAH1554.b1	GE291914
61935870	CCAH1554.g1	GE291915

61935871	CCAH1555.b1	GE291916
61935872	CCAH1555.g1	GE291917
61935873	CCAH1556.b1	GE291918
61935874	CCAH1556.g1	GE291919
61935875	CCAH1557.b1	GE291920
61935876	CCAH1557.g1	GE291921
61935877	CCAH1559.b1	GE291922
61935878	CCAH1559.g1	GE291923
61935879	CCAH1560.b1	GE291924
61935880	CCAH1560.g1	GE291925
61935881	CCAH1561.b1	GE291926
61935882	CCAH1561.g1	GE291927
61935883	CCAH1562.b1	GE291928
61935884	CCAH1563.b1	GE291929
61935885	CCAH1563.g1	GE291930
61935886	CCAH1564.g1	GE291931
61935887	CCAH1566.b1	GE291932
61935888	CCAH1566.g1	GE291933
61935889	CCAH1567.b1	GE291934
61935890	CCAH1567.g1	GE291935
61935891	CCAH1568.b1	GE291936
61935892	CCAH1568.g1	GE291937
61935893	CCAH1569.b1	GE291938
61935894	CCAH1569.g1	GE291939
61935895	CCAH1570.b1	GE291940
61935896	CCAH1570.g1	GE291941
61935897	CCAH1571.b1	GE291942
61935898	CCAH1571.g1	GE291943
61935899	CCAH1572.b1	GE291944
61935900	CCAH1572.g1	GE291945
61935901	CCAH1573.g1	GE291946
61935902	CCAH1574.b1	GE291947
61935903	CCAH1574.g1	GE291948
61935904	CCAH1575.b1	GE291949
61935905	CCAH1575.g1	GE291950
61935906	CCAH1576.b1	GE291951
61935907	CCAH1576.g1	GE291952
61935908	CCAH1577.g1	GE291953
61935909	CCAH1579.b1	GE291954
61935910	CCAH1579.g1	GE291955
61935911	CCAH1580.b1	GE291956
61935912	CCAH1580.g1	GE291957
61935913	CCAH1581.b1	GE291958
61935914	CCAH1582.b1	GE291959
61935915	CCAH1582.g1	GE291960
61935916	CCAH1583.b1	GE291961
61935917	CCAH1583.g1	GE291962
61935918	CCAH1584.b1	GE291963
61935919	CCAH1584.g1	GE291964
61935920	CCAH1585.b1	GE291965
61935921	CCAH1585.g1	GE291966
61935922	CCAH1586.b1	GE291967
61935923	CCAH1586.g1	GE291968
61935924	CCAH1587.b1	GE291969
61935925	CCAH1587.g1	GE291970
61935926	CCAH1588.b1	GE291971
61935927	CCAH1588.g1	GE291972
61935928	CCAH1589.b1	GE291973
61935929	CCAH1589.g1	GE291974
61935930	CCAH1590.b1	GE291975
61935931	CCAH1590.g1	GE291976
61935932	CCAH1591.b1	GE291977
61935933	CCAH1591.g1	GE291978

61935934	CCAH1592.b1	GE291979
61935935	CCAH1592.g1	GE291980
61935936	CCAH1593.g1	GE291981
61935937	CCAH1594.b1	GE291982
61935938	CCAH1595.b1	GE291983
61935939	CCAH1595.g1	GE291984
61935940	CCAH1596.b1	GE291985
61935941	CCAH1596.g1	GE291986
61935942	CCAH1597.b1	GE291987
61935943	CCAH1598.g1	GE291988
61935944	CCAH1600.b1	GE291989
61935945	CCAH1600.g1	GE291990
61935946	CCAH1601.b1	GE291991
61935947	CCAH1601.g1	GE291992
61935948	CCAH1602.b1	GE291993
61935949	CCAH1602.g1	GE291994
61935950	CCAH1603.b1	GE291995
61935951	CCAH1603.g1	GE291996
61935952	CCAH1604.b1	GE291997
61935953	CCAH1604.g1	GE291998
61935954	CCAH1605.b1	GE291999
61935955	CCAH1605.g1	GE292000
61935956	CCAH1607.b1	GE292001
61935957	CCAH1607.g1	GE292002
61935958	CCAH1609.g1	GE292003
61935959	CCAH1610.b1	GE292004
61935960	CCAH1610.g1	GE292005
61935961	CCAH1611.b1	GE292006
61935962	CCAH1611.g1	GE292007
61935963	CCAH1612.b1	GE292008
61935964	CCAH1612.g1	GE292009
61935965	CCAH1613.b1	GE292010
61935966	CCAH1613.g1	GE292011
61935967	CCAH1614.b1	GE292012
61935968	CCAH1614.g1	GE292013
61935969	CCAH1615.b1	GE292014
61935970	CCAH1615.g1	GE292015
61935971	CCAH1616.b1	GE292016
61935972	CCAH1617.b1	GE292017
61935973	CCAH1617.g1	GE292018
61935974	CCAH1618.b1	GE292019
61935975	CCAH1618.g1	GE292020
61935976	CCAH1619.b1	GE292021
61935977	CCAH1619.g1	GE292022
61935978	CCAH1620.b1	GE292023
61935979	CCAH1620.g1	GE292024
61935980	CCAH1621.b1	GE292025
61935981	CCAH1621.g1	GE292026
61935982	CCAH1622.g1	GE292027
61935983	CCAH1623.b1	GE292028
61935984	CCAH1623.g1	GE292029
61935985	CCAH1624.b1	GE292030
61935986	CCAH1624.g1	GE292031
61935987	CCAH1625.b1	GE292032
61935988	CCAH1625.g1	GE292033
61935989	CCAH1626.b1	GE292034
61935990	CCAH1626.g1	GE292035
61935991	CCAH1627.b1	GE292036
61935992	CCAH1627.g1	GE292037
61935993	CCAH1628.b1	GE292038
61935994	CCAH1628.g1	GE292039
61935995	CCAH1629.b1	GE292040
61935996	CCAH1629.g1	GE292041

61935997	CCAH1630.b1	GE292042
61935998	CCAH1630.g1	GE292043
61935999	CCAH1631.b1	GE292044
61936000	CCAH1631.g1	GE292045
61936001	CCAH1632.b1	GE292046
61936002	CCAH1633.b1	GE292047
61936003	CCAH1633.g1	GE292048
61936004	CCAH1634.b1	GE292049
61936005	CCAH1634.g1	GE292050
61936006	CCAH1635.b1	GE292051
61936007	CCAH1635.g1	GE292052
61936008	CCAH1636.b1	GE292053
61936009	CCAH1636.g1	GE292054
61936010	CCAH1637.b1	GE292055
61936011	CCAH1637.g1	GE292056
61936012	CCAH1638.b1	GE292057
61936013	CCAH1638.g1	GE292058
61936014	CCAH1641.b1	GE292059
61936015	CCAH1641.g1	GE292060
61936016	CCAH1642.b1	GE292061
61936017	CCAH1642.g1	GE292062
61936018	CCAH1643.b1	GE292063
61936019	CCAH1643.g1	GE292064
61936020	CCAH1644.b1	GE292065
61936021	CCAH1644.g1	GE292066
61936022	CCAH1645.b1	GE292067
61936023	CCAH1645.g1	GE292068
61936024	CCAH1646.b1	GE292069
61936025	CCAH1646.g1	GE292070
61936026	CCAH1647.b1	GE292071
61936027	CCAH1648.b1	GE292072
61936028	CCAH1648.g1	GE292073
61936029	CCAH1649.b1	GE292074
61936030	CCAH1649.g1	GE292075
61936031	CCAH1650.b1	GE292076
61936032	CCAH1650.g1	GE292077
61936033	CCAH1651.b1	GE292078
61936034	CCAH1651.g1	GE292079
61936035	CCAH1652.b1	GE292080
61936036	CCAH1653.b1	GE292081
61936037	CCAH1653.g1	GE292082
61936038	CCAH1654.b1	GE292083
61936039	CCAH1654.g1	GE292084
61936040	CCAH1655.b1	GE292085
61936041	CCAH1655.g1	GE292086
61936042	CCAH1656.b1	GE292087
61936043	CCAH1656.g1	GE292088
61936044	CCAH1657.b1	GE292089
61936045	CCAH1657.g1	GE292090
61936046	CCAH1658.b1	GE292091
61936047	CCAH1658.g1	GE292092
61936048	CCAH1659.b1	GE292093
61936049	CCAH1659.g1	GE292094
61936050	CCAH1660.b1	GE292095
61936051	CCAH1660.g1	GE292096
61936052	CCAH1661.b1	GE292097
61936053	CCAH1661.g1	GE292098
61936054	CCAH1662.b1	GE292099
61936055	CCAH1662.g1	GE292100
61936056	CCAH1663.b1	GE292101
61936057	CCAH1663.g1	GE292102
61936058	CCAH1664.b1	GE292103
61936059	CCAH1664.g1	GE292104

61936060	CCAH1665.b1	GE292105
61936061	CCAH1665.g1	GE292106
61936062	CCAH1666.b1	GE292107
61936063	CCAH1666.g1	GE292108
61936064	CCAH1667.b1	GE292109
61936065	CCAH1667.g1	GE292110
61936066	CCAH1668.g1	GE292111
61936067	CCAH1669.b1	GE292112
61936068	CCAH1670.b1	GE292113
61936069	CCAH1670.g1	GE292114
61936070	CCAH1671.b1	GE292115
61936071	CCAH1671.g1	GE292116
61936072	CCAH1673.b1	GE292117
61936073	CCAH1673.g1	GE292118
61936074	CCAH1674.b1	GE292119
61936075	CCAH1674.g1	GE292120
61936076	CCAH1675.b1	GE292121
61936077	CCAH1675.g1	GE292122
61936078	CCAH1676.b1	GE292123
61936079	CCAH1676.g1	GE292124
61936080	CCAH1677.b1	GE292125
61936081	CCAH1677.g1	GE292126
61936082	CCAH1678.b1	GE292127
61936083	CCAH1678.g1	GE292128
61936084	CCAH1679.b1	GE292129
61936085	CCAH1679.g1	GE292130
61936086	CCAH1680.b1	GE292131
61936087	CCAH1680.g1	GE292132
61936088	CCAH1681.b1	GE292133
61936089	CCAH1681.g1	GE292134
61936090	CCAH1683.b1	GE292135
61936091	CCAH1683.g1	GE292136
61936092	CCAH1687.b1	GE292137
61936093	CCAH1687.g1	GE292138
61936094	CCAH1688.b1	GE292139
61936095	CCAH1688.g1	GE292140
61936096	CCAH1689.b1	GE292141
61936097	CCAH1689.g1	GE292142
61936098	CCAH1690.b1	GE292143
61936099	CCAH1690.g1	GE292144
61936100	CCAH1691.b1	GE292145
61936101	CCAH1691.g1	GE292146
61936102	CCAH1692.b1	GE292147
61936103	CCAH1694.b1	GE292148
61936104	CCAH1695.b1	GE292149
61936105	CCAH1695.g1	GE292150
61936106	CCAH1696.b1	GE292151
61936107	CCAH1697.b1	GE292152
61936108	CCAH1697.g1	GE292153
61936109	CCAH1698.b1	GE292154
61936110	CCAH1698.g1	GE292155
61936111	CCAH1699.b1	GE292156
61936112	CCAH1699.g1	GE292157
61936113	CCAH1700.b1	GE292158
61936114	CCAH1700.g1	GE292159
61936115	CCAH1701.b1	GE292160
61936116	CCAH1701.g1	GE292161
61936117	CCAH1702.g1	GE292162
61936118	CCAH1703.b1	GE292163
61936119	CCAH1703.g1	GE292164
61936120	CCAH1704.b1	GE292165
61936121	CCAH1704.g1	GE292166
61936122	CCAH1705.b1	GE292167

61936123	CCAH1705.g1	GE292168
61936124	CCAH1706.b1	GE292169
61936125	CCAH1706.g1	GE292170
61936126	CCAH1707.b1	GE292171
61936127	CCAH1707.g1	GE292172
61936128	CCAH1708.b1	GE292173
61936129	CCAH1708.g1	GE292174
61936130	CCAH1709.b1	GE292175
61936131	CCAH1709.g1	GE292176
61936132	CCAH1710.g1	GE292177
61936133	CCAH1711.b1	GE292178
61936134	CCAH1711.g1	GE292179
61936135	CCAH1712.b1	GE292180
61936136	CCAH1712.g1	GE292181
61936137	CCAH1713.b1	GE292182
61936138	CCAH1713.g1	GE292183
61936139	CCAH1714.b1	GE292184
61936140	CCAH1714.g1	GE292185
61936141	CCAH1715.b1	GE292186
61936142	CCAH1715.g1	GE292187
61936143	CCAH1716.b1	GE292188
61936144	CCAH1716.g1	GE292189
61936145	CCAH1717.g1	GE292190
61936146	CCAH1718.b1	GE292191
61936147	CCAH1718.g1	GE292192
61936148	CCAH1719.b1	GE292193
61936149	CCAH1719.g1	GE292194
61936150	CCAH1721.b1	GE292195
61936151	CCAH1721.g1	GE292196
61936152	CCAH1722.b1	GE292197
61936153	CCAH1722.g1	GE292198
61936154	CCAH1723.b1	GE292199
61936155	CCAH1723.g1	GE292200
61936156	CCAH1724.b1	GE292201
61936157	CCAH1725.b1	GE292202
61936158	CCAH1725.g1	GE292203
61936159	CCAH1726.b1	GE292204
61936160	CCAH1726.g1	GE292205
61936161	CCAH1727.b1	GE292206
61936162	CCAH1728.b1	GE292207
61936163	CCAH1728.g1	GE292208
61936164	CCAH1729.b1	GE292209
61936165	CCAH1729.g1	GE292210
61936166	CCAH1730.b1	GE292211
61936167	CCAH1730.g1	GE292212
61936168	CCAH1731.g1	GE292213
61936169	CCAH1732.b1	GE292214
61936170	CCAH1732.g1	GE292215
61936171	CCAH1733.b1	GE292216
61936172	CCAH1734.b1	GE292217
61936173	CCAH1734.g1	GE292218
61936174	CCAH1735.b1	GE292219
61936175	CCAH1735.g1	GE292220
61936176	CCAH1736.b1	GE292221
61936177	CCAH1736.g1	GE292222
61936178	CCAH1737.b1	GE292223
61936179	CCAH1737.g1	GE292224
61936180	CCAH1738.b1	GE292225
61936181	CCAH1738.g1	GE292226
61936182	CCAH1739.b1	GE292227
61936183	CCAH1739.g1	GE292228
61936184	CCAH1740.b1	GE292229
61936185	CCAH1740.g1	GE292230

61936186	CCAH1741.b1	GE292231
61936187	CCAH1741.g1	GE292232
61936188	CCAH1742.b1	GE292233
61936189	CCAH1742.g1	GE292234
61936190	CCAH1743.b1	GE292235
61936191	CCAH1744.b1	GE292236
61936192	CCAH1744.g1	GE292237
61936193	CCAH1745.b1	GE292238
61936194	CCAH1746.b1	GE292239
61936195	CCAH1746.g1	GE292240
61936196	CCAH1747.b1	GE292241
61936197	CCAH1747.g1	GE292242
61936198	CCAH1748.b1	GE292243
61936199	CCAH1748.g1	GE292244
61936200	CCAH1749.b1	GE292245
61936201	CCAH1749.g1	GE292246
61936202	CCAH1750.b1	GE292247
61936203	CCAH1750.g1	GE292248
61936204	CCAH1752.b1	GE292249
61936205	CCAH1752.g1	GE292250
61936206	CCAH1753.b1	GE292251
61936207	CCAH1753.g1	GE292252
61936208	CCAH1754.b1	GE292253
61936209	CCAH1754.g1	GE292254
61936210	CCAH1755.b1	GE292255
61936211	CCAH1755.g1	GE292256
61936212	CCAH1756.b1	GE292257
61936213	CCAH1756.g1	GE292258
61936214	CCAH1757.b1	GE292259
61936215	CCAH1757.g1	GE292260
61936216	CCAH1758.b1	GE292261
61936217	CCAH1758.g1	GE292262
61936218	CCAH1759.b1	GE292263
61936219	CCAH1759.g1	GE292264
61936220	CCAH1760.b1	GE292265
61936221	CCAH1760.g1	GE292266
61936222	CCAH1761.b1	GE292267
61936223	CCAH1761.g1	GE292268
61936224	CCAH1762.b1	GE292269
61936225	CCAH1762.g1	GE292270
61936226	CCAH1763.b1	GE292271
61936227	CCAH1763.g1	GE292272
61936228	CCAH1764.b1	GE292273
61936229	CCAH1764.g1	GE292274
61936230	CCAH1765.b1	GE292275
61936231	CCAH1765.g1	GE292276
61936232	CCAH1766.b1	GE292277
61936233	CCAH1766.g1	GE292278
61936234	CCAH1767.b1	GE292279
61936235	CCAH1768.b1	GE292280
61936236	CCAH1768.g1	GE292281
61936237	CCAH1769.b1	GE292282
61936238	CCAH1769.g1	GE292283
61936239	CCAH1770.b1	GE292284
61936240	CCAH1770.g1	GE292285
61936241	CCAH1772.b1	GE292286
61936242	CCAH1772.g1	GE292287
61936243	CCAH1773.b1	GE292288
61936244	CCAH1773.g1	GE292289
61936245	CCAH1774.b1	GE292290
61936246	CCAH1774.g1	GE292291
61936247	CCAH1775.b1	GE292292
61936248	CCAH1775.g1	GE292293

61936249	CCAH1776.b1	GE292294
61936250	CCAH1776.g1	GE292295
61936251	CCAH1777.b1	GE292296
61936252	CCAH1777.g1	GE292297
61936253	CCAH1778.b1	GE292298
61936254	CCAH1778.g1	GE292299
61936255	CCAH1779.b1	GE292300
61936256	CCAH1779.g1	GE292301
61936257	CCAH1780.b1	GE292302
61936258	CCAH1780.g1	GE292303
61936259	CCAH1781.b1	GE292304
61936260	CCAH1781.g1	GE292305
61936261	CCAH1782.b1	GE292306
61936262	CCAH1782.g1	GE292307
61936263	CCAH1783.b1	GE292308
61936264	CCAH1784.b1	GE292309
61936265	CCAH1784.g1	GE292310
61936266	CCAH1785.b1	GE292311
61936267	CCAH1785.g1	GE292312
61936268	CCAH1786.g1	GE292313
61936269	CCAH1787.g1	GE292314
61936270	CCAH1788.b1	GE292315
61936271	CCAH1788.g1	GE292316
61936272	CCAH1789.b1	GE292317
61936273	CCAH1789.g1	GE292318
61936274	CCAH1790.b1	GE292319
61936275	CCAH1790.g1	GE292320
61936276	CCAH1791.b1	GE292321
61936277	CCAH1791.g1	GE292322
61936278	CCAH1792.b1	GE292323
61936279	CCAH1792.g1	GE292324
61936280	CCAH1793.b1	GE292325
61936281	CCAH1793.g1	GE292326
61936282	CCAH1794.b1	GE292327
61936283	CCAH1794.g1	GE292328
61936284	CCAH1795.b1	GE292329
61936285	CCAH1795.g1	GE292330
61936286	CCAH1797.b1	GE292331
61936287	CCAH1797.g1	GE292332
61936288	CCAH1798.b1	GE292333
61936289	CCAH1798.g1	GE292334
61936290	CCAH1799.b1	GE292335
61936291	CCAH1799.g1	GE292336
61936292	CCAH1800.b1	GE292337
61936293	CCAH1800.g1	GE292338
61936294	CCAH1801.b1	GE292339
61936295	CCAH1801.g1	GE292340
61936296	CCAH1802.b1	GE292341
61936297	CCAH1802.g1	GE292342
61936298	CCAH1803.b1	GE292343
61936299	CCAH1803.g1	GE292344
61936300	CCAH1804.b1	GE292345
61936301	CCAH1804.g1	GE292346
61936302	CCAH1806.b1	GE292347
61936303	CCAH1806.g1	GE292348
61936304	CCAH1807.b1	GE292349
61936305	CCAH1807.g1	GE292350
61936306	CCAH1808.b1	GE292351
61936307	CCAH1808.g1	GE292352
61936308	CCAH1809.b1	GE292353
61936309	CCAH1809.g1	GE292354
61936310	CCAH1810.b1	GE292355
61936311	CCAH1810.g1	GE292356

61936312	CCAH1811.b1	GE292357
61936313	CCAH1811.g1	GE292358
61936314	CCAH1812.b1	GE292359
61936315	CCAH1812.g1	GE292360
61936316	CCAH1813.b1	GE292361
61936317	CCAH1813.g1	GE292362
61936318	CCAH1815.b1	GE292363
61936319	CCAH1816.b1	GE292364
61936320	CCAH1816.g1	GE292365
61936321	CCAH1817.b1	GE292366
61936322	CCAH1817.g1	GE292367
61936323	CCAH1819.b1	GE292368
61936324	CCAH1819.g1	GE292369
61936325	CCAH1820.g1	GE292370
61936326	CCAH1821.b1	GE292371
61936327	CCAH1821.g1	GE292372
61936328	CCAH1823.b1	GE292373
61936329	CCAH1823.g1	GE292374
61936330	CCAH1825.b1	GE292375
61936331	CCAH1825.g1	GE292376
61936332	CCAH1826.b1	GE292377
61936333	CCAH1827.b1	GE292378
61936334	CCAH1828.b1	GE292379
61936335	CCAH1828.g1	GE292380
61936336	CCAH1830.b1	GE292381
61936337	CCAH1831.b1	GE292382
61936338	CCAH1831.g1	GE292383
61936339	CCAH1833.b1	GE292384
61936340	CCAH1833.g1	GE292385
61936341	CCAH1834.b1	GE292386
61936342	CCAH1834.g1	GE292387
61936343	CCAH1835.b1	GE292388
61936344	CCAH1835.g1	GE292389
61936345	CCAH1836.b1	GE292390
61936346	CCAH1836.g1	GE292391
61936347	CCAH1837.b1	GE292392
61936348	CCAH1837.g1	GE292393
61936349	CCAH1838.b1	GE292394
61936350	CCAH1838.g1	GE292395
61936351	CCAH1839.b1	GE292396
61936352	CCAH1839.g1	GE292397
61936353	CCAH1840.b1	GE292398
61936354	CCAH1841.b1	GE292399
61936355	CCAH1841.g1	GE292400
61936356	CCAH1842.b1	GE292401
61936357	CCAH1842.g1	GE292402
61936358	CCAH1843.b1	GE292403
61936359	CCAH1843.g1	GE292404
61936360	CCAH1844.b1	GE292405
61936361	CCAH1845.b1	GE292406
61936362	CCAH1845.g1	GE292407
61936363	CCAH1846.b1	GE292408
61936364	CCAH1846.g1	GE292409
61936365	CCAH1848.b1	GE292410
61936366	CCAH1849.b1	GE292411
61936367	CCAH1849.g1	GE292412
61936368	CCAH1850.b1	GE292413
61936369	CCAH1850.g1	GE292414
61936370	CCAH1851.b1	GE292415
61936371	CCAH1851.g1	GE292416
61936372	CCAH1852.b1	GE292417
61936373	CCAH1852.g1	GE292418
61936374	CCAH1853.b1	GE292419

61936375	CCAH1853.g1	GE292420
61936376	CCAH1854.b1	GE292421
61936377	CCAH1854.g1	GE292422
61936378	CCAH1855.b1	GE292423
61936379	CCAH1855.g1	GE292424
61936380	CCAH1856.b1	GE292425
61936381	CCAH1856.g1	GE292426
61936382	CCAH1857.b1	GE292427
61936383	CCAH1857.g1	GE292428
61936384	CCAH1858.b1	GE292429
61936385	CCAH1858.g1	GE292430
61936386	CCAH1859.b1	GE292431
61936387	CCAH1859.g1	GE292432
61936388	CCAH1860.b1	GE292433
61936389	CCAH1860.g1	GE292434
61936390	CCAH1861.b1	GE292435
61936391	CCAH1861.g1	GE292436
61936392	CCAH1862.b1	GE292437
61936393	CCAH1862.g1	GE292438
61936394	CCAH1863.b1	GE292439
61936395	CCAH1865.b1	GE292440
61936396	CCAH1865.g1	GE292441
61936397	CCAH1866.b1	GE292442
61936398	CCAH1866.g1	GE292443
61936399	CCAH1867.b1	GE292444
61936400	CCAH1867.g1	GE292445
61936401	CCAH1868.b1	GE292446
61936402	CCAH1868.g1	GE292447
61936403	CCAH1869.b1	GE292448
61936404	CCAH1869.g1	GE292449
61936405	CCAH1870.b1	GE292450
61936406	CCAH1870.g1	GE292451
61936407	CCAH1871.b1	GE292452
61936408	CCAH1871.g1	GE292453
61936409	CCAH1872.b1	GE292454
61936410	CCAH1872.g1	GE292455
61936411	CCAH1873.b1	GE292456
61936412	CCAH1873.g1	GE292457
61936413	CCAH1874.b1	GE292458
61936414	CCAH1874.g1	GE292459
61936415	CCAH1875.b1	GE292460
61936416	CCAH1875.g1	GE292461
61936417	CCAH1876.b1	GE292462
61936418	CCAH1876.g1	GE292463
61936419	CCAH1877.g1	GE292464
61936420	CCAH1878.b1	GE292465
61936421	CCAH1878.g1	GE292466
61936422	CCAH1879.b1	GE292467
61936423	CCAH1879.g1	GE292468
61936424	CCAH1880.b1	GE292469
61936425	CCAH1881.g1	GE292470
61936426	CCAH1882.g1	GE292471
61936427	CCAH1883.b1	GE292472
61936428	CCAH1884.b1	GE292473
61936429	CCAH1884.g1	GE292474
61936430	CCAH1886.g1	GE292475
61936431	CCAH1887.b1	GE292476
61936432	CCAH1887.g1	GE292477
61936433	CCAH1888.b1	GE292478
61936434	CCAH1888.g1	GE292479
61936435	CCAH1889.b1	GE292480
61936436	CCAH1889.g1	GE292481
61936437	CCAH1890.b1	GE292482

61936438	CCAH1890.g1	GE292483
61936439	CCAH1891.b1	GE292484
61936440	CCAH1891.g1	GE292485
61936441	CCAH1892.b1	GE292486
61936442	CCAH1892.g1	GE292487
61936443	CCAH1893.b1	GE292488
61936444	CCAH1893.g1	GE292489
61936445	CCAH1894.b1	GE292490
61936446	CCAH1894.g1	GE292491
61936447	CCAH1895.b1	GE292492
61936448	CCAH1895.g1	GE292493
61936449	CCAH1896.b1	GE292494
61936450	CCAH1896.g1	GE292495
61936451	CCAH1897.b1	GE292496
61936452	CCAH1897.g1	GE292497
61936453	CCAH1899.g1	GE292498
61936454	CCAH1900.b1	GE292499
61936455	CCAH1900.g1	GE292500
61936456	CCAH1901.b1	GE292501
61936457	CCAH1901.g1	GE292502
61936458	CCAH1902.b1	GE292503
61936459	CCAH1902.g1	GE292504
61936460	CCAH1903.b1	GE292505
61936461	CCAH1903.g1	GE292506
61936462	CCAH1904.b1	GE292507
61936463	CCAH1904.g1	GE292508
61936464	CCAH1905.b1	GE292509
61936465	CCAH1905.g1	GE292510
61936466	CCAH1906.b1	GE292511
61936467	CCAH1906.g1	GE292512
61936468	CCAH1907.b1	GE292513
61936469	CCAH1907.g1	GE292514
61936470	CCAH1908.b1	GE292515
61936471	CCAH1909.b1	GE292516
61936472	CCAH1909.g1	GE292517
61936473	CCAH1910.b1	GE292518
61936474	CCAH1910.g1	GE292519
61936475	CCAH1912.g1	GE292520
61936476	CCAH1913.b1	GE292521
61936477	CCAH1913.g1	GE292522
61936478	CCAH1914.b1	GE292523
61936479	CCAH1914.g1	GE292524
61936480	CCAH1915.b1	GE292525
61936481	CCAH1915.g1	GE292526
61936482	CCAH1916.b1	GE292527
61936483	CCAH1916.g1	GE292528
61936484	CCAH1917.b1	GE292529
61936485	CCAH1917.g1	GE292530
61936486	CCAH1918.b1	GE292531
61936487	CCAH1918.g1	GE292532
61936488	CCAH1921.b1	GE292533
61936489	CCAH1921.g1	GE292534
61936490	CCAH1922.b1	GE292535
61936491	CCAH1922.g1	GE292536
61936492	CCAH1923.b1	GE292537
61936493	CCAH1923.g1	GE292538
61936494	CCAH1924.b1	GE292539
61936495	CCAH1925.b1	GE292540
61936496	CCAH1925.g1	GE292541
61936497	CCAH1926.b1	GE292542
61936498	CCAH1926.g1	GE292543
61936499	CCAH1927.b1	GE292544
61936500	CCAH1927.g1	GE292545

61936501	CCAH1928.b1	GE292546
61936502	CCAH1928.g1	GE292547
61936503	CCAH1929.b1	GE292548
61936504	CCAH1929.g1	GE292549
61936505	CCAH1931.b1	GE292550
61936506	CCAH1932.b1	GE292551
61936507	CCAH1932.g1	GE292552
61936508	CCAH1933.b1	GE292553
61936509	CCAH1933.g1	GE292554
61936510	CCAH1934.b1	GE292555
61936511	CCAH1934.g1	GE292556
61936512	CCAH1935.b1	GE292557
61936513	CCAH1935.g1	GE292558
61936514	CCAH1936.g1	GE292559
61936515	CCAH1937.b1	GE292560
61936516	CCAH1937.g1	GE292561
61936517	CCAH1938.b1	GE292562
61936518	CCAH1938.g1	GE292563
61936519	CCAH1939.b1	GE292564
61936520	CCAH1939.g1	GE292565
61936521	CCAH1940.b1	GE292566
61936522	CCAH1940.g1	GE292567
61936523	CCAH1941.b1	GE292568
61936524	CCAH1942.b1	GE292569
61936525	CCAH1943.b1	GE292570
61936526	CCAH1943.g1	GE292571
61936527	CCAH1944.b1	GE292572
61936528	CCAH1944.g1	GE292573
61936529	CCAH1950.b1	GE292574
61936530	CCAH1950.g1	GE292575
61936531	CCAH1951.b1	GE292576
61936532	CCAH1951.g1	GE292577
61936533	CCAH1952.b1	GE292578
61936534	CCAH1952.g1	GE292579
61936535	CCAH1953.b1	GE292580
61936536	CCAH1953.g1	GE292581
61936537	CCAH1954.b1	GE292582
61936538	CCAH1954.g1	GE292583
61936539	CCAH1955.b1	GE292584
61936540	CCAH1955.g1	GE292585
61936541	CCAH1956.b1	GE292586
61936542	CCAH1956.g1	GE292587
61936543	CCAH1957.b1	GE292588
61936544	CCAH1957.g1	GE292589
61936545	CCAH1958.b1	GE292590
61936546	CCAH1958.g1	GE292591
61936547	CCAH1959.b1	GE292592
61936548	CCAH1959.g1	GE292593
61936549	CCAH1961.b1	GE292594
61936550	CCAH1962.b1	GE292595
61936551	CCAH1963.b1	GE292596
61936552	CCAH1963.g1	GE292597
61936553	CCAH1965.b1	GE292598
61936554	CCAH1965.g1	GE292599
61936555	CCAH1966.b1	GE292600
61936556	CCAH1966.g1	GE292601
61936557	CCAH1967.b1	GE292602
61936558	CCAH1967.g1	GE292603
61936559	CCAH1968.b1	GE292604
61936560	CCAH1968.g1	GE292605
61936561	CCAH1969.b1	GE292606
61936562	CCAH1969.g1	GE292607
61936563	CCAH1970.b1	GE292608

61936564	CCAH1970.g1	GE292609
61936565	CCAH1971.b1	GE292610
61936566	CCAH1971.g1	GE292611
61936567	CCAH1972.b1	GE292612
61936568	CCAH1972.g1	GE292613
61936569	CCAH1973.b1	GE292614
61936570	CCAH1973.g1	GE292615
61936571	CCAH1974.b1	GE292616
61936572	CCAH1975.b1	GE292617
61936573	CCAH1975.g1	GE292618
61936574	CCAH1976.g1	GE292619
61936575	CCAH1977.b1	GE292620
61936576	CCAH1978.b1	GE292621
61936577	CCAH1978.g1	GE292622
61936578	CCAH1979.b1	GE292623
61936579	CCAH1979.g1	GE292624
61936580	CCAH1981.b1	GE292625
61936581	CCAH1981.g1	GE292626
61936582	CCAH1982.b1	GE292627
61936583	CCAH1982.g1	GE292628
61936584	CCAH1986.b1	GE292629
61936585	CCAH1986.g1	GE292630
61936586	CCAH1987.b1	GE292631
61936587	CCAH1987.g1	GE292632
61936588	CCAH1988.b1	GE292633
61936589	CCAH1989.b1	GE292634
61936590	CCAH1989.g1	GE292635
61936591	CCAH1991.b1	GE292636
61936592	CCAH1991.g1	GE292637
61936593	CCAH1992.b1	GE292638
61936594	CCAH1992.g1	GE292639
61936595	CCAH1993.b1	GE292640
61936596	CCAH1993.g1	GE292641
61936597	CCAH1994.b1	GE292642
61936598	CCAH1994.g1	GE292643
61936599	CCAH1995.b1	GE292644
61936600	CCAH1995.g1	GE292645
61936601	CCAH1996.b1	GE292646
61936602	CCAH1996.g1	GE292647
61936603	CCAH1997.b1	GE292648
61936604	CCAH1997.g1	GE292649
61936605	CCAH1998.b1	GE292650
61936606	CCAH1998.g1	GE292651
61936607	CCAH1999.b1	GE292652
61936608	CCAH1999.g1	GE292653
61936609	CCAH2000.b1	GE292654
61936610	CCAH2000.g1	GE292655
61936611	CCAH2001.b1	GE292656
61936612	CCAH2001.g1	GE292657
61936613	CCAH2002.b1	GE292658
61936614	CCAH2002.g1	GE292659
61936615	CCAH2003.g1	GE292660
61936616	CCAH2004.b1	GE292661
61936617	CCAH2004.g1	GE292662
61936618	CCAH2005.b1	GE292663
61936619	CCAH2005.g1	GE292664
61936620	CCAH2006.b1	GE292665
61936621	CCAH2006.g1	GE292666
61936622	CCAH2007.b1	GE292667
61936623	CCAH2007.g1	GE292668
61936624	CCAH2008.b1	GE292669
61936625	CCAH2008.g1	GE292670
61936626	CCAH2009.b1	GE292671

61936627	CCAH2011.b1	GE292672
61936628	CCAH2011.g1	GE292673
61936629	CCAH2012.b1	GE292674
61936630	CCAH2012.g1	GE292675
61936631	CCAH2013.b1	GE292676
61936632	CCAH2015.b1	GE292677
61936633	CCAH2015.g1	GE292678
61936634	CCAH2018.b1	GE292679
61936635	CCAH2018.g1	GE292680
61936636	CCAH2019.b1	GE292681
61936637	CCAH2019.g1	GE292682
61936638	CCAH2021.b1	GE292683
61936639	CCAH2021.g1	GE292684
61936640	CCAH2023.b1	GE292685
61936641	CCAH2023.g1	GE292686
61936642	CCAH2026.b1	GE292687
61936643	CCAH2026.g1	GE292688
61936644	CCAH2027.b1	GE292689
61936645	CCAH2027.g1	GE292690
61936646	CCAH2028.b1	GE292691
61936647	CCAH2029.b1	GE292692
61936648	CCAH2029.g1	GE292693
61936649	CCAH2030.b1	GE292694
61936650	CCAH2030.g1	GE292695
61936651	CCAH2031.b1	GE292696
61936652	CCAH2031.g1	GE292697
61936653	CCAH2032.b1	GE292698
61936654	CCAH2032.g1	GE292699
61936655	CCAH2033.b1	GE292700
61936656	CCAH2033.g1	GE292701
61936657	CCAH2034.b1	GE292702
61936658	CCAH2034.g1	GE292703
61936659	CCAH2035.b1	GE292704
61936660	CCAH2035.g1	GE292705
61936661	CCAH2036.b1	GE292706
61936662	CCAH2036.g1	GE292707
61936663	CCAH2037.b1	GE292708
61936664	CCAH2037.g1	GE292709
61936665	CCAH2038.b1	GE292710
61936666	CCAH2041.b1	GE292711
61936667	CCAH2041.g1	GE292712
61936668	CCAH2042.b1	GE292713
61936669	CCAH2042.g1	GE292714
61936670	CCAH2043.b1	GE292715
61936671	CCAH2043.g1	GE292716
61936672	CCAH2044.b1	GE292717
61936673	CCAH2044.g1	GE292718
61936674	CCAH2046.b1	GE292719
61936675	CCAH2046.g1	GE292720
61936676	CCAH2047.b1	GE292721
61936677	CCAH2047.g1	GE292722
61936678	CCAH2048.b1	GE292723
61936679	CCAH2048.g1	GE292724
61936680	CCAH2049.b1	GE292725
61936681	CCAH2050.b1	GE292726
61936682	CCAH2050.g1	GE292727
61936683	CCAH2051.b1	GE292728
61936684	CCAH2053.b1	GE292729
61936685	CCAH2053.g1	GE292730
61936686	CCAH2054.b1	GE292731
61936687	CCAH2054.g1	GE292732
61936688	CCAH2055.b1	GE292733
61936689	CCAH2055.g1	GE292734

61936690	CCAH2056.b1	GE292735
61936691	CCAH2056.g1	GE292736
61936692	CCAH2057.b1	GE292737
61936693	CCAH2057.g1	GE292738
61936694	CCAH2058.b1	GE292739
61936695	CCAH2058.g1	GE292740
61936696	CCAH2059.b1	GE292741
61936697	CCAH2059.g1	GE292742
61936698	CCAH2060.b1	GE292743
61936699	CCAH2060.g1	GE292744
61936700	CCAH2061.b1	GE292745
61936701	CCAH2061.g1	GE292746
61936702	CCAH2062.b1	GE292747
61936703	CCAH2062.g1	GE292748
61936704	CCAH2063.b1	GE292749
61936705	CCAH2063.g1	GE292750
61936706	CCAH2064.b1	GE292751
61936707	CCAH2064.g1	GE292752
61936708	CCAH2065.b1	GE292753
61936709	CCAH2065.g1	GE292754
61936710	CCAH2066.b1	GE292755
61936711	CCAH2066.g1	GE292756
61936712	CCAH2067.b1	GE292757
61936713	CCAH2067.g1	GE292758
61936714	CCAH2068.b1	GE292759
61936715	CCAH2068.g1	GE292760
61936716	CCAH2069.b1	GE292761
61936717	CCAH2069.g1	GE292762
61936718	CCAH2070.b1	GE292763
61936719	CCAH2070.g1	GE292764
61936720	CCAH2071.b1	GE292765
61936721	CCAH2072.b1	GE292766
61936722	CCAH2072.g1	GE292767
61936723	CCAH2073.b1	GE292768
61936724	CCAH2073.g1	GE292769
61936725	CCAH2074.b1	GE292770
61936726	CCAH2074.g1	GE292771
61936727	CCAH2076.b1	GE292772
61936728	CCAH2076.g1	GE292773
61936729	CCAH2077.b1	GE292774
61936730	CCAH2077.g1	GE292775
61936731	CCAH2078.b1	GE292776
61936732	CCAH2078.g1	GE292777
61936733	CCAH2079.b1	GE292778
61936734	CCAH2080.b1	GE292779
61936735	CCAH2081.b1	GE292780
61936736	CCAH2081.g1	GE292781
61936737	CCAH2082.b1	GE292782
61936738	CCAH2082.g1	GE292783
61936739	CCAH2083.b1	GE292784
61936740	CCAH2083.g1	GE292785
61936741	CCAH2084.b1	GE292786
61936742	CCAH2084.g1	GE292787
61936743	CCAH2085.g1	GE292788
61936744	CCAH2086.b1	GE292789
61936745	CCAH2086.g1	GE292790
61936746	CCAH2087.b1	GE292791
61936747	CCAH2087.g1	GE292792
61936748	CCAH2088.b1	GE292793
61936749	CCAH2088.g1	GE292794
61936750	CCAH2089.b1	GE292795
61936751	CCAH2089.g1	GE292796
61936752	CCAH2090.b1	GE292797

61936753	CCAH2090.g1	GE292798
61936754	CCAH2091.b1	GE292799
61936755	CCAH2091.g1	GE292800
61936756	CCAH2092.b1	GE292801
61936757	CCAH2092.g1	GE292802
61936758	CCAH2093.b1	GE292803
61936759	CCAH2093.g1	GE292804
61936760	CCAH2094.b1	GE292805
61936761	CCAH2095.b1	GE292806
61936762	CCAH2095.g1	GE292807
61936763	CCAH2096.g1	GE292808
61936764	CCAH2097.b1	GE292809
61936765	CCAH2097.g1	GE292810
61936766	CCAH2098.b1	GE292811
61936767	CCAH2098.g1	GE292812
61936768	CCAH2099.b1	GE292813
61936769	CCAH2099.g1	GE292814
61936770	CCAH2100.b1	GE292815
61936771	CCAH2100.g1	GE292816
61936772	CCAH2101.b1	GE292817
61936773	CCAH2101.g1	GE292818
61936774	CCAH2102.b1	GE292819
61936775	CCAH2102.g1	GE292820
61936776	CCAH2103.b1	GE292821
61936777	CCAH2103.g1	GE292822
61936778	CCAH2104.b1	GE292823
61936779	CCAH2104.g1	GE292824
61936780	CCAH2106.b1	GE292825
61936781	CCAH2106.g1	GE292826
61936782	CCAH2107.b1	GE292827
61936783	CCAH2107.g1	GE292828
61936784	CCAH2108.b1	GE292829
61936785	CCAH2108.g1	GE292830
61936786	CCAH2109.b1	GE292831
61936787	CCAH2109.g1	GE292832
61936788	CCAH2110.b1	GE292833
61936789	CCAH2110.g1	GE292834
61936790	CCAH2111.b1	GE292835
61936791	CCAH2112.g1	GE292836
61936792	CCAH2113.b1	GE292837
61936793	CCAH2113.g1	GE292838
61936794	CCAH2114.b1	GE292839
61936795	CCAH2114.g1	GE292840
61936796	CCAH2115.b1	GE292841
61936797	CCAH2116.b1	GE292842
61936798	CCAH2116.g1	GE292843
61936799	CCAH2117.b1	GE292844
61936800	CCAH2117.g1	GE292845
61936801	CCAH2118.b1	GE292846
61936802	CCAH2118.g1	GE292847
61936803	CCAH2119.b1	GE292848
61936804	CCAH2119.g1	GE292849
61936805	CCAH2120.b1	GE292850
61936806	CCAH2121.b1	GE292851
61936807	CCAH2121.g1	GE292852
61936808	CCAH2122.b1	GE292853
61936809	CCAH2122.g1	GE292854
61936810	CCAH2123.b1	GE292855
61936811	CCAH2123.g1	GE292856
61936812	CCAH2124.b1	GE292857
61936813	CCAH2124.g1	GE292858
61936814	CCAH2125.b1	GE292859
61936815	CCAH2125.g1	GE292860

61936816	CCAH2126.b1	GE292861
61936817	CCAH2126.g1	GE292862
61936818	CCAH2127.b1	GE292863
61936819	CCAH2127.g1	GE292864
61936820	CCAH2128.b1	GE292865
61936821	CCAH2128.g1	GE292866
61936822	CCAH2129.b1	GE292867
61936823	CCAH2129.g1	GE292868
61936824	CCAH2130.b1	GE292869
61936825	CCAH2130.g1	GE292870
61936826	CCAH2132.b1	GE292871
61936827	CCAH2132.g1	GE292872
61936828	CCAH2133.b1	GE292873
61936829	CCAH2134.b1	GE292874
61936830	CCAH2134.g1	GE292875
61936831	CCAH2135.b1	GE292876
61936832	CCAH2135.g1	GE292877
61936833	CCAH2137.b1	GE292878
61936834	CCAH2137.g1	GE292879
61936835	CCAH2138.b1	GE292880
61936836	CCAH2138.g1	GE292881
61936837	CCAH2139.b1	GE292882
61936838	CCAH2139.g1	GE292883
61936839	CCAH2140.b1	GE292884
61936840	CCAH2140.g1	GE292885
61936841	CCAH2142.b1	GE292886
61936842	CCAH2142.g1	GE292887
61936843	CCAH2143.b1	GE292888
61936844	CCAH2143.g1	GE292889
61936845	CCAH2144.b1	GE292890
61936846	CCAH2144.g1	GE292891
61936847	CCAH2145.b1	GE292892
61936848	CCAH2145.g1	GE292893
61936849	CCAH2146.b1	GE292894
61936850	CCAH2148.b1	GE292895
61936851	CCAH2149.g1	GE292896
61936852	CCAH2150.b1	GE292897
61936853	CCAH2151.b1	GE292898
61936854	CCAH2152.b1	GE292899
61936855	CCAH2152.g1	GE292900
61936856	CCAH2153.b1	GE292901
61936857	CCAH2153.g1	GE292902
61936858	CCAH2154.b1	GE292903
61936859	CCAH2154.g1	GE292904
61936860	CCAH2155.b1	GE292905
61936861	CCAH2155.g1	GE292906
61936862	CCAH2156.b1	GE292907
61936863	CCAH2156.g1	GE292908
61936864	CCAH2157.b1	GE292909
61936865	CCAH2157.g1	GE292910
61936866	CCAH2159.b1	GE292911
61936867	CCAH2160.b1	GE292912
61936868	CCAH2160.g1	GE292913
61936869	CCAH2161.b1	GE292914
61936870	CCAH2161.g1	GE292915
61936871	CCAH2162.b1	GE292916
61936872	CCAH2162.g1	GE292917
61936873	CCAH2163.b1	GE292918
61936874	CCAH2163.g1	GE292919
61936875	CCAH2164.b1	GE292920
61936876	CCAH2164.g1	GE292921
61936877	CCAH2165.b1	GE292922
61936878	CCAH2165.g1	GE292923

61936879	CCAH2167.b1	GE292924
61936880	CCAH2167.g1	GE292925
61936881	CCAH2168.b1	GE292926
61936882	CCAH2168.g1	GE292927
61936883	CCAH2169.b1	GE292928
61936884	CCAH2169.g1	GE292929
61936885	CCAH2170.b1	GE292930
61936886	CCAH2170.g1	GE292931
61936887	CCAH2171.g1	GE292932
61936888	CCAH2172.b1	GE292933
61936889	CCAH2172.g1	GE292934
61936890	CCAH2173.b1	GE292935
61936891	CCAH2173.g1	GE292936
61936892	CCAH2175.g1	GE292937
61936893	CCAH2176.b1	GE292938
61936894	CCAH2177.b1	GE292939
61936895	CCAH2177.g1	GE292940
61936896	CCAH2181.g1	GE292941
61936897	CCAH2182.b1	GE292942
61936898	CCAH2182.g1	GE292943
61936899	CCAH2183.b1	GE292944
61936900	CCAH2184.b1	GE292945
61936901	CCAH2184.g1	GE292946
61936902	CCAH2185.b1	GE292947
61936903	CCAH2185.g1	GE292948
61936904	CCAH2186.b1	GE292949
61936905	CCAH2186.g1	GE292950
61936906	CCAH2187.b1	GE292951
61936907	CCAH2187.g1	GE292952
61936908	CCAH2188.b1	GE292953
61936909	CCAH2188.g1	GE292954
61936910	CCAH2189.b1	GE292955
61936911	CCAH2189.g1	GE292956
61936912	CCAH2190.b1	GE292957
61936913	CCAH2190.g1	GE292958
61936914	CCAH2191.b1	GE292959
61936915	CCAH2191.g1	GE292960
61936916	CCAH2192.g1	GE292961
61936917	CCAH2193.b1	GE292962
61936918	CCAH2193.g1	GE292963
61936919	CCAH2195.b1	GE292964
61936920	CCAH2195.g1	GE292965
61936921	CCAH2196.b1	GE292966
61936922	CCAH2196.g1	GE292967
61936923	CCAH2197.b1	GE292968
61936924	CCAH2197.g1	GE292969
61936925	CCAH2198.b1	GE292970
61936926	CCAH2198.g1	GE292971
61936927	CCAH2199.b1	GE292972
61936928	CCAH2199.g1	GE292973
61936929	CCAH2201.b1	GE292974
61936930	CCAH2201.g1	GE292975
61936931	CCAH2202.b1	GE292976
61936932	CCAH2202.g1	GE292977
61936933	CCAH2203.b1	GE292978
61936934	CCAH2203.g1	GE292979
61936935	CCAH2205.b1	GE292980
61936936	CCAH2205.g1	GE292981
61936937	CCAH2206.b1	GE292982
61936938	CCAH2206.g1	GE292983
61936939	CCAH2207.b1	GE292984
61936940	CCAH2209.b1	GE292985
61936941	CCAH2209.g1	GE292986

61936942	CCAH2210.b1	GE292987
61936943	CCAH2210.g1	GE292988
61936944	CCAH2211.g1	GE292989
61936945	CCAH2212.b1	GE292990
61936946	CCAH2212.g1	GE292991
61936947	CCAH2213.b1	GE292992
61936948	CCAH2213.g1	GE292993
61936949	CCAH2216.b1	GE292994
61936950	CCAH2217.b1	GE292995
61936951	CCAH2217.g1	GE292996
61936952	CCAH2218.b1	GE292997
61936953	CCAH2218.g1	GE292998
61936954	CCAH2219.b1	GE292999
61936955	CCAH2220.b1	GE293000
61936956	CCAH2220.g1	GE293001
61936957	CCAH2221.b1	GE293002
61936958	CCAH2221.g1	GE293003
61936959	CCAH2222.b1	GE293004
61936960	CCAH2222.g1	GE293005
61936961	CCAH2223.b1	GE293006
61936962	CCAH2223.g1	GE293007
61936963	CCAH2225.b1	GE293008
61936964	CCAH2225.g1	GE293009
61936965	CCAH2226.b1	GE293010
61936966	CCAH2227.b1	GE293011
61936967	CCAH2228.b1	GE293012
61936968	CCAH2228.g1	GE293013
61936969	CCAH2229.b1	GE293014
61936970	CCAH2229.g1	GE293015
61936971	CCAH2230.b1	GE293016
61936972	CCAH2230.g1	GE293017
61936973	CCAH2231.b1	GE293018
61936974	CCAH2231.g1	GE293019
61936975	CCAH2232.b1	GE293020
61936976	CCAH2232.g1	GE293021
61936977	CCAH2233.b1	GE293022
61936978	CCAH2233.g1	GE293023
61936979	CCAH2234.b1	GE293024
61936980	CCAH2234.g1	GE293025
61936981	CCAH2235.b1	GE293026
61936982	CCAH2236.b1	GE293027
61936983	CCAH2236.g1	GE293028
61936984	CCAH2238.b1	GE293029
61936985	CCAH2238.g1	GE293030
61936986	CCAH2239.b1	GE293031
61936987	CCAH2240.g1	GE293032
61936988	CCAH2241.b1	GE293033
61936989	CCAH2241.g1	GE293034
61936990	CCAH2242.g1	GE293035
61936991	CCAH2243.b1	GE293036
61936992	CCAH2243.g1	GE293037
61936993	CCAH2244.b1	GE293038
61936994	CCAH2244.g1	GE293039
61936995	CCAH2245.b1	GE293040
61936996	CCAH2246.b1	GE293041
61936997	CCAH2246.g1	GE293042
61936998	CCAH2247.b1	GE293043
61936999	CCAH2247.g1	GE293044
61937000	CCAH2248.b1	GE293045
61937001	CCAH2249.b1	GE293046
61937002	CCAH2249.g1	GE293047
61937003	CCAH2250.b1	GE293048
61937004	CCAH2250.g1	GE293049

61937005	CCAH2251.b1	GE293050
61937006	CCAH2251.g1	GE293051
61937007	CCAH2253.b1	GE293052
61937008	CCAH2253.g1	GE293053
61937009	CCAH2254.b1	GE293054
61937010	CCAH2254.g1	GE293055
61937011	CCAH2255.b1	GE293056
61937012	CCAH2255.g1	GE293057
61937013	CCAH2256.b1	GE293058
61937014	CCAH2256.g1	GE293059
61937015	CCAH2257.b1	GE293060
61937016	CCAH2258.b1	GE293061
61937017	CCAH2258.g1	GE293062
61937018	CCAH2259.g1	GE293063
61937019	CCAH2260.b1	GE293064
61937020	CCAH2260.g1	GE293065
61937021	CCAH2261.b1	GE293066
61937022	CCAH2261.g1	GE293067
61937023	CCAH2262.b1	GE293068
61937024	CCAH2262.g1	GE293069
61937025	CCAH2263.b1	GE293070
61937026	CCAH2264.b1	GE293071
61937027	CCAH2264.g1	GE293072
61937028	CCAH2266.b1	GE293073
61937029	CCAH2266.g1	GE293074
61937030	CCAH2267.b1	GE293075
61937031	CCAH2267.g1	GE293076
61937032	CCAH2268.b1	GE293077
61937033	CCAH2268.g1	GE293078
61937034	CCAH2269.b1	GE293079
61937035	CCAH2269.g1	GE293080
61937036	CCAH2270.b1	GE293081
61937037	CCAH2271.g1	GE293082
61937038	CCAH2273.b1	GE293083
61937039	CCAH2273.g1	GE293084
61937040	CCAH2274.b1	GE293085
61937041	CCAH2274.g1	GE293086
61937042	CCAH2275.g1	GE293087
61937043	CCAH2276.b1	GE293088
61937044	CCAH2276.g1	GE293089
61937045	CCAH2277.b1	GE293090
61937046	CCAH2277.g1	GE293091
61937047	CCAH2280.b1	GE293092
61937048	CCAH2280.g1	GE293093
61937049	CCAH2281.b1	GE293094
61937050	CCAH2281.g1	GE293095
61937051	CCAH2282.b1	GE293096
61937052	CCAH2282.g1	GE293097
61937053	CCAH2283.b1	GE293098
61937054	CCAH2283.g1	GE293099
61937055	CCAH2284.b1	GE293100
61937056	CCAH2284.g1	GE293101
61937057	CCAH2285.g1	GE293102
61937058	CCAH2287.b1	GE293103
61937059	CCAH2287.g1	GE293104
61937060	CCAH2288.b1	GE293105
61937061	CCAH2288.g1	GE293106
61937062	CCAH2289.b1	GE293107
61937063	CCAH2289.g1	GE293108
61937064	CCAH2290.b1	GE293109
61937065	CCAH2290.g1	GE293110
61937066	CCAH2291.b1	GE293111
61937067	CCAH2291.g1	GE293112

61937068	CCAH2292.b1	GE293113
61937069	CCAH2292.g1	GE293114
61937070	CCAH2293.b1	GE293115
61937071	CCAH2293.g1	GE293116
61937072	CCAH2294.b1	GE293117
61937073	CCAH2294.g1	GE293118
61937074	CCAH2295.b1	GE293119
61937075	CCAH2295.g1	GE293120
61937076	CCAH2296.b1	GE293121
61937077	CCAH2296.g1	GE293122
61937078	CCAH2299.b1	GE293123
61937079	CCAH2299.g1	GE293124
61937080	CCAH2300.b1	GE293125
61937081	CCAH2301.b1	GE293126
61937082	CCAH2301.g1	GE293127
61937083	CCAH2302.b1	GE293128
61937084	CCAH2302.g1	GE293129
61937085	CCAH2305.b1	GE293130
61937086	CCAH2305.g1	GE293131
61937087	CCAH2306.b1	GE293132
61937088	CCAH2306.g1	GE293133
61937089	CCAH2307.g1	GE293134
61937090	CCAH2308.b1	GE293135
61937091	CCAH2308.g1	GE293136
61937092	CCAH2310.b1	GE293137
61937093	CCAH2310.g1	GE293138
61937094	CCAH2311.b1	GE293139
61937095	CCAH2311.g1	GE293140
61937096	CCAH2312.b1	GE293141
61937097	CCAH2312.g1	GE293142
61937098	CCAH2313.b1	GE293143
61937099	CCAH2313.g1	GE293144
61937100	CCAH2314.b1	GE293145
61937101	CCAH2315.b1	GE293146
61937102	CCAH2315.g1	GE293147
61937103	CCAH2316.b1	GE293148
61937104	CCAH2317.b1	GE293149
61937105	CCAH2317.g1	GE293150
61937106	CCAH2318.b1	GE293151
61937107	CCAH2318.g1	GE293152
61937108	CCAH2319.b1	GE293153
61937109	CCAH2319.g1	GE293154
61937110	CCAH2320.b1	GE293155
61937111	CCAH2320.g1	GE293156
61937112	CCAH2321.b1	GE293157
61937113	CCAH2321.g1	GE293158
61937114	CCAH2322.b1	GE293159
61937115	CCAH2324.b1	GE293160
61937116	CCAH2325.b1	GE293161
61937117	CCAH2326.b1	GE293162
61937118	CCAH2326.g1	GE293163
61937119	CCAH2327.b1	GE293164
61937120	CCAH2327.g1	GE293165
61937121	CCAH2328.b1	GE293166
61937122	CCAH2328.g1	GE293167
61937123	CCAH2330.b1	GE293168
61937124	CCAH2330.g1	GE293169
61937125	CCAH2331.b1	GE293170
61937126	CCAH2331.g1	GE293171
61937127	CCAH2332.b1	GE293172
61937128	CCAH2332.g1	GE293173
61937129	CCAH2333.b1	GE293174
61937130	CCAH2333.g1	GE293175

61937131	CCAH2334.b1	GE293176
61937132	CCAH2334.g1	GE293177
61937133	CCAH2335.b1	GE293178
61937134	CCAH2335.g1	GE293179
61937135	CCAH2336.g1	GE293180
61937136	CCAH2337.b1	GE293181
61937137	CCAH2337.g1	GE293182
61937138	CCAH2338.b1	GE293183
61937139	CCAH2338.g1	GE293184
61937140	CCAH2339.b1	GE293185
61937141	CCAH2339.g1	GE293186
61937142	CCAH2340.b1	GE293187
61937143	CCAH2340.g1	GE293188
61937144	CCAH2341.b1	GE293189
61937145	CCAH2341.g1	GE293190
61937146	CCAH2342.b1	GE293191
61937147	CCAH2343.b1	GE293192
61937148	CCAH2343.g1	GE293193
61937149	CCAH2344.b1	GE293194
61937150	CCAH2344.g1	GE293195
61937151	CCAH2345.b1	GE293196
61937152	CCAH2345.g1	GE293197
61937153	CCAH2346.b1	GE293198
61937154	CCAH2346.g1	GE293199
61937155	CCAH2347.b1	GE293200
61937156	CCAH2347.g1	GE293201
61937157	CCAH2348.b1	GE293202
61937158	CCAH2348.g1	GE293203
61937159	CCAH2349.b1	GE293204
61937160	CCAH2350.b1	GE293205
61937161	CCAH2350.g1	GE293206
61937162	CCAH2351.b1	GE293207
61937163	CCAH2351.g1	GE293208
61937164	CCAH2353.b1	GE293209
61937165	CCAH2353.g1	GE293210
61937166	CCAH2354.b1	GE293211
61937167	CCAH2354.g1	GE293212
61937168	CCAH2355.b1	GE293213
61937169	CCAH2355.g1	GE293214
61937170	CCAH2356.b1	GE293215
61937171	CCAH2356.g1	GE293216
61937172	CCAH2357.b1	GE293217
61937173	CCAH2357.g1	GE293218
61937174	CCAH2358.b1	GE293219
61937175	CCAH2358.g1	GE293220
61937176	CCAH2359.b1	GE293221
61937177	CCAH2359.g1	GE293222
61937178	CCAH2360.b1	GE293223
61937179	CCAH2360.g1	GE293224
61937180	CCAH2362.b1	GE293225
61937181	CCAH2362.g1	GE293226
61937182	CCAH2363.b1	GE293227
61937183	CCAH2363.g1	GE293228
61937184	CCAH2364.b1	GE293229
61937185	CCAH2364.g1	GE293230
61937186	CCAH2365.b1	GE293231
61937187	CCAH2366.b1	GE293232
61937188	CCAH2366.g1	GE293233
61937189	CCAH2367.b1	GE293234
61937190	CCAH2367.g1	GE293235
61937191	CCAH2368.b1	GE293236
61937192	CCAH2368.g1	GE293237
61937193	CCAH2369.b1	GE293238

61937194	CCAH2369.g1	GE293239
61937195	CCAH2370.b1	GE293240
61937196	CCAH2371.b1	GE293241
61937197	CCAH2371.g1	GE293242
61937198	CCAH2372.b1	GE293243
61937199	CCAH2372.g1	GE293244
61937200	CCAH2373.b1	GE293245
61937201	CCAH2373.g1	GE293246
61937202	CCAH2374.b1	GE293247
61937203	CCAH2374.g1	GE293248
61937204	CCAH2375.b1	GE293249
61937205	CCAH2375.g1	GE293250
61937206	CCAH2376.b1	GE293251
61937207	CCAH2377.b1	GE293252
61937208	CCAH2377.g1	GE293253
61937209	CCAH2378.b1	GE293254
61937210	CCAH2379.b1	GE293255
61937211	CCAH2379.g1	GE293256
61937212	CCAH2380.b1	GE293257
61937213	CCAH2380.g1	GE293258
61937214	CCAH2382.b1	GE293259
61937215	CCAH2382.g1	GE293260
61937216	CCAH2383.b1	GE293261
61937217	CCAH2383.g1	GE293262
61937218	CCAH2384.b1	GE293263
61937219	CCAH2384.g1	GE293264
61937220	CCAH2387.b1	GE293265
61937221	CCAH2387.g1	GE293266
61937222	CCAH2388.g1	GE293267
61937223	CCAH2389.b1	GE293268
61937224	CCAH2389.g1	GE293269
61937225	CCAH2390.b1	GE293270
61937226	CCAH2390.g1	GE293271
61937227	CCAH2391.b1	GE293272
61937228	CCAH2391.g1	GE293273
61937229	CCAH2392.b1	GE293274
61937230	CCAH2392.g1	GE293275
61937231	CCAH2394.b1	GE293276
61937232	CCAH2394.g1	GE293277
61937233	CCAH2395.b1	GE293278
61937234	CCAH2395.g1	GE293279
61937235	CCAH2396.b1	GE293280
61937236	CCAH2397.b1	GE293281
61937237	CCAH2397.g1	GE293282
61937238	CCAH2398.b1	GE293283
61937239	CCAH2398.g1	GE293284
61937240	CCAH2399.b1	GE293285
61937241	CCAH2399.g1	GE293286
61937242	CCAH2400.b1	GE293287
61937243	CCAH2400.g1	GE293288
61937244	CCAH2401.b1	GE293289
61937245	CCAH2401.g1	GE293290
61937246	CCAH2402.b1	GE293291
61937247	CCAH2403.b1	GE293292
61937248	CCAH2403.g1	GE293293
61937249	CCAH2404.b1	GE293294
61937250	CCAH2404.g1	GE293295
61937251	CCAH2405.b1	GE293296
61937252	CCAH2405.g1	GE293297
61937253	CCAH2406.b1	GE293298
61937254	CCAH2406.g1	GE293299
61937255	CCAH2407.b1	GE293300
61937256	CCAH2407.g1	GE293301

61937257	CCAH2409.b1	GE293302
61937258	CCAH2411.b1	GE293303
61937259	CCAH2411.g1	GE293304
61937260	CCAH2412.b1	GE293305
61937261	CCAH2412.g1	GE293306
61937262	CCAH2413.b1	GE293307
61937263	CCAH2414.b1	GE293308
61937264	CCAH2415.g1	GE293309
61937265	CCAH2416.b1	GE293310
61937266	CCAH2416.g1	GE293311
61937267	CCAH2417.b1	GE293312
61937268	CCAH2417.g1	GE293313
61937269	CCAH2418.b1	GE293314
61937270	CCAH2419.b1	GE293315
61937271	CCAH2419.g1	GE293316
61937272	CCAH2420.b1	GE293317
61937273	CCAH2420.g1	GE293318
61937274	CCAH2422.b1	GE293319
61937275	CCAH2422.g1	GE293320
61937276	CCAH2423.b1	GE293321
61937277	CCAH2423.g1	GE293322
61937278	CCAH2425.b1	GE293323
61937279	CCAH2428.b1	GE293324
61937280	CCAH2428.g1	GE293325
61937281	CCAH2430.b1	GE293326
61937282	CCAH2430.g1	GE293327
61937283	CCAH2431.b1	GE293328
61937284	CCAH2431.g1	GE293329
61937285	CCAH2432.g1	GE293330
61937286	CCAH2433.b1	GE293331
61937287	CCAH2433.g1	GE293332
61937288	CCAH2434.b1	GE293333
61937289	CCAH2434.g1	GE293334
61937290	CCAH2435.b1	GE293335
61937291	CCAH2435.g1	GE293336
61937292	CCAH2436.b1	GE293337
61937293	CCAH2436.g1	GE293338
61937294	CCAH2438.b1	GE293339
61937295	CCAH2438.g1	GE293340
61937296	CCAH2439.b1	GE293341
61937297	CCAH2439.g1	GE293342
61937298	CCAH2441.b1	GE293343
61937299	CCAH2441.g1	GE293344
61937300	CCAH2442.b1	GE293345
61937301	CCAH2442.g1	GE293346
61937302	CCAH2443.b1	GE293347
61937303	CCAH2443.g1	GE293348
61937304	CCAH2444.b1	GE293349
61937305	CCAH2444.g1	GE293350
61937306	CCAH2445.b1	GE293351
61937307	CCAH2445.g1	GE293352
61937308	CCAH2446.g1	GE293353
61937309	CCAH2447.b1	GE293354
61937310	CCAH2447.g1	GE293355
61937311	CCAH2448.b1	GE293356
61937312	CCAH2449.b1	GE293357
61937313	CCAH2449.g1	GE293358
61937314	CCAH2450.b1	GE293359
61937315	CCAH2450.g1	GE293360
61937316	CCAH2451.b1	GE293361
61937317	CCAH2451.g1	GE293362
61937318	CCAH2452.b1	GE293363
61937319	CCAH2452.g1	GE293364

61937320	CCAH2453.b1	GE293365
61937321	CCAH2453.g1	GE293366
61937322	CCAH2454.b1	GE293367
61937323	CCAH2454.g1	GE293368
61937324	CCAH2455.b1	GE293369
61937325	CCAH2455.g1	GE293370
61937326	CCAH2456.b1	GE293371
61937327	CCAH2456.g1	GE293372
61937328	CCAH2458.b1	GE293373
61937329	CCAH2458.g1	GE293374
61937330	CCAH2459.b1	GE293375
61937331	CCAH2459.g1	GE293376
61937332	CCAH2460.b1	GE293377
61937333	CCAH2460.g1	GE293378
61937334	CCAH2461.b1	GE293379
61937335	CCAH2461.g1	GE293380
61937336	CCAH2462.b1	GE293381
61937337	CCAH2463.b1	GE293382
61937338	CCAH2463.g1	GE293383
61937339	CCAH2464.b1	GE293384
61937340	CCAH2464.g1	GE293385
61937341	CCAH2465.b1	GE293386
61937342	CCAH2465.g1	GE293387
61937343	CCAH2466.b1	GE293388
61937344	CCAH2466.g1	GE293389
61937345	CCAH2467.b1	GE293390
61937346	CCAH2467.g1	GE293391
61937347	CCAH2470.b1	GE293392
61937348	CCAH2470.g1	GE293393
61937349	CCAH2471.b1	GE293394
61937350	CCAH2471.g1	GE293395
61937351	CCAH2472.b1	GE293396
61937352	CCAH2472.g1	GE293397
61937353	CCAH2473.b1	GE293398
61937354	CCAH2473.g1	GE293399
61937355	CCAH2474.b1	GE293400
61937356	CCAH2474.g1	GE293401
61937357	CCAH2475.g1	GE293402
61937358	CCAH2476.b1	GE293403
61937359	CCAH2476.g1	GE293404
61937360	CCAH2477.b1	GE293405
61937361	CCAH2477.g1	GE293406
61937362	CCAH2478.b1	GE293407
61937363	CCAH2478.g1	GE293408
61937364	CCAH2479.b1	GE293409
61937365	CCAH2479.g1	GE293410
61937366	CCAH2480.b1	GE293411
61937367	CCAH2480.g1	GE293412
61937368	CCAH2481.b1	GE293413
61937369	CCAH2481.g1	GE293414
61937370	CCAH2482.b1	GE293415
61937371	CCAH2482.g1	GE293416
61937372	CCAH2483.b1	GE293417
61937373	CCAH2483.g1	GE293418
61937374	CCAH2484.b1	GE293419
61937375	CCAH2484.g1	GE293420
61937376	CCAH2485.b1	GE293421
61937377	CCAH2485.g1	GE293422
61937378	CCAH2486.b1	GE293423
61937379	CCAH2486.g1	GE293424
61937380	CCAH2487.b1	GE293425
61937381	CCAH2488.b1	GE293426
61937382	CCAH2489.b1	GE293427

61937383	CCAH2489.g1	GE293428
61937384	CCAH2490.b1	GE293429
61937385	CCAH2490.g1	GE293430
61937386	CCAH2491.b1	GE293431
61937387	CCAH2493.b1	GE293432
61937388	CCAH2493.g1	GE293433
61937389	CCAH2494.b1	GE293434
61937390	CCAH2494.g1	GE293435
61937391	CCAH2495.b1	GE293436
61937392	CCAH2496.g1	GE293437
61937393	CCAH2497.b1	GE293438
61937394	CCAH2497.g1	GE293439
61937395	CCAH2498.b1	GE293440
61937396	CCAH2498.g1	GE293441
61937397	CCAH2499.b1	GE293442
61937398	CCAH2499.g1	GE293443
61937399	CCAH2500.b1	GE293444
61937400	CCAH2501.b1	GE293445
61937401	CCAH2501.g1	GE293446
61937402	CCAH2503.b1	GE293447
61937403	CCAH2503.g1	GE293448
61937404	CCAH2504.b1	GE293449
61937405	CCAH2504.g1	GE293450
61937406	CCAH2505.b1	GE293451
61937407	CCAH2505.g1	GE293452
61937408	CCAH2506.b1	GE293453
61937409	CCAH2506.g1	GE293454
61937410	CCAH2507.b1	GE293455
61937411	CCAH2507.g1	GE293456
61937412	CCAH2508.b1	GE293457
61937413	CCAH2509.b1	GE293458
61937414	CCAH2510.b1	GE293459
61937415	CCAH2510.g1	GE293460
61937416	CCAH2511.b1	GE293461
61937417	CCAH2511.g1	GE293462
61937418	CCAH2512.b1	GE293463
61937419	CCAH2512.g1	GE293464
61937420	CCAH2513.b1	GE293465
61937421	CCAH2513.g1	GE293466
61937422	CCAH2514.b1	GE293467
61937423	CCAH2514.g1	GE293468
61937424	CCAH2516.b1	GE293469
61937425	CCAH2516.g1	GE293470
61937426	CCAH2517.b1	GE293471
61937427	CCAH2517.g1	GE293472
61937428	CCAH2518.b1	GE293473
61937429	CCAH2518.g1	GE293474
61937430	CCAH2520.b1	GE293475
61937431	CCAH2520.g1	GE293476
61937432	CCAH2521.b1	GE293477
61937433	CCAH2521.g1	GE293478
61937434	CCAH2522.b1	GE293479
61937435	CCAH2522.g1	GE293480
61937436	CCAH2523.b1	GE293481
61937437	CCAH2523.g1	GE293482
61937438	CCAH2524.b1	GE293483
61937439	CCAH2524.g1	GE293484
61937440	CCAH2526.b1	GE293485
61937441	CCAH2526.g1	GE293486
61937442	CCAH2527.b1	GE293487
61937443	CCAH2527.g1	GE293488
61937444	CCAH2528.b1	GE293489
61937445	CCAH2528.g1	GE293490

61937446	CCAH2529.b1	GE293491
61937447	CCAH2529.g1	GE293492
61937448	CCAH2530.b1	GE293493
61937449	CCAH2531.b1	GE293494
61937450	CCAH2531.g1	GE293495
61937451	CCAH2532.b1	GE293496
61937452	CCAH2532.g1	GE293497
61937453	CCAH2533.b1	GE293498
61937454	CCAH2533.g1	GE293499
61937455	CCAH2534.b1	GE293500
61937456	CCAH2534.g1	GE293501
61937457	CCAH2535.b1	GE293502
61937458	CCAH2535.g1	GE293503
61937459	CCAH2536.b1	GE293504
61937460	CCAH2536.g1	GE293505
61937461	CCAH2537.b1	GE293506
61937462	CCAH2537.g1	GE293507
61937463	CCAH2538.b1	GE293508
61937464	CCAH2538.g1	GE293509
61937465	CCAH2540.b1	GE293510
61937466	CCAH2540.g1	GE293511
61937467	CCAH2541.b1	GE293512
61937468	CCAH2541.g1	GE293513
61937469	CCAH2542.b1	GE293514
61937470	CCAH2542.g1	GE293515
61937471	CCAH2543.b1	GE293516
61937472	CCAH2543.g1	GE293517
61937473	CCAH2544.b1	GE293518
61937474	CCAH2545.b1	GE293519
61937475	CCAH2546.b1	GE293520
61937476	CCAH2546.g1	GE293521
61937477	CCAH2547.b1	GE293522
61937478	CCAH2547.g1	GE293523
61937479	CCAH2548.b1	GE293524
61937480	CCAH2548.g1	GE293525
61937481	CCAH2549.b1	GE293526
61937482	CCAH2549.g1	GE293527
61937483	CCAH2550.b1	GE293528
61937484	CCAH2550.g1	GE293529
61937485	CCAH2551.b1	GE293530
61937486	CCAH2551.g1	GE293531
61937487	CCAH2552.b1	GE293532
61937488	CCAH2552.g1	GE293533
61937489	CCAH2553.b1	GE293534
61937490	CCAH2553.g1	GE293535
61937491	CCAH2554.b1	GE293536
61937492	CCAH2555.b1	GE293537
61937493	CCAH2555.g1	GE293538
61937494	CCAH2556.b1	GE293539
61937495	CCAH2556.g1	GE293540
61937496	CCAH2558.g1	GE293541
61937497	CCAH2559.b1	GE293542
61937498	CCAH2559.g1	GE293543
61937499	CCAH2560.b1	GE293544
61937500	CCAH2560.g1	GE293545
61937501	CCAH2561.b1	GE293546
61937502	CCAH2561.g1	GE293547
61937503	CCAH2562.b1	GE293548
61937504	CCAH2562.g1	GE293549
61937505	CCAH2563.b1	GE293550
61937506	CCAH2563.g1	GE293551
61937507	CCAH2564.b1	GE293552
61937508	CCAH2564.g1	GE293553

61937509	CCAH2566.b1	GE293554
61937510	CCAH2566.g1	GE293555
61937511	CCAH2567.b1	GE293556
61937512	CCAH2567.g1	GE293557
61937513	CCAH2569.b1	GE293558
61937514	CCAH2569.g1	GE293559
61937515	CCAH2570.b1	GE293560
61937516	CCAH2570.g1	GE293561
61937517	CCAH2571.b1	GE293562
61937518	CCAH2571.g1	GE293563
61937519	CCAH2572.b1	GE293564
61937520	CCAH2572.g1	GE293565
61937521	CCAH2573.b1	GE293566
61937522	CCAH2573.g1	GE293567
61937523	CCAH2574.b1	GE293568
61937524	CCAH2574.g1	GE293569
61937525	CCAH2575.b1	GE293570
61937526	CCAH2576.b1	GE293571
61937527	CCAH2576.g1	GE293572
61937528	CCAH2577.b1	GE293573
61937529	CCAH2577.g1	GE293574
61937530	CCAH2578.b1	GE293575
61937531	CCAH2578.g1	GE293576
61937532	CCAH2579.b1	GE293577
61937533	CCAH2579.g1	GE293578
61937534	CCAH2580.b1	GE293579
61937535	CCAH2581.b1	GE293580
61937536	CCAH2581.g1	GE293581
61937537	CCAH2582.b1	GE293582
61937538	CCAH2582.g1	GE293583
61937539	CCAH2583.b1	GE293584
61937540	CCAH2583.g1	GE293585
61937541	CCAH2584.b1	GE293586
61937542	CCAH2584.g1	GE293587
61937543	CCAH2585.b1	GE293588
61937544	CCAH2585.g1	GE293589
61937545	CCAH2586.b1	GE293590
61937546	CCAH2586.g1	GE293591
61937547	CCAH2587.b1	GE293592
61937548	CCAH2588.b1	GE293593
61937549	CCAH2588.g1	GE293594
61937550	CCAH2589.b1	GE293595
61937551	CCAH2589.g1	GE293596
61937552	CCAH2591.b1	GE293597
61937553	CCAH2591.g1	GE293598
61937554	CCAH2592.b1	GE293599
61937555	CCAH2593.b1	GE293600
61937556	CCAH2593.g1	GE293601
61937557	CCAH2594.b1	GE293602
61937558	CCAH2594.g1	GE293603
61937559	CCAH2595.b1	GE293604
61937560	CCAH2595.g1	GE293605
61937561	CCAH2596.b1	GE293606
61937562	CCAH2596.g1	GE293607
61937563	CCAH2597.b1	GE293608
61937564	CCAH2597.g1	GE293609
61937565	CCAH2598.b1	GE293610
61937566	CCAH2598.g1	GE293611
61937567	CCAH2599.g1	GE293612
61937568	CCAH2600.b1	GE293613
61937569	CCAH2600.g1	GE293614
61937570	CCAH2601.b1	GE293615
61937571	CCAH2601.g1	GE293616

61937572	CCAH2602.b1	GE293617
61937573	CCAH2602.g1	GE293618
61937574	CCAH2603.b1	GE293619
61937575	CCAH2604.b1	GE293620
61937576	CCAH2604.g1	GE293621
61937577	CCAH2606.b1	GE293622
61937578	CCAH2606.g1	GE293623
61937579	CCAH2608.b1	GE293624
61937580	CCAH2610.b1	GE293625
61937581	CCAH2610.g1	GE293626
61937582	CCAH2611.b1	GE293627
61937583	CCAH2612.b1	GE293628
61937584	CCAH2612.g1	GE293629
61937585	CCAH2613.b1	GE293630
61937586	CCAH2613.g1	GE293631
61937587	CCAH2614.b1	GE293632
61937588	CCAH2614.g1	GE293633
61937589	CCAH2615.b1	GE293634
61937590	CCAH2615.g1	GE293635
61937591	CCAH2616.g1	GE293636
61937592	CCAH2618.g1	GE293637
61937593	CCAH2620.b1	GE293638
61937594	CCAH2621.b1	GE293639
61937595	CCAH2621.g1	GE293640
61937596	CCAH2622.b1	GE293641
61937597	CCAH2622.g1	GE293642
61937598	CCAH2623.b1	GE293643
61937599	CCAH2623.g1	GE293644
61937600	CCAH2624.b1	GE293645
61937601	CCAH2624.g1	GE293646
61937602	CCAH2625.b1	GE293647
61937603	CCAH2625.g1	GE293648
61937604	CCAH2626.b1	GE293649
61937605	CCAH2626.g1	GE293650
61937606	CCAH2627.b1	GE293651
61937607	CCAH2627.g1	GE293652
61937608	CCAH2628.b1	GE293653
61937609	CCAH2628.g1	GE293654
61937610	CCAH2629.b1	GE293655
61937611	CCAH2630.b1	GE293656
61937612	CCAH2631.b1	GE293657
61937613	CCAH2633.b1	GE293658
61937614	CCAH2633.g1	GE293659
61937615	CCAH2634.b1	GE293660
61937616	CCAH2634.g1	GE293661
61937617	CCAH2635.b1	GE293662
61937618	CCAH2635.g1	GE293663
61937619	CCAH2636.b1	GE293664
61937620	CCAH2636.g1	GE293665
61937621	CCAH2637.b1	GE293666
61937622	CCAH2637.g1	GE293667
61937623	CCAH2638.g1	GE293668
61937624	CCAH2639.b1	GE293669
61937625	CCAH2639.g1	GE293670
61937626	CCAH2640.b1	GE293671
61937627	CCAH2641.b1	GE293672
61937628	CCAH2641.g1	GE293673
61937629	CCAH2644.b1	GE293674
61937630	CCAH2644.g1	GE293675
61937631	CCAH2646.b1	GE293676
61937632	CCAH2646.g1	GE293677
61937633	CCAH2647.g1	GE293678
61937634	CCAH2648.b1	GE293679

61937635	CCAH2648.g1	GE293680
61937636	CCAH2649.b1	GE293681
61937637	CCAH2649.g1	GE293682
61937638	CCAH2650.b1	GE293683
61937639	CCAH2650.g1	GE293684
61937640	CCAH2651.b1	GE293685
61937641	CCAH2651.g1	GE293686
61937642	CCAH2652.b1	GE293687
61937643	CCAH2652.g1	GE293688
61937644	CCAH2653.b1	GE293689
61937645	CCAH2653.g1	GE293690
61937646	CCAH2655.b1	GE293691
61937647	CCAH2655.g1	GE293692
61937648	CCAH2656.b1	GE293693
61937649	CCAH2657.b1	GE293694
61937650	CCAH2657.g1	GE293695
61937651	CCAH2659.b1	GE293696
61937652	CCAH2660.b1	GE293697
61937653	CCAH2662.b1	GE293698
61937654	CCAH2662.g1	GE293699
61937655	CCAH2663.b1	GE293700
61937656	CCAH2663.g1	GE293701
61937657	CCAH2664.b1	GE293702
61937658	CCAH2664.g1	GE293703
61937659	CCAH2665.b1	GE293704
61937660	CCAH2665.g1	GE293705
61937661	CCAH2666.b1	GE293706
61937662	CCAH2666.g1	GE293707
61937663	CCAH2667.b1	GE293708
61937664	CCAH2667.g1	GE293709
61937665	CCAH2668.b1	GE293710
61937666	CCAH2668.g1	GE293711
61937667	CCAH2669.b1	GE293712
61937668	CCAH2669.g1	GE293713
61937669	CCAH2670.b1	GE293714
61937670	CCAH2670.g1	GE293715
61937671	CCAH2671.b1	GE293716
61937672	CCAH2671.g1	GE293717
61937673	CCAH2672.b1	GE293718
61937674	CCAH2672.g1	GE293719
61937675	CCAH2673.b1	GE293720
61937676	CCAH2674.b1	GE293721
61937677	CCAH2674.g1	GE293722
61937678	CCAH2675.b1	GE293723
61937679	CCAH2675.g1	GE293724
61937680	CCAH2676.b1	GE293725
61937681	CCAH2676.g1	GE293726
61937682	CCAH2677.b1	GE293727
61937683	CCAH2677.g1	GE293728
61937684	CCAH2678.b1	GE293729
61937685	CCAH2678.g1	GE293730
61937686	CCAH2679.b1	GE293731
61937687	CCAH2679.g1	GE293732
61937688	CCAH2680.b1	GE293733
61937689	CCAH2680.g1	GE293734
61937690	CCAH2681.b1	GE293735
61937691	CCAH2681.g1	GE293736
61937692	CCAH2683.b1	GE293737
61937693	CCAH2683.g1	GE293738
61937694	CCAH2684.b1	GE293739
61937695	CCAH2684.g1	GE293740
61937696	CCAH2685.b1	GE293741
61937697	CCAH2685.g1	GE293742

61937698	CCAH2686.b1	GE293743
61937699	CCAH2686.g1	GE293744
61937700	CCAH2687.g1	GE293745
61937701	CCAH2690.b1	GE293746
61937702	CCAH2690.g1	GE293747
61937703	CCAH2691.b1	GE293748
61937704	CCAH2692.b1	GE293749
61937705	CCAH2692.g1	GE293750
61937706	CCAH2694.b1	GE293751
61937707	CCAH2694.g1	GE293752
61937708	CCAH2696.b1	GE293753
61937709	CCAH2696.g1	GE293754
61937710	CCAH2697.b1	GE293755
61937711	CCAH2698.b1	GE293756
61937712	CCAH2698.g1	GE293757
61937713	CCAH2699.b1	GE293758
61937714	CCAH2699.g1	GE293759
61937715	CCAH2700.b1	GE293760
61937716	CCAH2701.b1	GE293761
61937717	CCAH2701.g1	GE293762
61937718	CCAH2702.b1	GE293763
61937719	CCAH2702.g1	GE293764
61937720	CCAH2703.b1	GE293765
61937721	CCAH2703.g1	GE293766
61937722	CCAH2704.b1	GE293767
61937723	CCAH2704.g1	GE293768
61937724	CCAH2705.b1	GE293769
61937725	CCAH2705.g1	GE293770
61937726	CCAH2706.b1	GE293771
61937727	CCAH2707.b1	GE293772
61937728	CCAH2707.g1	GE293773
61937729	CCAH2708.b1	GE293774
61937730	CCAH2709.b1	GE293775
61937731	CCAH2709.g1	GE293776
61937732	CCAH2710.b1	GE293777
61937733	CCAH2710.g1	GE293778
61937734	CCAH2711.b1	GE293779
61937735	CCAH2712.b1	GE293780
61937736	CCAH2713.b1	GE293781
61937737	CCAH2713.g1	GE293782
61937738	CCAH2714.b1	GE293783
61937739	CCAH2715.b1	GE293784
61937740	CCAH2715.g1	GE293785
61937741	CCAH2716.b1	GE293786
61937742	CCAH2716.g1	GE293787
61937743	CCAH2717.b1	GE293788
61937744	CCAH2720.b1	GE293789
61937745	CCAH2720.g1	GE293790
61937746	CCAH2721.b1	GE293791
61937747	CCAH2721.g1	GE293792
61937748	CCAH2722.b1	GE293793
61937749	CCAH2722.g1	GE293794
61937750	CCAH2723.b1	GE293795
61937751	CCAH2723.g1	GE293796
61937752	CCAH2724.b1	GE293797
61937753	CCAH2725.b1	GE293798
61937754	CCAH2726.b1	GE293799
61937755	CCAH2726.g1	GE293800
61937756	CCAH2727.b1	GE293801
61937757	CCAH2727.g1	GE293802
61937758	CCAH2729.b1	GE293803
61937759	CCAH2729.g1	GE293804
61937760	CCAH2730.b1	GE293805

61937761	CCAH2730.g1	GE293806
61937762	CCAH2731.g1	GE293807
61937763	CCAH2732.b1	GE293808
61937764	CCAH2733.b1	GE293809
61937765	CCAH2733.g1	GE293810
61937766	CCAH2734.b1	GE293811
61937767	CCAH2734.g1	GE293812
61937768	CCAH2735.b1	GE293813
61937769	CCAH2735.g1	GE293814
61937770	CCAH2736.b1	GE293815
61937771	CCAH2736.g1	GE293816
61937772	CCAH2737.b1	GE293817
61937773	CCAH2737.g1	GE293818
61937774	CCAH2740.b1	GE293819
61937775	CCAH2740.g1	GE293820
61937776	CCAH2741.b1	GE293821
61937777	CCAH2741.g1	GE293822
61937778	CCAH2742.b1	GE293823
61937779	CCAH2743.b1	GE293824
61937780	CCAH2743.g1	GE293825
61937781	CCAH2744.b1	GE293826
61937782	CCAH2744.g1	GE293827
61937783	CCAH2745.b1	GE293828
61937784	CCAH2745.g1	GE293829
61937785	CCAH2746.b1	GE293830
61937786	CCAH2746.g1	GE293831
61937787	CCAH2747.b1	GE293832
61937788	CCAH2748.g1	GE293833
61937789	CCAH2749.g1	GE293834
61937790	CCAH2750.b1	GE293835
61937791	CCAH2751.b1	GE293836
61937792	CCAH2751.g1	GE293837
61937793	CCAH2752.b1	GE293838
61937794	CCAH2752.g1	GE293839
61937795	CCAH2753.b1	GE293840
61937796	CCAH2753.g1	GE293841
61937797	CCAH2754.b1	GE293842
61937798	CCAH2754.g1	GE293843
61937799	CCAH2756.b1	GE293844
61937800	CCAH2756.g1	GE293845
61937801	CCAH2758.g1	GE293846
61937802	CCAH2759.b1	GE293847
61937803	CCAH2759.g1	GE293848
61937804	CCAH2760.b1	GE293849
61937805	CCAH2760.g1	GE293850
61937806	CCAH2761.g1	GE293851
61937807	CCAH2762.b1	GE293852
61937808	CCAH2762.g1	GE293853
61937809	CCAH2763.b1	GE293854
61937810	CCAH2764.b1	GE293855
61937811	CCAH2764.g1	GE293856
61937812	CCAH2765.b1	GE293857
61937813	CCAH2766.b1	GE293858
61937814	CCAH2766.g1	GE293859
61937815	CCAH2767.b1	GE293860
61937816	CCAH2767.g1	GE293861
61937817	CCAH2769.b1	GE293862
61937818	CCAH2769.g1	GE293863
61937819	CCAH2770.b1	GE293864
61937820	CCAH2770.g1	GE293865
61937821	CCAH2771.g1	GE293866
61937822	CCAH2772.g1	GE293867
61937823	CCAH2773.b1	GE293868

61937824	CCAH2773.g1	GE293869
61937825	CCAH2774.b1	GE293870
61937826	CCAH2775.b1	GE293871
61937827	CCAH2776.b1	GE293872
61937828	CCAH2777.b1	GE293873
61937829	CCAH2777.g1	GE293874
61937830	CCAH2778.b1	GE293875
61937831	CCAH2778.g1	GE293876
61937832	CCAH2779.b1	GE293877
61937833	CCAH2779.g1	GE293878
61937834	CCAH2780.b1	GE293879
61937835	CCAH2783.g1	GE293880
61937836	CCAH2784.b1	GE293881
61937837	CCAH2784.g1	GE293882
61937838	CCAH2785.b1	GE293883
61937839	CCAH2785.g1	GE293884
61937840	CCAH2786.b1	GE293885
61937841	CCAH2786.g1	GE293886
61937842	CCAH2789.b1	GE293887
61937843	CCAH2789.g1	GE293888
61937844	CCAH2790.b1	GE293889
61937845	CCAH2790.g1	GE293890
61937846	CCAH2791.b1	GE293891
61937847	CCAH2791.g1	GE293892
61937848	CCAH2792.b1	GE293893
61937849	CCAH2792.g1	GE293894
61937850	CCAH2793.b1	GE293895
61937851	CCAH2793.g1	GE293896
61937852	CCAH2794.b1	GE293897
61937853	CCAH2794.g1	GE293898
61937854	CCAH2795.b1	GE293899
61937855	CCAH2795.g1	GE293900
61937856	CCAH2796.b1	GE293901
61937857	CCAH2796.g1	GE293902
61937858	CCAH2797.b1	GE293903
61937859	CCAH2797.g1	GE293904
61937860	CCAH2798.b1	GE293905
61937861	CCAH2798.g1	GE293906
61937862	CCAH2799.b1	GE293907
61937863	CCAH2800.g1	GE293908
61937864	CCAH2801.b1	GE293909
61937865	CCAH2802.b1	GE293910
61937866	CCAH2803.b1	GE293911
61937867	CCAH2803.g1	GE293912
61937868	CCAH2804.b1	GE293913
61937869	CCAH2805.b1	GE293914
61937870	CCAH2806.b1	GE293915
61937871	CCAH2806.g1	GE293916
61937872	CCAH2807.b1	GE293917
61937873	CCAH2807.g1	GE293918
61937874	CCAH2808.b1	GE293919
61937875	CCAH2809.b1	GE293920
61937876	CCAH2810.b1	GE293921
61937877	CCAH2811.b1	GE293922
61937878	CCAH2811.g1	GE293923
61937879	CCAH2812.b1	GE293924
61937880	CCAH2812.g1	GE293925
61937881	CCAH2813.b1	GE293926
61937882	CCAH2813.g1	GE293927
61937883	CCAH2814.b1	GE293928
61937884	CCAH2814.g1	GE293929
61937885	CCAH2815.b1	GE293930
61937886	CCAH2816.b1	GE293931

61937887	CCAH2816.g1	GE293932
61937888	CCAH2817.g1	GE293933
61937889	CCAH2818.b1	GE293934
61937890	CCAH2820.g1	GE293935
61937891	CCAH2821.b1	GE293936
61937892	CCAH2821.g1	GE293937
61937893	CCAH2822.b1	GE293938
61937894	CCAH2822.g1	GE293939
61937895	CCAH2823.b1	GE293940
61937896	CCAH2823.g1	GE293941
61937897	CCAH2824.b1	GE293942
61937898	CCAH2824.g1	GE293943
61937899	CCAH2825.b1	GE293944
61937900	CCAH2826.b1	GE293945
61937901	CCAH2826.g1	GE293946
61937902	CCAH2827.b1	GE293947
61937903	CCAH2829.b1	GE293948
61937904	CCAH2829.g1	GE293949
61937905	CCAH2830.b1	GE293950
61937906	CCAH2830.g1	GE293951
61937907	CCAH2831.b1	GE293952
61937908	CCAH2832.b1	GE293953
61937909	CCAH2832.g1	GE293954
61937910	CCAH2833.b1	GE293955
61937911	CCAH2833.g1	GE293956
61937912	CCAH2835.b1	GE293957
61937913	CCAH2835.g1	GE293958
61937914	CCAH2836.b1	GE293959
61937915	CCAH2836.g1	GE293960
61937916	CCAH2837.b1	GE293961
61937917	CCAH2837.g1	GE293962
61937918	CCAH2838.b1	GE293963
61937919	CCAH2839.b1	GE293964
61937920	CCAH2840.b1	GE293965
61937921	CCAH2841.b1	GE293966
61937922	CCAH2841.g1	GE293967
61937923	CCAH2842.b1	GE293968
61937924	CCAH2842.g1	GE293969
61937925	CCAH2843.b1	GE293970
61937926	CCAH2844.b1	GE293971
61937927	CCAH2844.g1	GE293972
61937928	CCAH2845.b1	GE293973
61937929	CCAH2845.g1	GE293974
61937930	CCAH2846.b1	GE293975
61937931	CCAH2847.b1	GE293976
61937932	CCAH2847.g1	GE293977
61937933	CCAH2848.b1	GE293978
61937934	CCAH2848.g1	GE293979
61937935	CCAH2849.b1	GE293980
61937936	CCAH2850.b1	GE293981
61937937	CCAH2851.b1	GE293982
61937938	CCAH2851.g1	GE293983
61937939	CCAH2852.b1	GE293984
61937940	CCAH2852.g1	GE293985
61937941	CCAH2854.b1	GE293986
61937942	CCAH2855.b1	GE293987
61937943	CCAH2855.g1	GE293988
61937944	CCAH2856.b1	GE293989
61937945	CCAH2858.b1	GE293990
61937946	CCAH2858.g1	GE293991
61937947	CCAH2859.b1	GE293992
61937948	CCAH2859.g1	GE293993
61937949	CCAH2860.b1	GE293994

61937950	CCAH2861.b1	GE293995
61937951	CCAH2861.g1	GE293996
61937952	CCAH2862.b1	GE293997
61937953	CCAH2863.b1	GE293998
61937954	CCAH2863.g1	GE293999
61937955	CCAH2864.b1	GE294000
61937956	CCAH2865.b1	GE294001
61937957	CCAH2865.g1	GE294002
61937958	CCAH2866.b1	GE294003
61937959	CCAH2866.g1	GE294004
61937960	CCAH2867.b1	GE294005
61937961	CCAH2868.b1	GE294006
61937962	CCAH2869.b1	GE294007
61937963	CCAH2870.b1	GE294008
61937964	CCAH2871.b1	GE294009
61937965	CCAH2871.g1	GE294010
61937966	CCAH2872.b1	GE294011
61937967	CCAH2872.g1	GE294012
61937968	CCAH2873.g1	GE294013
61937969	CCAH2874.b1	GE294014
61937970	CCAH2874.g1	GE294015
61937971	CCAH2875.b1	GE294016
61937972	CCAH2875.g1	GE294017
61937973	CCAH2876.b1	GE294018
61937974	CCAH2876.g1	GE294019
61937975	CCAH2877.b1	GE294020
61937976	CCAH2877.g1	GE294021
61937977	CCAH2879.b1	GE294022
61937978	CCAH2879.g1	GE294023
61937979	CCAH2880.b1	GE294024
61937980	CCAH2880.g1	GE294025
61937981	CCAH2881.g1	GE294026
61937982	CCAH2882.b1	GE294027
61937983	CCAH2882.g1	GE294028
61937984	CCAH2883.b1	GE294029
61937985	CCAH2883.g1	GE294030
61937986	CCAH2884.b1	GE294031
61937987	CCAH2884.g1	GE294032
61937988	CCAH2885.b1	GE294033
61937989	CCAH2886.b1	GE294034
61937990	CCAH2886.g1	GE294035
61937991	CCAH2887.b1	GE294036
61937992	CCAH2887.g1	GE294037
61937993	CCAH2888.b1	GE294038
61937994	CCAH2888.g1	GE294039
61937995	CCAH2889.b1	GE294040
61937996	CCAH2889.g1	GE294041
61937997	CCAH2890.b1	GE294042
61937998	CCAH2890.g1	GE294043
61937999	CCAH2891.b1	GE294044
61938000	CCAH2892.b1	GE294045
61938001	CCAH2892.g1	GE294046
61938002	CCAH2893.b1	GE294047
61938003	CCAH2894.b1	GE294048
61938004	CCAH2894.g1	GE294049
61938005	CCAH2895.b1	GE294050
61938006	CCAH2895.g1	GE294051
61938007	CCAH2896.b1	GE294052
61938008	CCAH2896.g1	GE294053
61938009	CCAH2897.b1	GE294054
61938010	CCAH2898.b1	GE294055
61938011	CCAH2898.g1	GE294056
61938012	CCAH2900.b1	GE294057

61938013	CCAH2900.g1	GE294058
61938014	CCAH2901.b1	GE294059
61938015	CCAH2901.g1	GE294060
61938016	CCAH2902.b1	GE294061
61938017	CCAH2902.g1	GE294062
61938018	CCAH2903.b1	GE294063
61938019	CCAH2903.g1	GE294064
61938020	CCAH2904.b1	GE294065
61938021	CCAH2906.b1	GE294066
61938022	CCAH2907.b1	GE294067
61938023	CCAH2907.g1	GE294068
61938024	CCAH2908.b1	GE294069
61938025	CCAH2909.b1	GE294070
61938026	CCAH2909.g1	GE294071
61938027	CCAH2910.b1	GE294072
61938028	CCAH2910.g1	GE294073
61938029	CCAH2911.b1	GE294074
61938030	CCAH2911.g1	GE294075
61938031	CCAH2912.b1	GE294076
61938032	CCAH2912.g1	GE294077
61938033	CCAH2913.b1	GE294078
61938034	CCAH2913.g1	GE294079
61938035	CCAH2914.b1	GE294080
61938036	CCAH2914.g1	GE294081
61938037	CCAH2915.b1	GE294082
61938038	CCAH2916.b1	GE294083
61938039	CCAH2916.g1	GE294084
61938040	CCAH2917.b1	GE294085
61938041	CCAH2917.g1	GE294086
61938042	CCAH2918.b1	GE294087
61938043	CCAH2918.g1	GE294088
61938044	CCAH2919.b1	GE294089
61938045	CCAH2919.g1	GE294090
61938046	CCAH2920.b1	GE294091
61938047	CCAH2920.g1	GE294092
61938048	CCAH2921.b1	GE294093
61938049	CCAH2921.g1	GE294094
61938050	CCAH2922.b1	GE294095
61938051	CCAH2922.g1	GE294096
61938052	CCAH2923.b1	GE294097
61938053	CCAH2924.b1	GE294098
61938054	CCAH2925.g1	GE294099
61938055	CCAH2927.b1	GE294100
61938056	CCAH2928.b1	GE294101
61938057	CCAH2928.g1	GE294102
61938058	CCAH2929.b1	GE294103
61938059	CCAH2929.g1	GE294104
61938060	CCAH2930.b1	GE294105
61938061	CCAH2930.g1	GE294106
61938062	CCAH2931.b1	GE294107
61938063	CCAH2933.b1	GE294108
61938064	CCAH2933.g1	GE294109
61938065	CCAH2934.b1	GE294110
61938066	CCAH2934.g1	GE294111
61938067	CCAH2935.b1	GE294112
61938068	CCAH2935.g1	GE294113
61938069	CCAH2936.b1	GE294114
61938070	CCAH2936.g1	GE294115
61938071	CCAH2937.b1	GE294116
61938072	CCAH2937.g1	GE294117
61938073	CCAH2938.b1	GE294118
61938074	CCAH2938.g1	GE294119
61938075	CCAH2939.b1	GE294120

61938076	CCAH2939.g1	GE294121
61938077	CCAH2940.b1	GE294122
61938078	CCAH2940.g1	GE294123
61938079	CCAH2941.b1	GE294124
61938080	CCAH2943.b1	GE294125
61938081	CCAH2943.g1	GE294126
61938082	CCAH2944.b1	GE294127
61938083	CCAH2945.b1	GE294128
61938084	CCAH2945.g1	GE294129
61938085	CCAH2946.b1	GE294130
61938086	CCAH2946.g1	GE294131
61938087	CCAH2947.b1	GE294132
61938088	CCAH2947.g1	GE294133
61938089	CCAH2948.b1	GE294134
61938090	CCAH2948.g1	GE294135
61938091	CCAH2949.b1	GE294136
61938092	CCAH2949.g1	GE294137
61938093	CCAH2950.b1	GE294138
61938094	CCAH2951.b1	GE294139
61938095	CCAH2951.g1	GE294140
61938096	CCAH2952.b1	GE294141
61938097	CCAH2953.b1	GE294142
61938098	CCAH2954.b1	GE294143
61938099	CCAH2954.g1	GE294144
61938100	CCAH2955.b1	GE294145
61938101	CCAH2955.g1	GE294146
61938102	CCAH2956.b1	GE294147
61938103	CCAH2957.b1	GE294148
61938104	CCAH2957.g1	GE294149
61938105	CCAH2958.b1	GE294150
61938106	CCAH2959.b1	GE294151
61938107	CCAH2960.b1	GE294152
61938108	CCAH2960.g1	GE294153
61938109	CCAH2961.g1	GE294154
61938110	CCAH2962.b1	GE294155
61938111	CCAH2963.b1	GE294156
61938112	CCAH2963.g1	GE294157
61938113	CCAH2964.b1	GE294158
61938114	CCAH2964.g1	GE294159
61938115	CCAH2965.b1	GE294160
61938116	CCAH2966.b1	GE294161
61938117	CCAH2967.b1	GE294162
61938118	CCAH2967.g1	GE294163
61938119	CCAH2968.b1	GE294164
61938120	CCAH2968.g1	GE294165
61938121	CCAH2969.b1	GE294166
61938122	CCAH2970.g1	GE294167
61938123	CCAH2972.g1	GE294168
61938124	CCAH2973.b1	GE294169
61938125	CCAH2973.g1	GE294170
61938126	CCAH2974.b1	GE294171
61938127	CCAH2974.g1	GE294172
61938128	CCAH2975.b1	GE294173
61938129	CCAH2977.b1	GE294174
61938130	CCAH2977.g1	GE294175
61938131	CCAH2978.b1	GE294176
61938132	CCAH2978.g1	GE294177
61938133	CCAH2979.b1	GE294178
61938134	CCAH2980.b1	GE294179
61938135	CCAH2981.b1	GE294180
61938136	CCAH2981.g1	GE294181
61938137	CCAH2982.b1	GE294182
61938138	CCAH2982.g1	GE294183

61938139	CCAH2983.b1	GE294184
61938140	CCAH2983.g1	GE294185
61938141	CCAH2984.b1	GE294186
61938142	CCAH2985.b1	GE294187
61938143	CCAH2986.b1	GE294188
61938144	CCAH2986.g1	GE294189
61938145	CCAH2987.b1	GE294190
61938146	CCAH2987.g1	GE294191
61938147	CCAH2989.b1	GE294192
61938148	CCAH2989.g1	GE294193
61938149	CCAH2990.b1	GE294194
61938150	CCAH2990.g1	GE294195
61938151	CCAH2991.b1	GE294196
61938152	CCAH2993.b1	GE294197
61938153	CCAH2995.b1	GE294198
61938154	CCAH2995.g1	GE294199
61938155	CCAH2996.b1	GE294200
61938156	CCAH2996.g1	GE294201
61938157	CCAH2997.b1	GE294202
61938158	CCAH2997.g1	GE294203
61938159	CCAH2998.b1	GE294204
61938160	CCAH2999.g1	GE294205
61938161	CCAH3000.b1	GE294206
61938162	CCAH3001.b1	GE294207
61938163	CCAH3001.g1	GE294208
61938164	CCAH3002.b1	GE294209
61938165	CCAH3003.b1	GE294210
61938166	CCAH3003.g1	GE294211
61938167	CCAH3004.b1	GE294212
61938168	CCAH3005.b1	GE294213
61938169	CCAH3005.g1	GE294214
61938170	CCAH3006.b1	GE294215
61938171	CCAH3006.g1	GE294216
61938172	CCAH3007.b1	GE294217
61938173	CCAH3007.g1	GE294218
61938174	CCAH3008.b1	GE294219
61938175	CCAH3009.b1	GE294220
61938176	CCAH3009.g1	GE294221
61938177	CCAH3010.b1	GE294222
61938178	CCAH3010.g1	GE294223
61938179	CCAH3011.b1	GE294224
61938180	CCAH3011.g1	GE294225
61938181	CCAH3012.b1	GE294226
61938182	CCAH3012.g1	GE294227
61938183	CCAH3013.b1	GE294228
61938184	CCAH3013.g1	GE294229
61938185	CCAH3014.b1	GE294230
61938186	CCAH3014.g1	GE294231
61938187	CCAH3015.b1	GE294232
61938188	CCAH3016.b1	GE294233
61938189	CCAH3016.g1	GE294234
61938190	CCAH3017.b1	GE294235
61938191	CCAH3017.g1	GE294236
61938192	CCAH3018.g1	GE294237
61938193	CCAH3019.b1	GE294238
61938194	CCAH3019.g1	GE294239
61938195	CCAH3020.b1	GE294240
61938196	CCAH3020.g1	GE294241
61938197	CCAH3021.b1	GE294242
61938198	CCAH3021.g1	GE294243
61938199	CCAH3022.g1	GE294244
61938200	CCAH3023.b1	GE294245
61938201	CCAH3023.g1	GE294246

61938202	CCAH3024.b1	GE294247
61938203	CCAH3024.g1	GE294248
61938204	CCAH3025.b1	GE294249
61938205	CCAH3025.g1	GE294250
61938206	CCAH3026.b1	GE294251
61938207	CCAH3026.g1	GE294252
61938208	CCAH3027.b1	GE294253
61938209	CCAH3027.g1	GE294254
61938210	CCAH3028.b1	GE294255
61938211	CCAH3028.g1	GE294256
61938212	CCAH3029.b1	GE294257
61938213	CCAH3029.g1	GE294258
61938214	CCAH3030.b1	GE294259
61938215	CCAH3030.g1	GE294260
61938216	CCAH3031.b1	GE294261
61938217	CCAH3032.b1	GE294262
61938218	CCAH3033.b1	GE294263
61938219	CCAH3033.g1	GE294264
61938220	CCAH3034.b1	GE294265
61938221	CCAH3034.g1	GE294266
61938222	CCAH3035.b1	GE294267
61938223	CCAH3036.b1	GE294268
61938224	CCAH3036.g1	GE294269
61938225	CCAH3037.b1	GE294270
61938226	CCAH3037.g1	GE294271
61938227	CCAH3038.b1	GE294272
61938228	CCAH3039.b1	GE294273
61938229	CCAH3040.b1	GE294274
61938230	CCAH3040.g1	GE294275
61938231	CCAH3041.b1	GE294276
61938232	CCAH3041.g1	GE294277
61938233	CCAH3042.b1	GE294278
61938234	CCAH3042.g1	GE294279
61938235	CCAH3043.b1	GE294280
61938236	CCAH3043.g1	GE294281
61938237	CCAH3044.b1	GE294282
61938238	CCAH3044.g1	GE294283
61938239	CCAH3045.b1	GE294284
61938240	CCAH3046.b1	GE294285
61938241	CCAH3046.g1	GE294286
61938242	CCAH3047.b1	GE294287
61938243	CCAH3047.g1	GE294288
61938244	CCAH3048.b1	GE294289
61938245	CCAH3049.b1	GE294290
61938246	CCAH3049.g1	GE294291
61938247	CCAH3050.b1	GE294292
61938248	CCAH3050.g1	GE294293
61938249	CCAH3051.b1	GE294294
61938250	CCAH3051.g1	GE294295
61938251	CCAH3052.b1	GE294296
61938252	CCAH3053.b1	GE294297
61938253	CCAH3053.g1	GE294298
61938254	CCAH3054.b1	GE294299
61938255	CCAH3054.g1	GE294300
61938256	CCAH3055.b1	GE294301
61938257	CCAH3055.g1	GE294302
61938258	CCAH3056.b1	GE294303
61938259	CCAH3057.b1	GE294304
61938260	CCAH3058.b1	GE294305
61938261	CCAH3058.g1	GE294306
61938262	CCAH3059.b1	GE294307
61938263	CCAH3059.g1	GE294308
61938264	CCAH3060.b1	GE294309

61938265	CCAH3060.g1	GE294310
61938266	CCAH3061.b1	GE294311
61938267	CCAH3062.b1	GE294312
61938268	CCAH3062.g1	GE294313
61938269	CCAH3063.b1	GE294314
61938270	CCAH3063.g1	GE294315
61938271	CCAH3064.b1	GE294316
61938272	CCAH3064.g1	GE294317
61938273	CCAH3065.b1	GE294318
61938274	CCAH3066.b1	GE294319
61938275	CCAH3066.g1	GE294320
61938276	CCAH3067.g1	GE294321
61938277	CCAH3068.b1	GE294322
61938278	CCAH3068.g1	GE294323
61938279	CCAH3069.b1	GE294324
61938280	CCAH3070.b1	GE294325
61938281	CCAH3070.g1	GE294326
61938282	CCAH3071.b1	GE294327
61938283	CCAH3071.g1	GE294328
61938284	CCAH3072.b1	GE294329
61938285	CCAH3072.g1	GE294330
61938286	CCAH3073.b1	GE294331
61938287	CCAH3073.g1	GE294332
61938288	CCAH3074.b1	GE294333
61938289	CCAH3075.b1	GE294334
61938290	CCAH3075.g1	GE294335
61938291	CCAH3076.b1	GE294336
61938292	CCAH3077.b1	GE294337
61938293	CCAH3077.g1	GE294338
61938294	CCAH3078.b1	GE294339
61938295	CCAH3080.b1	GE294340
61938296	CCAH3080.g1	GE294341
61938297	CCAH3081.b1	GE294342
61938298	CCAH3082.b1	GE294343
61938299	CCAH3083.b1	GE294344
61938300	CCAH3083.g1	GE294345
61938301	CCAH3084.b1	GE294346
61938302	CCAH3084.g1	GE294347
61938303	CCAH3085.b1	GE294348
61938304	CCAH3086.b1	GE294349
61938305	CCAH3086.g1	GE294350
61938306	CCAH3087.b1	GE294351
61938307	CCAH3087.g1	GE294352
61938308	CCAH3090.b1	GE294353
61938309	CCAH3091.b1	GE294354
61938310	CCAH3091.g1	GE294355
61938311	CCAH3092.b1	GE294356
61938312	CCAH3092.g1	GE294357
61938313	CCAH3093.b1	GE294358
61938314	CCAH3093.g1	GE294359
61938315	CCAH3094.b1	GE294360
61938316	CCAH3094.g1	GE294361
61938317	CCAH3095.b1	GE294362
61938318	CCAH3096.b1	GE294363
61938319	CCAH3097.b1	GE294364
61938320	CCAH3098.b1	GE294365
61938321	CCAH3100.b1	GE294366
61938322	CCAH3101.b1	GE294367
61938323	CCAH3102.b1	GE294368
61938324	CCAH3104.b1	GE294369
61938325	CCAH3105.b1	GE294370
61938326	CCAH3107.b1	GE294371
61938327	CCAH3107.g1	GE294372

61938328	CCAH3108.b1	GE294373
61938329	CCAH3109.b1	GE294374
61938330	CCAH3109.g1	GE294375
61938331	CCAH3110.b1	GE294376
61938332	CCAH3110.g1	GE294377
61938333	CCAH3111.b1	GE294378
61938334	CCAH3112.b1	GE294379
61938335	CCAH3112.g1	GE294380
61938336	CCAH3113.b1	GE294381
61938337	CCAH3114.b1	GE294382
61938338	CCAH3114.g1	GE294383
61938339	CCAH3116.b1	GE294384
61938340	CCAH3117.b1	GE294385
61938341	CCAH3117.g1	GE294386
61938342	CCAH3118.b1	GE294387
61938343	CCAH3119.b1	GE294388
61938344	CCAH3119.g1	GE294389
61938345	CCAH3120.b1	GE294390
61938346	CCAH3120.g1	GE294391
61938347	CCAH3121.b1	GE294392
61938348	CCAH3122.b1	GE294393
61938349	CCAH3123.b1	GE294394
61938350	CCAH3124.b1	GE294395
61938351	CCAH3124.g1	GE294396
61938352	CCAH3125.b1	GE294397
61938353	CCAH3125.g1	GE294398
61938354	CCAH3126.b1	GE294399
61938355	CCAH3126.g1	GE294400
61938356	CCAH3127.b1	GE294401
61938357	CCAH3127.g1	GE294402
61938358	CCAH3128.b1	GE294403
61938359	CCAH3129.b1	GE294404
61938360	CCAH3129.g1	GE294405
61938361	CCAH3130.b1	GE294406
61938362	CCAH3131.b1	GE294407
61938363	CCAH3132.b1	GE294408
61938364	CCAH3132.g1	GE294409
61938365	CCAH3133.b1	GE294410
61938366	CCAH3133.g1	GE294411
61938367	CCAH3134.b1	GE294412
61938368	CCAH3134.g1	GE294413
61938369	CCAH3135.b1	GE294414
61938370	CCAH3136.b1	GE294415
61938371	CCAH3137.b1	GE294416
61938372	CCAH3139.b1	GE294417
61938373	CCAH3139.g1	GE294418
61938374	CCAH3140.b1	GE294419
61938375	CCAH3140.g1	GE294420
61938376	CCAH3142.b1	GE294421
61938377	CCAH3142.g1	GE294422
61938378	CCAH3143.b1	GE294423
61938379	CCAH3143.g1	GE294424
61938380	CCAH3144.b1	GE294425
61938381	CCAH3145.b1	GE294426
61938382	CCAH3146.b1	GE294427
61938383	CCAH3147.b1	GE294428
61938384	CCAH3147.g1	GE294429
61938385	CCAH3148.b1	GE294430
61938386	CCAH3148.g1	GE294431
61938387	CCAH3149.b1	GE294432
61938388	CCAH3149.g1	GE294433
61938389	CCAH3150.b1	GE294434
61938390	CCAH3151.b1	GE294435

61938391	CCAH3151.g1	GE294436
61938392	CCAH3153.b1	GE294437
61938393	CCAH3154.b1	GE294438
61938394	CCAH3154.g1	GE294439
61938395	CCAH3155.b1	GE294440
61938396	CCAH3155.g1	GE294441
61938397	CCAH3156.b1	GE294442
61938398	CCAH3157.b1	GE294443
61938399	CCAH3157.g1	GE294444
61938400	CCAH3158.b1	GE294445
61938401	CCAH3159.b1	GE294446
61938402	CCAH3160.b1	GE294447
61938403	CCAH3160.g1	GE294448
61938404	CCAH3162.b1	GE294449
61938405	CCAH3163.b1	GE294450
61938406	CCAH3164.b1	GE294451
61938407	CCAH3164.g1	GE294452
61938408	CCAH3165.b1	GE294453
61938409	CCAH3166.g1	GE294454
61938410	CCAH3167.b1	GE294455
61938411	CCAH3167.g1	GE294456
61938412	CCAH3168.b1	GE294457
61938413	CCAH3169.b1	GE294458
61938414	CCAH3169.g1	GE294459
61938415	CCAH3170.b1	GE294460
61938416	CCAH3171.b1	GE294461
61938417	CCAH3172.b1	GE294462
61938418	CCAH3173.b1	GE294463
61938419	CCAH3173.g1	GE294464
61938420	CCAH3174.b1	GE294465
61938421	CCAH3175.b1	GE294466
61938422	CCAH3175.g1	GE294467
61938423	CCAH3176.b1	GE294468
61938424	CCAH3177.b1	GE294469
61938425	CCAH3178.b1	GE294470
61938426	CCAH3178.g1	GE294471
61938427	CCAH3179.b1	GE294472
61938428	CCAH3180.b1	GE294473
61938429	CCAH3180.g1	GE294474
61938430	CCAH3181.b1	GE294475
61938431	CCAH3182.b1	GE294476
61938432	CCAH3182.g1	GE294477
61938433	CCAH3183.b1	GE294478
61938434	CCAH3183.g1	GE294479
61938435	CCAH3184.b1	GE294480
61938436	CCAH3184.g1	GE294481
61938437	CCAH3185.b1	GE294482
61938438	CCAH3185.g1	GE294483
61938439	CCAH3186.b1	GE294484
61938440	CCAH3186.g1	GE294485
61938441	CCAH3187.b1	GE294486
61938442	CCAH3187.g1	GE294487
61938443	CCAH3188.b1	GE294488
61938444	CCAH3190.b1	GE294489
61938445	CCAH3193.b1	GE294490
61938446	CCAH3193.g1	GE294491
61938447	CCAH3194.b1	GE294492
61938448	CCAH3194.g1	GE294493
61938449	CCAH3196.b1	GE294494
61938450	CCAH3196.g1	GE294495
61938451	CCAH3197.b1	GE294496
61938452	CCAH3198.g1	GE294497
61938453	CCAH3199.b1	GE294498

61938454	CCAH3201.g1	GE294499
61938455	CCAH3204.b1	GE294500
61938456	CCAH3204.g1	GE294501
61938457	CCAH3205.b1	GE294502
61938458	CCAH3205.g1	GE294503
61938459	CCAH3206.b1	GE294504
61938460	CCAH3206.g1	GE294505
61938461	CCAH3208.b1	GE294506
61938462	CCAH3208.g1	GE294507
61938463	CCAH3209.b1	GE294508
61938464	CCAH3210.b1	GE294509
61938465	CCAH3212.b1	GE294510
61938466	CCAH3212.g1	GE294511
61938467	CCAH3213.b1	GE294512
61938468	CCAH3214.b1	GE294513
61938469	CCAH3215.b1	GE294514
61938470	CCAH3215.g1	GE294515
61938471	CCAH3216.b1	GE294516
61938472	CCAH3216.g1	GE294517
61938473	CCAH3218.g1	GE294518
61938474	CCAH3219.b1	GE294519
61938475	CCAH3219.g1	GE294520
61938476	CCAH3220.b1	GE294521
61938477	CCAH3220.g1	GE294522
61938478	CCAH3221.b1	GE294523
61938479	CCAH3222.b1	GE294524
61938480	CCAH3222.g1	GE294525
61938481	CCAH3223.b1	GE294526
61938482	CCAH3223.g1	GE294527
61938483	CCAH3224.b1	GE294528
61938484	CCAH3225.b1	GE294529
61938485	CCAH3225.g1	GE294530
61938486	CCAH3228.b1	GE294531
61938487	CCAH3228.g1	GE294532
61938488	CCAH3229.g1	GE294533
61938489	CCAH3230.b1	GE294534
61938490	CCAH3230.g1	GE294535
61938491	CCAH3231.b1	GE294536
61938492	CCAH3231.g1	GE294537
61938493	CCAH3232.b1	GE294538
61938494	CCAH3233.b1	GE294539
61938495	CCAH3233.g1	GE294540
61938496	CCAH3234.b1	GE294541
61938497	CCAH3235.b1	GE294542
61938498	CCAH3235.g1	GE294543
61938499	CCAH3236.b1	GE294544
61938500	CCAH3236.g1	GE294545
61938501	CCAH3237.b1	GE294546
61938502	CCAH3238.b1	GE294547
61938503	CCAH3238.g1	GE294548
61938504	CCAH3239.b1	GE294549
61938505	CCAH3239.g1	GE294550
61938506	CCAH3240.b1	GE294551
61938507	CCAH3241.b1	GE294552
61938508	CCAH3241.g1	GE294553
61938509	CCAH3242.b1	GE294554
61938510	CCAH3243.b1	GE294555
61938511	CCAH3243.g1	GE294556
61938512	CCAH3244.b1	GE294557
61938513	CCAH3244.g1	GE294558
61938514	CCAH3245.b1	GE294559
61938515	CCAH3245.g1	GE294560
61938516	CCAH3247.b1	GE294561

61938517	CCAH3247.g1	GE294562
61938518	CCAH3248.b1	GE294563
61938519	CCAH3248.g1	GE294564
61938520	CCAH3249.b1	GE294565
61938521	CCAH3249.g1	GE294566
61938522	CCAH3250.b1	GE294567
61938523	CCAH3250.g1	GE294568
61938524	CCAH3252.b1	GE294569
61938525	CCAH3253.b1	GE294570
61938526	CCAH3254.b1	GE294571
61938527	CCAH3255.b1	GE294572
61938528	CCAH3255.g1	GE294573
61938529	CCAH3256.b1	GE294574
61938530	CCAH3257.b1	GE294575
61938531	CCAH3257.g1	GE294576
61938532	CCAH3258.b1	GE294577
61938533	CCAH3258.g1	GE294578
61938534	CCAH3259.b1	GE294579
61938535	CCAH3260.b1	GE294580
61938536	CCAH3261.b1	GE294581
61938537	CCAH3262.b1	GE294582
61938538	CCAH3262.g1	GE294583
61938539	CCAH3263.b1	GE294584
61938540	CCAH3264.b1	GE294585
61938541	CCAH3264.g1	GE294586
61938542	CCAH3265.b1	GE294587
61938543	CCAH3265.g1	GE294588
61938544	CCAH3266.b1	GE294589
61938545	CCAH3266.g1	GE294590
61938546	CCAH3267.b1	GE294591
61938547	CCAH3267.g1	GE294592
61938548	CCAH3268.b1	GE294593
61938549	CCAH3268.g1	GE294594
61938550	CCAH3269.g1	GE294595
61938551	CCAH3270.b1	GE294596
61938552	CCAH3270.g1	GE294597
61938553	CCAH3271.b1	GE294598
61938554	CCAH3271.g1	GE294599
61938555	CCAH3272.b1	GE294600
61938556	CCAH3272.g1	GE294601
61938557	CCAH3273.b1	GE294602
61938558	CCAH3274.g1	GE294603
61938559	CCAH3275.b1	GE294604
61938560	CCAH3275.g1	GE294605
61938561	CCAH3276.b1	GE294606
61938562	CCAH3277.b1	GE294607
61938563	CCAH3277.g1	GE294608
61938564	CCAH3278.b1	GE294609
61938565	CCAH3278.g1	GE294610
61938566	CCAH3279.b1	GE294611
61938567	CCAH3280.b1	GE294612
61938568	CCAH3280.g1	GE294613
61938569	CCAH3283.b1	GE294614
61938570	CCAH3284.b1	GE294615
61938571	CCAH3285.b1	GE294616
61938572	CCAH3285.g1	GE294617
61938573	CCAH3286.b1	GE294618
61938574	CCAH3286.g1	GE294619
61938575	CCAH3287.b1	GE294620
61938576	CCAH3288.b1	GE294621
61938577	CCAH3288.g1	GE294622
61938578	CCAH3289.b1	GE294623
61938579	CCAH3290.b1	GE294624

61938580	CCAH3292.b1	GE294625
61938581	CCAH3293.b1	GE294626
61938582	CCAH3296.b1	GE294627
61938583	CCAH3297.b1	GE294628
61938584	CCAH3297.g1	GE294629
61938585	CCAH3299.b1	GE294630
61938586	CCAH3299.g1	GE294631
61938587	CCAH3301.b1	GE294632
61938588	CCAH3302.b1	GE294633
61938589	CCAH3302.g1	GE294634
61938590	CCAH3303.b1	GE294635
61938591	CCAH3304.b1	GE294636
61938592	CCAH3304.g1	GE294637
61938593	CCAH3305.b1	GE294638
61938594	CCAH3305.g1	GE294639
61938595	CCAH3306.b1	GE294640
61938596	CCAH3308.b1	GE294641
61938597	CCAH3309.b1	GE294642
61938598	CCAH3309.g1	GE294643
61938599	CCAH3310.b1	GE294644
61938600	CCAH3310.g1	GE294645
61938601	CCAH3311.b1	GE294646
61938602	CCAH3312.b1	GE294647
61938603	CCAH3313.b1	GE294648
61938604	CCAH3313.g1	GE294649
61938605	CCAH3314.b1	GE294650
61938606	CCAH3315.b1	GE294651
61938607	CCAH3315.g1	GE294652
61938608	CCAH3316.b1	GE294653
61938609	CCAH3316.g1	GE294654
61938610	CCAH3318.b1	GE294655
61938611	CCAH3319.b1	GE294656
61938612	CCAH3319.g1	GE294657
61938613	CCAH3321.b1	GE294658
61938614	CCAH3322.b1	GE294659
61938615	CCAH3322.g1	GE294660
61938616	CCAH3323.b1	GE294661
61938617	CCAH3323.g1	GE294662
61938618	CCAH3324.b1	GE294663
61938619	CCAH3324.g1	GE294664
61938620	CCAH3325.b1	GE294665
61938621	CCAH3325.g1	GE294666
61938622	CCAH3326.b1	GE294667
61938623	CCAH3326.g1	GE294668
61938624	CCAH3328.b1	GE294669
61938625	CCAH3328.g1	GE294670
61938626	CCAH3329.b1	GE294671
61938627	CCAH3329.g1	GE294672
61938628	CCAH3330.b1	GE294673
61938629	CCAH3331.b1	GE294674
61938630	CCAH3331.g1	GE294675
61938631	CCAH3332.b1	GE294676
61938632	CCAH3333.b1	GE294677
61938633	CCAH3334.b1	GE294678
61938634	CCAH3334.g1	GE294679
61938635	CCAH3335.b1	GE294680
61938636	CCAH3336.b1	GE294681
61938637	CCAH3337.b1	GE294682
61938638	CCAH3337.g1	GE294683
61938639	CCAH3338.b1	GE294684
61938640	CCAH3338.g1	GE294685
61938641	CCAH3339.b1	GE294686
61938642	CCAH3339.g1	GE294687

61938643	CCAH3340.b1	GE294688
61938644	CCAH3340.g1	GE294689
61938645	CCAH3341.b1	GE294690
61938646	CCAH3341.g1	GE294691
61938647	CCAH3343.b1	GE294692
61938648	CCAH3344.b1	GE294693
61938649	CCAH3344.g1	GE294694
61938650	CCAH3345.b1	GE294695
61938651	CCAH3346.b1	GE294696
61938652	CCAH3346.g1	GE294697
61938653	CCAH3347.b1	GE294698
61938654	CCAH3347.g1	GE294699
61938655	CCAH3348.b1	GE294700
61938656	CCAH3348.g1	GE294701
61938657	CCAH3349.b1	GE294702
61938658	CCAH3349.g1	GE294703
61938659	CCAH3350.b1	GE294704
61938660	CCAH3351.b1	GE294705
61938661	CCAH3352.b1	GE294706
61938662	CCAH3352.g1	GE294707
61938663	CCAH3353.b1	GE294708
61938664	CCAH3353.g1	GE294709
61938665	CCAH3354.b1	GE294710
61938666	CCAH3355.b1	GE294711
61938667	CCAH3355.g1	GE294712
61938668	CCAH3356.b1	GE294713
61938669	CCAH3357.b1	GE294714
61938670	CCAH3358.b1	GE294715
61938671	CCAH3359.b1	GE294716
61938672	CCAH3359.g1	GE294717
61938673	CCAH3361.b1	GE294718
61938674	CCAH3362.b1	GE294719
61938675	CCAH3363.b1	GE294720
61938676	CCAH3364.b1	GE294721
61938677	CCAH3364.g1	GE294722
61938678	CCAH3365.b1	GE294723
61938679	CCAH3365.g1	GE294724
61938680	CCAH3366.b1	GE294725
61938681	CCAH3366.g1	GE294726
61938682	CCAH3367.b1	GE294727
61938683	CCAH3367.g1	GE294728
61938684	CCAH3368.b1	GE294729
61938685	CCAH3369.b1	GE294730
61938686	CCAH3369.g1	GE294731
61938687	CCAH3370.b1	GE294732
61938688	CCAH3371.b1	GE294733
61938689	CCAH3371.g1	GE294734
61938690	CCAH3372.g1	GE294735
61938691	CCAH3374.b1	GE294736
61938692	CCAH3374.g1	GE294737
61938693	CCAH3375.g1	GE294738
61938694	CCAH3376.b1	GE294739
61938695	CCAH3376.g1	GE294740
61938696	CCAH3377.b1	GE294741
61938697	CCAH3377.g1	GE294742
61938698	CCAH3378.b1	GE294743
61938699	CCAH3379.b1	GE294744
61938700	CCAH3380.b1	GE294745
61938701	CCAH3381.b1	GE294746
61938702	CCAH3381.g1	GE294747
61938703	CCAH3382.b1	GE294748
61938704	CCAH3382.g1	GE294749
61938705	CCAH3383.b1	GE294750

61938706	CCAH3383.g1	GE294751
61938707	CCAH3384.b1	GE294752
61938708	CCAH3384.g1	GE294753
61938709	CCAH3385.b1	GE294754
61938710	CCAH3385.g1	GE294755
61938711	CCAH3386.b1	GE294756
61938712	CCAH3386.g1	GE294757
61938713	CCAH3387.b1	GE294758
61938714	CCAH3387.g1	GE294759
61938715	CCAH3388.b1	GE294760
61938716	CCAH3389.b1	GE294761
61938717	CCAH3389.g1	GE294762
61938718	CCAH3390.b1	GE294763
61938719	CCAH3390.g1	GE294764
61938720	CCAH3391.b1	GE294765
61938721	CCAH3391.g1	GE294766
61938722	CCAH3392.b1	GE294767
61938723	CCAH3393.b1	GE294768
61938724	CCAH3393.g1	GE294769
61938725	CCAH3395.b1	GE294770
61938726	CCAH3395.g1	GE294771
61938727	CCAH3396.b1	GE294772
61938728	CCAH3397.b1	GE294773
61938729	CCAH3397.g1	GE294774
61938730	CCAH3398.b1	GE294775
61938731	CCAH3399.b1	GE294776
61938732	CCAH3399.g1	GE294777
61938733	CCAH3400.b1	GE294778
61938734	CCAH3401.b1	GE294779
61938735	CCAH3401.g1	GE294780
61938736	CCAH3402.b1	GE294781
61938737	CCAH3402.g1	GE294782
61938738	CCAH3403.b1	GE294783
61938739	CCAH3404.b1	GE294784
61938740	CCAH3404.g1	GE294785
61938741	CCAH3405.b1	GE294786
61938742	CCAH3405.g1	GE294787
61938743	CCAH3406.b1	GE294788
61938744	CCAH3406.g1	GE294789
61938745	CCAH3407.b1	GE294790
61938746	CCAH3408.b1	GE294791
61938747	CCAH3409.b1	GE294792
61938748	CCAH3409.g1	GE294793
61938749	CCAH3410.b1	GE294794
61938750	CCAH3410.g1	GE294795
61938751	CCAH3411.b1	GE294796
61938752	CCAH3411.g1	GE294797
61938753	CCAH3414.b1	GE294798
61938754	CCAH3415.b1	GE294799
61938755	CCAH3417.b1	GE294800
61938756	CCAH3417.g1	GE294801
61938757	CCAH3418.b1	GE294802
61938758	CCAH3418.g1	GE294803
61938759	CCAH3419.b1	GE294804
61938760	CCAH3420.b1	GE294805
61938761	CCAH3421.b1	GE294806
61938762	CCAH3422.b1	GE294807
61938763	CCAH3424.b1	GE294808
61938764	CCAH3425.b1	GE294809
61938765	CCAH3425.g1	GE294810
61938766	CCAH3426.b1	GE294811
61938767	CCAH3427.b1	GE294812
61938768	CCAH3428.b1	GE294813

61938769	CCAH3430.b1	GE294814
61938770	CCAH3430.g1	GE294815
61938771	CCAH3431.b1	GE294816
61938772	CCAH3431.g1	GE294817
61938773	CCAH3432.b1	GE294818
61938774	CCAH3432.g1	GE294819
61938775	CCAH3433.b1	GE294820
61938776	CCAH3433.g1	GE294821
61938777	CCAH3434.b1	GE294822
61938778	CCAH3434.g1	GE294823
61938779	CCAH3436.b1	GE294824
61938780	CCAH3437.b1	GE294825
61938781	CCAH3437.g1	GE294826
61938782	CCAH3438.b1	GE294827
61938783	CCAH3439.b1	GE294828
61938784	CCAH3439.g1	GE294829
61938785	CCAH3440.b1	GE294830
61938786	CCAH3441.g1	GE294831
61938787	CCAH3442.b1	GE294832
61938788	CCAH3442.g1	GE294833
61938789	CCAH3443.b1	GE294834
61938790	CCAH3443.g1	GE294835
61938791	CCAH3446.b1	GE294836
61938792	CCAH3446.g1	GE294837
61938793	CCAH3447.b1	GE294838
61938794	CCAH3447.g1	GE294839
61938795	CCAH3448.b1	GE294840
61938796	CCAH3449.b1	GE294841
61938797	CCAH3450.b1	GE294842
61938798	CCAH3450.g1	GE294843
61938799	CCAH3451.g1	GE294844
61938800	CCAH3452.b1	GE294845
61938801	CCAH3453.b1	GE294846
61938802	CCAH3455.b1	GE294847
61938803	CCAH3457.b1	GE294848
61938804	CCAH3457.g1	GE294849
61938805	CCAH3458.b1	GE294850
61938806	CCAH3458.g1	GE294851
61938807	CCAH3459.b1	GE294852
61938808	CCAH3460.b1	GE294853
61938809	CCAH3460.g1	GE294854
61938810	CCAH3461.b1	GE294855
61938811	CCAH3461.g1	GE294856
61938812	CCAH3462.b1	GE294857
61938813	CCAH3462.g1	GE294858
61938814	CCAH3463.b1	GE294859
61938815	CCAH3464.b1	GE294860
61938816	CCAH3464.g1	GE294861
61938817	CCAH3465.b1	GE294862
61938818	CCAH3466.b1	GE294863
61938819	CCAH3466.g1	GE294864
61938820	CCAH3467.b1	GE294865
61938821	CCAH3468.b1	GE294866
61938822	CCAH3468.g1	GE294867
61938823	CCAH3469.b1	GE294868
61938824	CCAH3469.g1	GE294869
61938825	CCAH3470.g1	GE294870
61938826	CCAH3471.g1	GE294871
61938827	CCAH3472.b1	GE294872
61938828	CCAH3472.g1	GE294873
61938829	CCAH3473.b1	GE294874
61938830	CCAH3474.b1	GE294875
61938831	CCAH3474.g1	GE294876

61938832	CCAH3475.b1	GE294877
61938833	CCAH3475.g1	GE294878
61938834	CCAH3476.b1	GE294879
61938835	CCAH3476.g1	GE294880
61938836	CCAH3477.b1	GE294881
61938837	CCAH3477.g1	GE294882
61938838	CCAH3478.b1	GE294883
61938839	CCAH3478.g1	GE294884
61938840	CCAH3479.b1	GE294885
61938841	CCAH3479.g1	GE294886
61938842	CCAH3480.b1	GE294887
61938843	CCAH3480.g1	GE294888
61938844	CCAH3481.b1	GE294889
61938845	CCAH3482.b1	GE294890
61938846	CCAH3482.g1	GE294891
61938847	CCAH3483.b1	GE294892
61938848	CCAH3483.g1	GE294893
61938849	CCAH3484.b1	GE294894
61938850	CCAH3485.b1	GE294895
61938851	CCAH3485.g1	GE294896
61938852	CCAH3486.b1	GE294897
61938853	CCAH3486.g1	GE294898
61938854	CCAH3487.g1	GE294899
61938855	CCAH3488.b1	GE294900
61938856	CCAH3488.g1	GE294901
61938857	CCAH3489.b1	GE294902
61938858	CCAH3490.b1	GE294903
61938859	CCAH3490.g1	GE294904
61938860	CCAH3491.b1	GE294905
61938861	CCAH3491.g1	GE294906
61938862	CCAH3492.b1	GE294907
61938863	CCAH3493.b1	GE294908
61938864	CCAH3493.g1	GE294909
61938865	CCAH3494.b1	GE294910
61938866	CCAH3495.b1	GE294911
61938867	CCAH3495.g1	GE294912
61938868	CCAH3496.b1	GE294913
61938869	CCAH3496.g1	GE294914
61938870	CCAH3497.b1	GE294915
61938871	CCAH3498.b1	GE294916
61938872	CCAH3499.b1	GE294917
61938873	CCAH3500.b1	GE294918
61938874	CCAH3500.g1	GE294919
61938875	CCAH3501.b1	GE294920
61938876	CCAH3501.g1	GE294921
61938877	CCAH3502.b1	GE294922
61938878	CCAH3504.b1	GE294923
61938879	CCAH3504.g1	GE294924
61938880	CCAH3505.b1	GE294925
61938881	CCAH3506.b1	GE294926
61938882	CCAH3507.b1	GE294927
61938883	CCAH3507.g1	GE294928
61938884	CCAH3508.b1	GE294929
61938885	CCAH3508.g1	GE294930
61938886	CCAH3509.g1	GE294931
61938887	CCAH3510.b1	GE294932
61938888	CCAH3510.g1	GE294933
61938889	CCAH3511.b1	GE294934
61938890	CCAH3512.b1	GE294935
61938891	CCAH3513.b1	GE294936
61938892	CCAH3513.g1	GE294937
61938893	CCAH3514.b1	GE294938
61938894	CCAH3514.g1	GE294939

61938895	CCAH3515.b1	GE294940
61938896	CCAH3516.b1	GE294941
61938897	CCAH3517.b1	GE294942
61938898	CCAH3517.g1	GE294943
61938899	CCAH3518.b1	GE294944
61938900	CCAH3519.b1	GE294945
61938901	CCAH3519.g1	GE294946
61938902	CCAH3520.b1	GE294947
61938903	CCAH3520.g1	GE294948
61938904	CCAH3521.b1	GE294949
61938905	CCAH3521.g1	GE294950
61938906	CCAH3522.b1	GE294951
61938907	CCAH3522.g1	GE294952
61938908	CCAH3523.b1	GE294953
61938909	CCAH3523.g1	GE294954
61938910	CCAH3524.b1	GE294955
61938911	CCAH3525.b1	GE294956
61938912	CCAH3526.b1	GE294957
61938913	CCAH3527.b1	GE294958
61938914	CCAH3527.g1	GE294959
61938915	CCAH3529.b1	GE294960
61938916	CCAH3530.b1	GE294961
61938917	CCAH3531.b1	GE294962
61938918	CCAH3531.g1	GE294963
61938919	CCAH3532.b1	GE294964
61938920	CCAH3533.b1	GE294965
61938921	CCAH3533.g1	GE294966
61938922	CCAH3534.b1	GE294967
61938923	CCAH3534.g1	GE294968
61938924	CCAH3535.b1	GE294969
61938925	CCAH3535.g1	GE294970
61938926	CCAH3536.b1	GE294971
61938927	CCAH3536.g1	GE294972
61938928	CCAH3537.b1	GE294973
61938929	CCAH3538.b1	GE294974
61938930	CCAH3538.g1	GE294975
61938931	CCAH3539.b1	GE294976
61938932	CCAH3539.g1	GE294977
61938933	CCAH3541.b1	GE294978
61938934	CCAH3541.g1	GE294979
61938935	CCAH3542.b1	GE294980
61938936	CCAH3542.g1	GE294981
61938937	CCAH3543.b1	GE294982
61938938	CCAH3543.g1	GE294983
61938939	CCAH3544.b1	GE294984
61938940	CCAH3544.g1	GE294985
61938941	CCAH3545.b1	GE294986
61938942	CCAH3546.g1	GE294987
61938943	CCAH3547.b1	GE294988
61938944	CCAH3547.g1	GE294989
61938945	CCAH3548.b1	GE294990
61938946	CCAH3549.b1	GE294991
61938947	CCAH3549.g1	GE294992
61938948	CCAH3551.b1	GE294993
61938949	CCAH3551.g1	GE294994
61938950	CCAH3552.b1	GE294995
61938951	CCAH3552.g1	GE294996
61938952	CCAH3553.b1	GE294997
61938953	CCAH3554.g1	GE294998
61938954	CCAH3555.b1	GE294999
61938955	CCAH3555.g1	GE295000
61938956	CCAH3556.b1	GE295001
61938957	CCAH3557.b1	GE295002

61938958	CCAH3557.g1	GE295003
61938959	CCAH3558.b1	GE295004
61938960	CCAH3558.g1	GE295005
61938961	CCAH3559.b1	GE295006
61938962	CCAH3561.b1	GE295007
61938963	CCAH3562.b1	GE295008
61938964	CCAH3562.g1	GE295009
61938965	CCAH3563.b1	GE295010
61938966	CCAH3564.b1	GE295011
61938967	CCAH3564.g1	GE295012
61938968	CCAH3565.b1	GE295013
61938969	CCAH3565.g1	GE295014
61938970	CCAH3566.b1	GE295015
61938971	CCAH3566.g1	GE295016
61938972	CCAH3567.b1	GE295017
61938973	CCAH3568.b1	GE295018
61938974	CCAH3568.g1	GE295019
61938975	CCAH3569.b1	GE295020
61938976	CCAH3570.b1	GE295021
61938977	CCAH3571.b1	GE295022
61938978	CCAH3572.b1	GE295023
61938979	CCAH3573.b1	GE295024
61938980	CCAH3574.b1	GE295025
61938981	CCAH3574.g1	GE295026
61938982	CCAH3575.b1	GE295027
61938983	CCAH3575.g1	GE295028
61938984	CCAH3576.b1	GE295029
61938985	CCAH3576.g1	GE295030
61938986	CCAH3577.b1	GE295031
61938987	CCAH3578.b1	GE295032
61938988	CCAH3579.b1	GE295033
61938989	CCAH3579.g1	GE295034
61938990	CCAH3580.b1	GE295035
61938991	CCAH3581.b1	GE295036
61938992	CCAH3581.g1	GE295037
61938993	CCAH3582.b1	GE295038
61938994	CCAH3582.g1	GE295039
61938995	CCAH3583.b1	GE295040
61938996	CCAH3584.b1	GE295041
61938997	CCAH3584.g1	GE295042
61938998	CCAH3585.b1	GE295043
61938999	CCAH3585.g1	GE295044
61939000	CCAH3586.b1	GE295045
61939001	CCAH3586.g1	GE295046
61939002	CCAH3587.b1	GE295047
61939003	CCAH3587.g1	GE295048
61939004	CCAH3588.b1	GE295049
61939005	CCAH3589.b1	GE295050
61939006	CCAH3590.b1	GE295051
61939007	CCAH3590.g1	GE295052
61939008	CCAH3591.b1	GE295053
61939009	CCAH3591.g1	GE295054
61939010	CCAH3592.b1	GE295055
61939011	CCAH3592.g1	GE295056
61939012	CCAH3593.b1	GE295057
61939013	CCAH3594.b1	GE295058
61939014	CCAH3595.b1	GE295059
61939015	CCAH3595.g1	GE295060
61939016	CCAH3596.b1	GE295061
61939017	CCAH3597.b1	GE295062
61939018	CCAH3597.g1	GE295063
61939019	CCAH3598.b1	GE295064
61939020	CCAH3599.b1	GE295065

61939021	CCAH3600.b1	GE295066
61939022	CCAH3601.b1	GE295067
61939023	CCAH3602.b1	GE295068
61939024	CCAH3604.b1	GE295069
61939025	CCAH3607.g1	GE295070
61939026	CCAH3608.b1	GE295071
61939027	CCAH3609.b1	GE295072
61939028	CCAH3609.g1	GE295073
61939029	CCAH3610.b1	GE295074
61939030	CCAH3611.b1	GE295075
61939031	CCAH3612.b1	GE295076
61939032	CCAH3613.b1	GE295077
61939033	CCAH3614.b1	GE295078
61939034	CCAH3615.b1	GE295079
61939035	CCAH3616.b1	GE295080
61939036	CCAH3616.g1	GE295081
61939037	CCAH3617.b1	GE295082
61939038	CCAH3617.g1	GE295083
61939039	CCAH3618.b1	GE295084
61939040	CCAH3618.g1	GE295085
61939041	CCAH3619.b1	GE295086
61939042	CCAH3619.g1	GE295087
61939043	CCAH3622.b1	GE295088
61939044	CCAH3622.g1	GE295089
61939045	CCAH3623.b1	GE295090
61939046	CCAH3623.g1	GE295091
61939047	CCAH3624.b1	GE295092
61939048	CCAH3625.b1	GE295093
61939049	CCAH3625.g1	GE295094
61939050	CCAH3626.b1	GE295095
61939051	CCAH3626.g1	GE295096
61939052	CCAH3628.b1	GE295097
61939053	CCAH3629.b1	GE295098
61939054	CCAH3631.b1	GE295099
61939055	CCAH3633.b1	GE295100
61939056	CCAH3633.g1	GE295101
61939057	CCAH3634.b1	GE295102
61939058	CCAH3634.g1	GE295103
61939059	CCAH3635.b1	GE295104
61939060	CCAH3635.g1	GE295105
61939061	CCAH3636.b1	GE295106
61939062	CCAH3638.b1	GE295107
61939063	CCAH3638.g1	GE295108
61939064	CCAH3639.b1	GE295109
61939065	CCAH3639.g1	GE295110
61939066	CCAH3640.b1	GE295111
61939067	CCAH3640.g1	GE295112
61939068	CCAH3642.b1	GE295113
61939069	CCAH3642.g1	GE295114
61939070	CCAH3644.b1	GE295115
61939071	CCAH3645.b1	GE295116
61939072	CCAH3645.g1	GE295117
61939073	CCAH3646.b1	GE295118
61939074	CCAH3646.g1	GE295119
61939075	CCAH3647.b1	GE295120
61939076	CCAH3647.g1	GE295121
61939077	CCAH3648.b1	GE295122
61939078	CCAH3648.g1	GE295123
61939079	CCAH3649.b1	GE295124
61939080	CCAH3649.g1	GE295125
61939081	CCAH3650.b1	GE295126
61939082	CCAH3652.b1	GE295127
61939083	CCAH3652.g1	GE295128

61939084	CCAH3653.b1	GE295129
61939085	CCAH3653.g1	GE295130
61939086	CCAH3654.b1	GE295131
61939087	CCAH3655.b1	GE295132
61939088	CCAH3655.g1	GE295133
61939089	CCAH3656.b1	GE295134
61939090	CCAH3656.g1	GE295135
61939091	CCAH3658.b1	GE295136
61939092	CCAH3659.b1	GE295137
61939093	CCAH3659.g1	GE295138
61939094	CCAH3660.b1	GE295139
61939095	CCAH3660.g1	GE295140
61939096	CCAH3661.b1	GE295141
61939097	CCAH3661.g1	GE295142
61939098	CCAH3662.b1	GE295143
61939099	CCAH3662.g1	GE295144
61939100	CCAH3663.b1	GE295145
61939101	CCAH3663.g1	GE295146
61939102	CCAH3664.b1	GE295147
61939103	CCAH3664.g1	GE295148
61939104	CCAH3666.b1	GE295149
61939105	CCAH3666.g1	GE295150
61939106	CCAH3667.g1	GE295151
61939107	CCAH3668.b1	GE295152
61939108	CCAH3668.g1	GE295153
61939109	CCAH3669.b1	GE295154
61939110	CCAH3669.g1	GE295155
61939111	CCAH3670.b1	GE295156
61939112	CCAH3670.g1	GE295157
61939113	CCAH3671.b1	GE295158
61939114	CCAH3671.g1	GE295159
61939115	CCAH3672.b1	GE295160
61939116	CCAH3673.b1	GE295161
61939117	CCAH3674.b1	GE295162
61939118	CCAH3677.b1	GE295163
61939119	CCAH3677.g1	GE295164
61939120	CCAH3679.b1	GE295165
61939121	CCAH3680.b1	GE295166
61939122	CCAH3681.b1	GE295167
61939123	CCAH3681.g1	GE295168
61939124	CCAH3682.b1	GE295169
61939125	CCAH3682.g1	GE295170
61939126	CCAH3683.b1	GE295171
61939127	CCAH3684.b1	GE295172
61939128	CCAH3685.b1	GE295173
61939129	CCAH3685.g1	GE295174
61939130	CCAH3686.b1	GE295175
61939131	CCAH3686.g1	GE295176
61939132	CCAH3687.b1	GE295177
61939133	CCAH3687.g1	GE295178
61939134	CCAH3688.b1	GE295179
61939135	CCAH3688.g1	GE295180
61939136	CCAH3689.g1	GE295181
61939137	CCAH3690.b1	GE295182
61939138	CCAH3690.g1	GE295183
61939139	CCAH3691.b1	GE295184
61939140	CCAH3691.g1	GE295185
61939141	CCAH3692.b1	GE295186
61939142	CCAH3692.g1	GE295187
61939143	CCAH3693.b1	GE295188
61939144	CCAH3693.g1	GE295189
61939145	CCAH3694.b1	GE295190
61939146	CCAH3695.b1	GE295191

61939147	CCAH3695.g1	GE295192
61939148	CCAH3696.b1	GE295193
61939149	CCAH3697.b1	GE295194
61939150	CCAH3698.b1	GE295195
61939151	CCAH3698.g1	GE295196
61939152	CCAH3700.b1	GE295197
61939153	CCAH3700.g1	GE295198
61939154	CCAH3702.b1	GE295199
61939155	CCAH3702.g1	GE295200
61939156	CCAH3703.b1	GE295201
61939157	CCAH3703.g1	GE295202
61939158	CCAH3704.g1	GE295203
61939159	CCAH3705.b1	GE295204
61939160	CCAH3706.b1	GE295205
61939161	CCAH3706.g1	GE295206
61939162	CCAH3707.b1	GE295207
61939163	CCAH3707.g1	GE295208
61939164	CCAH3708.b1	GE295209
61939165	CCAH3709.g1	GE295210
61939166	CCAH3710.b1	GE295211
61939167	CCAH3711.b1	GE295212
61939168	CCAH3712.b1	GE295213
61939169	CCAH3712.g1	GE295214
61939170	CCAH3713.b1	GE295215
61939171	CCAH3714.b1	GE295216
61939172	CCAH3715.b1	GE295217
61939173	CCAH3715.g1	GE295218
61939174	CCAH3716.b1	GE295219
61939175	CCAH3717.b1	GE295220
61939176	CCAH3717.g1	GE295221
61939177	CCAH3718.b1	GE295222
61939178	CCAH3718.g1	GE295223
61939179	CCAH3719.b1	GE295224
61939180	CCAH3720.b1	GE295225
61939181	CCAH3720.g1	GE295226
61939182	CCAH3721.b1	GE295227
61939183	CCAH3722.b1	GE295228
61939184	CCAH3723.b1	GE295229
61939185	CCAH3723.g1	GE295230
61939186	CCAH3724.b1	GE295231
61939187	CCAH3725.b1	GE295232
61939188	CCAH3725.g1	GE295233
61939189	CCAH3726.b1	GE295234
61939190	CCAH3726.g1	GE295235
61939191	CCAH3727.b1	GE295236
61939192	CCAH3728.b1	GE295237
61939193	CCAH3728.g1	GE295238
61939194	CCAH3729.b1	GE295239
61939195	CCAH3731.b1	GE295240
61939196	CCAH3732.b1	GE295241
61939197	CCAH3732.g1	GE295242
61939198	CCAH3733.b1	GE295243
61939199	CCAH3733.g1	GE295244
61939200	CCAH3735.b1	GE295245
61939201	CCAH3735.g1	GE295246
61939202	CCAH3736.b1	GE295247
61939203	CCAH3736.g1	GE295248
61939204	CCAH3738.b1	GE295249
61939205	CCAH3738.g1	GE295250
61939206	CCAH3739.b1	GE295251
61939207	CCAH3739.g1	GE295252
61939208	CCAH3742.b1	GE295253
61939209	CCAH3743.b1	GE295254

61939210	CCAH3743.g1	GE295255
61939211	CCAH3744.b1	GE295256
61939212	CCAH3744.g1	GE295257
61939213	CCAH3745.b1	GE295258
61939214	CCAH3745.g1	GE295259
61939215	CCAH3746.b1	GE295260
61939216	CCAH3746.g1	GE295261
61939217	CCAH3747.b1	GE295262
61939218	CCAH3748.b1	GE295263
61939219	CCAH3748.g1	GE295264
61939220	CCAH3749.b1	GE295265
61939221	CCAH3749.g1	GE295266
61939222	CCAH3750.b1	GE295267
61939223	CCAH3751.b1	GE295268
61939224	CCAH3751.g1	GE295269
61939225	CCAH3752.b1	GE295270
61939226	CCAH3752.g1	GE295271
61939227	CCAH3753.b1	GE295272
61939228	CCAH3753.g1	GE295273
61939229	CCAH3755.b1	GE295274
61939230	CCAH3755.g1	GE295275
61939231	CCAH3756.b1	GE295276
61939232	CCAH3756.g1	GE295277
61939233	CCAH3757.b1	GE295278
61939234	CCAH3757.g1	GE295279
61939235	CCAH3759.b1	GE295280
61939236	CCAH3759.g1	GE295281
61939237	CCAH3760.b1	GE295282
61939238	CCAH3761.b1	GE295283
61939239	CCAH3762.b1	GE295284
61939240	CCAH3763.b1	GE295285
61939241	CCAH3763.g1	GE295286
61939242	CCAH3764.b1	GE295287
61939243	CCAH3764.g1	GE295288
61939244	CCAH3765.b1	GE295289
61939245	CCAH3765.g1	GE295290
61939246	CCAH3766.b1	GE295291
61939247	CCAH3766.g1	GE295292
61939248	CCAH3767.b1	GE295293
61939249	CCAH3767.g1	GE295294
61939250	CCAH3768.b1	GE295295
61939251	CCAH3768.g1	GE295296
61939252	CCAH3769.b1	GE295297
61939253	CCAH3770.b1	GE295298
61939254	CCAH3770.g1	GE295299
61939255	CCAH3771.b1	GE295300
61939256	CCAH3771.g1	GE295301
61939257	CCAH3772.b1	GE295302
61939258	CCAH3772.g1	GE295303
61939259	CCAH3773.b1	GE295304
61939260	CCAH3773.g1	GE295305
61939261	CCAH3774.b1	GE295306
61939262	CCAH3774.g1	GE295307
61939263	CCAH3776.b1	GE295308
61939264	CCAH3776.g1	GE295309
61939265	CCAH3777.b1	GE295310
61939266	CCAH3778.b1	GE295311
61939267	CCAH3779.b1	GE295312
61939268	CCAH3780.b1	GE295313
61939269	CCAH3780.g1	GE295314
61939270	CCAH3782.b1	GE295315
61939271	CCAH3782.g1	GE295316
61939272	CCAH3783.b1	GE295317

61939273	CCAH3784.b1	GE295318
61939274	CCAH3784.g1	GE295319
61939275	CCAH3785.b1	GE295320
61939276	CCAH3786.b1	GE295321
61939277	CCAH3787.b1	GE295322
61939278	CCAH3787.g1	GE295323
61939279	CCAH3788.b1	GE295324
61939280	CCAH3789.g1	GE295325
61939281	CCAH3790.b1	GE295326
61939282	CCAH3790.g1	GE295327
61939283	CCAH3791.b1	GE295328
61939284	CCAH3791.g1	GE295329
61939285	CCAH3792.b1	GE295330
61939286	CCAH3793.b1	GE295331
61939287	CCAH3794.b1	GE295332
61939288	CCAH3795.b1	GE295333
61939289	CCAH3796.b1	GE295334
61939290	CCAH3797.b1	GE295335
61939291	CCAH3798.b1	GE295336
61939292	CCAH3798.g1	GE295337
61939293	CCAH3799.b1	GE295338
61939294	CCAH3800.b1	GE295339
61939295	CCAH3801.b1	GE295340
61939296	CCAH3801.g1	GE295341
61939297	CCAH3803.b1	GE295342
61939298	CCAH3803.g1	GE295343
61939299	CCAH3804.b1	GE295344
61939300	CCAH3806.b1	GE295345
61939301	CCAH3806.g1	GE295346
61939302	CCAH3807.b1	GE295347
61939303	CCAH3807.g1	GE295348
61939304	CCAH3808.g1	GE295349
61939305	CCAH3809.b1	GE295350
61939306	CCAH3809.g1	GE295351
61939307	CCAH3810.b1	GE295352
61939308	CCAH3811.b1	GE295353
61939309	CCAH3812.b1	GE295354
61939310	CCAH3812.g1	GE295355
61939311	CCAH3813.b1	GE295356
61939312	CCAH3814.g1	GE295357
61939313	CCAH3815.b1	GE295358
61939314	CCAH3816.g1	GE295359
61939315	CCAH3817.b1	GE295360
61939316	CCAH3818.b1	GE295361
61939317	CCAH3819.b1	GE295362
61939318	CCAH3820.b1	GE295363
61939319	CCAH3821.b1	GE295364
61939320	CCAH3821.g1	GE295365
61939321	CCAH3822.b1	GE295366
61939322	CCAH3822.g1	GE295367
61939323	CCAH3823.b1	GE295368
61939324	CCAH3823.g1	GE295369
61939325	CCAH3824.g1	GE295370
61939326	CCAH3825.b1	GE295371
61939327	CCAH3825.g1	GE295372
61939328	CCAH3826.b1	GE295373
61939329	CCAH3826.g1	GE295374
61939330	CCAH3827.b1	GE295375
61939331	CCAH3827.g1	GE295376
61939332	CCAH3828.b1	GE295377
61939333	CCAH3828.g1	GE295378
61939334	CCAH3829.b1	GE295379
61939335	CCAH3830.b1	GE295380

61939336	CCAH3830.g1	GE295381
61939337	CCAH3832.b1	GE295382
61939338	CCAH3833.b1	GE295383
61939339	CCAH3834.b1	GE295384
61939340	CCAH3835.b1	GE295385
61939341	CCAH3835.g1	GE295386
61939342	CCAH3836.b1	GE295387
61939343	CCAH3836.g1	GE295388
61939344	CCAH3837.b1	GE295389
61939345	CCAH3837.g1	GE295390
61939346	CCAH3838.b1	GE295391
61939347	CCAH3839.b1	GE295392
61939348	CCAH4609.b1	GE295393
61939349	CCAH4610.b1	GE295394
61939350	CCAH4610.g1	GE295395
61939351	CCAH4611.b1	GE295396
61939352	CCAH4611.g1	GE295397
61939353	CCAH4612.b1	GE295398
61939354	CCAH4612.g1	GE295399
61939355	CCAH4613.b1	GE295400
61939356	CCAH4613.g1	GE295401
61939357	CCAH4614.b1	GE295402
61939358	CCAH4615.b1	GE295403
61939359	CCAH4615.g1	GE295404
61939360	CCAH4616.b1	GE295405
61939361	CCAH4616.g1	GE295406
61939362	CCAH4617.b1	GE295407
61939363	CCAH4617.g1	GE295408
61939364	CCAH4618.b1	GE295409
61939365	CCAH4618.g1	GE295410
61939366	CCAH4619.b1	GE295411
61939367	CCAH4619.g1	GE295412
61939368	CCAH4620.g1	GE295413
61939369	CCAH4621.b1	GE295414
61939370	CCAH4621.g1	GE295415
61939371	CCAH4622.b1	GE295416
61939372	CCAH4622.g1	GE295417
61939373	CCAH4623.b1	GE295418
61939374	CCAH4623.g1	GE295419
61939375	CCAH4624.b1	GE295420
61939376	CCAH4625.b1	GE295421
61939377	CCAH4625.g1	GE295422
61939378	CCAH4626.b1	GE295423
61939379	CCAH4626.g1	GE295424
61939380	CCAH4627.b1	GE295425
61939381	CCAH4628.b1	GE295426
61939382	CCAH4628.g1	GE295427
61939383	CCAH4629.b1	GE295428
61939384	CCAH4629.g1	GE295429
61939385	CCAH4630.b1	GE295430
61939386	CCAH4631.b1	GE295431
61939387	CCAH4631.g1	GE295432
61939388	CCAH4632.b1	GE295433
61939389	CCAH4632.g1	GE295434
61939390	CCAH4633.b1	GE295435
61939391	CCAH4633.g1	GE295436
61939392	CCAH4634.b1	GE295437
61939393	CCAH4634.g1	GE295438
61939394	CCAH4635.b1	GE295439
61939395	CCAH4635.g1	GE295440
61939396	CCAH4636.b1	GE295441
61939397	CCAH4637.b1	GE295442
61939398	CCAH4637.g1	GE295443

61939399	CCAH4638.g1	GE295444
61939400	CCAH4639.b1	GE295445
61939401	CCAH4639.g1	GE295446
61939402	CCAH4641.b1	GE295447
61939403	CCAH4641.g1	GE295448
61939404	CCAH4643.b1	GE295449
61939405	CCAH4644.b1	GE295450
61939406	CCAH4644.g1	GE295451
61939407	CCAH4645.b1	GE295452
61939408	CCAH4645.g1	GE295453
61939409	CCAH4646.b1	GE295454
61939410	CCAH4646.g1	GE295455
61939411	CCAH4647.b1	GE295456
61939412	CCAH4647.g1	GE295457
61939413	CCAH4648.b1	GE295458
61939414	CCAH4648.g1	GE295459
61939415	CCAH4649.b1	GE295460
61939416	CCAH4649.g1	GE295461
61939417	CCAH4650.b1	GE295462
61939418	CCAH4650.g1	GE295463
61939419	CCAH4651.b1	GE295464
61939420	CCAH4651.g1	GE295465
61939421	CCAH4652.b1	GE295466
61939422	CCAH4653.b1	GE295467
61939423	CCAH4653.g1	GE295468
61939424	CCAH4654.b1	GE295469
61939425	CCAH4654.g1	GE295470
61939426	CCAH4655.b1	GE295471
61939427	CCAH4655.g1	GE295472
61939428	CCAH4656.b1	GE295473
61939429	CCAH4656.g1	GE295474
61939430	CCAH4657.b1	GE295475
61939431	CCAH4657.g1	GE295476
61939432	CCAH4658.b1	GE295477
61939433	CCAH4658.g1	GE295478
61939434	CCAH4659.b1	GE295479
61939435	CCAH4659.g1	GE295480
61939436	CCAH4660.b1	GE295481
61939437	CCAH4660.g1	GE295482
61939438	CCAH4661.b1	GE295483
61939439	CCAH4661.g1	GE295484
61939440	CCAH4662.b1	GE295485
61939441	CCAH4662.g1	GE295486
61939442	CCAH4664.g1	GE295487
61939443	CCAH4665.b1	GE295488
61939444	CCAH4668.b1	GE295489
61939445	CCAH4668.g1	GE295490
61939446	CCAH4669.b1	GE295491
61939447	CCAH4669.g1	GE295492
61939448	CCAH4670.b1	GE295493
61939449	CCAH4670.g1	GE295494
61939450	CCAH4671.b1	GE295495
61939451	CCAH4672.b1	GE295496
61939452	CCAH4672.g1	GE295497
61939453	CCAH4673.g1	GE295498
61939454	CCAH4674.b1	GE295499
61939455	CCAH4674.g1	GE295500
61939456	CCAH4675.b1	GE295501
61939457	CCAH4675.g1	GE295502
61939458	CCAH4676.b1	GE295503
61939459	CCAH4676.g1	GE295504
61939460	CCAH4677.b1	GE295505
61939461	CCAH4678.b1	GE295506

61939462	CCAH4678.g1	GE295507
61939463	CCAH4679.b1	GE295508
61939464	CCAH4679.g1	GE295509
61939465	CCAH4680.b1	GE295510
61939466	CCAH4681.b1	GE295511
61939467	CCAH4681.g1	GE295512
61939468	CCAH4682.b1	GE295513
61939469	CCAH4682.g1	GE295514
61939470	CCAH4684.b1	GE295515
61939471	CCAH4684.g1	GE295516
61939472	CCAH4685.b1	GE295517
61939473	CCAH4685.g1	GE295518
61939474	CCAH4686.b1	GE295519
61939475	CCAH4686.g1	GE295520
61939476	CCAH4687.b1	GE295521
61939477	CCAH4687.g1	GE295522
61939478	CCAH4688.b1	GE295523
61939479	CCAH4688.g1	GE295524
61939480	CCAH4689.b1	GE295525
61939481	CCAH4689.g1	GE295526
61939482	CCAH4690.b1	GE295527
61939483	CCAH4690.g1	GE295528
61939484	CCAH4691.b1	GE295529
61939485	CCAH4691.g1	GE295530
61939486	CCAH4692.b1	GE295531
61939487	CCAH4692.g1	GE295532
61939488	CCAH4693.g1	GE295533
61939489	CCAH4694.b1	GE295534
61939490	CCAH4694.g1	GE295535
61939491	CCAH4695.b1	GE295536
61939492	CCAH4696.b1	GE295537
61939493	CCAH4696.g1	GE295538
61939494	CCAH4697.b1	GE295539
61939495	CCAH4697.g1	GE295540
61939496	CCAH4698.b1	GE295541
61939497	CCAH4698.g1	GE295542
61939498	CCAH4699.b1	GE295543
61939499	CCAH4699.g1	GE295544
61939500	CCAH4700.b1	GE295545
61939501	CCAH4700.g1	GE295546
61939502	CCAH4701.b1	GE295547
61939503	CCAH4701.g1	GE295548
61939504	CCAH4702.b1	GE295549
61939505	CCAH4702.g1	GE295550
61939506	CCAH4703.b1	GE295551
61939507	CCAH4703.g1	GE295552
61939508	CCAH4705.b1	GE295553
61939509	CCAH4705.g1	GE295554
61939510	CCAH4706.b1	GE295555
61939511	CCAH4706.g1	GE295556
61939512	CCAH4707.b1	GE295557
61939513	CCAH4707.g1	GE295558
61939514	CCAH4708.b1	GE295559
61939515	CCAH4708.g1	GE295560
61939516	CCAH4709.b1	GE295561
61939517	CCAH4709.g1	GE295562
61939518	CCAH4710.b1	GE295563
61939519	CCAH4710.g1	GE295564
61939520	CCAH4711.b1	GE295565
61939521	CCAH4711.g1	GE295566
61939522	CCAH4713.b1	GE295567
61939523	CCAH4713.g1	GE295568
61939524	CCAH4714.b1	GE295569

61939525	CCAH4714.g1	GE295570
61939526	CCAH4715.b1	GE295571
61939527	CCAH4715.g1	GE295572
61939528	CCAH4716.b1	GE295573
61939529	CCAH4717.b1	GE295574
61939530	CCAH4717.g1	GE295575
61939531	CCAH4718.b1	GE295576
61939532	CCAH4718.g1	GE295577
61939533	CCAH4719.b1	GE295578
61939534	CCAH4719.g1	GE295579
61939535	CCAH4720.b1	GE295580
61939536	CCAH4720.g1	GE295581
61939537	CCAH4721.b1	GE295582
61939538	CCAH4721.g1	GE295583
61939539	CCAH4722.g1	GE295584
61939540	CCAH4724.b1	GE295585
61939541	CCAH4724.g1	GE295586
61939542	CCAH4725.b1	GE295587
61939543	CCAH4726.b1	GE295588
61939544	CCAH4726.g1	GE295589
61939545	CCAH4727.b1	GE295590
61939546	CCAH4727.g1	GE295591
61939547	CCAH4728.b1	GE295592
61939548	CCAH4728.g1	GE295593
61939549	CCAH4729.b1	GE295594
61939550	CCAH4729.g1	GE295595
61939551	CCAH4730.b1	GE295596
61939552	CCAH4730.g1	GE295597
61939553	CCAH4731.b1	GE295598
61939554	CCAH4731.g1	GE295599
61939555	CCAH4732.b1	GE295600
61939556	CCAH4733.b1	GE295601
61939557	CCAH4734.b1	GE295602
61939558	CCAH4734.g1	GE295603
61939559	CCAH4735.b1	GE295604
61939560	CCAH4735.g1	GE295605
61939561	CCAH4736.b1	GE295606
61939562	CCAH4736.g1	GE295607
61939563	CCAH4737.b1	GE295608
61939564	CCAH4737.g1	GE295609
61939565	CCAH4738.b1	GE295610
61939566	CCAH4738.g1	GE295611
61939567	CCAH4739.b1	GE295612
61939568	CCAH4739.g1	GE295613
61939569	CCAH4740.b1	GE295614
61939570	CCAH4740.g1	GE295615
61939571	CCAH4741.b1	GE295616
61939572	CCAH4741.g1	GE295617
61939573	CCAH4742.b1	GE295618
61939574	CCAH4742.g1	GE295619
61939575	CCAH4743.b1	GE295620
61939576	CCAH4743.g1	GE295621
61939577	CCAH4744.b1	GE295622
61939578	CCAH4744.g1	GE295623
61939579	CCAH4745.b1	GE295624
61939580	CCAH4746.b1	GE295625
61939581	CCAH4746.g1	GE295626
61939582	CCAH4747.b1	GE295627
61939583	CCAH4747.g1	GE295628
61939584	CCAH4748.b1	GE295629
61939585	CCAH4749.b1	GE295630
61939586	CCAH4749.g1	GE295631
61939587	CCAH4750.b1	GE295632

61939588	CCAH4751.b1	GE295633
61939589	CCAH4751.g1	GE295634
61939590	CCAH4752.b1	GE295635
61939591	CCAH4752.g1	GE295636
61939592	CCAH4753.b1	GE295637
61939593	CCAH4754.b1	GE295638
61939594	CCAH4754.g1	GE295639
61939595	CCAH4756.b1	GE295640
61939596	CCAH4756.g1	GE295641
61939597	CCAH4757.b1	GE295642
61939598	CCAH4757.g1	GE295643
61939599	CCAH4758.b1	GE295644
61939600	CCAH4758.g1	GE295645
61939601	CCAH4759.b1	GE295646
61939602	CCAH4759.g1	GE295647
61939603	CCAH4761.b1	GE295648
61939604	CCAH4761.g1	GE295649
61939605	CCAH4763.b1	GE295650
61939606	CCAH4763.g1	GE295651
61939607	CCAH4765.b1	GE295652
61939608	CCAH4765.g1	GE295653
61939609	CCAH4766.b1	GE295654
61939610	CCAH4767.b1	GE295655
61939611	CCAH4767.g1	GE295656
61939612	CCAH4768.b1	GE295657
61939613	CCAH4768.g1	GE295658
61939614	CCAH4769.b1	GE295659
61939615	CCAH4769.g1	GE295660
61939616	CCAH4770.b1	GE295661
61939617	CCAH4770.g1	GE295662
61939618	CCAH4772.b1	GE295663
61939619	CCAH4772.g1	GE295664
61939620	CCAH4773.b1	GE295665
61939621	CCAH4773.g1	GE295666
61939622	CCAH4774.b1	GE295667
61939623	CCAH4774.g1	GE295668
61939624	CCAH4775.g1	GE295669
61939625	CCAH4776.b1	GE295670
61939626	CCAH4776.g1	GE295671
61939627	CCAH4777.b1	GE295672
61939628	CCAH4777.g1	GE295673
61939629	CCAH4778.b1	GE295674
61939630	CCAH4778.g1	GE295675
61939631	CCAH4779.b1	GE295676
61939632	CCAH4779.g1	GE295677
61939633	CCAH4781.b1	GE295678
61939634	CCAH4781.g1	GE295679
61939635	CCAH4782.b1	GE295680
61939636	CCAH4782.g1	GE295681
61939637	CCAH4783.b1	GE295682
61939638	CCAH4783.g1	GE295683
61939639	CCAH4785.b1	GE295684
61939640	CCAH4785.g1	GE295685
61939641	CCAH4786.b1	GE295686
61939642	CCAH4786.g1	GE295687
61939643	CCAH4787.g1	GE295688
61939644	CCAH4788.b1	GE295689
61939645	CCAH4789.b1	GE295690
61939646	CCAH4790.b1	GE295691
61939647	CCAH4790.g1	GE295692
61939648	CCAH4791.b1	GE295693
61939649	CCAH4791.g1	GE295694
61939650	CCAH4792.b1	GE295695

61939651	CCAH4792.g1	GE295696
61939652	CCAH4793.b1	GE295697
61939653	CCAH4793.g1	GE295698
61939654	CCAH4794.b1	GE295699
61939655	CCAH4795.b1	GE295700
61939656	CCAH4795.g1	GE295701
61939657	CCAH4796.b1	GE295702
61939658	CCAH4796.g1	GE295703
61939659	CCAH4797.b1	GE295704
61939660	CCAH4797.g1	GE295705
61939661	CCAH4798.b1	GE295706
61939662	CCAH4798.g1	GE295707
61939663	CCAH4799.b1	GE295708
61939664	CCAH4799.g1	GE295709
61939665	CCAH4800.b1	GE295710
61939666	CCAH4801.b1	GE295711
61939667	CCAH4801.g1	GE295712
61939668	CCAH4802.b1	GE295713
61939669	CCAH4802.g1	GE295714
61939670	CCAH4804.b1	GE295715
61939671	CCAH4804.g1	GE295716
61939672	CCAH4805.b1	GE295717
61939673	CCAH4805.g1	GE295718
61939674	CCAH4806.b1	GE295719
61939675	CCAH4806.g1	GE295720
61939676	CCAH4807.b1	GE295721
61939677	CCAH4807.g1	GE295722
61939678	CCAH4808.b1	GE295723
61939679	CCAH4808.g1	GE295724
61939680	CCAH4809.b1	GE295725
61939681	CCAH4809.g1	GE295726
61939682	CCAH4810.b1	GE295727
61939683	CCAH4811.b1	GE295728
61939684	CCAH4811.g1	GE295729
61939685	CCAH4812.b1	GE295730
61939686	CCAH4813.g1	GE295731
61939687	CCAH4814.g1	GE295732
61939688	CCAH4815.b1	GE295733
61939689	CCAH4815.g1	GE295734
61939690	CCAH4816.b1	GE295735
61939691	CCAH4817.b1	GE295736
61939692	CCAH4818.b1	GE295737
61939693	CCAH4818.g1	GE295738
61939694	CCAH4819.b1	GE295739
61939695	CCAH4820.b1	GE295740
61939696	CCAH4820.g1	GE295741
61939697	CCAH4821.b1	GE295742
61939698	CCAH4821.g1	GE295743
61939699	CCAH4824.b1	GE295744
61939700	CCAH4824.g1	GE295745
61939701	CCAH4825.b1	GE295746
61939702	CCAH4825.g1	GE295747
61939703	CCAH4828.b1	GE295748
61939704	CCAH4829.b1	GE295749
61939705	CCAH4829.g1	GE295750
61939706	CCAH4830.b1	GE295751
61939707	CCAH4830.g1	GE295752
61939708	CCAH4831.b1	GE295753
61939709	CCAH4831.g1	GE295754
61939710	CCAH4832.b1	GE295755
61939711	CCAH4832.g1	GE295756
61939712	CCAH4833.b1	GE295757
61939713	CCAH4833.g1	GE295758

61939714	CCAH4834.b1	GE295759
61939715	CCAH4834.g1	GE295760
61939716	CCAH4835.b1	GE295761
61939717	CCAH4836.b1	GE295762
61939718	CCAH4836.g1	GE295763
61939719	CCAH4837.b1	GE295764
61939720	CCAH4840.b1	GE295765
61939721	CCAH4840.g1	GE295766
61939722	CCAH4842.b1	GE295767
61939723	CCAH4842.g1	GE295768
61939724	CCAH4843.b1	GE295769
61939725	CCAH4845.b1	GE295770
61939726	CCAH4845.g1	GE295771
61939727	CCAH4847.b1	GE295772
61939728	CCAH4847.g1	GE295773
61939729	CCAH4848.b1	GE295774
61939730	CCAH4848.g1	GE295775
61939731	CCAH4850.b1	GE295776
61939732	CCAH4850.g1	GE295777
61939733	CCAH4851.b1	GE295778
61939734	CCAH4851.g1	GE295779
61939735	CCAH4852.b1	GE295780
61939736	CCAH4852.g1	GE295781
61939737	CCAH4853.b1	GE295782
61939738	CCAH4853.g1	GE295783
61939739	CCAH4854.b1	GE295784
61939740	CCAH4854.g1	GE295785
61939741	CCAH4855.b1	GE295786
61939742	CCAH4855.g1	GE295787
61939743	CCAH4856.b1	GE295788
61939744	CCAH4856.g1	GE295789
61939745	CCAH4857.b1	GE295790
61939746	CCAH4857.g1	GE295791
61939747	CCAH4859.b1	GE295792
61939748	CCAH4859.g1	GE295793
61939749	CCAH4860.b1	GE295794
61939750	CCAH4860.g1	GE295795
61939751	CCAH4862.b1	GE295796
61939752	CCAH4862.g1	GE295797
61939753	CCAH4863.b1	GE295798
61939754	CCAH4863.g1	GE295799
61939755	CCAH4864.b1	GE295800
61939756	CCAH4864.g1	GE295801
61939757	CCAH4865.b1	GE295802
61939758	CCAH4865.g1	GE295803
61939759	CCAH4866.b1	GE295804
61939760	CCAH4866.g1	GE295805
61939761	CCAH4867.b1	GE295806
61939762	CCAH4867.g1	GE295807
61939763	CCAH4868.b1	GE295808
61939764	CCAH4868.g1	GE295809
61939765	CCAH4869.b1	GE295810
61939766	CCAH4870.b1	GE295811
61939767	CCAH4870.g1	GE295812
61939768	CCAH4871.b1	GE295813
61939769	CCAH4871.g1	GE295814
61939770	CCAH4872.g1	GE295815
61939771	CCAH4873.b1	GE295816
61939772	CCAH4874.b1	GE295817
61939773	CCAH4874.g1	GE295818
61939774	CCAH4875.b1	GE295819
61939775	CCAH4875.g1	GE295820
61939776	CCAH4876.b1	GE295821

61939777	CCAH4876.g1	GE295822
61939778	CCAH4877.b1	GE295823
61939779	CCAH4877.g1	GE295824
61939780	CCAH4878.b1	GE295825
61939781	CCAH4878.g1	GE295826
61939782	CCAH4879.b1	GE295827
61939783	CCAH4879.g1	GE295828
61939784	CCAH4880.b1	GE295829
61939785	CCAH4880.g1	GE295830
61939786	CCAH4882.b1	GE295831
61939787	CCAH4882.g1	GE295832
61939788	CCAH4883.b1	GE295833
61939789	CCAH4883.g1	GE295834
61939790	CCAH4884.b1	GE295835
61939791	CCAH4884.g1	GE295836
61939792	CCAH4885.b1	GE295837
61939793	CCAH4886.b1	GE295838
61939794	CCAH4886.g1	GE295839
61939795	CCAH4887.b1	GE295840
61939796	CCAH4887.g1	GE295841
61939797	CCAH4888.b1	GE295842
61939798	CCAH4888.g1	GE295843
61939799	CCAH4890.b1	GE295844
61939800	CCAH4890.g1	GE295845
61939801	CCAH4891.b1	GE295846
61939802	CCAH4891.g1	GE295847
61939803	CCAH4892.b1	GE295848
61939804	CCAH4892.g1	GE295849
61939805	CCAH4893.b1	GE295850
61939806	CCAH4893.g1	GE295851
61939807	CCAH4894.b1	GE295852
61939808	CCAH4894.g1	GE295853
61939809	CCAH4895.b1	GE295854
61939810	CCAH4895.g1	GE295855
61939811	CCAH4896.b1	GE295856
61939812	CCAH4896.g1	GE295857
61939813	CCAH4897.b1	GE295858
61939814	CCAH4897.g1	GE295859
61939815	CCAH4898.b1	GE295860
61939816	CCAH4898.g1	GE295861
61939817	CCAH4899.g1	GE295862
61939818	CCAH4900.b1	GE295863
61939819	CCAH4900.g1	GE295864
61939820	CCAH4901.b1	GE295865
61939821	CCAH4902.b1	GE295866
61939822	CCAH4902.g1	GE295867
61939823	CCAH4904.b1	GE295868
61939824	CCAH4904.g1	GE295869
61939825	CCAH4905.b1	GE295870
61939826	CCAH4905.g1	GE295871
61939827	CCAH4906.b1	GE295872
61939828	CCAH4906.g1	GE295873
61939829	CCAH4907.b1	GE295874
61939830	CCAH4907.g1	GE295875
61939831	CCAH4910.b1	GE295876
61939832	CCAH4910.g1	GE295877
61939833	CCAH4912.b1	GE295878
61939834	CCAH4912.g1	GE295879
61939835	CCAH4914.b1	GE295880
61939836	CCAH4914.g1	GE295881
61939837	CCAH4915.b1	GE295882
61939838	CCAH4915.g1	GE295883
61939839	CCAH4916.b1	GE295884

61939840	CCAH4916.g1	GE295885
61939841	CCAH4917.b1	GE295886
61939842	CCAH4917.g1	GE295887
61939843	CCAH4918.b1	GE295888
61939844	CCAH4918.g1	GE295889
61939845	CCAH4919.b1	GE295890
61939846	CCAH4919.g1	GE295891
61939847	CCAH4920.b1	GE295892
61939848	CCAH4920.g1	GE295893
61939849	CCAH4921.b1	GE295894
61939850	CCAH4921.g1	GE295895
61939851	CCAH4922.b1	GE295896
61939852	CCAH4923.b1	GE295897
61939853	CCAH4923.g1	GE295898
61939854	CCAH4924.b1	GE295899
61939855	CCAH4924.g1	GE295900
61939856	CCAH4925.b1	GE295901
61939857	CCAH4925.g1	GE295902
61939858	CCAH4926.b1	GE295903
61939859	CCAH4926.g1	GE295904
61939860	CCAH4927.b1	GE295905
61939861	CCAH4927.g1	GE295906
61939862	CCAH4928.b1	GE295907
61939863	CCAH4928.g1	GE295908
61939864	CCAH4929.b1	GE295909
61939865	CCAH4929.g1	GE295910
61939866	CCAH4931.b1	GE295911
61939867	CCAH4932.b1	GE295912
61939868	CCAH4932.g1	GE295913
61939869	CCAH4933.b1	GE295914
61939870	CCAH4933.g1	GE295915
61939871	CCAH4934.b1	GE295916
61939872	CCAH4934.g1	GE295917
61939873	CCAH4935.b1	GE295918
61939874	CCAH4935.g1	GE295919
61939875	CCAH4936.b1	GE295920
61939876	CCAH4936.g1	GE295921
61939877	CCAH4937.b1	GE295922
61939878	CCAH4938.b1	GE295923
61939879	CCAH4938.g1	GE295924
61939880	CCAH4939.b1	GE295925
61939881	CCAH4939.g1	GE295926
61939882	CCAH4940.b1	GE295927
61939883	CCAH4940.g1	GE295928
61939884	CCAH4941.g1	GE295929
61939885	CCAH4942.g1	GE295930
61939886	CCAH4943.b1	GE295931
61939887	CCAH4943.g1	GE295932
61939888	CCAH4944.b1	GE295933
61939889	CCAH4944.g1	GE295934
61939890	CCAH4945.b1	GE295935
61939891	CCAH4945.g1	GE295936
61939892	CCAH4947.b1	GE295937
61939893	CCAH4947.g1	GE295938
61939894	CCAH4948.b1	GE295939
61939895	CCAH4948.g1	GE295940
61939896	CCAH4949.b1	GE295941
61939897	CCAH4949.g1	GE295942
61939898	CCAH4950.b1	GE295943
61939899	CCAH4950.g1	GE295944
61939900	CCAH4951.b1	GE295945
61939901	CCAH4951.g1	GE295946
61939902	CCAH4952.b1	GE295947

61939903	CCAH4953.b1	GE295948
61939904	CCAH4954.b1	GE295949
61939905	CCAH4954.g1	GE295950
61939906	CCAH4956.b1	GE295951
61939907	CCAH4956.g1	GE295952
61939908	CCAH4957.b1	GE295953
61939909	CCAH4957.g1	GE295954
61939910	CCAH4958.b1	GE295955
61939911	CCAH4958.g1	GE295956
61939912	CCAH4959.b1	GE295957
61939913	CCAH4959.g1	GE295958
61939914	CCAH4960.b1	GE295959
61939915	CCAH4960.g1	GE295960
61939916	CCAH4961.b1	GE295961
61939917	CCAH4961.g1	GE295962
61939918	CCAH4962.b1	GE295963
61939919	CCAH4962.g1	GE295964
61939920	CCAH4963.b1	GE295965
61939921	CCAH4963.g1	GE295966
61939922	CCAH4965.b1	GE295967
61939923	CCAH4965.g1	GE295968
61939924	CCAH4966.b1	GE295969
61939925	CCAH4966.g1	GE295970
61939926	CCAH4967.b1	GE295971
61939927	CCAH4967.g1	GE295972
61939928	CCAH4968.b1	GE295973
61939929	CCAH4968.g1	GE295974
61939930	CCAH4969.b1	GE295975
61939931	CCAH4969.g1	GE295976
61939932	CCAH4970.b1	GE295977
61939933	CCAH4970.g1	GE295978
61939934	CCAH4971.b1	GE295979
61939935	CCAH4971.g1	GE295980
61939936	CCAH4972.b1	GE295981
61939937	CCAH4972.g1	GE295982
61939938	CCAH4973.b1	GE295983
61939939	CCAH4974.b1	GE295984
61939940	CCAH4974.g1	GE295985
61939941	CCAH4976.b1	GE295986
61939942	CCAH4976.g1	GE295987
61939943	CCAH4977.b1	GE295988
61939944	CCAH4977.g1	GE295989
61939945	CCAH4979.g1	GE295990
61939946	CCAH4980.b1	GE295991
61939947	CCAH4980.g1	GE295992
61939948	CCAH4982.b1	GE295993
61939949	CCAH4983.b1	GE295994
61939950	CCAH4983.g1	GE295995
61939951	CCAH4984.b1	GE295996
61939952	CCAH4984.g1	GE295997
61939953	CCAH4985.b1	GE295998
61939954	CCAH4985.g1	GE295999
61939955	CCAH4986.b1	GE296000
61939956	CCAH4986.g1	GE296001
61939957	CCAH4987.b1	GE296002
61939958	CCAH4987.g1	GE296003
61939959	CCAH4988.g1	GE296004
61939960	CCAH4989.b1	GE296005
61939961	CCAH4989.g1	GE296006
61939962	CCAH4990.b1	GE296007
61939963	CCAH4990.g1	GE296008
61939964	CCAH4991.b1	GE296009
61939965	CCAH4991.g1	GE296010

61939966	CCAH500.g6	GE296011
61939967	CCAH5377.b1	GE296012
61939968	CCAH5377.g1	GE296013
61939969	CCAH5378.g1	GE296014
61939970	CCAH5379.b1	GE296015
61939971	CCAH5379.g1	GE296016
61939972	CCAH5380.b1	GE296017
61939973	CCAH5380.g1	GE296018
61939974	CCAH5381.b1	GE296019
61939975	CCAH5381.g1	GE296020
61939976	CCAH5382.b1	GE296021
61939977	CCAH5382.g1	GE296022
61939978	CCAH5383.b1	GE296023
61939979	CCAH5383.g1	GE296024
61939980	CCAH5384.b1	GE296025
61939981	CCAH5384.g1	GE296026
61939982	CCAH5386.b1	GE296027
61939983	CCAH5387.b1	GE296028
61939984	CCAH5387.g1	GE296029
61939985	CCAH5388.b1	GE296030
61939986	CCAH5388.g1	GE296031
61939987	CCAH5389.b1	GE296032
61939988	CCAH5389.g1	GE296033
61939989	CCAH5390.g1	GE296034
61939990	CCAH5391.b1	GE296035
61939991	CCAH5391.g1	GE296036
61939992	CCAH5392.b1	GE296037
61939993	CCAH5392.g1	GE296038
61939994	CCAH5393.b1	GE296039
61939995	CCAH5393.g1	GE296040
61939996	CCAH5394.g1	GE296041
61939997	CCAH5395.b1	GE296042
61939998	CCAH5395.g1	GE296043
61939999	CCAH5396.b1	GE296044
61940000	CCAH5398.b1	GE296045
61940001	CCAH5398.g1	GE296046
61940002	CCAH5399.b1	GE296047
61940003	CCAH5399.g1	GE296048
61940004	CCAH5400.b1	GE296049
61940005	CCAH5400.g1	GE296050
61940006	CCAH5401.b1	GE296051
61940007	CCAH5401.g1	GE296052
61940008	CCAH5402.b1	GE296053
61940009	CCAH5402.g1	GE296054
61940010	CCAH5403.g1	GE296055
61940011	CCAH5404.g1	GE296056
61940012	CCAH5405.g1	GE296057
61940013	CCAH5406.b1	GE296058
61940014	CCAH5406.g1	GE296059
61940015	CCAH5407.b1	GE296060
61940016	CCAH5410.b1	GE296061
61940017	CCAH5411.b1	GE296062
61940018	CCAH5411.g1	GE296063
61940019	CCAH5412.g1	GE296064
61940020	CCAH5413.b1	GE296065
61940021	CCAH5413.g1	GE296066
61940022	CCAH5415.b1	GE296067
61940023	CCAH5415.g1	GE296068
61940024	CCAH5416.g1	GE296069
61940025	CCAH5417.g1	GE296070
61940026	CCAH5418.g1	GE296071
61940027	CCAH5419.b1	GE296072
61940028	CCAH5419.g1	GE296073

61940029	CCAH5420.b1	GE296074
61940030	CCAH5420.g1	GE296075
61940031	CCAH5421.g1	GE296076
61940032	CCAH5422.g1	GE296077
61940033	CCAH5423.g1	GE296078
61940034	CCAH5426.b1	GE296079
61940035	CCAH5426.g1	GE296080
61940036	CCAH5427.b1	GE296081
61940037	CCAH5427.g1	GE296082
61940038	CCAH5428.b1	GE296083
61940039	CCAH5428.g1	GE296084
61940040	CCAH5429.b1	GE296085
61940041	CCAH5429.g1	GE296086
61940042	CCAH5430.b1	GE296087
61940043	CCAH5430.g1	GE296088
61940044	CCAH5431.g1	GE296089
61940045	CCAH5432.b1	GE296090
61940046	CCAH5432.g1	GE296091
61940047	CCAH5434.g1	GE296092
61940048	CCAH5435.b1	GE296093
61940049	CCAH5435.g1	GE296094
61940050	CCAH5436.b1	GE296095
61940051	CCAH5436.g1	GE296096
61940052	CCAH5437.b1	GE296097
61940053	CCAH5437.g1	GE296098
61940054	CCAH5438.b1	GE296099
61940055	CCAH5439.b1	GE296100
61940056	CCAH5439.g1	GE296101
61940057	CCAH5440.b1	GE296102
61940058	CCAH5440.g1	GE296103
61940059	CCAH5442.g1	GE296104
61940060	CCAH5443.b1	GE296105
61940061	CCAH5443.g1	GE296106
61940062	CCAH5444.g1	GE296107
61940063	CCAH5445.g1	GE296108
61940064	CCAH5446.b1	GE296109
61940065	CCAH5446.g1	GE296110
61940066	CCAH5448.b1	GE296111
61940067	CCAH5448.g1	GE296112
61940068	CCAH5449.b1	GE296113
61940069	CCAH5449.g1	GE296114
61940070	CCAH5450.b1	GE296115
61940071	CCAH5450.g1	GE296116
61940072	CCAH5451.g1	GE296117
61940073	CCAH5453.b1	GE296118
61940074	CCAH5453.g1	GE296119
61940075	CCAH5455.b1	GE296120
61940076	CCAH5455.g1	GE296121
61940077	CCAH5456.b1	GE296122
61940078	CCAH5457.b1	GE296123
61940079	CCAH5457.g1	GE296124
61940080	CCAH5458.b1	GE296125
61940081	CCAH5458.g1	GE296126
61940082	CCAH5459.b1	GE296127
61940083	CCAH5460.b1	GE296128
61940084	CCAH5460.g1	GE296129
61940085	CCAH5461.b1	GE296130
61940086	CCAH5462.b1	GE296131
61940087	CCAH5462.g1	GE296132
61940088	CCAH5463.b1	GE296133
61940089	CCAH5463.g1	GE296134
61940090	CCAH5464.b1	GE296135
61940091	CCAH5464.g1	GE296136

61940092	CCAH5465.g1	GE296137
61940093	CCAH5466.b1	GE296138
61940094	CCAH5468.b1	GE296139
61940095	CCAH5468.g1	GE296140
61940096	CCAH5469.b1	GE296141
61940097	CCAH5469.g1	GE296142
61940098	CCAH5470.b1	GE296143
61940099	CCAH5470.g1	GE296144
61940100	CCAH5471.b1	GE296145
61940101	CCAH5471.g1	GE296146
61940102	CCAH5472.b1	GE296147
61940103	CCAH5473.b1	GE296148
61940104	CCAH5473.g1	GE296149
61940105	CCAH5474.b1	GE296150
61940106	CCAH5474.g1	GE296151
61940107	CCAH5475.b1	GE296152
61940108	CCAH5475.g1	GE296153
61940109	CCAH5476.b1	GE296154
61940110	CCAH5476.g1	GE296155
61940111	CCAH5477.b1	GE296156
61940112	CCAH5477.g1	GE296157
61940113	CCAH5478.b1	GE296158
61940114	CCAH5478.g1	GE296159
61940115	CCAH5479.b1	GE296160
61940116	CCAH5479.g1	GE296161
61940117	CCAH5480.b1	GE296162
61940118	CCAH5480.g1	GE296163
61940119	CCAH5481.b1	GE296164
61940120	CCAH5482.b1	GE296165
61940121	CCAH5482.g1	GE296166
61940122	CCAH5483.b1	GE296167
61940123	CCAH5483.g1	GE296168
61940124	CCAH5484.b1	GE296169
61940125	CCAH5484.g1	GE296170
61940126	CCAH5485.b1	GE296171
61940127	CCAH5485.g1	GE296172
61940128	CCAH5486.g1	GE296173
61940129	CCAH5488.b1	GE296174
61940130	CCAH5488.g1	GE296175
61940131	CCAH5489.g1	GE296176
61940132	CCAH5490.g1	GE296177
61940133	CCAH5491.b1	GE296178
61940134	CCAH5491.g1	GE296179
61940135	CCAH5492.b1	GE296180
61940136	CCAH5492.g1	GE296181
61940137	CCAH5493.b1	GE296182
61940138	CCAH5493.g1	GE296183
61940139	CCAH5494.g1	GE296184
61940140	CCAH5495.b1	GE296185
61940141	CCAH5495.g1	GE296186
61940142	CCAH5496.g1	GE296187
61940143	CCAH5497.b1	GE296188
61940144	CCAH5498.b1	GE296189
61940145	CCAH5498.g1	GE296190
61940146	CCAH5499.b1	GE296191
61940147	CCAH5499.g1	GE296192
61940148	CCAH5500.b1	GE296193
61940149	CCAH5500.g1	GE296194
61940150	CCAH5501.g1	GE296195
61940151	CCAH5502.b1	GE296196
61940152	CCAH5503.b1	GE296197
61940153	CCAH5503.g1	GE296198
61940154	CCAH5504.b1	GE296199

61940155	CCAH5504.g1	GE296200
61940156	CCAH5505.g1	GE296201
61940157	CCAH5506.b1	GE296202
61940158	CCAH5506.g1	GE296203
61940159	CCAH5507.b1	GE296204
61940160	CCAH5507.g1	GE296205
61940161	CCAH5508.g1	GE296206
61940162	CCAH5509.b1	GE296207
61940163	CCAH5509.g1	GE296208
61940164	CCAH5510.b1	GE296209
61940165	CCAH5510.g1	GE296210
61940166	CCAH5512.g1	GE296211
61940167	CCAH5513.g1	GE296212
61940168	CCAH5514.g1	GE296213
61940169	CCAH5515.b1	GE296214
61940170	CCAH5515.g1	GE296215
61940171	CCAH5516.b1	GE296216
61940172	CCAH5516.g1	GE296217
61940173	CCAH5517.g1	GE296218
61940174	CCAH5518.g1	GE296219
61940175	CCAH5519.b1	GE296220
61940176	CCAH5521.g1	GE296221
61940177	CCAH5522.b1	GE296222
61940178	CCAH5522.g1	GE296223
61940179	CCAH5523.b1	GE296224
61940180	CCAH5523.g1	GE296225
61940181	CCAH5524.b1	GE296226
61940182	CCAH5524.g1	GE296227
61940183	CCAH5525.b1	GE296228
61940184	CCAH5525.g1	GE296229
61940185	CCAH5526.b1	GE296230
61940186	CCAH5526.g1	GE296231
61940187	CCAH5527.g1	GE296232
61940188	CCAH5528.b1	GE296233
61940189	CCAH5528.g1	GE296234
61940190	CCAH5529.b1	GE296235
61940191	CCAH5529.g1	GE296236
61940192	CCAH5530.g1	GE296237
61940193	CCAH5531.b1	GE296238
61940194	CCAH5532.b1	GE296239
61940195	CCAH5532.g1	GE296240
61940196	CCAH5533.b1	GE296241
61940197	CCAH5533.g1	GE296242
61940198	CCAH5534.b1	GE296243
61940199	CCAH5534.g1	GE296244
61940200	CCAH5535.b1	GE296245
61940201	CCAH5535.g1	GE296246
61940202	CCAH5536.b1	GE296247
61940203	CCAH5537.b1	GE296248
61940204	CCAH5538.g1	GE296249
61940205	CCAH5539.b1	GE296250
61940206	CCAH5539.g1	GE296251
61940207	CCAH5540.g1	GE296252
61940208	CCAH5541.g1	GE296253
61940209	CCAH5543.g1	GE296254
61940210	CCAH5546.b1	GE296255
61940211	CCAH5547.g1	GE296256
61940212	CCAH5548.g1	GE296257
61940213	CCAH5549.b1	GE296258
61940214	CCAH5549.g1	GE296259
61940215	CCAH5551.b1	GE296260
61940216	CCAH5551.g1	GE296261
61940217	CCAH5552.b1	GE296262

61940218	CCAH5553.b1	GE296263
61940219	CCAH5553.g1	GE296264
61940220	CCAH5555.b1	GE296265
61940221	CCAH5558.b1	GE296266
61940222	CCAH5558.g1	GE296267
61940223	CCAH5559.b1	GE296268
61940224	CCAH5559.g1	GE296269
61940225	CCAH5560.b1	GE296270
61940226	CCAH5560.g1	GE296271
61940227	CCAH5561.g1	GE296272
61940228	CCAH5562.b1	GE296273
61940229	CCAH5562.g1	GE296274
61940230	CCAH5563.b1	GE296275
61940231	CCAH5563.g1	GE296276
61940232	CCAH5565.b1	GE296277
61940233	CCAH5565.g1	GE296278
61940234	CCAH5566.b1	GE296279
61940235	CCAH5567.b1	GE296280
61940236	CCAH5567.g1	GE296281
61940237	CCAH5568.b1	GE296282
61940238	CCAH5569.b1	GE296283
61940239	CCAH5570.b1	GE296284
61940240	CCAH5570.g1	GE296285
61940241	CCAH5571.g1	GE296286
61940242	CCAH5573.b1	GE296287
61940243	CCAH5573.g1	GE296288
61940244	CCAH5574.b1	GE296289
61940245	CCAH5574.g1	GE296290
61940246	CCAH5575.b1	GE296291
61940247	CCAH5575.g1	GE296292
61940248	CCAH5576.g1	GE296293
61940249	CCAH5577.b1	GE296294
61940250	CCAH5577.g1	GE296295
61940251	CCAH5578.b1	GE296296
61940252	CCAH5578.g1	GE296297
61940253	CCAH5579.b1	GE296298
61940254	CCAH5579.g1	GE296299
61940255	CCAH5580.b1	GE296300
61940256	CCAH5580.g1	GE296301
61940257	CCAH5581.b1	GE296302
61940258	CCAH5581.g1	GE296303
61940259	CCAH5582.g1	GE296304
61940260	CCAH5583.b1	GE296305
61940261	CCAH5583.g1	GE296306
61940262	CCAH5586.g1	GE296307
61940263	CCAH5587.g1	GE296308
61940264	CCAH5588.b1	GE296309
61940265	CCAH5588.g1	GE296310
61940266	CCAH5589.b1	GE296311
61940267	CCAH5590.b1	GE296312
61940268	CCAH5590.g1	GE296313
61940269	CCAH5591.g1	GE296314
61940270	CCAH5592.b1	GE296315
61940271	CCAH5592.g1	GE296316
61940272	CCAH5593.b1	GE296317
61940273	CCAH5593.g1	GE296318
61940274	CCAH5595.b1	GE296319
61940275	CCAH5595.g1	GE296320
61940276	CCAH5596.b1	GE296321
61940277	CCAH5596.g1	GE296322
61940278	CCAH5597.g1	GE296323
61940279	CCAH5598.b1	GE296324
61940280	CCAH5598.g1	GE296325

61940281	CCAH5599.b1	GE296326
61940282	CCAH5599.g1	GE296327
61940283	CCAH5600.b1	GE296328
61940284	CCAH5600.g1	GE296329
61940285	CCAH5601.g1	GE296330
61940286	CCAH5602.b1	GE296331
61940287	CCAH5603.b1	GE296332
61940288	CCAH5603.g1	GE296333
61940289	CCAH5604.g1	GE296334
61940290	CCAH5605.b1	GE296335
61940291	CCAH5605.g1	GE296336
61940292	CCAH5606.b1	GE296337
61940293	CCAH5607.g1	GE296338
61940294	CCAH5608.g1	GE296339
61940295	CCAH5609.g1	GE296340
61940296	CCAH5610.g1	GE296341
61940297	CCAH5611.g1	GE296342
61940298	CCAH5612.b1	GE296343
61940299	CCAH5613.g1	GE296344
61940300	CCAH5616.g1	GE296345
61940301	CCAH5617.b1	GE296346
61940302	CCAH5617.g1	GE296347
61940303	CCAH5618.b1	GE296348
61940304	CCAH5618.g1	GE296349
61940305	CCAH5619.b1	GE296350
61940306	CCAH5619.g1	GE296351
61940307	CCAH5620.b1	GE296352
61940308	CCAH5620.g1	GE296353
61940309	CCAH5621.b1	GE296354
61940310	CCAH5621.g1	GE296355
61940311	CCAH5622.b1	GE296356
61940312	CCAH5622.g1	GE296357
61940313	CCAH5623.g1	GE296358
61940314	CCAH5624.b1	GE296359
61940315	CCAH5624.g1	GE296360
61940316	CCAH5625.b1	GE296361
61940317	CCAH5625.g1	GE296362
61940318	CCAH5626.g1	GE296363
61940319	CCAH5627.b1	GE296364
61940320	CCAH5627.g1	GE296365
61940321	CCAH5628.b1	GE296366
61940322	CCAH5628.g1	GE296367
61940323	CCAH5629.b1	GE296368
61940324	CCAH5629.g1	GE296369
61940325	CCAH5630.b1	GE296370
61940326	CCAH5630.g1	GE296371
61940327	CCAH5631.b1	GE296372
61940328	CCAH5631.g1	GE296373
61940329	CCAH5633.b1	GE296374
61940330	CCAH5633.g1	GE296375
61940331	CCAH5634.g1	GE296376
61940332	CCAH5635.b1	GE296377
61940333	CCAH5635.g1	GE296378
61940334	CCAH5636.g1	GE296379
61940335	CCAH5637.g1	GE296380
61940336	CCAH5638.b1	GE296381
61940337	CCAH5639.g1	GE296382
61940338	CCAH5640.b1	GE296383
61940339	CCAH5640.g1	GE296384
61940340	CCAH5641.b1	GE296385
61940341	CCAH5641.g1	GE296386
61940342	CCAH5642.b1	GE296387
61940343	CCAH5642.g1	GE296388

61940344	CCAH5644.g1	GE296389
61940345	CCAH5645.b1	GE296390
61940346	CCAH5645.g1	GE296391
61940347	CCAH5647.b1	GE296392
61940348	CCAH5647.g1	GE296393
61940349	CCAH5648.b1	GE296394
61940350	CCAH5648.g1	GE296395
61940351	CCAH5650.g1	GE296396
61940352	CCAH5651.b1	GE296397
61940353	CCAH5651.g1	GE296398
61940354	CCAH5652.b1	GE296399
61940355	CCAH5653.b1	GE296400
61940356	CCAH5653.g1	GE296401
61940357	CCAH5655.b1	GE296402
61940358	CCAH5655.g1	GE296403
61940359	CCAH5656.b1	GE296404
61940360	CCAH5656.g1	GE296405
61940361	CCAH5657.b1	GE296406
61940362	CCAH5657.g1	GE296407
61940363	CCAH5658.b1	GE296408
61940364	CCAH5659.g1	GE296409
61940365	CCAH5660.g1	GE296410
61940366	CCAH5661.b1	GE296411
61940367	CCAH5661.g1	GE296412
61940368	CCAH5662.b1	GE296413
61940369	CCAH5662.g1	GE296414
61940370	CCAH5663.b1	GE296415
61940371	CCAH5663.g1	GE296416
61940372	CCAH5665.b1	GE296417
61940373	CCAH5665.g1	GE296418
61940374	CCAH5666.g1	GE296419
61940375	CCAH5667.b1	GE296420
61940376	CCAH5667.g1	GE296421
61940377	CCAH5668.b1	GE296422
61940378	CCAH5668.g1	GE296423
61940379	CCAH5669.b1	GE296424
61940380	CCAH5669.g1	GE296425
61940381	CCAH5670.b1	GE296426
61940382	CCAH5670.g1	GE296427
61940383	CCAH5673.b1	GE296428
61940384	CCAH5673.g1	GE296429
61940385	CCAH5674.b1	GE296430
61940386	CCAH5675.b1	GE296431
61940387	CCAH5675.g1	GE296432
61940388	CCAH5676.b1	GE296433
61940389	CCAH5676.g1	GE296434
61940390	CCAH5677.g1	GE296435
61940391	CCAH5679.b1	GE296436
61940392	CCAH5679.g1	GE296437
61940393	CCAH5680.b1	GE296438
61940394	CCAH5680.g1	GE296439
61940395	CCAH5681.b1	GE296440
61940396	CCAH5681.g1	GE296441
61940397	CCAH5682.g1	GE296442
61940398	CCAH5683.g1	GE296443
61940399	CCAH5684.b1	GE296444
61940400	CCAH5685.b1	GE296445
61940401	CCAH5685.g1	GE296446
61940402	CCAH5686.b1	GE296447
61940403	CCAH5687.b1	GE296448
61940404	CCAH5687.g1	GE296449
61940405	CCAH5688.b1	GE296450
61940406	CCAH5688.g1	GE296451

61940407	CCAH5689.b1	GE296452
61940408	CCAH5689.g1	GE296453
61940409	CCAH5694.b1	GE296454
61940410	CCAH5694.g1	GE296455
61940411	CCAH5695.b1	GE296456
61940412	CCAH5695.g1	GE296457
61940413	CCAH5696.b1	GE296458
61940414	CCAH5696.g1	GE296459
61940415	CCAH5698.b1	GE296460
61940416	CCAH5698.g1	GE296461
61940417	CCAH5699.b1	GE296462
61940418	CCAH5699.g1	GE296463
61940419	CCAH5700.g1	GE296464
61940420	CCAH5701.b1	GE296465
61940421	CCAH5701.g1	GE296466
61940422	CCAH5702.b1	GE296467
61940423	CCAH5703.g1	GE296468
61940424	CCAH5704.g1	GE296469
61940425	CCAH5705.g1	GE296470
61940426	CCAH5707.b1	GE296471
61940427	CCAH5707.g1	GE296472
61940428	CCAH5708.b1	GE296473
61940429	CCAH5708.g1	GE296474
61940430	CCAH5709.g1	GE296475
61940431	CCAH5710.g1	GE296476
61940432	CCAH5713.b1	GE296477
61940433	CCAH5713.g1	GE296478
61940434	CCAH5714.b1	GE296479
61940435	CCAH5715.b1	GE296480
61940436	CCAH5715.g1	GE296481
61940437	CCAH5716.b1	GE296482
61940438	CCAH5716.g1	GE296483
61940439	CCAH5717.b1	GE296484
61940440	CCAH5717.g1	GE296485
61940441	CCAH5718.b1	GE296486
61940442	CCAH5718.g1	GE296487
61940443	CCAH5719.g1	GE296488
61940444	CCAH5720.b1	GE296489
61940445	CCAH5720.g1	GE296490
61940446	CCAH5721.g1	GE296491
61940447	CCAH5722.g1	GE296492
61940448	CCAH5723.b1	GE296493
61940449	CCAH5723.g1	GE296494
61940450	CCAH5725.b1	GE296495
61940451	CCAH5725.g1	GE296496
61940452	CCAH5726.b1	GE296497
61940453	CCAH5726.g1	GE296498
61940454	CCAH5727.b1	GE296499
61940455	CCAH5727.g1	GE296500
61940456	CCAH5728.b1	GE296501
61940457	CCAH5728.g1	GE296502
61940458	CCAH5729.b1	GE296503
61940459	CCAH5729.g1	GE296504
61940460	CCAH5730.g1	GE296505
61940461	CCAH5731.b1	GE296506
61940462	CCAH5731.g1	GE296507
61940463	CCAH5732.b1	GE296508
61940464	CCAH5732.g1	GE296509
61940465	CCAH5733.g1	GE296510
61940466	CCAH5734.b1	GE296511
61940467	CCAH5734.g1	GE296512
61940468	CCAH5735.g1	GE296513
61940469	CCAH5736.b1	GE296514

61940470	CCAH5736.g1	GE296515
61940471	CCAH5737.b1	GE296516
61940472	CCAH5737.g1	GE296517
61940473	CCAH5738.b1	GE296518
61940474	CCAH5738.g1	GE296519
61940475	CCAH5739.g1	GE296520
61940476	CCAH5740.g1	GE296521
61940477	CCAH5743.b1	GE296522
61940478	CCAH5745.b1	GE296523
61940479	CCAH5745.g1	GE296524
61940480	CCAH5747.b1	GE296525
61940481	CCAH5747.g1	GE296526
61940482	CCAH5748.b1	GE296527
61940483	CCAH5748.g1	GE296528
61940484	CCAH5749.b1	GE296529
61940485	CCAH5749.g1	GE296530
61940486	CCAH5750.b1	GE296531
61940487	CCAH5750.g1	GE296532
61940488	CCAH5751.b1	GE296533
61940489	CCAH5752.g1	GE296534
61940490	CCAH5754.b1	GE296535
61940491	CCAH5754.g1	GE296536
61940492	CCAH5755.b1	GE296537
61940493	CCAH5757.b1	GE296538
61940494	CCAH5757.g1	GE296539
61940495	CCAH5761.b1	GE296540
61940496	CCAH5761.g1	GE296541
61940497	CCAH5762.b1	GE296542
61940498	CCAH5762.g1	GE296543
61940499	CCAH5763.b1	GE296544
61940500	CCAH5763.g1	GE296545
61940501	CCAH5764.b1	GE296546
61940502	CCAH5764.g1	GE296547
61940503	CCAH5765.b1	GE296548
61940504	CCAH5765.g1	GE296549
61940505	CCAH5766.b1	GE296550
61940506	CCAH5767.b1	GE296551
61940507	CCAH5767.g1	GE296552
61940508	CCAH5769.b1	GE296553
61940509	CCAH5769.g1	GE296554
61940510	CCAH5770.b1	GE296555
61940511	CCAH5770.g1	GE296556
61940512	CCAH5771.b1	GE296557
61940513	CCAH5771.g1	GE296558
61940514	CCAH5772.g1	GE296559
61940515	CCAH5773.b1	GE296560
61940516	CCAH5773.g1	GE296561
61940517	CCAH5774.b1	GE296562
61940518	CCAH5774.g1	GE296563
61940519	CCAH5775.b1	GE296564
61940520	CCAH5775.g1	GE296565
61940521	CCAH5776.b1	GE296566
61940522	CCAH5776.g1	GE296567
61940523	CCAH5777.b1	GE296568
61940524	CCAH5777.g1	GE296569
61940525	CCAH5780.b1	GE296570
61940526	CCAH5780.g1	GE296571
61940527	CCAH5781.b1	GE296572
61940528	CCAH5781.g1	GE296573
61940529	CCAH5782.b1	GE296574
61940530	CCAH5782.g1	GE296575
61940531	CCAH5783.b1	GE296576
61940532	CCAH5783.g1	GE296577

61940533	CCAH5784.g1	GE296578
61940534	CCAH5785.b1	GE296579
61940535	CCAH5786.b1	GE296580
61940536	CCAH5787.b1	GE296581
61940537	CCAH5788.b1	GE296582
61940538	CCAH5788.g1	GE296583
61940539	CCAH5790.b1	GE296584
61940540	CCAH5790.g1	GE296585
61940541	CCAH5791.b1	GE296586
61940542	CCAH5791.g1	GE296587
61940543	CCAH5792.b1	GE296588
61940544	CCAH5792.g1	GE296589
61940545	CCAH5793.b1	GE296590
61940546	CCAH5793.g1	GE296591
61940547	CCAH5794.b1	GE296592
61940548	CCAH5794.g1	GE296593
61940549	CCAH5795.b1	GE296594
61940550	CCAH5796.b1	GE296595
61940551	CCAH5796.g1	GE296596
61940552	CCAH5797.b1	GE296597
61940553	CCAH5798.b1	GE296598
61940554	CCAH5798.g1	GE296599
61940555	CCAH5799.b1	GE296600
61940556	CCAH5799.g1	GE296601
61940557	CCAH580.b6	GE296602
61940558	CCAH580.g6	GE296603
61940559	CCAH5800.b1	GE296604
61940560	CCAH5800.g1	GE296605
61940561	CCAH5801.b1	GE296606
61940562	CCAH5802.b1	GE296607
61940563	CCAH5802.g1	GE296608
61940564	CCAH5803.b1	GE296609
61940565	CCAH5803.g1	GE296610
61940566	CCAH5804.b1	GE296611
61940567	CCAH5804.g1	GE296612
61940568	CCAH5806.b1	GE296613
61940569	CCAH5806.g1	GE296614
61940570	CCAH5807.b1	GE296615
61940571	CCAH5807.g1	GE296616
61940572	CCAH5808.b1	GE296617
61940573	CCAH5808.g1	GE296618
61940574	CCAH5809.b1	GE296619
61940575	CCAH5809.g1	GE296620
61940576	CCAH5810.b1	GE296621
61940577	CCAH5811.b1	GE296622
61940578	CCAH5811.g1	GE296623
61940579	CCAH5812.b1	GE296624
61940580	CCAH5812.g1	GE296625
61940581	CCAH5813.b1	GE296626
61940582	CCAH5813.g1	GE296627
61940583	CCAH5814.b1	GE296628
61940584	CCAH5814.g1	GE296629
61940585	CCAH5815.b1	GE296630
61940586	CCAH5815.g1	GE296631
61940587	CCAH5816.b1	GE296632
61940588	CCAH5816.g1	GE296633
61940589	CCAH5817.b1	GE296634
61940590	CCAH5817.g1	GE296635
61940591	CCAH5818.b1	GE296636
61940592	CCAH5818.g1	GE296637
61940593	CCAH5819.b1	GE296638
61940594	CCAH5819.g1	GE296639
61940595	CCAH5820.b1	GE296640

61940596	CCAH5820.g1	GE296641
61940597	CCAH5821.b1	GE296642
61940598	CCAH5821.g1	GE296643
61940599	CCAH5822.b1	GE296644
61940600	CCAH5823.b1	GE296645
61940601	CCAH5825.g1	GE296646
61940602	CCAH5826.b1	GE296647
61940603	CCAH5827.b1	GE296648
61940604	CCAH5827.g1	GE296649
61940605	CCAH5828.b1	GE296650
61940606	CCAH5829.b1	GE296651
61940607	CCAH5829.g1	GE296652
61940608	CCAH5830.g1	GE296653
61940609	CCAH5831.b1	GE296654
61940610	CCAH5831.g1	GE296655
61940611	CCAH5832.b1	GE296656
61940612	CCAH5832.g1	GE296657
61940613	CCAH5833.b1	GE296658
61940614	CCAH5834.b1	GE296659
61940615	CCAH5834.g1	GE296660
61940616	CCAH5836.b1	GE296661
61940617	CCAH5837.b1	GE296662
61940618	CCAH5838.b1	GE296663
61940619	CCAH5838.g1	GE296664
61940620	CCAH5839.b1	GE296665
61940621	CCAH5840.b1	GE296666
61940622	CCAH5841.b1	GE296667
61940623	CCAH5841.g1	GE296668
61940624	CCAH5842.b1	GE296669
61940625	CCAH5842.g1	GE296670
61940626	CCAH5843.b1	GE296671
61940627	CCAH5843.g1	GE296672
61940628	CCAH5845.b1	GE296673
61940629	CCAH5845.g1	GE296674
61940630	CCAH5846.b1	GE296675
61940631	CCAH5846.g1	GE296676
61940632	CCAH5847.b1	GE296677
61940633	CCAH5847.g1	GE296678
61940634	CCAH5849.b1	GE296679
61940635	CCAH5850.b1	GE296680
61940636	CCAH5851.b1	GE296681
61940637	CCAH5852.b1	GE296682
61940638	CCAH5853.b1	GE296683
61940639	CCAH5853.g1	GE296684
61940640	CCAH5855.b1	GE296685
61940641	CCAH5855.g1	GE296686
61940642	CCAH5856.b1	GE296687
61940643	CCAH5857.b1	GE296688
61940644	CCAH5857.g1	GE296689
61940645	CCAH5859.b1	GE296690
61940646	CCAH5859.g1	GE296691
61940647	CCAH5860.b1	GE296692
61940648	CCAH5860.g1	GE296693
61940649	CCAH5861.b1	GE296694
61940650	CCAH5861.g1	GE296695
61940651	CCAH5862.b1	GE296696
61940652	CCAH5863.b1	GE296697
61940653	CCAH5863.g1	GE296698
61940654	CCAH5864.b1	GE296699
61940655	CCAH5867.b1	GE296700
61940656	CCAH5868.b1	GE296701
61940657	CCAH5868.g1	GE296702
61940658	CCAH5869.b1	GE296703

61940659	CCAH5869.g1	GE296704
61940660	CCAH5870.b1	GE296705
61940661	CCAH5871.b1	GE296706
61940662	CCAH5872.b1	GE296707
61940663	CCAH5872.g1	GE296708
61940664	CCAH5873.b1	GE296709
61940665	CCAH5873.g1	GE296710
61940666	CCAH5874.b1	GE296711
61940667	CCAH5875.b1	GE296712
61940668	CCAH5876.b1	GE296713
61940669	CCAH5876.g1	GE296714
61940670	CCAH5878.b1	GE296715
61940671	CCAH5878.g1	GE296716
61940672	CCAH5879.b1	GE296717
61940673	CCAH5880.g1	GE296718
61940674	CCAH5882.b1	GE296719
61940675	CCAH5882.g1	GE296720
61940676	CCAH5883.b1	GE296721
61940677	CCAH5883.g1	GE296722
61940678	CCAH5885.b1	GE296723
61940679	CCAH5885.g1	GE296724
61940680	CCAH5887.b1	GE296725
61940681	CCAH5887.g1	GE296726
61940682	CCAH5888.b1	GE296727
61940683	CCAH5888.g1	GE296728
61940684	CCAH5889.b1	GE296729
61940685	CCAH5889.g1	GE296730
61940686	CCAH5890.b1	GE296731
61940687	CCAH5891.b1	GE296732
61940688	CCAH5892.b1	GE296733
61940689	CCAH5892.g1	GE296734
61940690	CCAH5893.b1	GE296735
61940691	CCAH5893.g1	GE296736
61940692	CCAH5894.b1	GE296737
61940693	CCAH5895.b1	GE296738
61940694	CCAH5895.g1	GE296739
61940695	CCAH5896.b1	GE296740
61940696	CCAH5896.g1	GE296741
61940697	CCAH5897.b1	GE296742
61940698	CCAH5897.g1	GE296743
61940699	CCAH5898.b1	GE296744
61940700	CCAH5898.g1	GE296745
61940701	CCAH5899.b1	GE296746
61940702	CCAH5900.b1	GE296747
61940703	CCAH5900.g1	GE296748
61940704	CCAH5901.b1	GE296749
61940705	CCAH5901.g1	GE296750
61940706	CCAH5903.b1	GE296751
61940707	CCAH5903.g1	GE296752
61940708	CCAH5904.g1	GE296753
61940709	CCAH5905.b1	GE296754
61940710	CCAH5905.g1	GE296755
61940711	CCAH5906.b1	GE296756
61940712	CCAH5906.g1	GE296757
61940713	CCAH5907.b1	GE296758
61940714	CCAH5907.g1	GE296759
61940715	CCAH5909.b1	GE296760
61940716	CCAH5909.g1	GE296761
61940717	CCAH5910.b1	GE296762
61940718	CCAH5911.b1	GE296763
61940719	CCAH5911.g1	GE296764
61940720	CCAH5912.b1	GE296765
61940721	CCAH5914.b1	GE296766

61940722	CCAH5916.b1	GE296767
61940723	CCAH5916.g1	GE296768
61940724	CCAH5917.b1	GE296769
61940725	CCAH5917.g1	GE296770
61940726	CCAH5918.b1	GE296771
61940727	CCAH5919.b1	GE296772
61940728	CCAH5919.g1	GE296773
61940729	CCAH5920.b1	GE296774
61940730	CCAH5921.b1	GE296775
61940731	CCAH5921.g1	GE296776
61940732	CCAH5922.b1	GE296777
61940733	CCAH5924.b1	GE296778
61940734	CCAH5924.g1	GE296779
61940735	CCAH5925.b1	GE296780
61940736	CCAH5926.b1	GE296781
61940737	CCAH5926.g1	GE296782
61940738	CCAH5927.b1	GE296783
61940739	CCAH5927.g1	GE296784
61940740	CCAH5928.b1	GE296785
61940741	CCAH5928.g1	GE296786
61940742	CCAH5929.g1	GE296787
61940743	CCAH5931.b1	GE296788
61940744	CCAH5931.g1	GE296789
61940745	CCAH5932.b1	GE296790
61940746	CCAH5933.b1	GE296791
61940747	CCAH5933.g1	GE296792
61940748	CCAH5934.b1	GE296793
61940749	CCAH5934.g1	GE296794
61940750	CCAH5935.b1	GE296795
61940751	CCAH5935.g1	GE296796
61940752	CCAH5936.g1	GE296797
61940753	CCAH5937.b1	GE296798
61940754	CCAH5937.g1	GE296799
61940755	CCAH5938.b1	GE296800
61940756	CCAH5939.b1	GE296801
61940757	CCAH5939.g1	GE296802
61940758	CCAH5940.b1	GE296803
61940759	CCAH5940.g1	GE296804
61940760	CCAH5941.b1	GE296805
61940761	CCAH5941.g1	GE296806
61940762	CCAH5942.b1	GE296807
61940763	CCAH5942.g1	GE296808
61940764	CCAH5944.g1	GE296809
61940765	CCAH5945.b1	GE296810
61940766	CCAH5945.g1	GE296811
61940767	CCAH5946.b1	GE296812
61940768	CCAH5946.g1	GE296813
61940769	CCAH5947.b1	GE296814
61940770	CCAH5947.g1	GE296815
61940771	CCAH5948.b1	GE296816
61940772	CCAH5948.g1	GE296817
61940773	CCAH5949.b1	GE296818
61940774	CCAH5949.g1	GE296819
61940775	CCAH5950.b1	GE296820
61940776	CCAH5950.g1	GE296821
61940777	CCAH5951.b1	GE296822
61940778	CCAH5951.g1	GE296823
61940779	CCAH5953.b1	GE296824
61940780	CCAH5953.g1	GE296825
61940781	CCAH5954.b1	GE296826
61940782	CCAH5954.g1	GE296827
61940783	CCAH5955.b1	GE296828
61940784	CCAH5955.g1	GE296829

61940785	CCAH5956.b1	GE296830
61940786	CCAH5957.b1	GE296831
61940787	CCAH5958.b1	GE296832
61940788	CCAH5958.g1	GE296833
61940789	CCAH5959.g1	GE296834
61940790	CCAH5960.g1	GE296835
61940791	CCAH5962.b1	GE296836
61940792	CCAH5962.g1	GE296837
61940793	CCAH5963.b1	GE296838
61940794	CCAH5963.g1	GE296839
61940795	CCAH5964.b1	GE296840
61940796	CCAH5964.g1	GE296841
61940797	CCAH5965.b1	GE296842
61940798	CCAH5965.g1	GE296843
61940799	CCAH5966.b1	GE296844
61940800	CCAH5966.g1	GE296845
61940801	CCAH5967.b1	GE296846
61940802	CCAH5967.g1	GE296847
61940803	CCAH5970.b1	GE296848
61940804	CCAH5970.g1	GE296849
61940805	CCAH5971.b1	GE296850
61940806	CCAH5971.g1	GE296851
61940807	CCAH5972.b1	GE296852
61940808	CCAH5972.g1	GE296853
61940809	CCAH5973.b1	GE296854
61940810	CCAH5973.g1	GE296855
61940811	CCAH5974.b1	GE296856
61940812	CCAH5974.g1	GE296857
61940813	CCAH5975.b1	GE296858
61940814	CCAH5976.b1	GE296859
61940815	CCAH5977.b1	GE296860
61940816	CCAH5978.b1	GE296861
61940817	CCAH5979.b1	GE296862
61940818	CCAH5979.g1	GE296863
61940819	CCAH5980.b1	GE296864
61940820	CCAH5980.g1	GE296865
61940821	CCAH5981.b1	GE296866
61940822	CCAH5981.g1	GE296867
61940823	CCAH5982.b1	GE296868
61940824	CCAH5982.g1	GE296869
61940825	CCAH5985.b1	GE296870
61940826	CCAH5985.g1	GE296871
61940827	CCAH5986.b1	GE296872
61940828	CCAH5987.b1	GE296873
61940829	CCAH5987.g1	GE296874
61940830	CCAH5988.b1	GE296875
61940831	CCAH5988.g1	GE296876
61940832	CCAH5989.b1	GE296877
61940833	CCAH5989.g1	GE296878
61940834	CCAH5990.b1	GE296879
61940835	CCAH5990.g1	GE296880
61940836	CCAH5991.b1	GE296881
61940837	CCAH5992.b1	GE296882
61940838	CCAH5992.g1	GE296883
61940839	CCAH5993.b1	GE296884
61940840	CCAH5994.b1	GE296885
61940841	CCAH5994.g1	GE296886
61940842	CCAH5995.b1	GE296887
61940843	CCAH5995.g1	GE296888
61940844	CCAH5996.b1	GE296889
61940845	CCAH5996.g1	GE296890
61940846	CCAH5997.b1	GE296891
61940847	CCAH5997.g1	GE296892

61940848	CCAH5998.b1	GE296893
61940849	CCAH5998.g1	GE296894
61940850	CCAH5999.b1	GE296895
61940851	CCAH5999.g1	GE296896
61940852	CCAH6000.b1	GE296897
61940853	CCAH6000.g1	GE296898
61940854	CCAH6001.b1	GE296899
61940855	CCAH6002.b1	GE296900
61940856	CCAH6002.g1	GE296901
61940857	CCAH6003.b1	GE296902
61940858	CCAH6003.g1	GE296903
61940859	CCAH6004.b1	GE296904
61940860	CCAH6005.b1	GE296905
61940861	CCAH6005.g1	GE296906
61940862	CCAH6006.b1	GE296907
61940863	CCAH6007.b1	GE296908
61940864	CCAH6007.g1	GE296909
61940865	CCAH6008.b1	GE296910
61940866	CCAH6008.g1	GE296911
61940867	CCAH6009.b1	GE296912
61940868	CCAH6009.g1	GE296913
61940869	CCAH6010.b1	GE296914
61940870	CCAH6011.b1	GE296915
61940871	CCAH6011.g1	GE296916
61940872	CCAH6012.g1	GE296917
61940873	CCAH6014.b1	GE296918
61940874	CCAH6014.g1	GE296919
61940875	CCAH6016.b1	GE296920
61940876	CCAH6016.g1	GE296921
61940877	CCAH6017.b1	GE296922
61940878	CCAH6018.b1	GE296923
61940879	CCAH6018.g1	GE296924
61940880	CCAH6019.b1	GE296925
61940881	CCAH6020.b1	GE296926
61940882	CCAH6020.g1	GE296927
61940883	CCAH6021.b1	GE296928
61940884	CCAH6021.g1	GE296929
61940885	CCAH6022.b1	GE296930
61940886	CCAH6022.g1	GE296931
61940887	CCAH6023.b1	GE296932
61940888	CCAH6024.b1	GE296933
61940889	CCAH6026.b1	GE296934
61940890	CCAH6026.g1	GE296935
61940891	CCAH6028.b1	GE296936
61940892	CCAH6029.b1	GE296937
61940893	CCAH6030.b1	GE296938
61940894	CCAH6030.g1	GE296939
61940895	CCAH6031.b1	GE296940
61940896	CCAH6031.g1	GE296941
61940897	CCAH6032.b1	GE296942
61940898	CCAH6033.b1	GE296943
61940899	CCAH6034.b1	GE296944
61940900	CCAH6034.g1	GE296945
61940901	CCAH6035.b1	GE296946
61940902	CCAH6035.g1	GE296947
61940903	CCAH6036.b1	GE296948
61940904	CCAH6036.g1	GE296949
61940905	CCAH6037.b1	GE296950
61940906	CCAH6037.g1	GE296951
61940907	CCAH6038.b1	GE296952
61940908	CCAH6039.b1	GE296953
61940909	CCAH6039.g1	GE296954
61940910	CCAH6040.b1	GE296955

61940911	CCAH6040.g1	GE296956
61940912	CCAH6041.b1	GE296957
61940913	CCAH6042.b1	GE296958
61940914	CCAH6042.g1	GE296959
61940915	CCAH6043.b1	GE296960
61940916	CCAH6044.g1	GE296961
61940917	CCAH6045.b1	GE296962
61940918	CCAH6045.g1	GE296963
61940919	CCAH6046.b1	GE296964
61940920	CCAH6047.b1	GE296965
61940921	CCAH6050.b1	GE296966
61940922	CCAH6050.g1	GE296967
61940923	CCAH6051.b1	GE296968
61940924	CCAH6051.g1	GE296969
61940925	CCAH6052.b1	GE296970
61940926	CCAH6052.g1	GE296971
61940927	CCAH6053.b1	GE296972
61940928	CCAH6054.b1	GE296973
61940929	CCAH6054.g1	GE296974
61940930	CCAH6055.b1	GE296975
61940931	CCAH6057.b1	GE296976
61940932	CCAH6057.g1	GE296977
61940933	CCAH6058.b1	GE296978
61940934	CCAH6058.g1	GE296979
61940935	CCAH6059.b1	GE296980
61940936	CCAH6059.g1	GE296981
61940937	CCAH6062.b1	GE296982
61940938	CCAH6062.g1	GE296983
61940939	CCAH6063.b1	GE296984
61940940	CCAH6064.b1	GE296985
61940941	CCAH6064.g1	GE296986
61940942	CCAH6065.b1	GE296987
61940943	CCAH6065.g1	GE296988
61940944	CCAH6066.b1	GE296989
61940945	CCAH6066.g1	GE296990
61940946	CCAH6067.b1	GE296991
61940947	CCAH6067.g1	GE296992
61940948	CCAH6068.b1	GE296993
61940949	CCAH6068.g1	GE296994
61940950	CCAH6069.b1	GE296995
61940951	CCAH6069.g1	GE296996
61940952	CCAH6070.b1	GE296997
61940953	CCAH6070.g1	GE296998
61940954	CCAH6071.b1	GE296999
61940955	CCAH6071.g1	GE297000
61940956	CCAH6073.b1	GE297001
61940957	CCAH6073.g1	GE297002
61940958	CCAH6074.b1	GE297003
61940959	CCAH6075.b1	GE297004
61940960	CCAH6075.g1	GE297005
61940961	CCAH6076.g1	GE297006
61940962	CCAH6077.b1	GE297007
61940963	CCAH6078.b1	GE297008
61940964	CCAH6079.b1	GE297009
61940965	CCAH6079.g1	GE297010
61940966	CCAH6080.b1	GE297011
61940967	CCAH6080.g1	GE297012
61940968	CCAH6081.b1	GE297013
61940969	CCAH6081.g1	GE297014
61940970	CCAH6082.b1	GE297015
61940971	CCAH6082.g1	GE297016
61940972	CCAH6083.b1	GE297017
61940973	CCAH6083.g1	GE297018

61940974	CCAH6084.b1	GE297019
61940975	CCAH6084.g1	GE297020
61940976	CCAH6085.b1	GE297021
61940977	CCAH6086.b1	GE297022
61940978	CCAH6086.g1	GE297023
61940979	CCAH6087.b1	GE297024
61940980	CCAH6087.g1	GE297025
61940981	CCAH6088.b1	GE297026
61940982	CCAH6088.g1	GE297027
61940983	CCAH6089.b1	GE297028
61940984	CCAH6090.b1	GE297029
61940985	CCAH6090.g1	GE297030
61940986	CCAH6092.b1	GE297031
61940987	CCAH6093.b1	GE297032
61940988	CCAH6093.g1	GE297033
61940989	CCAH6094.b1	GE297034
61940990	CCAH6094.g1	GE297035
61940991	CCAH6095.g1	GE297036
61940992	CCAH6096.b1	GE297037
61940993	CCAH6096.g1	GE297038
61940994	CCAH6097.b1	GE297039
61940995	CCAH6097.g1	GE297040
61940996	CCAH6098.b1	GE297041
61940997	CCAH6098.g1	GE297042
61940998	CCAH6099.b1	GE297043
61940999	CCAH6100.b1	GE297044
61941000	CCAH6100.g1	GE297045
61941001	CCAH6101.b1	GE297046
61941002	CCAH6101.g1	GE297047
61941003	CCAH6102.b1	GE297048
61941004	CCAH6102.g1	GE297049
61941005	CCAH6103.b1	GE297050
61941006	CCAH6103.g1	GE297051
61941007	CCAH6104.b1	GE297052
61941008	CCAH6104.g1	GE297053
61941009	CCAH6105.b1	GE297054
61941010	CCAH6105.g1	GE297055
61941011	CCAH6106.b1	GE297056
61941012	CCAH6106.g1	GE297057
61941013	CCAH6107.b1	GE297058
61941014	CCAH6108.b1	GE297059
61941015	CCAH6109.b1	GE297060
61941016	CCAH6109.g1	GE297061
61941017	CCAH6110.b1	GE297062
61941018	CCAH6110.g1	GE297063
61941019	CCAH6111.b1	GE297064
61941020	CCAH6111.g1	GE297065
61941021	CCAH6112.b1	GE297066
61941022	CCAH6112.g1	GE297067
61941023	CCAH6113.b1	GE297068
61941024	CCAH6113.g1	GE297069
61941025	CCAH6114.b1	GE297070
61941026	CCAH6114.g1	GE297071
61941027	CCAH6116.b1	GE297072
61941028	CCAH6116.g1	GE297073
61941029	CCAH6117.b1	GE297074
61941030	CCAH6117.g1	GE297075
61941031	CCAH6118.b1	GE297076
61941032	CCAH6118.g1	GE297077
61941033	CCAH6119.g1	GE297078
61941034	CCAH6120.b1	GE297079
61941035	CCAH6121.g1	GE297080
61941036	CCAH6122.b1	GE297081

61941037	CCAH6123.b1	GE297082
61941038	CCAH6123.g1	GE297083
61941039	CCAH6125.b1	GE297084
61941040	CCAH6125.g1	GE297085
61941041	CCAH6126.b1	GE297086
61941042	CCAH6126.g1	GE297087
61941043	CCAH6127.b1	GE297088
61941044	CCAH6128.b1	GE297089
61941045	CCAH6128.g1	GE297090
61941046	CCAH6129.b1	GE297091
61941047	CCAH6129.g1	GE297092
61941048	CCAH6130.b1	GE297093
61941049	CCAH6130.g1	GE297094
61941050	CCAH6131.b1	GE297095
61941051	CCAH6131.g1	GE297096
61941052	CCAH6132.b1	GE297097
61941053	CCAH6132.g1	GE297098
61941054	CCAH6134.g1	GE297099
61941055	CCAH6136.b1	GE297100
61941056	CCAH6137.b1	GE297101
61941057	CCAH6138.b1	GE297102
61941058	CCAH6138.g1	GE297103
61941059	CCAH6139.b1	GE297104
61941060	CCAH6140.b1	GE297105
61941061	CCAH6140.g1	GE297106
61941062	CCAH6141.b1	GE297107
61941063	CCAH6141.g1	GE297108
61941064	CCAH6142.b1	GE297109
61941065	CCAH6142.g1	GE297110
61941066	CCAH6143.g1	GE297111
61941067	CCAH6146.b1	GE297112
61941068	CCAH6146.g1	GE297113
61941069	CCAH6147.b1	GE297114
61941070	CCAH6147.g1	GE297115
61941071	CCAH6148.b1	GE297116
61941072	CCAH6148.g1	GE297117
61941073	CCAH6149.b1	GE297118
61941074	CCAH6149.g1	GE297119
61941075	CCAH6150.b1	GE297120
61941076	CCAH6150.g1	GE297121
61941077	CCAH6153.b1	GE297122
61941078	CCAH6153.g1	GE297123
61941079	CCAH6154.b1	GE297124
61941080	CCAH6154.g1	GE297125
61941081	CCAH6155.b1	GE297126
61941082	CCAH6155.g1	GE297127
61941083	CCAH6156.b1	GE297128
61941084	CCAH6156.g1	GE297129
61941085	CCAH6157.b1	GE297130
61941086	CCAH6157.g1	GE297131
61941087	CCAH6158.b1	GE297132
61941088	CCAH6158.g1	GE297133
61941089	CCAH6159.b1	GE297134
61941090	CCAH6159.g1	GE297135
61941091	CCAH6160.b1	GE297136
61941092	CCAH6160.g1	GE297137
61941093	CCAH6161.b1	GE297138
61941094	CCAH6161.g1	GE297139
61941095	CCAH6162.b1	GE297140
61941096	CCAH6162.g1	GE297141
61941097	CCAH6163.b1	GE297142
61941098	CCAH6164.b1	GE297143
61941099	CCAH6164.g1	GE297144

61941100	CCAH6165.b1	GE297145
61941101	CCAH6165.g1	GE297146
61941102	CCAH6166.b1	GE297147
61941103	CCAH6166.g1	GE297148
61941104	CCAH6167.b1	GE297149
61941105	CCAH6167.g1	GE297150
61941106	CCAH6168.b1	GE297151
61941107	CCAH6168.g1	GE297152
61941108	CCAH6169.b1	GE297153
61941109	CCAH6169.g1	GE297154
61941110	CCAH6172.b1	GE297155
61941111	CCAH6172.g1	GE297156
61941112	CCAH6173.b1	GE297157
61941113	CCAH6173.g1	GE297158
61941114	CCAH6174.b1	GE297159
61941115	CCAH6175.b1	GE297160
61941116	CCAH6176.b1	GE297161
61941117	CCAH6177.b1	GE297162
61941118	CCAH6178.g1	GE297163
61941119	CCAH6179.b1	GE297164
61941120	CCAH6179.g1	GE297165
61941121	CCAH6180.b1	GE297166
61941122	CCAH6180.g1	GE297167
61941123	CCAH6181.b1	GE297168
61941124	CCAH6181.g1	GE297169
61941125	CCAH6183.b1	GE297170
61941126	CCAH6183.g1	GE297171
61941127	CCAH6185.b1	GE297172
61941128	CCAH6185.g1	GE297173
61941129	CCAH6186.b1	GE297174
61941130	CCAH6186.g1	GE297175
61941131	CCAH6187.b1	GE297176
61941132	CCAH6187.g1	GE297177
61941133	CCAH6188.b1	GE297178
61941134	CCAH6189.g1	GE297179
61941135	CCAH6191.b1	GE297180
61941136	CCAH6191.g1	GE297181
61941137	CCAH6193.b1	GE297182
61941138	CCAH6193.g1	GE297183
61941139	CCAH6194.g1	GE297184
61941140	CCAH6195.b1	GE297185
61941141	CCAH6196.b1	GE297186
61941142	CCAH6196.g1	GE297187
61941143	CCAH6197.b1	GE297188
61941144	CCAH6197.g1	GE297189
61941145	CCAH6198.b1	GE297190
61941146	CCAH6199.b1	GE297191
61941147	CCAH6199.g1	GE297192
61941148	CCAH6200.b1	GE297193
61941149	CCAH6200.g1	GE297194
61941150	CCAH6202.b1	GE297195
61941151	CCAH6203.b1	GE297196
61941152	CCAH6203.g1	GE297197
61941153	CCAH6204.b1	GE297198
61941154	CCAH6204.g1	GE297199
61941155	CCAH6205.b1	GE297200
61941156	CCAH6205.g1	GE297201
61941157	CCAH6206.b1	GE297202
61941158	CCAH6206.g1	GE297203
61941159	CCAH6207.b1	GE297204
61941160	CCAH6207.g1	GE297205
61941161	CCAH6208.b1	GE297206
61941162	CCAH6208.g1	GE297207

61941163	CCAH6209.b1	GE297208
61941164	CCAH6209.g1	GE297209
61941165	CCAH6210.b1	GE297210
61941166	CCAH6211.b1	GE297211
61941167	CCAH6211.g1	GE297212
61941168	CCAH6212.b1	GE297213
61941169	CCAH6212.g1	GE297214
61941170	CCAH6213.b1	GE297215
61941171	CCAH6214.b1	GE297216
61941172	CCAH6215.b1	GE297217
61941173	CCAH6215.g1	GE297218
61941174	CCAH6217.b1	GE297219
61941175	CCAH6217.g1	GE297220
61941176	CCAH6218.b1	GE297221
61941177	CCAH6219.b1	GE297222
61941178	CCAH6219.g1	GE297223
61941179	CCAH6220.b1	GE297224
61941180	CCAH6220.g1	GE297225
61941181	CCAH6222.b1	GE297226
61941182	CCAH6222.g1	GE297227
61941183	CCAH6223.b1	GE297228
61941184	CCAH6223.g1	GE297229
61941185	CCAH6224.g1	GE297230
61941186	CCAH6225.g1	GE297231
61941187	CCAH6226.b1	GE297232
61941188	CCAH6226.g1	GE297233
61941189	CCAH6227.b1	GE297234
61941190	CCAH6227.g1	GE297235
61941191	CCAH6228.b1	GE297236
61941192	CCAH6228.g1	GE297237
61941193	CCAH6229.b1	GE297238
61941194	CCAH6229.g1	GE297239
61941195	CCAH6230.b1	GE297240
61941196	CCAH6230.g1	GE297241
61941197	CCAH6231.b1	GE297242
61941198	CCAH6232.b1	GE297243
61941199	CCAH6233.b1	GE297244
61941200	CCAH6233.g1	GE297245
61941201	CCAH6234.b1	GE297246
61941202	CCAH6234.g1	GE297247
61941203	CCAH6235.b1	GE297248
61941204	CCAH6236.b1	GE297249
61941205	CCAH6236.g1	GE297250
61941206	CCAH6237.b1	GE297251
61941207	CCAH6238.b1	GE297252
61941208	CCAH6239.b1	GE297253
61941209	CCAH6240.b1	GE297254
61941210	CCAH6240.g1	GE297255
61941211	CCAH6241.b1	GE297256
61941212	CCAH6241.g1	GE297257
61941213	CCAH6242.b1	GE297258
61941214	CCAH6242.g1	GE297259
61941215	CCAH6243.b1	GE297260
61941216	CCAH6243.g1	GE297261
61941217	CCAH6244.b1	GE297262
61941218	CCAH6244.g1	GE297263
61941219	CCAH6246.b1	GE297264
61941220	CCAH6246.g1	GE297265
61941221	CCAH6247.g1	GE297266
61941222	CCAH6248.b1	GE297267
61941223	CCAH6249.g1	GE297268
61941224	CCAH6250.g1	GE297269
61941225	CCAH6251.b1	GE297270

61941226	CCAH6251.g1	GE297271
61941227	CCAH6252.b1	GE297272
61941228	CCAH6252.g1	GE297273
61941229	CCAH6253.b1	GE297274
61941230	CCAH6253.g1	GE297275
61941231	CCAH6254.b1	GE297276
61941232	CCAH6254.g1	GE297277
61941233	CCAH6255.b1	GE297278
61941234	CCAH6255.g1	GE297279
61941235	CCAH6256.b1	GE297280
61941236	CCAH6256.g1	GE297281
61941237	CCAH6257.b1	GE297282
61941238	CCAH6257.g1	GE297283
61941239	CCAH6258.b1	GE297284
61941240	CCAH6258.g1	GE297285
61941241	CCAH6259.g1	GE297286
61941242	CCAH6261.g1	GE297287
61941243	CCAH6262.b1	GE297288
61941244	CCAH6262.g1	GE297289
61941245	CCAH6263.b1	GE297290
61941246	CCAH6264.b1	GE297291
61941247	CCAH6264.g1	GE297292
61941248	CCAH6265.b1	GE297293
61941249	CCAH6265.g1	GE297294
61941250	CCAH6267.b1	GE297295
61941251	CCAH6267.g1	GE297296
61941252	CCAH6268.b1	GE297297
61941253	CCAH6268.g1	GE297298
61941254	CCAH6269.b1	GE297299
61941255	CCAH6270.b1	GE297300
61941256	CCAH6270.g1	GE297301
61941257	CCAH6271.b1	GE297302
61941258	CCAH6271.g1	GE297303
61941259	CCAH6272.b1	GE297304
61941260	CCAH6272.g1	GE297305
61941261	CCAH6274.b1	GE297306
61941262	CCAH6275.b1	GE297307
61941263	CCAH6276.b1	GE297308
61941264	CCAH6276.g1	GE297309
61941265	CCAH6277.b1	GE297310
61941266	CCAH6277.g1	GE297311
61941267	CCAH6278.b1	GE297312
61941268	CCAH6281.b1	GE297313
61941269	CCAH6282.b1	GE297314
61941270	CCAH6282.g1	GE297315
61941271	CCAH6283.b1	GE297316
61941272	CCAH6283.g1	GE297317
61941273	CCAH6284.b1	GE297318
61941274	CCAH6284.g1	GE297319
61941275	CCAH6285.b1	GE297320
61941276	CCAH6285.g1	GE297321
61941277	CCAH6286.b1	GE297322
61941278	CCAH6286.g1	GE297323
61941279	CCAH6287.b1	GE297324
61941280	CCAH6287.g1	GE297325
61941281	CCAH6288.b1	GE297326
61941282	CCAH6288.g1	GE297327
61941283	CCAH6289.b1	GE297328
61941284	CCAH6291.b1	GE297329
61941285	CCAH6291.g1	GE297330
61941286	CCAH6292.b1	GE297331
61941287	CCAH6292.g1	GE297332
61941288	CCAH6293.b1	GE297333

61941289	CCAH6293.g1	GE297334
61941290	CCAH6294.b1	GE297335
61941291	CCAH6294.g1	GE297336
61941292	CCAH6295.b1	GE297337
61941293	CCAH6295.g1	GE297338
61941294	CCAH6296.g1	GE297339
61941295	CCAH6297.b1	GE297340
61941296	CCAH6298.b1	GE297341
61941297	CCAH6298.g1	GE297342
61941298	CCAH6299.b1	GE297343
61941299	CCAH6299.g1	GE297344
61941300	CCAH6300.b1	GE297345
61941301	CCAH6300.g1	GE297346
61941302	CCAH6301.b1	GE297347
61941303	CCAH6301.g1	GE297348
61941304	CCAH6302.g1	GE297349
61941305	CCAH6303.b1	GE297350
61941306	CCAH6303.g1	GE297351
61941307	CCAH6304.b1	GE297352
61941308	CCAH6304.g1	GE297353
61941309	CCAH6305.b1	GE297354
61941310	CCAH6305.g1	GE297355
61941311	CCAH6306.b1	GE297356
61941312	CCAH6306.g1	GE297357
61941313	CCAH6307.b1	GE297358
61941314	CCAH6307.g1	GE297359
61941315	CCAH6308.b1	GE297360
61941316	CCAH6308.g1	GE297361
61941317	CCAH6309.b1	GE297362
61941318	CCAH6309.g1	GE297363
61941319	CCAH6310.b1	GE297364
61941320	CCAH6310.g1	GE297365
61941321	CCAH6311.g1	GE297366
61941322	CCAH6312.b1	GE297367
61941323	CCAH6313.b1	GE297368
61941324	CCAH6313.g1	GE297369
61941325	CCAH6314.b1	GE297370
61941326	CCAH6314.g1	GE297371
61941327	CCAH6315.b1	GE297372
61941328	CCAH6315.g1	GE297373
61941329	CCAH6316.b1	GE297374
61941330	CCAH6316.g1	GE297375
61941331	CCAH6317.g1	GE297376
61941332	CCAH6318.b1	GE297377
61941333	CCAH6318.g1	GE297378
61941334	CCAH6319.b1	GE297379
61941335	CCAH6319.g1	GE297380
61941336	CCAH6321.b1	GE297381
61941337	CCAH6321.g1	GE297382
61941338	CCAH6322.b1	GE297383
61941339	CCAH6323.b1	GE297384
61941340	CCAH6323.g1	GE297385
61941341	CCAH6324.b1	GE297386
61941342	CCAH6324.g1	GE297387
61941343	CCAH6325.b1	GE297388
61941344	CCAH6325.g1	GE297389
61941345	CCAH6327.b1	GE297390
61941346	CCAH6327.g1	GE297391
61941347	CCAH6328.g1	GE297392
61941348	CCAH6329.b1	GE297393
61941349	CCAH6329.g1	GE297394
61941350	CCAH6330.b1	GE297395
61941351	CCAH6330.g1	GE297396

61941352	CCAH6331.b1	GE297397
61941353	CCAH6331.g1	GE297398
61941354	CCAH6332.g1	GE297399
61941355	CCAH6333.g1	GE297400
61941356	CCAH6334.b1	GE297401
61941357	CCAH6334.g1	GE297402
61941358	CCAH6335.b1	GE297403
61941359	CCAH6335.g1	GE297404
61941360	CCAH6337.b1	GE297405
61941361	CCAH6337.g1	GE297406
61941362	CCAH6338.b1	GE297407
61941363	CCAH6339.b1	GE297408
61941364	CCAH6339.g1	GE297409
61941365	CCAH6340.b1	GE297410
61941366	CCAH6340.g1	GE297411
61941367	CCAH6341.b1	GE297412
61941368	CCAH6341.g1	GE297413
61941369	CCAH6342.b1	GE297414
61941370	CCAH6343.b1	GE297415
61941371	CCAH6343.g1	GE297416
61941372	CCAH6344.b1	GE297417
61941373	CCAH6346.b1	GE297418
61941374	CCAH6346.g1	GE297419
61941375	CCAH6347.b1	GE297420
61941376	CCAH6347.g1	GE297421
61941377	CCAH6350.b1	GE297422
61941378	CCAH6350.g1	GE297423
61941379	CCAH6351.b1	GE297424
61941380	CCAH6351.g1	GE297425
61941381	CCAH6352.b1	GE297426
61941382	CCAH6352.g1	GE297427
61941383	CCAH6353.b1	GE297428
61941384	CCAH6353.g1	GE297429
61941385	CCAH6354.b1	GE297430
61941386	CCAH6354.g1	GE297431
61941387	CCAH6355.b1	GE297432
61941388	CCAH6356.b1	GE297433
61941389	CCAH6356.g1	GE297434
61941390	CCAH6357.b1	GE297435
61941391	CCAH6357.g1	GE297436
61941392	CCAH6358.b1	GE297437
61941393	CCAH6358.g1	GE297438
61941394	CCAH6359.g1	GE297439
61941395	CCAH6360.b1	GE297440
61941396	CCAH6360.g1	GE297441
61941397	CCAH6361.b1	GE297442
61941398	CCAH6361.g1	GE297443
61941399	CCAH6362.b1	GE297444
61941400	CCAH6362.g1	GE297445
61941401	CCAH6363.b1	GE297446
61941402	CCAH6363.g1	GE297447
61941403	CCAH6364.b1	GE297448
61941404	CCAH6364.g1	GE297449
61941405	CCAH6365.b1	GE297450
61941406	CCAH6366.b1	GE297451
61941407	CCAH6366.g1	GE297452
61941408	CCAH6367.b1	GE297453
61941409	CCAH6367.g1	GE297454
61941410	CCAH6368.b1	GE297455
61941411	CCAH6368.g1	GE297456
61941412	CCAH6369.b1	GE297457
61941413	CCAH6369.g1	GE297458
61941414	CCAH6370.g1	GE297459

61941415	CCAH6371.b1	GE297460
61941416	CCAH6371.g1	GE297461
61941417	CCAH6372.b1	GE297462
61941418	CCAH6372.g1	GE297463
61941419	CCAH6374.b1	GE297464
61941420	CCAH6374.g1	GE297465
61941421	CCAH6377.b1	GE297466
61941422	CCAH6377.g1	GE297467
61941423	CCAH6378.b1	GE297468
61941424	CCAH6378.g1	GE297469
61941425	CCAH6379.b1	GE297470
61941426	CCAH6379.g1	GE297471
61941427	CCAH6380.b1	GE297472
61941428	CCAH6380.g1	GE297473
61941429	CCAH6381.b1	GE297474
61941430	CCAH6381.g1	GE297475
61941431	CCAH6382.b1	GE297476
61941432	CCAH6382.g1	GE297477
61941433	CCAH6383.b1	GE297478
61941434	CCAH6383.g1	GE297479
61941435	CCAH6384.b1	GE297480
61941436	CCAH6384.g1	GE297481
61941437	CCAH6385.b1	GE297482
61941438	CCAH6385.g1	GE297483
61941439	CCAH6386.b1	GE297484
61941440	CCAH6386.g1	GE297485
61941441	CCAH6387.b1	GE297486
61941442	CCAH6387.g1	GE297487
61941443	CCAH6388.b1	GE297488
61941444	CCAH6389.b1	GE297489
61941445	CCAH6389.g1	GE297490
61941446	CCAH6390.b1	GE297491
61941447	CCAH6390.g1	GE297492
61941448	CCAH6391.b1	GE297493
61941449	CCAH6391.g1	GE297494
61941450	CCAH6392.b1	GE297495
61941451	CCAH6392.g1	GE297496
61941452	CCAH6393.b1	GE297497
61941453	CCAH6393.g1	GE297498
61941454	CCAH6394.b1	GE297499
61941455	CCAH6395.b1	GE297500
61941456	CCAH6395.g1	GE297501
61941457	CCAH6396.b1	GE297502
61941458	CCAH6396.g1	GE297503
61941459	CCAH6397.b1	GE297504
61941460	CCAH6397.g1	GE297505
61941461	CCAH6398.b1	GE297506
61941462	CCAH6398.g1	GE297507
61941463	CCAH6399.g1	GE297508
61941464	CCAH6401.b1	GE297509
61941465	CCAH6401.g1	GE297510
61941466	CCAH6402.b1	GE297511
61941467	CCAH6402.g1	GE297512
61941468	CCAH6403.b1	GE297513
61941469	CCAH6403.g1	GE297514
61941470	CCAH6404.b1	GE297515
61941471	CCAH6405.b1	GE297516
61941472	CCAH6405.g1	GE297517
61941473	CCAH6406.b1	GE297518
61941474	CCAH6406.g1	GE297519
61941475	CCAH6407.g1	GE297520
61941476	CCAH6408.b1	GE297521
61941477	CCAH6408.g1	GE297522

61941478	CCAH6409.b1	GE297523
61941479	CCAH6409.g1	GE297524
61941480	CCAH6411.b1	GE297525
61941481	CCAH6411.g1	GE297526
61941482	CCAH6412.g1	GE297527
61941483	CCAH6413.b1	GE297528
61941484	CCAH6413.g1	GE297529
61941485	CCAH6414.b1	GE297530
61941486	CCAH6414.g1	GE297531
61941487	CCAH6415.b1	GE297532
61941488	CCAH6415.g1	GE297533
61941489	CCAH6416.b1	GE297534
61941490	CCAH6417.b1	GE297535
61941491	CCAH6418.b1	GE297536
61941492	CCAH6418.g1	GE297537
61941493	CCAH6419.b1	GE297538
61941494	CCAH6419.g1	GE297539
61941495	CCAH6420.b1	GE297540
61941496	CCAH6420.g1	GE297541
61941497	CCAH6421.b1	GE297542
61941498	CCAH6421.g1	GE297543
61941499	CCAH6422.b1	GE297544
61941500	CCAH6422.g1	GE297545
61941501	CCAH6425.b1	GE297546
61941502	CCAH6425.g1	GE297547
61941503	CCAH6426.b1	GE297548
61941504	CCAH6427.g1	GE297549
61941505	CCAH6428.b1	GE297550
61941506	CCAH6428.g1	GE297551
61941507	CCAH6429.b1	GE297552
61941508	CCAH6429.g1	GE297553
61941509	CCAH6430.b1	GE297554
61941510	CCAH6430.g1	GE297555
61941511	CCAH6431.b1	GE297556
61941512	CCAH6431.g1	GE297557
61941513	CCAH6433.b1	GE297558
61941514	CCAH6433.g1	GE297559
61941515	CCAH6434.b1	GE297560
61941516	CCAH6434.g1	GE297561
61941517	CCAH6435.b1	GE297562
61941518	CCAH6435.g1	GE297563
61941519	CCAH6436.b1	GE297564
61941520	CCAH6436.g1	GE297565
61941521	CCAH6437.b1	GE297566
61941522	CCAH6437.g1	GE297567
61941523	CCAH6438.b1	GE297568
61941524	CCAH6438.g1	GE297569
61941525	CCAH6439.b1	GE297570
61941526	CCAH6439.g1	GE297571
61941527	CCAH6440.b1	GE297572
61941528	CCAH6441.g1	GE297573
61941529	CCAH6442.b1	GE297574
61941530	CCAH6442.g1	GE297575
61941531	CCAH6443.b1	GE297576
61941532	CCAH6443.g1	GE297577
61941533	CCAH6444.b1	GE297578
61941534	CCAH6444.g1	GE297579
61941535	CCAH6445.b1	GE297580
61941536	CCAH6445.g1	GE297581
61941537	CCAH6446.b1	GE297582
61941538	CCAH6446.g1	GE297583
61941539	CCAH6447.b1	GE297584
61941540	CCAH6448.b1	GE297585

61941541	CCAH6448.g1	GE297586
61941542	CCAH6449.g1	GE297587
61941543	CCAH6450.b1	GE297588
61941544	CCAH6450.g1	GE297589
61941545	CCAH6452.b1	GE297590
61941546	CCAH6452.g1	GE297591
61941547	CCAH6453.g1	GE297592
61941548	CCAH6454.b1	GE297593
61941549	CCAH6454.g1	GE297594
61941550	CCAH6455.b1	GE297595
61941551	CCAH6455.g1	GE297596
61941552	CCAH6457.b1	GE297597
61941553	CCAH6457.g1	GE297598
61941554	CCAH6458.b1	GE297599
61941555	CCAH6458.g1	GE297600
61941556	CCAH6459.b1	GE297601
61941557	CCAH6459.g1	GE297602
61941558	CCAH6460.b1	GE297603
61941559	CCAH6461.b1	GE297604
61941560	CCAH6461.g1	GE297605
61941561	CCAH6462.b1	GE297606
61941562	CCAH6462.g1	GE297607
61941563	CCAH6464.b1	GE297608
61941564	CCAH6464.g1	GE297609
61941565	CCAH6465.b1	GE297610
61941566	CCAH6465.g1	GE297611
61941567	CCAH6466.b1	GE297612
61941568	CCAH6466.g1	GE297613
61941569	CCAH6467.b1	GE297614
61941570	CCAH6467.g1	GE297615
61941571	CCAH6468.b1	GE297616
61941572	CCAH6468.g1	GE297617
61941573	CCAH6469.g1	GE297618
61941574	CCAH6470.b1	GE297619
61941575	CCAH6471.b1	GE297620
61941576	CCAH6471.g1	GE297621
61941577	CCAH6472.b1	GE297622
61941578	CCAH6472.g1	GE297623
61941579	CCAH6473.b1	GE297624
61941580	CCAH6473.g1	GE297625
61941581	CCAH6474.b1	GE297626
61941582	CCAH6475.b1	GE297627
61941583	CCAH6475.g1	GE297628
61941584	CCAH6476.b1	GE297629
61941585	CCAH6477.b1	GE297630
61941586	CCAH6478.g1	GE297631
61941587	CCAH6479.b1	GE297632
61941588	CCAH6479.g1	GE297633
61941589	CCAH6480.b1	GE297634
61941590	CCAH6480.g1	GE297635
61941591	CCAH6481.b1	GE297636
61941592	CCAH6482.b1	GE297637
61941593	CCAH6482.g1	GE297638
61941594	CCAH6483.b1	GE297639
61941595	CCAH6483.g1	GE297640
61941596	CCAH6485.b1	GE297641
61941597	CCAH6485.g1	GE297642
61941598	CCAH6486.b1	GE297643
61941599	CCAH6486.g1	GE297644
61941600	CCAH6488.b1	GE297645
61941601	CCAH6488.g1	GE297646
61941602	CCAH6489.b1	GE297647
61941603	CCAH6489.g1	GE297648

61941604	CCAH6490.b1	GE297649
61941605	CCAH6490.g1	GE297650
61941606	CCAH6491.b1	GE297651
61941607	CCAH6491.g1	GE297652
61941608	CCAH6492.b1	GE297653
61941609	CCAH6492.g1	GE297654
61941610	CCAH6493.b1	GE297655
61941611	CCAH6493.g1	GE297656
61941612	CCAH6494.b1	GE297657
61941613	CCAH6494.g1	GE297658
61941614	CCAH6495.b1	GE297659
61941615	CCAH6495.g1	GE297660
61941616	CCAH6496.b1	GE297661
61941617	CCAH6496.g1	GE297662
61941618	CCAH6497.b1	GE297663
61941619	CCAH6497.g1	GE297664
61941620	CCAH6498.b1	GE297665
61941621	CCAH6498.g1	GE297666
61941622	CCAH6499.b1	GE297667
61941623	CCAH6499.g1	GE297668
61941624	CCAH6500.b1	GE297669
61941625	CCAH6501.b1	GE297670
61941626	CCAH6501.g1	GE297671
61941627	CCAH6502.b1	GE297672
61941628	CCAH6502.g1	GE297673
61941629	CCAH6503.g1	GE297674
61941630	CCAH6504.b1	GE297675
61941631	CCAH6504.g1	GE297676
61941632	CCAH6505.g1	GE297677
61941633	CCAH6506.b1	GE297678
61941634	CCAH6507.b1	GE297679
61941635	CCAH6507.g1	GE297680
61941636	CCAH6508.b1	GE297681
61941637	CCAH6508.g1	GE297682
61941638	CCAH6509.b1	GE297683
61941639	CCAH6510.b1	GE297684
61941640	CCAH6510.g1	GE297685
61941641	CCAH6511.b1	GE297686
61941642	CCAH6512.b1	GE297687
61941643	CCAH6512.g1	GE297688
61941644	CCAH6513.b1	GE297689
61941645	CCAH6513.g1	GE297690
61941646	CCAH6514.b1	GE297691
61941647	CCAH6514.g1	GE297692
61941648	CCAH6515.g1	GE297693
61941649	CCAH6516.b1	GE297694
61941650	CCAH6516.g1	GE297695
61941651	CCAH6518.b1	GE297696
61941652	CCAH6518.g1	GE297697
61941653	CCAH6519.b1	GE297698
61941654	CCAH6519.g1	GE297699
61941655	CCAH6521.b1	GE297700
61941656	CCAH6521.g1	GE297701
61941657	CCAH6522.g1	GE297702
61941658	CCAH6523.b1	GE297703
61941659	CCAH6523.g1	GE297704
61941660	CCAH6524.b1	GE297705
61941661	CCAH6524.g1	GE297706
61941662	CCAH6525.b1	GE297707
61941663	CCAH6525.g1	GE297708
61941664	CCAH6526.b1	GE297709
61941665	CCAH6526.g1	GE297710
61941666	CCAH6527.b1	GE297711

61941667	CCAH769.b1	GE297712
61941668	CCAH769.g1	GE297713
61941669	CCAH770.b1	GE297714
61941670	CCAH771.b1	GE297715
61941671	CCAH771.g1	GE297716
61941672	CCAH772.g1	GE297717
61941673	CCAH773.b1	GE297718
61941674	CCAH773.g1	GE297719
61941675	CCAH774.b1	GE297720
61941676	CCAH774.g1	GE297721
61941677	CCAH775.b1	GE297722
61941678	CCAH775.g1	GE297723
61941679	CCAH776.b1	GE297724
61941680	CCAH776.g1	GE297725
61941681	CCAH777.b1	GE297726
61941682	CCAH777.g1	GE297727
61941683	CCAH778.b1	GE297728
61941684	CCAH779.b1	GE297729
61941685	CCAH779.g1	GE297730
61941686	CCAH780.b1	GE297731
61941687	CCAH780.g1	GE297732
61941688	CCAH781.b1	GE297733
61941689	CCAH781.g1	GE297734
61941690	CCAH782.b1	GE297735
61941691	CCAH782.g1	GE297736
61941692	CCAH783.b1	GE297737
61941693	CCAH783.g1	GE297738
61941694	CCAH784.b1	GE297739
61941695	CCAH784.g1	GE297740
61941696	CCAH785.b1	GE297741
61941697	CCAH785.g1	GE297742
61941698	CCAH786.b1	GE297743
61941699	CCAH786.g1	GE297744
61941700	CCAH787.b1	GE297745
61941701	CCAH787.g1	GE297746
61941702	CCAH788.b1	GE297747
61941703	CCAH788.g1	GE297748
61941704	CCAH789.b1	GE297749
61941705	CCAH790.g1	GE297750
61941706	CCAH791.b1	GE297751
61941707	CCAH791.g1	GE297752
61941708	CCAH792.b1	GE297753
61941709	CCAH792.g1	GE297754
61941710	CCAH793.b1	GE297755
61941711	CCAH793.g1	GE297756
61941712	CCAH795.b1	GE297757
61941713	CCAH795.g1	GE297758
61941714	CCAH796.b1	GE297759
61941715	CCAH796.g1	GE297760
61941716	CCAH797.b1	GE297761
61941717	CCAH797.g1	GE297762
61941718	CCAH798.b1	GE297763
61941719	CCAH798.g1	GE297764
61941720	CCAH799.b1	GE297765
61941721	CCAH799.g1	GE297766
61941722	CCAH800.b1	GE297767
61941723	CCAH800.g1	GE297768
61941724	CCAH801.b1	GE297769
61941725	CCAH801.g1	GE297770
61941726	CCAH802.b1	GE297771
61941727	CCAH802.g1	GE297772
61941728	CCAH803.b1	GE297773
61941729	CCAH803.g1	GE297774

61941730	CCAH804.b1	GE297775
61941731	CCAH806.b1	GE297776
61941732	CCAH806.g1	GE297777
61941733	CCAH807.b1	GE297778
61941734	CCAH808.b1	GE297779
61941735	CCAH808.g1	GE297780
61941736	CCAH809.b1	GE297781
61941737	CCAH809.g1	GE297782
61941738	CCAH810.b1	GE297783
61941739	CCAH811.b1	GE297784
61941740	CCAH811.g1	GE297785
61941741	CCAH813.b1	GE297786
61941742	CCAH813.g1	GE297787
61941743	CCAH814.b1	GE297788
61941744	CCAH814.g1	GE297789
61941745	CCAH815.b1	GE297790
61941746	CCAH815.g1	GE297791
61941747	CCAH816.b1	GE297792
61941748	CCAH816.g1	GE297793
61941749	CCAH817.b1	GE297794
61941750	CCAH817.g1	GE297795
61941751	CCAH818.b1	GE297796
61941752	CCAH818.g1	GE297797
61941753	CCAH819.b1	GE297798
61941754	CCAH819.g1	GE297799
61941755	CCAH820.b1	GE297800
61941756	CCAH820.g1	GE297801
61941757	CCAH821.b1	GE297802
61941758	CCAH821.g1	GE297803
61941759	CCAH822.b1	GE297804
61941760	CCAH822.g1	GE297805
61941761	CCAH823.b1	GE297806
61941762	CCAH823.g1	GE297807
61941763	CCAH824.b1	GE297808
61941764	CCAH824.g1	GE297809
61941765	CCAH825.g1	GE297810
61941766	CCAH826.b1	GE297811
61941767	CCAH826.g1	GE297812
61941768	CCAH827.b1	GE297813
61941769	CCAH827.g1	GE297814
61941770	CCAH828.b1	GE297815
61941771	CCAH828.g1	GE297816
61941772	CCAH829.b1	GE297817
61941773	CCAH830.b1	GE297818
61941774	CCAH830.g1	GE297819
61941775	CCAH832.b1	GE297820
61941776	CCAH833.b1	GE297821
61941777	CCAH833.g1	GE297822
61941778	CCAH834.b1	GE297823
61941779	CCAH834.g1	GE297824
61941780	CCAH835.b1	GE297825
61941781	CCAH835.g1	GE297826
61941782	CCAH836.b1	GE297827
61941783	CCAH836.g1	GE297828
61941784	CCAH837.g1	GE297829
61941785	CCAH839.b1	GE297830
61941786	CCAH839.g1	GE297831
61941787	CCAH841.b1	GE297832
61941788	CCAH841.g1	GE297833
61941789	CCAH843.b1	GE297834
61941790	CCAH843.g1	GE297835
61941791	CCAH844.b1	GE297836
61941792	CCAH844.g1	GE297837

61941793	CCAH845.b1	GE297838
61941794	CCAH845.g1	GE297839
61941795	CCAH846.b1	GE297840
61941796	CCAH846.g1	GE297841
61941797	CCAH848.b1	GE297842
61941798	CCAH848.g1	GE297843
61941799	CCAH849.b1	GE297844
61941800	CCAH849.g1	GE297845
61941801	CCAH850.b1	GE297846
61941802	CCAH850.g1	GE297847
61941803	CCAH851.b1	GE297848
61941804	CCAH852.b1	GE297849
61941805	CCAH852.g1	GE297850
61941806	CCAH853.b1	GE297851
61941807	CCAH853.g1	GE297852
61941808	CCAH854.b1	GE297853
61941809	CCAH854.g1	GE297854
61941810	CCAH855.b1	GE297855
61941811	CCAH855.g1	GE297856
61941812	CCAH856.b1	GE297857
61941813	CCAH856.g1	GE297858
61941814	CCAH857.b1	GE297859
61941815	CCAH857.g1	GE297860
61941816	CCAH858.b1	GE297861
61941817	CCAH859.b1	GE297862
61941818	CCAH860.b1	GE297863
61941819	CCAH860.g1	GE297864
61941820	CCAH862.b1	GE297865
61941821	CCAH862.g1	GE297866
61941822	CCAH863.b1	GE297867
61941823	CCAH863.g1	GE297868
61941824	CCAH865.b1	GE297869
61941825	CCAH866.b1	GE297870
61941826	CCAH866.g1	GE297871
61941827	CCAH867.b1	GE297872
61941828	CCAH867.g1	GE297873
61941829	CCAH868.b1	GE297874
61941830	CCAH869.b1	GE297875
61941831	CCAH869.g1	GE297876
61941832	CCAH870.b1	GE297877
61941833	CCAH870.g1	GE297878
61941834	CCAH871.b1	GE297879
61941835	CCAH871.g1	GE297880
61941836	CCAH873.b1	GE297881
61941837	CCAH873.g1	GE297882
61941838	CCAH874.b1	GE297883
61941839	CCAH874.g1	GE297884
61941840	CCAH875.b1	GE297885
61941841	CCAH876.b1	GE297886
61941842	CCAH876.g1	GE297887
61941843	CCAH877.b1	GE297888
61941844	CCAH877.g1	GE297889
61941845	CCAH878.g1	GE297890
61941846	CCAH879.b1	GE297891
61941847	CCAH879.g1	GE297892
61941848	CCAH880.b1	GE297893
61941849	CCAH880.g1	GE297894
61941850	CCAH881.b1	GE297895
61941851	CCAH881.g1	GE297896
61941852	CCAH882.b1	GE297897
61941853	CCAH882.g1	GE297898
61941854	CCAH883.b1	GE297899
61941855	CCAH883.g1	GE297900

61941856	CCAH884.b1	GE297901
61941857	CCAH884.g1	GE297902
61941858	CCAH885.b1	GE297903
61941859	CCAH885.g1	GE297904
61941860	CCAH887.b1	GE297905
61941861	CCAH887.g1	GE297906
61941862	CCAH888.b1	GE297907
61941863	CCAH888.g1	GE297908
61941864	CCAH890.b1	GE297909
61941865	CCAH890.g1	GE297910
61941866	CCAH891.b1	GE297911
61941867	CCAH891.g1	GE297912
61941868	CCAH893.b1	GE297913
61941869	CCAH894.b1	GE297914
61941870	CCAH894.g1	GE297915
61941871	CCAH895.b1	GE297916
61941872	CCAH895.g1	GE297917
61941873	CCAH896.b1	GE297918
61941874	CCAH896.g1	GE297919
61941875	CCAH897.b1	GE297920
61941876	CCAH897.g1	GE297921
61941877	CCAH898.b1	GE297922
61941878	CCAH898.g1	GE297923
61941879	CCAH899.b1	GE297924
61941880	CCAH899.g1	GE297925
61941881	CCAH901.b1	GE297926
61941882	CCAH901.g1	GE297927
61941883	CCAH902.b1	GE297928
61941884	CCAH902.g1	GE297929
61941885	CCAH903.b1	GE297930
61941886	CCAH903.g1	GE297931
61941887	CCAH904.b1	GE297932
61941888	CCAH904.g1	GE297933
61941889	CCAH905.b1	GE297934
61941890	CCAH905.g1	GE297935
61941891	CCAH906.b1	GE297936
61941892	CCAH906.g1	GE297937
61941893	CCAH907.b1	GE297938
61941894	CCAH907.g1	GE297939
61941895	CCAH908.b1	GE297940
61941896	CCAH908.g1	GE297941
61941897	CCAH909.b1	GE297942
61941898	CCAH909.g1	GE297943
61941899	CCAH910.b1	GE297944
61941900	CCAH910.g1	GE297945
61941901	CCAH911.b1	GE297946
61941902	CCAH911.g1	GE297947
61941903	CCAH912.g1	GE297948
61941904	CCAH913.b1	GE297949
61941905	CCAH914.b1	GE297950
61941906	CCAH914.g1	GE297951
61941907	CCAH915.b1	GE297952
61941908	CCAH915.g1	GE297953
61941909	CCAH916.b1	GE297954
61941910	CCAH916.g1	GE297955
61941911	CCAH917.b1	GE297956
61941912	CCAH917.g1	GE297957
61941913	CCAH918.b1	GE297958
61941914	CCAH918.g1	GE297959
61941915	CCAH919.b1	GE297960
61941916	CCAH919.g1	GE297961
61941917	CCAH920.b1	GE297962
61941918	CCAH920.g1	GE297963

61941919	CCAH921.b1	GE297964
61941920	CCAH921.g1	GE297965
61941921	CCAH923.b1	GE297966
61941922	CCAH923.g1	GE297967
61941923	CCAH924.b1	GE297968
61941924	CCAH924.g1	GE297969
61941925	CCAH925.b1	GE297970
61941926	CCAH925.g1	GE297971
61941927	CCAH926.b1	GE297972
61941928	CCAH926.g1	GE297973
61941929	CCAH927.b1	GE297974
61941930	CCAH927.g1	GE297975
61941931	CCAH928.b1	GE297976
61941932	CCAH928.g1	GE297977
61941933	CCAH929.b1	GE297978
61941934	CCAH929.g1	GE297979
61941935	CCAH930.b1	GE297980
61941936	CCAH930.g1	GE297981
61941937	CCAH931.b1	GE297982
61941938	CCAH931.g1	GE297983
61941939	CCAH932.b1	GE297984
61941940	CCAH932.g1	GE297985
61941941	CCAH933.b1	GE297986
61941942	CCAH933.g1	GE297987
61941943	CCAH934.b1	GE297988
61941944	CCAH935.b1	GE297989
61941945	CCAH935.g1	GE297990
61941946	CCAH936.b1	GE297991
61941947	CCAH936.g1	GE297992
61941948	CCAH937.b1	GE297993
61941949	CCAH937.g1	GE297994
61941950	CCAH938.b1	GE297995
61941951	CCAH938.g1	GE297996
61941952	CCAH939.b1	GE297997
61941953	CCAH939.g1	GE297998
61941954	CCAH940.b1	GE297999
61941955	CCAH940.g1	GE298000
61941956	CCAH942.b1	GE298001
61941957	CCAH942.g1	GE298002
61941958	CCAH943.b1	GE298003
61941959	CCAH943.g1	GE298004
61941960	CCAH944.b1	GE298005
61941961	CCAH944.g1	GE298006
61941962	CCAH945.b1	GE298007
61941963	CCAH945.g1	GE298008
61941964	CCAH946.b1	GE298009
61941965	CCAH946.g1	GE298010
61941966	CCAH947.b1	GE298011
61941967	CCAH947.g1	GE298012
61941968	CCAH948.b1	GE298013
61941969	CCAH948.g1	GE298014
61941970	CCAH949.b1	GE298015
61941971	CCAH949.g1	GE298016
61941972	CCAH950.b1	GE298017
61941973	CCAH950.g1	GE298018
61941974	CCAH951.b1	GE298019
61941975	CCAH951.g1	GE298020
61941976	CCAH952.b1	GE298021
61941977	CCAH952.g1	GE298022
61941978	CCAH953.b1	GE298023
61941979	CCAH953.g1	GE298024
61941980	CCAH954.b1	GE298025
61941981	CCAH954.g1	GE298026

61941982	CCAH955.b1	GE298027
61941983	CCAH955.g1	GE298028
61941984	CCAH957.b1	GE298029
61941985	CCAH957.g1	GE298030
61941986	CCAH958.b1	GE298031
61941987	CCAH958.g1	GE298032
61941988	CCAH959.g1	GE298033
61941989	CCAH960.b1	GE298034
61941990	CCAH961.b1	GE298035
61941991	CCAH961.g1	GE298036
61941992	CCAH962.b1	GE298037
61941993	CCAH962.g1	GE298038
61941994	CCAH963.b1	GE298039
61941995	CCAH963.g1	GE298040
61941996	CCAH964.b1	GE298041
61941997	CCAH964.g1	GE298042
61941998	CCAH965.b1	GE298043
61941999	CCAH965.g1	GE298044
61942000	CCAH966.b1	GE298045
61942001	CCAH966.g1	GE298046
61942002	CCAH967.b1	GE298047
61942003	CCAH967.g1	GE298048
61942004	CCAH968.b1	GE298049
61942005	CCAH968.g1	GE298050
61942006	CCAH969.b1	GE298051
61942007	CCAH970.b1	GE298052
61942008	CCAH970.g1	GE298053
61942009	CCAH971.b1	GE298054
61942010	CCAH971.g1	GE298055
61942011	CCAH972.b1	GE298056
61942012	CCAH973.b1	GE298057
61942013	CCAH973.g1	GE298058
61942014	CCAH974.b1	GE298059
61942015	CCAH974.g1	GE298060
61942016	CCAH975.b1	GE298061
61942017	CCAH976.b1	GE298062
61942018	CCAH976.g1	GE298063
61942019	CCAH977.b1	GE298064
61942020	CCAH977.g1	GE298065
61942021	CCAH978.b1	GE298066
61942022	CCAH978.g1	GE298067
61942023	CCAH979.b1	GE298068
61942024	CCAH980.b1	GE298069
61942025	CCAH980.g1	GE298070
61942026	CCAH981.b1	GE298071
61942027	CCAH981.g1	GE298072
61942028	CCAH982.b1	GE298073
61942029	CCAH982.g1	GE298074
61942030	CCAH983.b1	GE298075
61942031	CCAH984.b1	GE298076
61942032	CCAH985.b1	GE298077
61942033	CCAH985.g1	GE298078
61942034	CCAH986.b1	GE298079
61942035	CCAH986.g1	GE298080
61942036	CCAH987.b1	GE298081
61942037	CCAH987.g1	GE298082
61942038	CCAH988.b1	GE298083
61942039	CCAH988.g1	GE298084
61942040	CCAH989.b1	GE298085
61942041	CCAH989.g1	GE298086
61942042	CCAH990.b1	GE298087
61942043	CCAH990.g1	GE298088
61942044	CCAH991.b1	GE298089

61942045	CCAH991.g1	GE298090
61942046	CCAH992.b1	GE298091
61942047	CCAH992.g1	GE298092
61942048	CCAH993.b1	GE298093
61942049	CCAH993.g1	GE298094
61942050	CCAH996.b1	GE298095
61942051	CCAH996.g1	GE298096
61942052	CCAH998.b1	GE298097
61942053	CCAH998.g1	GE298098
61942054	CCAH999.b1	GE298099
61942055	CCAH999.g1	GE298100
