# Supplementary material for: Transcriptomic response of the mycoparasitic fungus Trichoderma atroviride to the presence of a fungal prey
Source: BMC Genomics. 2009 Nov 30;10:567. doi: 10.1186/1471-2164-10-567 (PMC2794292; doi:10.1186/1471-2164-10-567)
Supplement: Additional file 9 — Total list of genes identified in this study. this table lists number of all ESTs under the four conditions (abbreviated as in Additional File 3) and their identification obtained during this study. [file 1471-2164-10-567-S9.PDF]

Additional File S9. Total list of genes identified in this study

A - RNA processing

Prot Id	KOG Id	MP	MG	LC	IC	KOG Description
12549	KOG2146			1	1	Splicing coactivator SRm160/300, subunit SRm160 (contains PWI domain)
126865	KOG0123		1			Polyadenylate-binding protein (RRM superfamily)
127075	KOG0338			1	1	ATP-dependent RNA helicase
127105	KOG0816			1		Protein involved in mRNA turnover
127138	KOG4209	2		1		Splicing factor RNPS1, SR protein superfamily
127354	KOG4008		1			rRNA processing protein RRP7
127484	KOG1869		2			Splicing coactivator SRm160/300, subunit SRm300
127659	KOG0796			8		Spliceosome subunit
127809	KOG2573	1		2		Ribosome biogenesis protein - Nop56p/Sik1p
127914	KOG0265	1	1	1		U5 snRNP-specific protein-like factor and related proteins
127967	KOG0265		1		1	U5 snRNP-specific protein-like factor and related proteins
128209	KOG3392		1			Exon-exon junction complex, Magoh component
128329	KOG2330		2			Splicing factor 3b, subunit 2
128630	KOG0331	1		2		ATP-dependent RNA helicase
128990	KOG3763	1		1		mRNA export factor TAP/MEX67
129080	KOG0335		2	1		ATP-dependent RNA helicase
129183	KOG2793		1			Putative N2,N2-dimethylguanosine tRNA methyltransferase
129286	KOG1689	1				mRNA cleavage factor I subunit
129458	KOG2868			1		Decapping enzyme complex component DCP1
129585	KOG2146	1				Splicing coactivator SRm160/300, subunit SRm160 (contains PWI domain)
130240	KOG1609				1	Protein involved in mRNA turnover and stability
130541	KOG0152			1		Spliceosomal protein FBP11/Splicing factor PRP40
130542	KOG0063	1		2		RNAse L inhibitor, ABC superfamily
130681	KOG4676	1		1		Splicing factor, arginine/serine-rich
131373	KOG2793				1	Putative N2,N2-dimethylguanosine tRNA methyltransferase
131648	KOG4205			1		RNA-binding protein musashi/mRNA cleavage and polyadenylation factor I complex, subunit HRP1
132043	KOG2162	1	2			Nonsense-mediated mRNA decay protein
132378	KOG2793		1			Putative N2,N2-dimethylguanosine tRNA methyltransferase
132423	KOG2047		1			mRNA splicing factor
132454	KOG1137				1	mRNA cleavage and polyadenylation factor II complex, BRR5 (CPSF subunit)
132594	KOG0988		1		1	RNA-directed RNA polymerase QDE-1 required for posttranscriptional gene silencing and RNA interference

132616	KOG0346	1				RNA helicase
132991	KOG1869		1		1	Splicing coactivator SRm160/300, subunit SRm300
133087	KOG0272		1	2		U4/U6 small nuclear ribonucleoprotein Prp4 (contains WD40 repeats)
133680	KOG2044		1			5'-3' exonuclease HKE1/RAT1
133719	KOG3980		1			RNA 3'-terminal phosphate cyclase
133720	KOG2809		2	1		Telomerase elongation inhibitor/RNA maturation protein PINX1
133736	KOG0334	1	2			RNA helicase
134053	KOG2808		1			U5 snRNP-associated RNA splicing factor
134178	KOG0127				1	Nucleolar protein fibrillarin NOP77 (RRM superfamily)
134823	KOG2441			1		mRNA splicing factor/probable chromatin binding snw family nuclear protein
134864	KOG0335	2				ATP-dependent RNA helicase
135379	KOG1898			2		Splicing factor 3b, subunit 3
135662	KOG3448	1	2	1		Predicted snRNP core protein
135674	KOG0120	2		2	1	Splicing factor U2AF, large subunit (RRM superfamily)
136115	KOG3702		1			Nuclear polyadenylated RNA binding protein
136954	KOG2777				1	tRNA-specific adenosine deaminase 1
137057	KOG1869	1				Splicing coactivator SRm160/300, subunit SRm300
137141	KOG1781				2	Small Nuclear ribonucleoprotein splicing factor
138350	KOG1869			1		Splicing coactivator SRm160/300, subunit SRm300
138808	KOG1869	1		2		Splicing coactivator SRm160/300, subunit SRm300
13883	KOG1975			1		mRNA cap methyltransferase
139050	KOG1869	1				Splicing coactivator SRm160/300, subunit SRm300
139066	KOG1258	1		1		mRNA processing protein
140927	KOG4150		2			Predicted ATP-dependent RNA helicase
141282	KOG1869		1			Splicing coactivator SRm160/300, subunit SRm300
141485	KOG3460		1	2		Small nuclear ribonucleoprotein (snRNP) LSM3
141573	KOG3068		1			mRNA splicing factor
141989	KOG1446		1			Histone H3 (Lys4) methyltransferase complex and RNA cleavage factor II complex, subunit SWD2
142257	KOG2793			2		Putative N2,N2-dimethylguanosine tRNA methyltransferase
14313	KOG1040	1		4		Polyadenylation factor I complex, subunit, Yth1 (CPSF subunit)
143150	KOG0332		1			ATP-dependent RNA helicase
143226	KOG4655		1			U3 small nucleolar ribonucleoprotein (snoRNP) component
143930	KOG3482		1			Small nuclear ribonucleoprotein (snRNP) SMF
143973	KOG0330			1		ATP-dependent RNA helicase
143987	KOG0670		1			U4/U6-associated splicing factor PRP4

144430	KOG3080	1	3			Nucleolar protein-like/EBNA1-binding protein
144770	KOG0123			1		Polyadenylate-binding protein (RRM superfamily)
145432	KOG0123	2				Polyadenylate-binding protein (RRM superfamily)
145463	KOG1070	2				rRNA processing protein Rrp5
146357	KOG0150		1	2		Spliceosomal protein FBP21
146568	KOG3167	1	3	1		Box H/ACA snoRNP component, involved in ribosomal RNA pseudouridinylation
146612	KOG4046			1		RNase MRP and P, subunit POP4/p29
146702	KOG1475	1	7	4		Ribosomal protein RPL1/RPL2/RL4L4
146867	KOG0342		1	1		ATP-dependent RNA helicase pitchoune
147667	KOG0113		1	2		U1 small nuclear ribonucleoprotein (RRM superfamily)
147695	KOG2572			1		Ribosome biogenesis protein - Nop58p/Nop5p
147846	KOG4676			1		Splicing factor, arginine/serine-rich
148154	KOG0213				4	Splicing factor 3b, subunit 1
148324	KOG0333				1	U5 snRNP-like RNA helicase subunit
148711	KOG3387	1	1			60S ribosomal protein 15.5kD/SNU13, NHP2/L7A family (includes ribonuclease P subunit p38), involved in splicing
148749	KOG0113	2	3	2		U1 small nuclear ribonucleoprotein (RRM superfamily)
149378	KOG1642			2		Ribonuclease, T2 family
149391	KOG0331				1	ATP-dependent RNA helicase
149513	KOG2560			1		RNA splicing factor - Slu7p
149635	KOG3741				2	Poly(A) ribonuclease subunit
149822	KOG0947	1				Cytoplasmic exosomal RNA helicase SKI2, DEAD-box superfamily
149951	KOG1642	1	2			Ribonuclease, T2 family
150747	KOG1869			1		Splicing coactivator SRm160/300, subunit SRm300
151108	KOG0331		1			ATP-dependent RNA helicase
151396	KOG1902		1			Putative signal transduction protein involved in RNA splicing
152256	KOG0123			1		Polyadenylate-binding protein (RRM superfamily)
152405	KOG0123			1		Polyadenylate-binding protein (RRM superfamily)
152598	KOG1869			3		Splicing coactivator SRm160/300, subunit SRm300
153246	KOG2481	1				Protein required for normal rRNA processing
153634	KOG2375	1	2			Protein interacting with poly(A)-binding protein
153764	KOG0132		1			RNA polymerase II C-terminal domain-binding protein RA4, contains RPR and RRM domains
154108	KOG1869			1		Splicing coactivator SRm160/300, subunit SRm300
154676	KOG0701	1				dsRNA-specific nuclease Dicer and related ribonucleases
154688	KOG1869		1			Splicing coactivator SRm160/300, subunit SRm300
154866	KOG2051				1	Nonsense-mediated mRNA decay 2 protein

155659	KOG1869	1				Splicing coactivator SRm160/300, subunit SRm300
155698	KOG1869		1			Splicing coactivator SRm160/300, subunit SRm300
155791	KOG0647		1	1		mRNA export protein (contains WD40 repeats)
156253	KOG2217	2				U4/U6.U5 snRNP associated protein
156315	KOG1049	1				Polyadenylation factor I complex, subunit FIP1
156831	KOG1869	1				Splicing coactivator SRm160/300, subunit SRm300
156902	KOG2669			1		Regulator of nuclear mRNA
157352	KOG3801	1				Uncharacterized conserved protein BCN92
157370	KOG1040			1		Polyadenylation factor I complex, subunit, Yth1 (CPSF subunit)
157468	KOG0121			1	1	Nuclear cap-binding protein complex, subunit CBP20 (RRM superfamily)
157738	KOG1122	1				tRNA and rRNA cytosine-C5-methylase (nucleolar protein NOL1/NOP2)
157851	KOG3909		1			Queuine-tRNA ribosyltransferase
157949	KOG1098		1			Putative SAM-dependent rRNA methyltransferase SPB1
157986	KOG1795	2				U5 snRNP spliceosome subunit
158065	KOG1774		1			Small nuclear ribonucleoprotein E
159009	KOG1994			1		Predicted RNA binding protein, contains G-patch and Zn-finger domains
159312	KOG1869	1		1		Splicing coactivator SRm160/300, subunit SRm300
159967	KOG0106		1			Alternative splicing factor SRp55/B52/SRp75 (RRM superfamily)
160536	KOG2044		1			5'-3' exonuclease HKE1/RAT1
17548	KOG1040	1		2		Polyadenylation factor I complex, subunit, Yth1 (CPSF subunit)
20128	KOG1609				1	Protein involved in mRNA turnover and stability
2757	KOG1902		1			Putative signal transduction protein involved in RNA splicing
31448	KOG1895	1				mRNA cleavage and polyadenylation factor II complex, subunit PTA1
31464	KOG4209	2		1		Splicing factor RNPS1, SR protein superfamily
31927	KOG0106	1		1		Alternative splicing factor SRp55/B52/SRp75 (RRM superfamily)
32074	KOG0131				1	Splicing factor 3b, subunit 4
36105	KOG1908	4	1			Ribonuclease inhibitor type leucine-rich repeat proteins
37400	KOG3026		2	1		Splicing factor SPF30
40399	KOG2749		1			mRNA cleavage and polyadenylation factor IA/II complex, subunit CLP1
40784	KOG0123			1		Polyadenylate-binding protein (RRM superfamily)
41068	KOG2749		1			mRNA cleavage and polyadenylation factor IA/II complex, subunit CLP1
42274	KOG3408	1	3			U1-like Zn-finger-containing protein, probabl erole in RNA processing/splicing
46199	KOG0701	1				dsRNA-specific nuclease Dicer and related ribonucleases
46839	KOG1784	1	1			Small Nuclear ribonucleoprotein splicing factor
47313	KOG2190			1		PolyC-binding proteins alphaCP-1 and related KH domain proteins

47539	KOG1869				4	Splicing coactivator SRm160/300, subunit SRm300
50235	KOG1596		4	2		Fibrillarin and related nucleolar RNA-binding proteins
51942	KOG3801	1				Uncharacterized conserved protein BCN92
52531	KOG1104				1	Nuclear cap-binding complex, subunit NCBP1/CBP80
53649	KOG0948			1		Nuclear exosomal RNA helicase MTR4, DEAD-box superfamily
6062	KOG2793		1			Putative N2,N2-dimethylguanosine tRNA methyltransferase
6746	KOG1869			1		Splicing coactivator SRm160/300, subunit SRm300
6799	KOG1817				1	Ribonuclease
80131	KOG1869				2	Splicing coactivator SRm160/300, subunit SRm300
81992	KOG2190			1		PolyC-binding proteins alphaCP-1 and related KH domain proteins
8969	KOG1869			3		Splicing coactivator SRm160/300, subunit SRm300
92022	KOG1869	1				Splicing coactivator SRm160/300, subunit SRm300
93410	KOG1104				1	Nuclear cap-binding complex, subunit NCBP1/CBP80
9445	KOG1784	1	1			Small Nuclear ribonucleoprotein splicing factor
94571	KOG1642	1	1	1		Ribonuclease, T2 family

B-chromatin synthesis

10208	KOG2684	1				Sirtuin 5 and related class III sirtuins (SIR2 family)
127007	KOG0264		2	3		Nucleosome remodeling factor, subunit CAF1/NURF55/MSI1
127198	KOG0386				2	Chromatin remodeling complex SWI/SNF, component SWI2 and related ATPases (DNA/RNA helicase superfamily)
127742	KOG3265	1				Histone chaperone involved in gene silencing
127883	KOG1472		3			Histone acetyltransferase SAGA/ADA, catalytic subunit PCAF/GCN5 and related proteins
128343	KOG1342				2	Histone deacetylase complex, catalytic component RPD3
128425	KOG4191			1		Histone acetyltransferases PCAF/SAGA/ADA, subunit TADA3L/NGG1
128442	KOG1911				1	Heterochromatin-associated protein HP1 and related CHROMO domain proteins
128482	KOG1009	1			2	Chromatin assembly complex 1 subunit B/CAC2 (contains WD40 repeats)
129632	KOG3844	1				Predicted component of NuA3 histone acetyltransferase complex
129788	KOG2266		1			Chromatin-associated protein Dek and related proteins, contains SAP DNA binding domain
130464	KOG2510	1				SWI-SNF chromatin-remodeling complex protein
130931	KOG0933		1	1	1	Structural maintenance of chromosome protein 2 (chromosome condensation complex Condensin, subunit E)
131774	KOG1279			1		Chromatin remodeling factor subunit and related transcription factors
131894	KOG1082				2	Histone H3 (Lys9) methyltransferase SUV39H1/Clr4, required for transcriptional silencing
131899	KOG1156		1	1		N-terminal acetyltransferase
131938	KOG1245				4	Chromatin remodeling complex WSTF-ISWI, large subunit (contains heterochromatin localization, PHD and BROMO domains)
132017	KOG1343	1	1			Histone deacetylase complex, catalytic component HDA1

132023	KOG2682			1		NAD-dependent histone deacetylases and class I sirtuins (SIR2 family)
132105	KOG3118			1		Disrupter of silencing SAS10
132155	KOG1827		1			Chromatin remodeling complex RSC, subunit RSC1/Polybromo and related proteins
132326	KOG0526	1				Nucleosome-binding factor SPN, POB3 subunit
132482	KOG2510			1		SWI-SNF chromatin-remodeling complex protein
132615	KOG2510				1	SWI-SNF chromatin-remodeling complex protein
133200	KOG2510	2				SWI-SNF chromatin-remodeling complex protein
133830	KOG2748			1		Uncharacterized conserved protein, contains chromo domain
134177	KOG2025			1		Chromosome condensation complex Condensin, subunit G
134823	KOG2441			1		mRNA splicing factor/probable chromatin binding snw family nuclear protein
135777	KOG2328	1				Chromosome condensation complex Condensin, subunit H
135826	KOG2130			1		Phosphatidylserine-specific receptor PtdSerR, contains JmjC domain
136602	KOG0355			1		DNA topoisomerase type II
138053	KOG1911		1	1		Heterochromatin-associated protein HP1 and related CHROMO domain proteins
138648	KOG2570	1			2	SWI/SNF transcription activation complex subunit
140985	KOG2747			1	1	Histone acetyltransferase (MYST family)
141989	KOG1446		1			Histone H3 (Lys4) methyltransferase complex and RNA cleavage factor II complex, subunit SWD2
142778	KOG1911		1	3		Heterochromatin-associated protein HP1 and related CHROMO domain proteins
144299	KOG2510	1	1	1		SWI-SNF chromatin-remodeling complex protein
145823	KOG1757		1	2		Histone 2A
146642	KOG1507	7	2	2		Nucleosome assembly protein NAP-1
146680	KOG3467	20	19	12		Histone H4
146854	KOG2747			1		Histone acetyltransferase (MYST family)
148488	KOG3001		2			Dosage compensation regulatory complex/histone acetyltransferase complex, subunit MSL-3/MRG15/EAF3,
149131	KOG1020	1				Sister chromatid cohesion protein SCC2/Nipped-B
150041	KOG1744	7	8	7		Histone H2B
150043	KOG1756	3	8	9		Histone 2A
150320	KOG4012	1	1	2		Histone H1
150943	KOG1911	1	2			Heterochromatin-associated protein HP1 and related CHROMO domain proteins
150985	KOG0979			3		Structural maintenance of chromosome protein SMC5/Spr18, SMC superfamily
151349	KOG4563	1				Cell cycle-regulated histone H1-binding protein
151499	KOG4012		1	1		Histone H1
151740	KOG2510	1		3		SWI-SNF chromatin-remodeling complex protein
152566	KOG2683				1	Sirtuin 4 and related class II sirtuins (SIR2 family)
152710	KOG1911			1		Heterochromatin-associated protein HP1 and related CHROMO domain proteins

153461	KOG1279		1			Chromatin remodeling factor subunit and related transcription factors
155130	KOG1649	1	1			SWI-SNF chromatin remodeling complex, Snf5 subunit
155553	KOG4204				1	Histone deacetylase complex, SIN3 component
156760	KOG2535				1	RNA polymerase II elongator complex, subunit ELP3/histone acetyltransferase
157021	KOG1973				1	Chromatin remodeling protein, contains PHD Zn-finger
158250	KOG1745	13	8	6		Histones H3 and H4
158311	KOG2084	1				Predicted histone tail methylase containing SET domain
19944	KOG4012	1		3		Histone H1
23	KOG0890		1			Protein kinase of the PI-3 kinase family involved in mitotic growth, DNA repair and meiotic recombination
26909	KOG1911	1	1			Heterochromatin-associated protein HP1 and related CHROMO domain proteins
29109	KOG0889	1				Histone acetyltransferase SAGA, TRRAP/TRA1 component, PI-3 kinase superfamily
36261	KOG2747		1			Histone acetyltransferase (MYST family)
37345	KOG2747				1	Histone acetyltransferase (MYST family)
51870	KOG2510				1	SWI-SNF chromatin-remodeling complex protein
54191	KOG2084	2		1		Predicted histone tail methylase containing SET domain
6871	KOG2682			2		NAD-dependent histone deacetylases and class I sirtuins (SIR2 family)
730	KOG1472	1				Histone acetyltransferase SAGA/ADA, catalytic subunit PCAF/GCN5 and related proteins
83530	KOG1245		1			Chromatin remodeling complex WSTF-ISWI, large subunit (contains heterochromatin localization, PHD and BROMO domains)
84081	KOG1245			1		Chromatin remodeling complex WSTF-ISWI, large subunit (contains heterochromatin localization, PHD and BROMO domains)
85610	KOG1472	1				Histone acetyltransferase SAGA/ADA, catalytic subunit PCAF/GCN5 and related proteins
**C-energy metabolism**						
127154	KOG0557			2		Dihydrolipoamide acetyltransferase
127178	KOG0134				1	NADH:flavin oxidoreductase/12-oxophytodienoate reductase
127345	KOG1254	4	1	4	3	ATP-citrate lyase
128831	KOG2614		3	2		Kynurenine 3-monooxygenase and related flavoprotein monooxygenases
130529	KOG0369	4		1	1	Pyruvate carboxylase
130625	KOG1254		5			ATP-citrate lyase
131412	KOG0754	18	7	17	1	Mitochondrial oxodicarboxylate carrier protein
132631	KOG1575	1	4			Voltage-gated shaker-like K+ channel, subunit beta/KCNAB
133076	KOG3855	6	1	1		Monooxygenase involved in coenzyme Q (ubiquinone) biosynthesis
133842	KOG3049	1	2	3		Succinate dehydrogenase, Fe-S protein subunit
133927	KOG1350	3	1	2		F0F1-type ATP synthase, beta subunit
135138	KOG1758	2	2	3		Mitochondrial F1F0-ATP synthase, subunit delta/ATP16
135641	KOG3976	3	7	4		Mitochondrial F1F0-ATP synthase, subunit b/ATP4

135957	KOG0749	20	12	30		Mitochondrial ADP/ATP carrier proteins
137037	KOG3469	1	2	5		Cytochrome c oxidase, subunit VIa/COX13
137358	KOG3378	4			5	Globins and related hemoproteins
137543	KOG2865	7	2	5		NADH:ubiquinone oxidoreductase, NDUFA9/39kDa subunit
137869	KOG3025	9	2	2		Mitochondrial F1F0-ATP synthase, subunit c/ATP9/proteolipid
141239	KOG2617	3	5			Citrate synthase
141439	KOG3352		4	1		Cytochrome c oxidase, subunit Vb/COX4
142217	KOG1626		3	1		Inorganic pyrophosphatase/Nucleosome remodeling factor, subunit NURF38
142715	KOG1626	1	3	3		Inorganic pyrophosphatase/Nucleosome remodeling factor, subunit NURF38
143042	KOG2404	4	1			Fumarate reductase, flavoprotein subunit
143500	KOG3855	8	21	26	1	Monooxygenase involved in coenzyme Q (ubiquinone) biosynthesis
144531	KOG1353	2	3			F0F1-type ATP synthase, alpha subunit
146022	KOG1671	4	1	5		Ubiquinol cytochrome c reductase, subunit RIP1
146329	KOG0232	2	2	3		Vacuolar H+-ATPase V0 sector, subunits c/c'
146350	KOG0535	2	3	1		Sulfite oxidase, molybdopterin-binding component
146445	KOG3389	2	3	1		NADH:ubiquinone oxidoreductase, NDUFS4/18 kDa subunit
146458	KOG0767	2	3	6		Mitochondrial phosphate carrier protein
146585	KOG2614		5	5		Kynurenine 3-monooxygenase and related flavoprotein monooxygenases
146687	KOG3366	1	3	6		Mitochondrial F1F0-ATP synthase, subunit d/ATP7
146690	KOG4075	3		1	1	Cytochrome c oxidase, subunit IV/COX5b
147737	KOG1260		2	4		Isocitrate lyase
148721	KOG3857		2	3		Alcohol dehydrogenase, class IV
150014	KOG0453	7	4	2		Aconitase/homoaconitase (aconitase superfamily)
150897	KOG0537	4	4	2		Cytochrome b5
151212	KOG3453	6	6	5		Cytochrome c
152040	KOG1494	4	1	2		NAD-dependent malate dehydrogenase
153771	KOG0756	3	7	7		Mitochondrial tricarboxylate/dicarboxylate carrier proteins
157160	KOG0558				3	Dihydrolipoamide transacylase (alpha-keto acid dehydrogenase E2 subunit)
159401	KOG1748	2	1	2	3	Acyl carrier protein/NADH-ubiquinone oxidoreductase, NDUFAB1/SDAP subunit
159564	KOG2450	4	1	7		Aldehyde dehydrogenase
32889	KOG2658	3	1	2		NADH:ubiquinone oxidoreductase, NDUFV1/51kDa subunit
34158	KOG1563	3		1		Mitochondrial protein Surfeit 1/SURF1/SHY1, required for expression of cytochrome oxidase
34335	KOG2658	3	1	2		NADH:ubiquinone oxidoreductase, NDUFV1/51kDa subunit
35034	KOG1563	3		1		Mitochondrial protein Surfeit 1/SURF1/SHY1, required for expression of cytochrome oxidase
41478	KOG1748	2	1	1	3	Acyl carrier protein/NADH-ubiquinone oxidoreductase, NDUFAB1/SDAP subunit

43962	KOG3954	2	6	3		Electron transfer flavoprotein, alpha subunit
51995	KOG4075	3		1	1	Cytochrome c oxidase, subunit IV/COX5b
77737	KOG0069		3	2		Glyoxylate/hydroxypyruvate reductase (D-isomer-specific 2-hydroxy acid dehydrogenase superfamily)
87417	KOG1261	2	1	1	3	Malate synthase

D-cell cycle

126880	KOG1173	1				Anaphase-promoting complex (APC), Cdc16 subunit
127006	KOG0600		1			Cdc2-related protein kinase
127117	KOG0822		1		1	Protein kinase inhibitor
127363	KOG0082		1	1		G-protein alpha subunit (small G protein superfamily)
127560	KOG2874			2		rRNA processing protein
127771	KOG3022		3	1		Predicted ATPase, nucleotide-binding
127851	KOG2043		2	1		Signaling protein SWIFT and related BRCT domain proteins
128409	KOG3300		2			NADH:ubiquinone oxidoreductase, B16.6 subunit/cell death-regulatory protein
128499	KOG1938		1		1	Protein with predicted involvement in meiosis (GSG1)
129011	KOG1213		2			Sister chromatid cohesion complex Cohesin, subunit RAD21/SCC1
129500	KOG4259				1	Putative nucleic acid-binding protein Hcc-1/proliferation associated cytokine-inducible protein, contains SAP domain
129527	KOG0305	1			1	Anaphase promoting complex, Cdc20, Cdh1, and Ama1 subunits
130162	KOG3999				1	Checkpoint 9-1-1 complex, HUS1 component
130711	KOG0995	1	1	2		Centromere-associated protein HEC1
130811	KOG2166	1	3	1		Cullins
130931	KOG0933		1	1	1	Structural maintenance of chromosome protein 2 (chromosome condensation complex Condensin, subunit E)
131628	KOG4151	1				Myosin assembly protein/sexual cycle protein and related proteins
132408	KOG0668			1		Casein kinase II, alpha subunit
133008	KOG1036			1		Mitotic spindle checkpoint protein BUB3, WD repeat superfamily
133139	KOG0580		2	1		Serine/threonine protein kinase
133438	KOG2035	1				Replication factor C, subunit RFC3
133720	KOG2809		2	2		Telomerase elongation inhibitor/RNA maturation protein PINX1
133919	KOG2043				1	Signaling protein SWIFT and related BRCT domain proteins
133990	KOG2739	1				Leucine-rich acidic nuclear protein
134177	KOG2025			1		Chromosome condensation complex Condensin, subunit G
134854	KOG2045	1		1		5'-3' exonuclease XRN1/KEM1/SEP1 involved in DNA strand exchange and mRNA turnover
135080	KOG3000	1		2		Microtubule-binding protein involved in cell cycle control
135139	KOG0672		1			Halotolerance protein HAL3 (contains flavoprotein domain)
135777	KOG2328	1				Chromosome condensation complex Condensin, subunit H

135988	KOG1547		2	4		Septin CDC10 and related P-loop GTPases
136266	KOG2867	1				Phosphotyrosyl phosphatase activator
136814	KOG1852				1	Cell cycle-associated protein
137979	KOG2218	1				ER to golgi transport protein/RAD50-interacting protein 1
138111	KOG0028				1	Ca2+-binding protein (centrin/caltractin), EF-Hand superfamily protein
138502	KOG4115	1		1		Dynein-associated protein Roadblock
140885	KOG4124			1		Putative transcriptional repressor regulating G2/M transition
141173	KOG0373			1		Serine/threonine specific protein phosphatase involved in cell cycle control, PP2A-related
141591	KOG2277			1		S-M checkpoint control protein CID1 and related nucleotidyltransferases
142437	KOG0672			1		Halotolerance protein HAL3 (contains flavoprotein domain)
145571	KOG3694	1				Protein required for meiosis
146394	KOG0653	4			4	Cyclin B and related kinase-activating proteins
146642	KOG1507	7	2	2		Nucleosome assembly protein NAP-1
146779	KOG3022				1	Predicted ATPase, nucleotide-binding
147075	KOG2163		1	1		Centromere/kinetochore protein zw10 involved in mitotic chromosome segregation
148232	KOG1746		1			Defender against cell death protein/oligosaccharyltransferase, epsilon subunit
148272	KOG0615		1	3		Serine/threonine protein kinase Chk2 and related proteins
148654	KOG0653		2			Cyclin B and related kinase-activating proteins
148848	KOG2655	1		1		Septin family protein (P-loop GTPase)
149006	KOG2043			2		Signaling protein SWIFT and related BRCT domain proteins
149131	KOG1020	1				Sister chromatid cohesion protein SCC2/Nipped-B
149153	KOG2513	1				Protein required for meiotic chromosome segregation
149298	KOG2151			3		Predicted transcriptional regulator
149483	KOG1547				1	Septin CDC10 and related P-loop GTPases
149670	KOG0590		2			Checkpoint kinase and related serine/threonine protein kinases
149814	KOG3004		2			Meiotic chromosome segregation protein
150123	KOG1546		1	1		Metacaspase involved in regulation of apoptosis
150985	KOG0979			3		Structural maintenance of chromosome protein SMC5/Spr18, SMC superfamily
151349	KOG4563	1				Cell cycle-regulated histone H1-binding protein
152249	KOG2398	2		3		Predicted proline-serine-threonine phosphatase-interacting protein (PSTPIP)
152365	KOG1546			2		Metacaspase involved in regulation of apoptosis
152419	KOG2655		4	3		Septin family protein (P-loop GTPase)
152582	KOG0892			1		Protein kinase ATM/Tel1, involved in telomere length regulation and DNA repair
152596	KOG0082	1		3	1	G-protein alpha subunit (small G protein superfamily)
152940	KOG2688			1		Transcription-associated recombination protein - Thp1p

153576	KOG4466				1	Component of histone deacetylase complex (breast carcinoma metastasis suppressor 1 protein in human)
154549	KOG2739		2			Leucine-rich acidic nuclear protein
154987	KOG2101		1		1	Intermediate filament-like protein, sorting nexins, and related proteins containing PX (PhoX) domain(s)
155131	KOG3772		1			M-phase inducer phosphatase
155476	KOG0590	2	1			Checkpoint kinase and related serine/threonine protein kinases
157081	KOG2867	1				Phosphotyrosyl phosphatase activator
157824	KOG2011			1		Sister chromatid cohesion complex Cohesin, subunit STAG/IRR1/SCC3
157855	KOG2655		1	1		Septin family protein (P-loop GTPase)
157930	KOG2398	1		2	1	Predicted proline-serine-threonine phosphatase-interacting protein (PSTPIP)
159187	KOG3772		1			M-phase inducer phosphatase
160022	KOG0440	1	2			Cell cycle-associated protein Mob1-1
160509	KOG0018		1			Structural maintenance of chromosome protein 1 (sister chromatid cohesion complex Cohesin, subunit SMC1)
16449	KOG4842		2			Protein involved in sister chromatid separation and/or segregation
16898	KOG3022		2	1	1	Predicted ATPase, nucleotide-binding
1739	KOG2043			1		Signaling protein SWIFT and related BRCT domain proteins
18585	KOG4613	1		2		Predicted component of DNA replication checkpoint response mechanism (S-M checkpoint)
20453	KOG2101	2	1			Intermediate filament-like protein, sorting nexins, and related proteins containing PX (PhoX) domain(s)
23	KOG0890			1		Protein kinase of the PI-3 kinase family involved in mitotic growth, DNA repair and meiotic recombination
2877	KOG2655			1		Septin family protein (P-loop GTPase)
29109	KOG0889	1				Histone acetyltransferase SAGA, TRRAP/TRA1 component, PI-3 kinase superfamily
31526	KOG2186	2			1	Cell growth-regulating nucleolar protein
32558	KOG0653		1	2		Cyclin B and related kinase-activating proteins
35122	KOG2151			2		Predicted transcriptional regulator
35164	KOG3431	1		2		Apoptosis-related protein/predicted DNA-binding protein
39908	KOG1546			3		Metacaspase involved in regulation of apoptosis
40680	KOG3772		1			M-phase inducer phosphatase
42219	KOG1509	2	1	2		Predicted nucleic acid-binding protein ASMTL
46679	KOG4139			1		Protein kinase essential for the initiation of DNA replication
47535	KOG0580		2			Serine/threonine protein kinase
47921	KOG3484			1		Cyclin-dependent protein kinase CDC28, regulatory subunit CKS1, and related proteins
52451	KOG1126				1	DNA-binding cell division cycle control protein
567	KOG2011			1		Sister chromatid cohesion complex Cohesin, subunit STAG/IRR1/SCC3
78278	KOG2186	2			1	Cell growth-regulating nucleolar protein
80526	KOG1509	2	1	2		Predicted nucleic acid-binding protein ASMTL
81746	KOG4139			1		Protein kinase essential for the initiation of DNA replication

82040	KOG3194			1	2	Checkpoint 9-1-1 complex, RAD1 component
86222	KOG3431	2		1		Apoptosis-related protein/predicted DNA-binding protein
88749	KOG2655		3	3		Septin family protein (P-loop GTPase)
94504	KOG0005		2			Ubiquitin-like protein

E-amino acid metabolism

127322	KOG1282	3	5	1		Serine carboxypeptidases (lysosomal cathepsin A)
127351	KOG3023	1				Glutamate-cysteine ligase regulatory subunit
127425	KOG2467		1			Glycine/serine hydroxymethyltransferase
127449	KOG0454	3		1		3-isopropylmalate dehydratase (aconitase superfamily)
127498	KOG1412	1			1	Aspartate aminotransferase/Glutamic oxaloacetic transaminase AAT2/GOT1
127748	KOG2467	1				Glycine/serine hydroxymethyltransferase
127954	KOG1286	1		1		Amino acid transporters
128242	KOG1593				3	Asparaginase
128316	KOG1402	4		3		Ornithine aminotransferase
128466	KOG2697		1		5	Histidinol dehydrogenase
128700	KOG0683	1				Glutamine synthetase
128719	KOG2275	3	2			Aminoacylase ACY1 and related metalloexopeptidases
128844	KOG1383			2		Glutamate decarboxylase/sphingosine phosphate lyase
129085	KOG1154	1	1		2	Gamma-glutamyl kinase
129202	KOG2436	1	1	1	1	Acetylglutamate kinase/acetylglutamate synthase
129397	KOG4165	3		1		Gamma-glutamyl phosphate reductase
129473	KOG0062	1		1		ATPase component of ABC transporters with duplicated ATPase domains/Translation elongation factor EF-3b
129821	KOG0456				2	Aspartate kinase
129885	KOG4354	2	1			N-acetyl-gamma-glutamyl-phosphate reductase
129918	KOG0692		1			Pentafunctional AROM protein
129976	KOG2413			1		Xaa-Pro aminopeptidase
129984	KOG1316	3	2			Argininosuccinate lyase
130100	KOG0453	1				Aconitase/homoaconitase (aconitase superfamily)
130126	KOG0622			3		Ornithine decarboxylase
130160	KOG1304		1			Amino acid transporters
130441	KOG3055				2	Phosphoribosylformimino-5-aminoimidazole carboxamide ribonucleotide (ProFAR) isomerase
131191	KOG2367	1	1			Alpha-isopropylmalate synthase/homocitrate synthase
131592	KOG2831	1				ATP phosphoribosyltransferase
131608	KOG1395			2		Tryptophan synthase beta chain

131799	KOG4750			1		Serine O-acetyltransferase
131971	KOG1282	1	1			Serine carboxypeptidases (lysosomal cathepsin A)
132065	KOG1939			1		Oxoprolinase
132536	KOG0053	1			1	Cystathionine beta-lyases/cystathionine gamma-synthases
132566	KOG1046	1	3	1		Puromycin-sensitive aminopeptidase and related aminopeptidases
132886	KOG1359			1		Glycine C-acetyltransferase/2-amino-3-ketobutyrate-CoA ligase
133430	KOG1223	3	1	1	1	Isochorismate synthase
133486	KOG1251	1				Serine racemase
133982	KOG0062		1			ATPase component of ABC transporters with duplicated ATPase domains/Translation elongation factor EF-3b
134194	KOG0805		2			Carbon-nitrogen hydrolase
134949	KOG1252	1	1			Cystathionine beta-synthase and related enzymes
135249	KOG2276	1	2			Metalloexopeptidases
135389	KOG3124		1			Pyrroline-5-carboxylate reductase
135477	KOG1289			1		Amino acid transporters
136004	KOG2263	3	2	2		Methionine synthase II (cobalamin-independent)
137018	KOG0806	1		1		Carbon-nitrogen hydrolase
137475	KOG1289		1			Amino acid transporters
137525	KOG0053	2				Cystathionine beta-lyases/cystathionine gamma-synthases
137935	KOG0629	1				Glutamate decarboxylase and related proteins
138144	KOG2965	1	1	1		Arginase
138933	KOG1401	1				Acetylornithine aminotransferase
138983	KOG1430	1				C-3 sterol dehydrogenase/3-beta-hydroxysteroid dehydrogenase and related dehydrogenases
139124	KOG0571	4	2	2		Asparagine synthase (glutamine-hydrolyzing)
139588	KOG2276			1		Metalloexopeptidases
139966	KOG1286	1		1		Amino acid transporters
140022	KOG2367	4		3		Alpha-isopropylmalate synthase/homocitrate synthase
140096	KOG1405	2		1		4-aminobutyrate aminotransferase
140394	KOG1441			1		Glucose-6-phosphate/phosphate and phosphoenolpyruvate/phosphate antiporter
140635	KOG0172	7	4			Lysine-ketoglutarate reductase/saccharopine dehydrogenase
141213	KOG0399	2				Glutamate synthase
141385	KOG2250		1	2		Glutamate/leucine/phenylalanine/valine dehydrogenases
142194	KOG1401			1		Acetylornithine aminotransferase
142486	KOG0806				2	Carbon-nitrogen hydrolase
142705	KOG1709			1		Guanidinoacetate methyltransferase and related proteins
142728	KOG2449	1	1	2		Methylmalonate semialdehyde dehydrogenase

143079	KOG0053	3				Cystathionine beta-lyases/cystathionine gamma-synthases
143855	KOG3216	1	1			Diamine acetyltransferase
144060	KOG1185	1	1			Thiamine pyrophosphate-requiring enzyme
144243	KOG2448	4				Dihydroxy-acid dehydratase
144897	KOG0692				1	Pentafunctional AROM protein
144900	KOG1237	1				H+/oligopeptide symporter
144950	KOG2348	2				Urea transporter
146352	KOG1237	2	1	2		H+/oligopeptide symporter
150014	KOG0453	8	4	2		Aconitase/homoaconitase (aconitase superfamily)
150078	KOG1184	16	1	1		Thiamine pyrophosphate-requiring enzyme
150731	KOG0138	2				Glutaryl-CoA dehydrogenase
150819	KOG0258			1		Alanine aminotransferase
151021	KOG4777	3		2		Aspartate-semialdehyde dehydrogenase
151384	KOG2770	3				Aminomethyl transferase
151523	KOG1430	1		3		C-3 sterol dehydrogenase/3-beta-hydroxysteroid dehydrogenase and related dehydrogenases
151712	KOG1047	1		1		Bifunctional leukotriene A4 hydrolase/aminopeptidase LTA4H
151723	KOG0628	1			1	Aromatic-L-amino-acid/L-histidine decarboxylase
151726	KOG0453	1				Aconitase/homoaconitase (aconitase superfamily)
151876	KOG2448			2		Dihydroxy-acid dehydratase
152520	KOG0062				2	ATPase component of ABC transporters with duplicated ATPase domains/Translation elongation factor EF-3b
152602	KOG4201	7		1		Anthranilate synthase component II
152716	KOG1441		1		1	Glucose-6-phosphate/phosphate and phosphoenolpyruvate/phosphate antiporter
152857	KOG1250	1		1		Threonine/serine dehydratases
152967	KOG2964	1	1			Arginase family protein
153042	KOG1289	1				Amino acid transporters
153054	KOG2352		3			Predicted spermine/spermidine synthase
153339	KOG1481				1	Cysteine synthase
153492	KOG1448	2	2	1		Ribose-phosphate pyrophosphokinase
154274	KOG2275	1				Aminoacylase ACY1 and related metalloexopeptidases
154439	KOG1046	1		2		Puromycin-sensitive aminopeptidase and related aminopeptidases
154470	KOG3124	1				Pyrroline-5-carboxylate reductase
154609	KOG1305				1	Amino acid transporter protein
154685	KOG0257	2				Kynurenine aminotransferase, glutamine transaminase K
154783	KOG0805		1			Carbon-nitrogen hydrolase
154860	KOG0053		1			Cystathionine beta-lyases/cystathionine gamma-synthases

154983	KOG0455		1			Homoserine dehydrogenase
155042	KOG2352				2	Predicted spermine/spermidine synthase
155433	KOG3627	12	1	29		Trypsin
155976	KOG0975	1				Branched chain aminotransferase BCAT1, pyridoxal phosphate enzymes type IV superfamily
156144	KOG0257		1	2		Kynurenine aminotransferase, glutamine transaminase K
156340	KOG0062	1	1	5		ATPase component of ABC transporters with duplicated ATPase domains/Translation elongation factor EF-3b
156406	KOG1287				1	Amino acid transporters
156629	KOG2410		1			Gamma-glutamyltransferase
156938	KOG0068		1		1	D-3-phosphoglycerate dehydrogenase, D-isomer-specific 2-hydroxy acid dehydrogenase superfamily
157245	KOG1438	1	1			Anthranilate phosphoribosyltransferase
157299	KOG3007			1		Mu-crystallin
157321	KOG0975	4	1			Branched chain aminotransferase BCAT1, pyridoxal phosphate enzymes type IV superfamily
157364	KOG2575		1			Glucosyltransferase - Alg6p
157505	KOG0238		1	2		3-Methylcrotonyl-CoA carboxylase, biotin-containing subunit/Propionyl-CoA carboxylase, alpha chain/Acetyl-CoA carboxylase, biotin carboxylase subunit
157675	KOG1289				3	Amino acid transporters
157793	KOG1250	1				Threonine/serine dehydratases
158028	KOG2414		1			Putative Xaa-Pro aminopeptidase
158094	KOG2380			2		Prephenate dehydrogenase (NADP+)
158818	KOG1286	2				Amino acid transporters
159247	KOG0189		1	1		Phosphoadenosine phosphosulfate reductase
159605	KOG0257	3	1			Kynurenine aminotransferase, glutamine transaminase K
160861	KOG1383	2		1	1	Glutamate decarboxylase/sphingosine phosphate lyase
160893	KOG1706		4	2		Argininosuccinate synthase
160938	KOG2551	1	1	4		Phospholipase/carboxyhydrolase
186	KOG1304		1			Amino acid transporters
33403	KOG0785		2			Isocitrate dehydrogenase, alpha subunit
3348	KOG1305				1	Amino acid transporter protein
33542	KOG4064		1	1		Cysteine dioxygenase CDO1
35395	KOG0786	3		1		3-isopropylmalate dehydrogenase
36504	KOG1286	1				Amino acid transporters
37287	KOG0629	1				Glutamate decarboxylase and related proteins
42213	KOG2616	6	1	1		Pyridoxalphosphate-dependent enzyme/predicted threonine synthase
42391	KOG2964	1	1			Arginase family protein
43145	KOG2250			2	1	Glutamate/leucine/phenylalanine/valine dehydrogenases
46652	KOG1401			1		Acetylornithine aminotransferase

49139	KOG0257		1	2		Kynurenine aminotransferase, glutamine transaminase K
51792	KOG1289		1			Amino acid transporters
52018	KOG0238			2		3-Methylcrotonyl-CoA carboxylase, biotin-containing subunit/Propionyl-CoA carboxylase, alpha chain/Acetyl-CoA carboxylase, biotin carboxylase subunit
52073	KOG4128		1			Bleomycin hydrolases and aminopeptidases of cysteine protease family
52078	KOG1591	1				Prolyl 4-hydroxylase alpha subunit
584	KOG1591	1				Prolyl 4-hydroxylase alpha subunit
6067	KOG1593				4	Asparaginase
77838	KOG1185			1		Thiamine pyrophosphate-requiring enzyme
78616	KOG0785		2			Isocitrate dehydrogenase, alpha subunit
80293	KOG0189		1	1		Phosphoadenosine phosphosulfate reductase
81185	KOG0784			1		Isocitrate dehydrogenase, gamma subunit
81358	KOG1304			1		Amino acid transporters
83295	KOG4128		1			Bleomycin hydrolases and aminopeptidases of cysteine protease family
87638	KOG1411	3	2	3		Aspartate aminotransferase/Glutamic oxaloacetic transaminase AAT1/GOT2
90784	KOG1237	3				H+/oligopeptide symporter
92316	KOG1251			1	1	Serine racemase
93017	KOG1289		1			Amino acid transporters
94783	KOG0257	5			1	Kynurenine aminotransferase, glutamine transaminase K

F-nucleotide metabolism

10736	KOG1096		1			Adenosine monophosphate deaminase
127817	KOG3985	3	1	1		Methylthioadenosine phosphorylase MTAP
128261	KOG2387			1		CTP synthase (UTP-ammonia lyase)
129539	KOG3347		1	1		Predicted nucleotide kinase/nuclear protein involved oxidative stress response
130158	KOG1385		1	4		Nucleoside phosphatase
130348	KOG0572		1			Glutamine phosphoribosylpyrophosphate amidotransferase
131410	KOG2056		1			Equilibrative nucleoside transporter protein
133051	KOG2835	1				Phosphoribosylamidoimidazole-succinocarboxamide synthase
133088	KOG2938	3				Predicted inosine-uridine preferring nucleoside hydrolase
133589	KOG3968		1			Atrazine chlorohydrolase/guanine deaminase
133737	KOG2555				2	AICAR transformylase/IMP cyclohydrolase/methylglyoxal synthase
134218	KOG1097	1				Adenine deaminase/adenosine deaminase
135574	KOG0833	1	3	1		Cytidine deaminase
135790	KOG3079			1		Uridylate kinase/adenylate kinase
136419	KOG1018			1		Cytosine deaminase FCY1 and related enzymes

136872	KOG1377	1	2			Uridine 5'- monophosphate synthase/orotate phosphoribosyltransferase
138304	KOG2550	1			2	IMP dehydrogenase/GMP reductase
139428	KOG2466	2	3	2		Uridine permease/thiamine transporter/allantoin transport
139677	KOG2584	1				Dihydroorotase and related enzymes
140864	KOG2550	1	1	2		IMP dehydrogenase/GMP reductase
141607	KOG4419			1		5' nucleotidase
142164	KOG2700	1	1			Adenylosuccinate lyase
142207	KOG1907			1		Phosphoribosylformylglycinamidine synthase
143209	KOG3127		2	1		Deoxycytidylate deaminase
143521	KOG1622				1	GMP synthase
146420	KOG1712			2		Adenine phosphoribosyl transferases
146507	KOG1448	1	2	1		Ribose-phosphate pyrophosphokinase
148414	KOG4145		2	1		Allantoicase
148478	KOG0707	1				Guanylate kinase
150658	KOG3981				1	Deoxyribose-phosphate aldolase
150786	KOG2584	2	1	2	4	Dihydroorotase and related enzymes
151985	KOG4419			1	1	5' nucleotidase
152077	KOG1097	2				Adenine deaminase/adenosine deaminase
152298	KOG3179		1			Predicted glutamine synthetase
152788	KOG2056			1		Equilibrative nucleoside transporter protein
153492	KOG1448	3	2	1		Ribose-phosphate pyrophosphokinase
154438	KOG1096	1	1			Adenosine monophosphate deaminase
154508	KOG1567			1		Ribonucleotide reductase, beta subunit
155643	KOG3078	1				Adenylate kinase
155747	KOG2902	1	2			Dihydroorotase
156719	KOG4419			1		5' nucleotidase
156989	KOG3078		2			Adenylate kinase
157130	KOG2938	1				Predicted inosine-uridine preferring nucleoside hydrolase
158745	KOG0888	12	25	8		Nucleoside diphosphate kinase
160302	KOG1377	1				Uridine 5'- monophosphate synthase/orotate phosphoribosyltransferase
160881	KOG1355	2		1	1	Adenylosuccinate synthase
160937	KOG1528			1		Salt-sensitive 3'-phosphoadenosine-5'-phosphatase HAL2/SAL1
21041	KOG1096	1	2			Adenosine monophosphate deaminase
22709	KOG2056		1			Equilibrative nucleoside transporter protein
3312	KOG1097	2				Adenine deaminase/adenosine deaminase

4662	KOG1355	1				Adenylosuccinate synthase
47950	KOG2902	1	2			Dihydroorotase
48767	KOG3127		2	1		Deoxycytidylate deaminase
77709	KOG0888	7	21	6		Nucleoside diphosphate kinase
79558	KOG1096		1			Adenosine monophosphate deaminase
83005	KOG3370	2	1			dUTPase
94524	KOG3379	2	1			Diadenosine polyphosphate hydrolase and related proteins of the histidine triad (HIT) family
**G-carbohydrate metabolism**						
10358	KOG1192				1	UDP-glucuronosyl and UDP-glucosyl transferase
127020	KOG3742	1			1	Glycogen synthase
127370	KOG0523	1				Transketolase
127540	KOG2504	1				Monocarboxylate transporter
128381	KOG2323	2	2			Pyruvate kinase
128410	KOG1192			1		UDP-glucuronosyl and UDP-glucosyl transferase
128710	KOG1192			1		UDP-glucuronosyl and UDP-glucosyl transferase
129072	KOG3147				2	6-phosphogluconolactonase - like protein
129518	KOG1950		11	18	5	Glycosyl transferase, family 8 - glycogenin
129529	KOG4626			1		O-linked N-acetylglucosamine transferase OGT
129633	KOG4157	1		1		beta-1,6-N-acetylglucosaminyltransferase, contains WSC domain
129753	KOG0234	1	1			Fructose-6-phosphate 2-kinase/fructose-2,6-biphosphatase
129790	KOG2178	1				Predicted sugar kinase
130287	KOG4157			1		beta-1,6-N-acetylglucosaminyltransferase, contains WSC domain
130331	KOG1643	9	6	1		Triosephosphate isomerase
130934	KOG2855	1				Ribokinase
131335	KOG1604		1			Predicted mutarotase
131461	KOG4157		1	1		beta-1,6-N-acetylglucosaminyltransferase, contains WSC domain
131462	KOG2533		1	1		Permease of the major facilitator superfamily
131492	KOG1369			1		Hexokinase
131548	KOG4157				1	beta-1,6-N-acetylglucosaminyltransferase, contains WSC domain
131943	KOG2234			1		Predicted UDP-galactose transporter
131999	KOG4157	1				beta-1,6-N-acetylglucosaminyltransferase, contains WSC domain
132157	KOG1879			1		UDP-glucose:glycoprotein glucosyltransferase
132215	KOG1192	1			1	UDP-glucuronosyl and UDP-glucosyl transferase
132257	KOG2772	1				Transaldolase

132261	KOG2741			1		Dimeric dihydrodiol dehydrogenase
132481	KOG4157	1				beta-1,6-N-acetylglucosaminyltransferase, contains WSC domain
132739	KOG1203	1				Predicted dehydrogenase
132969	KOG4157			1		beta-1,6-N-acetylglucosaminyltransferase, contains WSC domain
133123	KOG4157			1		beta-1,6-N-acetylglucosaminyltransferase, contains WSC domain
133882	KOG2772	6	3	6		Transaldolase
133908	KOG4157			1		beta-1,6-N-acetylglucosaminyltransferase, contains WSC domain
134257	KOG1442		2			GDP-fucose transporter
134536	KOG0449	1	1			Succinate dehydrogenase, cytochrome b subunit
135714	KOG4157		8	2		beta-1,6-N-acetylglucosaminyltransferase, contains WSC domain
135904	KOG4748			2		Subunit of Golgi mannosyltransferase complex
136112	KOG0563	2	2			Glucose-6-phosphate 1-dehydrogenase
136507	KOG2099	2	11	10		Glycogen phosphorylase
136537	KOG4157			3		beta-1,6-N-acetylglucosaminyltransferase, contains WSC domain
137148	KOG0235	1				Phosphoglycerate mutase
137467	KOG4157		1			beta-1,6-N-acetylglucosaminyltransferase, contains WSC domain
137875	KOG1050		1			Trehalose-6-phosphate synthase component TPS1 and related subunits
138654	KOG1583	1	1			UDP-N-acetylglucosamine transporter
138825	KOG2537		1			Phosphoglucomutase/phosphomannomutase
138874	KOG4157		2			beta-1,6-N-acetylglucosaminyltransferase, contains WSC domain
139512	KOG4153	4	5	5		Fructose 1,6-bisphosphate aldolase
13963	KOG2504	1				Monocarboxylate transporter
140018	KOG2440			1		Pyrophosphate-dependent phosphofructo-1-kinase
140394	KOG1441			1		Glucose-6-phosphate/phosphate and phosphoenolpyruvate/phosphate antiporter
140425	KOG1594		1			Uncharacterized enzymes related to aldose 1-epimerase
140629	KOG1050			1		Trehalose-6-phosphate synthase component TPS1 and related subunits
141071	KOG4157	1				beta-1,6-N-acetylglucosaminyltransferase, contains WSC domain
141221	KOG2517	2	1	1		Ribulose kinase and related carbohydrate kinases
141594	KOG4157		3	2		beta-1,6-N-acetylglucosaminyltransferase, contains WSC domain
141849	KOG4472			1		Glycolipid 2-alpha-mannosyltransferase (alpha-1,2-mannosyltransferase)
142203	KOG4157		1	2		beta-1,6-N-acetylglucosaminyltransferase, contains WSC domain
142709	KOG1369	1	4	1	3	Hexokinase
142728	KOG2449		1	2		Methylmalonate semialdehyde dehydrogenase
142939	KOG3075		1		1	Ribose 5-phosphate isomerase
143112	KOG1444		2	2		Nucleotide-sugar transporter VRG4/SQV-7

143663	KOG0657	9	8	9		Glyceraldehyde 3-phosphate dehydrogenase
143878	KOG0631			1		Galactokinase
144062	KOG2533	4	2	2		Permease of the major facilitator superfamily
144542	KOG2178	1			1	Predicted sugar kinase
145098	KOG0450	1	4	2		2-oxoglutarate dehydrogenase, E1 subunit
145242	KOG4157			2		beta-1,6-N-acetylglucosaminyltransferase, contains WSC domain
145866	KOG2533			1		Permease of the major facilitator superfamily
145889	KOG0235			1		Phosphoglycerate mutase
146041	KOG1050	2		3		Trehalose-6-phosphate synthase component TPS1 and related subunits
146182	KOG3221		3			Glycolipid transfer protein
146349	KOG1367	3	1	5		3-phosphoglycerate kinase
146527	KOG2631			1	4	Class II aldolase/adducin N-terminal domain protein
146790	KOG2533	1				Permease of the major facilitator superfamily
148392	KOG0637		1			Sucrose transporter and related proteins
148429	KOG4157		1	1		beta-1,6-N-acetylglucosaminyltransferase, contains WSC domain
148949	KOG0372			1		Serine/threonine specific protein phosphatase involved in glycogen accumulation, PP2A-related
148970	KOG3734	1				Predicted phosphoglycerate mutase
149074	KOG4157		1	1		beta-1,6-N-acetylglucosaminyltransferase, contains WSC domain
149595	KOG2533			1		Permease of the major facilitator superfamily
149728	KOG2504			1	1	Monocarboxylate transporter
149789	KOG2504			1		Monocarboxylate transporter
150261	KOG2533			1		Permease of the major facilitator superfamily
150540	KOG2843	2		1	1	Fumarylacetoacetase
150696	KOG4157	1		1		beta-1,6-N-acetylglucosaminyltransferase, contains WSC domain
150760	KOG2246			1		Galactosyltransferases
151065	KOG2504	1				Monocarboxylate transporter
151976	KOG2666			1		UDP-glucose/GDP-mannose dehydrogenase
152177	KOG2533		3	1		Permease of the major facilitator superfamily
152486	KOG2533		1			Permease of the major facilitator superfamily
152716	KOG1441		2			Glucose-6-phosphate/phosphate and phosphoenolpyruvate/phosphate antiporter
152800	KOG2533		1			Permease of the major facilitator superfamily
153642	KOG1604	1				Predicted mutarotase
153721	KOG0234		3			Fructose-6-phosphate 2-kinase/fructose-2,6-biphosphatase
153973	KOG4472			1		Glycolipid 2-alpha-mannosyltransferase (alpha-1,2-mannosyltransferase)
154204	KOG2504	2	1	2		Monocarboxylate transporter

154847	KOG2855	2	2		1	Ribokinase
154960	KOG4157	2				beta-1,6-N-acetylglucosaminyltransferase, contains WSC domain
155005	KOG1458	2				Fructose-1,6-bisphosphatase
155518	KOG3893				1	Mannosyltransferase
156021	KOG2757	1				Mannose-6-phosphate isomerase
156116	KOG2854		5	5	1	Possible pfkB family carbohydrate kinase
156403	KOG2533	1				Permease of the major facilitator superfamily
156683	KOG2533		1			Permease of the major facilitator superfamily
157035	KOG2670	12	8	3		Enolase
157077	KOG3974	1	1			Predicted sugar kinase
157161	KOG3354				2	Gluconate kinase
157252	KOG3111	1	1	1		D-ribulose-5-phosphate 3-epimerase
157268	KOG2944		1			Glyoxalase
157364	KOG2575		1			Glucosyltransferase - Alg6p
157577	KOG1369			1		Hexokinase
157696	KOG0658			3		Glycogen synthase kinase-3
158821	KOG4157				1	beta-1,6-N-acetylglucosaminyltransferase, contains WSC domain
159181	KOG4157	1		3		beta-1,6-N-acetylglucosaminyltransferase, contains WSC domain
159301	KOG0470	1	3	8		1,4-alpha-glucan branching enzyme/starch branching enzyme II
160457	KOG4157			2		beta-1,6-N-acetylglucosaminyltransferase, contains WSC domain
22466	KOG4157		1	1		beta-1,6-N-acetylglucosaminyltransferase, contains WSC domain
24597	KOG1583	1	1			UDP-N-acetylglucosamine transporter
28177	KOG4157			1		beta-1,6-N-acetylglucosaminyltransferase, contains WSC domain
29536	KOG4157			1		beta-1,6-N-acetylglucosaminyltransferase, contains WSC domain
30993	KOG4157			1		beta-1,6-N-acetylglucosaminyltransferase, contains WSC domain
3646	KOG1369			1		Hexokinase
38102	KOG1460			1		GDP-mannose pyrophosphorylase
3854	KOG4157				1	beta-1,6-N-acetylglucosaminyltransferase, contains WSC domain
38887	KOG2234			1		Predicted UDP-galactose transporter
39327	KOG0224	5	3	1		Aquaporin (major intrinsic protein family)
39628	KOG2517	3				Ribulose kinase and related carbohydrate kinases
40363	KOG0234	1	1			Fructose-6-phosphate 2-kinase/fructose-2,6-biphosphatase
44118	KOG2517	2	1			Ribulose kinase and related carbohydrate kinases
44963	KOG2504	2	1	2		Monocarboxylate transporter
48158	KOG2504		4			Monocarboxylate transporter

48521	KOG2504		4			Monocarboxylate transporter
50442	KOG2533	3	1	1		Permease of the major facilitator superfamily
52056	KOG1369			1		Hexokinase
54517	KOG0235	1		1		Phosphoglycerate mutase
78225	KOG3076	1				5'-phosphoribosylglycinamide formyltransferase
78534	KOG2653	5	8	2		6-phosphogluconate dehydrogenase
80164	KOG1460			1		GDP-mannose pyrophosphorylase
80344	KOG0234	1	1			Fructose-6-phosphate 2-kinase/fructose-2,6-biphosphatase
81764	KOG4157			1		beta-1,6-N-acetylglucosaminyltransferase, contains WSC domain
82369	KOG2504		1	1		Monocarboxylate transporter
87024	KOG4513	2	2	2	1	Phosphoglycerate mutase
8871	KOG4157			1		beta-1,6-N-acetylglucosaminyltransferase, contains WSC domain
89049	KOG1879			1		UDP-glucose:glycoprotein glucosyltransferase
91357	KOG2638	1	4	5		UDP-glucose pyrophosphorylase
94829	KOG2951			1	2	Inositol monophosphatase
**J-protein synthesis**						
126865	KOG0123		1			Polyadenylate-binding protein (RRM superfamily)
127065	KOG2553				2	Pseudouridylate synthase
127171	KOG1715			1		Mitochondrial/chloroplast ribosomal protein L12
127329	KOG0437			1	1	Leucyl-tRNA synthetase
127454	KOG0469	5	5	11		Elongation factor 2
127560	KOG2874			2		rRNA processing protein
127609	KOG1740			1		Predicted mitochondrial/chloroplast ribosomal protein S17
127809	KOG2573	1		2		Ribosome biogenesis protein - Nop56p/Sik1p
127962	KOG1612	1		1		Exosomal 3'-5' exoribonuclease complex, subunit Rrp42
128033	KOG2102		1			Exosomal 3'-5' exoribonuclease complex, subunit Rrp44/Dis3
128075	KOG1637	5	1			Threonyl-tRNA synthetase
128143	KOG3933				1	Mitochondrial ribosomal protein S28
128334	KOG3073		1	1		Protein required for 18S rRNA maturation and 40S ribosome biogenesis
128553	KOG4772			1	3	Predicted tRNA-splicing endonuclease subunit
128718	KOG1468			1		Predicted translation initiation factor related to eIF-2B alpha/beta/delta subunits (CIG2/IDI2)

128727	KOG1885	1	1	2		Lysyl-tRNA synthetase (class II)
128756	KOG2590			1		RNA-binding protein LARP/SRO9 and related La domain proteins
128808	KOG2767	1		2		Translation initiation factor 5 (eIF-5)
129043	KOG1754	4	6	8		40S ribosomal protein S15/S22
129086	KOG2298		1	1		Glycyl-tRNA synthetase and related class II tRNA synthetase
129179	KOG0857	6	15	9		60s ribosomal protein L10
129389	KOG2523			1		Predicted RNA-binding protein with PUA domain
129473	KOG0062	2	3	3		ATPase component of ABC transporters with duplicated ATPase domains/Translation elongation factor EF-3b
129775	KOG3506		3	2		40S ribosomal protein S29
129792	KOG1669				2	Predicted mRNA cap-binding protein related to eIF-4E
129811	KOG1627	3	7	2		Translation elongation factor EF-1 gamma
129919	KOG2311			2		NAD/FAD-utilizing protein possibly involved in translation
130028	KOG1211				1	Amidases
130153	KOG1035	1		1		eIF-2alpha kinase GCN2
130311	KOG2021	1				Nuclear mRNA export factor receptor LOS1/Exportin-t (importin beta superfamily)
130544	KOG3928		1	1		Mitochondrial ribosome small subunit component, mediator of apoptosis DAP3
130545	KOG1765		1			Regulator of ribosome synthesis
130567	KOG0327	8	4	3		Translation initiation factor 4F, helicase subunit (eIF-4A) and related helicases
130572	KOG0432	2	1	2		Valyl-tRNA synthetase
130719	KOG3070	1				Predicted RNA-binding protein containing PIN domain and invovled in translation or RNA processing
131030	KOG1211	1				Amidases
131227	KOG3262			1		H/ACA small nucleolar RNP component GAR1
131907	KOG0408	1				Mitochondrial/chloroplast ribosomal protein S11
131910	KOG0432			1		Valyl-tRNA synthetase
131929	KOG1211				2	Amidases
132179	KOG0901		1			60S ribosomal protein L14/L17/L23
132317	KOG1637	1				Threonyl-tRNA synthetase
132344	KOG0433				1	Isoleucyl-tRNA synthetase
132590	KOG0878	1	5	1		60S ribosomal protein L32
132872	KOG1148	1	3			Glutaminyl-tRNA synthetase
133202	KOG2411		1			Aspartyl-tRNA synthetase, mitochondrial
133676	KOG1749	7	11	14		40S ribosomal protein S23
133982	KOG0062		1			ATPase component of ABC transporters with duplicated ATPase domains/Translation elongation factor EF-3b
134058	KOG3184	3	4	2		60S ribosomal protein L7
134137	KOG3791		1			Predicted RNA-binding protein involved in translational regulation

134151	KOG0556	1			1	Aspartyl-tRNA synthetase
134444	KOG0328	3	3	2		Predicted ATP-dependent RNA helicase FAL1, involved in rRNA maturation, DEAD-box superfamily
134605	KOG0436		1			Methionyl-tRNA synthetase
134650	KOG2007	3	1	2		Cysteinyl-tRNA synthetase
134653	KOG2915	1		1		tRNA(1-methyladenosine) methyltransferase, subunit GCD14
135217	KOG2815	1	1	1		Mitochondrial/choloroplast ribosomal protein S15
135287	KOG0643	1		1		Translation initiation factor 3, subunit i (eIF-3i)/TGF-beta receptor-interacting protein (TRIP-1)
135459	KOG1698			2		Mitochondrial/chloroplast ribosomal protein L19
135897	KOG1936	1	2			Histidyl-tRNA synthetase
135953	KOG3291	6	7	8		Ribosomal protein S7
136132	KOG2479	1		1		Translation initiation factor 3, subunit d (eIF-3d)
136292	KOG1711	1	1			Mitochondrial/chloroplast ribosomal protein L22
136770	KOG0555		1	1		Asparaginyl-tRNA synthetase
136781	KOG0465		1			Mitochondrial elongation factor
137657	KOG1461	1		1	2	Translation initiation factor 2B, epsilon subunit (eIF-2Bepsilon/GCD6)
137745	KOG2317	2		1		Putative translation initiation inhibitor UK114/IBM1
138079	KOG0459			2		Polypeptide release factor 3
138429	KOG1041		1			Translation initiation factor 2C (eIF-2C) and related proteins
138651	KOG0434	1	1	1		Isoleucyl-tRNA synthetase
138759	KOG3401	3	9	6		60S ribosomal protein L26
138916	KOG3031		1	1		Protein required for biogenesis of the ribosomal 60S subunit
139257	KOG2975	1				Translation initiation factor 3, subunit f (eIF-3f)
139837	KOG0187	2	9	3		40S ribosomal protein S17
139949	KOG3419			1		Mitochondrial/chloroplast ribosomal protein S16
140053	KOG1614		1			Exosomal 3'-5' exoribonuclease complex, subunit Rrp45
141026	KOG1723		1			60s ribosomal protein L30 isolog
141125	KOG3283	2	1	1		40S ribosomal protein S8
142339	KOG1144	1		2		Translation initiation factor 5B (eIF-5B)
142346	KOG3271	10	6	6		Translation initiation factor 5A (eIF-5A)
142558	KOG2916	2	1			Translation initiation factor 2, alpha subunit (eIF-2alpha)
142570	KOG4089	1				Predicted mitochondrial ribosomal protein L23
142792	KOG3406	18	21	27		40S ribosomal protein S12
142846	KOG0402	5	8	1		60S ribosomal protein L37
142901	KOG2278				1	RNA:NAD 2'-phosphotransferase TPT1
143020	KOG0468		1			U5 snRNP-specific protein

143459	KOG3424	6	16	14	1	40S ribosomal protein S24
143482	KOG0556	3	1	3		Aspartyl-tRNA synthetase
143977	KOG2963			2		RNA-binding protein required for 60S ribosomal subunit biogenesis
144116	KOG3204	1		2		60S ribosomal protein L13a
144335	KOG3353	2	7	4		60S ribosomal protein L22
144419	KOG2509			1	1	Seryl-tRNA synthetase
144770	KOG0123			1		Polyadenylate-binding protein (RRM superfamily)
145000	KOG3421	1	2	2		60S ribosomal protein L14
145206	KOG0460	1	1	1		Mitochondrial translation elongation factor Tu
145290	KOG0435	1				Leucyl-tRNA synthetase
145432	KOG0123	2				Polyadenylate-binding protein (RRM superfamily)
145607	KOG1462	1	1			Translation initiation factor 2B, gamma subunit (eIF-2Bgamma/GCD1)
145861	KOG1212		1			Amidases
145887	KOG2144	1				Tyrosyl-tRNA synthetase, cytoplasmic
146028	KOG4163	1				Prolyl-tRNA synthetase
146064	KOG1569	1	1			50S ribosomal protein L1
146088	KOG3320	5	3	3	1	40S ribosomal protein S7
146098	KOG3445		1	2		Mitochondrial/chloroplast ribosomal protein 36a
146126	KOG0400	4	13	5		40S ribosomal protein S13
146154	KOG3452	3	4			60S ribosomal protein L36
146160	KOG0887	12	7	10		60S ribosomal protein L35A/L37
146169	KOG1714	6	2	7		60s ribosomal protein L18
146202	KOG0122		4	1		Translation initiation factor 3, subunit g (eIF-3g)
146204	KOG2613	2	1	1		NMD protein affecting ribosome stability and mRNA decay
146217	KOG0378	5	5	3	1	40S ribosomal protein S4
146234	KOG3257	31	24	27		Mitochondrial/chloroplast ribosomal protein L11
146279	KOG3166	4	2	9	1	60S ribosomal protein L7A
146284	KOG0004	26	23	13		Ubiquitin/40S ribosomal protein S27a fusion
146285	KOG1768	2	1	3		40s ribosomal protein S26
146312	KOG0875	10	12	9		60S ribosomal protein L5
146339	KOG3464	12	4	11		60S ribosomal protein L44
146381	KOG1613		2	1		Exosomal 3'-5' exoribonuclease complex, subunit Rrp43
146396	KOG0829	3	4	2		60S ribosomal protein L18A
146413	KOG2317		3	1		Putative translation initiation inhibitor UK114/IBM1
146448	KOG1678	10	6	4		60s ribosomal protein L15

146450	KOG1646	12	9	11		40S ribosomal protein S6
146486	KOG3185	1	1	1		Translation initiation factor 6 (eIF-6)
146551	KOG0009	8	12	6		Ubiquitin-like/40S ribosomal S30 protein fusion
146560	KOG2988	11	11	8		60S ribosomal protein L30
146576	KOG1762	19	22	22		60s acidic ribosomal protein P1
146596	KOG2309	11	11	5		60s ribosomal protein L2/L8
146639	KOG1628	12	21	10	1	40S ribosomal protein S3A
146653	KOG1069			2		Exosomal 3'-5' exoribonuclease complex, subunit Rrp46
146673	KOG3449	18	26	20		60S acidic ribosomal protein P2
146720	KOG3411	2	1	3		40S ribosomal protein S19
146727	KOG3203			3		Mitochondrial/chloroplast ribosomal protein L13
146742	KOG3412	14	5	10		60S ribosomal protein L28
146887	KOG1467	1	1			Translation initiation factor 2B, delta subunit (eIF-2Bdelta/GCD2)
146978	KOG1147	5	1			Glutamyl-tRNA synthetase
147043	KOG1384				2	tRNA delta(2)-isopentenylpyrophosphate transferase
147106	KOG2102		1			Exosomal 3'-5' exoribonuclease complex, subunit Rrp44/Dis3
147678	KOG1488			1		Translational repressor Pumilio/PUF3 and related RNA-binding proteins (Puf superfamily)
147695	KOG2572		1	1		Ribosome biogenesis protein - Nop58p/Nop5p
147699	KOG0830			1	1	40S ribosomal protein SA (P40)/Laminin receptor 1
148078	KOG0898	13	14	12	3	40S ribosomal protein S15
148127	KOG2154			2		Predicted nucleolar protein involved in ribosome biogenesis
148319	KOG0466	1		1		Translation initiation factor 2, gamma subunit (eIF-2gamma; GTPase)
148397	KOG3311	2				Ribosomal protein S18
148399	KOG2145		1	2		Cytoplasmic tryptophanyl-tRNA synthetase
148711	KOG3387	1	1			60S ribosomal protein 15.5kD/SNU13, NHP2/L7A family (includes ribonuclease P subunit p38), involved in splicing
148774	KOG1144		1			Translation initiation factor 5B (eIF-5B)
148795	KOG2050	1				Puf family RNA-binding protein
148863	KOG3301		5	2		Ribosomal protein S4
148873	KOG0556			1		Aspartyl-tRNA synthetase
14894	KOG1467	1	1			Translation initiation factor 2B, delta subunit (eIF-2Bdelta/GCD2)
149241	KOG0062	1				ATPase component of ABC transporters with duplicated ATPase domains/Translation elongation factor EF-3b
149439	KOG1722	4	7	12		60s ribosomal protein L24
149520	KOG1732	5	8	6		60S ribosomal protein L21
149533	KOG1770	2				Translation initiation factor 1 (eIF-1/SUI1)
149719	KOG3502	7	10	7		40S ribosomal protein S28

149856	KOG1212			1		Amidases
149873	KOG2784	2	1			Phenylalanyl-tRNA synthetase, beta subunit
150196	KOG0832			1		Mitochondrial/chloroplast ribosomal protein S2
150369	KOG2206			1		Exosome 3'-5' exoribonuclease complex, subunit PM/SCL-100 (Rrp6)
150386	KOG2758			1		Translation initiation factor 3, subunit e (eIF-3e)
150394	KOG0900	3	7	4		40S ribosomal protein S20
150496	KOG3295	10	9	10	1	60S Ribosomal protein L13
150849	KOG3291		1			Ribosomal protein S7
151388	KOG3255	18	18	9		60S ribosomal protein L9
151793	KOG2768	9	5	2		Translation initiation factor 2, beta subunit (eIF-2beta)
151975	KOG1211	1				Amidases
152256	KOG0123			1		Polyadenylate-binding protein (RRM superfamily)
152304	KOG1466			1		Translation initiation factor 2B, alpha subunit (eIF-2Balpha/GCN3)
152405	KOG0123			1		Polyadenylate-binding protein (RRM superfamily)
152409	KOG1211			1		Amidases
152501	KOG2539			1		Mitochondrial/chloroplast ribosome small subunit component
152520	KOG0062				2	ATPase component of ABC transporters with duplicated ATPase domains/Translation elongation factor EF-3b
152715	KOG2971		1			RNA-binding protein required for biogenesis of the ribosomal 60S subunit
153342	KOG0188	6	2			Alanyl-tRNA synthetase
153387	KOG1885		1	1		Lysyl-tRNA synthetase (class II)
153608	KOG2049				1	Translational repressor MPT5/PUF4 and related RNA-binding proteins (Puf superfamily)
154192	KOG1212	3	2	1		Amidases
154384	KOG1750			1		Mitochondrial/chloroplast ribosomal protein S12
154464	KOG2314	3		1		Translation initiation factor 3, subunit b (eIF-3b)
154923	KOG3769		1	1	1	Ribonuclease III domain proteins
155381	KOG4548	1	1		1	Mitochondrial ribosomal protein L17
155817	KOG1247		1		1	Methionyl-tRNA synthetase
155873	KOG3323	1				D-Tyr-tRNA (Tyr) deacylase
156103	KOG1570	5	4	7		60S ribosomal protein L10A
156107	KOG0688	2				Peptide chain release factor 1 (eRF1)
156281	KOG0746	13	6	6		60S ribosomal protein L3 and related proteins
156340	KOG0062	1	1	7		ATPase component of ABC transporters with duplicated ATPase domains/Translation elongation factor EF-3b
156634	KOG3492			1		Ribosome biogenesis protein NIP7
156699	KOG1211				2	Amidases
156948	KOG0886	3	7	7		40S ribosomal protein S2

157073	KOG0397	6	15	6		60S ribosomal protein L11
157151	KOG3436	4	3	9	3	60S ribosomal protein L35
157209	KOG3435			1		Mitochondrial/chloroplast ribosomal protein L54/L37
157341	KOG1560	2				Translation initiation factor 3, subunit h (eIF-3h)
157617	KOG3271	8	10	21		Translation initiation factor 5A (eIF-5A)
157697	KOG2529				2	Pseudouridine synthase
158032	KOG3677	1	3			RNA polymerase I-associated factor - PAF67
158106	KOG1149		1			Glutamyl-tRNA synthetase (mitochondrial)
158174	KOG1211			1		Amidases
158228	KOG3504	2	3	2		60S ribosomal protein L29
158703	KOG3499	2	5	5		60S ribosomal protein L38
159048	KOG1767	7	5	7		40S ribosomal protein S25
15905	KOG1612	1	1	1		Exosomal 3'-5' exoribonuclease complex, subunit Rrp42
159818	KOG3181		6	10		40S ribosomal protein S3
159925	KOG0003	19	15	12		Ubiquitin/60s ribosomal protein L40 fusion
160324	KOG3344	6	5	7		40s ribosomal protein s10
160841	KOG1790	4	7	5		60s ribosomal protein L34
160958	KOG2713	1	1			Mitochondrial tryptophanyl-tRNA synthetase
18817	KOG1750			1		Mitochondrial/chloroplast ribosomal protein S12
27968	KOG4639		1			RNase P/RNase MRP subunit POP5
2977	KOG4685		1			tRNA splicing endonuclease SEN2
30861	KOG3499	2	5	5		60S ribosomal protein L38
31487	KOG0438			1		Mitochondrial/chloroplast ribosomal protein L2
31515	KOG1767	5	5	7		40S ribosomal protein S25
32564	KOG3445		1	2		Mitochondrial/chloroplast ribosomal protein 36a
32958	KOG3301	8	13	9		Ribosomal protein S4
33818	KOG2198		1	2		tRNA cytosine-5-methylases and related enzymes of the NOL1/NOP2/sun superfamily
34076	KOG3403		7	4		Translation initiation factor 1A (eIF-1A)
35174	KOG2198	1		2		tRNA cytosine-5-methylases and related enzymes of the NOL1/NOP2/sun superfamily
35217	KOG3475	2	7	9		60S ribosomal protein L37
36999	KOG1461	1	1		2	Translation initiation factor 2B, epsilon subunit (eIF-2Bepsilon/GCD6)
39278	KOG1211				2	Amidases
40024	KOG1211			1		Amidases
40332	KOG0187	2	9	4		40S ribosomal protein S17
40784	KOG0123			1		Polyadenylate-binding protein (RRM superfamily)

41638	KOG3486	8	5	5		40S ribosomal protein S21
41715	KOG3073	1	1	1		Protein required for 18S rRNA maturation and 40S ribosome biogenesis
41980	KOG1211	1		1	1	Amidases
42058	KOG3486	9	6	5		40S ribosomal protein S21
42202	KOG1670		1			Translation initiation factor 4F, cap-binding subunit (eIF-4E) and related cap-binding proteins
42669	KOG1670		1			Translation initiation factor 4F, cap-binding subunit (eIF-4E) and related cap-binding proteins
47556	KOG0003	24	19	12		Ubiquitin/60s ribosomal protein L40 fusion
47649	KOG3254		1			Mitochondrial/chloroplast ribosomal protein L6
4853	KOG4772			1	3	Predicted tRNA-splicing endonuclease subunit
49376	KOG3344	7	6	7		40s ribosomal protein s10
51325	KOG2317	1		1		Putative translation initiation inhibitor UK114/IBM1
51448	KOG2038			1		CAATT-binding transcription factor/60S ribosomal subunit biogenesis protein
52576	KOG0398		1			Mitochondrial/chloroplast ribosomal protein L5/L7
52627	KOG0398		1			Mitochondrial/chloroplast ribosomal protein L5/L7
78525	KOG0438			1		Mitochondrial/chloroplast ribosomal protein L2
78759	KOG3301	10	22	12	1	Ribosomal protein S4
79355	KOG3505	1			1	Mitochondrial/chloroplast ribosomal protein L33-like
80258	KOG1779	3				40s ribosomal protein S27
81737	KOG4639		1	1		RNase P/RNase MRP subunit POP5
83539	KOG0407	4	7	5		40S ribosomal protein S14
84957	KOG2072	3	2	2		Translation initiation factor 3, subunit a (eIF-3a)
85616	KOG1242	13	15	15	1	Protein containing adaptin N-terminal region
89549	KOG1885	1		1		Lysyl-tRNA synthetase (class II)
91500	KOG2278				1	RNA:NAD 2'-phosphotransferase TPT1
92814	KOG1195	3	1			Arginyl-tRNA synthetase
92969	KOG2317	1		1		Putative translation initiation inhibitor UK114/IBM1
94456	KOG0815	4	3	9		60S acidic ribosomal protein P0
94749	KOG1728	11	13	14	2	40S ribosomal protein S11
94917	KOG0002	8	6	5		60s ribosomal protein L39
94986	KOG1694	6	3	4		60s ribosomal protein L6

K-transcription

10208	KOG2684	1				Sirtuin 5 and related class III sirtuins (SIR2 family)
11811	KOG1883	2				Cofactor required for Sp1 transcriptional activation, subunit 3
127198	KOG0386				2	Chromatin remodeling complex SWI/SNF, component SWI2 and related ATPases (DNA/RNA helicase superfamily)

127259	KOG1952				3	Transcription factor NF-X1, contains NFX-type Zn2+-binding and R3H domains
127707	KOG0384			1		Chromodomain-helicase DNA-binding protein
127742	KOG3265	1				Histone chaperone involved in gene silencing
127829	KOG0051		2	1		RNA polymerase I termination factor, Myb superfamily
127851	KOG2043		1	1		Signaling protein SWIFT and related BRCT domain proteins
127883	KOG1472		2	1		Histone acetyltransferase SAGA/ADA, catalytic subunit PCAF/GCN5 and related proteins
128478	KOG2652			1		RNA polymerase II transcription initiation factor TFIIA, large chain
129048	KOG3149		1			Transcription initiation factor IIF, auxiliary subunit
129458	KOG2868			1		Decapping enzyme complex component DCP1
129494	KOG1874	1		1		KEKE-like motif-containing transcription regulator (Rlr1)/suppressor of sin4
129638	KOG0260		1			RNA polymerase II, large subunit
129909	KOG2076			1		RNA polymerase III transcription factor TFIIIC
129996	KOG1034	1	1	1		Transcriptional repressor EED/ESC/FIE, required for transcriptional silencing, WD repeat superfamily
130415	KOG1883	2				Cofactor required for Sp1 transcriptional activation, subunit 3
130631	KOG3104	1				Mod5 protein sorting/negative effector of RNA Pol III synthesis
130798	KOG1878				1	Nuclear receptor coregulator SMRT/SMRTER, contains Myb-like domains
130835	KOG1001			1		Helicase-like transcription factor HLTF/DNA helicase RAD5, DEAD-box superfamily
130892	KOG1474			1		Transcription initiation factor TFIID, subunit BDF1 and related bromodomain proteins
130946	KOG1001	1				Helicase-like transcription factor HLTF/DNA helicase RAD5, DEAD-box superfamily
131081	KOG1015				1	Transcription regulator XNP/ATRX, DEAD-box superfamily
131555	KOG3227	1		1		Calcium-responsive transcription coactivator
131687	KOG2483				2	Upstream transcription factor 2/L-myc-2 protein
131894	KOG1082				2	Histone H3 (Lys9) methyltransferase SUV39H1/Clr4, required for transcriptional silencing
132023	KOG2682			1		NAD-dependent histone deacetylases and class I sirtuins (SIR2 family)
132082	KOG1874	2	1	3		KEKE-like motif-containing transcription regulator (Rlr1)/suppressor of sin4
132108	KOG3227				1	Calcium-responsive transcription coactivator
132121	KOG2814		1			Transcription coactivator complex, P50 component (LigT RNA ligase/phosphodiesterase family)
132155	KOG1827		1			Chromatin remodeling complex RSC, subunit RSC1/Polybromo and related proteins
132326	KOG0526	1				Nucleosome-binding factor SPN, POB3 subunit
132408	KOG0668			1		Casein kinase II, alpha subunit
132468	KOG0215	1				RNA polymerase III, second largest subunit
132491	KOG1474	1				Transcription initiation factor TFIID, subunit BDF1 and related bromodomain proteins
132650	KOG0216			1		RNA polymerase I, second largest subunit
132971	KOG0837	21	13	11		Transcriptional activator of the JUN family
133411	KOG2680			1		DNA helicase TIP49, TBP-interacting protein

133538	KOG1999				1	RNA polymerase II transcription elongation factor DSIF/SUPT5H/SPT5
133813	KOG1474		1			Transcription initiation factor TFIID, subunit BDF1 and related bromodomain proteins
133919	KOG2043				1	Signaling protein SWIFT and related BRCT domain proteins
133982	KOG2355		1			Predicted ABC-type transport, ATPase component/CCR4 associated factor
134490	KOG1037	2	1			NAD+ ADP-ribosyltransferase Parp, required for poly-ADP ribosylation of nuclear proteins
134552	KOG0849	1				Transcription factor PRD and related proteins, contain PAX and HOX domains
134974	KOG1474			1		Transcription initiation factor TFIID, subunit BDF1 and related bromodomain proteins
135292	KOG0385		1	1		Chromatin remodeling complex WSTF-ISWI, small subunit
135737	KOG3613				1	Dopey and related predicted leucine zipper transcription factors
136064	KOG1601	3		1	1	GATA-4/5/6 transcription factors
136988	KOG4005			1	4	Transcription factor XBP-1
137221	KOG1084		1			Transcription factor TCF20
137322	KOG1414	3	4	14		Transcriptional activator FOSB/c-Fos and related bZIP transcription factors
137669	KOG0627			1		Heat shock transcription factor
138030	KOG3227		1			Calcium-responsive transcription coactivator
138048	KOG2773			1		Apoptosis antagonizing transcription factor/protein transport protein
138648	KOG2570	1			2	SWI/SNF transcription activation complex subunit
138765	KOG1083		2			Putative transcription factor ASH1/LIN-59
138844	KOG1499		1	7		Protein arginine N-methyltransferase PRMT1 and related enzymes
138995	KOG3298	1		1		DNA-directed RNA polymerase subunit E'
139091	KOG2402		1			Paf1/RNA polymerase II complex, RTF1 component (involved in regulation of TATA box-binding protein)
139169	KOG1878				1	Nuclear receptor coregulator SMRT/SMRTER, contains Myb-like domains
140088	KOG4183			1		RNA polymerase I 49 kDa subunit
140885	KOG4124			1		Putative transcriptional repressor regulating G2/M transition
141098	KOG2587	1		2		RNA polymerase III (C) subunit
141284	KOG0709	1	1	2		CREB/ATF family transcription factor
141780	KOG1001			2		Helicase-like transcription factor HLTF/DNA helicase RAD5, DEAD-box superfamily
142107	KOG1474	1		1		Transcription initiation factor TFIID, subunit BDF1 and related bromodomain proteins
142113	KOG1878			1		Nuclear receptor coregulator SMRT/SMRTER, contains Myb-like domains
142409	KOG3463		1	1		Transcription initiation factor IIA, gamma subunit
142686	KOG1414	3		2		Transcriptional activator FOSB/c-Fos and related bZIP transcription factors
143600	KOG1414	1	1			Transcriptional activator FOSB/c-Fos and related bZIP transcription factors
143979	KOG4210	1	1			Nuclear localization sequence binding protein
144839	KOG1967			1		DNA repair/transcription protein Mms19
144980	KOG2039	1	1	1		Transcriptional coactivator p100

145671	KOG1657	1		1		CCAAT-binding factor, subunit C (HAP5)
145960	KOG2294				1	Transcription factor of the Forkhead/HNF3 family
146337	KOG4210		2			Nuclear localization sequence binding protein
146436	KOG1105	1			1	Transcription elongation factor TFIIS/Cofactor of enhancer-binding protein Sp1
146598	KOG3404	1				G10 protein/predicted nuclear transcription regulator
147923	KOG1123	1			1	RNA polymerase II transcription initiation/nucleotide excision repair factor TFIIH, 3'-5' helicase subunit SSL2
148488	KOG3001		2			Dosage compensation regulatory complex/histone acetyltransferase complex, subunit MSL-3/MRG15/EAF3, and related CHROMO domain-containing proteins
148632	KOG1318				2	Helix loop helix transcription factor EB
148668	KOG0260			2		RNA polymerase II, large subunit
148798	KOG2351	1	1	1		RNA polymerase II, fourth largest subunit
148897	KOG0773	2		1		Transcription factor MEIS1 and related HOX domain proteins
148919	KOG1904	1				Transcription coactivator
149006	KOG2043			2		Signaling protein SWIFT and related BRCT domain proteins
149291	KOG3902	1				Histone acetyltransferase PCAF/SAGA, subunit SUPT3H/SPT3
149298	KOG2151			3		Predicted transcriptional regulator
149657	KOG3227			1		Calcium-responsive transcription coactivator
150071	KOG2406	1				MADS box transcription factor
150837	KOG2588	1		1		Predicted DNA-binding protein
150965	KOG3804		2	1		Transcription factor NERF and related proteins, contain ETS domain
151043	KOG0214	1	1	1		RNA polymerase II, second largest subunit
151367	KOG2588		1			Predicted DNA-binding protein
151438	KOG1878			4	1	Nuclear receptor coregulator SMRT/SMRTER, contains Myb-like domains
151694	KOG3398	2	4	2		Transcription factor MBF1
152531	KOG1001	1				Helicase-like transcription factor HLTF/DNA helicase RAD5, DEAD-box superfamily
152566	KOG2683				1	Sirtuin 4 and related class II sirtuins (SIR2 family)
152692	KOG4274				3	Positive cofactor 2 (PC2), subunit of a multiprotein coactivator of RNA polymerase II
152830	KOG0939		1			E3 ubiquitin-protein ligase/Putative upstream regulatory element binding protein
153298	KOG2424		2			Protein involved in transcription start site selection
153351	KOG3497	2	1			DNA-directed RNA polymerase, subunit RPB10
153576	KOG4466				1	Component of histone deacetylase complex (breast carcinoma metastasis suppressor 1 protein in human)
153649	KOG0939	3				E3 ubiquitin-protein ligase/Putative upstream regulatory element binding protein
153671	KOG0627			2		Heat shock transcription factor
153717	KOG1522	1				RNA polymerase II, subunit POLR2C/RPB3
153764	KOG0132		1			RNA polymerase II C-terminal domain-binding protein RA4, contains RPR and RRM domains

154118	KOG1878				1	Nuclear receptor coregulator SMRT/SMRTER, contains Myb-like domains
154414	KOG1861	2	2	2		Leucine permease transcriptional regulator
154634	KOG1668	4	11	4		Elongation factor 1 beta/delta chain
154952	KOG2588	2		1	1	Predicted DNA-binding protein
155337	KOG2478		1			Putative RNA polymerase II regulator
155577	KOG4167			1		Predicted DNA-binding protein, contains SANT and ELM2 domains
155588	KOG0008			1		Transcription initiation factor TFIID, subunit TAF1
155880	KOG0260		1			RNA polymerase II, large subunit
155987	KOG0666			1		Cyclin C-dependent kinase CDK8
156175	KOG3901	1				Transcription initiation factor IID subunit
156425	KOG3803				1	Transcription factor containing C2HC type Zn finger
156760	KOG2535				1	RNA polymerase II elongator complex, subunit ELP3/histone acetyltransferase
157185	KOG1548			1		Transcription elongation factor TAT-SF1
157248	KOG1597		1	1		Transcription initiation factor TFIIB
157372	KOG3227			1		Calcium-responsive transcription coactivator
157550	KOG2239	2	4	1		Transcription factor containing NAC and TS-N domains
157864	KOG2261			1	1	Polycomb enhancer protein, EPC
158032	KOG3677	1	3			RNA polymerase I-associated factor - PAF67
158347	KOG0526	1				Nucleosome-binding factor SPN, POB3 subunit
159104	KOG3227	4	3	2		Calcium-responsive transcription coactivator
159404	KOG3119		1	3	1	Basic region leucine zipper transcription factor
159630	KOG3149	1	1	2	1	Transcription initiation factor IIF, auxiliary subunit
160651	KOG0773		1			Transcription factor MEIS1 and related HOX domain proteins
160687	KOG4086				1	Transcriptional regulator SOH1
1739	KOG2043			1		Signaling protein SWIFT and related BRCT domain proteins
2522	KOG2406	1				MADS box transcription factor
31569	KOG2473			1		RNA polymerase III transcription factor (TF)IIIC subunit
31816	KOG1952				2	Transcription factor NF-X1, contains NFX-type Zn2+-binding and R3H domains
32363	KOG4392		1			RNA polymerase, subunit L
33800	KOG1510	1				RNA polymerase II holoenzyme and mediator subcomplex, subunit SURB7/SRB7
33835	KOG2068	1			1	MOT2 transcription factor
34026	KOG1601				1	GATA-4/5/6 transcription factors
3468	KOG1474		1			Transcription initiation factor TFIID, subunit BDF1 and related bromodomain proteins
35122	KOG2151				2	Predicted transcriptional regulator
35526	KOG1831	1				Negative regulator of transcription

36009	KOG1001			2		Helicase-like transcription factor HLTF/DNA helicase RAD5, DEAD-box superfamily
3796	KOG1499			1		Protein arginine N-methyltransferase PRMT1 and related enzymes
38953	KOG0570	1			1	Transcriptional coactivator
39888	KOG1620	1				Inositol polyphosphate multikinase, component of the ARGR transcription regulatory complex
40052	KOG4274		1		2	Positive cofactor 2 (PC2), subunit of a multiprotein coactivator of RNA polymerase II
40869	KOG4183			1		RNA polymerase I 49 kDa subunit
42270	KOG1605	1		1	1	TFIIF-interacting CTD phosphatase, including NLI-interacting factor (involved in RNA polymerase II regulation)
43514	KOG3227				1	Calcium-responsive transcription coactivator
43863	KOG3836	1				HLH transcription factor EBF/Olf-1 and related DNA binding proteins
4486	KOG2478		1			Putative RNA polymerase II regulator
47194	KOG0837	15	7	8		Transcriptional activator of the JUN family
48533	KOG0387			1		Transcription-coupled repair protein CSB/RAD26 (contains SNF2 family DNA-dependent ATPase domain)
49393	KOG3438			1	1	DNA-directed RNA polymerase, subunit L
49507	KOG0147		1	2		Transcriptional coactivator CAPER (RRM superfamily)
51101	KOG1015			1		Transcription regulator XNP/ATRX, DEAD-box superfamily
51448	KOG2038			1		CAATT-binding transcription factor/60S ribosomal subunit biogenesis protein
53508	KOG3924		1			Putative protein methyltransferase involved in meiosis and transcriptional silencing (Dot1)
6281	KOG1474				1	Transcription initiation factor TFIID, subunit BDF1 and related bromodomain proteins
6871	KOG2682			2		NAD-dependent histone deacetylases and class I sirtuins (SIR2 family)
730	KOG1472	1				Histone acetyltransferase SAGA/ADA, catalytic subunit PCAF/GCN5 and related proteins
77798	KOG4392		1			RNA polymerase, subunit L
77865	KOG2691		2	2		RNA polymerase II subunit 9
77949	KOG3405	1		1		RNA polymerase subunit K
78735	KOG1510	1				RNA polymerase II holoenzyme and mediator subcomplex, subunit SURB7/SRB7
78807	KOG1601				1	GATA-4/5/6 transcription factors
81218	KOG1001			2		Helicase-like transcription factor HLTF/DNA helicase RAD5, DEAD-box superfamily
8162	KOG0869	2	2	1		CCAAT-binding factor, subunit A (HAP3)
83183	KOG3297		1			DNA-directed RNA polymerase subunit E'
8345	KOG0015	1	1			Regulator of arginine metabolism and related MADS box-containing transcription factors
83963	KOG1034	1	1	1		Transcriptional repressor EED/ESC/FIE, required for transcriptional silencing, WD repeat superfamily
85236	KOG0869	2	2	1		CCAAT-binding factor, subunit A (HAP3)
85610	KOG1472	1				Histone acetyltransferase SAGA/ADA, catalytic subunit PCAF/GCN5 and related proteins
85738	KOG1878	1		1		Nuclear receptor coregulator SMRT/SMRTER, contains Myb-like domains
8660	KOG4661		1	1		Hsp27-ERE-TATA-binding protein/Scaffold attachment factor (SAF-B)
89336	KOG0323			2		TFIIF-interacting CTD phosphatases, including NLI-interacting factor

91742	KOG0387			1		Transcription-coupled repair protein CSB/RAD26 (contains SNF2 family DNA-dependent ATPase domain)
9203	KOG3423	1				Transcription initiation factor TFIID, subunit TAF10 (also component of histone acetyltransferase SAGA)
92084	KOG0147		1	2		Transcriptional coactivator CAPER (RRM superfamily)

L-cellular repair

127244	KOG1361	1				Predicted hydrolase involved in interstrand cross-link repair
127490	KOG1433	1				DNA repair protein RAD51/RHP55
127655	KOG0219				1	Mismatch repair ATPase MSH2 (MutS family)
127704	KOG2519			2		5'-3' exonuclease
127851	KOG2043		2	1		Signaling protein SWIFT and related BRCT domain proteins
128482	KOG1009	1			2	Chromatin assembly complex 1 subunit B/CAC2 (contains WD40 repeats)
128652	KOG1929				1	Nucleotide excision repair factor NEF2, RAD4/CUT5 component
129013	KOG0478	1				DNA replication licensing factor, MCM4 component
129064	KOG0011		1			Nucleotide excision repair factor NEF2, RAD23 component
129339	KOG2095		1			DNA polymerase iota/DNA damage inducible protein
129348	KOG1968	1				Replication factor C, subunit RFC1 (large subunit)
129659	KOG3752			1		Ribonuclease H
129817	KOG0298	1				DEAD box-containing helicase-like transcription factor/DNA repair protein
130058	KOG2795		1			Catalytic subunit of the meiotic double strand break transesterase
130835	KOG1001			1		Helicase-like transcription factor HLTF/DNA helicase RAD5, DEAD-box superfamily
130946	KOG1001	1				Helicase-like transcription factor HLTF/DNA helicase RAD5, DEAD-box superfamily
130949	KOG0012	2		1		DNA damage inducible protein
131009	KOG0968			1		DNA polymerase zeta, catalytic subunit
131771	KOG4141			1		DNA repair and recombination protein RAD52/RAD22
132326	KOG0526	1				Nucleosome-binding factor SPN, POB3 subunit
133031	KOG0351				2	ATP-dependent DNA helicase
133438	KOG2035	1				Replication factor C, subunit RFC3
133680	KOG2044		1			5'-3' exonuclease HKE1/RAT1
133840	KOG3084				1	NADH pyrophosphatase I of the Nudix family of hydrolases
133844	KOG0967			2		ATP-dependent DNA ligase I
133919	KOG2043				1	Signaling protein SWIFT and related BRCT domain proteins
134195	KOG2310	1				DNA repair exonuclease MRE11
134490	KOG1037	2	1			NAD+ ADP-ribosyltransferase Parp, required for poly-ADP ribosylation of nuclear proteins
134831	KOG1625	1	1			DNA polymerase alpha-primase complex, polymerase-associated subunit B
134854	KOG2045	1		1		5'-3' exonuclease XRN1/KEM1/SEP1 involved in DNA strand exchange and mRNA turnover

135759	KOG0482		1	1		DNA replication licensing factor, MCM7 component
136408	KOG0981	1				DNA topoisomerase I
136747	KOG1979	1			1	DNA mismatch repair protein - MLH1 family
137574	KOG0250		1			DNA repair protein RAD18 (SMC family protein)
137902	KOG1798	1			2	DNA polymerase epsilon, catalytic subunit A
139221	KOG1921		1			Endonuclease III
139224	KOG4283				1	Transcription-coupled repair protein CSA, contains WD40 domain
139326	KOG0851		1	1		Single-stranded DNA-binding replication protein A (RPA), large (70 kD) subunit and related ssDNA-binding proteins
140303	KOG0133			5		Deoxyribodipyrimidine photolyase/cryptochrome
140797	KOG2994	1	3			Uracil DNA glycosylase
140993	KOG0480				1	DNA replication licensing factor, MCM6 component
141315	KOG1977				1	DNA mismatch repair protein - MLH3 family
141780	KOG1001			2		Helicase-like transcription factor HLTF/DNA helicase RAD5, DEAD-box superfamily
142312	KOG0479		1			DNA replication licensing factor, MCM3 component
144379	KOG2534				2	DNA polymerase IV (family X)
144488	KOG3997	1		1		Major apurinic/apyrimidinic endonuclease/3'-repair diesterase APN1
1448	KOG1929				2	Nucleotide excision repair factor NEF2, RAD4/CUT5 component
144839	KOG1967			1		DNA repair/transcription protein Mms19
145306	KOG1508	2		1		DNA replication factor/protein phosphatase inhibitor SET/SPR-2
146031	KOG1897	1		1		Damage-specific DNA binding complex, subunit DDB1
146472	KOG3041			1		Nucleoside diphosphate-sugar hydrolase of the MutT (NUDIX) family
147783	KOG1956	1				DNA topoisomerase III alpha
147923	KOG1123	1			1	RNA polymerase II transcription initiation/nucleotide excision repair factor TFIIH, 3'-5' helicase subunit SSL2
148310	KOG2401		1			Predicted MutS-related protein involved in mismatch repair
148368	KOG0981	1	2			DNA topoisomerase I
149006	KOG2043			2		Signaling protein SWIFT and related BRCT domain proteins
149131	KOG1020	1				Sister chromatid cohesion protein SCC2/Nipped-B
149406	KOG0442	1				Structure-specific endonuclease ERCC1-XPF, catalytic component XPF/ERCC4
149578	KOG4835		1			DNA-binding protein C1D involved in regulation of double-strand break repair
150009	KOG0390	1				DNA repair protein, SNF2 family
150084	KOG2372		1			Oxidation resistance protein
150119	KOG2248	1				3'-5' exonuclease
150169	KOG2851				1	Eukaryotic-type DNA primase, catalytic (small) subunit
150247	KOG1906	1				DNA polymerase sigma
150985	KOG0979			3		Structural maintenance of chromosome protein SMC5/Spr18, SMC superfamily

151487	KOG0989			1		Replication factor C, subunit RFC4
152531	KOG1001	1				Helicase-like transcription factor HLTF/DNA helicase RAD5, DEAD-box superfamily
152582	KOG0892			1		Protein kinase ATM/Tel1, involved in telomere length regulation and DNA repair
153112	KOG1167		1			Serine/threonine protein kinase of the CDC7 subfamily involved in DNA synthesis, repair and recombination
154179	KOG3041			1		Nucleoside diphosphate-sugar hydrolase of the MutT (NUDIX) family
154422	KOG2179				1	Nucleotide excision repair complex XPC-HR23B, subunit XPC/DPB11
155	KOG3041		1			Nucleoside diphosphate-sugar hydrolase of the MutT (NUDIX) family
155118	KOG2228		1			Origin recognition complex, subunit 4
157118	KOG1636		3	2		DNA polymerase delta processivity factor (proliferating cell nuclear antigen)
157714	KOG0991			1		Replication factor C, subunit RFC2
159024	KOG1803				1	DNA helicase
160687	KOG4086				1	Transcriptional regulator SOH1
183	KOG0351				2	ATP-dependent DNA helicase
1963	KOG1514			1		Origin recognition complex, subunit 1, and related proteins
2057	KOG1906	1				DNA polymerase sigma
20970	KOG2228		1			Origin recognition complex, subunit 4
23	KOG0890		1			Protein kinase of the PI-3 kinase family involved in mitotic growth, DNA repair and meiotic recombination
26548	KOG1508	2		1		DNA replication factor/protein phosphatase inhibitor SET/SPR-2
29109	KOG0889	1				Histone acetyltransferase SAGA, TRRAP/TRA1 component, PI-3 kinase superfamily
31133	KOG0217	1	1			Mismatch repair ATPase MSH6 (MutS family)
34781	KOG1294	1				Apurinic/apyrimidinic endonuclease and related enzymes
35082	KOG2534				1	DNA polymerase IV (family X)
36009	KOG1001			2		Helicase-like transcription factor HLTF/DNA helicase RAD5, DEAD-box superfamily
39170	KOG1921		1			Endonuclease III
43027	KOG1968				1	Replication factor C, subunit RFC1 (large subunit)
43084	KOG1968				1	Replication factor C, subunit RFC1 (large subunit)
4527	KOG3020	1				TatD-related DNase
46287	KOG2134			2		Polynucleotide kinase 3' phosphatase
48533	KOG0387			1		Transcription-coupled repair protein CSB/RAD26 (contains SNF2 family DNA-dependent ATPase domain)
77536	KOG2795		1			Catalytic subunit of the meiotic double strand break transesterase
79062	KOG0891			1		DNA-dependent protein kinase
81218	KOG1001			2		Helicase-like transcription factor HLTF/DNA helicase RAD5, DEAD-box superfamily
81746	KOG4139			1		Protein kinase essential for the initiation of DNA replication
82040	KOG3194			1	2	Checkpoint 9-1-1 complex, RAD1 component
85190	KOG2875				1	8-oxoguanine DNA glycosylase

86191	KOG1294	1				Apurinic/apyrimidinic endonuclease and related enzymes
91742	KOG0387			1		Transcription-coupled repair protein CSB/RAD26 (contains SNF2 family DNA-dependent ATPase domain)
92100	KOG1942			1		DNA helicase, TBP-interacting protein
**M-cell walls and membranes**						
128464	KOG2388			1		UDP-N-acetylglucosamine pyrophosphorylase
129991	KOG2515		1			Mannosyltransferase
130496	KOG1437		1	2		Fasciclin and related adhesion glycoproteins
130756	KOG3364				1	Membrane protein involved in organellar division
135904	KOG4748			2		Subunit of Golgi mannosyltransferase complex
137482	KOG1066		1			Glucosidase II catalytic (alpha) subunit and related enzymes, glycosyl hydrolase family 31
138952	KOG1268	2	3	2		Glucosamine 6-phosphate synthetases, contain amidotransferase and phosphosugar isomerase domains
140247	KOG0916	1	3	1		1,3-beta-glucan synthase/callose synthase catalytic subunit
140608	KOG3396	1				Glucosamine-phosphate N-acetyltransferase
140795	KOG1550	1	2	1		Extracellular protein SEL-1 and related proteins
142365	KOG2571		1	3		Chitin synthase/hyaluronan synthase (glycosyltransferases)
143107	KOG2571	4	7			Chitin synthase/hyaluronan synthase (glycosyltransferases)
143215	KOG3144		1		1	Ethanolamine-P-transferase GPI11/PIG-F, involved in glycosylphosphatidylinositol anchor biosynthesis
152332	KOG1371	2				UDP-glucose 4-epimerase/UDP-sulfoquinovose synthase
152437	KOG2686	1				Choline kinase
153214	KOG2571		2	1		Chitin synthase/hyaluronan synthase (glycosyltransferases)
154895	KOG2571		1			Chitin synthase/hyaluronan synthase (glycosyltransferases)
159644	KOG3332			1	1	N-acetylglucosaminyl phosphatidylinositol de-N-acetylase
16409	KOG3332			1	1	N-acetylglucosaminyl phosphatidylinositol de-N-acetylase
29317	KOG1066	2				Glucosidase II catalytic (alpha) subunit and related enzymes, glycosyl hydrolase family 31
38102	KOG1460			1		GDP-mannose pyrophosphorylase
77708	KOG1066	2				Glucosidase II catalytic (alpha) subunit and related enzymes, glycosyl hydrolase family 31
80164	KOG1460			1		GDP-mannose pyrophosphorylase
**N-cell motility**						
130286	KOG3896	1				Dynactin, subunit p62
138502	KOG4115	1		1		Dynein-associated protein Roadblock
139908	KOG3905			1		Dynein light intermediate chain
146344	KOG4081	4	3	1		Dynein light chain

O-posttranslational events

11107	KOG0742			1		AAA+-type ATPase
11690	KOG2100		1			Dipeptidyl aminopeptidase
126880	KOG1173	1				Anaphase-promoting complex (APC), Cdc16 subunit
127080	KOG0721	1		1		Molecular chaperone (DnaJ superfamily)
127278	KOG0366		1			Protein geranylgeranyltransferase type II, beta subunit
127322	KOG1282	3	2			Serine carboxypeptidases (lysosomal cathepsin A)
127419	KOG0170	2				E3 ubiquitin protein ligase
127602	KOG1358		2	2		Serine palmitoyltransferase
127848	KOG0687	1	1			26S proteasome regulatory complex, subunit RPN7/PSMD6
127864	KOG0800	1				FOG: Predicted E3 ubiquitin ligase
127947	KOG1556		2	3		26S proteasome regulatory complex, subunit RPN8/PSMD7
128024	KOG2160	1				Armadillo/beta-catenin-like repeat-containing protein
128117	KOG0712		1			Molecular chaperone (DnaJ superfamily)
128384	KOG2062	1				26S proteasome regulatory complex, subunit RPN2/PSMD1
128434	KOG2879				1	Predicted E3 ubiquitin ligase
128447	KOG0101	2	1	5		Molecular chaperones HSP70/HSC70, HSP70 superfamily
128455	KOG0879		2			U-snRNP-associated cyclophilin type peptidyl-prolyl cis-trans isomerase
128479	KOG0305		1			Anaphase promoting complex, Cdc20, Cdh1, and Ama1 subunits
128756	KOG2590			1		RNA-binding protein LARP/SRO9 and related La domain proteins
128965	KOG0357		2			Chaperonin complex component, TCP-1 epsilon subunit (CCT5)
129116	KOG3946				2	Glutaminyl cyclase
129135	KOG2608			1		Endoplasmic reticulum membrane-associated oxidoreductin involved in disulfide bond formation
129206	KOG0855		1			Alkyl hydroperoxide reductase, thiol specific antioxidant and related enzymes
129227	KOG0546	1	1			HSP90 co-chaperone CPR7/Cyclophilin
129404	KOG1339	5				Aspartyl protease
12948	KOG0743	1				AAA+-type ATPase
129481	KOG0418		2			Ubiquitin-protein ligase
129493	KOG0019	23	4	3		Molecular chaperone (HSP90 family)
129527	KOG0305	1				Anaphase promoting complex, Cdc20, Cdh1, and Ama1 subunits
129529	KOG4626			1		O-linked N-acetylglucosamine transferase OGT
129589	KOG0552	1	1	8	1	FKBP-type peptidyl-prolyl cis-trans isomerase
129627	KOG1051			1		Chaperone HSP104 and related ATP-dependent Clp proteases
129633	KOG4157	1		1		beta-1,6-N-acetylglucosaminyltransferase, contains WSC domain
130109	KOG1373		2	2		Transport protein Sec61, alpha subunit

130123	KOG0961			1		Predicted Zn2+-dependent endopeptidase, insulinase superfamily
130231	KOG3986				1	Protein phosphatase, regulatory subunit PPP1R3C/D
130287	KOG4157			1		beta-1,6-N-acetylglucosaminyltransferase, contains WSC domain
130377	KOG0940		1			Ubiquitin protein ligase RSP5/NEDD4
130504	KOG2015				1	NEDD8-activating complex, catalytic component UBA3
130574	KOG0417				1	Ubiquitin-protein ligase
130667	KOG1769		4			Ubiquitin-like proteins
130747	KOG3090	1		1		Prohibitin-like protein
130869	KOG0826	1		1		Predicted E3 ubiquitin ligase involved in peroxisome organization
130872	KOG1943				1	Beta-tubulin folding cofactor D
130908	KOG3061			1		Proteasome maturation factor
131046	KOG2100		1			Dipeptidyl aminopeptidase
131311	KOG4157			1		beta-1,6-N-acetylglucosaminyltransferase, contains WSC domain
131357	KOG3313				1	Molecular chaperone Prefoldin, subunit 3
131459	KOG0417	5	4	8		Ubiquitin-protein ligase
131461	KOG4157		3	1		beta-1,6-N-acetylglucosaminyltransferase, contains WSC domain
131548	KOG4157				1	beta-1,6-N-acetylglucosaminyltransferase, contains WSC domain
131628	KOG4151	1				Myosin assembly protein/sexual cycle protein and related proteins
131661	KOG2164	1	1			Predicted E3 ubiquitin ligase
131680	KOG0179		1			20S proteasome, regulatory subunit beta type PSMB1/PRE7
131742	KOG0841	10	9	5		Multifunctional chaperone (14-3-3 family)
131758	KOG0715	1			1	Molecular chaperone (DnaJ superfamily)
131866	KOG1339	1	1	3		Aspartyl protease
131904	KOG0743		1			AAA+-type ATPase
131971	KOG1282		1			Serine carboxypeptidases (lysosomal cathepsin A)
131999	KOG4157	1				beta-1,6-N-acetylglucosaminyltransferase, contains WSC domain
132167	KOG1555	1		3	2	26S proteasome regulatory complex, subunit RPN11
132229	KOG0549	1	1			FKBP-type peptidyl-prolyl cis-trans isomerase
132465	KOG0101	11	1	4		Molecular chaperones HSP70/HSC70, HSP70 superfamily
132474	KOG1815				1	Predicted E3 ubiquitin ligase
132504	KOG0734			1		AAA+-type ATPase containing the peptidase M41 domain
132566	KOG1046	1	2	1	1	Puromycin-sensitive aminopeptidase and related aminopeptidases
132660	KOG1153	3	1	1		Subtilisin-related protease/Vacuolar protease B
132758	KOG0907	1				Thioredoxin
132860	KOG0505	1				Myosin phosphatase, regulatory subunit

132969	KOG4157			1	beta-1,6-N-acetylglucosaminyltransferase, contains WSC domain
133039	KOG0691		1		Molecular chaperone (DnaJ superfamily)
133123	KOG4157			1	beta-1,6-N-acetylglucosaminyltransferase, contains WSC domain
133312	KOG0712		2		Molecular chaperone (DnaJ superfamily)
133436	KOG3607			1	Meltrins, fertilins and related Zn-dependent metalloproteinases of the ADAMs family
133619	KOG0867		1	1	Glutathione S-transferase
133908	KOG4157		1	1	beta-1,6-N-acetylglucosaminyltransferase, contains WSC domain
133938	KOG0406			2	Glutathione S-transferase
134074	KOG1554			1	COP9 signalosome, subunit CSN5
134257	KOG1442		2		GDP-fucose transporter
134490	KOG1037	2	1		NAD+ ADP-ribosyltransferase Parp, required for poly-ADP ribosylation of nuclear proteins
134573	KOG0743		1	1	AAA+-type ATPase
134680	KOG0714			1	Molecular chaperone (DnaJ superfamily)
134682	KOG0425			1	Ubiquitin-protein ligase
135203	KOG1760		1	1	Molecular chaperone Prefoldin, subunit 4
135222	KOG0741		1		AAA+-type ATPase
135714	KOG4157		7	2	beta-1,6-N-acetylglucosaminyltransferase, contains WSC domain
135900	KOG0175		1	2	20S proteasome, regulatory subunit beta type PSMB5/PSMB8/PRE2
135925	KOG0364		1	1	Chaperonin complex component, TCP-1 gamma subunit (CCT3)
136495	KOG0425	2			Ubiquitin-protein ligase
136537	KOG4157			3	beta-1,6-N-acetylglucosaminyltransferase, contains WSC domain
13680	KOG1339		1	2	Aspartyl protease
136876	KOG1439		2	3	RAB proteins geranylgeranyltransferase component A (RAB escort protein)
13693	KOG0730	2			AAA+-type ATPase
137195	KOG1641		1		Mitochondrial chaperonin
137219	KOG0101		1		Molecular chaperones HSP70/HSC70, HSP70 superfamily
137224	KOG1812		1		Predicted E3 ubiquitin ligase
137451	KOG1339		1		Aspartyl protease
137467	KOG4157		1		beta-1,6-N-acetylglucosaminyltransferase, contains WSC domain
137482	KOG1066		1		Glucosidase II catalytic (alpha) subunit and related enzymes, glycosyl hydrolase family 31
137486	KOG4157		1		beta-1,6-N-acetylglucosaminyltransferase, contains WSC domain
137510	KOG3566	3			Glycosylphosphatidylinositol anchor attachment protein GAA1
137555	KOG1867			1	Ubiquitin-specific protease
137693	KOG0361		1		Chaperonin complex component, TCP-1 eta subunit (CCT7)
137844	KOG1339	3	8	4	Aspartyl protease

137853	KOG0727	1		2		26S proteasome regulatory complex, ATPase RPT3
137943	KOG0541			2		Alkyl hydroperoxide reductase/peroxiredoxin
137975	KOG1812	1	1	2		Predicted E3 ubiquitin ligase
138223	KOG3478		1			Prefoldin subunit 6, KE2 family
138353	KOG0942	2				E3 ubiquitin protein ligase
138435	KOG2725		1			Cytochrome oxidase assembly factor COX15
138844	KOG1499		1	3		Protein arginine N-methyltransferase PRMT1 and related enzymes
138874	KOG4157		2			beta-1,6-N-acetylglucosaminyltransferase, contains WSC domain
138945	KOG1140			1		N-end rule pathway, recognition component UBR1
139622	KOG0178	1	2	1		20S proteasome, regulatory subunit alpha type PSMA4/PRE9
139913	KOG2018		1			Predicted dinucleotide-utilizing enzyme involved in molybdopterin and thiamine biosynthesis
139926	KOG1498		1			26S proteasome regulatory complex, subunit RPN5/PSMD12
140028	KOG1439	1			1	RAB proteins geranylgeranyltransferase component A (RAB escort protein)
140255	KOG3151		1	1		26S proteasome regulatory complex, subunit RPN12/PSMD8
140379	KOG0421		1	1		Ubiquitin-protein ligase
140456	KOG2738	1		1		Putative methionine aminopeptidase
140545	KOG2195	2		1		Transferrin receptor and related proteins containing the protease-associated (PA) domain
140585	KOG1039			1		Predicted E3 ubiquitin ligase
140795	KOG1550	1	3	2		Extracellular protein SEL-1 and related proteins
140834	KOG4628		1	1		Predicted E3 ubiquitin ligase
140907	KOG0742	6				AAA+-type ATPase
141071	KOG4157	1				beta-1,6-N-acetylglucosaminyltransferase, contains WSC domain
141137	KOG0885			2		Peptidyl-prolyl cis-trans isomerase
141143	KOG0884	1				Similar to cyclophilin-type peptidyl-prolyl cis-trans isomerase
141254	KOG3359			1		Dolichyl-phosphate-mannose:protein O-mannosyl transferase
141426	KOG0908		1			Thioredoxin-like protein
141550	KOG2100			1		Dipeptidyl aminopeptidase
141594	KOG4157					beta-1,6-N-acetylglucosaminyltransferase, contains WSC domain
141643	KOG1464		1	2		COP9 signalosome, subunit CSN2
141989	KOG1446		1			Histone H3 (Lys4) methyltransferase complex and RNA cleavage factor II complex, subunit SWD2
142040	KOG1339	1				Aspartyl protease
**142125**	**KOG0101**				**3**	**Molecular chaperones HSP70/HSC70, HSP70 superfamily**
142142	KOG0181	2	2			20S proteasome, regulatory subunit alpha type PSMA2/PRE8
142337	KOG0103	7	3	3		Molecular chaperones HSP105/HSP110/SSE1, HSP70 superfamily
142433	KOG0896			1		Ubiquitin-conjugating enzyme E2

142461	KOG0867		1	1		Glutathione S-transferase
142535	KOG3696			1		Aspartyl beta-hydroxylase
142538	KOG0100	5		2		Molecular chaperones GRP78/BiP/KAR2, HSP70 superfamily
142612	KOG0729	2	1	2		26S proteasome regulatory complex, ATPase RPT1
142615	KOG0358	2	3	2		Chaperonin complex component, TCP-1 delta subunit (CCT4)
142713	KOG2291	3			2	Oligosaccharyltransferase, alpha subunit (ribophorin I)
143106	KOG3525		2			Subtilisin-like proprotein convertase
143112	KOG1444		1	2		Nucleotide-sugar transporter VRG4/SQV-7
143215	KOG3144				2	Ethanolamine-P-transferase GPI11/PIG-F, involved in glycosylphosphatidylinositol anchor biosynthesis
143286	KOG3083		2	1		Prohibitin
143656	KOG0356			1		Mitochondrial chaperonin, Cpn60/Hsp60p
143670	KOG1816		1			Ubiquitin fusion-degradation protein
144227	KOG0726	3	3	1		26S proteasome regulatory complex, ATPase RPT2
144314	KOG2778	3		1		Ubiquitin C-terminal hydrolase
144327	KOG0802		2	2		E3 ubiquitin ligase
144458	KOG3501	1	3			Molecular chaperone Prefoldin, subunit 1
144594	KOG3192			1		Mitochondrial J-type chaperone
144612	KOG4614				1	Inner membrane protein required for assembly of the F0 sector of ATP synthase
144690	KOG3359	2	1	1		Dolichyl-phosphate-mannose:protein O-mannosyl transferase
145242	KOG4157	1		2		beta-1,6-N-acetylglucosaminyltransferase, contains WSC domain
145311	KOG1863		1			Ubiquitin carboxyl-terminal hydrolase
145554	KOG0712		2	1		Molecular chaperone (DnaJ superfamily)
146080	KOG0177	1		1		20S proteasome, regulatory subunit beta type PSMB2/PRE1
146112	KOG0183	1	2	1		20S proteasome, regulatory subunit alpha type PSMA7/PRE6
146119	KOG0710	28				Molecular chaperone (small heat-shock protein Hsp26/Hsp42)
146132	KOG0712	4		2		Molecular chaperone (DnaJ superfamily)
146150	KOG0182	2	3	1		20S proteasome, regulatory subunit alpha type PSMA6/SCL1
146164	KOG0908	2	3	1		Thioredoxin-like protein
146209	KOG0429		2			Ubiquitin-conjugating enzyme-related protein Ft1, involved in programmed cell death
146215	KOG0541	5	3	3	3	Alkyl hydroperoxide reductase/peroxiredoxin
146225	KOG1872	2		4		Ubiquitin-specific protease
146241	KOG0724			4		Zuotin and related molecular chaperones (DnaJ superfamily), contains DNA-binding domains
146255	KOG0359	3		3	2	Chaperonin complex component, TCP-1 zeta subunit (CCT6)
146273	KOG0894			2		Ubiquitin-protein ligase
146319	KOG0710	15				Molecular chaperone (small heat-shock protein Hsp26/Hsp42)

146387	KOG0717		3	2		Molecular chaperone (DnaJ superfamily)
146399	KOG2729	1	2			ER vesicle integral membrane protein involved in establishing cell polarity, signaling and protein degradation
146505	KOG0416	2	1			Ubiquitin-protein ligase
146528	KOG0675	3		2		Calnexin
146551	KOG0009	7	11	7		Ubiquitin-like/40S ribosomal S30 protein fusion
146577	KOG2689			1		Predicted ubiquitin regulatory protein
146592	KOG1724	7	3	4		SCF ubiquitin ligase, Skp1 component
146610	KOG3206	2		2	2	Alpha-tubulin folding cofactor B
146617	KOG0417	1				Ubiquitin-protein ligase
146697	KOG2160	4	1			Armadillo/beta-catenin-like repeat-containing protein
146703	KOG0191		6	3		Thioredoxin/protein disulfide isomerase
146764	KOG1153	1				Subtilisin-related protease/Vacuolar protease B
146928	KOG1239			1		Inner membrane protein translocase involved in respiratory chain assembly
146965	KOG0737				1	AAA+-type ATPase
147208	KOG0863	1	2	1		20S proteasome, regulatory subunit alpha type PSMA1/PRE5
147338	KOG0779		1			Protease, Ulp1 family
147394	KOG4266	3				Subtilisin kexin isozyme-1/site 1 protease, subtilase superfamily
147450	KOG2195	1	1			Transferrin receptor and related proteins containing the protease-associated (PA) domain
148028	KOG2292	2	2	1		Oligosaccharyltransferase, STT3 subunit
148129	KOG0173	1		1		20S proteasome, regulatory subunit beta type PSMB7/PSMB10/PUP1
148228	KOG4098			1		Molecular chaperone Prefoldin, subunit 2
148232	KOG1746		1			Defender against cell death protein/oligosaccharyltransferase, epsilon subunit
148430	KOG1815	1	2			Predicted E3 ubiquitin ligase
148614	KOG0911		2			Glutaredoxin-related protein
148968	KOG1349	1				Gpi-anchor transamidase
148987	KOG1812			1		Predicted E3 ubiquitin ligase
149283	KOG0419		1			Ubiquitin-protein ligase
149341	KOG0102	4		2		Molecular chaperones mortalin/PBP74/GRP75, HSP70 superfamily
149352	KOG0176	1	2	1		20S proteasome, regulatory subunit alpha type PSMA5/PUP2
149988	KOG0742			1		AAA+-type ATPase
150123	KOG1546			2		Metacaspase involved in regulation of apoptosis
150409	KOG2512		1	1		Beta-tubulin folding cofactor C
150720	KOG0739			1		AAA+-type ATPase
15079	KOG2195	2		1		Transferrin receptor and related proteins containing the protease-associated (PA) domain
15092	KOG0651	3	5	1		26S proteasome regulatory complex, ATPase RPT4

150957	KOG0001	30	20	13		Ubiquitin and ubiquitin-like proteins
151269	KOG2540	1				Cytochrome oxidase assembly factor COX11
151287	KOG3158	7	3		1	HSP90 co-chaperone p23
151419	KOG1752			1		Glutaredoxin and related proteins
151489	KOG0895	1		1		Ubiquitin-conjugating enzyme
151712	KOG1047	1	1			Bifunctional leukotriene A4 hydrolase/aminopeptidase LTA4H
151809	KOG4580		2			Component of vacuolar transporter chaperone (Vtc) involved in vacuole fusion
152000	KOG0652	1		1		26S proteasome regulatory complex, ATPase RPT5
152365	KOG1546		1	2		Metacaspase involved in regulation of apoptosis
152559	KOG1339		1			Aspartyl protease
152588	KOG0730	2	1	2		AAA+-type ATPase
152790	KOG0865	4	2	2		Cyclophilin type peptidyl-prolyl cis-trans isomerase
152830	KOG0939		1			E3 ubiquitin-protein ligase/Putative upstream regulatory element binding protein
152909	KOG3250		1			COP9 signalosome, subunit CSN7
152981	KOG1339		1			Aspartyl protease
153046	KOG0854	1	1	1		Alkyl hydroperoxide reductase, thiol specific antioxidant and related enzymes
153069	KOG0742	5	1	3		AAA+-type ATPase
153371	KOG0404		2			Thioredoxin reductase
153378	KOG0960	1	1	2		Mitochondrial processing peptidase, beta subunit, and related enzymes (insulinase superfamily)
153483	KOG4265		1			Predicted E3 ubiquitin ligase
153628	KOG2358	1		1		NifU-like domain-containing proteins
153649	KOG0939	2				E3 ubiquitin-protein ligase/Putative upstream regulatory element binding protein
153834	KOG2042			1		Ubiquitin fusion degradation protein-2
153859	KOG0728	1	1			26S proteasome regulatory complex, ATPase RPT6
153885	KOG1357		2			Serine palmitoyltransferase
153908	KOG0448		4	1		Mitofusin 1 GTPase, involved in mitochondrila biogenesis
153990	KOG1339		1	3		Aspartyl protease
154234	KOG0367		1			Protein geranylgeranyltransferase Type I, beta subunit
154327	KOG2012		1	1		Ubiquitin activating enzyme UBA1
154439	KOG1046	1		2		Puromycin-sensitive aminopeptidase and related aminopeptidases
154469	KOG2231		1		1	Predicted E3 ubiquitin ligase
154577	KOG0742		1			AAA+-type ATPase
155050	KOG2089	3	3	1		Metalloendopeptidase family - saccharolysin & thimet oligopeptidase
155093	KOG1568		1			Mitochondrial inner membrane protease, subunit IMP2
155232	KOG3470	2		2		Beta-tubulin folding cofactor A

155283	KOG0882	1	1			Cyclophilin-related peptidyl-prolyl cis-trans isomerase
155537	KOG2447			2		Oligosaccharyltransferase, delta subunit (ribophorin II)
155973	KOG0911	1	1			Glutaredoxin-related protein
156322	KOG2924		1			Deoxyhypusine synthase
156464	KOG0191	1				Thioredoxin/protein disulfide isomerase
156710	KOG2194	1				Aminopeptidases of the M20 family
157053	KOG3048	1		1		Molecular chaperone Prefoldin, subunit 5
157070	KOG0714	1	1	1		Molecular chaperone (DnaJ superfamily)
157100	KOG2100			1		Dipeptidyl aminopeptidase
157172	KOG1051	3		1		Chaperone HSP104 and related ATP-dependent Clp proteases
157453	KOG1051	12				Chaperone HSP104 and related ATP-dependent Clp proteases
157515	KOG0101		1			Molecular chaperones HSP70/HSC70, HSP70 superfamily
157521	KOG4114		3	1		Cytochrome c oxidase assembly protein PET191
157576	KOG0101			1		Molecular chaperones HSP70/HSC70, HSP70 superfamily
157649	KOG2918		1			Carboxymethyl transferase
157681	KOG4146			1		Ubiquitin-like protein
157963	KOG0428	1	1			Non-canonical ubiquitin conjugating enzyme 1
158012	KOG2067				1	Mitochondrial processing peptidase, alpha subunit
158370	KOG2182			2		Hydrolytic enzymes of the alpha/beta hydrolase fold
158381	KOG0714			1		Molecular chaperone (DnaJ superfamily)
158516	KOG0045	1			2	Cytosolic Ca2+-dependent cysteine protease (calpain), large subunit (EF-Hand protein superfamily)
158578	KOG0360	1				Chaperonin complex component, TCP-1 alpha subunit (CCT1)
158821	KOG4157				1	beta-1,6-N-acetylglucosaminyltransferase, contains WSC domain
159020	KOG0841	10	9	5		Multifunctional chaperone (14-3-3 family)
159174	KOG2541		1			Palmitoyl protein thioesterase
159181	KOG4157	1		3		beta-1,6-N-acetylglucosaminyltransferase, contains WSC domain
159353	KOG3457	10	9	5		Sec61 protein translocation complex, beta subunit
159436	KOG0548	9		1		Molecular co-chaperone STI1
159588	KOG1815	2				Predicted E3 ubiquitin ligase
159598	KOG3259	1		1		Peptidyl-prolyl cis-trans isomerase
160099	KOG4412		1			26S proteasome regulatory complex, subunit PSMD10
160199	KOG4400		1			E3 ubiquitin ligase interacting with arginine methyltransferase
160233	KOG1339	1	1			Aspartyl protease
160364	KOG0406		1			Glutathione S-transferase
160443	KOG0908	1				Thioredoxin-like protein

160446	KOG0548			1		Molecular co-chaperone STI1
160469	KOG0840		1			ATP-dependent Clp protease, proteolytic subunit
160795	KOG0880	8	5	4		Peptidyl-prolyl cis-trans isomerase
160834	KOG0710	13		1		Molecular chaperone (small heat-shock protein Hsp26/Hsp42)
16768	KOG2903			1		Predicted glutathione S-transferase
17201	KOG0867				1	Glutathione S-transferase
19731	KOG3493	1		1		Ubiquitin-like protein
20332	KOG0190			1		Protein disulfide isomerase (prolyl 4-hydroxylase beta subunit)
20499	KOG0961			1		Predicted Zn2+-dependent endopeptidase, insulinase superfamily
20565	KOG1868				1	Ubiquitin C-terminal hydrolase
207	KOG4626			1		O-linked N-acetylglucosamine transferase OGT
21592	KOG0170	2		1		E3 ubiquitin protein ligase
22596	KOG0743	1		1		AAA+-type ATPase
24799	KOG0717		1	2		Molecular chaperone (DnaJ superfamily)
24897	KOG0317				1	Predicted E3 ubiquitin ligase, integral peroxisomal membrane protein
26812	KOG0800			2		FOG: Predicted E3 ubiquitin ligase
27361	KOG1812		1			Predicted E3 ubiquitin ligase
27960	KOG0549	1	1			FKBP-type peptidyl-prolyl cis-trans isomerase
29181	KOG0779		1			Protease, Ulp1 family
29317	KOG1066	2				Glucosidase II catalytic (alpha) subunit and related enzymes, glycosyl hydrolase family 31
29988	KOG0427		1			Ubiquitin conjugating enzyme
32001	KOG0180		2			20S proteasome, regulatory subunit beta type PSMB3/PUP3
32815	KOG0176				1	20S proteasome, regulatory subunit alpha type PSMA5/PUP2
33483	KOG0429		2			Ubiquitin-conjugating enzyme-related protein Ft1, involved in programmed cell death
33703	KOG3160			2		Gamma-interferon inducible lysosomal thiol reductase
34284	KOG3061			1		Proteasome maturation factor
34304	KOG3160			2		Gamma-interferon inducible lysosomal thiol reductase
34426	KOG1812		1			Predicted E3 ubiquitin ligase
35658	KOG0365		3	1		Beta subunit of farnesyltransferase
38102	KOG1460			1		GDP-mannose pyrophosphorylase
39107	KOG3158	7	2	1	1	HSP90 co-chaperone p23
39908	KOG1546			2		Metacaspase involved in regulation of apoptosis
42332	KOG2195				1	Transferrin receptor and related proteins containing the protease-associated (PA) domain
44293	KOG0174			2		20S proteasome, regulatory subunit beta type PSMB6/PSMB9/PRE3
4443	KOG1339		1			Aspartyl protease

44468	KOG0544	4	4	1		FKBP-type peptidyl-prolyl cis-trans isomerase
44662	KOG0544	5	3	1		FKBP-type peptidyl-prolyl cis-trans isomerase
44843	KOG4642	1		1		Chaperone-dependent E3 ubiquitin protein ligase (contains TPR repeats)
4708	KOG0691		1			Molecular chaperone (DnaJ superfamily)
47259	KOG0737		1	1		AAA+-type ATPase
49192	KOG3607			1		Meltrins, fertilins and related Zn-dependent metalloproteinases of the ADAMs family
4987	KOG0191	1				Thioredoxin/protein disulfide isomerase
500	KOG2231		1	1	1	Predicted E3 ubiquitin ligase
50153	KOG0548			1		Molecular co-chaperone STI1
50884	KOG1752		1			Glutaredoxin and related proteins
52660	KOG0880	8	5	4		Peptidyl-prolyl cis-trans isomerase
53069	KOG1863		1			Ubiquitin carboxyl-terminal hydrolase
53908	KOG2930		1			SCF ubiquitin ligase, Rbx1 component
54917	KOG2182		1			Hydrolytic enzymes of the alpha/beta hydrolase fold
54959	KOG2182		1			Hydrolytic enzymes of the alpha/beta hydrolase fold
6829	KOG1813			1		Predicted E3 ubiquitin ligase
7480	KOG2884		2			26S proteasome regulatory complex, subunit RPN10/PSMD4
770	KOG1868				1	Ubiquitin C-terminal hydrolase
78486	KOG0180		2			20S proteasome, regulatory subunit beta type PSMB3/PUP3
79732	KOG0651	3	4			26S proteasome regulatory complex, ATPase RPT4
80164	KOG1460			1		GDP-mannose pyrophosphorylase
80722	KOG2358	1		1		NifU-like domain-containing proteins
81764	KOG4157			1		beta-1,6-N-acetylglucosaminyltransferase, contains WSC domain
82826	KOG0840		1			ATP-dependent Clp protease, proteolytic subunit
83812	KOG1815		1			Predicted E3 ubiquitin ligase
8726	KOG3355			1		Mitochondrial sulfhydryl oxidase involved in the biogenesis of cytosolic Fe/S proteins
89166	KOG3457	2	4	7		Sec61 protein translocation complex, beta subunit
89493	KOG2195				1	Transferrin receptor and related proteins containing the protease-associated (PA) domain
90432	KOG2903		1			Predicted glutathione S-transferase
90591	KOG0363			2		Chaperonin complex component, TCP-1 beta subunit (CCT2)
91923	KOG0911	1				Glutaredoxin-related protein
92038	KOG1870				1	Ubiquitin C-terminal hydrolase
9223	KOG0855		1			Alkyl hydroperoxide reductase, thiol specific antioxidant and related enzymes
94090	KOG1815		1			Predicted E3 ubiquitin ligase
94401	KOG1651	3		1		Glutathione peroxidase

94503	KOG3090	1		1		Prohibitin-like protein
94504	KOG0005		2			Ubiquitin-like protein
95049	KOG0710	14		1		Molecular chaperone (small heat-shock protein Hsp26/Hsp42)
9571	KOG3470	2		2		Beta-tubulin folding cofactor A
**P-inorganic metabolism**						
11490	KOG1650	1				Predicted K+/H+-antiporter
127014	KOG3599	1	1			Ca2+-modulated nonselective cation channel polycystin
127280	KOG2399		1			K+-dependent Na+:Ca2+ antiporter
127385	KOG0252	1	1	2		Inorganic phosphate transporter
128510	KOG0560	1	1			Sulfite reductase (ferredoxin)
128680	KOG0207	1				Cation transport ATPase
128996	KOG0682		1			Ammonia permease
130081	KOG4475		1			FOG: Immunoglobin and related proteins
131047	KOG0252		1			Inorganic phosphate transporter
131293	KOG0203				2	Na+/K+ ATPase, alpha subunit
131832	KOG3599	2	12	3		Ca2+-modulated nonselective cation channel polycystin
131875	KOG0202	1				Ca2+ transporting ATPase
132201	KOG1483	1				Zn2+ transporter ZNT1 and related Cd2+/Zn2+ transporters (cation diffusion facilitator superfamily)
132525	KOG1161		2			Protein involved in vacuolar polyphosphate accumulation, contains SPX domain
133370	KOG0047	2	4	1		Catalase
134071	KOG4475			1		FOG: Immunoglobin and related proteins
134873	KOG1172				2	Na+-independent Cl/HCO3 exchanger AE1 and related transporters (SLC4 family)
135139	KOG0672		1			Halotolerance protein HAL3 (contains flavoprotein domain)
135158	KOG3599	6	1	1		Ca2+-modulated nonselective cation channel polycystin
13567	KOG0252		1			Inorganic phosphate transporter
135724	KOG0876			1		Manganese superoxide dismutase
138239	KOG0039	1				Ferric reductase, NADH/NADPH oxidase and related proteins
138484	KOG3386		2	1	1	Copper transporter
139416	KOG0209	3		1		P-type ATPase
139680	KOG0252			1		Inorganic phosphate transporter
140066	KOG0056		1	1		Heavy metal exporter HMT1, ABC superfamily
140391	KOG0207		1	1	2	Cation transport ATPase
140545	KOG2195	1				Transferrin receptor and related proteins containing the protease-associated (PA) domain
141234	KOG0205	7	4	4		Plasma membrane H+-transporting ATPase

142437	KOG0672			1		Halotolerance protein HAL3 (contains flavoprotein domain)
142670	KOG3599	1	2	3		Ca2+-modulated nonselective cation channel polycystin
143414	KOG0252		1			Inorganic phosphate transporter
144050	KOG2493		2	5		Na+/Pi symporter
144647	KOG0236	1	1			Sulfate/bicarbonate/oxalate exchanger SAT-1 and related transporters (SLC26 family)
145639	KOG4554	1	2			Protein involved in inorganic phosphate transport
146305	KOG1603	1	2			Copper chaperone
146439	KOG0039	1		1	1	Ferric reductase, NADH/NADPH oxidase and related proteins
147450	KOG2195	1	1			Transferrin receptor and related proteins containing the protease-associated (PA) domain
147496	KOG1558		1	1		Fe2+/Zn2+ regulated transporter
147585	KOG2474		2			Zinc transporter and related ZIP domain-containing proteins
147615	KOG4499		1			Ca2+-binding protein Regucalcin/SMP30
147727	KOG2825	1			3	Putative arsenite-translocating ATPase
147790	KOG2882	1	3	2		p-Nitrophenyl phosphatase
148686	KOG0236			1		Sulfate/bicarbonate/oxalate exchanger SAT-1 and related transporters (SLC26 family)
148974	KOG1484	1				Putative Zn2+ transporter MSC2 (cation diffusion facilitator superfamily)
149409	KOG2662			2		Magnesium transporters: CorA family
149548	KOG0636	1	2			ATP sulfurylase (sulfate adenylyltransferase)
150286	KOG0252		1			Inorganic phosphate transporter
150319	KOG4475		1			FOG: Immunoglobin and related proteins
150642	KOG0207			1		Cation transport ATPase
150699	KOG0498			1		K+-channel ERG and related proteins, contain PAS/PAC sensor domain
15079	KOG2195	2		1		Transferrin receptor and related proteins containing the protease-associated (PA) domain
151207	KOG1120			1		Fe-S cluster biosynthesis protein ISA1 (contains a HesB-like domain)
151353	KOG0039				2	Ferric reductase, NADH/NADPH oxidase and related proteins
151634	KOG3126	2	2	4		Porin/voltage-dependent anion-selective channel protein
152289	KOG0047	2				Catalase
152568	KOG1485	2		1		Mitochondrial Fe2+ transporter MMT1 and related transporters (cation diffusion facilitator superfamily)
152585	KOG0056		1	1		Heavy metal exporter HMT1, ABC superfamily
153208	KOG1397	1				Ca2+/H+ antiporter VCX1 and related proteins
153728	KOG4505			1	9	Na+/H+ antiporter
153855	KOG1397		1		2	Ca2+/H+ antiporter VCX1 and related proteins
153884	KOG4505		1			Na+/H+ antiporter
154018	KOG0205	1	1			Plasma membrane H+-transporting ATPase
154625	KOG0876		2	1		Manganese superoxide dismutase

154863	KOG1397	1		1		Ca2+/H+ antiporter VCX1 and related proteins
156625	KOG0039	1				Ferric reductase, NADH/NADPH oxidase and related proteins
157528	KOG0441	6	11	1		Cu2+/Zn2+ superoxide dismutase SOD1
160937	KOG1528			1		Salt-sensitive 3'-phosphoadenosine-5'-phosphatase HAL2/SAL1
161047	KOG4656	1	5	4		Copper chaperone for superoxide dismutase
161051	KOG3386			1		Copper transporter
161054	KOG0682			2		Ammonia permease
161081	KOG1483	1				Zn2+ transporter ZNT1 and related Cd2+/Zn2+ transporters (cation diffusion facilitator superfamily)
161088	KOG2474		2			Zinc transporter and related ZIP domain-containing proteins
29278	KOG2474		2			Zinc transporter and related ZIP domain-containing proteins
29594	KOG0236			1		Sulfate/bicarbonate/oxalate exchanger SAT-1 and related transporters (SLC26 family)
31995	KOG3386			1		Copper transporter
32336	KOG3386			1		Copper transporter
40496	KOG0047				2	Catalase
42332	KOG2195				1	Transferrin receptor and related proteins containing the protease-associated (PA) domain
47235	KOG3599		2	3		Ca2+-modulated nonselective cation channel polycystin
50660	KOG0682			2		Ammonia permease
53278	KOG4656	1	6	4		Copper chaperone for superoxide dismutase
58	KOG1397	1			3	Ca2+/H+ antiporter VCX1 and related proteins
81240	KOG1397			1		Ca2+/H+ antiporter VCX1 and related proteins
8253	KOG1120			1		Fe-S cluster biosynthesis protein ISA1 (contains a HesB-like domain)
82773	KOG0682			2		Ammonia permease
85895	KOG1397		1			Ca2+/H+ antiporter VCX1 and related proteins
87802	KOG1485				2	Mitochondrial Fe2+ transporter MMT1 and related transporters (cation diffusion facilitator superfamily)
89493	KOG2195				1	Transferrin receptor and related proteins containing the protease-associated (PA) domain
94658	KOG3126	2	2	4		Porin/voltage-dependent anion-selective channel protein

T-Signal Transduction

10932	KOG3519		1		1	Invasion-inducing protein TIAM1/CDC24 and related RhoGEF GTPases
12339	KOG0671				1	LAMMER dual specificity kinases
126920	KOG2085		1			Serine/threonine protein phosphatase 2A, regulatory subunit
126985	KOG4363	1		1		Putative growth response protein
127014	KOG3599	1	1			Ca2+-modulated nonselective cation channel polycystin
127040	KOG4297	1				C-type lectin
127118	KOG4297	1				C-type lectin

127205	KOG3895	1	1		2	Synaptic vesicle protein Synapsin
127315	KOG0032		1	2		Ca2+/calmodulin-dependent protein kinase, EF-Hand protein superfamily
127363	KOG0082		1			G-protein alpha subunit (small G protein superfamily)
127478	KOG4297	1				C-type lectin
127525	KOG2262		2			Sexual differentiation process protein ISP4
127634	KOG4035			1		Coeffector of mDia Rho GTPase, regulates actin polymerization and cell adhesion turnover
127739	KOG2210		2			Oxysterol-binding protein
127851	KOG2043		2			Signaling protein SWIFT and related BRCT domain proteins
128219	KOG1116		1			Sphingosine kinase, involved in sphingolipid metabolism
128614	KOG0519		1			Sensory transduction histidine kinase
128623	KOG0698	1				Serine/threonine protein phosphatase
128906	KOG2595			1	1	Predicted GTPase activator protein
129182	KOG0698		1	3		Serine/threonine protein phosphatase
129293	KOG0521	1				Putative GTPase activating proteins (GAPs)
129300	KOG0998		1			Synaptic vesicle protein EHS-1 and related EH domain proteins
129415	KOG0748			1		Predicted membrane proteins, contain hemolysin III domain
129432	KOG2262			1		Sexual differentiation process protein ISP4
129529	KOG4626			1		O-linked N-acetylglucosamine transferase OGT
129868	KOG4297	1				C-type lectin
130231	KOG3986				1	Protein phosphatase, regulatory subunit PPP1R3C/D
130310	KOG4162			1		Predicted calmodulin-binding protein
130535	KOG1187		1			Serine/threonine protein kinase
130576	KOG2999		1			Regulator of Rac1, required for phagocytosis and cell migration
130604	KOG0519				2	Sensory transduction histidine kinase
130638	KOG3872		1			FOG: FHA domain
130659	KOG3519		1		1	Invasion-inducing protein TIAM1/CDC24 and related RhoGEF GTPases
130680	KOG4297		1		1	C-type lectin
130703	KOG1187		1			Serine/threonine protein kinase
130898	KOG0706			1		Predicted GTPase-activating protein
131087	KOG1450				1	Predicted Rho GTPase-activating protein
131157	KOG4297		1			C-type lectin
131199	KOG3329	1	2	1		RAN guanine nucleotide release factor
131201	KOG3275	2	4			Zinc-binding protein of the histidine triad (HIT) family
131266	KOG3348		2	1		BolA (bacterial stress-induced morphogen)-related protein
131656	KOG1187				2	Serine/threonine protein kinase

131832	KOG3599	2	2	11	1	Ca2+-modulated nonselective cation channel polycystin
131861	KOG4369	1				RTK signaling protein MASK/UNC-44
132193	KOG0230	1				Phosphatidylinositol-4-phosphate 5-kinase and related FYVE finger-containing proteins
132408	KOG0668			1		Casein kinase II, alpha subunit
132511	KOG4019		1			Calcineurin-mediated signaling pathway inhibitor DSCR1
132529	KOG1924		1			RhoA GTPase effector DIA/Diaphanous
132578	KOG3171			3		Conserved phosducin-like protein
132795	KOG4297		1	1	1	C-type lectin
132860	KOG0505	1				Myosin phosphatase, regulatory subunit
133015	KOG3589				4	G protein signaling regulators
133072	KOG4297	4	1	8		C-type lectin
133085	KOG4293				1	Predicted membrane protein, contains DoH and Cytochrome b-561/ferric reductase transmembrane domains
133147	KOG0585		1			Ca2+/calmodulin-dependent protein kinase kinase beta and related serine/threonine protein kinases
133486	KOG1251	1				Serine racemase
133533	KOG0519	1				Sensory transduction histidine kinase
133564	KOG4361			1		BCL2-associated athanogene-like proteins and related BAG family chaperone regulators
133629	KOG4297	1				C-type lectin
133685	KOG4297	2		1		C-type lectin
133728	KOG3671		1			Actin regulatory protein (Wiskott-Aldrich syndrome protein)
133786	KOG0578	1		1	2	p21-activated serine/threonine protein kinase
133829	KOG4297			1		C-type lectin
133919	KOG2043				1	Signaling protein SWIFT and related BRCT domain proteins
133922	KOG1164	1		2		Casein kinase (serine/threonine/tyrosine protein kinase)
134074	KOG1554			1		COP9 signalosome, subunit CSN5
134108	KOG1187		1	1		Serine/threonine protein kinase
134128	KOG0585	1	1			Ca2+/calmodulin-dependent protein kinase kinase beta and related serine/threonine protein kinases
134137	KOG3791		1			Predicted RNA-binding protein involved in translational regulation
134165	KOG0583	1				Serine/threonine protein kinase
134220	KOG2262	2				Sexual differentiation process protein ISP4
134459	KOG3886	1				GTP-binding protein
134712	KOG1922			1		Rho GTPase effector BNI1 and related formins
135158	KOG3599	7	1	1		Ca2+-modulated nonselective cation channel polycystin
135287	KOG0643	1	1	1		Translation initiation factor 3, subunit i (eIF-3i)/TGF-beta receptor-interacting protein (TRIP-1)
135375	KOG1487			2	1	GTP-binding protein DRG1 (ODN superfamily)
135653	KOG0201		1	1		Serine/threonine protein kinase

135826	KOG2130			1		Phosphatidylserine-specific receptor PtdSerR, contains JmjC domain
135988	KOG1547		2	4		Septin CDC10 and related P-loop GTPases
136196	KOG0748		1			Predicted membrane proteins, contain hemolysin III domain
136266	KOG2867	1				Phosphotyrosyl phosphatase activator
136417	KOG0044		3			Ca2+ sensor (EF-Hand superfamily)
136442	KOG2641			1		Predicted seven transmembrane receptor - rhodopsin family
136513	KOG4297	1		3		C-type lectin
136566	KOG0371			1		Serine/threonine protein phosphatase 2A, catalytic subunit
136849	KOG0507		1	1		CASK-interacting adaptor protein (caskin) and related proteins with ankyrin repeats and SAM domain
136948	KOG2262	2	1	1		Sexual differentiation process protein ISP4
137146	KOG1363		1		2	Predicted regulator of the ubiquitin pathway (contains UAS and UBX domains)
137193	KOG0078	1				GTP-binding protein SEC4, small G protein superfamily, and related Ras family GTP-binding proteins
137770	KOG2397	1	1		2	Protein kinase C substrate, 80 KD protein, heavy chain
137796	KOG4389	1				Acetylcholinesterase/Butyrylcholinesterase
137893	KOG3979	1				FGF receptor activating protein 1
137944	KOG2128		1	2		Ras GTPase-activating protein family - IQGAP
138227	KOG0229			1	1	Phosphatidylinositol-4-phosphate 5-kinase
138338	KOG1187		1	1		Serine/threonine protein kinase
138844	KOG1499		1	3		Protein arginine N-methyltransferase PRMT1 and related enzymes
139704	KOG0598			1		Ribosomal protein S6 kinase and related proteins
139709	KOG3575			1		FOG: Hormone receptors
139763	KOG2283			1		Clathrin coat dissociation kinase GAK/PTEN/Auxilin and related tyrosine phosphatases
139899	KOG2322			1		N-methyl-D-aspartate receptor glutamate-binding subunit
140303	KOG0133			5		Deoxyribodipyrimidine photolyase/cryptochrome
140330	KOG0598	1		1		Ribosomal protein S6 kinase and related proteins
140414	KOG0531	1	2	3		Protein phosphatase 1, regulatory subunit, and related proteins
140480	KOG0032	1				Ca2+/calmodulin-dependent protein kinase, EF-Hand protein superfamily
14076	KOG4293	1				Predicted membrane protein, contains DoH and Cytochrome b-561/ferric reductase transmembrane domains
140795	KOG1550	1	2	2		Extracellular protein SEL-1 and related proteins
141006	KOG4203	1		2		Armadillo/beta-Catenin/plakoglobin
141173	KOG0373			1		Serine/threonine specific protein phosphatase involved in cell cycle control, PP2A-related
141302	KOG3671		1			Actin regulatory protein (Wiskott-Aldrich syndrome protein)
141581	KOG1786	1				Lysosomal trafficking regulator LYST and related BEACH and WD40 repeat proteins
141643	KOG1464		2	1		COP9 signalosome, subunit CSN2
141716	KOG4136	2				Predicted mitochondrial cholesterol transporter

141873	KOG3205		3	1		Rho GDP-dissociation inhibitor
141935	KOG1027	2	1	1		Serine/threonine protein kinase and endoribonuclease ERN1/IRE1, sensor of the unfolded protein response pathway
142127	KOG0661	1	2	1		MAPK related serine/threonine protein kinase
142670	KOG3599	1	2	4		Ca2+-modulated nonselective cation channel polycystin
142790	KOG0581	2				Mitogen-activated protein kinase kinase (MAP2K)
142946	KOG0748		1			Predicted membrane proteins, contain hemolysin III domain
143095	KOG2265		1	1		Nuclear distribution protein NUDC
143595	KOG2127		2	1		Calmodulin-binding protein CRAG, contains DENN domain
144101	KOG1118		1			Lysophosphatidic acid acyltransferase endophilin/SH3GL, involved in synaptic vesicle formation
144669	KOG1187		2			Serine/threonine protein kinase
144749	KOG0703	1				Predicted GTPase-activating protein
144760	KOG4293			1		Predicted membrane protein, contains DoH and Cytochrome b-561/ferric reductase transmembrane domains
145322	KOG0034		3	2		Ca2+/calmodulin-dependent protein phosphatase (calcineurin subunit B), EF-Hand superfamily protein
145861	KOG1212	1	1			Amidases
146071	KOG1486		2	1		GTP-binding protein DRG2 (ODN superfamily)
146191	KOG0660	7		4	1	Mitogen-activated protein kinase
146399	KOG2729	1	2	1		ER vesicle integral membrane protein involved in establishing cell polarity, signaling and protein degradation
146573	KOG3217	3			1	Protein tyrosine phosphatase
146638	KOG0027	6	24	12	3	Calmodulin and related proteins (EF-Hand superfamily)
146724	KOG4203	3	2	2		Armadillo/beta-Catenin/plakoglobin
146751	KOG1118	4	2	2		Lysophosphatidic acid acyltransferase endophilin/SH3GL, involved in synaptic vesicle formation
147071	KOG0169				1	Phosphoinositide-specific phospholipase C
147218	KOG2174		2	1		Leptin receptor gene-related protein
147224	KOG2197		1			Ypt/Rab-specific GTPase-activating protein GYP7 and related proteins
147261	KOG1215	1				Low-density lipoprotein receptors containing Ca2+-binding EGF-like domains
147406	KOG1215		1			Low-density lipoprotein receptors containing Ca2+-binding EGF-like domains
147452	KOG1435		1			Sterol reductase/lamin B receptor
147615	KOG4499		1			Ca2+-binding protein Regucalcin/SMP30
147751	KOG0581		1			Mitogen-activated protein kinase kinase (MAP2K)
147835	KOG1259	1				Nischarin, modulator of integrin alpha5 subunit action
148010	KOG0037	1				Ca2+-binding protein, EF-Hand protein superfamily
148265	KOG0519		2	1	1	Sensory transduction histidine kinase
148346	KOG2210		2		1	Oxysterol-binding protein
148556	KOG4076	1	1			Regulator of ATP-sensitive K+ channels Alpha-endosulfine/ARPP-19 and related cAMP-regulated phosphoproteins
148743	KOG0279	9	4	7		G protein beta subunit-like protein

148949	KOG0372		1	1		Serine/threonine specific protein phosphatase involved in glycogen accumulation, PP2A-related
149006	KOG2043			2		Signaling protein SWIFT and related BRCT domain proteins
149115	KOG2268	2	1			Serine/threonine protein kinase
149483	KOG1547				1	Septin CDC10 and related P-loop GTPases
149630	KOG2085			2	1	Serine/threonine protein phosphatase 2A, regulatory subunit
149807	KOG0519			2		Sensory transduction histidine kinase
149856	KOG1212			1		Amidases
150085	KOG0660	2	1			Mitogen-activated protein kinase
150562	KOG0519				3	Sensory transduction histidine kinase
150699	KOG0498			1		K+-channel ERG and related proteins, contain PAS/PAC sensor domain
150843	KOG2232	2	1			Ceramidases
150909	KOG0598				1	Ribosomal protein S6 kinase and related proteins
151141	KOG0592		1			3-phosphoinositide-dependent protein kinase (PDK1)
151145	KOG4561	1	1			Uncharacterized conserved protein, contains TBC domain
151225	KOG2839		1			Diadenosine and diphosphoinositol polyphosphate phosphohydrolase
151229	KOG0084		4	1		GTPase Rab1/YPT1, small G protein superfamily, and related GTP-binding proteins
151396	KOG1902		1			Putative signal transduction protein involved in RNA splicing
151402	KOG0902	1				Phosphatidylinositol 4-kinase
151535	KOG0618	1				Serine/threonine phosphatase 2C containing leucine-rich repeats, similar to SCN circadian oscillatory protein (SCOP)
151594	KOG1435			1		Sterol reductase/lamin B receptor
151848	KOG1187			1		Serine/threonine protein kinase
151976	KOG2666			1		UDP-glucose/GDP-mannose dehydrogenase
152029	KOG2210				1	Oxysterol-binding protein
152359	KOG1217	4				Fibrillins and related proteins containing Ca2+-binding EGF-like domains
152582	KOG0892			1		Protein kinase ATM/Tel1, involved in telomere length regulation and DNA repair
152596	KOG0082	1		3	1	G-protein alpha subunit (small G protein superfamily)
152615	KOG1187		1		1	Serine/threonine protein kinase
152855	KOG1630	3		1	1	Growth hormone-induced protein and related proteins
152868	KOG0519	1				Sensory transduction histidine kinase
152909	KOG3250		1			COP9 signalosome, subunit CSN7
153053	KOG1218		1	1		Proteins containing Ca2+-binding EGF-like domains
153399	KOG0374	2		1		Serine/threonine specific protein phosphatase PP1, catalytic subunit
153468	KOG2124	1	1		1	Glycosylphosphatidylinositol anchor synthesis protein
153817	KOG0198				1	MEKK and related serine/threonine protein kinases
154192	KOG1212	3	2			Amidases

154450	KOG2264	1				Exostosin EXT1L
154539	KOG0211			1		Protein phosphatase 2A regulatory subunit A and related proteins
154663	KOG0787	1				Dehydrogenase kinase
154704	KOG4297		1			C-type lectin
154761	KOG3519	1	1			Invasion-inducing protein TIAM1/CDC24 and related RhoGEF GTPases
154891	KOG0787				1	Dehydrogenase kinase
155290	KOG0598		2			Ribosomal protein S6 kinase and related proteins
155482	KOG0748			1		Predicted membrane proteins, contain hemolysin III domain
155635	KOG0251	1		1		Clathrin assembly protein AP180 and related proteins, contain ENTH domain
155842	KOG0660	1	2			Mitogen-activated protein kinase
156154	KOG0698				1	Serine/threonine protein phosphatase
156631	KOG0256			2		1-aminocyclopropane-1-carboxylate synthase, and related proteins
156952	KOG4203	1	1			Armadillo/beta-Catenin/plakoglobin
157026	KOG1187	1		2		Serine/threonine protein kinase
157081	KOG2867	1				Phosphotyrosyl phosphatase activator
157393	KOG2126				2	Glycosylphosphatidylinositol anchor synthesis protein
157400	KOG4363	1			1	Putative growth response protein
157499	KOG0581				2	Mitogen-activated protein kinase kinase (MAP2K)
157590	KOG0169	2			1	Phosphoinositide-specific phospholipase C
157648	KOG0906			1		Phosphatidylinositol 3-kinase VPS34, involved in signal transduction
158015	KOG3872	1				FOG: FHA domain
158261	KOG0660				1	Mitogen-activated protein kinase
158454	KOG0519	1				Sensory transduction histidine kinase
158516	KOG0045	1			2	Cytosolic Ca2+-dependent cysteine protease (calpain), large subunit (EF-Hand protein superfamily)
158851	KOG0169	1	1			Phosphoinositide-specific phospholipase C
158900	KOG0374			2		Serine/threonine specific protein phosphatase PP1, catalytic subunit
159209	KOG3170	2				Conserved phosducin-like protein
159326	KOG2370			1	2	Cactin
159774	KOG0694		1	3		Serine/threonine protein kinase
160054	KOG0598		1		1	Ribosomal protein S6 kinase and related proteins
160100	KOG4297				2	C-type lectin
160131	KOG1818				2	Membrane trafficking and cell signaling protein HRS, contains VHS and FYVE domains
1739	KOG2043			1		Signaling protein SWIFT and related BRCT domain proteins
176	KOG3417				2	Ras1 guanine nucleotide exchange factor
18713	KOG4297	1	2	1		C-type lectin

19135	KOG0027		1	1		Calmodulin and related proteins (EF-Hand superfamily)
20010	KOG0230	1				Phosphatidylinositol-4-phosphate 5-kinase and related FYVE finger-containing proteins
207	KOG4626			1		O-linked N-acetylglucosamine transferase OGT
21808	KOG0201				1	Serine/threonine protein kinase
22003	KOG0199			1		ACK and related non-receptor tyrosine kinases
22952	KOG1187		1			Serine/threonine protein kinase
23	KOG0890		1			Protein kinase of the PI-3 kinase family involved in mitotic growth, DNA repair and meiotic recombination
26283	KOG2264	1				Exostosin EXT1L
2757	KOG1902		1			Putative signal transduction protein involved in RNA splicing
28981	KOG0671				1	LAMMER dual specificity kinases
28999	KOG1435			3		Sterol reductase/lamin B receptor
29109	KOG0889	1				Histone acetyltransferase SAGA, TRRAP/TRA1 component, PI-3 kinase superfamily
29134	KOG1435		1	3		Sterol reductase/lamin B receptor
29997	KOG4363	1		1		Putative growth response protein
30240	KOG0583			1		Serine/threonine protein kinase
30351	KOG1187	3	1	1		Serine/threonine protein kinase
31997	KOG2262			1	4	Sexual differentiation process protein ISP4
33408	KOG4297		1			C-type lectin
33890	KOG1290	2	3	1		Serine/threonine protein kinase
34166	KOG0199			1		ACK and related non-receptor tyrosine kinases
3796	KOG1499			1		Protein arginine N-methyltransferase PRMT1 and related enzymes
38000	KOG4369	1				RTK signaling protein MASK/UNC-44
41459	KOG2264	2	1	2		Exostosin EXT1L
41568	KOG0581	1	1			Mitogen-activated protein kinase kinase (MAP2K)
42096	KOG2264	2	1	2		Exostosin EXT1L
42500	KOG2128			1		Ras GTPase-activating protein family - IQGAP
43161	KOG4203	1		2		Armadillo/beta-Catenin/plakoglobin
43498	KOG0583				2	Serine/threonine protein kinase
46760	KOG0027		2	3		Calmodulin and related proteins (EF-Hand superfamily)
47055	KOG0027		3	3		Calmodulin and related proteins (EF-Hand superfamily)
47235	KOG3599		2	3		Ca2+-modulated nonselective cation channel polycystin
47390	KOG0598		1		1	Ribosomal protein S6 kinase and related proteins
47458	KOG3589				3	G protein signaling regulators
47694	KOG1909				1	Ran GTPase-activating protein
48379	KOG2262				2	Sexual differentiation process protein ISP4

49155	KOG0583		2	1		Serine/threonine protein kinase
51228	KOG1354	1				Serine/threonine protein phosphatase 2A, regulatory subunit
51365	KOG1354	1				Serine/threonine protein phosphatase 2A, regulatory subunit
51486	KOG3797				1	Peripheral-type benzodiazepine receptor and related proteins
51833	KOG0198				1	MEKK and related serine/threonine protein kinases
51924	KOG0903	1				Phosphatidylinositol 4-kinase, involved in intracellular trafficking and secretion
52267	KOG3699		2	1		Cytoskeletal protein Adducin
52423	KOG3699		2	1		Cytoskeletal protein Adducin
5273	KOG0660				1	Mitogen-activated protein kinase
55193	KOG1118	3	3	1		Lysophosphatidic acid acyltransferase endophilin/SH3GL, involved in synaptic vesicle formation
78083	KOG2262			3	3	Sexual differentiation process protein ISP4
78098	KOG1487			2	1	GTP-binding protein DRG1 (ODN superfamily)
821	KOG1989			1		ARK protein kinase family
83194	KOG1989			1		ARK protein kinase family
85273	KOG0521	1				Putative GTPase activating proteins (GAPs)
88927	KOG3739	1				Stress-activated MAP kinase-interacting protein, Sin1p
91139	KOG2675	1	2	1		Adenylate cyclase-associated protein (CAP/Srv2p)
92316	KOG1251			1	1	Serine racemase
**U-secretion**						
126968	KOG1953	1				Targeting complex (TRAPP) subunit
127205	KOG3895	1	1			Synaptic vesicle protein Synapsin
128115	KOG3081	1	1			Vesicle coat complex COPI, epsilon subunit
128208	KOG4635			1		Vacuolar import and degradation protein
128354	KOG3284	1				Vacuolar sorting protein VPS28
128488	KOG0928				2	Pattern-formation protein/guanine nucleotide exchange factor
128532	KOG0932	1				Guanine nucleotide exchange factor EFA6
128994	KOG3998		1	2		Putative cargo transport protein ERV29
128996	KOG3796		1			Ammonium transporter RHBG
129046	KOG1688		2			Golgi proteins involved in ER retention (RER)
129091	KOG3368				1	Transport protein particle (TRAPP) complex subunit
129119	KOG2273	1				Membrane coat complex Retromer, subunit VPS5/SNX1, Sorting nexins, and related PX domain-containing proteins
129135	KOG2608			1		Endoplasmic reticulum membrane-associated oxidoreductin involved in disulfide bond formation
129222	KOG3336			2		Predicted member of the intramitochondrial sorting protein family
129300	KOG0998		1			Synaptic vesicle protein EHS-1 and related EH domain proteins

129524	KOG1062		2	1		Vesicle coat complex AP-1, gamma subunit
129583	KOG0096	2	5	8		GTPase Ran/TC4/GSP1 (nuclear protein transport pathway), small G protein superfamily
129636	KOG2319	2		1		Vacuolar assembly/sorting protein VPS9
129673	KOG4072		1			Signal peptidase complex, subunit SPC25
129729	KOG0566			1		Inositol-1,4,5-triphosphate 5-phosphatase (synaptojanin), INP51/INP52/INP53 family
129759	KOG0845			1		Nuclear pore complex, Nup98 component (sc Nup145/Nup100/Nup116)
129922	KOG4112			1		Signal peptidase subunit
129991	KOG2515		1			Mannosyltransferase
130031	KOG4224		1			Armadillo repeat protein VAC8 required for vacuole fusion, inheritance and cytosol-to-vacuole protein targeting
130109	KOG1373		3	4		Transport protein Sec61, alpha subunit
130211	KOG2115	1				Vacuolar sorting protein VPS45
130311	KOG2021	1				Nuclear mRNA export factor receptor LOS1/Exportin-t (importin beta superfamily)
130354	KOG3771	2	2	2		Amphiphysin
130632	KOG0934		1	3		Clathrin adaptor complex, small subunit
130932	KOG0277			1		Peroxisomal targeting signal type 2 receptor
131102	KOG0812			2		SNARE protein SED5/Syntaxin 5
131221	KOG1985	1	1			Vesicle coat complex COPII, subunit SEC24/subunit SFB2
131374	KOG3630	2				Nuclear pore complex, Nup214/CAN component
131379	KOG0058	2		1		Peptide exporter, ABC superfamily
131761	KOG3133	1				40 kDa farnesylated protein associated with peroxisomes
131893	KOG1058	1				Vesicle coat complex COPI, beta subunit
131909	KOG1652		1	1		Mitochondrial import inner membrane translocase, subunit TIM17
131963	KOG0276		1	2		Vesicle coat complex COPI, beta' subunit
132112	KOG3907		2	1		ZIP-like zinc transporter proteins
132296	KOG0439					VAMP-associated protein involved in inositol metabolism
132347	KOG0092		1	1		GTPase Rab5/YPT51 and related small G protein superfamily GTPases
132404	KOG3875	1				Peroxisomal biogenesis protein peroxin
132653	KOG0985	1	3	1		Vesicle coat protein clathrin, heavy chain
132769	KOG2274	1				Predicted importin 9
132900	KOG1809			1		Vacuolar protein sorting-associated protein
133422	KOG1586	3	2	1		Protein required for fusion of vesicles in vesicular transport, alpha-SNAP
133641	KOG2063			1		Vacuolar assembly/sorting proteins VPS39/VAM6/VPS3
133979	KOG0446			1		Vacuolar sorting protein VPS1, dynamin, and related proteins
134028	KOG3745				2	Exocyst subunit - Sec10p
134044	KOG0446	1	2	3		Vacuolar sorting protein VPS1, dynamin, and related proteins

134075	KOG2273				1	Membrane coat complex Retromer, subunit VPS5/SNX1, Sorting nexins, and related PX domain-containing proteins
134257	KOG1442		2			GDP-fucose transporter
134945	KOG0090	2				Signal recognition particle receptor, beta subunit (small G protein superfamily)
135436	KOG2319	1				Vacuolar assembly/sorting protein VPS9
135609	KOG0946	1		1		ER-Golgi vesicle-tethering protein p115
136197	KOG2058			1		Ypt/Rab GTPase activating protein
136909	KOG1792			4		Reticulon
137167	KOG1991	2			3	Nuclear transport receptor RANBP7/RANBP8 (importin beta superfamily)
137193	KOG0078	1				GTP-binding protein SEC4, small G protein superfamily, and related Ras family GTP-binding proteins
137259	KOG2527		1	2		Sorting nexin SNX11
137979	KOG2218	1				ER to golgi transport protein/RAD50-interacting protein 1
138121	KOG4573	1				Phosphoprotein involved in cytoplasm to vacuole targeting and autophagy
138128	KOG2104	1	2			Nuclear transport factor 2
138254	KOG0057	1	2			Mitochondrial Fe/S cluster exporter, ABC superfamily
138548	KOG3330	1	1			Transport protein particle (TRAPP) complex subunit
138643	KOG3202	1				SNARE protein TLG1/Syntaxin 6
138960	KOG2834		1			Nuclear pore complex, rNpl4 component (sc Npl4)
138964	KOG4699	2	1		3	Preprotein translocase subunit Sec66
139042	KOG3745	1	1			Exocyst subunit - Sec10p
139576	KOG2171		1			Karyopherin (importin) beta 3
139720	KOG0859	1				Synaptobrevin/VAMP-like protein
139876	KOG3498	2	1	2		Preprotein translocase, gamma subunit
140314	KOG0086		3	1		GTPase Rab4, small G protein superfamily
140711	KOG3480		1			Mitochondrial import inner membrane translocase, subunits TIM10/TIM12
141581	KOG1786	1				Lysosomal trafficking regulator LYST and related BEACH and WD40 repeat proteins
141819	KOG3878	2				Protein involved in maintenance of Golgi structure and ER-Golgi transport
142019	KOG2635	2	2			Medium subunit of clathrin adaptor complex
142415	KOG1332	1				Vesicle coat complex COPII, subunit SEC13
142476	KOG4186			1		Peroxisomal biogenesis protein (peroxin)
143112	KOG1444		2	1		Nucleotide-sugar transporter VRG4/SQV-7
143259	KOG2993	1				Cytoplasm to vacuole targeting protein
143328	KOG1301		1			Vesicle trafficking protein Sly1 (Sec1 family)
143952	KOG3342			1		Signal peptidase I
144261	KOG2273			2		Membrane coat complex Retromer, subunit VPS5/SNX1, Sorting nexins, and related PX domain-containing proteins
144355	KOG0058	1				Peptide exporter, ABC superfamily

144363	KOG4031			2		Vesicle coat protein clathrin, light chain
144632	KOG1692	2	3			Putative cargo transport protein EMP24 (p24 protein family)
144672	KOG0819			1		Annexin
144746	KOG3385	1				V-SNARE
144943	KOG0092	2	1	1		GTPase Rab5/YPT51 and related small G protein superfamily GTPases
145049	KOG0781			1		Signal recognition particle receptor, alpha subunit
145354	KOG1725		1	1		Protein involved in membrane traffic (YOP1/TB2/DP1/HVA22 family)
145883	KOG2668			1		Flotillins
146094	KOG0077	1	5	1		Vesicle coat complex COPII, GTPase subunit SAR1
146108	KOG3839	1	1	1		Lectin VIP36, involved in the transport of glycoproteins carrying high mannose-type glycans
146197	KOG4056	1	1	1		Translocase of outer mitochondrial membrane complex, subunit TOM20
146304	KOG0811	1		1		SNARE protein PEP12/VAM3/Syntaxin 7/Syntaxin 17
146306	KOG1666		2	4		V-SNARE
146380	KOG0860	1				Synaptobrevin/VAMP-like protein
146399	KOG2729	1	2			ER vesicle integral membrane protein involved in establishing cell polarity, signaling and protein degradation
146456	KOG3325		2			Membrane coat complex Retromer, subunit VPS29/PEP11
146459	KOG4111		1			Translocase of outer mitochondrial membrane complex, subunit TOM22
146525	KOG3343	2	1	1		Vesicle coat complex COPI, zeta subunit
146572	KOG3337	4				Protein similar to predicted member of the intramitochondrial sorting protein family
146579	KOG0071		1	1		GTP-binding ADP-ribosylation factor Arf6 (dArf3)
146629	KOG3771			1		Amphiphysin
146660	KOG0070	5	4	4		GTP-binding ADP-ribosylation factor Arf1
146679	KOG3487		1			TRAPP 20 K subunit
146928	KOG1239			1		Inner membrane protein translocase involved in respiratory chain assembly
147005	KOG0937		1	1		Adaptor complexes medium subunit family
147049	KOG3315		2			Transport protein particle (TRAPP) complex subunit
147058	KOG0565				1	Inositol polyphosphate 5-phosphatase and related proteins
147198	KOG0862			1		Synaptobrevin/VAMP-like protein SEC22
147281	KOG3225			1		Mitochondrial import inner membrane translocase, subunit TIM22
147300	KOG1693	1				emp24/gp25L/p24 family of membrane trafficking proteins
147335	KOG3372		1			Signal peptidase complex subunit
147706	KOG2020	2	1			Nuclear transport receptor CRM1/MSN5 (importin beta superfamily)
148132	KOG3630				1	Nuclear pore complex, Nup214/CAN component
148556	KOG4076	1	1			Regulator of ATP-sensitive K+ channels Alpha-endosulfine/ARPP-19 and related cAMP-regulated phosphoproteins
148635	KOG2887	1				Membrane protein involved in ER to Golgi transport

148763	KOG2148	1				Exocyst protein Sec3
148848	KOG2655	1		1		Septin family protein (P-loop GTPase)
148947	KOG2273	1				Membrane coat complex Retromer, subunit VPS5/SNX1, Sorting nexins, and related PX domain-containing proteins
149055	KOG2215	1				Exocyst complex subunit
149116	KOG2081				1	Nuclear transport regulator
149621	KOG1078		1			Vesicle coat complex COPI, gamma subunit
149763	KOG4324			1	1	Guanine nucleotide exchange factor
149817	KOG3232	1				Vacuolar assembly/sorting protein DID2
150066	KOG1761	1				Signal recognition particle, subunit Srp14
150097	KOG2020	1		1		Nuclear transport receptor CRM1/MSN5 (importin beta superfamily)
150138	KOG3983				1	Golgi protein
150316	KOG4697				1	Integral membrane protein involved in transport between the late Golgi and endosome
150324	KOG1984		1	2		Vesicle coat complex COPII, subunit SFB3
150464	KOG2604	1	1	1		Subunit of cis-Golgi transport vesicle tethering complex - Sec34p
150609	KOG0845	1	1		1	Nuclear pore complex, Nup98 component (sc Nup145/Nup100/Nup116)
151229	KOG0084		4	1	1	GTPase Rab1/YPT1, small G protein superfamily, and related GTP-binding proteins
151303	KOG1092	1	2	1		Ypt/Rab-specific GTPase-activating protein GYP1
151455	KOG1060	1		2		Vesicle coat complex AP-3, beta subunit
151581	KOG0566				1	Inositol-1,4,5-triphosphate 5-phosphatase (synaptojanin), INP51/INP52/INP53 family
151618	KOG0547				1	Translocase of outer mitochondrial membrane complex, subunit TOM70/TOM72
151757	KOG1690				1	emp24/gp25L/p24 family of membrane trafficking proteins
151809	KOG4580		2			Component of vacuolar transporter chaperone (Vtc) involved in vacuole fusion
151833	KOG1691			2		emp24/gp25L/p24 family of membrane trafficking proteins
152051	KOG2319	1		1		Vacuolar assembly/sorting protein VPS9
152060	KOG2580				1	Mitochondrial import inner membrane translocase, subunit TIM44
152419	KOG2655		4	3		Septin family protein (P-loop GTPase)
152543	KOG1241	1		2	2	Karyopherin (importin) beta 1
152713	KOG0087	7	4	8		GTPase Rab11/YPT3, small G protein superfamily
152750	KOG2171	1			1	Karyopherin (importin) beta 3
152772	KOG0292		1			Vesicle coat complex COPI, alpha subunit
153536	KOG0094	1	1			GTPase Rab6/YPT6/Ryh1, small G protein superfamily
153568	KOG1077	1			1	Vesicle coat complex AP-2, alpha subunit
154987	KOG2101				1	Intermediate filament-like protein, sorting nexins, and related proteins containing PX (PhoX) domain(s)
155093	KOG1568		1			Mitochondrial inner membrane protease, subunit IMP2
155635	KOG0251	1		1		Clathrin assembly protein AP180 and related proteins, contain ENTH domain

155639	KOG3106	1	2			ER lumen protein retaining receptor
155910	KOG0439	1		1		VAMP-associated protein involved in inositol metabolism
156231	KOG0307	1				Vesicle coat complex COPII, subunit SEC31
156416	KOG1107	1	1			Membrane coat complex Retromer, subunit VPS35
156911	KOG0809	1				SNARE protein TLG2/Syntaxin 16
156919	KOG2060	1				Rab3 effector RIM1 and related proteins, contain PDZ and C2 domains
157005	KOG0946	1		3		ER-Golgi vesicle-tethering protein p115
157019	KOG3065				1	SNAP-25 (synaptosome-associated protein) component of SNARE complex
157152	KOG3208	1			2	SNARE protein GS28
157648	KOG0906			1		Phosphatidylinositol 3-kinase VPS34, involved in signal transduction
15770	KOG2927		1			Membrane component of ER protein translocation complex
157818	KOG0166	2	2	1		Karyopherin (importin) alpha
157855	KOG2655		1	1		Septin family protein (P-loop GTPase)
157927	KOG1059			1		Vesicle coat complex AP-3, delta subunit
158001	KOG1119	1				Mitochondrial Fe-S cluster biosynthesis protein ISA2 (contains a HesB-like domain)
158019	KOG2280				1	Vacuolar assembly/sorting protein VPS16
158037	KOG0446				1	Vacuolar sorting protein VPS1, dynamin, and related proteins
158216	KOG2176		1		1	Exocyst complex, subunit SEC15
158350	KOG4540		1	1		Putative lipase essential for disintegration of autophagic bodies inside the vacuole
158785	KOG4449	1	1	2		Translocase of outer mitochondrial membrane complex, subunit TOM7
159627	KOG0864		4	3		Ran-binding protein RANBP1 and related RanBD domain proteins
159809	KOG3465	1	2			Signal recognition particle, subunit Srp9
160131	KOG1818				2	Membrane trafficking and cell signaling protein HRS, contains VHS and FYVE domains
160160	KOG1656	1	3	1		Protein involved in glucose derepression and pre-vacuolar endosome protein sorting
160277	KOG0072		3	3		GTP-binding ADP-ribosylation factor-like protein ARL1
160362	KOG0845	1			1	Nuclear pore complex, Nup98 component (sc Nup145/Nup100/Nup116)
160702	KOG0819		1			Annexin
16421	KOG3103	1		1		Rab GTPase interacting factor, Golgi membrane protein
19106	KOG3465	1	1			Signal recognition particle, subunit Srp9
20453	KOG2101	2	1			Intermediate filament-like protein, sorting nexins, and related proteins containing PX (PhoX) domain(s)
24260	KOG4809	1		1		Rab6 GTPase-interacting protein involved in endosome-to-TGN transport
28233	KOG3480		1			Mitochondrial import inner membrane translocase, subunits TIM10/TIM12
28353	KOG4449	1	1	2		Translocase of outer mitochondrial membrane complex, subunit TOM7
2877	KOG2655			1		Septin family protein (P-loop GTPase)
3298	KOG0946	1		1		ER-Golgi vesicle-tethering protein p115

35895	KOG2066			1		Vacuolar assembly/sorting protein VPS41
36671	KOG1985	1	1			Vesicle coat complex COPII, subunit SEC24/subunit SFB2
37595	KOG2066			1		Vacuolar assembly/sorting protein VPS41
38666	KOG1060	1		2		Vesicle coat complex AP-3, beta subunit
38868	KOG1092	1	2	1		Ypt/Rab-specific GTPase-activating protein GYP1
39622	KOG0811	1		1		SNARE protein PEP12/VAM3/Syntaxin 7/Syntaxin 17
49830	KOG1586	3	2	1		Protein required for fusion of vesicles in vesicular transport, alpha-SNAP
4984	KOG0819		1			Annexin
51924	KOG0903	1				Phosphatidylinositol 4-kinase, involved in intracellular trafficking and secretion
51927	KOG2034				1	Vacuolar sorting protein PEP3/VPS18
52775	KOG1986	1	1		1	Vesicle coat complex COPII, subunit SEC23
54688	KOG2655			1		Septin family protein (P-loop GTPase)
79634	KOG3229		2	1		Vacuolar sorting protein VPS24
79692	KOG4521	1				Nuclear pore complex, Nup160 component
79737	KOG2834		1			Nuclear pore complex, rNpl4 component (sc Npl4)
79912	KOG2740	1				Clathrin-associated protein medium chain
79937	KOG1691			2		emp24/gp25L/p24 family of membrane trafficking proteins
80531	KOG4097	6	4	8		Succinate dehydrogenase membrane anchor subunit and related proteins
83218	KOG2034				1	Vacuolar sorting protein PEP3/VPS18
87465	KOG3330	1	1			Transport protein particle (TRAPP) complex subunit
87797	KOG4521	1				Nuclear pore complex, Nup160 component
88749	KOG2655		4	3		Septin family protein (P-loop GTPase)
93217	KOG0903	1				Phosphatidylinositol 4-kinase, involved in intracellular trafficking and secretion
94369	KOG4076				1	Regulator of ATP-sensitive K+ channels Alpha-endosulfine/ARPP-19 and related cAMP-regulated phosphoproteins
94532	KOG0935	1		2		Clathrin adaptor complex, small subunit
**V-defense**						
127040	KOG4297	1				C-type lectin
127118	KOG4297	1				C-type lectin
127478	KOG4297	1				C-type lectin
128358	KOG1529			1		Mercaptopyruvate sulfurtransferase/thiosulfate sulfurtransferase
129396	KOG1216		2	1		von Willebrand factor and related coagulation proteins
129703	KOG1515			1		Arylacetamide deacetylase
129868	KOG4297	1				C-type lectin
130680	KOG4297		1			C-type lectin

131157	KOG4297		1			C-type lectin
131211	KOG0624		1			dsRNA-activated protein kinase inhibitor P58, contains TPR and DnaJ domains
132493	KOG4730		1			D-arabinono-1, 4-lactone oxidase
132795	KOG4297		1	1	1	C-type lectin
133072	KOG4297	4	1	9		C-type lectin
133629	KOG4297	1				C-type lectin
133685	KOG4297	2		1		C-type lectin
133829	KOG4297			1		C-type lectin
135366	KOG1216	5	4	1		von Willebrand factor and related coagulation proteins
135570	KOG1216	3	2	7		von Willebrand factor and related coagulation proteins
136513	KOG4297	1		3		C-type lectin
139054	KOG1216		1	1		von Willebrand factor and related coagulation proteins
141163	KOG1502		7	13		Flavonol reductase/cinnamoyl-CoA reductase
143942	KOG4003			1		Pyrazinamidase/nicotinamidase PNC1
145665	KOG1826			1		Ras GTPase activating protein RasGAP/neurofibromin
146183	KOG1515	1	1	1		Arylacetamide deacetylase
146435	KOG1962	1	4	1		B-cell receptor-associated protein and related proteins
147012	KOG2764				1	Putative transcriptional regulator DJ-1
148705	KOG1515		1			Arylacetamide deacetylase
150498	KOG1726	2				HVA22/DP1 gene product-related proteins
150924	KOG1515	1		4	1	Arylacetamide deacetylase
151712	KOG1047	1			1	Bifunctional leukotriene A4 hydrolase/aminopeptidase LTA4H
1529	KOG2622				1	Putative myrosinase precursor
154704	KOG4297		1			C-type lectin
155156	KOG1515				1	Arylacetamide deacetylase
158131	KOG1502		6	3		Flavonol reductase/cinnamoyl-CoA reductase
159395	KOG2764	1		3		Putative transcriptional regulator DJ-1
159902	KOG1502			1		Flavonol reductase/cinnamoyl-CoA reductase
18713	KOG4297	1	2	1		C-type lectin
23138	KOG4297	1				C-type lectin
26090	KOG1515	1	1	2		Arylacetamide deacetylase
33408	KOG4297		1			C-type lectin
37109	KOG1216	1				von Willebrand factor and related coagulation proteins
53261	KOG1502	2				Flavonol reductase/cinnamoyl-CoA reductase
7314	KOG1572		1	2		Predicted protein tyrosine phosphatase

79919	KOG4297	1	2	1		C-type lectin
92318	KOG4003			1		Pyrazinamidase/nicotinamidase PNC1
**W-extracellular structures**						
128976	KOG3871	1				Cell adhesion complex protein bystin
129396	KOG1216		2	1		von Willebrand factor and related coagulation proteins
130496	KOG1437		1	2		Fasciclin and related adhesion glycoproteins
135056	KOG1747		2			Protein tyrosine kinase 9/actin monomer-binding protein
135366	KOG1216	1	5	1		von Willebrand factor and related coagulation proteins
135570	KOG1216	3	2	7		von Willebrand factor and related coagulation proteins
136638	KOG1736		1			Glia maturation factor beta
139054	KOG1216		1	1		von Willebrand factor and related coagulation proteins
152276	KOG3781			1		Dystroglycan
37109	KOG1216	1				von Willebrand factor and related coagulation proteins
86260	KOG1216			1		von Willebrand factor and related coagulation proteins
**Y-Nuclear structure**						
127744	KOG2992			1		Nucleolar GTPase/ATPase p130
128118	KOG2992	1				Nucleolar GTPase/ATPase p130
128239	KOG2992			1		Nucleolar GTPase/ATPase p130
129159	KOG2992	2				Nucleolar GTPase/ATPase p130
129759	KOG0845			1		Nuclear pore complex, Nup98 component (sc Nup145/Nup100/Nup116)
130070	KOG2086		1			Protein tyrosine phosphatase SHP1/Cofactor for p97 ATPase-mediated vesicle membrane fusion
130135	KOG2992		1			Nucleolar GTPase/ATPase p130
130311	KOG2021	1				Nuclear mRNA export factor receptor LOS1/Exportin-t (importin beta superfamily)
130450	KOG2992		1			Nucleolar GTPase/ATPase p130
130474	KOG2992				2	Nucleolar GTPase/ATPase p130
130909	KOG2992			1		Nucleolar GTPase/ATPase p130
131340	KOG2992	1				Nucleolar GTPase/ATPase p130
131374	KOG3630	2				Nuclear pore complex, Nup214/CAN component
131645	KOG2992				1	Nucleolar GTPase/ATPase p130
131817	KOG2992	1				Nucleolar GTPase/ATPase p130
132769	KOG2274	1				Predicted importin 9
133879	KOG2992		1			Nucleolar GTPase/ATPase p130
134406	KOG2992		2	1		Nucleolar GTPase/ATPase p130

134495	KOG2992	1				Nucleolar GTPase/ATPase p130
137167	KOG1991	2			3	Nuclear transport receptor RANBP7/RANBP8 (importin beta superfamily)
138960	KOG2834		1			Nuclear pore complex, rNpl4 component (sc Npl4)
139345	KOG2992		1			Nucleolar GTPase/ATPase p130
139576	KOG2171		1			Karyopherin (importin) beta 3
140190	KOG2992	3				hypothetical protein
140292	KOG2992	1	2			Nucleolar GTPase/ATPase p130
140826	KOG2992		1	1		Nucleolar GTPase/ATPase p130
140839	KOG2992				1	Nucleolar GTPase/ATPase p130
141271	KOG2992		1	3		Nucleolar GTPase/ATPase p130
141366	KOG2992	1		1		Nucleolar GTPase/ATPase p130
141422	KOG2992			1		Nucleolar GTPase/ATPase p130
143197	KOG2992				1	Nucleolar GTPase/ATPase p130
146614	KOG2992	5	1	4		Nucleolar GTPase/ATPase p130
147706	KOG2020	2	1			Nuclear transport receptor CRM1/MSN5 (importin beta superfamily)
147759	KOG2992	1				Nucleolar GTPase/ATPase p130
148132	KOG3630				2	Nuclear pore complex, Nup214/CAN component
149778	KOG2992			2		Nucleolar GTPase/ATPase p130
150097	KOG2020	1		1		Nuclear transport receptor CRM1/MSN5 (importin beta superfamily)
150609	KOG0845	1	2			Nuclear pore complex, Nup98 component (sc Nup145/Nup100/Nup116)
152427	KOG2992			1		Nucleolar GTPase/ATPase p130
152543	KOG1241	1		3	2	Karyopherin (importin) beta 1
152750	KOG2171	1			1	Karyopherin (importin) beta 3
155079	KOG2992	1		2	1	Nucleolar GTPase/ATPase p130
157228	KOG2992		1	1		Nucleolar GTPase/ATPase p130
157286	KOG2992				1	Nucleolar GTPase/ATPase p130
160362	KOG0845	1			1	Nuclear pore complex, Nup98 component (sc Nup145/Nup100/Nup116)
30147	KOG2992				1	Nucleolar GTPase/ATPase p130
30547	KOG2992				2	Nucleolar GTPase/ATPase p130
34840	KOG2992	2		1		Nucleolar GTPase/ATPase p130
35729	KOG2992			1	2	Nucleolar GTPase/ATPase p130
38329	KOG2992				1	Nucleolar GTPase/ATPase p130
78453	KOG2992				1	Nucleolar GTPase/ATPase p130
79112	KOG2992			1	2	Nucleolar GTPase/ATPase p130
79622	KOG2992				1	Nucleolar GTPase/ATPase p130

79692	KOG4521	1				Nuclear pore complex, Nup160 component
79737	KOG2834		1			Nuclear pore complex, rNpl4 component (sc Npl4)
87797	KOG4521	1				Nuclear pore complex, Nup160 component
94244	KOG2992				1	Nucleolar GTPase/ATPase p130
**Z-cytosceleton**						
11786	KOG0161			1		Myosin class II heavy chain
127052	KOG0161				1	Myosin class II heavy chain
127223	KOG1376			2		Alpha tubulin
127634	KOG4035			1		Coeffector of mDia Rho GTPase, regulates actin polymerization and cell adhesion turnover
128353	KOG0161		1			Myosin class II heavy chain
128426	KOG1830	2				Wiskott Aldrich syndrome proteins
129107	KOG0613		1			Projectin/twitchin and related proteins
129691	KOG0676			1		Actin and related proteins
130468	KOG1587		1			Cytoplasmic dynein intermediate chain
130479	KOG0161			2		Myosin class II heavy chain
130652	KOG1374				2	Gamma tubulin
130768	KOG0161	1				Myosin class II heavy chain
130793	KOG4462	1			2	WASP-interacting protein VRP1/WIP, contains WH2 domain
130879	KOG1057			1		Arp2/3 complex-interacting protein VIP1/Asp1, involved in regulation of actin cytoskeleton
130967	KOG3671			1		Actin regulatory protein (Wiskott-Aldrich syndrome protein)
131013	KOG0980		2	1		Actin-binding protein SLA2/Huntingtin-interacting protein Hip1
131255	KOG0242	1				Kinesin-like protein
131509	KOG0836			1		F-actin capping protein, alpha subunit
132391	KOG0613		1	2		Projectin/twitchin and related proteins
132475	KOG4568	1				Cytoskeleton-associated protein and related proteins
132483	KOG1376	1	1	2		Alpha tubulin
132529	KOG1924		1			RhoA GTPase effector DIA/Diaphanous
133697	KOG0516	1	1			Dystonin, GAS (Growth-arrest-specific protein), and related proteins
133728	KOG3671		1			Actin regulatory protein (Wiskott-Aldrich syndrome protein)
134712	KOG1922			1		Rho GTPase effector BNI1 and related formins
134980	KOG0161		1			Myosin class II heavy chain
135080	KOG3000	1		2		Microtubule-binding protein involved in cell cycle control
135988	KOG1547	1	2	4		Septin CDC10 and related P-loop GTPases
136041	KOG1375	1	5	5		Beta tubulin

136901	KOG0613		1	1		Projectin/twitchin and related proteins
137421	KOG0613		3			Projectin/twitchin and related proteins
138661	KOG1654	6	1	5		Microtubule-associated anchor protein involved in autophagy and membrane trafficking
138970	KOG0613	1			1	Projectin/twitchin and related proteins
140701	KOG0243			1		Kinesin-like protein
141003	KOG2659		1	1		LisH motif-containing protein
141006	KOG4203	1		2		Armadillo/beta-Catenin/plakoglobin
141036	KOG3380	1		2		Actin-related protein Arp2/3 complex, subunit ARPC5
141302	KOG3671				1	Actin regulatory protein (Wiskott-Aldrich syndrome protein)
14132	KOG1376	1	1	3		Alpha tubulin
141335	KOG3155			1		Actin-related protein Arp2/3 complex, subunit ARPC3
142327	KOG2675	1	2	1		Adenylate cyclase-associated protein (CAP/Srv2p)
142817	KOG3476		1			Microtubule-associated protein CRIPT
143355	KOG3565			1	1	Cdc42-interacting protein CIP4
144443	KOG0161		3	2		Myosin class II heavy chain
144651	KOG0242	1		1		Kinesin-like protein
144686	KOG1523	2	1			Actin-related protein Arp2/3 complex, subunit ARPC1/p41-ARC
145808	KOG1727	3	6	11		Microtubule-binding protein (translationally controlled tumor protein)
145883	KOG2668			1		Flotillins
146172	KOG0046	1	4	2		Ca2+-binding actin-bundling protein (fimbrin/plastin), EF-Hand protein superfamily
146195	KOG1755	4	5	4		Profilin
146227	KOG1876		1	1		Actin-related protein Arp2/3 complex, subunit ARPC4
146603	KOG1735	3	12	6		Actin depolymerizing factor
146717	KOG1003	5	9	6		Actin filament-coating protein tropomyosin
146724	KOG4203	3	2	2		Armadillo/beta-Catenin/plakoglobin
147167	KOG0516		1			Dystonin, GAS (Growth-arrest-specific protein), and related proteins
147364	KOG0313		2			Microtubule binding protein YTM1 (contains WD40 repeats)
147552	KOG1840		1			Kinesin light chain
147671	KOG0517			1		Beta-spectrin
147835	KOG1259	1				Nischarin, modulator of integrin alpha5 subunit action
147940	KOG0035	1	7			Ca2+-binding actin-bundling protein (actinin), alpha chain (EF-Hand protein superfamily)
147954	KOG0303		2			Actin-binding protein Coronin, contains WD40 repeats
148148	KOG2826	1	5	1		Actin-related protein Arp2/3 complex, subunit ARPC2
148848	KOG2655	1		1		Septin family protein (P-loop GTPase)
149285	KOG0240			1	1	Kinesin (SMY1 subfamily)

149350	KOG2001				1	Gamma-tubulin complex, DGRIP84/SPC97 component
149483	KOG1547				1	Septin CDC10 and related P-loop GTPases
151248	KOG0678	2	1	3		Actin-related protein Arp2/3 complex, subunit Arp3
151544	KOG3958		1	1		Putative dynamitin
151800	KOG0318	2	2	1		WD40 repeat stress protein/actin interacting protein
152251	KOG0613		1			Projectin/twitchin and related proteins
152419	KOG2655		4	3		Septin family protein (P-loop GTPase)
152998	KOG1003	1				Actin filament-coating protein tropomyosin
153294	KOG0161	1		1		Myosin class II heavy chain
154987	KOG2101		1		1	Intermediate filament-like protein, sorting nexins, and related proteins containing PX (PhoX) domain(s)
155032	KOG1375	3	6	7		Beta tubulin
156952	KOG4203	1	1			Armadillo/beta-Catenin/plakoglobin
156969	KOG0160	1	3	2	2	Myosin class V heavy chain
157685	KOG0676	17	3	18		Actin and related proteins
157855	KOG2655		1	1		Septin family protein (P-loop GTPase)
158649	KOG0676			1		Actin and related proteins
159070	KOG2842		1	1		Interferon-related protein PC4 like
159490	KOG3430		3	2		Dynein light chain type 1
160239	KOG0677	3		1		Actin-related protein Arp2/3 complex, subunit Arp2
160725	KOG3655	1		1		Drebrins and related actin binding proteins
160740	KOG1830		1	2		Wiskott Aldrich syndrome proteins
16561	KOG0517			1		Beta-spectrin
20453	KOG2101		1			Intermediate filament-like protein, sorting nexins, and related proteins containing PX (PhoX) domain(s)
2768	KOG0613				1	Projectin/twitchin and related proteins
2877	KOG2655			1		Septin family protein (P-loop GTPase)
30051	KOG0161			1		Myosin class II heavy chain
31347	KOG0516	1				Dystonin, GAS (Growth-arrest-specific protein), and related proteins
31706	KOG3655			2		Drebrins and related actin binding proteins
40669	KOG3655				1	Drebrins and related actin binding proteins
40807	KOG3655				1	Drebrins and related actin binding proteins
42373	KOG3430		2	2		Dynein light chain type 1
43161	KOG4203	2		1		Armadillo/beta-Catenin/plakoglobin
45494	KOG1840				1	Kinesin light chain
45606	KOG1840			1		Kinesin light chain
45942	KOG1853	1				LIS1-interacting protein NUDE

47574	KOG3756	1			1	Pinin (desmosome-associated protein)
52257	KOG0613	1	1			Projectin/twitchin and related proteins
52267	KOG3699		2	1		Cytoskeletal protein Adducin
52423	KOG3699		2	1		Cytoskeletal protein Adducin
52784	KOG3655	1	1	1		Drebrins and related actin binding proteins
53101	KOG0028				2	Ca2+-binding protein (centrin/caltractin), EF-Hand superfamily protein
54688	KOG2655			1		Septin family protein (P-loop GTPase)
55018	KOG0613	1		1		Projectin/twitchin and related proteins
7573	KOG3174	1			1	F-actin capping protein, beta subunit
81483	KOG1853	1				LIS1-interacting protein NUDE
82360	KOG0677	3		1		Actin-related protein Arp2/3 complex, subunit Arp2
85503	KOG3655			2		Drebrins and related actin binding proteins
85599	KOG0613	1	2			Projectin/twitchin and related proteins
86627	KOG1840		1	2		Kinesin light chain
88749	KOG2655		3	2		Septin family protein (P-loop GTPase)
91139	KOG2675	1	2	1		Adenylate cyclase-associated protein (CAP/Srv2p)
